# Enantio- and diastereoselective diarylmethylation of 1,3-dicarbonyl compounds†

**DOI:** 10.1039/d0sc00142b

**Published:** 2020-05-25

**Authors:** Xin Li, Songtao He, Qiuling Song

**Affiliations:** Institute of Next Generation Matter Transformation, College of Materials Science & Engineering, Huaqiao University 668 Jimei Blvd Xiamen Fujian China qsong@hqu.edu.cn

## Abstract

A Cu-catalyzed enantio- and diastereoselective diarylmethylation of 1,3-dicarbonyl compounds is described. It is a successful example of constructing all-carbon quaternary stereocenters from 3 °C–H nucleophiles, a challenging topic in synthetic chemistry. In the present work, two contiguous stereocenters are constructed with high levels of stereoselectivity and atom economy. The broad scope of 1,3-dicarbonyl nucleophiles and the tolerance of a wide range of functional groups make this protocol of great importance in the synthesis of chiral diarylmethane compounds.

## Abstract 2

A Cu-catalyzed enantio- and diastereoselective diarylmethylation of 1,3-dicarbonyl compounds is described. Two contiguous stereocenters including one all-carbon quaternary stereocenter are constructed with good stereoselectivity and atom economy.

## Graphical abstract

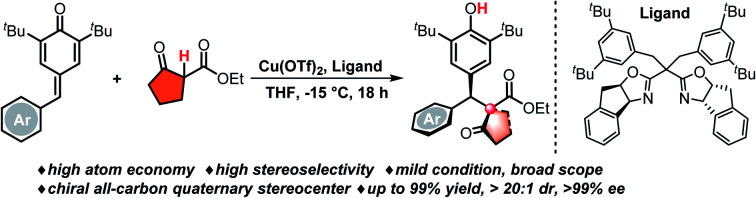

Chiral diarylmethanes are ubiquitous in numerous natural products, medicinal molecules and bioactive compounds.^1^ As a consequence, many elegant strategies have been developed to construct those structures. Among them, the asymmetric addition of an appropriate nucleophile to an aryl substituted quinone methide is considered as a direct and efficient access to chiral diarylmethanes.^2^ During the past decade, with the development of many new kinds of nucleophiles and new modes of asymmetric catalytic systems, a variety of C–C^3^ and C–X^4^ bonds have been forged to furnish the target chiral diarylmethanes. Among those strategies developed, employing C–H nucleophiles is regarded as an ideal method because 100% atom economy can be achieved in that transformation. For instance, in 2013, Fan reported a beautiful work, in which malonic esters incorporated with *para*-quinone methides (*p*-QMs) enantioselectively under a chiral phase-transfer catalytic system (Scheme 1, route A).^3*a*^ Soon after, Jørgensen demonstrated a new organocatalytic concept to construct two contiguous stereocenters enantio- and diastereoselectively using their iconic chiral secondary amine catalysts (Scheme 1, route B).^3*b*^ Although great achievements have been made, the next progress in this field, *i.e.* employing 3° C–H nucleophiles to construct a more challenging all-carbon quaternary stereocenter^5^ was proven difficult, which may be attributed to the increased steric hindrance and the decreased acidity of the 3° C–H nucleophiles. To date, successful nucleophiles were restricted to oxindoles.^6^ Therefore, an efficient protocol which highly stereoselectively affords the two stereocenters with a broad 3° C–H nucleophiles scope is demanded. In this communication, we address this problem by employing a series of 1,3-dicarbonyl compounds under a copper-chiral bisoxazoline catalytic system (Scheme 1, route C).

**Scheme 1:**
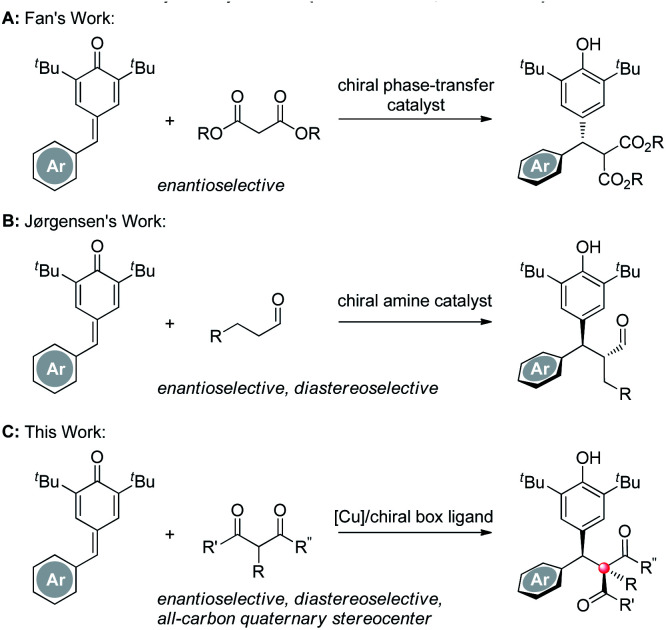
Enantioselective diarylmethylation of 1,3-dicarbonyl compounds.

We commenced our investigation by using *p*-QM **1a** and 1,3-dicarbonyl compound **2a** as model substrates. After preliminary screening, a combination of 10 mol% of Cu(OTf)_2_ and 12 mol% of a 1-amino-2-indanol derived bisoxazoline ligand **L1** catalyzed the reaction efficiently, affording the desired product with 98% isolated yield, 4.6 : 1 dr and 78% ee at 0 °C (Table 1, entry 1).^7^ Based on that result, a structural modification of the ligands family was carried out to improve the stereoselectivity of the reaction (entries 2–16). Gratifyingly, among a small library of 15 synthesized ligands, a 3,5-di-*tert*-butyl-benzyl substituted ligand **L8** gave an excellent outcome (98% isolated yield, 11.5 : 1 dr and 94% ee, entry 9). Meanwhile, those data showed that steric factor plays a crucial role in the performance of the ligands. For example, increasing the steric hindrance on the benzene ring of indanol resulted in a dramatic decrease on yield, and slightly decreases on dr and ee (entry 2 *vs.* entry 5). Besides, ligands bearing 4-substituted benzyl groups all gave much lower yields and poorer diastereoselectivities than their 3,5-disubstituted counterparts (entries 9–11 *vs.* entries 13–15). Further optimization showed that a higher stereoselectivity can be achieved when the reaction proceeded at −15 °C (entry 17).^8^

**Table 1:** Screening of ligands^a^

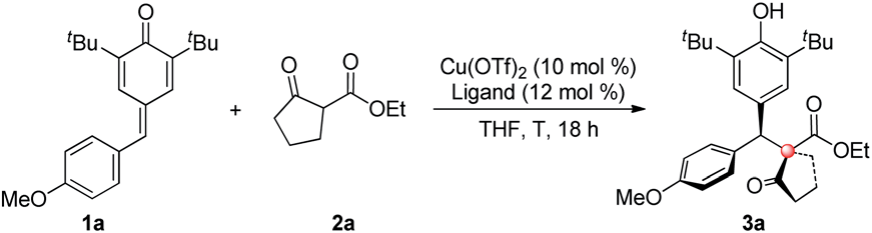					
Entry	Ligand	*T* [°C]	Yield^b^ [%]	dr^c^	ee^d^ [%]
1	**L1**	0	98	4.6 : 1	78
2	**L1**	−10	98	4.7 : 1	81
3	**L2**	−10	92	4.4 : 1	83
4	**L3**	−10	90	4.0 : 1	79
5	**L4**	−10	37	4.6 : 1	75
6	**L5**	−10	24	3.8 : 1	73
7	**L6**	−10	96	4.2 : 1	83
8	**L7**	−10	95	3.6 : 1	77
9	**L8**	−10	98	11.5 : 1	94
10	**L9**	−10	98	6.7 : 1	86
11	**L10**	−10	69	5.7 : 1	88
12	**L11**	−10	97	5.3 : 1	85
13	**L12**	−10	48	3.4 : 1	83
14	**L13**	−10	40	4.3 : 1	74
15	**L14**	−10	56	4.2 : 1	70
16	**L15**	−10	25	3.6 : 1	90
17	**L8**	−15	98	14.6 : 1	98
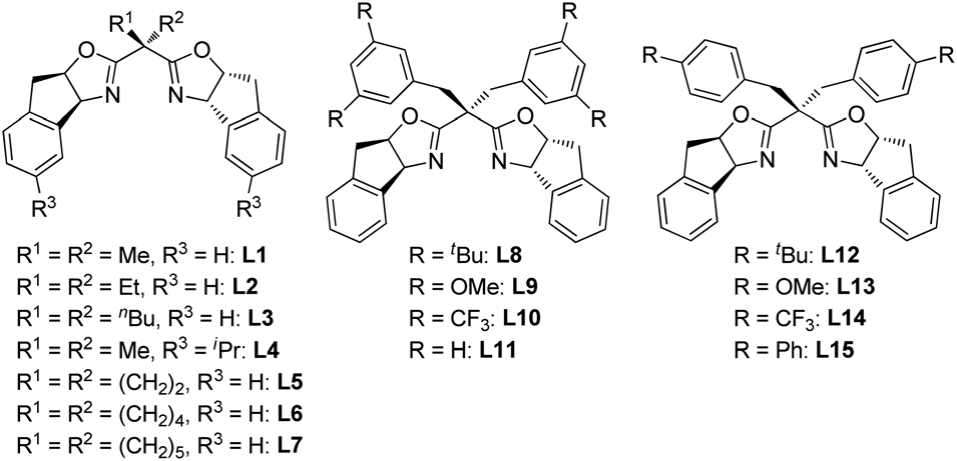					

a Reaction conditions: *p*-quinone methide (0.05 mmol), ethyl 2-oxocyclopentanecarboxylate (0.075 mmol), Cu(OTf)_2_ (10 mol%), ligand (12 mol%), THF (1.0 mL), 18 h.

b Yield of the isolated product.

c Determined by ^1^H-NMR of the crude reaction mixture.

d Determined by HPLC analysis and referred to the major diastereoisomer.

After the optimization works, a series of 1,3-dicarbonyl compounds were submitted to the standard reaction conditions to test the generalizability of the reaction toward C–H nucleophiles (Table 2). For 2 °C–H nucleophiles, ethyl 3-oxo-3-phenylpropanoate derivatives all gave good results with respect to yields, dr and ee's (**3b–m**). The electronic factor of the benzene rings has little influence on the reactivity and almost no influence on the stereoselectivity. For example, substrates bearing electron-withdrawing substituents on the benzene rings reacted more slowly under the same condition, thus resulting in lower conversions and yields (**3c–g**, **3k**). Nevertheless, both electron-donating and electron-withdrawing substituents gave excellent stereoselectivities (up to >20 : 1 dr, up to >99% ee). In contrast, steric factor plays an important role in the reactivity and stereoselectivity of the substrates. For instance, *m*-bromo substituted substrate (**3l**) required a higher reaction temperature (ambient temperature)^9^ to afford the product but with a poorer dr and ee than those bearing smaller substituents such as methyl (**3j**) or chlorine atom (**3k**). *O*-Methyl (**3m**) also required a higher reaction temperature than *m*- and *p*-methyl (**3h** and **3j**). The size of either the groups connected to the carbonyl or the ester groups determines the outcomes of the reactions. For example, with the increase of the size of ester groups, the reactivity and stereoselectivity of the nucleophiles both decreased (**3n–p**). Although a smaller cyclopropyl substituted ethyl acetoacetate gave poor diastereoselectivity and moderate enantioselectivity (**3q**), larger cyclopentyl and cyclohexyl substituents afforded the target products in good yields and excellent stereoselectivities (**3r** and **3s**). Heterocyclic substituents are compatible with the reaction (**3t** and **3u**).

**Table 2:** Scope of 1,3-dicarbonyl nucleophiles^a^^,^^b^

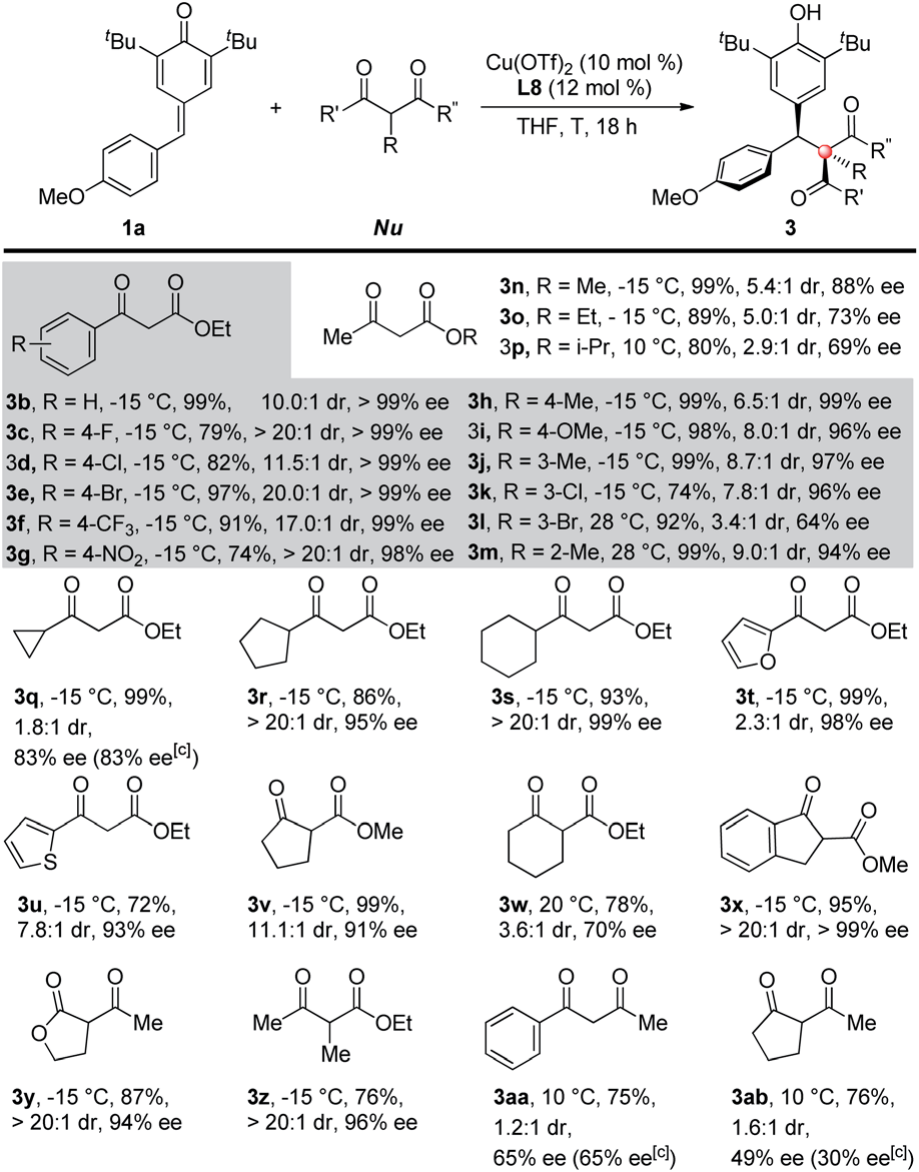

a Reaction conditions: *p*-quinone methide (0.05 mmol), 1,3-dicarbonyl compound (0.075 mmol), Cu(OTf)_2_ (10 mol%), ligand (12 mol%), THF (1.0 mL), 18 h.

b Isolated yield.

c ee's of minor diastereoisomers.

More importantly, a diverse variety of 3° C–H nucleophiles exhibited equal reactivities and stereoselectivities. Substrates that differed in aromatic, aliphatic or acyclic carbon skeletons, both esters and lactones, all constructed the desired all-carbon quaternary stereocenters in high efficiencies (**3v–z**). Interestingly, the existence of an ester subunit in the substrates was proven to be vital to both reactivity and stereoselectivity. For example, 1,3-diketones, both 2° and 3° C–H nucleophiles (**3aa** and **3ab**), gave less yields and dramatic drops on the stereoselectivities in contrast to β-ketoesters. The different coordination properties between esters and carbonyls may account for the phenomenon.

Next, the scope of *p*-QMs was investigated (Table 3). The data revealed that both the electronic and steric factors are crucial to the reactivity and stereoselectivity of the *p*-QMs. In general, *p*-QMs bearing electron-poor aryls are less reactive towards the nucleophiles. They required an elevated temperature in order to provide the target products. However, most of them still maintained good yields and stereoselectivities (**4a–h**). In contrast, other substrates bearing electron-rich aryls have comparable reactivity with **1a**. They reacted with the nucleophiles at lower temperatures with good yields and stereoselectivities (**4i–o**). For a *p*-QM bearing an electron-neutral phenyl, interestingly, both the yield and stereoselectivity were moderate (**4p**), which indicated that electronic factor is not the only element that determines the outcome of the reaction. And once more, the steric factor played a crucial role in the reaction. For example, more sterically hindered *o*- and *m*-methoxy substituted substrates required higher reaction temperatures while gave modest yields and even poorer ee's (**4q** and **4r**). *p*-QMs bearing di-substituted aryls afforded the target product with moderate to good stereoselectivities (**4s–u**), which was attributed to the increased steric hindrance. Fused rings containing substrates also proceeded well (**4v–x**). As anticipated, more steric hindered 1-naphthyl (**4v**) required a higher reaction temperature but afforded lower dr and ee than its 2-naphthyl counterpart (**4w**). Again, heterocyclic rings are compatible with the reaction (**4y–ab**).^10^ The relative and absolute configuration of **4z** was unequivocally determined by single crystal X-ray diffraction (Scheme 2).^11^

**Table 3:** Scope of *p*-quinone methides^a^^,^^b^

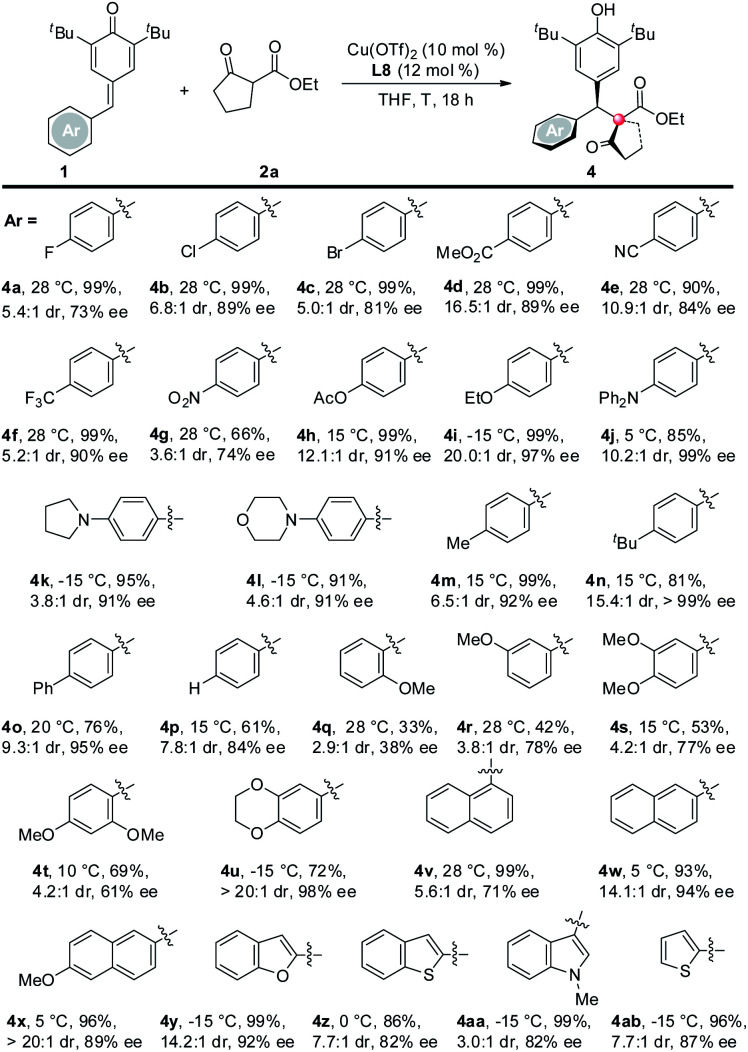

a Reaction conditions: *p*-quinone methide (0.05 mmol), ethyl 2-oxocyclopentanecarboxylate (0.075 mmol), Cu(OTf)_2_ (10 mol%), ligand (12 mol%), THF (1.0 mL), 18 h.

b Isolated yield.

**Scheme 2:**
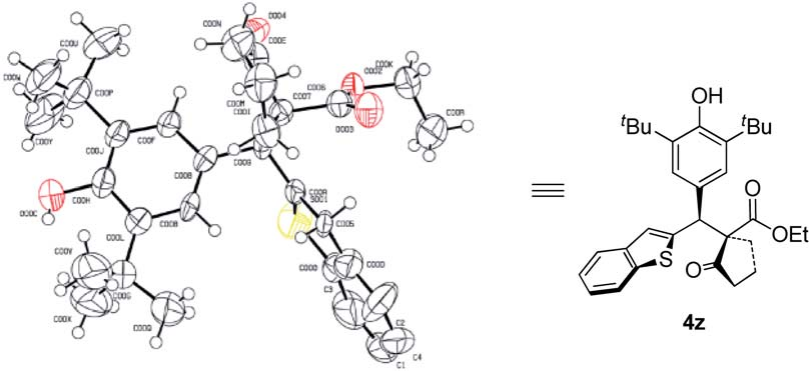
X-ray crystal structure of compound **4z**.

To understand the substituents' influence on the reactivity and stereoselectivity of the *p*-QMs, a series of substrates with different substituents on the *p*-QM skeletons were tested. First, the aromatic rings on the C-6 position of the *p*-QMs are necessary to maintain a high level of stereoselectivity, for a C-6 methyl substituted substrate suffered both low reactivity and poor stereoselectivity (Scheme 3A). Besides, the substituents on the C-3 position are important as well. For methyl, the smallest substituent tested, it brings a poor discrimination between the Si- and Re-faces of the *p*-QM planes, thus resulting in the formation of unselective products (Scheme 3B, **6a**). When a 3-methyl-3'-*tert*-butyl substrate was employed, the stereoselectivity improved but was still moderate (**6b**). Isopropyl, which has a smaller size than *tert*-butyl, afforded the product slightly less stereoselectively than the *tert*-butyl did (**6c**). Interestingly, a bigger TMS group exhibited modestly better steric control (**6d**). Unlike alkyl substituents, C-3 aryl rings substituents don't obey the above-mentioned “size law”, showing a deleterious effect on the reactivity and stereoselectivity (**6e**). On the basis of all of the experimental results, the high stereoselectivity of this reaction may owe to the combined interaction among the chiral catalyst, *p*-QMs, and the nucleophiles. Thus, a possible catalytic cycle and a plausible transition state which determines the absolute configuration of the products are proposed (Scheme 3C). Once the chiral Lewis acid catalyst **I** was generated from Cu(OTf)_2_ and the box ligand, the subsequent ligand exchange and deprotonation of 1,3-dicarbonyl nucleophiles formed an intermediate **II**, which then underwent an asymmetric 1,6-addition with the *p*-QMs to afford the final chiral product with high levels of entioselectivity and diastereoselectivity. The presumed transition state rationalizes the observed absolute stereochemical outcome. In this model, the chiral catalyst determines a Si-face attack on the *p*-QM planes, the ester groups of nucleophiles are opposite the aryls of *p*-QMs to minimize the steric hindrance or provide additional π-stacking interactions (for **3b–m**, **3x**).

**Scheme 3:**
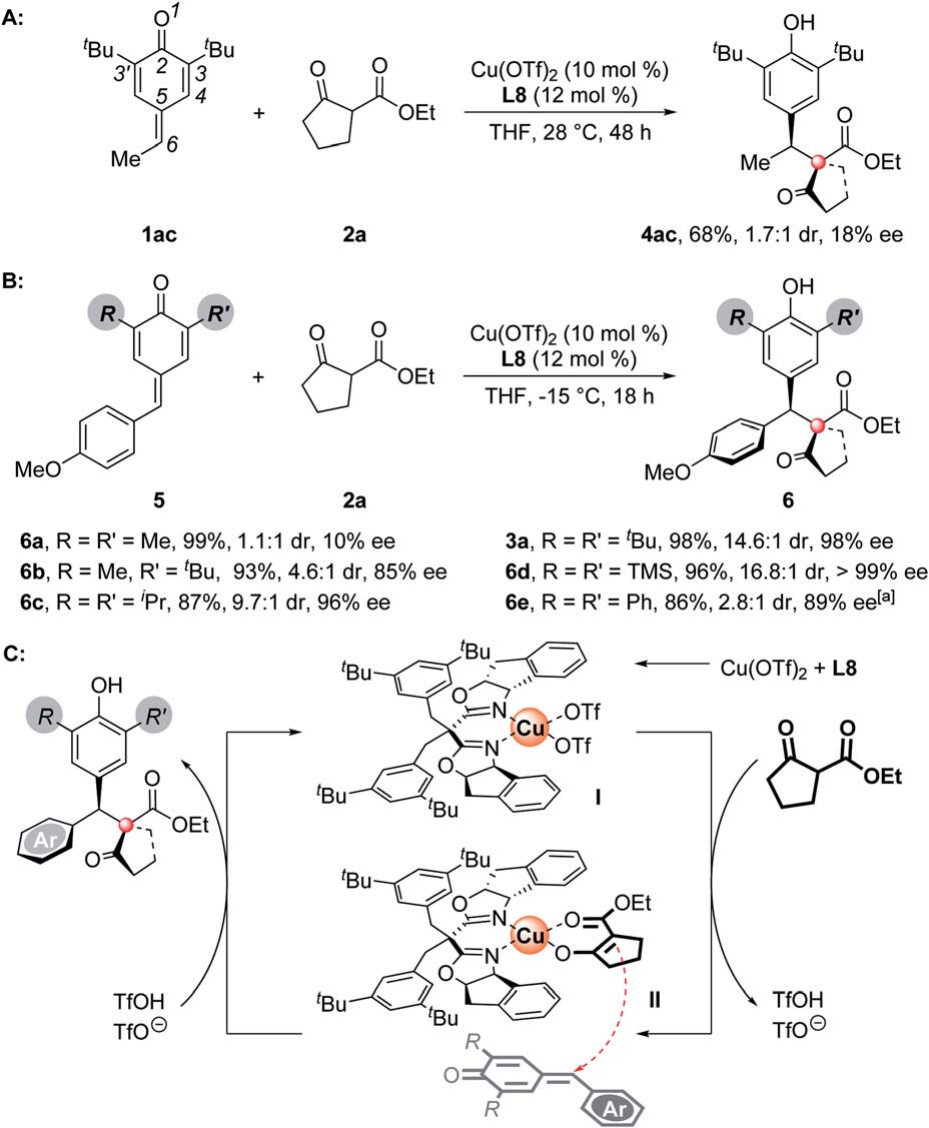
Substituents' influence on stereoselectivity and proposed mechanism. ^*a*^Reaction conducted at −5 °C.

The reaction can be carried out in gram-scale using 3 mol% catalyst loading without the destruction on yields and ee's, only at the expense of prolonged reaction times (Scheme 4A). Although these synthesized products are unstable under the acidic AlCl_3_-induced de-*tert*-butylation process,^12^ after reducing the carbonyls to either hydroxyls (**3a**) or methylene groups (**3b**),^13^ the *tert*-butyls on the aryl rings can be readily removed by AlCl_3_ without any loss of the optical purities (Scheme 4B).

**Scheme 4:**
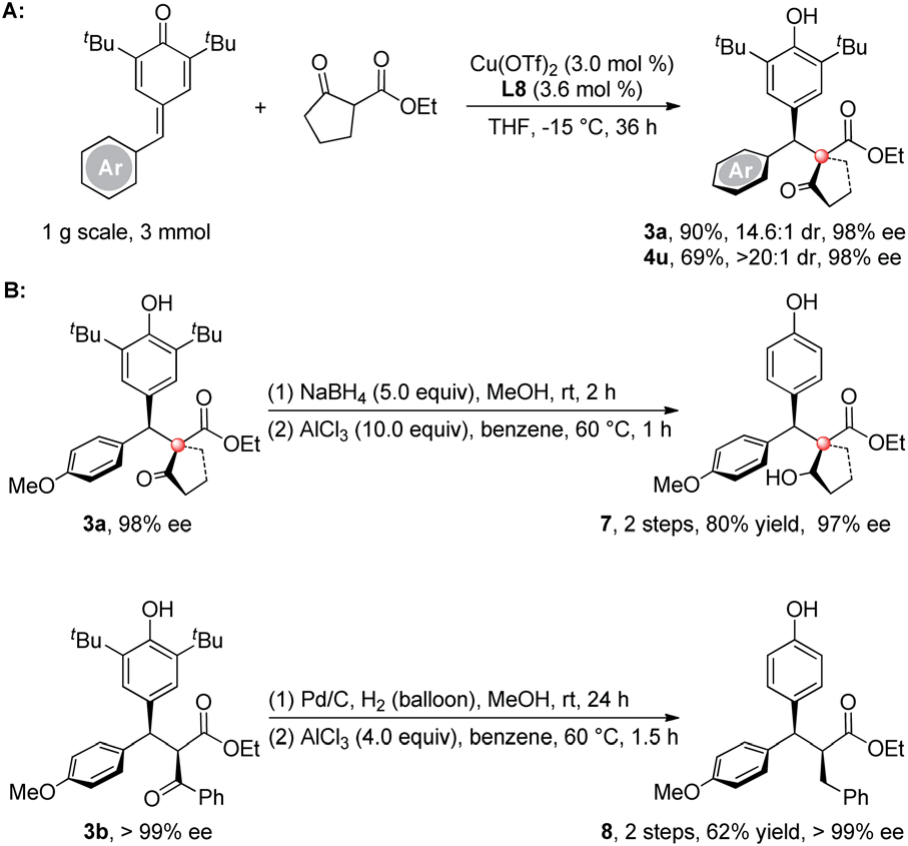
Gram-scale synthesis and de-*tert*-butylation of the products.

## Conclusions

In conclusion, we have developed a simple but efficient protocol to construct two contiguous stereocenters including one all-carbon quaternary stereocenter from a broad scope of *p*-QMs and 1,3-dicarbonyl compounds with high atom economy. Good yields and excellent stereoselectivities are achieved in most cases. The application of the copper-chiral bisoxazoline catalytic system to other asymmetric synthesis is under investigated in our group.

## Conflicts of interest

There are no conflicts to declare.

## Supplementary Material

SC-011-D0SC00142B-s001

SC-011-D0SC00142B-s002
