# Supplementary material for: Enantio- and diastereoselective diarylmethylation of 1,3-dicarbonyl compounds
Source: Chem Sci. 2020 May 25;11(23):5969–73. doi: 10.1039/d0sc00142b (PMC8159382; doi:10.1039/d0sc00142b)
Supplement: SC-011-D0SC00142B-s001 [file SC-011-D0SC00142B-s001.pdf]

Electronic Supplementary Material (ESI) for Chemical Science.
This journal is © The Royal Society of Chemistry 2020

Enantio- and Diastereoselective Diarylmethylation of 1,3-Dicarbonyl Compounds

Xin Li, Songtao He, and Qiuling Song *

Institute of Next Generation Matter Transformation, College of Materials Science & Engineering, Huaqiao University, 668 Jimei Blvd, Xiamen, Fujian, China

*qsong@hqu.edu.cn

Supporting Information

Table of Contents

I. General Information 1

II. Preparation of Materials 2

2.1 Synthesis of *p*-Quinone Methide Substrates 2

2.2 Synthesis of L1-L3, L5, L8-L15 5

2.3 Synthesis of L4 7

2.4 Synthesis of L6, L7 8

III. Screening of Reaction Conditions 9

IV. Diarylmethylation of 1,3-Dicarbonyl Compounds 10

V. Gram-Scale Synthesis 32

VI. Synthesis of Compounds 7 and 8 32

6.1 Synthesis of Compound 7 32

6.2 Synthesis of Compound 8 33

VII. X-Ray Crystallographic Analysis 35

7.1 Relative Configuration of 3g 35

7.2 Relative Configuration of 3v 37

7.3 Absolute Configuration of 4z 39

VIII. NMR Spectra and HPLC Spectra 40

I. General Information

All air or moisture sensitive reactions were conducted in oven-dried glassware under nitrogen atmosphere using dry solvents. Anhydrous tetrahydrofuran was freshly distilled from sodium-benzophenone. Anhydrous benzene and toluene were freshly distilled from sodium. Unless otherwise stated, chemicals and reagents were used as

received. Flash column chromatography was performed over silica gel (200-300 mesh) purchased from Qindao Bangkai Co., China. ^{1}H and ^{13}C NMR spectra were recorded on a Bruker AV 500 MHz NMR spectrometer using residue solvent peaks as an internal standard (^{1}H NMR: $CDCl_3$ at 7.26 ppm, ^{13}C NMR: $CDCl_3$ at 77.0 ppm). HRMS were recorded on an Agilent 6545 Q-TOF LC/MS instrument with electrospray ionization (ESI) technique. X-ray diffraction was performed on a Gemini E X-ray single crystal diffractometer. Analytical chiral HPLC was performed on an Agilent 1600 Infinity instrument with Daicel Chiralcel OD-H column, or Daicel Chiralpak AD-H, IA-3, IC-3 columns. Optical rotations were measured on an Aton Paar MCP 150 polarimeter.

II. Preparation of Materials

2.1 Synthesis of *p*-Quinone Methide Substrates

p-Quinone methide substrates **1a-1ab, 5b, 5c,** and **5e** were synthesized according to the literature reported procedure.[1]

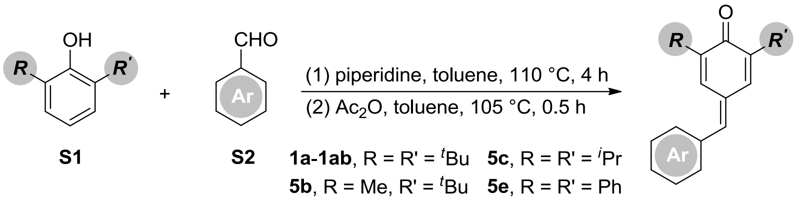

The characterization data of unreported compounds are shown as follows; the other quinone methides are known compounds.

4-((3,5-di-*tert*-butyl-4-oxocyclohexa-2,5-dien-1-ylidene)methyl)phenyl acetate (1h)

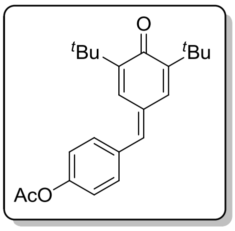

(yellow solid)

^{1}H NMR (500 MHz, $CDCl_3$) δ 7.51 (d, *J* = 2.4 Hz, 1H), 7.50 (d, *J* = 8.6 Hz, 2H), 7.22 (d, *J* = 8.6 Hz, 2H), 7.16 (s, 1H), 7.02 (d, *J* = 2.4 Hz, 1H), 2.36 (s, 3H), 1.35 (s, 9H), 1.32 (s, 9H).

^{13}C NMR (125 MHz, $CDCl_3$) δ 186.5, 169.1, 151.2, 149.7, 147.9, 141.1, 135.0, 133.6, 132.1, 131.4, 127.4, 122.0, 35.5, 35.0, 29.6, 29.5, 21.1.

HRMS: (ESI$^+$) $[M+Na]^+$ calcd for $C_{23}H_{28}O_3Na$: 375.1931, found: 353.2111.

2,6-di-*tert*-butyl-4-(4-ethoxybenzylidene)cyclohexa-2,5-dienone (1i) (yellow solid)

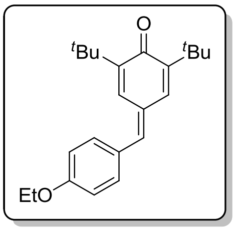

^{1}H NMR (500 MHz, $CDCl_3$) δ 7.58 (d, *J* = 2.4 Hz, 1H), 7.46 (d, *J* = 8.7 Hz, 2H), 7.15 (s, 1H), 7.03 (d, *J* = 2.4 Hz, 1H), 6.99 (d, *J* = 8.7 Hz, 2H), 4.13 (q, *J* = 7.0 Hz, 2H), 1.48 (t, *J* = 7.0 Hz, 3H), 1.36 (s, 9H), 1.34 (s, 9H).

^{13}C NMR (125 MHz, $CDCl_3$) δ 186.5, 160.0, 149.0, 147.2, 142.7, 135.4, 132.2, 130.5, 128.5, 127.8, 114.9, 63.7, 35.4, 35.0, 29.6, 29.5, 14.8.

HRMS: (ESI$^+$) $[M+H]^+$ calcd for $C_{23}H_{31}O_2$: 339.2319, found: 339.2325.

2,6-di-*tert*-butyl-4-(4-(diphenylamino)benzylidene)cyclohexa-2,5-dienone (1j)
(yellow solid)

[1] D. Richter, N. Hampel, T. Singer, A. R. Ofial, H. Mayr, *Eur. J. Org. Chem.* **2009,** *19*, 3203

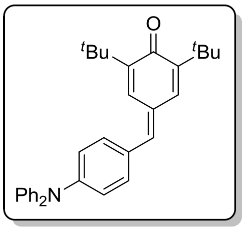

^{1}H NMR (500 MHz, $CDCl_3$) δ 7.64 (d, *J* = 2.4 Hz, 1H), 7.38 (d, *J* = 8.7 Hz, 2H), 7.37 – 7.31 (m, 4H), 7.23 – 7.18 (m, 4H), 7.15 (tt, *J* = 7.5, 1.2 Hz, 2H), 7.09 (d, *J* = 2.7 Hz, 2H), 7.07 (s, 1H), 7.02 (d, *J* = 2.4 Hz, 1H), 1.36 (s, 9H), 1.34 (s, 9H).

^{13}C NMR (125 MHz, $CDCl_3$) δ 186.4, 149.1, 148.8, 147.0, 146.8, 142.6, 135.5, 131.9, 130.2, 129.6, 128.9, 127.7, 125.7, 124.3, 121.2, 35.5, 35.0, 29.62, 29.55.

HRMS: (ESI^+) $[M+Na]^+$ calcd for $C_{33}H_{35}NONa$: 484.2611, found: 484.2615.

2,6-di-*tert*-butyl-4-(4-morpholinobenzylidene)cyclohexa-2,5-dienone (1k) (yellow solid)

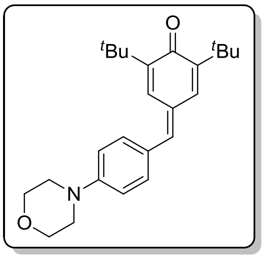

^{1}H NMR (500 MHz, $CDCl_3$) δ 7.63 (d, *J* = 2.4 Hz, 1H), 7.47 (d, *J* = 8.8 Hz, 2H), 7.12 (s, 1H), 7.02 (d, *J* = 2.4 Hz, 1H), 6.97 (d, *J* = 8.8 Hz, 2H), 3.98 – 3.84 (m, 4H), 3.40 – 3.26 (m, 4H), 1.36 (s, 9H), 1.35 (s, 9H).

^{13}C NMR (125 MHz, $CDCl_3$) δ 186.4, 151.6, 148.7, 146.9, 143.0, 135.5, 132.3, 129.8, 127.9, 127.0, 114.6, 66.7, 48.0, 35.4, 34.9, 29.62, 29.55.

HRMS: (ESI^+) $[M+H]^+$ calcd for $C_{25}H_{34}NO_2$: 380.2584, found: 380.2590.

2,6-di-*tert*-butyl-4-(2,4-dimethoxybenzylidene)cyclohexa-2,5-dienone (1t) (yellow solid)

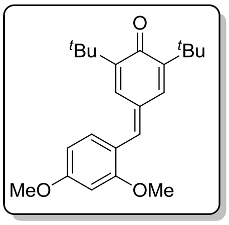

^{1}H NMR (500 MHz, $CDCl_3$) δ 7.50 (d, *J* = 2.4 Hz, 1H), 7.39 (s, 1H), 7.38 (d, *J* = 9.6 Hz, 1H), 7.08 (d, *J* = 2.4 Hz, 1H), 6.61 (dd, *J* = 8.5, 2.4 Hz, 1H), 6.53 (d, *J* = 2.4 Hz, 1H), 3.90 (s, 6H), 1.36 (s, 9H), 1.32 (s, 9H).

^{13}C NMR (125 MHz, $CDCl_3$) δ 186.6, 162.5, 159.8, 148.6, 146.9, 138.6, 135.5, 132.8, 130.3, 128.3, 118.2, 105.0, 98.6, 55.6, 55.5, 35.4, 35.0, 29.6, 29.5.

HRMS: (ESI^+) $[M+H]^+$ calcd for $C_{23}H_{31}O_3$: 355.2268, found: 355.2266.

2,6-di-*tert*-butyl-4-((2,3-dihydrobenzo[*b*][1,4]dioxin-6-yl)methylene)cyclohexa-2,5-dienone (1u) (yellow solid)

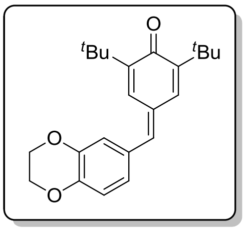

^{1}H NMR (500 MHz, $CDCl_3$) δ 7.58 (d, *J* = 2.4 Hz, 1H), 7.08 (s, 1H), 7.05 (d, *J* = 2.0 Hz, 1H), 7.04 – 6.98 (m, 2H), 6.96 (d, *J* = 8.3 Hz, 1H), 4.37 – 4.33 (m, 2H), 4.33 – 4.30 (m, 2H), 1.35 (s, 9H), 1.34 (s, 9H).

^{13}C NMR (125 MHz, $CDCl_3$) δ 186.5, 149.1, 147.3, 144.9, 143.8, 142.3, 135.3, 130.9, 129.6, 127.7, 124.4, 119.3, 117.7, 64.6, 64.3, 35.5, 35.0, 29.6, 29.5.

HRMS: (ESI^+) $[M+Na]^+$ calcd for $C_{23}H_{28}O_3Na$: 375.1931, found: 375.1937.

2,6-di-*tert*-butyl-4-((6-methoxynaphthalen-2-yl)methylene)cyclohexa-2,5-dienone (1x) (yellow solid)

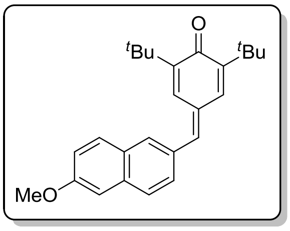

^{1}H NMR (500 MHz, $CDCl_3$) δ 7.90 (d, *J* = 1.6 Hz, 1H), 7.81 (dd, *J* = 8.7, 6.0 Hz, 2H), 7.68 (d, *J* = 2.4 Hz, 1H), 7.58 (dd, *J* = 8.7, 1.6 Hz, 1H), 7.34 (s, 1H), 7.23 (dd, *J* = 8.9, 2.5 Hz, 1H), 7.18 (d, *J* = 2.5 Hz, 1H), 7.09 (d, *J* = 2.4 Hz, 1H), 3.98 (s, 3H), 1.38 (s, 9H), 1.35 (s, 9H).

^{13}C NMR (125 MHz, $CDCl_3$) δ 186.5, 159.0, 149.3, 147.6, 142.9, 135.2, 134.8, 131.6, 131.4, 130.7, 130.1, 128.8, 128.0, 128.0, 127.3, 119.7, 105.9, 55.4, 35.5, 35.0, 29.60, 29.56.

HRMS: (ESI^+) $[M+H]^+$ calcd for $C_{26}H_{31}O_2$: 375.2319, found: 375.2317.

4-(benzofuran-2-ylmethylene)-2,6-di-*tert*-butylcyclohexa-2,5-dienone (1y)(yellow solid)

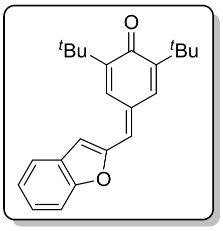

^{1}H NMR (500 MHz, $CDCl_3$) δ 8.39 (d, *J* = 2.4 Hz, 1H), 7.64 (dt, *J* = 7.8, 1.0 Hz, 1H), 7.57 – 7.51 (m, 1H), 7.41 (ddd, *J* = 8.4, 7.2, 1.3 Hz, 1H), 7.36 – 7.24 (m, 1H), 7.05 (s, 1H), 6.99 (d, *J* = 2.4 Hz, 1H), 6.81 (s, 1H), 1.43 (s, 9H), 1.36 (s, 9H).

^{13}C NMR (125 MHz, $CDCl_3$) δ 186.1, 156.4, 154.4, 149.5, 148.4, 134.6, 131.3, 128.5, 128.1, 126.8, 125.9, 123.7, 121.8, 113.9, 111.4, 35.7, 35.1, 29.63, 29.59.

HRMS: (ESI$^+$) [M+H]$^+$ calcd for $C_{23}H_{27}O_2$: 335.2006, found: 335.2002.

4-(benzo[*b*]thiophen-2-ylmethylene)-2,6-di-*tert*-butylcyclohexa-2,5-dienone (1z)

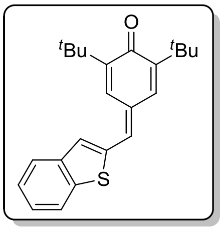

(yellow solid)

^{1}H NMR (500 MHz, $CDCl_3$) δ 7.96 (d, *J* = 2.5 Hz, 1H), 7.90 – 7.86 (m, 1H), 7.86 – 7.81 (m, 1H), 7.57 (s, 1H), 7.46 – 7.36 (m, 2H), 7.28 (s, 1H), 7.02 (d, *J* = 2.5 Hz, 1H), 1.43 (s, 9H), 1.36 (s, 9H).

^{13}C NMR (125 MHz, $CDCl_3$) δ 186.1, 149.7, 148.0, 142.3, 139.1, 138.7, 135.0, 134.3, 131.1, 130.9, 127.0, 126.4, 125.2, 124.5, 122.3, 35.8, 35.1, 29.7, 29.6.

HRMS: (ESI$^+$) [M+H]$^+$ calcd for $C_{23}H_{27}OS$: 351.1777, found: 351.1772.

2,6-di-*tert*-butyl-4-((1-methyl-1*H*-indol-3-yl)methylene)cyclohexa-2,5-dienone (1aa)

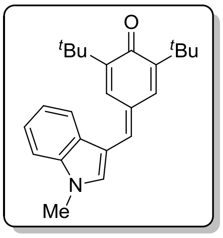

(yellow solid)

^{1}H NMR (500 MHz, $CDCl_3$) δ 7.83 (d, *J* = 7.9 Hz, 1H), 7.74 (d, *J* = 2.4 Hz, 1H), 7.50 (d, *J* = 0.8 Hz, 1H), 7.41 (d, *J* = 1.1 Hz, 1H), 7.40 – 7.36 (m, 1H), 7.31 (ddd, *J* = 8.0, 6.8, 1.3 Hz, 1H), 7.14 (d, *J* = 2.4 Hz, 1H), 3.94 (s, 3H), 1.40 (s, 9H), 1.39 (s, 9H).

^{13}C NMR (125 MHz, $CDCl_3$) δ 186.4, 148.1, 146.0, 137.1, 135.4, 134.7, 131.4, 128.1, 128.1, 128.0, 123.4, 121.4, 119.2, 113.0, 109.9, 35.4, 34.9, 33.6, 29.7, 29.6.

HRMS: (ESI$^+$) [M+H]$^+$ calcd for $C_{24}H_{30}NO$: 348.2322, found: 348.2322.

2-(*tert*-butyl)-4-(4-methoxybenzylidene)-6-methylcyclohexa-2,5-dienone (5b) (1.27:1 dr)

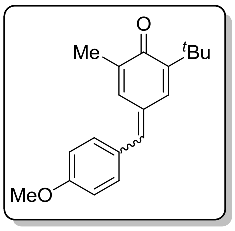

(yellow solid)

^{1}H NMR (500 MHz, $CDCl_3$) δ 7.65 (d, *J* = 2.6 Hz, 1H), 7.48 (d, *J* = 8.7 Hz, 2H), 7.17 (s, 1H), 7.07 (d, *J* = 2.6 Hz, 1H), 7.00 (d, *J* = 8.7 Hz, 2H), 3.89 (s, 3H), 2.08 (s, 3H), 1.36 (s, 9H) (major isomer).

^{13}C NMR (125 MHz, $CDCl_3$) δ 186.6, 160.7, 145.7, 143.1, 137.6, 137.1, 132.3, 130.1, 129.1, 128.5, 114.4, 55.4, 34.7, 29.3, 17.2 (major isomer).

HRMS: (ESI$^+$) [M+Na]$^+$ calcd for $C_{19}H_{22}O_2Na$: 305.1512, found: 305.1521.

p-Quinone methide **1ac** was synthesized according to the literature reported procedure, and it is a known compound.[2]

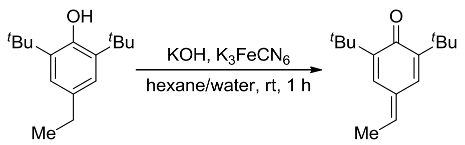

S3 **1ac**

[2] Z.-Q. Liu, P.-S. You, L.-D. Zhang, D.-Q. Liu, S.-S. Liu, X.-Y. Guan, *Molecules* **2020**, *25*, 539

p-Quinone methide **5a** was synthesized according to the literature reported procedure, and it is a known compound.[3]

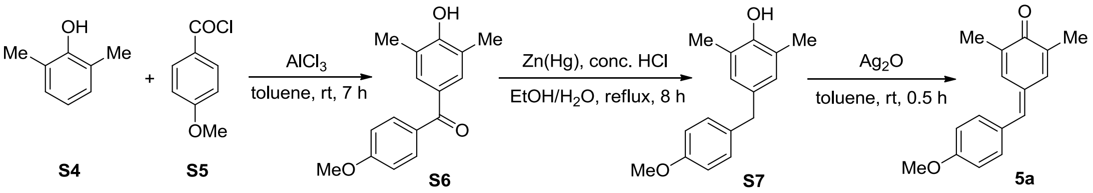

p-Quinone methide **5d** was synthesized according to the literature reported procedure.[4]

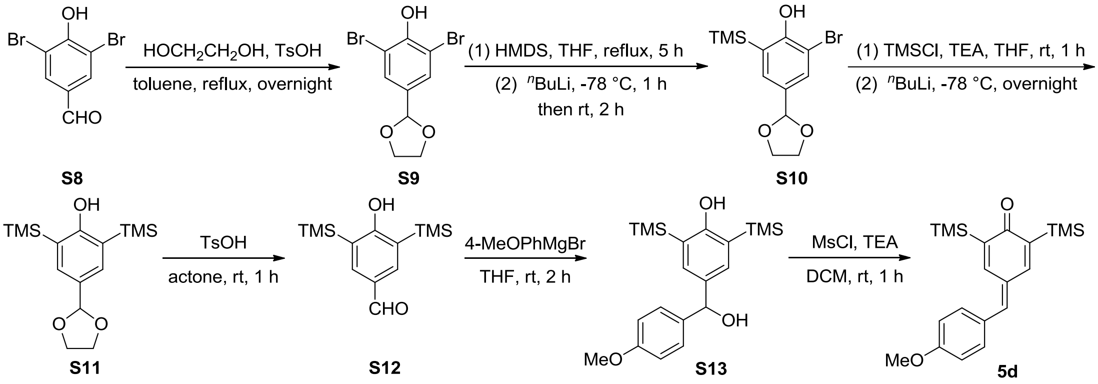

4-(4-methoxybenzylidene)-2,6-bis(trimethylsilyl)cyclohexa-2,5-dienone (5d)

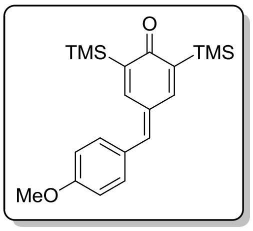

(yellow solid)

^{1}H NMR (500 MHz, $CDCl_3$) δ 7.92 (d, *J* = 2.0 Hz, 1H), 7.50 (d, *J* = 8.7 Hz, 2H), 7.36 (d, *J* = 2.0 Hz, 1H), 7.23 (s, 1H), 7.02 (d, *J* = 8.7 Hz, 1H), 3.91 (s, 3H), 0.27 (s, 9H), 0.25 (s, 9H).

^{13}C NMR (125 MHz, $CDCl_3$) δ 193.1, 161.1, 149.7, 144.2, 142.9, 141.8, 140.7, 132.6, 129.8, 128.1, 114.5, 55.4, -1.4.

HRMS: (ESI^+) $[M+H]^+$ calcd for $C_{20}H_{29}O_2Si_2$: 357.1701, found: 357.1705.

2.2 Synthesis of L1-L3, L5, L8-L15

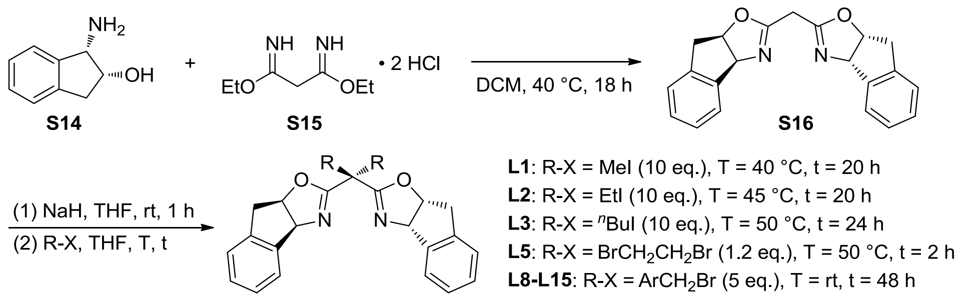

Compound **S16** was synthesized according to the literature reported procedure.[5]

To a solution of **S16** in anhydrous THF was added NaH (7.0 equiv) in portions at rt. The resulting mixture was stirred at rt for 1 hour, and then the corresponding halide was

---

[3] T. Uno, H. Ohta, A. Yamane, M. Kubo, T. Itoh, *Journal of Polymer Science, Part A: Polymer Chemistry,* **2015**, *53*, 437

[4] L. Roisera, M. Wasera, *Org. Lett.* **2017**, *19*, 2338

[5] J. L. Hofstra, A. H. Cherney, C. M. Ordner, S. E. Reisman, *J. Am. Chem. Soc.* **2018**, *140*, 139

added. The mixture was continued to stir at the temperature indicated. After completion, the reaction was quenched with saturated NH_4Cl and extracted with CH_2Cl_2. The combined organic layers were dried with Na_2SO_4, filtered, and concentrated under reduced pressure. The residue was purified by silica gel column chromatography (petroleum ether/acetone) to afford the corresponding pure product.

Ligands **L1**, **L5**, **L8**, **L9**, **L11**, and **L12** are known compounds.

(3a*S*,3a'*S*,8a*R*,8a'*R*)-2,2'-(pentane-3,3-diyl)bis(8,8a-dihydro-3a*H*-indeno[1,2-*d*]oxazole) (L2) (white solid)

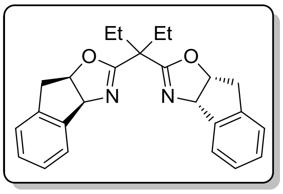

^{1}H NMR (500 MHz, $CDCl_3$) δ 7.51 (dd, *J* = 6.9, 1.8 Hz, 2H), 7.29 – 7.23 (m, 6H), 5.55 (d, *J* = 7.7 Hz, 2H), 5.24 (ddd, *J* = 8.1, 6.9, 1.5 Hz, 2H), 3.31 (dd, *J* = 17.8, 6.9 Hz, 2H), 2.97 (dd, *J* = 17.8, 1.5 Hz, 2H), 1.92 (qd, *J* = 7.5, 3.2 Hz, 4H), 0.56 (t, *J* = 7.5 Hz, 6H).

^{13}C NMR (125 MHz, $CDCl_3$) δ 168.5, 141.9, 139.7, 128.2, 127.3, 125.6, 125.0, 83.3, 76.4, 49.0, 39.6, 35.0, 24.7.

HRMS: (ESI$^+$) $[M+H]^+$ calcd for $C_{25}H_{27}N_2O_2$: 387.2067, found: 387.2073.

Specific rotation: $[\alpha]_D^{25}$ = –246.1° (c = 1.0, CH_2Cl_2).

(3a*S*,3a'*S*,8a*R*,8a'*R*)-2,2'-(nonane-5,5-diyl)bis(8,8a-dihydro-3a*H*-indeno[1,2-*d*]oxazole) (L3) (white solid)

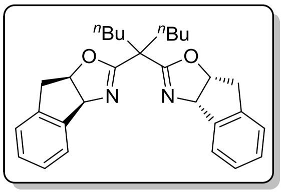

^{1}H NMR (500 MHz, $CDCl_3$) δ 7.55 – 7.50 (m, 2H), 7.31 – 7.22 (m, 6H), 5.56 (d, *J* = 7.5 Hz, 2H), 5.24 (ddd, *J* = 7.9, 6.8, 1.5 Hz, 2H), 3.30 (dd, *J* = 17.8, 6.7 Hz, 2H), 2.97 (dd, *J* = 17.7, 1.4 Hz, 2H), 1.87 (qdd, *J* = 13.8, 11.6, 5.0 Hz, 4H), 1.24 – 1.04 (m, 4H), 1.02 – 0.89 (m, 2H), 0.76 – 0.66 (m, 2H), 0.68 (t, *J* = 7.3 Hz, 6H).

^{13}C NMR (125 MHz, $CDCl_3$) δ 168.5, 141.9, 139.7, 128.2, 127.3, 125.6, 125.0, 83.3, 76.4, 49.0, 39.6, 35.0, 24.7.

HRMS: (ESI$^+$) $[M+H]^+$ calcd for $C_{29}H_{35}N_2O_2$: 443.2693, found: 443.2690.

Specific rotation: $[\alpha]_D^{25}$ = –205.2° (c = 1.0, CH_2Cl_2).

(3a*S*,3a'*S*,8a*R*,8a'*R*)-2,2'-(1,3-bis(3,5-bis(trifluoromethyl)phenyl)propane-2,2-diyl)bis(8,8a-dihydro-3a*H*-indeno[1,2-*d*]oxazole) (L10) (pale yellow solid)

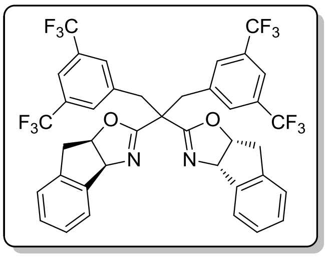

^{1}H NMR (500 MHz, $CDCl_3$) δ 7.73 (s, 2H), 7.65 (d, *J* = 1.6 Hz, 4H), 7.45 (dd, *J* = 6.8, 1.8 Hz, 2H), 7.35 – 7.22 (m, 6H), 5.48 (d, *J* = 7.9 Hz, 2H), 5.16 – 5.08 (m, 2H), 3.57 (d, *J* = 14.0 Hz, 2H), 3.29 (dd, *J* = 18.0, 7.1 Hz, 2H), 3.14 (d, *J* = 14.0 Hz, 2H), 2.84 (dd, *J* = 18.0, 1.6 Hz, 2H).

^{13}C NMR (125 MHz, $CDCl_3$) δ 165.2, 141.0, 139.2, 138.7, 131.3 (q, *J* = 32.5 Hz), 130.3 (q, *J* = 3.8 Hz), 128.6, 127.6, 125.5, 125.0, 123.2 (q, *J* = 271.3 Hz), 121.0 (hept, *J* = 3.8 Hz), 83.3, 76.3, 47.9, 40.3, 39.4.

HRMS: (ESI$^+$) $[M+H]^+$ calcd for $C_{39}H_{27}F_{12}N_2O_2$: 783.1875, found: 783.1873.

Specific rotation: $[\alpha]_D^{25}$ = –175.4° (c = 2.0, CH_2Cl_2).

(3a*S*,3a'*S*,8a*R*,8a'*R*)-2,2'-(1,3-bis(4-methoxyphenyl)propane-2,2-diyl)bis(8,8a-dihydro-3a*H*-indeno[1,2-*d*]oxazole) (L13) (white solid)

^{1}H NMR (500 MHz, $CDCl_3$) δ 7.48 (d, *J* = 7.3 Hz, 2H), 7.40 – 7.30 (m, 6H), 6.67 (d, *J* = 8.6 Hz, 4H), 6.34 (d, *J* = 8.7 Hz, 4H), 5.63 (d, *J* = 7.9 Hz, 2H), 5.39 – 5.28 (m, 2H), 3.70 (s, 6H), 3.38 (dd, *J* = 18.0, 6.7 Hz, 2H), 3.19 (d, *J* = 14.3 Hz, 2H), 3.12 (d, *J* = 18.0 Hz, 2H), 2.99 (d, *J* =

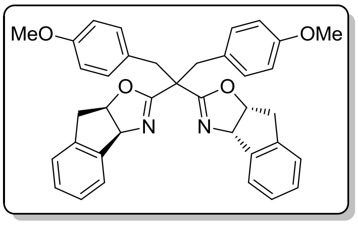

14.3 Hz, 2H).

^{13}C NMR (125 MHz, $CDCl_3$) δ 167.5, 157.9, 141.6, 139.8, 131.2, 128.3, 128.1, 127.4, 125.8, 125.1, 113.0, 83.4, 76.5, 55.0, 47.8, 39.3, 37.9.

HRMS: (ESI$^+$) $[M+H]^+$ calcd for $C_{37}H_{35}N_2O_4$: 571.2591, found: 571.2597.

Specific rotation: $[\alpha]_D^{25}$ = –55.8° (c = 0.5, CH_2Cl_2).

(3a*S*,3a'*S*,8a*R*,8a'*R*)-2,2'-(1,3-bis(4-(trifluoromethyl)phenyl)propane-2,2-diyl)bis(8,8a-dihydro-3a*H*-indeno[1,2-*d*]oxazole) (L14) (pale yellow solid)

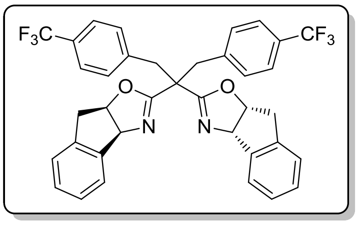

^{1}H NMR (500 MHz, $CDCl_3$) δ 7.47 – 7.42 (m, 2H), 7.42 – 7.38 (m, 2H), 7.37 – 7.30 (m, 4H), 7.00 (d, *J* = 8.0 Hz, 4H), 6.84 (d, *J* = 8.0 Hz, 4H), 5.64 (d, *J* = 7.8 Hz, 2H), 5.35 (ddd, *J* = 7.8, 6.6, 1.1 Hz, 2H), 3.38 (dd, *J* = 20.0, 10.0 Hz, 2H), 3.35 (d, *J* = 10.0 Hz, 2H), 3.05 (d, *J* = 10.0 Hz, 2H), 3.04 (d, *J* = 20.0 Hz, 2H).

^{13}C NMR (125 MHz, $CDCl_3$) δ 166.7, 141.4, 139.9, 139.6, 130.4, 128.7, 128.6 (q, *J* = 32.5 Hz), 127.7, 125.7, 125.2, 124.5 (q, *J* = 3.8 Hz), 124.2 (q, *J* = 271.3 Hz), 83.9, 76.4, 47.2, 39.7, 39.2.

HRMS: (ESI$^+$) $[M+H]^+$ calcd for $C_{37}H_{29}F_6N_2O_2$: 647.2128, found: 647.2130.

Specific rotation: $[\alpha]_D^{25}$ = –114.9° (c = 1.0, CH_2Cl_2).

(3a*S*,3a'*S*,8a*R*,8a'*R*)-2,2'-(1,3-di([1,1'-biphenyl]-4-yl)propane-2,2-diyl)bis(8,8a-dihydro-3a*H*-indeno[1,2-*d*]oxazole) (L15) (white solid)

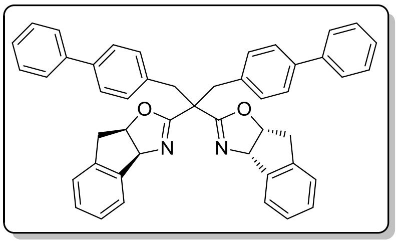

^{1}H NMR (500 MHz, $CDCl_3$) δ 7.54 – 7.48 (m, 6H), 7.44 (dd, *J* = 8.5, 6.9 Hz, 4H), 7.38 – 7.29 (m, 8H), 7.07 (d, *J* = 8.2 Hz, 4H), 6.87 (d, *J* = 8.2 Hz, 4H), 5.67 (d, *J* = 7.8 Hz, 2H), 5.37 (ddd, *J* = 8.0, 6.8, 1.3 Hz, 2H), 3.39 (dd, *J* = 20.0, 10.0 Hz, 2H), 3.35 (d, *J* = 15.0 Hz, 2H), 3.15 (d, *J* = 15.0 Hz, 2H), 3.13 (d, *J* = 15.0 Hz, 2H).

^{13}C NMR (125 MHz, $CDCl_3$) δ 167.3, 141.6, 141.0, 139.8, 139.0, 135.2, 130.6, 128.6, 128.4, 127.5, 127.0, 126.9, 126.3, 125.8, 125.1, 83.5, 76.5, 47.6, 39.4, 38.5.

HRMS: (ESI$^+$) $[M+Na]^+$ calcd for $C_{47}H_{38}N_2O_2Na$: 685.2825, found: 685.2818.

Specific rotation: $[\alpha]_D^{25}$ = –113.4° (c = 0.5, CH_2Cl_2).

2.3 Synthesis of L4

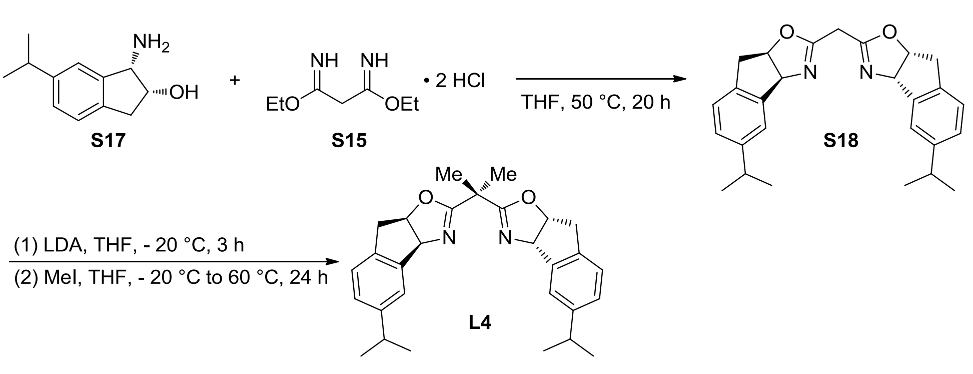

Compounds **S17**, **S18** were synthesized according to the literature reported procedure.[6]
To a solution of **S18** (414 mg, 1.0 mmol) in anhydrous THF was added LDA (2.0 M in THF, 1.0 mL, 2.0 equiv) dropwise at -20 °C. The solution was stirred at -20 °C for additional 3 hours, and then CH_3I (710 mg, 5.0 equiv) was added. The solution was warmed to rt and heated at 60 °C for 24 hours. After completion, the reaction was quenched with saturated NH_4Cl and extracted with CH_2Cl_2. The combined organic layers were dried with Na_2SO_4, filtered, and concentrated under reduced pressure. The residue was purified by silica gel column chromatography (petroleum ether/acetone) to afford the pure product.

(3a*S*,3a'*S*,8a*R*,8a'*R*)-2,2'-(cyclopentane-1,1-diyl)bis(8,8a-dihydro-3a*H*-indeno[1,2-*d*]oxazole) (L4) (pale yellow solid)

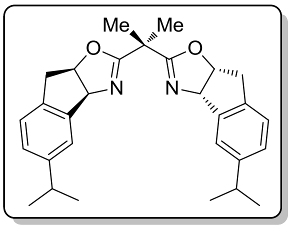

¹H NMR (500 MHz, $CDCl_3$) δ 7.41 (s, 2H), 7.16 (d, *J* = 1.4 Hz, 4H), 5.52 (d, *J* = 8.0 Hz, 2H), 5.31 (td, *J* = 7.6, 2.1 Hz, 2H), 3.32 (dd, *J* = 17.7, 7.2 Hz, 2H), 3.01 (dd, *J* = 17.7, 2.1 Hz, 2H), 2.94 (hept, *J* = 7.0 Hz, 2H), 1.45 (s, 6H), 1.27 (d, *J* = 7.0 Hz, 12H).

¹³C NMR (125 MHz, $CDCl_3$) δ 169.1, 148.3, 141.9, 137.1, 126.8, 124.8, 123.5, 83.5, 76.5, 39.4, 38.6, 34.0, 24.2, 24.1, 24.0.

HRMS: (ESI^+) $[M+H]^+$ calcd for $C_{29}H_{35}N_2O_2$: 443.2693, found: 443.2697.

2.4 Synthesis of L6, L7

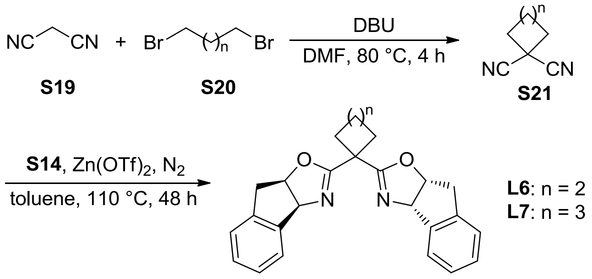

Compound **S21** was synthesized according to the literature reported procedure.[7]
A solution of **S21** and $Zn(OTf)_2$ (1.0 equiv) in anhydrous toluene was stirred at rt for 10 minutes, and then **S14** (2.2 equiv) was added. The mixture was heated at 110 °C for 48 hours under nitrogen atmosphere. After completion, ethyl acetate was added, and the organic layer was washed by saturated $NaHCO_3$ and brine. The collected organic layer was dried with Na_2SO_4, filtered, and concentrated under reduced pressure. The residue was purified by silica gel column chromatography (petroleum ether/acetone) to afford the corresponding pure products.

(3a*S*,3a'*S*,8a*R*,8a'*R*)-2,2'-(cyclopentane-1,1-diyl)bis(8,8a-dihydro-3a*H*-indeno[1,2-*d*]oxazole) (L6) (white solid)

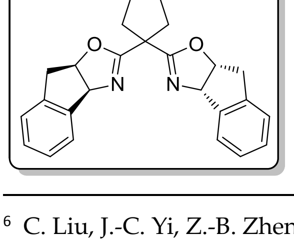

¹H NMR (500 MHz, $CDCl_3$) δ 7.54 – 7.48 (m, 2H), 7.31 – 7.22 (m, 6H), 5.53 (d, *J* = 7.8 Hz, 2H), 5.27 (td, *J* = 7.4, 1.7 Hz, 2H), 3.31 (dd, *J* = 17.8, 7.0 Hz, 2H), 2.97 (dd, *J* = 17.8, 1.7 Hz, 2H), 2.28 – 2.16 (m, 2H), 2.16 – 2.07 (m, 2H), 1.68 – 1.48 (m, 4H).

---

6 C. Liu, J.-C. Yi, Z.-B. Zheng, Y. Tang, L.-X. Dai, S.-L. You, *Angew. Chem. Int. Ed.* **2016**, *55*, 751

7 T.-Y. Tsai, K.-S. Shia, H.-J. Liu, *Synlett* **2003**, 97

^{13}C NMR (125 MHz, $CDCl_3$) δ 168.5, 141.9, 139.7, 128.2, 127.3, 125.6, 125.0, 83.3, 76.4, 49.0, 39.6, 35.0, 24.7.

HRMS: (ESI^+) $[M+H]^+$ calcd for $C_{25}H_{25}N_2O_2$: 385.1911, found: 385.1920.

Specific rotation: $[\alpha]_D^{25}$ = –335.9° (c = 1.0, CH_2Cl_2).

(3a*S*,3a'*S*,8a*R*,8a'*R*)-2,2'-(cyclohexane-1,1-diyl)bis(8,8a-dihydro-3a*H*-indeno[1,2-*d*]oxazole) (L7) (white solid)

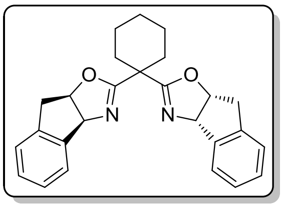

^{1}H NMR (500 MHz, $CDCl_3$) δ 7.55 – 7.48 (m, 2H), 7.32 – 7.25 (m, 4H), 7.23 (dd, *J* = 6.2, 2.4 Hz, 2H), 5.55 (d, *J* = 7.8 Hz, 2H), 5.27 – 5.22 (m, 2H), 3.31 (dd, *J* = 17.8, 6.9 Hz, 2H), 2.95 (dd, *J* = 17.8, 1.6 Hz, 2H), 1.99 (td, *J* = 8.1, 3.6 Hz, 2H), 1.91 (ddd, *J* = 13.6, 8.4, 2.9 Hz, 2H), 1.55 – 1.40 (m, 2H), 1.35 – 1.23 (m, 4H).

^{13}C NMR (125 MHz, $CDCl_3$) δ 168.5, 141.9, 139.7, 128.2, 127.3, 125.6, 125.0, 83.3, 76.4, 49.0, 39.6, 35.0, 24.7.

HRMS: (ESI^+) $[M+H]^+$ calcd for $C_{26}H_{27}N_2O_2$: 399.2067, found: 399.2063.

Specific rotation: $[\alpha]_D^{25}$ = –272.8° (c = 0.5, CH_2Cl_2).

III. Screening of Reaction Conditions

Table S1. Preliminary Screening of Ligands

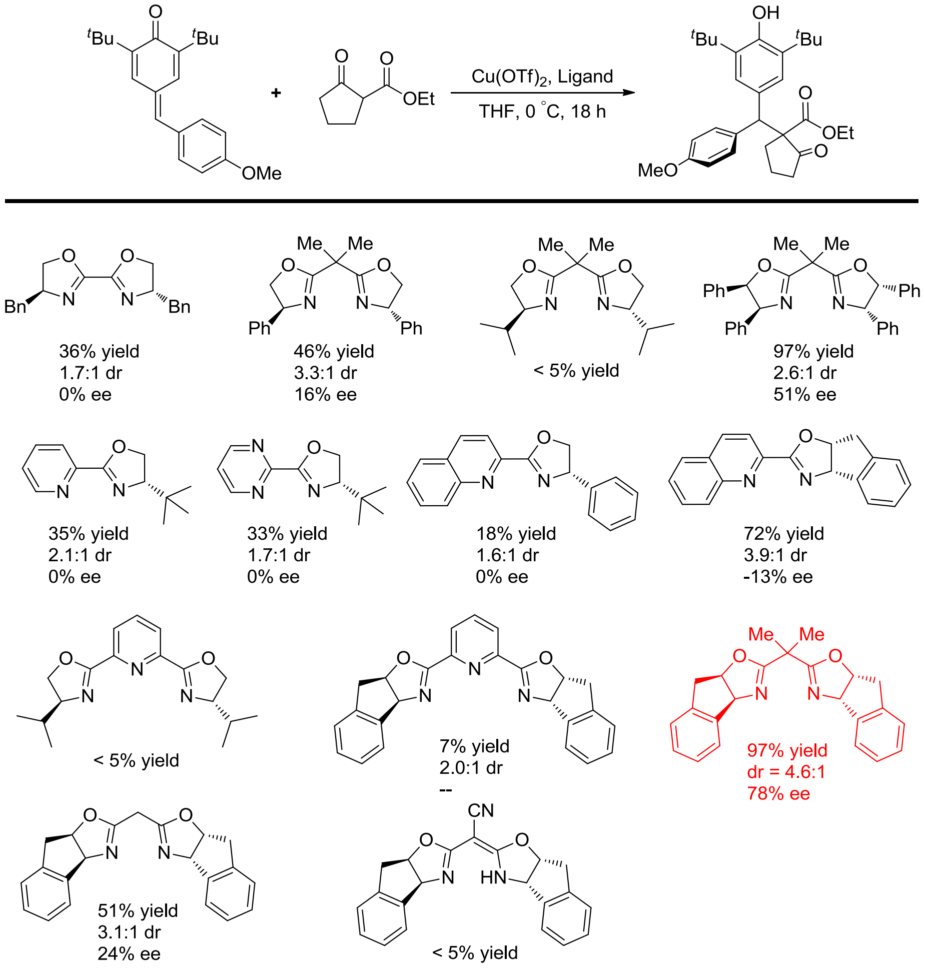

Table S2. Optimization of Reaction Conditions

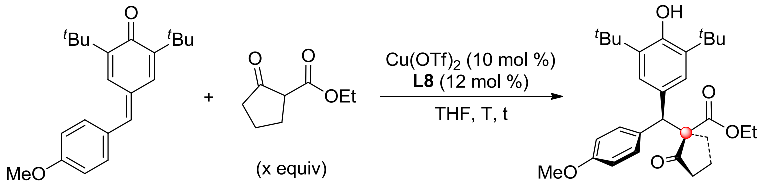

entry	T (°C)	(x equiv)	t (h)	yield (%)	dr	ee (%)
1	-10	1.5	18	98	11.5:1	94
2	-15	1.5	18	98	14.6:1	98
3	-20	1.5	18	67	14.5:1	96
4	-20	1.5	24	73	14.5:1	97
5	-30	1.5	18	33	15.6:1	97
6	-30	1.5	24	46	15.4:1	96
7	-30	1.5	36	51	15.6:1	96
8	-15	1.2	18	73	13.9:1	94
9	-15	1.2	24	82	13.8:1	95
10	-15	1.5	12	89	14.3:1	97
11	-15	1.8	12	93	14.2:1	97
12	-15	1.8	18	98	14.7:1	98
13[a]	-15	1.5	24	0	--	--

a: Without $Cu(OTf)_2$

IV. Diarylmethylation of 1,3-Dicarbonyl Compounds

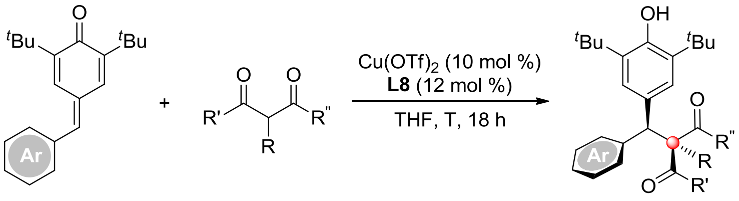

An oven-dried Schlenk tube was charged with $Cu(OTf)_2$ (1.8 mg, 0.005 mmol, 10 mol%), **L8** (4.4 mg, 0.006 mmol, 12 mol%), *p*-quinone methide substrates (0.05 mmol) and a stir bar. The tube was degased and refilled with nitrogen for 3 times, and then anhydrous THF (0.5 mL) was injected to the tube via a syringe. The reaction mixture was cooled to the temperature indicated. After stirring for 3 minutes, a solution of 1,3-dicarbonyl compounds (0.075 mol, 1.5 equiv) in anhydrous THF (0.5 mL) was injected to the reaction mixture via a syringe. After stirring at the same temperature for additional 18 hours, the reaction was quenched with saturated NH_4Cl and extracted with ethyl acetate (3 × 20 mL). The combined organic layers were dried with Na_2SO_4, filtered, and concentrated under reduced pressure. The residue was purified by silica gel column chromatography (petroleum ether/ethyl acetate) to afford the pure product.

(*R*)-ethyl 1-((*S*)-(3,5-di-*tert*-butyl-4-hydroxyphenyl)(4-methoxyphenyl)methyl)-2-oxo-cyclopentanecarboxylate (3a) (colorless oil)

^{1}H NMR (500 MHz, $CDCl_3$) δ 7.22 (d, *J* = 8.6 Hz, 2H), 6.90 (s, 2H), 6.82 (d, *J* = 8.7 Hz, 2H),

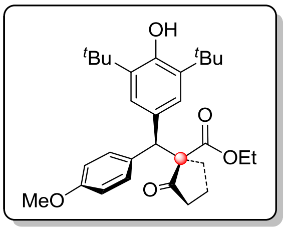

5.12 (s, 1H), 5.09 (s, 1H), 3.98 (dq, *J* = 10.7, 7.1 Hz, 1H), 3.89 (dq, *J* = 10.7, 7.1 Hz, 1H), 3.80 (s, 3H), 2.99 (dddd, *J* = 12.7, 7.2, 4.0, 1.4 Hz, 1H), 2.32 – 2.23 (m, 1H), 2.20 (ddd, *J* = 13.1, 9.3, 7.4 Hz, 1H), 1.82 (dddd, *J* = 15.3, 12.1, 9.4, 7.6 Hz, 1H), 1.69 (dt, *J* = 18.5, 9.1 Hz, 1H), 1.46 – 1.39 (m, 1H), 1.36 (s, 18H), 0.91 (t, *J* = 7.1 Hz, 3H).

^{13}C NMR (125 MHz, $CDCl_3$) δ 214.3, 169.0, 158.0, 152.4, 135.4, 133.6, 131.0, 130.0, 126.5, 113.5, 66.6, 61.5, 55.2, 54.5, 38.7, 34.3, 30.3, 29.4, 19.5, 13.6.

HRMS: (ESI$^+$) $[M+Na]^+$ calcd for $C_{30}H_{40}O_5Na$: 503.2768, found: 503.2770.

Specific rotation: $[\alpha]_D^{25}$ = –96.2° (c = 1.0, CH_2Cl_2).

HPLC analysis of the product: Daicel Chiralcel OD-H column, hexane/*i*-PrOH = 99.0:1.0, 0.7 mL/min, λ = 210 nm, retention time: 7.58 min (minor), 10.29 min (major).

(2*R*,3*S*)-ethyl 2-benzoyl-3-(3,5-di-*tert*-butyl-4-hydroxyphenyl)-3-(4-methoxyphenyl) propanoate (3b) (dark yellow solid)

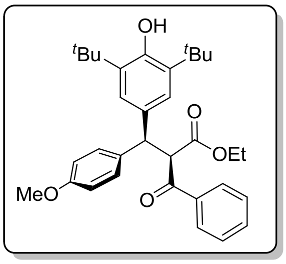

^{1}H NMR (500 MHz, $CDCl_3$) δ 7.88 (dd, *J* = 8.1, 1.3 Hz, 2H), 7.55 – 7.49 (m, 1H), 7.40 (t, *J* = 7.7 Hz, 2H), 7.32 (d, *J* = 8.7 Hz, 2H), 6.92 (s, 2H), 6.86 (d, *J* = 8.7 Hz, 2H), 5.34 (d, *J* = 11.9 Hz, 1H), 4.90 (d, *J* = 11.9 Hz, 1H), 4.91 (s, 1H), 4.00 (qd, *J* = 7.1, 1.8 Hz, 2H), 3.79 (s, 3H), 1.24 (s, 18H), 1.04 (t, *J* = 7.1 Hz, 3H).

^{13}C NMR (125 MHz, $CDCl_3$) δ 194.5, 167.9, 158.2, 152.1, 137.5, 135.5, 134.6, 133.1, 131.8, 129.0, 128.43, 128.35, 124.4, 113.8, 61.4, 59.7, 55.2, 50.9, 34.1, 30.0, 13.8.

HRMS: (ESI$^+$) $[M+Na]^+$ calcd for $C_{33}H_{40}O_5Na$: 539.2768, found: 539.2768.

Specific rotation: $[\alpha]_D^{25}$ = +3.6° (c = 1.0, CH_2Cl_2).

HPLC analysis of the product: Daicel Chiralcel OD-H column, hexane/*i*-PrOH = 98.5:1.5, 0.7 mL/min, λ = 210 nm, retention time: 14.80 min (minor), 16.91 min (major).

(2*R*,3*S*)-ethyl 3-(3,5-di-*tert*-butyl-4-hydroxyphenyl)-2-(4-fluorobenzoyl)-3-(4-methoxy-phenyl)propanoate (3c) (dark yellow solid)

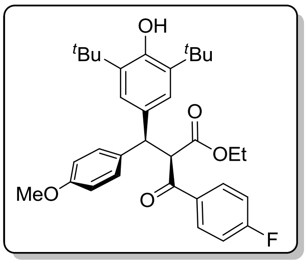

^{1}H NMR (500 MHz, $CDCl_3$) δ 7.92 (dd, *J* = 8.7, 5.5 Hz, 2H), 7.35 – 7.27 (m, 2H), 7.08 (t, *J* = 8.6 Hz, 2H), 6.91 (s, 2H), 6.89 – 6.82 (m, 2H), 5.27 (d, *J* = 11.8 Hz, 1H), 4.93 (s, 1H), 4.89 (d, *J* = 11.8 Hz, 1H), 4.00 (qd, *J* = 7.1, 1.9 Hz, 2H), 3.79 (s, 3H), 1.26 (s, 18H), 1.04 (t, *J* = 7.1 Hz, 3H).

^{13}C NMR (125 MHz, $CDCl_3$) δ 192.9, 167.8, 165.7 (d, *J* = 253.8 Hz), 158.3, 152.2, 135.7, 134.5, 134.0 (d, *J* = 2.5 Hz), 131.9, 131.1 (d, *J* = 10.0 Hz), 129.0, 124.4, 115.6 (d, *J* = 22.5 Hz), 113.9, 61.5, 59.9, 55.2, 50.9, 34.2, 30.1, 13.8.

^{19}F NMR (471 MHz, $CDCl_3$) δ -104.82.

HRMS: (ESI$^+$) $[M+Na]^+$ calcd for $C_{33}H_{39}FO_5Na$: 557.2674, found: 557.2678.

Specific rotation: $[\alpha]_D^{25}$ = +2.2° (c = 1.0, CH_2Cl_2).

HPLC analysis of the product: Daicel Chiralpak IC-3 column, hexane/*i*-PrOH = 98.5:1.5, 0.8 mL/min, λ = 254 nm, retention time: 19.23 min (major).

(2*R*,3*S*)-ethyl 2-(4-chlorobenzoyl)-3-(3,5-di-*tert*-butyl-4-hydroxyphenyl)-3-(4-methoxy-phenyl)propanoate (3d) (yellow solid)

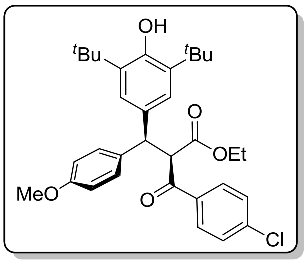

^{1}H NMR (500 MHz, $CDCl_3$) δ 7.81 (d, *J* = 8.6 Hz, 2H), 7.37 (d, *J* = 8.6 Hz, 2H), 7.31 (d, *J* = 8.7 Hz, 2H), 6.91 (s, 2H), 6.86 (d, *J* = 8.7 Hz, 2H), 5.26 (d, *J* = 11.8 Hz, 1H), 4.94 (s, 1H), 4.87 (d, *J* = 11.8 Hz, 1H), 4.01 (qd, *J* = 7.1, 1.7 Hz, 2H), 3.79 (s, 3H), 1.26 (s, 18H), 1.04 (t, *J* = 7.1 Hz, 3H).

^{13}C NMR (125 MHz, $CDCl_3$) δ 193.4, 167.7, 158.2, 152.2, 139.6, 135.8, 135.6, 134.4, 131.7, 129.7, 128.9, 128.7, 124.4, 113.9, 61.5, 59.9, 55.2, 50.9, 34.1, 30.1, 13.8.

HRMS: (ESI$^+$) $[M+Na]^+$ calcd for $C_{33}H_{39}{}^{35}ClO_5Na$: 573.2378, found: 573.2381; $[M+Na]^+$ calcd for $C_{33}H_{39}{}^{37}ClO_5Na$: 575.2349, found: 575.2368.

Specific rotation: $[\alpha]_D^{25}$ = -8.0° (c = 1.0, CH_2Cl_2).

HPLC analysis of the product: Daicel Chiralpak AD-H column, hexane/*i*-PrOH = 90.0:10.0, 0.5 mL/min, λ = 210 nm, retention time: 12.68 min (minor), 13.83 min (major).

(2*R*,3*S*)-ethyl 2-(4-bromobenzoyl)-3-(3,5-di-*tert*-butyl-4-hydroxyphenyl)-3-(4-methoxyphenyl)propanoate (3e) (yellow solid)

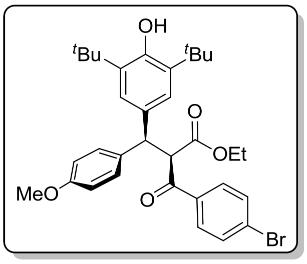

^{1}H NMR (500 MHz, $CDCl_3$) δ 7.72 (d, *J* = 8.6 Hz, 2H), 7.54 (d, *J* = 8.6 Hz, 2H), 7.30 (d, *J* = 8.7 Hz, 2H), 6.90 (s, 2H), 6.86 (d, *J* = 8.7 Hz, 2H), 5.24 (d, *J* = 11.8 Hz, 1H), 4.94 (s, 1H), 4.87 (d, *J* = 11.8 Hz, 1H), 4.00 (qd, *J* = 7.1, 1.6 Hz, 2H), 3.79 (s, 3H), 1.26 (s, 18H), 1.04 (t, *J* = 7.1 Hz, 3H).

^{13}C NMR (125 MHz, $CDCl_3$) δ 193.7, 167.7, 158.3, 152.2, 136.2, 135.6, 134.4, 131.7, 131.7, 129.8, 128.9, 128.4, 124.4, 113.9, 61.5, 59.9, 55.2, 50.9, 34.1, 30.1, 13.9.

HRMS: (ESI$^+$) $[M+Na]^+$ calcd for $C_{33}H_{39}{}^{79}BrO_5Na$: 617.1873, found: 617.1874; $[M+Na]^+$ calcd for $C_{33}H_{39}{}^{81}BrO_5Na$: 619.1853, found: 619.1861.

Specific rotation: $[\alpha]_D^{25}$ = -4.0° (c = 1.0, CH_2Cl_2).

HPLC analysis of the product: Daicel Chiralpak IC-H column, hexane/*i*-PrOH = 98.5:1.5, 0.8 mL/min, λ = 210 nm, retention time: 26.03 min (major).

(2*R*,3*S*)-ethyl 3-(3,5-di-*tert*-butyl-4-hydroxyphenyl)-3-(4-methoxyphenyl)-2-(4-(trifluoromethyl)benzoyl)propanoate (3f) (red solid)

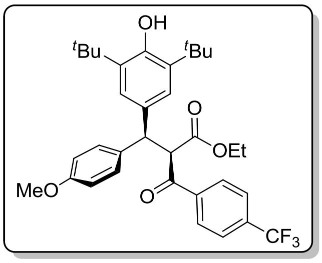

^{1}H NMR (500 MHz, $CDCl_3$) δ 7.93 (d, *J* = 8.2 Hz, 2H), 7.65 (d, *J* = 8.2 Hz, 2H), 7.32 (d, *J* = 8.6 Hz, 2H), 6.89 (s, 2H), 6.87 (d, *J* = 8.6 Hz, 2H), 5.30 (d, *J* = 11.8 Hz, 1H), 4.94 (s, 1H), 4.87 (d, *J* = 11.8 Hz, 1H), 4.02 (qd, *J* = 7.1, 1.1 Hz, 2H), 3.80 (s, 3H), 1.24 (s, 18H), 1.06 (t, *J* = 7.1 Hz, 3H).

^{13}C NMR (125 MHz, $CDCl_3$) δ 194.1, 167.5, 158.4, 152.3, 140.4, 135.8, 134.28 (q, *J* = 32.5 Hz), 134.26, 131.5, 128.8, 128.5, 125.4 (q, *J* = 3.8 Hz), 124.5, 123.5 (q, *J* = 271.3 Hz), 114.0, 61.6, 60.3, 55.2, 51.2, 34.1, 30.1, 13.9.

^{19}F NMR (471 MHz, $CDCl_3$) δ -63.23.

HRMS: (ESI$^+$) $[M+Na]^+$ calcd for $C_{34}H_{39}F_3O_5Na$: 607.2642, found: 607.2644.

Specific rotation: $[\alpha]_D^{25}$ = -15.9° (c = 1.0, CH_2Cl_2).

HPLC analysis of the product: Daicel Chiralpak AD-H column, hexane/*i*-PrOH = 92.0:8.0, 0.5 mL/min, λ = 210 nm, retention time: 9.90 min (minor), 10.57 min (major).

(2*R*,3*S*)-ethyl 3-(3,5-di-*tert*-butyl-4-hydroxyphenyl)-3-(4-methoxyphenyl)-2-(4-nitro-

benzoyl)propanoate (3g) (yellow solid)

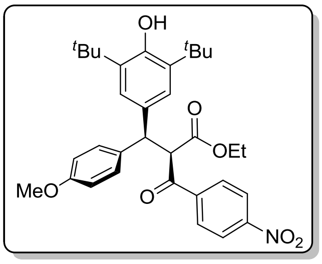

^{1}H NMR (500 MHz, $CDCl_3$) δ 8.23 (d, *J* = 8.8 Hz, 2H), 7.96 (d, *J* = 8.8 Hz, 2H), 7.31 (d, *J* = 8.7 Hz, 2H), 6.90 (s, 2H), 6.87 (d, *J* = 8.7 Hz, 2H), 5.28 (d, *J* = 11.8 Hz, 1H), 4.96 (s, 1H), 4.87 (d, *J* = 11.8 Hz, 1H), 4.03 (q, *J* = 7.1 Hz, 2H), 3.80 (s, 3H), 1.25 (s, 18H), 1.06 (t, *J* = 7.1 Hz, 3H).

^{13}C NMR (125 MHz, $CDCl_3$) δ 193.8, 167.3, 158.4, 152.4, 150.0, 142.0, 135.8, 133.9, 131.4, 129.2, 128.8, 124.4, 123.6, 114.0, 61.8, 60.6, 55.2, 51.1, 34.1, 30.1, 13.9.

HRMS: (ESI^+) $[M+Na]^+$ calcd for $C_{33}H_{39}NO_7Na$: 584.2619, found: 584.2619.

Specific rotation: $[\alpha]_D^{25}$ = -5.7° (c = 1.0, CH_2Cl_2)

HPLC analysis of the product: Daicel Chiralpak IA-3 column, hexane/*i*-PrOH = 90.0:10.0, 0.5 mL/min, *λ* = 254 nm, retention time: 16.50 min (major), 17.59 min (minor).

(2*R*,3*S*)-ethyl 3-(3,5-di-*tert*-butyl-4-hydroxyphenyl)-3-(4-methoxyphenyl)-2-(4-methylbenzoyl)propanoate (3h) (colorless oil)

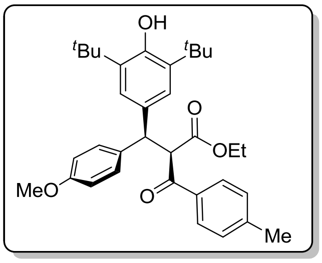

^{1}H NMR (500 MHz, $CDCl_3$) δ 7.80 (d, *J* = 8.1 Hz, 2H), 7.32 (d, *J* = 8.6 Hz, 2H), 7.20 (d, *J* = 8.1 Hz, 2H), 6.92 (s, 2H), 6.85 (d, *J* = 8.6 Hz, 2H), 5.31 (d, *J* = 11.8 Hz, 1H), 4.91 (s, 1H), 4.89 (d, *J* = 11.8 Hz, 1H), 3.98 (qd, *J* = 7.1, 2.8 Hz, 2H), 3.79 (s, 3H), 2.39 (s, 3H), 1.25 (s, 18H), 1.03 (t, *J* = 7.1 Hz, 3H).

^{13}C NMR (125 MHz, $CDCl_3$) δ 193.9, 168.1, 158.1, 152.1, 144.0, 135.4, 135.0, 134.7, 132.0, 129.1, 129.0, 128.6, 124.4, 113.8, 61.3, 59.6, 55.2, 50.8, 34.1, 30.1, 21.6, 13.8.

HRMS: (ESI^+) $[M+Na]^+$ calcd for $C_{34}H_{42}O_5Na$: 553.2924, found: 553.2925.

Specific rotation: $[\alpha]_D^{25}$ = -3.7° (c = 1.0, CH_2Cl_2)

HPLC analysis of the product: Daicel Chiralpak IA-3 column, hexane/*i*-PrOH = 90.0:10.0, 0.6 mL/min, *λ* = 254 nm, retention time: 10.04 min (minor), 12.25 min (major).

(2*R*,3*S*)-ethyl 3-(3,5-di-*tert*-butyl-4-hydroxyphenyl)-2-(4-methoxybenzoyl)-3-(4-methoxyphenyl)propanoate (3i) (yellow oil)

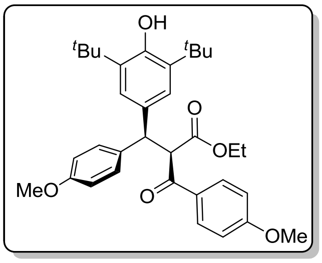

^{1}H NMR (500 MHz, $CDCl_3$) δ 7.91 (d, *J* = 9.0 Hz, 2H), 7.31 (d, *J* = 8.6 Hz, 2H), 6.93 (s, 2H), 6.88 (d, *J* = 9.0 Hz, 2H), 6.85 (d, *J* = 8.6 Hz, 2H), 5.28 (d, *J* = 11.9 Hz, 1H), 4.91 (s, 1H), 4.90 (d, *J* = 11.1 Hz, 1H), 3.98 (qd, *J* = 7.1, 3.1 Hz, 2H), 3.86 (s, 3H), 3.79 (s, 3H), 1.26 (s, 18H), 1.02 (t, *J* = 7.1 Hz, 3H).

^{13}C NMR (125 MHz, $CDCl_3$) δ 192.5, 168.2, 163.6, 158.1, 152.0, 135.4, 134.8, 132.1, 130.9, 130.8, 130.4, 129.0, 124.4, 113.8, 61.3, 59.4, 55.5, 55.2, 50.6, 34.1, 30.1, 13.9.

HRMS: (ESI^+) $[M+K]^+$ calcd for $C_{34}H_{42}O_6K$: 585.2613, found: 585.2638.

Specific rotation: $[\alpha]_D^{25}$ = +2.6° (c = 1.0, CH_2Cl_2)

HPLC analysis of the product: Daicel Chiralpak AD-H column, hexane/*i*-PrOH = 90.0:10.0, 0.5 mL/min, *λ* = 210 nm, retention time: 20.60 min (minor), 28.98 min (major).

(2*R*,3*S*)-ethyl 3-(3,5-di-*tert*-butyl-4-hydroxyphenyl)-3-(4-methoxyphenyl)-2-(3-methylbenzoyl)propanoate (3j) (colorless oil)

^{1}H NMR (500 MHz, $CDCl_3$) δ 7.71 (d, *J* = 7.6 Hz, 1H), 7.63 (s, 1H), 7.36 – 7.27 (m, 4H), 6.92

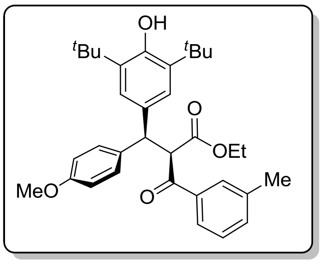

(s, 2H), 6.86 (d, *J* = 8.7 Hz, 2H), 5.33 (d, *J* = 11.8 Hz, 1H), 4.91 (s, 1H), 4.89 (d, *J* = 11.8 Hz, 1H), 4.00 (qt, *J* = 7.1, 3.7 Hz, 2H), 3.79 (s, 3H), 2.37 (s, 3H), 1.25 (s, 18H), 1.04 (t, *J* = 7.1 Hz, 3H).

^{13}C NMR (125 MHz, $CDCl_3$) δ 194.8, 168.0, 158.2, 152.1, 138.2, 137.7, 135.5, 134.7, 133.9, 131.9, 129.0, 128.8, 128.3, 125.6, 124.4, 113.8, 61.4, 59.7, 55.2, 51.0, 34.1, 30.1, 21.3, 13.9.

HRMS: (ESI$^+$) [M+Na]$^+$ calcd for $C_{34}H_{42}O_5Na$: 553.2924, found: 553.2923.

Specific rotation: $[\alpha]_D^{25}$ = -0.7° (c = 1.0, CH_2Cl_2)

HPLC analysis of the product: Daicel Chiralpak AD-H column, hexane/*i*-PrOH = 92.0:8.0, 0.5 mL/min, λ = 254 nm, retention time: 12.97 min (minor), 13.67 min (major).

(2*R*,3*S*)-ethyl 2-(3-chlorobenzoyl)-3-(3,5-di-*tert*-butyl-4-hydroxyphenyl)-3-(4-methoxyphenyl)propanoate (3k) (colorless oil)

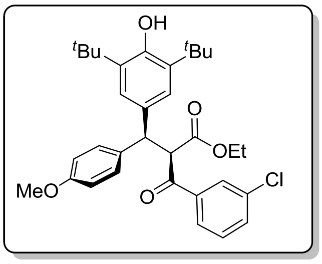

^{1}H NMR (500 MHz, $CDCl_3$) δ 7.78 – 7.72 (m, 2H), 7.49 (ddd, *J* = 8.0, 2.1, 1.0 Hz, 1H), 7.37 – 7.29 (m, 3H), 6.90 (s, 2H), 6.86 (d, *J* = 8.7 Hz, 2H), 5.25 (d, *J* = 11.8 Hz, 1H), 4.94 (s, 1H), 4.86 (d, *J* = 11.8 Hz, 1H), 4.02 (qd, *J* = 7.1, 2.8 Hz, 2H), 3.80 (s, 3H), 1.26 (s, 18H), 1.06 (t, *J* = 7.1 Hz, 3H).

^{13}C NMR (125 MHz, $CDCl_3$) δ 193.7, 167.6, 158.3, 152.3, 139.2, 135.7, 134.8, 134.3, 132.9, 131.5, 129.7, 128.9, 128.3, 126.3, 124.5, 113.9, 61.6, 59.9, 55.2, 51.1, 34.1, 30.1, 13.9.

HRMS: (ESI$^+$) [M+Na]$^+$ calcd for $C_{33}H_{39}{}^{35}ClO_5Na$: 573.2378, found: 573.2375; [M+Na]$^+$ calcd for $C_{33}H_{39}{}^{37}ClO_5Na$: 575.2349, found: 575.2366.

Specific rotation: $[\alpha]_D^{25}$ = -21.5° (c = 1.0, CH_2Cl_2)

HPLC analysis of the product: Daicel Chiralpak IC-3 column, hexane/*i*-PrOH = 98.5:1.5, 0.8 mL/min, λ = 210 nm, retention time: 15.79 min (major), 26.68 min (minor).

(2*R*,3*S*)-ethyl 2-(3-bromobenzoyl)-3-(3,5-di-*tert*-butyl-4-hydroxyphenyl)-3-(4-methoxyphenyl)propanoate (3l) (yellow oil)

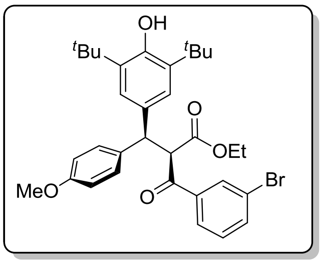

^{1}H NMR (500 MHz, $CDCl_3$) δ 7.91 (t, *J* = 1.8 Hz, 1H), 7.79 (dt, *J* = 7.7, 1.3 Hz, 1H), 7.66 – 7.63 (m, 1H), 7.37 – 7.28 (m, 3H), 6.90 (s, 2H), 6.86 (d, *J* = 8.7 Hz, 2H), 5.25 (d, *J* = 11.8 Hz, 1H), 4.95 (s, 1H), 4.86 (d, *J* = 11.8 Hz, 1H), 4.02 (tq, *J* = 7.1, 3.3 Hz, 2H), 3.80 (s, 3H), 1.27 (s, 18H), 1.06 (t, *J* = 7.1 Hz, 3H).

^{13}C NMR (125 MHz, $CDCl_3$) δ^{13}C NMR (126 MHz, Chloroform-*d*) δ 193.6, 167.6, 158.3, 152.3, 139.4, 135.8, 135.8, 134.4, 131.6, 131.2, 130.0, 128.9, 126.8, 124.5, 122.8, 113.9, 61.5, 60.0, 55.2, 51.1, 34.2, 30.1, 13.9.

HRMS: (ESI$^+$) [M+Na]$^+$ calcd for $C_{33}H_{39}{}^{79}BrO_5Na$: 617.1873, found: 617.1875; [M+Na]$^+$ calcd for $C_{33}H_{39}{}^{81}BrO_5Na$: 619.1853, found: 617.1862.

Specific rotation: $[\alpha]_D^{25}$ = -18.5° (c = 1.0, CH_2Cl_2)

HPLC analysis of the product: Daicel Chiralpak IC-3 column, hexane/*i*-PrOH = 98.5:1.5, 0.8 mL/min, λ = 254 nm, retention time: 17.99 min (major), 27.53 min (minor).

(2*R*,3*S*)-ethyl 3-(3,5-di-*tert*-butyl-4-hydroxyphenyl)-3-(4-methoxyphenyl)-2-(2-methylbenzoyl)propanoate (3m) (dark yellow oil)

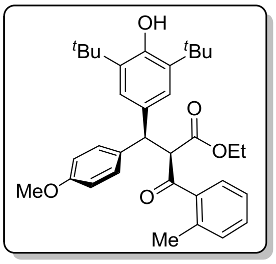

^{1}**H NMR** (500 MHz, $CDCl_3$) δ 7.89 (dd, *J* = 7.3, 1.6 Hz, 1H), 7.38 – 7.29 (m, 4H), 7.08 (d, *J* = 7.3 Hz, 1H), 6.95 (s, 2H), 6.83 (d, *J* = 8.6 Hz, 2H), 5.26 (d, *J* = 12.1 Hz, 1H), 4.98 (s, 1H), 4.80 (d, *J* = 12.1 Hz, 1H), 4.08 (tq, *J* = 7.1, 3.4 Hz, 2H), 3.77 (s, 3H), 1.80 (s, 3H), 1.24 (s, 18H), 1.12 (t, *J* = 7.1 Hz, 3H).

^{13}**C NMR** (125 MHz, $CDCl_3$) δ 197.8, 168.0, 158.2, 152.4, 139.3, 138.8, 135.6, 134.8, 131.5, 131.28, 131.25, 128.6, 128.1, 125.5, 124.5, 113.9, 62.1, 61.4, 55.2, 52.1, 34.1, 29.9, 19.6, 14.0.

HRMS: (ESI$^+$) $[M+Na]^+$ calcd for $C_{34}H_{42}O_5Na$: 553.2924, found: 553.2929.

Specific rotation: $[\alpha]_D^{25}$ = –25.3° (c = 1.0, CH_2Cl_2).

HPLC analysis of the product: Daicel Chiralcel OD-H column, hexane/*i*-PrOH = 98.0:2.0, 0.5 mL/min, λ = 210 nm, retention time: 15.68 min (minor), 17.99 min (major).

(*R*)-methyl 2-((*S*)-(3,5-di-*tert*-butyl-4-hydroxyphenyl)(4-methoxyphenyl)methyl)-3-oxobutanoate (3n) (yellow oil)

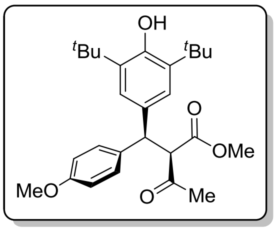

^{1}**H NMR** (500 MHz, $CDCl_3$) δ 7.23 (d, *J* = 8.7 Hz, 2H), 7.03 (s, 2H), 6.83 (d, *J* = 8.7 Hz, 2H), 5.08 (s, 1H), 4.64 (d, *J* = 12.1 Hz, 1H), 4.46 (d, *J* = 12.1 Hz, 1H), 3.78 (s, 3H), 3.55 (s, 3H), 2.04 (s, 3H), 1.41 (s, 18H).

^{13}**C NMR** (125 MHz, $CDCl_3$) δ 202.3, 168.4, 158.2, 152.5, 136.0, 134.3, 131.7, 128.5, 124.3, 113.9, 65.8, 55.1, 52.4, 50.5, 34.3, 30.3, 30.1.

HRMS: (ESI$^+$) $[M+Na]^+$ calcd for $C_{27}H_{36}O_5Na$: 463.2455, found: 463.2459.

Specific rotation: $[\alpha]_D^{25}$ = +2.5° (c = 1.0, CH_2Cl_2).

HPLC analysis of the product: Daicel Chiralcel OD-H column, hexane/*i*-PrOH = 98.5:1.5, 0.7 mL/min, λ = 280 nm, retention time: 10.91 min (major), 13.38 min (minor).

(*R*)-ethyl 2-((*S*)-(3,5-di-*tert*-butyl-4-hydroxyphenyl)(4-methoxyphenyl)methyl)-3-oxobutanoate (3o) (yellow oil)

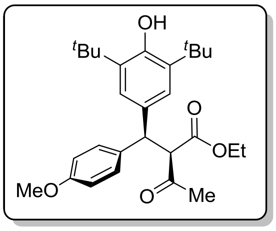

^{1}**H NMR** (500 MHz, $CDCl_3$) δ 7.24 (d, *J* = 8.7 Hz, 2H), 7.04 (s, 2H), 6.82 (d, *J* = 8.7 Hz, 2H), 5.08 (s, 1H), 4.63 (d, *J* = 12.1 Hz, 1H), 4.44 (d, *J* = 12.1 Hz, 1H), 4.00 (qd, *J* = 7.1, 1.6 Hz, 2H), 3.77 (s, 3H), 2.05 (s, 3H), 1.41 (s, 18H), 1.05 (t, *J* = 7.1 Hz, 3H).

^{13}**C NMR** (125 MHz, $CDCl_3$) δ 202.4, 167.9, 158.2, 152.5, 136.0, 134.4, 131.8, 128.6, 124.2, 113.9, 66.0, 61.3, 55.2, 50.5, 34.3, 31.6, 30.3, 13.8.

HRMS: (ESI$^+$) $[M+Na]^+$ calcd for $C_{28}H_{38}O_5Na$: 477.2611, found: 477.2613.

Specific rotation: $[\alpha]_D^{25}$ = +5.5° (c = 1.0, CH_2Cl_2)

HPLC analysis of the product: Daicel Chiralpak IA-3 column, hexane/*i*-PrOH = 96.0:4.0, 0.6 mL/min, λ = 280 nm, retention time: 9.82 min (major), 12.30 min (minor).

(*R*)-isopropyl 2-((*S*)-(3,5-di-*tert*-butyl-4-hydroxyphenyl)(4-methoxyphenyl)methyl)-3-oxobutanoate (3p) (yellow oil)

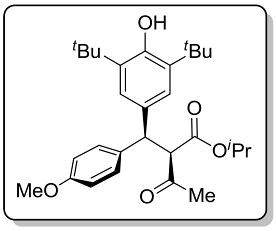

^{1}**H NMR** (500 MHz, $CDCl_3$) δ 7.24 (d, *J* = 8.7 Hz, 2H), 7.04 (s, 2H), 6.82 (d, *J* = 8.7 Hz, 2H), 5.07 (s, 1H), 4.84 (hept, *J* = 6.3 Hz, 1H), 4.62 (d, *J* = 12.2 Hz, 1H), 4.41 (d, *J* = 12.2 Hz, 1H), 3.77 (s, 3H), 1.41 (s, 18H), 1.05 (d, *J* = 6.3 Hz, 3H), 1.00 (d, *J* = 6.3 Hz, 3H).

^{13}**C NMR** (125 MHz, $CDCl_3$) δ 202.4, 167.5, 158.2, 152.5, 135.9, 134.5, 132.0, 128.7, 124.2, 113.8, 68.9, 66.2, 55.2, 50.5, 34.3, 31.6, 30.3, 21.4, 21.3.

HRMS: (ESI$^+$) [M+Na]$^+$ calcd for $C_{29}H_{40}O_5Na$: 491.2768, found: 491.2768.

Specific rotation: $[\alpha]_D^{25}$ = +6.0° (c = 1.0, CH_2Cl_2)

HPLC analysis of the product: Daicel Chiralpak IA-3 column, hexane/*i*-PrOH = 97.0:3.0, 0.45 mL/min, λ = 210 nm, retention time: 13.53 min (major), 17.21 min (minor).

(2*R*/*S*,3*S*)-ethyl 2-(cyclopropanecarbonyl)-3-(3,5-di-tert-butyl-4-hydroxyphenyl)-3-(4-methoxyphenyl)propanoate (3q) (colorless oil)

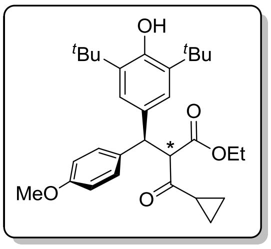

^{1}**H NMR** (500 MHz, $CDCl_3$) δ 7.22 (d, *J* = 8.7 Hz, 2H), 7.09 (s, 2H), 6.82 – 6.79 (m, 2H), 5.05 (s, 1H), 4.72 (d, *J* = 10.0 Hz, 1H), 4.52 (d, *J* = 10.0 Hz, 1H), 4.01 – 3.96 (m, 2H), 3.77 (s, 3H), 2.17 (tt, *J* = 7.7, 5.4 Hz, 1H), 1.42 (s, 18H), 0.98 (t, *J* = 7.1 Hz, 3H), 0.93 – 0.84 (m, 2H), 0.75 – 0.67 (m, 2H) (diastereoisomer **A**).

δ 7.27 (d, *J* = 8.7 Hz, 2H), 7.04 (s, 2H), 6.83 (d, *J* = 8.7 Hz, 2H), 5.05 (s, 1H), 4.70 (d, *J* = 12.1 Hz, 1H), 4.54 (d, *J* = 12.1 Hz, 1H), 4.02 (qd, *J* = 7.1, 2.8 Hz, 2H), 3.78 (s, 3H), 2.05 (tt, *J* = 7.6, 4.8 Hz, 1H), 1.40 (s, 18H), 1.07 (t, *J* = 7.1 Hz, 3H), 0.85 – 0.72 (m, 4H) (diastereoisomer **B**).

^{13}**C NMR** (125 MHz, $CDCl_3$) δ 204.1, 168.21, 158.15, 152.36, 135.7, 134.3, 132.5, 128.9, 124.2, 113.9, 66.5, 61.1, 55.16, 50.5, 34.3, 20.5, 13.8, 11.9, 11.40. (diastereoisomer **A**).

δ 204.0, 168.17, 158.20, 152.34, 135.8, 134.6, 132.1, 128.7, 124.5, 113.9, 66.3, 61.2, 55.18, 50.6, 30.3, 21.0, 13.9, 11.8, 11.36. (diastereoisomer **B**).

HRMS: (ESI$^+$) [M+Na]$^+$ calcd for $C_{30}H_{40}O_5Na$: 503.2768, found: 503.2776.

Specific rotation: $[\alpha]_D^{25}$ = -3.8° (c = 1.0, CH_2Cl_2)

HPLC analysis of the product: Daicel Chiralpak AD-H column, hexane/*i*-PrOH = 95.0:5.0, 0.5 mL/min, λ = 280 nm, retention time: 12.71 min (major), 16.56 min (minor) (diastereoisomer **A**)/15.47 min (minor), 17.16 min (major) (diastereoisomer **B**).

(2*R*,3*S*)-ethyl 2-(cyclopentanecarbonyl)-3-(3,5-di-*tert*-butyl-4-hydroxyphenyl)-3-(4-methoxyphenyl)propanoate (3r) (colorless oil)

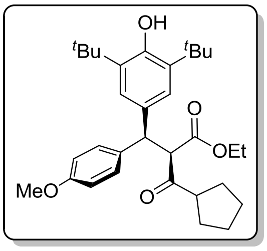

^{1}**H NMR** (500 MHz, $CDCl_3$) δ 7.28 (d, *J* = 8.7 Hz, 2H), 7.02 (s, 2H), 6.84 (d, *J* = 8.7 Hz, 2H), 4.68 (d, *J* = 12.0 Hz, 1H), 4.52 (d, *J* = 12.0 Hz, 1H), 4.07 – 3.94 (m, 2H), 3.78 (s, 3H), 2.71 (m, 1H), 1.78 – 1.17 (m, 8H), 1.39 (s, 3H), 1.03 (t, *J* = 7.1 Hz, 3H).

^{13}**C NMR** (125 MHz, $CDCl_3$) δ 206.2, 167.9, 158.2, 152.4, 135.9, 134.6, 132.2, 128.7, 124.8 113.9, 65.0, 61.2, 55.2, 52.4, 50.5, 34.3, 30.3, 28.51, 28.49, 25.8, 25.7, 13.9.

HRMS: (ESI$^+$) [M+Na]$^+$ calcd for $C_{32}H_{44}O_5Na$: 531.3081, found: 531.3086.

Specific rotation: $[\alpha]_D^{25}$ = -40.6° (c = 1.0, CH_2Cl_2)

HPLC analysis of the product: Daicel Chiralpak IA-3 column, hexane/*i*-PrOH = 97.0:3.0, 0.45 mL/min, λ = 210 nm, retention time: 16.53 min (major), 30.26 min (minor).

(2*R*,3*S*)-ethyl 2-(cyclohexanecarbonyl)-3-(3,5-di-*tert*-butyl-4-hydroxyphenyl)-3-(4-methoxyphenyl)propanoate (3s) (colorless oil)

^{1}**H NMR** (500 MHz, $CDCl_3$) δ 7.28 (d, *J* = 8.7 Hz, 2H), 7.02 (s, 2H), 6.84 (d, *J* = 8.7 Hz, 2H), 5.06 (s, 1H), 4.63 (d, *J* = 12.1 Hz, 1H), 4.56 (d, *J* = 12.1 Hz, 1H), 4.05 – 3.95 (m, 2H), 3.78 (s, 3H), 2.04 (m, 1H), 1.76 – 1.67 (m, 2H), 1.62 – 1.54 (m, 2H), 1.40 (s, 18H), 1.34 – 1.22 (m, 2H),

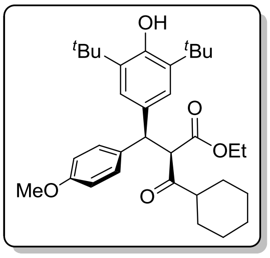

1.20 – 1.08 (m, 2H), 1.04 (t, *J* = 7.1 Hz, 3H), 1.01 – 0.83 (m, 2H).

^{13}C NMR (125 MHz, $CDCl_3$) δ 207.1, 167.9, 158.1, 152.5, 135.9, 134.5, 132.1, 128.5, 124.7, 113.9, 63.5, 61.1, 55.2, 52.3, 50.9, 34.3, 30.2, 28.0, 27.4, 25.72, 25.67, 25.2, 13.9.

HRMS: (ESI^+) $[M+Na]^+$ calcd for $C_{33}H_{46}O_5Na$: 545.3237, found: 545.3238.

Specific rotation: $[\alpha]_D^{25}$ = -59.3° (c = 1.0, CH_2Cl_2).

HPLC analysis of the product: Daicel Chiralcel OD-H column, hexane/*i*-PrOH = 98.0:2.0, 0.5 mL/min, λ = 210 nm, retention time: 11.13 min (major), 15.24 min (minor).

(2*R*,3*S*)-ethyl 3-(3,5-di-*tert*-butyl-4-hydroxyphenyl)-2-(furan-2-carbonyl)-3-(4-methoxyphenyl)propanoate (3t) (yellow oil)

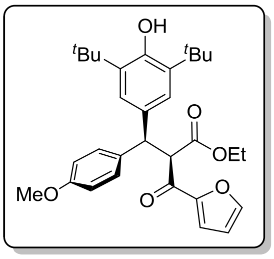

^{1}H NMR (500 MHz, $CDCl_3$) δ 7.58 (d, *J* = 1.6 Hz, 1H), 7.32 (d, *J* = 8.7 Hz, 2H), 7.21 (d, *J* = 3.3 Hz, 1H), 7.00 (s, 2H), 6.85 (d, *J* = 8.7 Hz, 2H), 6.51 (dd, *J* = 3.6, 1.7 Hz, 1H), 5.15 (d, *J* = 12.0 Hz, 1H), 4.95 (s, 1H), 4.87 (d, *J* = 12.0 Hz, 1H), 3.99 (q, *J* = 7.1 Hz, 2H), 3.79 (s, 3H), 1.31 (s, 18H), 1.03 (t, *J* = 7.1 Hz, 3H).

^{13}C NMR (125 MHz, $CDCl_3$) δ 182.3, 167.7, 158.2, 152.4, 152.2, 147.0, 135.5, 134.5, 131.8, 128.9, 124.3, 118.5, 113.8, 112.5, 61.4, 59.7, 55.2, 50.3, 34.2, 30.2, 13.8.

HRMS: (ESI^+) $[M+Na]^+$ calcd for $C_{31}H_{38}O_6Na$: 529.2561, found: 529.2569.

Specific rotation: $[\alpha]_D^{25}$ = -3.7° (c = 1.0, CH_2Cl_2)

HPLC analysis of the product: Daicel Chiralpak IC-3 column, hexane/*i*-PrOH = 95.0:5.0, 0.8 mL/min, λ = 280 nm, retention time: 25.24 min (major), 31.33 min (minor).

(2*R*,3*S*)-ethyl 3-(3,5-di-*tert*-butyl-4-hydroxyphenyl)-3-(4-methoxyphenyl)-2-(thiophene-2-carbonyl)propanoate (3u) (yellow oil)

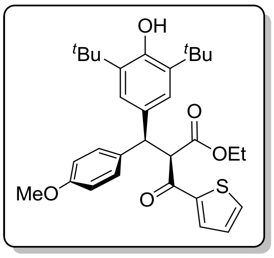

^{1}H NMR (500 MHz, $CDCl_3$) δ 7.77 (dd, *J* = 3.8, 1.1 Hz, 1H), 7.62 (dd, *J* = 4.9, 1.1 Hz, 1H), 7.31 (d, *J* = 8.6 Hz, 2H), 7.08 (dd, *J* = 4.9, 3.8 Hz, 1H), 6.97 (s, 2H), 6.86 (d, *J* = 8.6 Hz, 2H), 5.09 (d, *J* = 11.9 Hz, 1H), 4.94 (s, 1H), 4.89 (d, *J* = 11.9 Hz, 1H), 3.99 (q, *J* = 7.1 Hz, 2H), 3.80 (s, 3H), 1.28 (s, 18H), 1.03 (t, *J* = 7.1 Hz, 3H).

^{13}C NMR (125 MHz, $CDCl_3$) δ 186.4, 167.7, 158.3, 152.2, 144.4, 135.6, 134.5, 134.5, 132.8, 131.9, 129.0, 128.0, 124.4, 113.9, 61.4, 55.2, 50.6, 46.5, 34.2, 30.1, 13.8.

HRMS: (ESI^+) $[M+Na]^+$ calcd for $C_{31}H_{38}O_5SNa$: 545.2332, found: 545.2339.

Specific rotation: $[\alpha]_D^{25}$ = -30.4 (c = 1.0, CH_2Cl_2)

HPLC analysis of the product: Daicel Chiralpak IC-3 column, hexane/*i*-PrOH = 90.0:10.0, 0.8 mL/min, λ = 210 nm, retention time: 13.12 min (major), 15.58 min (minor).

(*R*)-methyl 1-((*S*)-(3,5-di-*tert*-butyl-4-hydroxyphenyl)(4-methoxyphenyl)methyl)-2-oxocyclopentanecarboxylate (3v) (pale yellow solid)

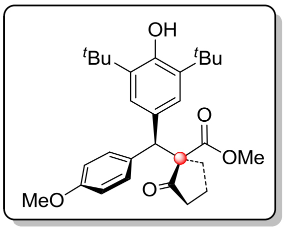

^{1}H NMR (500 MHz, $CDCl_3$) δ 7.21 (d, *J* = 8.7 Hz, 2H), 6.91 (s, 2H), 6.83 (d, *J* = 8.7 Hz, 2H), 5.12 (s, 1H), 5.10 (s, 1H), 3.80 (s, 3H), 3.47 (s, 3H), 3.00 (dddd, *J* = 12.8, 7.3, 4.0, 1.4 Hz, 1H), 2.33 – 2.17 (m, 2H), 1.89 – 1.74 (m, 1H), 1.74 – 1.66 (m, 1H), 1.48 – 1.38 (m, 1H), 1.37 (s, 18H).

^{13}C NMR (125 MHz, $CDCl_3$) δ 214.4, 169.5, 157.9, 152.4, 135.4, 133.4, 130.7, 129.9, 126.5, 113.5, 66.8, 55.1, 54.6, 52.7, 38.8, 34.3, 30.3, 29.2, 19.6.

HRMS: (ESI^+) $[M+Na]^+$ calcd for $C_{29}H_{38}O_5Na$: 489.2611, found: 489.2612.

Specific rotation: $[\alpha]_D^{25}$ = -84.6° (c = 1.0, CH_2Cl_2).

HPLC analysis of the product: Daicel Chiralcel OD-H column, hexane/*i*-PrOH = 98.5:1.5, 0.7 mL/min, λ = 210 nm, retention time: 9.40 min (major), 11.33 min (minor).

(*R*)-ethyl 1-((*S*)-(3,5-di-*tert*-butyl-4-hydroxyphenyl)(4-methoxyphenyl)methyl)-2-oxo-cyclohexanecarboxylate (3w) (colorless oil)

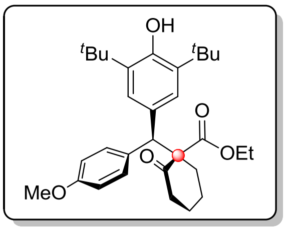

^{1}H NMR (500 MHz, $CDCl_3$) δ 7.37 (d, *J* = 8.7 Hz, 2H), 7.23 (s, 2H), 6.79 (d, *J* = 8.7 Hz, 2H), 5.05 (s, 1H), 4.87 (s, 1H), 3.98 – 3.88 (m, 1H), 3.85 – 3.73 (m, 1H), 3.77 (s, 3H), 2.61 (dd, *J* = 13.8, 3.0 Hz, 1H), 2.52 – 2.46 (m, 1H), 2.46 – 2.37 (m, 1H), 1.95 (ddd, *J* = 12.6, 6.0, 3.0 Hz, 1H), 1.80 – 1.71 (m, 2H), 1.60 – 1.51 (m, 2H), 1.42 (s, 18H), 0.95 (t, *J* = 7.1 Hz, 3H).

^{13}C NMR (125 MHz, $CDCl_3$) δ 206.7, 171.0, 158.1, 152.1, 134.9, 133.6, 131.7, 131.4, 127.3, 113.1, 66.5, 61.1, 55.2, 42.0, 34.4, 31.6, 30.4, 29.7, 26.8, 22.9, 13.6.

HRMS: (ESI^+) $[M+Na]^+$ calcd for $C_{31}H_{42}O_5Na$: 517.2924, found: 517.2927.

Specific rotation: $[\alpha]_D^{25}$ = +13.6° (c = 1.0, CH_2Cl_2)

HPLC analysis of the product: Daicel Chiralpak AD-H column, hexane/*i*-PrOH = 98.5:1.5, 0.5 mL/min, λ = 210 nm, retention time: 11.99 min (major), 15.34 min (minor).

(*S*)-methyl 1-((*S*)-(3,5-di-*tert*-butyl-4-hydroxyphenyl)(4-methoxyphenyl)methyl)-2-oxo-2,3-dihydro-1H-indene-1-carboxylate (3x) (colorless oil)

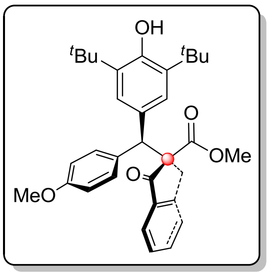

^{1}H NMR (500 MHz, $CDCl_3$) δ 7.63 (d, *J* = 7.5 Hz, 1H), 7.46 (t, *J* = 7.5 Hz, 1H), 7.30 (d, *J* = 7.5 Hz, 1H), 7.25 (t, *J* = 7.5 Hz, 1H), 7.22 (d, *J* = 8.7 Hz, 2H), 6.87 (d, *J* = 8.7 Hz, 2H), 6.75 (s, 2H), 5.39 (s, 1H), 4.88 (s, 1H), 4.18 (d, *J* = 17.1 Hz, 1H), 3.83 (s, 3H), 3.57 (s, 3H), 3.52 (d, *J* = 17.1 Hz, 1H), 1.20 (s, 18H).

^{13}C NMR (125 MHz, $CDCl_3$) δ 201.7, 170.2, 158.0, 153.9, 152.2, 135.2, 135.0, 133.8, 130.2, 129.7, 127.2, 126.1, 125.8, 124.3, 113.7, 66.5, 55.2, 54.1, 52.9, 34.1, 33.6, 30.1.

HRMS: (ESI^+) $[M+Na]^+$ calcd for $C_{33}H_{38}O_5Na$: 537.2611, found: 537.2616.

Specific rotation: $[\alpha]_D^{25}$ = -207.9 (c = 1.0, CH_2Cl_2)

HPLC analysis of the product: Daicel Chiralpak IC-3 column, hexane/*i*-PrOH = 98.5:1.5, 0.8 mL/min, λ = 210 nm, retention time: 23.46 min (major), 28.36 min (minor).

(*S*)-3-acetyl-3-((*S*)-(3,5-di-*tert*-butyl-4-hydroxyphenyl)(4-methoxyphenyl)methyl)di hydrofuran-2(3H)-one (3y) (colorless oil)

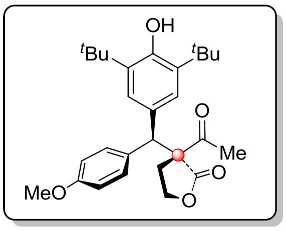

^{1}H NMR (500 MHz, $CDCl_3$) δ 7.10 (d, *J* = 8.7 Hz, 2H), 6.94 (s, 2H), 6.86 (d, *J* = 8.7 Hz, 2H), 5.25 (s, 1H), 5.18 (s, 1H), 4.06 (td, *J* = 8.3, 7.3 Hz, 1H), 3.81 (s, 3H), 3.49 (td, *J* = 8.7, 4.6 Hz, 1H), 3.30 (ddd, *J* = 12.7, 8.3, 4.6 Hz, 1H), 2.31 (ddd, *J* = 12.7, 9.1, 7.3 Hz, 1H), 2.24 (s, 3H), 1.37 (s, 18H).

^{13}C NMR (125 MHz, $CDCl_3$) δ 201.6, 175.4, 158.5, 152.9, 135.9, 131.4, 130.1, 129.9, 126.0, 114.2, 67.5, 66.3, 55.2, 53.7, 34.4, 30.2, 25.9, 25.5.

HRMS: (ESI^+) $[M+Na]^+$ calcd for $C_{28}H_{36}O_5Na$:475.2455, found: 475.2460.

Specific rotation: $[\alpha]_D^{25}$ = -120.2° (c = 0.5, CH_2Cl_2)
HPLC analysis of the product: Daicel Chiralpak AD-H column, hexane/*i*-PrOH = 97.5:2.5, 0.5 mL/min, λ = 210 nm, retention time: 15.70 min (major), 18.05 min (minor).

(*R*)-ethyl 2-((*S*)-(3,5-di-tert-butyl-4-hydroxyphenyl)(4-methoxyphenyl)methyl)-2-methyl-3-oxobutanoate (3z) (colorless oil)

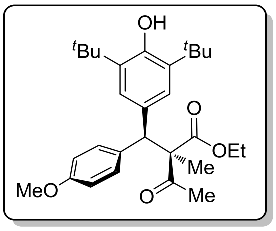

^{1}H NMR (500 MHz, $CDCl_3$)δ 7.27 (d, *J* = 8.7 Hz, 2H), 7.09 (s, 2H), 6.80 (d, *J* = 8.7 Hz, 2H), 5.09 (s, 1H), 5.08 (s, 1H), 4.09 – 4.00 (m, 2H), 3.79 (s, 3H), 2.03 (s, 3H), 1.48 (s, 3H), 1.40 (s, 18H), 1.11 (t, *J* = 7.1 Hz, 3H).

^{13}C NMR (125 MHz, $CDCl_3$) δ 205.1, 172.1, 158.1, 152.3, 135.4, 133.8, 131.0, 130.9, 126.5, 113.3, 65.5, 61.4, 55.2, 53.7, 34.4, 30.4, 27.2, 18.4, 13.8.

HRMS: (ESI^+) $[M+Na]^+$ calcd for $C_{29}H_{40}O_5Na$: 491.2768, found: 491.2775.
Specific rotation: $[\alpha]_D^{25}$ = +9.1 (c = 1.0, CH_2Cl_2)
HPLC analysis of the product: Daicel Chiralpak IC-3 column, hexane/*i*-PrOH = 97.0:3.0, 0.8 mL/min, λ = 210 nm, retention time: 7.20 min (minor), 10.36 min (major).

(*R*/*S*)-2-((*S*)-(3,5-di-*tert*-butyl-4-hydroxyphenyl)(4-methoxyphenyl)methyl)-1-phenyl butane-1,3-dione (3aa) (colorless oil)

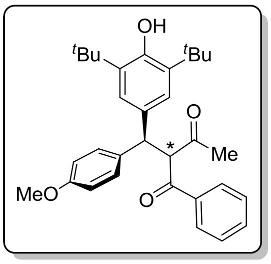

^{1}H NMR (500 MHz, $CDCl_3$) δ 7.99 – 7.93 (m, 2H), 7.58 – 7.48 (m, 1H), 7.43 (t, *J* = 7.7 Hz, 2H), 7.17 (d, *J* = 8.7 Hz, 2H), 7.13 (s, 2H), 6.70 (d, *J* = 8.7 Hz, 2H), 5.51 (d, *J* = 4.3 Hz, 1H), 5.49 (d, *J* = 4.3 Hz, 1H), 5.11 (s, 1H), 3.69 (s, 3H), 2.01 (s, 3H), 1.44 (s, 18H) (diastereoisomer **A**).

δ 7.87 – 7.80 (m, 2H), 7.58 – 7.48 (m, 1H), 7.39 (t, *J* = 7.7 Hz, 2H), 7.34 – 7.29 (m, 2H), 6.92 (s, 2H), 6.87 (d, *J* = 8.7 Hz, 2H), 4.98 (d, *J* = 11.9 Hz, 1H), 4.92 (d, *J* = 11.9 Hz, 1H), 4.91 (s, 1H), 3.80 (s, 3H), 2.13 (s, 3H), 1.24 (s, 18H) (diastereoisomer **B**).

^{13}C NMR (125 MHz, $CDCl_3$) δ 203.1, 194.7, 158.0, 152.6, 137.2, 136.1, 134.7, 133.4, 132.0, 130.0, 128.59, 128.5, 124.7, 113.9, 70.0, 55.1, 51.0, 34.4, 30.6, 27.7 (diastereoisomer **A**).

δ 203.6, 195.7, 158.4, 152.2, 137.7, 135.7, 134.0, 133.2, 132.1, 128.7, 128.63, 128.4, 124.3, 114.2, 69.3, 55.2, 51.6, 34.2, 30.1, 28.1 (diastereoisomer **B**).

HRMS: (ESI^+) $[M+Na]^+$ calcd for $C_{32}H_{38}O_4Na$: 509.2662, found: 509.2669.
Specific rotation: $[\alpha]_D^{25}$ = –14.9° (c = 1.0, CH_2Cl_2).
HPLC analysis of the product: Daicel Chiralcel OD-H column, hexane/*i*-PrOH = 98.5:1.5, 0.7 mL/min, λ = 254 nm, retention time: 9.69 min (major), 11.93 min (minor) (diastereoisomer **A**)/13.06 min (minor), 19.23 min (major) (diastereoisomer **B**).

(*R*/*S*)-2-acetyl-2-((*S*)-(3,5-di-*tert*-butyl-4-hydroxyphenyl)(4-methoxyphenyl)methyl) cyclopentanone (3ab) (colorless oil)

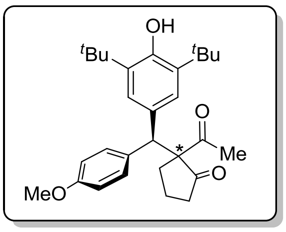

^{1}H NMR (500 MHz, $CDCl_3$) δ 7.14 (d, *J* = 8.7 Hz, 2H), 6.95 (s, 2H), 6.83 (d, *J* = 8.7 Hz, 2H), 5.23 (s, 1H), 5.10 (s, 1H), 3.80 (s, 3H), 3.14 – 3.06 (m, 1H), 2.27 – 2.13 (m, 1H), 2.11 (s, 3H), 2.09 – 1.97 (m, 1H), 1.78 – 1.65 (m, 2H), 1.50 – 1.38 (m, 1H), 1.36 (s, 18H) (diastereoisomer A).

δ 6.99 – 6.94 (m, 2H), 6.81 (s, 2H), 6.78 (d, *J* = 8.7 Hz, 2H), 5.19 (s,

1H), 5.11 (s, 1H), 3.78 (s, 3H), 3.24 – 3.14 (m, 1H), 2.27 – 2.13 (m, 1H), 2.08 – 1.98 (m, 1H), 2.06 (s, 3H), 1.77 – 1.65 (m, 2H), 1.50 – 1.38 (m, 1H), 1.41 (s, 18H) (diastereoisomer B).

^{13}C NMR (125 MHz, $CDCl_3$) δ 215.68, 203.0, 158.2, 135.5, 132.7, 131.3, 130.0, 126.2, 124.3, 114.0, 74.8, 55.2, 54.8, 34.3, 31.6, 30.3, 27.3, 26.1, 19.4 (diastereoisomer A).

δ^{13}C NMR (126 MHz, Chloroform-*d*) δ 215.72, 202.6, 152.5, 136.0, 133.4, 131.0, 130.8, 125.6, 124.9, 113.5, 75.1, 55.1, 54.6, 39.2, 31.9, 30.4, 27.5, 25.9, 19.7 (diastereoisomer B).

HRMS: (ESI$^+$) [M+Na]$^+$ calcd for $C_{29}H_{38}O_4Na$: 473.2662, found: 473.2667.

Specific rotation: $[\alpha]_D^{25}$ = -18.3° (c = 0.9, CH_2Cl_2)

HPLC analysis of the product: Daicel Chiralpak IC-3 column, hexane/*i*-PrOH = 98.5:1.5, 0.8 mL/min, λ = 210 nm, retention time: 7.04 min (major), 7.46 min (minor) (diastereoisomer **A**)/8.80 min (minor), 9.74 min (major) (diastereoisomer **B**).

(*R*)-ethyl 1-((*S*)-(3,5-di-*tert*-butyl-4-hydroxyphenyl)(4-fluorophenyl)methyl)-2-oxo-cyclopentanecarboxylate (4a) (pale yellow oil)

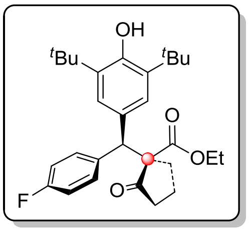

^{1}H NMR (500 MHz, $CDCl_3$) δ 7.33 – 7.22 (m, 2H), 6.98 (t, *J* = 8.7 Hz, 2H), 6.86 (s, 2H), 5.18 (s, 1H), 5.12 (s, 1H), 3.99 (dq, *J* = 10.8, 7.0 Hz, 1H), 3.90 (dq, *J* = 10.8, 7.1 Hz, 1H), 2.99 (ddd, *J* = 12.3, 7.2, 4.2 Hz, 1H), 2.28 (ddd, *J* = 18.2, 8.4, 4.6 Hz, 1H), 2.19 (ddd, *J* = 13.0, 9.2, 7.5 Hz, 1H), 1.93 – 1.78 (m, 1H), 1.70 (dt, *J* = 18.4, 9.2 Hz, 1H), 1.50 – 1.36 (m, 1H), 1.36 (s, 18H), 0.90 (t, *J* = 7.1 Hz, 3H).

^{13}C NMR (125 MHz, $CDCl_3$) δ 214.09, 168.77, 161.3 (d, *J* = 243.8 Hz), 152.51, 137.3 (d, *J* = 3.8 Hz), 135.52, 130.4, 130.3 (d, *J* = 7.5 Hz), 126.42, 115.0 (d, *J* = 21.3 Hz), 66.4, 61.6, 54.4, 38.6, 34.3, 30.2, 29.2, 19.5, 13.5.

^{19}F NMR (471 MHz, $CDCl_3$) δ -116.73.

HRMS: (ESI$^+$) [M+Na]$^+$ calcd for $C_{29}H_{37}FO_4Na$: 491.2568, found: 491.2570.

Specific rotation: $[\alpha]_D^{25}$ = –74.8° (c = 1.0, CH_2Cl_2).

HPLC analysis of the product: Daicel Chiralcel OD-H column, hexane/*i*-PrOH = 99.5:0.5, 0.5 mL/min, λ = 254 nm, retention time: 18.06 min (major), 22.09 min (minor).

(*R*)-ethyl 1-((*S*)-(4-chlorophenyl)(3,5-di-*tert*-butyl-4-hydroxyphenyl)methyl)-2-oxo-cyclopentanecarboxylate (4b) (yellow solid)

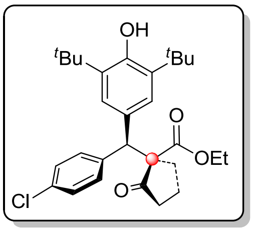

^{1}H NMR (500 MHz, $CDCl_3$) δ 7.27 (d, *J* = 8.6 Hz, 1H), 7.23 (d, *J* = 8.6 Hz, 2H), 6.85 (s, 2H), 5.16 (s, 1H), 5.12 (s, 1H), 3.99 (dq, *J* = 10.8, 7.1 Hz, 1H), 3.91 (dq, *J* = 10.8, 7.1 Hz, 1H), 2.99 (ddd, *J* = 12.3, 7.2, 4.0 Hz, 1H), 2.28 (ddd, *J* = 18.5, 8.4, 4.5 Hz, 1H), 2.23 – 2.15 (m, 1H), 1.86 (ddt, *J* = 16.8, 12.6, 7.2 Hz, 1H), 1.69 (dt, *J* = 18.4, 9.2 Hz, 1H), 1.44 – 1.38 (m, 1H), 1.36 (s, 18H), 0.92 (t, *J* = 7.1 Hz, 3H).

^{13}C NMR (125 MHz, $CDCl_3$) δ 214.0, 168.8, 152.6, 140.2, 132.1, 131.3, 130.2, 128.3, 126.5, 125.5, 66.2, 61.7, 54.6, 38.6, 34.3, 30.3, 29.3, 19.5, 13.6.

HRMS: (ESI$^+$) [M+Na]$^+$ calcd for $C_{29}H_{37}{}^{35}ClO_4Na$: 507.2273, found: 507.2279; [M+Na]$^+$ calcd for $C_{29}H_{37}{}^{37}ClO_4Na$: 509.2243, found: 509.2259.

Specific rotation: $[\alpha]_D^{25}$ = –48.0° (c = 0.5, CH_2Cl_2).

HPLC analysis of the product: Daicel Chiralcel OD-H column, hexane/*i*-PrOH = 99.5:0.5, 0.5 mL/min, λ = 210 nm, retention time: 16.60 min (minor), 17.48 min (major).

(*R*)-ethyl 1-((*S*)-(4-bromophenyl)(3,5-di-*tert*-butyl-4-hydroxyphenyl)methyl)-2-oxo-cyclopentanecarboxylate (4c) (yellow solid)

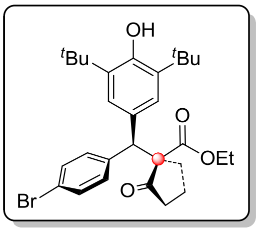

^{1}H NMR (500 MHz, $CDCl_3$) δ 7.41 (d, *J* = 8.5 Hz, 1H), 7.17 (d, *J* = 8.5 Hz, 2H), 6.85 (s, 2H), 5.14 (s, 1H), 5.12 (s, 1H), 3.99 (dq, *J* = 10.8, 7.1 Hz, 1H), 3.91 (dq, *J* = 10.8, 7.1 Hz, 1H), 2.98 (dddd, *J* = 12.9, 7.4, 4.1, 1.4 Hz, 1H), 2.32 – 2.23 (m, 1H), 2.19 (ddd, *J* = 13.1, 9.1, 7.5 Hz, 1H), 1.90 – 1.80 (m, 1H), 1.70 (dd, *J* = 18.3, 9.1 Hz, 1H), 1.45 – 1.34 (m, 1H), 1.37 (s, 18H), 0.92 (t, *J* = 7.1 Hz, 3H).

^{13}C NMR (125 MHz, $CDCl_3$) δ 214.0, 168.8, 152.6, 140.7, 135.6, 131.3, 130.6, 130.0, 126.5, 120.1, 66.2, 61.7, 54.6, 38.6, 34.3, 30.2, 29.3, 19.5, 13.6.

HRMS: (ESI$^+$) [M+Na]$^+$ calcd for $C_{29}H_{37}{}^{79}BrO_4Na$: 551.1767, found: 551.1770; [M+Na]$^+$ calcd for $C_{29}H_{37}{}^{81}BrO_4Na$: 553.1747, found: 553.1755.

Specific rotation: $[\alpha]_D^{25}$ = –62.8° (c = 1.0, CH_2Cl_2).

HPLC analysis of the product: Daicel Chiralcel OD-H column, hexane/*i*-PrOH = 99.5:0.5, 0.5 mL/min, λ = 210 nm, retention time: 17.55 min (major), 23.51 min (minor).

methyl 4-((*S*)-(3,5-di-*tert*-butyl-4-hydroxyphenyl)((*R*)-1-(ethoxycarbonyl)-2-oxocyclopentyl)methyl)benzoate (4d) (colorless oil)

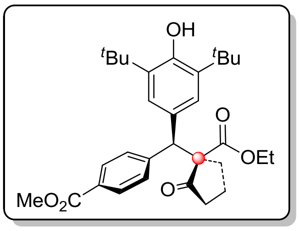

^{1}H NMR (500 MHz, $CDCl_3$) δ 7.97 (d, *J* = 8.4 Hz, 2H), 7.37 (d, *J* = 8.4 Hz, 2H), 6.83 (s, 2H), 5.25 (s, 1H), 5.13 (s, 1H), 3.99 – 3.93 (m, 1H), 3.92 (s, 3H), 3.91 – 3.85 (m, 1H), 3.03 (dddd, *J* = 12.9, 7.4, 4.3, 1.3 Hz, 1H), 2.34 – 2.19 (m, 2H), 1.92 – 1.81 (m, 1H), 1.76 – 1.65 (m, 1H), 1.42 – 1.36 (m, 1H), 1.35 (s, 18H), 0.86 (t, *J* = 7.1 Hz, 3H).

^{13}C NMR (125 MHz, $CDCl_3$) δ 213.8, 168.8, 167.0, 152.6, 147.3, 135.7, 129.9, 129.5, 128.9, 128.1, 126.6, 66.1, 61.6, 55.1, 52.0, 38.6, 34.3, 30.3, 29.4, 19.5, 13.5.

HRMS: (ESI$^+$) [M+Na]$^+$ calcd for $C_{31}H_{40}O_6Na$: 531.2717, found: 531.2720.

Specific rotation: $[\alpha]_D^{25}$ = -98.5° (c = 1.0, CH_2Cl_2)

HPLC analysis of the product: Daicel Chiralpak IC-3 column, hexane/*i*-PrOH = 90.0:10.0, 0.8 mL/min, λ = 254 nm, retention time: 7.65 min (major), 7.94 min (minor).

(*R*)-ethyl 1-((*S*)-(4-cyanophenyl)(3,5-di-*tert*-butyl-4-hydroxyphenyl)methyl)-2-oxocyclopentanecarboxylate (4e) (colorless oil)

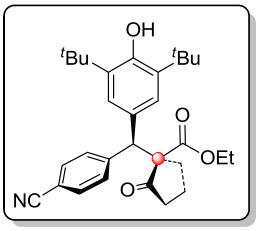

^{1}H NMR (500 MHz, $CDCl_3$) δ 7.60 (d, *J* = 8.4 Hz, 2H), 7.41 (d, *J* = 8.4 Hz, 2H), 6.79 (s, 2H), 5.24 (s, 1H), 5.16 (s, 1H), 3.99 (dq, *J* = 10.8, 7.1 Hz, 1H), 3.91 (dq, *J* = 10.8, 7.1 Hz, 1H), 3.04 – 2.96 (m, 1H), 2.34 – 2.26 (m, 1H), 2.21 (ddd, *J* = 13.1, 9.0, 7.6 Hz, 1H), 1.93 – 1.82 (m, 1H), 1.70 (dt, *J* = 18.4, 9.2 Hz, 1H), 1.44 – 1.36 (m, 1H), 1.35 (s, 18H), 0.90 (t, *J* = 7.1 Hz, 3H).

^{13}C NMR (125 MHz, $CDCl_3$) δ 213.4, 168.7, 152.8, 147.6, 135.9, 132.0, 129.6, 129.3, 126.5, 118.8, 110.2, 65.8, 61.8, 55.1, 38.4, 34.3, 30.2, 29.4, 19.5, 13.6.

HRMS: (ESI$^+$) [M+Na]$^+$ calcd for $C_{30}H_{37}NO_4Na$: 498.2615, found: 498.2617.

Specific rotation: $[\alpha]_D^{25}$ = -98.9° (c = 1.0, CH_2Cl_2)

HPLC analysis of the product: Daicel Chiralpak IC-3 column, hexane/*i*-PrOH = 97.0:3.0, 0.8 mL/min, λ = 254 nm, retention time: 20.77 min (major), 22.26 min (minor).

(*R*)-ethyl 1-((*S*)-(3,5-di-*tert*-butyl-4-hydroxyphenyl)(4-(trifluoromethyl)phenyl)methyl)-2-oxocyclopentanecarboxylate (4f) (colorless oil)

^{1}H NMR (500 MHz, $CDCl_3$) δ 7.56 (d, *J* = 8.1 Hz, 2H), 7.42 (d, *J* = 8.1 Hz, 2H), 6.84 (s, 2H),

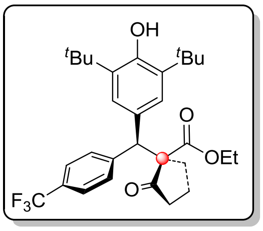

5.24 (s, 1H), 5.14 (s, 1H), 3.98 (dq, J = 10.8, 7.1 Hz, 1H), 3.90 (dq, J = 10.8, 7.1 Hz, 1H), 3.02 (ddd, J = 12.3, 7.4, 4.2 Hz, 1H), 2.34 – 2.20 (m, 2H), 1.94 – 1.83 (m, 1H), 1.71 (dt, J = 18.3, 9.1 Hz, 1H), 1.46 – 1.36 (m, 1H), 1.36 (s, 18H), 0.88 (t, J = 7.1 Hz, 3H).

^{13}C NMR (125 MHz, $CDCl_3$) δ 213.6, 168.8, 152.7, 146.0, 135.8, 130.3, 129.2, 128.6 (q, J = 39.0 Hz), 126.6, 125.1 (q, J = 4.5 Hz), 124.2 (q, J = 324.0 Hz), 66.0, 61.7, 55.0, 38.5, 34.3, 30.2, 29.5, 19.5, 13.5.

^{19}F NMR (471 MHz, $CDCl_3$) δ -62.44.

HRMS: (ESI$^+$) $[M+Na]^+$ calcd for $C_{30}H_{37}F_3O_4Na$: 541.2536, found: 541.2531.

Specific rotation: $[\alpha]_D^{25}$ = –82.3° (c = 1.0, CH_2Cl_2).

HPLC analysis of the product: Daicel Chiralcel OD-H column, hexane/*i*-PrOH = 98.5:1.5, 0.7 mL/min, λ = 210 nm, retention time: 6.21 min (major), 6.70 min (minor).

(*R*)-ethyl 1-((*S*)-(3,5-di-*tert*-butyl-4-hydroxyphenyl)(4-nitrophenyl)methyl)-2-oxocyclopentanecarboxylate (4g) (colorless oil)

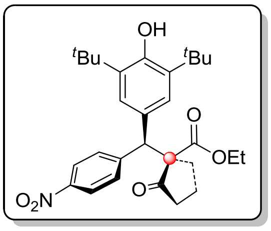

^{1}H NMR (500 MHz, $CDCl_3$) δ 8.18 (d, J = 8.8 Hz, 2H), 7.46 (d, J = 8.8 Hz, 2H), 6.81 (s, 2H), 5.30 (s, 1H), 5.17 (s, 1H), 4.01 – 3.96 (m, 1H), 3.96 – 3.91 (m, 1H), 3.02 (ddd, J = 12.4, 7.4, 4.3 Hz, 2H), 2.37 – 2.20 (m, 2H), 1.96 – 1.85 (m, 1H), 1.71 (dt, J = 18.5, 9.2 Hz, 1H), 1.47 – 1.36 (m, 1H), 1.36 (s, 18H), 0.92 (t, J = 7.1 Hz, 3H).

^{13}C NMR (125 MHz, $CDCl_3$) δ 213.3, 168.7, 152.9, 149.8, 146.4, 136.0, 130.9, 129.7, 126.6, 123.5, 65.8, 61.8, 54.9, 38.4, 34.4, 31.6, 30.2, 19.5, 13.6.

HRMS: (ESI$^+$) $[M+Na]^+$ calcd for $C_{29}H_{37}NO_6Na$: 518.2513, found: 518.2519.

Specific rotation: $[\alpha]_D^{25}$ = -94.4° (c = 0.5, CH_2Cl_2)

HPLC analysis of the product: Daicel Chiralpak IC-3 column, hexane/*i*-PrOH = 98.5:1.5, 0.8 mL/min, λ = 254 nm, retention time: 20.63 min (major), 22.87 min (minor).

(*R*)-ethyl 1-((*S*)-(4-acetoxyphenyl)(3,5-di-*tert*-butyl-4-hydroxyphenyl)methyl)-2-oxocyclopentanecarboxylate (4h) (colorless oil)

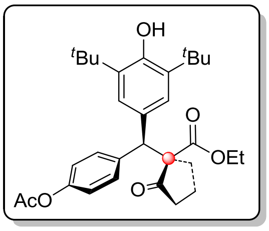

^{1}H NMR (500 MHz, $CDCl_3$) δ 7.30 (d, J = 8.5 Hz, 2H), 7.02 (d, J = 8.5 Hz, 1H), 6.88 (s, 2H), 5.18 (s, 1H), 5.11 (s, 1H), 4.02 (dq, J = 10.6, 7.1 Hz, 1H), 3.89 (dq, J = 10.6, 7.1 Hz, 1H), 3.00 (dddd, J = 12.9, 7.4, 4.1, 1.3 Hz, 1H), 2.37 – 2.16 (m, 2H), 2.29 (s, 3H), 1.92 – 1.78 (m, 1H), 1.70 (dt, J = 18.4, 9.1 Hz, 1H), 1.47 – 1.33 (m, 1H), 1.36 (s, 18H), 0.89 (t, J = 7.1 Hz, 3H).

^{13}C NMR (125 MHz, $CDCl_3$) δ 214.0, 169.1, 168.9, 152.5, 149.2, 139.2, 135.6, 130.5, 129.8, 126.6, 121.2, 66.4, 61.7, 54.7, 38.6, 34.3, 30.3, 29.5, 21.1, 19.5, 13.5.

HRMS: (ESI$^+$) $[M+Na]^+$ calcd for $C_{31}H_{40}O_6Na$: 531.2717, found: 531.2722.

Specific rotation: $[\alpha]_D^{25}$ = -79.9° (c = 1.0, CH_2Cl_2)

HPLC analysis of the product: Daicel Chiralpak IC-3 column, hexane/*i*-PrOH = 97.5:2.5, 0.6 mL/min, λ = 210 nm, retention time: 18.97 min (minor), 20.95 min (major).

(*R*)-ethyl 1-((*S*)-(3,5-di-*tert*-butyl-4-hydroxyphenyl)(4-ethoxyphenyl)methyl)-2-oxocyclopentanecarboxylate (4i) (yellow oil)

^{1}H NMR (500 MHz, $CDCl_3$) δ 7.20 (d, J = 8.7 Hz, 2H), 6.90 (s, 2H), 6.81 (d, J = 8.7 Hz, 2H), 5.12 (s, 1H), 5.09 (s, 1H), 4.07 – 3.94 (m, 3H), 3.89 (dq, J = 10.7, 7.1 Hz, 1H), 3.05 – 2.93 (m, 1H), 2.32 – 2.22 (m, 1H), 2.22 – 2.15 (m, 1H), 1.89 – 1.75 (m, 1H), 1.70 (dd, J = 18.3, 9.1 Hz,

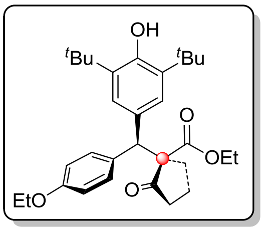

1H), 1.44 – 1.38 (m, 1H), 1.41 (t, *J* = 7.1 Hz, 3H), 1.36 (s, 18H), 0.90 (t, *J* = 7.1 Hz, 3H).

^{13}C NMR (125 MHz, $CDCl_3$) δ 214.3, 169.0, 157.3, 152.4, 135.4, 133.5, 131.1, 130.0, 126.5, 114.2, 66.6, 63.4, 61.5, 54.6, 38.7, 34.3, 30.3, 29.4, 19.5, 14.8, 13.6.

HRMS: (ESI$^+$) [M+Na]$^+$ calcd for $C_{31}H_{42}O_5Na$: 517.2924, found: 517.2928.

Specific rotation: $[\alpha]_D^{25}$ = -102.5° (c =1.0, CH_2Cl_2)

HPLC analysis of the product: Daicel Chiralpak AD-H column, hexane/*i*-PrOH = 98.5:1.5, 0.5 mL/min, λ = 210 nm, retention time: 15.62 min (minor), 16.39 min (major).

(*R*)-ethyl 1-((*S*)-(3,5-di-*tert*-butyl-4-hydroxyphenyl)(4-(diphenylamino)phenyl)methyl)-2-oxocyclopentanecarboxylate (4j) (yellow oil)

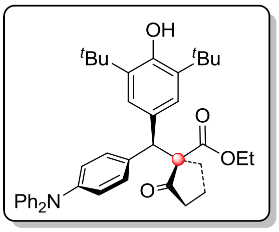

^{1}H NMR (500 MHz, $CDCl_3$) δ 7.28 – 7.17 (m, 6H), 7.07 – 7.02 (m, 6H), 7.00 (t, *J* = 8.4 Hz, 2H), 6.92 (s, 2H), 5.16 (s, 1H), 5.13 (s, 1H), 4.13 (dq, *J* = 10.7, 7.1 Hz, 1H), 3.93 (dq, *J* = 10.7, 7.1 Hz, 1H), 2.99 (tt, *J* = 10.7, 3.3 Hz, 1H), 2.34 – 2.25 (m, 1H), 2.18 (ddd, *J* = 13.1, 9.2, 7.4 Hz, 1H), 1.91 – 1.80 (m, 1H), 1.72 (dt, *J* = 18.5, 9.2 Hz, 1H), 1.50 – 1.36 (m, 1H), 1.39 (s, 18H), 0.96 (t, *J* = 7.1 Hz, 3H).

^{13}C NMR (125 MHz, $CDCl_3$) δ 214.4, 168.8, 152.4, 147.8, 145.9, 136.2, 135.4, 130.8, 129.8, 129.1, 126.5, 124.4, 123.7, 122.4, 66.6, 61.6, 54.7, 38.7, 34.4, 30.3, 29.3, 19.6, 13.6.

HRMS: (ESI$^+$) [M+K]$^+$ calcd for $C_{41}H_{47}NO_4K$: 656.3137, found: 656.3136.

Specific rotation: $[\alpha]_D^{25}$ = -60.2° (c = 1.0, CH_2Cl_2)

HPLC analysis of the product: Daicel Chiralpak IA-3 column, hexane/*i*-PrOH = 98.0:2.0, 0.35 mL/min, λ = 280 nm, retention time: 14.52 min (major), 17.69 min (minor).

(*R*)-ethyl 1-((*S*)-(3,5-di-*tert*-butyl-4-hydroxyphenyl)(4-(pyrrolidin-1-yl)phenyl)methyl)-2-oxocyclopentanecarboxylate (4k) (red oil)

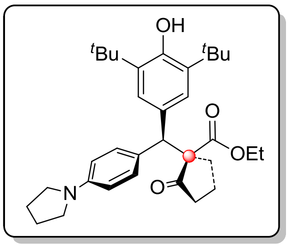

^{1}H NMR (500 MHz, $CDCl_3$) δ 7.14 (d, *J* = 8.7 Hz, 2H), 6.98 (s, 2H), 6.48 (d, *J* = 8.7 Hz, 2H), 5.05 (s, 1H), 5.02 (s, 1H), 3.99 – 3.94 (m, 1H), 3.94 – 3.87 (m, 1H), 3.26 (ddd, *J* = 7.3, 4.9, 2.4 Hz, 4H), 2.98 (dddd, *J* = 12.8, 7.2, 3.9, 1.4 Hz, 1H), 2.30 – 2.18 (m, 2H), 2.00 (td, *J* = 7.7, 7.1, 3.9 Hz, 4H), 1.84 – 1.74 (m, 1H), 1.73 – 1.65 (m, 1H), 1.46 – 1.32 (m, 1H), 1.37 (s, 18H), 0.95 (t, *J* = 7.1 Hz, 3H).

^{13}C NMR (125 MHz, $CDCl_3$) δ 214.5, 169.2, 152.2, 146.5, 135.2, 131.7, 129.8, 128.1, 126.5, 111.5, 66.9, 61.4, 54.8, 47.7, 38.9, 34.3, 30.4, 29.6, 25.4, 19.6, 13.7.

HRMS: (ESI$^+$) [M+Na]$^+$ calcd for $C_{33}H_{45}NO_4Na$: 542.3241, found: 542.3244.

Specific rotation: $[\alpha]_D^{25}$ = –53.6° (c = 0.25, CH_2Cl_2).

HPLC analysis of the product: Daicel Chiralcel OD-H column, hexane/*i*-PrOH = 98.5:1.5, 0.7 mL/min, λ = 254 nm, retention time: 9.15 min (minor), 11.46 min (major).

(*R*)-ethyl 1-((*S*)-(3,5-di-*tert*-butyl-4-hydroxyphenyl)(4-morpholinophenyl)methyl)-2-oxocyclopentanecarboxylate (4l) (brown oil)

^{1}H NMR (500 MHz, $CDCl_3$) δ 7.21 (d, *J* = 8.7 Hz, 2H), 6.92 (s, 2H), 6.84 (d, *J* = 8.7 Hz, 2H), 5.08 (s, 2H), 3.98 (dq, *J* = 10.7, 7.1 Hz, 1H), 3.94 – 3.83 (m, 5H), 3.12 (q, *J* = 4.3 Hz, 4H), 2.98 (dddd, *J* = 12.8, 7.2, 3.9, 1.4 Hz, 1H), 2.30 – 2.16 (m, 2H), 1.88 – 1.76 (m, 1H), 1.68 (dt, *J* = 18.4, 9.2 Hz, 1H), 1.44 – 1.34 (m, 1H), 1.36 (s, 18H), 0.90 (t, *J* = 7.1 Hz, 3H).

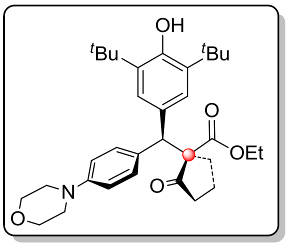

^{13}C NMR (125 MHz, $CDCl_3$) δ 214.3, 169.0, 152.3, 135.4, 131.0, 130.7, 129.7, 126.5, 125.6, 115.5, 66.9, 66.6, 61.5, 54.6, 49.6, 38.7, 34.3, 30.3, 29.7, 19.5, 13.6.

HRMS: (ESI$^+$) $[M+Na]^+$ calcd for $C_{33}H_{45}NO_4Na$: 558.3190, found: 558.3183.

Specific rotation: $[\alpha]_D^{25}$ = -42.4° (c = 1.1, CH_2Cl_2)

HPLC analysis of the product: Daicel Chiralpak IC-3 column, hexane/*i*-PrOH = 90.0:10.0, 0.8 mL/min, λ = 254 nm, retention time: 7.39 min (minor), 8.02 min (major).

(*R*)-ethyl 1-((*S*)-(3,5-di-*tert*-butyl-4-hydroxyphenyl)(p-tolyl)methyl)-2-oxocyclopentane-carboxylate (4m) (colorless oil)

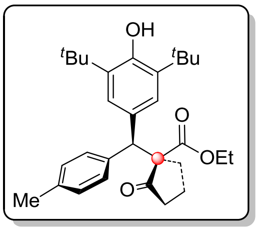

^{1}H NMR (500 MHz, $CDCl_3$) δ 7.22 – 7.13 (m, 2H), 7.08 (d, *J* = 7.8 Hz, 2H), 6.91 (s, 2H), 5.13 (s, 1H), 5.09 (s, 1H), 3.97 (dq, *J* = 8.6, 7.11 Hz, 2H), 3.90 (dq, *J* = 8.6, 7.1 Hz, 2H), 3.00 (ddd, *J* = 12.1, 7.2, 4.0 Hz, 1H), 2.34 – 2.17 (m, 2H), 2.31 (s, 3H), 1.82 (ddd, *J* = 20.8, 12.9, 5.1 Hz, 1H), 1.69 (dt, *J* = 18.5, 9.2 Hz, 1H), 1.44 – 1.36 (m, 1H), 1.37 (s, 18H), 0.90 (t, *J* = 7.1 Hz, 3H).

^{13}C NMR (125 MHz, $CDCl_3$) δ 214.5, 169.0, 152.4, 138.3, 135.6, 135.3, 130.9, 128.8, 128.8, 126.5, 66.5, 61.5, 55.0, 38.8, 34.3, 30.3, 29.4, 21.0, 19.6, 13.5.

HRMS: (ESI$^+$) $[M+Na]^+$ calcd for $C_{30}H_{40}O_4Na$: 487.2819, found: 487.2818.

Specific rotation: $[\alpha]_D^{25}$ = -76.2° (c = 0.9, CH_2Cl_2)

HPLC analysis of the product: Daicel Chiralpak IC-3 column, hexane/*i*-PrOH = 98.5:1.5, 0.7 mL/min, λ = 210 nm, retention time: 7.58 min (minor), 8.17 min (major).

(*R*)-ethyl 1-((*S*)-(4-(tert-butyl)phenyl)(3,5-di-*tert*-butyl-4-hydroxyphenyl)methyl)-2-oxo-cyclopentanecarboxylate (4n) (colorless oil)

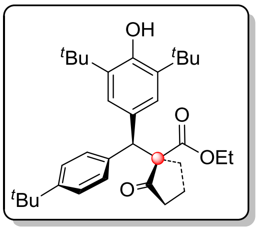

^{1}H NMR (500 MHz, $CDCl_3$) δ 7.28 (d, *J* = 8.3 Hz, 2H), 7.22 (d, *J* = 8.3 Hz, 2H), 6.93 (s, 2H), 5.12 (s, 1H), 5.08 (s, 1H), 4.01 (dq, *J* = 10.8, 7.1 Hz, 1H), 3.86 (dq, *J* = 10.8, 7.1 Hz, 1H), 3.01 (ddt, *J* = 12.3, 7.7, 3.3 Hz, 1H), 2.25 (dddd, *J* = 17.2, 13.2, 8.6, 5.7 Hz, 2H), 1.89 – 1.77 (m, 1H), 1.70 (dt, *J* = 18.5, 9.2 Hz, 1H), 1.44 – 1.36 (m, 1H), 1.36 (s, 18H), 1.29 (s, 9H), 0.81 (t, *J* = 7.1 Hz, 3H).

^{13}C NMR (125 MHz, $CDCl_3$) δ 214.4, 169.0, 152.4, 149.1, 138.3, 135.4, 130.9, 128.6, 126.6, 125.0, 66.6, 61.5, 55.0, 38.7, 34.3, 31.3, 30.4, 30.3, 29.6, 19.6, 13.3.

HRMS: (ESI$^+$) $[M+Na]^+$ calcd for $C_{33}H_{46}O_4Na$: 529.3288, found: 529.3291.

Specific rotation: $[\alpha]_D^{25}$ = –87.9° (c = 1.0, CH_2Cl_2).

HPLC analysis of the product: Daicel Chiralcel OD-H column, hexane/*i*-PrOH = 99.5:0.5, 0.7 mL/min, λ = 210 nm, retention time: 8.27 min (minor), 10.98 min (major).

(*R*)-ethyl 1-((*S*)-[1,1'-biphenyl]-4-yl(3,5-di-*tert*-butyl-4-hydroxyphenyl)methyl)-2-oxo-cyclopentanecarboxylate (4o) (yellow solid)

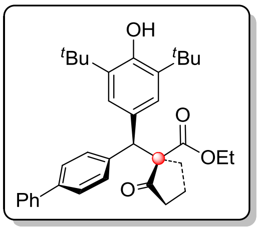

^{1}H NMR (500 MHz, $CDCl_3$) δ 7.65 – 7.59 (m, 2H), 7.55 (d, *J* = 8.4 Hz, 2H), 7.45 (t, *J* = 7.8 Hz, 2H), 7.38 (d, *J* = 8.4 Hz, 2H), 7.37 – 7.32 (m, 1H), 6.94 (s, 2H), 5.23 (s, 1H), 5.12 (s, 1H), 4.01 (dq, *J* = 10.8, 7.1 Hz, 1H), 3.91 (dq, *J* = 10.8, 7.1 Hz, 1H), 3.06 (dddd, *J* = 12.9, 7.3, 4.1, 1.4 Hz, 1H), 2.41 – 2.22 (m, 2H), 1.87 (ddt, *J* = 20.1, 9.6, 3.2 Hz, 1H),

1.72 (dt, *J* = 18.5, 9.2 Hz, 1H), 1.47 – 1.33 (m, 1H), 1.37 (s, 18H), 0.89 (t, *J* = 7.1 Hz, 3H).

^{13}C NMR (125 MHz, $CDCl_3$) δ 214.3, 168.9, 152.5, 140.7, 140.7, 138.9, 135.5, 130.5, 129.3, 128.7, 127.1, 126.9, 126.8, 126.6, 66.4, 61.6, 55.0, 38.7, 34.3, 30.3, 29.5, 19.6, 13.5.

HRMS: (ESI$^+$) $[M+Na]^+$ calcd for $C_{35}H_{42}O_4Na$: 549.2975, found: 549.2974.

Specific rotation: $[\alpha]_D^{25}$ = -94.2° (c = 0.9, CH_2Cl_2)

HPLC analysis of the product: Daicel Chiralpak IA-3 column, hexane/*i*-PrOH = 97.0:3.0, 0.5 mL/min, *λ* = 210 nm, retention time: 11.19 min (minor), 12.60 min (major).

(*R*)-ethyl 1-((*S*)-(3,5-di-*tert*-butyl-4-hydroxyphenyl)(phenyl)methyl)-2-oxocyclopentanecarboxylate (4p) (colorless oil)

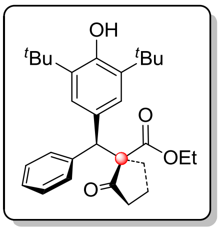

^{1}H NMR (500 MHz, $CDCl_3$) δ 7.33 – 7.08 (m, 5H), 6.89 (s, 2H), 5.19 (s, 1H), 5.10 (s, 1H), 3.97 (dq, *J* = 10.8, 7.1 Hz, 1H), 3.87 (dq, *J* = 10.8, 7.1 Hz, 1H), 3.11 – 2.97 (m, 1H), 2.26 (dddd, *J* = 22.4, 13.2, 7.1, 4.4 Hz, 2H), 1.92 – 1.80 (m, 1H), 1.70 (dt, *J* = 18.3, 9.2 Hz, 1H), 1.46 – 1.34 (m, 1H), 1.36 (s, 18H), 0.85 (t, *J* = 7.1 Hz, 3H).

^{13}C NMR (125 MHz, $CDCl_3$) δ 214.3, 169.0, 152.4, 141.6, 135.5, 130.8, 128.9, 128.2, 126.6, 126.2, 66.4, 61.5, 55.3, 38.7, 34.3, 30.3, 29.5, 19.6, 13.5.

HRMS: (ESI$^+$) $[M+Na]^+$ calcd for $C_{29}H_{38}O_4Na$: 473.2662, found: 473.2662.

Specific rotation: $[\alpha]_D^{25}$ = –72.7° (c = 1.0, CH_2Cl_2).

HPLC analysis of the product: Daicel Chiralcel OD-H column, hexane/*i*-PrOH = 98.5:1.5, 0.7 mL/min, *λ* = 210 nm, retention time: 6.67 min (minor), 7.69 min (major).

(*R*)-ethyl 1-((R)-(3,5-di-tert-butyl-4-hydroxyphenyl)(2-methoxyphenyl)methyl)-2-oxocyclopentanecarboxylate (4q) (colorless oil)

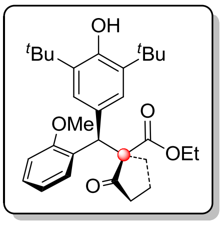

^{1}H NMR (500 MHz, $CDCl_3$) δ 7.33 (dd, *J* = 7.6, 1.6 Hz, 1H), 7.18 (td, *J* = 7.8, 1.6 Hz, 1H), 6.94 (s, 2H), 6.91 (td, *J* = 7.6, 1.1 Hz, 1H), 6.82 (dd, *J* = 8.2, 1.1 Hz, 1H), 5.66 (s, 1H), 5.06 (s, 1H), 3.97 (dq, *J* = 10.7, 7.1 Hz, 1H), 3.82 (dq, *J* = 10.7, 7.1 Hz, 1H), 3.74 (s, 3H), 3.09 – 2.97 (m, 1H), 2.31 – 2.16 (m, 2H), 1.88 – 1.75 (m, 1H), 1.70 (dt, *J* = 18.2, 9.0 Hz, 1H), 1.46 – 1.29 (m, 1H), 1.36 (s, 18H), 0.80 (t, *J* = 7.1 Hz, 3H).

^{13}C NMR (125 MHz, $CDCl_3$) δ 214.5, 169.0, 157.3, 152.3, 135.2, 130.4, 130.3, 128.3, 127.4, 126.4, 119.9, 111.0, 66.5, 61.3, 55.7, 47.7, 38.8, 34.3, 30.4, 29.9, 19.6, 13.4.

HRMS: (ESI$^+$) $[M+Na]^+$ calcd for $C_{30}H_{40}O_5Na$: 503.2768, found: 503.2774.

Specific rotation: $[\alpha]_D^{25}$ = -32.4° (c = 1.0, CH_2Cl_2)

HPLC analysis of the product: Daicel Chiralpak IC-3 column, hexane/*i*-PrOH = 98.5:1.5, 0.8 mL/min, *λ* = 280 nm, retention time: 11.59 min (major), 16.17 min (minor).

(*R*)-ethyl 1-((*R*)-(3,5-di-*tert*-butyl-4-hydroxyphenyl)(3-methoxyphenyl)methyl)-2-oxocyclopentanecarboxylate (4r) (colorless oil)

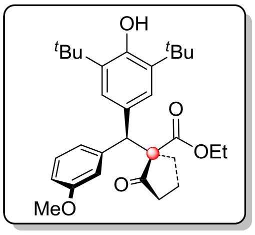

^{1}H NMR (500 MHz, $CDCl_3$) δ 7.20 (t, *J* = 7.9 Hz, 1H), 6.92 (s, 2H), 6.91 – 6.88 (m, 1H), 6.85 (t, *J* = 2.1 Hz, 1H), 6.75 (dd, *J* = 8.2, 2.5 Hz, 1H), 5.14 (s, 1H), 5.10 (s, 1H), 4.00 (dq, *J* = 10.8, 7.1 Hz, 1H), 3.89 (dq, *J* = 10.8, 7.1 Hz, 1H), 3.79 (s, 3H), 3.10 – 2.97 (m, 1H), 2.36 – 2.17 (m, 2H), 1.83 (dddd, *J* = 18.0, 14.6, 9.1, 7.4 Hz, 1H), 1.70 (dd, *J* = 18.3, 9.1 Hz, 1H), 1.42 – 1.32 (m, 1H), 1.37 (s, 18H), 0.89 (t, *J* = 7.1 Hz, 3H).

^{13}C NMR (125 MHz, $CDCl_3$) δ 214.4, 168.8, 159.4, 152.5, 143.0, 135.4, 130.4, 129.1, 126.5,

121.2, 114.8, 112.0, 66.5, 61.5, 55.4, 55.1, 38.7, 34.3, 30.3, 29.5, 19.6, 13.5.

HRMS: (ESI$^+$) [M+Na]$^+$ calcd for $C_{30}H_{40}O_5Na$: 503.2768, found: 503.2770.

Specific rotation: $[\alpha]_D^{25}$ = -68.1° (c = 1.0, CH_2Cl_2)

HPLC analysis of the product: Daicel Chiralpak IC-3 column, hexane/*i*-PrOH = 98.5:1.5, 0.8 mL/min, λ = 280 nm, retention time: 11.64 min (major), 12.96 min (minor).

(*R*)-ethyl 1-((*R*)-(3,5-di-*tert*-butyl-4-hydroxyphenyl)(3,4-dimethoxyphenyl)methyl)-2-oxocyclopentanecarboxylate (4s) (yellow oil)

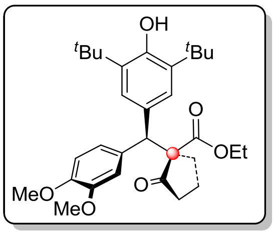

^{1}H NMR (500 MHz, $CDCl_3$) δ 6.94 (s, 2H), 6.90 – 6.82 (m, 2H), 6.80 (d, *J* = 8.3 Hz, 1H), 5.10 (s, 1H), 5.09 (s, 1H), 3.98 (dq, *J* = 10.9, 7.1 Hz, 1H), 3.88 (dq, *J* = 10.9, 7.1 Hz, 1H), 3.87 (s, 3H), 3.85 (s, 3H), 3.03 – 2.95 (m, 1H), 2.27 (ddd, *J* = 18.3, 8.1, 4.0 Hz, 1H), 2.18 (ddd, *J* = 13.1, 9.5, 7.4 Hz, 1H), 1.88 – 1.78 (m, 1H), 1.69 (dt, *J* = 18.5, 9.2 Hz, 1H), 1.44 – 1.35 (m, 1H), 1.37 (s, 18H), 0.91 (t, *J* = 7.1 Hz, 3H).

^{13}C NMR (125 MHz, $CDCl_3$) δ 214.2, 168.9, 152.4, 148.6, 147.5, 135.5, 134.1, 130.8, 126.4, 120.8, 113.1, 110.8, 66.8, 61.5, 55.82, 55.79, 55.0, 38.7, 34.3, 30.3, 29.5, 19.6, 13.7.

HRMS: (ESI$^+$) [M+Na]$^+$ calcd for $C_{31}H_{42}O_6Na$: 533.2874, found: 533.2878.

Specific rotation: $[\alpha]_D^{25}$ = -64.9° (c = 1.0, CH_2Cl_2)

HPLC analysis of the product: Daicel Chiralpak IC-3 column, hexane/*i*-PrOH = 95.0:5.0, 0.8 mL/min, λ = 280 nm, retention time: 15.51 min (major), 18.37 min (minor).

(*R*)-ethyl 1-((*R*)-(3,5-di-*tert*-butyl-4-hydroxyphenyl)(2,4-dimethoxyphenyl)methyl)-2-oxocyclopentanecarboxylate (4t) (colorless oil)

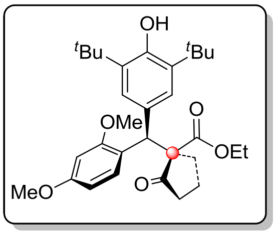

^{1}H NMR (500 MHz, $CDCl_3$) δ 7.24 (d, *J* = 8.4 Hz, 1H), 6.95 (s, 2H), 6.45 (dd, *J* = 8.4, 2.5 Hz, 1H), 6.40 (d, *J* = 2.5 Hz, 1H), 5.56 (s, 1H), 5.05 (s, 1H), 4.01 – 3.92 (m, 1H), 3.91 – 3.82 (m, 1H), 3.81 (s, 3H), 3.72 (s, 3H), 2.99 (dddd, *J* = 12.9, 7.3, 4.1, 1.5 Hz, 1H), 2.25 (dddd, *J* = 18.0, 8.1, 4.1, 1.4 Hz, 1H), 2.17 (ddd, *J* = 13.1, 9.2, 7.4 Hz, 1H), 1.85 – 1.74 (m, 1H), 1.69 (ddd, *J* = 18.3, 9.8, 8.6 Hz, 1H), 1.41 – 1.32 (m, 1H), 1.37 (s, 18H), 0.86 (t, *J* = 7.1 Hz, 3H).

^{13}C NMR (125 MHz, $CDCl_3$) δ 214.5, 169.1, 159.2, 158.3, 152.2, 135.2, 130.8, 128.9, 126.4, 122.8, 103.3, 98.9, 66.7, 61.3, 55.6, 55.2, 47.2, 38.8, 34.3, 30.4, 19.6, 13.5.

HRMS: (ESI$^+$) [M+Na]$^+$ calcd for $C_{31}H_{42}O_6Na$: 533.2874, found: 533.2876.

Specific rotation: $[\alpha]_D^{25}$ = -30.1° (c =1.0, CH_2Cl_2)

HPLC analysis of the product: Daicel Chiralpak AD-H column, hexane/*i*-PrOH = 97.0:3.0, 0.5 mL/min, λ = 210 nm, retention time: 17.01 min (minor), 20.67 min (major).

(*R*)-ethyl 1-((*R*)-(3,5-di-*tert*-butyl-4-hydroxyphenyl)(2,3-dihydrobenzo[*b*][1,4]dioxin-7-yl)methyl)-2-oxocyclopentanecarboxylate (4u) (colorless oil)

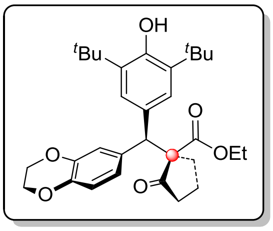

^{1}H NMR (500 MHz, $CDCl_3$) δ 6.92 (s, 2H), 6.81 (d, *J* = 1.2 Hz, 1H), 6.77 (d, *J* = 1.2 Hz, 2H), 5.09 (s, 1H), 5.04 (s, 1H), 4.24 (d, *J* = 1.9 Hz, 4H), 4.04 (dq, *J* = 10.7, 7.1 Hz, 1H), 3.93 (dq, *J* = 10.7, 7.1 Hz, 1H), 2.97 (dtd, *J* = 12.9, 5.5, 4.5, 2.7 Hz, 1H), 2.24 (tddd, *J* = 20.5, 13.2, 6.7, 4.5 Hz, 2H), 1.81 (tdd, *J* = 17.2, 15.7, 7.1, 4.6 Hz, 1H), 1.67 (dt, *J* = 18.5, 9.3 Hz, 1H), 1.46 – 1.36 (m, 1H), 1.37 (s, 18H), 0.96 (t, *J* = 7.1 Hz, 3H).

^{13}C **NMR** (125 MHz, $CDCl_3$) δ 214.2, 169.0, 152.4, 143.1, 141.9, 135.4, 134.9, 130.8, 126.5, 121.9, 118.0, 116.7, 66.5, 64.4, 64.3, 61.5, 54.6, 38.7, 34.3, 30.3, 29.4, 19.6, 13.6.

HRMS: (ESI+) [M+Na]+ calcd for $C_{31}H_{40}O_6Na$: 531.2717, found: 531.2722.

Specific rotation: $[\alpha]_D^{25}$ = -97.2° (c = 0.5, CH_2Cl_2)

HPLC analysis of the product: Daicel Chiralpak IC-3 column, hexane/*i*-PrOH = 95.0:5.0, 0.8 mL/min, λ = 210 nm, retention time: 10.11 min (minor), 10.60 min (major).

(*R*)-ethyl 1-((*R*)-(3,5-di-*tert*-butyl-4-hydroxyphenyl)(naphthalen-1-yl)methyl)-2-oxo-cyclopentanecarboxylate (4v) (yellow solid)

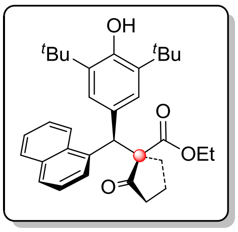

^{1}H **NMR** (500 MHz, $CDCl_3$) δ 8.24 – 8.14 (m, 1H), 7.86 – 7.77 (m, 1H), 7.73 (d, *J* = 8.2 Hz, 1H), 7.50 (dd, *J* = 7.3, 1.1 Hz, 1H), 7.47 – 7.41 (m, 3H), 6.95 (s, 2H), 6.05 (s, 1H), 5.06 (s, 1H), 3.81 (dq, *J* = 10.7, 7.1 Hz, 1H), 3.68 (dq, *J* = 10.7, 7.1 Hz, 1H), 3.22 (ddd, *J* = 12.6, 7.4, 4.7 Hz, 1H), 2.41 – 2.27 (m, 2H), 1.89 (dp, *J* = 12.2, 8.3 Hz, 1H), 1.76 (dt, *J* = 18.0, 8.9 Hz, 1H), 1.37 – 1.25 (m, 1H), 1.33 (s, 18H), 0.43 (t, *J* = 7.1 Hz, 3H).

^{13}C **NMR** (125 MHz, $CDCl_3$) δ 214.6, 168.9, 152.6, 137.6, 135.6, 134.1, 132.1, 129.8, 128.5, 127.2, 126.2, 125.9, 125.3, 124.6, 124.4, 124.1, 66.9, 61.3, 50.9, 38.8, 34.3, 30.3, 29.7, 19.6, 13.1.

HRMS: (ESI+) [M+Na]+ calcd for $C_{33}H_{40}O_4Na$: 523.2819, found: 523.2819.

Specific rotation: $[\alpha]_D^{25}$ = -101.9° (c =1.0, CH_2Cl_2)

HPLC analysis of the product: Daicel Chiralpak AD-H column, hexane/*i*-PrOH = 98.5:1.5, 0.5 mL/min, λ = 210 nm, retention time: 15.24 min (major), 18.52 min (minor).

(*R*)-ethyl 1-((*S*)-(3,5-di-*tert*-butyl-4-hydroxyphenyl)(naphthalen-2-yl)methyl)-2-oxo-cyclopentanecarboxylate (4w) (pale yellow solid)

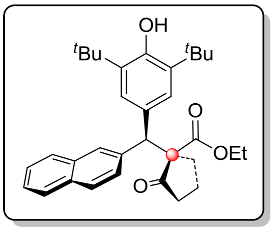

^{1}H **NMR** (500 MHz, $CDCl_3$) δ 7.86 – 7.78 (m, 2H), 7.78 – 7.73 (m, 2H), 7.46 (ddd, *J* = 6.9, 4.3, 1.8 Hz, 2H), 7.40 (dd, *J* = 8.5, 1.9 Hz, 1H), 6.94 (s, 2H), 5.35 (s, 1H), 5.10 (s, 1H), 4.00 – 3.78 (m, 2H), 3.16 (ddd, *J* = 12.6, 7.5, 4.4 Hz, 1H), 2.42 – 2.22 (m, 2H), 1.88 (dp, *J* = 12.6, 8.6 Hz, 1H), 1.72 (dt, *J* = 18.2, 9.1 Hz, 1H), 1.47 – 1.38 (m, 1H), 1.35 (s, 18H), 0.78 (t, *J* = 7.1 Hz, 3H).

^{13}C **NMR** (125 MHz, $CDCl_3$) δ 214.2, 169.1, 152.5, 139.3, 135.6, 133.3, 132.1, 130.5, 128.1, 127.9, 127.8, 127.5, 126.8, 126.7, 125.8, 125.5, 66.4, 61.6, 55.4, 38.7, 34.3, 30.3, 29.6, 19.6, 13.5.

HRMS: (ESI+) [M+Na]+ calcd for $C_{33}H_{40}O_4Na$: 523.2819, found: 523.2823.

Specific rotation: $[\alpha]_D^{25}$ = -108.2° (c = 0.8, CH_2Cl_2)

HPLC analysis of the product: Daicel Chiralpak IC-3 column, hexane/*i*-PrOH = 98.5:1.5, 0.8 mL/min, λ = 254 nm, retention time: 8.89 min (major), 10.93 min (minor).

(*R*)-ethyl 1-((*S*)-(3,5-di-*tert*-butyl-4-hydroxyphenyl)(6-methoxynaphthalen-2-yl)methyl) -2-oxocyclopentanecarboxylate (4x) (yellow solid)

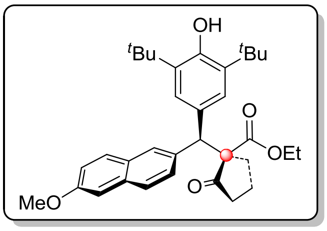

^{1}H **NMR** (500 MHz, $CDCl_3$) δ 7.70 (d, *J* = 8.9 Hz, 1H), 7.67 (d, *J* = 1.7 Hz, 1H), 7.66 (d, *J* = 8.6 Hz, 1H), 7.37 (dd, *J* = 8.5, 1.9 Hz, 1H), 7.14 (dd, *J* = 8.9, 2.5 Hz, 1H), 7.11 (d, *J* = 2.4 Hz, 1H), 6.95 (s, 2H), 5.32 (s, 1H), 5.10 (s, 1H), 3.93 (s, 3H), 3.97 – 3.87 (m, 1H), 3.90 – 3.80 (m, 1H), 3.18 – 3.10 (m, 1H), 2.35 – 2.25 (m, 2H), 1.93 – 1.81 (m, 1H), 1.72 (dt, *J* = 18.3, 9.1 Hz, 1H), 1.43 – 1.32 (m, 1H), 1.35 (s, 18H), 0.78 (t, *J* = 7.1 Hz, 3H).

^{13}C NMR (125 MHz, $CDCl_3$) δ 214.3, 169.1, 157.4, 152.5, 150.0, 136.9, 135.5, 133.1, 130.8, 129.3, 128.8, 128.5, 126.70, 126.66, 118.6, 105.6, 66.5, 61.5, 55.24, 55.22, 38.7, 34.3, 30.3, 29.5, 19.6, 13.5.

HRMS: (ESI^+) $[M+Na]^+$ calcd for $C_{34}H_{42}O_5Na$: 553.2924, found: 553.2928.

Specific rotation: $[\alpha]_D^{25}$ = –128.7° (c = 1.0, CH_2Cl_2).

HPLC analysis of the product: Daicel Chiralcel OD-H column, hexane/*i*-PrOH = 98.5:1.5, 0.7 mL/min, λ = 210 nm, retention time: 9.22 min (minor), 9.90 min (major).

(*R*)-ethyl 1-((*S*)-benzo[*b*]furan-2-yl(3,5-di-*tert*-butyl-4-hydroxyphenyl)methyl)-2-oxo-cyclopentanecarboxylate (4y) (colorless oil)

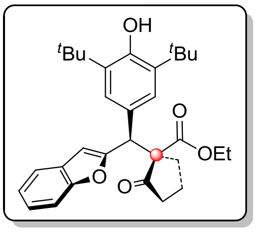

^{1}H NMR (500 MHz, $CDCl_3$) δ 7.52 (dd, *J* = 7.3, 1.5 Hz, 1H), 7.45 – 7.41 (m, 1H), 7.24 (td, *J* = 7.3, 1.6 Hz, 1H), 7.20 (td, *J* = 7.3, 1.6 Hz, 1H), 7.06 (s, 2H), 6.55 (s, 1H), 5.33 (s, 1H), 5.17 (s, 1H), 4.10 (dq, *J* = 10.8, 7.1 Hz, 1H), 4.03 (dq, *J* = 10.8, 7.1 Hz, 1H), 2.99 (dddd, *J* = 13.2, 7.5, 4.2, 1.2 Hz, 1H), 2.37 (ddd, *J* = 13.5, 9.1, 7.6 Hz, 1H), 2.30 – 2.21 (m, 1H), 1.97 – 1.83 (m, 1H), 1.71 – 1.59 (m, 2H), 1.47 – 1.36 (m, 1H), 1.39 (s, 18H), 1.03 (t, *J* = 7.1 Hz, 3H).

^{13}C NMR (125 MHz, $CDCl_3$) δ 213.0, 168.9, 158.0, 154.5, 153.1, 135.8, 128.4, 127.4, 126.6, 123.6, 122.5, 120.6, 110.9, 104.2, 65.3, 61.8, 49.0, 38.7, 34.3, 30.3, 28.8, 19.6, 13.8.

HRMS: (ESI^+) $[M+Na]^+$ calcd for $C_{31}H_{38}O_5Na$: 513.2611, found: 513.2619.

Specific rotation: $[\alpha]_D^{25}$ = -86.5° (c = 1.0, CH_2Cl_2)

HPLC analysis of the product: Daicel Chiralpak IC-3 column, hexane/*i*-PrOH = 95.0:5.0, 0.8 mL/min, λ = 210 nm, retention time: 5.53 min (minor), 5.80 min (major).

(*R*)-ethyl 1-((*S*)-benzo[*b*]thiophen-2-yl(3,5-di-*tert*-butyl-4-hydroxyphenyl)methyl)-2-oxocyclopentanecarboxylate (4z) (yellow solid)

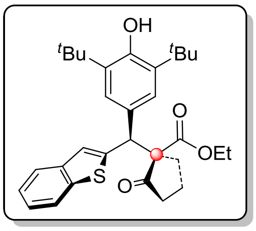

^{1}H NMR (500 MHz, $CDCl_3$) δ 7.76 (d, *J* = 7.9 Hz, 1H), 7.71 (dd, *J* = 7.6, 1.3 Hz, 1H), 7.33 (td, *J* = 7.5, 1.3 Hz, 1H), 7.30 – 7.25 (m, 1H), 7.17 (s, 1H), 7.08 (s, 2H), 5.46 (s, 1H), 5.16 (s, 1H), 4.07 (dq, *J* = 10.8, 7.1 Hz, 1H), 4.00 (dq, *J* = 10.8, 7.1 Hz, 1H), 3.04 (ddd, *J* = 12.7, 7.7, 4.4 Hz, 1H), 2.37 (ddd, *J* = 13.3, 8.9, 7.7 Hz, 1H), 2.26 (ddd, *J* = 18.1, 8.6, 4.5 Hz, 1H), 1.89 (dp, *J* = 12.5, 8.8 Hz, 1H), 1.66 (dt, *J* = 18.4, 9.2 Hz, 1H), 1.47 – 1.36 (m, 1H), 1.39 (s, 18H), 0.99 (t, *J* = 7.1 Hz, 3H).

^{13}C NMR (125 MHz, $CDCl_3$) δ 213.1, 168.9, 152.9, 145.6, 139.6, 139.5, 135.6, 129.0, 126.6, 124.0, 123.8, 123.1, 122.2, 121.9, 66.7, 61.8, 51.0, 38.7, 34.4, 30.3, 28.9, 19.5, 13.7.

HRMS: (ESI^+) $[M+Na]^+$ calcd for $C_{31}H_{38}O_4SNa$: 529.2383, found: 529.2386.

Specific rotation: $[\alpha]_D^{25}$ = -82.2° (c = 1.0, CH_2Cl_2)

HPLC analysis of the product: Daicel Chiralpak IA-3 column, hexane/*i*-PrOH = 99.0:1.0, 0.55 mL/min, λ = 254 nm, retention time: 14.19 min (major), 14.50 min (minor).

(*R*)-ethyl 1-((*R*)-(3,5-di-*tert*-butyl-4-hydroxyphenyl)(1-methyl-1H-indol-3-yl)methyl)-2-oxocyclopentanecarboxylate (4aa) (colorless oil)

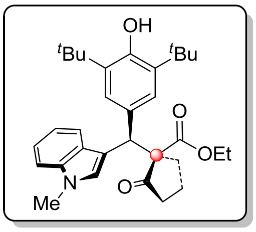

^{1}H NMR (500 MHz, $CDCl_3$) δ 7.48 (dt, *J* = 7.9, 0.9 Hz, 1H), 7.25 (t, *J* = 9.0 Hz, 1H), 7.18 (ddd, *J* = 8.1, 6.9, 1.1 Hz, 1H), 7.08 (s, 2H), 7.06 – 7.02 (m, 1H), 7.01 (s, 1H), 5.42 (s, 1H), 5.05 (s, 1H), 3.91 (dq, *J* = 10.7, 7.1 Hz, 1H), 3.86 (dq, *J* = 10.7, 7.1 Hz, 1H), 3.79 (s, 3H), 2.96 (dddd, *J* = 12.9, 7.3, 4.3, 1.4 Hz, 1H), 2.36 – 2.29 (m, 1H), 2.27 – 2.19 (m,

1H), 1.85 – 1.75 (m, 1H), 1.71 – 1.62 (m, 1H), 1.38 – 1.28 (m, 1H), 1.37 (s, 18H), 0.75 (t, *J* = 7.1 Hz, 3H).

^{13}C NMR (125 MHz, $CDCl_3$) δ 214.1, 169.6, 152.4, 136.6, 135.2, 130.1, 128.2, 126.5, 125.5, 121.5, 119.8, 118.6, 114.6, 108.6, 67.0, 61.4, 46.5, 39.1, 34.3, 32.8, 30.4, 29.1, 19.4, 13.4.

HRMS: (ESI$^+$) [M+Na]$^+$ calcd for $C_{32}H_{41}NO_4Na$: 526.2928, found: 526.2930.

Specific rotation: $[\alpha]_D^{25}$ = -50.9° (c = 0.8, CH_2Cl_2)

HPLC analysis of the product: Daicel Chiralpak IC-3 column, hexane/*i*-PrOH = 90.0:10.0, 0.8 mL/min, λ = 280 nm, retention time: 5.71 min (major), 6.03 min (minor).

(*R*)-ethyl 1-((*S*)-(3,5-di-*tert*-butyl-4-hydroxyphenyl)(thiophen-2-yl)methyl)-2-oxocyclopentanecarboxylate (4ab) (yellow solid)

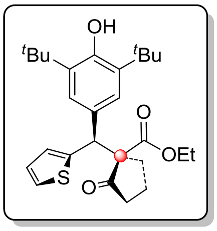

^{1}H NMR (500 MHz, $CDCl_3$) δ 7.20 – 7.14 (m, 1H), 7.04 (s, 2H), 7.00 – 6.89 (m, 2H), 5.42 (s, 1H), 5.12 (s, 1H), 4.08 (dq, *J* = 10.8, 7.1 Hz, 1H), 3.98 (dq, *J* = 10.8, 7.1 Hz, 1H), 2.94 (ddt, *J* = 12.7, 7.9, 4.0 Hz, 1H), 2.33 – 2.20 (m, 2H), 1.92 – 1.78 (m, 1H), 1.71 – 1.58 (m, 1H), 1.47 – 1.38 (m, 1H), 1.39 (s, 18H), 1.03 (t, *J* = 7.1 Hz, 3H).

^{13}C NMR (125 MHz, $CDCl_3$) δ 213.3, 168.8, 152.7, 144.1, 135.5, 129.7, 126.5, 126.2, 125.8, 123.9, 67.1, 61.7, 50.3, 38.8, 34.4, 30.3, 28.8, 19.5, 13.6.

HRMS: (ESI$^+$) [M+Na]$^+$ calcd for $C_{27}H_{36}O_4SNa$: 479.2227, found: 479.2231.

Specific rotation: $[\alpha]_D^{25}$ = -50.1° (c = 1.0, CH_2Cl_2)

HPLC analysis of the product: Daicel Chiralpak IA-3 column, hexane/*i*-PrOH = 99.0:1.0, 0.5 mL/min, λ = 280 nm, retention time: 12.50 min (major), 15.51 min (minor).

(*R*)-ethyl 1-((*S*)-1-(3-(*tert*-butyl)-4-hydroxy-5-methylphenyl)ethyl)-2-oxocyclopentanecarboxylate (4ac) (yellow oil)

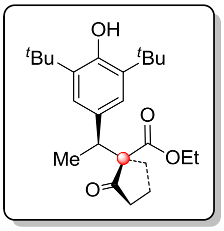

^{1}H NMR (500 MHz, $CDCl_3$) δ^{1}H NMR (500 MHz, Chloroform-*d*) δ 7.02 (s, 2H), 5.13 (s, 1H), 4.34 – 4.15 (m, 2H), 3.83 (q, *J* = 7.1 Hz, 1H), 2.62 – 2.47 (m, 1H), 2.20 – 2.04 (m, 2H), 1.88 – 1.68 (m, 2H), 1.57 – 1.48 (m, 1H), 1.44 (s, 18H), 1.30 (d, *J* = 7.1 Hz, 3H), 1.26 (t, *J* = 7.1 Hz, 3H) (diastereoisomer **A**).

δ 6.99 (s, 2H), 5.09 (s, 1H), 4.09 – 3.95 (m, 2H), 3.65 (q, *J* = 7.1 Hz, 1H), 2.46 – 2.36 (m, 1H), 2.01 – 1.93 (m, 1H), 1.79 – 1.68 (m, 2H), 1.55 – 1.45 (m, 1H), 1.43 (s, 18H), 1.39 – 1.32 (m, 1H), 1.30 (d, *J* = 7.1 Hz, 3H), 1.12 (t, *J* = 7.1 Hz, 3H) (diastereoisomer **B**).

^{13}C NMR (125 MHz, $CDCl_3$) δ 214.4, 170.0, 152.53, 135.3, 131.3, 125.7, 66.42, 61.6, 42.8, 39.0, 34.34, 30.4, 27.3, 19.3, 16.3, 14.1 (diastereoisomer **A**).

δ 214.6, 169.7, 152.47, 135.4, 132.7, 124.7, 66.35, 61.3, 43.8, 39.2, 34.30, 30.3, 29.4, 19.5, 17.7, 13.9 (diastereoisomer **B**).

HRMS: (ESI$^+$) [M+Na]$^+$ calcd for $C_{21}H_{30}O_4Na$: 369.2036, found: 369.2040.

Specific rotation: $[\alpha]_D^{25}$ = -15.3° (c = 1.2, CH_2Cl_2)

HPLC analysis of the product: Daicel Chiralpak IC-3 column, hexane/*i*-PrOH = 98.5:1.5, 0.8 mL/min, λ = 280 nm, retention time: 11.93 min (major), 12.44 min (minor).

(*R*)-ethyl 1-((*S*)-(4-hydroxy-3,5-dimethylphenyl)(4-methoxyphenyl)methyl)-2-oxocyclopentanecarboxylate (6a) (pale yellow oil)

^{1}H NMR (500 MHz, $CDCl_3$) δ 7.18 (d, *J* = 8.7 Hz, 2H), 6.87 (s, 2H), 6.82 (d, *J* = 8.7 Hz, 2H), 5.08 (s, 1H), 4.61 (s, 1H), 4.06 – 3.87 (m, 2H), 3.79 (s, 3H), 3.12 – 2.96 (m, 1H), 2.35 – 2.23 (m,

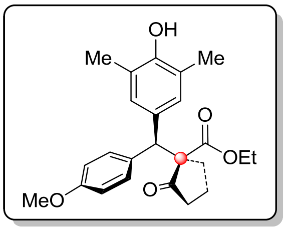

2H), 2.21 (s, 6H), 1.95 – 1.80 (m, 1H), 1.74 (dq, *J* = 19.1, 9.5 Hz, 1H), 1.56 (dtt, *J* = 20.8, 8.1, 4.3 Hz, 1H), 0.92 (t, *J* = 7.1 Hz, 3H) (diastereoisomer **A**).

δ 7.04 (d, *J* = 8.7 Hz, 2H), 6.76 (d, *J* = 8.7 Hz, 2H), 6.69 (s, 2H), 5.07 (s, 1H), 4.60 (s, 1H), 4.06 – 3.85 (m, 2H), 3.76 (s, 3H), 3.10 – 2.92 (m, 1H), 2.34 – 2.23 (m, 2H), 2.16 (s, 6H), 1.97 – 1.80 (m, 1H), 1.74 (dq, *J* = 19.1, 9.5 Hz, 1H), 1.57 (dtd, *J* = 18.1, 8.1, 3.9 Hz, 1H), 0.91 (t, *J* = 7.1 Hz, 3H) (diastereoisomer **B**).

^{13}C NMR (125 MHz, $CDCl_3$) δ 214.2, 168.79, 158.2, 150.9, 133.8, 133.0, 131.1, 129.8, 122.6, 113.6, 66.4, 61.56, 55.2, 53.7, 38.6, 29.30, 19.80, 15.98, 13.6 (diastereoisomer **A**).

δ 214.3, 168.84, 157.9, 150.6, 133.0, 132.2, 130.2, 129.1, 122.7, 113.5, 66.5, 61.52, 55.1, 53.6, 38.6, 29.34, 19.78, 16.01, 13.6 (diastereoisomer **B**).

HRMS: (ESI$^+$) $[M+Na]^+$ calcd for $C_{24}H_{28}O_5Na$: 419.1829, found: 419.1834.

Specific rotation: $[\alpha]_D^{25}$ = –86.2° (c = 0.9, CH_2Cl_2).

HPLC analysis of the product: Daicel Chiralpak IA-3 column, hexane/*i*-PrOH = 95.0:5.0, 0.5 mL/min, λ = 210 nm, retention time: 33.00 min (major), 44.60 min (minor).

(*R*)-ethyl 1-((*S*)-(3-(*tert*-butyl)-4-hydroxy-5-methylphenyl)(4-methoxyphenyl)methyl)-2-oxocyclopentanecarboxylate (6b) (yellow oil)

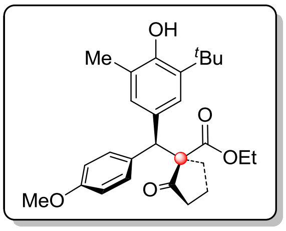

^{1}H NMR (500 MHz, $CDCl_3$) δ 7.20 (d, *J* = 8.7 Hz, 2H), 6.93 (d, *J* = 2.2 Hz, 1H), 6.82 (d, *J* = 8.7 Hz, 2H), 6.65 (d, *J* = 2.2 Hz, 1H), 5.11 (s, 1H), 4.69 (s, 1H), 3.99 (dq, *J* = 10.7, 7.1 Hz, 1H), 3.90 (dq, *J* = 10.7, 7.1 Hz, 1H), 3.80 (s, 3H), 3.04 – 2.96 (m, 1H), 2.30 – 2.21 (m, 2H), 2.15 (s, 3H), 1.90 – 1.81 (m, 1H), 1.78 – 1.67 (m, 1H), 1.50 (qd, *J* = 8.5, 4.1 Hz, 1H), 1.35 (s, 9H), 0.91 (t, *J* = 7.1 Hz, 3H).

^{13}C NMR (125 MHz, $CDCl_3$) δ 214.3, 168.9, 157.9, 151.3, 135.2, 133.7, 131.7, 131.0, 129.9, 126.8, 122.7, 113.6, 66.5, 61.6, 55.2, 54.1, 38.7, 34.5, 29.7, 29.3, 19.7, 16.1, 13.6.

HRMS: (ESI$^+$) $[M+Na]^+$ calcd for $C_{27}H_{34}O_5Na$: 461.2298, found: 461.2303.

Specific rotation: $[\alpha]_D^{25}$ = –93.0° (c = 0.8, CH_2Cl_2).

HPLC analysis of the product: Daicel Chiralpak IC-3 column, hexane/*i*-PrOH = 97.5:2.5, 0.8 mL/min, λ = 280 nm, retention time: 14.88 min (major), 17.35 min (minor).

(*R*)-ethyl 1-((*S*)-(4-hydroxy-3,5-diisopropylphenyl)(4-methoxyphenyl)methyl)-2-oxo-cyclopentanecarboxylate (6c) (pale yellow oil)

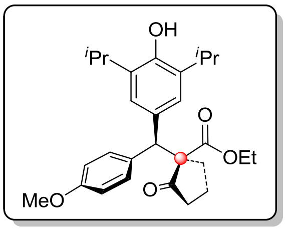

^{1}H NMR (500 MHz, $CDCl_3$) δ 7.20 (d, *J* = 8.3 Hz, 2H), 6.83 (d, *J* = 8.3 Hz, 2H), 6.77 (s, 2H), 5.15 (s, 1H), 4.73 (s, 1H), 3.99 (dq, *J* = 10.6, 7.1 Hz, 1H), 3.91 (dq, *J* = 10.6, 7.1 Hz, 1H), 3.80 (s, 3H), 3.07 (hept, *J* = 6.8 Hz, 2H), 3.01 (ddd, *J* = 12.1, 7.2, 4.0 Hz, 1H), 2.24 (ddt, *J* = 26.3, 16.6, 6.3 Hz, 2H), 1.83 (dq, *J* = 21.0, 8.8 Hz, 1H), 1.76 – 1.66 (m, 1H), 1.42 (ddt, *J* = 12.4, 8.2, 4.1 Hz, 1H), 1.22 (d, *J* = 6.8 Hz, 6H), 1.14 (d, *J* = 6.8 Hz, 6H), 0.92 (t, *J* = 7.1 Hz, 3H).

^{13}C NMR (125 MHz, $CDCl_3$) δ 214.4, 169.0, 157.9, 148.5, 133.6, 133.3, 132.4, 129.9, 125.1, 113.5, 66.5, 61.6, 55.2, 54.3, 38.7, 29.3, 27.1, 22.7, 22.7, 19.6, 13.6.

HRMS: (ESI$^+$) $[M+Na]^+$ calcd for $C_{28}H_{36}O_5Na$: 475.2455, found: 475.2458.

Specific rotation: $[\alpha]_D^{25}$ = –79.1° (c = 0.9, CH_2Cl_2).

HPLC analysis of the product: Daicel Chiralpak AD-H column, hexane/*i*-PrOH = 95.0:5.0,

0.5 mL/min, λ = 280 nm, retention time: 21.11 min (major), 22.37 min (minor).

(*R*)-ethyl 1-((*S*)-(4-hydroxy-3,5-bis(trimethylsilyl)phenyl)(4-methoxyphenyl)methyl)-2-oxocyclopentanecarboxylate (6d) (yellow oil)

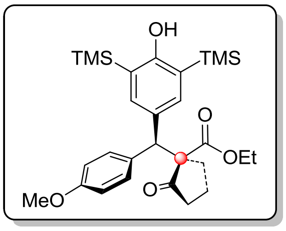

^{1}H NMR (500 MHz, $CDCl_3$) δ 7.21 (d, *J* = 8.7 Hz, 2H), 7.09 (s, 2H), 6.83 (d, *J* = 8.7 Hz, 2H), 5.13 (s, 1H), 4.94 (s, 1H), 3.99 (dq, *J* = 10.7, 7.1 Hz, 1H), 3.91 (dq, *J* = 10.7, 7.1 Hz, 1H), 3.80 (s, 3H), 3.10 – 2.87 (m, 1H), 2.35 – 2.24 (m, 1H), 2.23 – 2.11 (m, 1H), 1.86 (dtt, *J* = 12.2, 9.9, 7.7 Hz, 1H), 1.72 (ddd, *J* = 18.8, 10.2, 8.9 Hz, 1H), 1.54 (qd, *J* = 8.6, 3.8 Hz, 1H), 0.91 (t, *J* = 7.1 Hz, 3H), 0.28 (s, 18H).

^{13}C NMR (125 MHz, $CDCl_3$) δ 214.0, 168.8, 163.9, 158.0, 138.1, 133.4, 131.9, 130.0, 123.6, 113.5, 66.6, 61.6, 55.2, 53.9, 38.6, 29.3, 19.7, 13.6, -0.6.

HRMS: (ESI^+) $[M+Na]^+$ calcd for $C_{28}H_{40}O_5Si_2Na$: 535.2306, found: 535.2306.

Specific rotation: $[\alpha]_D^{25}$ = –82.8° (c = 1.2, CH_2Cl_2).

HPLC analysis of the product: Daicel Chiralpak IC-3 column, hexane/*i*-PrOH = 99.2:0.8, 0.5 mL/min, λ = 210 nm, retention time: 17.27 min (major).

(*R*)-ethyl 1-((*S*)-(2'-hydroxy-[1,1':3',1''-terphenyl]-5'-yl)(4-methoxyphenyl)methyl)-2-oxocyclopentanecarboxylate (6e) (yellow oil)

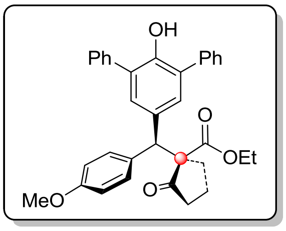

^{1}H NMR (500 MHz, $CDCl_3$) δ 7.58 – 7.33 (m, 10H), 7.23 (d, *J* = 8.7 Hz, 2H), 7.06 (s, 2H), 6.82 (d, *J* = 8.7 Hz, 2H), 5.39 (s, 1H), 5.23 (s, 1H), 4.05 – 3.96 (m, 1H), 3.96 – 3.90 (m, 1H), 3.79 (s, 3H), 3.08 (ddd, *J* = 12.1, 5.2, 2.5 Hz, 1H), 2.44 – 2.30 (m, 2H), 2.03 – 1.83 (m, 2H), 1.74 – 1.66 (m, 1H), 0.93 (t, *J* = 7.1 Hz, 3H) (diastereoisomer **A**).

δ^{1}H NMR (500 MHz, Chloroform-*d*) δ 7.57 – 7.36 (m, 10H), 7.10 (d, *J* = 8.7 Hz, 2H), 6.95 (s, 2H), 6.78 (d, *J* = 8.7 Hz, 2H), 5.32 (s, 1H), 5.24 (s, 1H), 4.18 – 4.11 (m, 1H), 4.07 – 3.97 (m, 1H), 3.82 (s, 3H), 3.10 – 3.01 (m, 1H), 2.45 – 2.22 (m, 2H), 2.00 – 1.83 (m, 2H), 1.69 (dq, *J* = 10.9, 3.8, 3.2 Hz, 1H), 0.95 (t, *J* = 7.1 Hz, 3H) (diastereoisomer **B**).

^{13}C NMR (125 MHz, $CDCl_3$) δ 214.0, 213.9, 170.0, 168.83, 168.79, 168.7, 158.3, 158.1, 148.1, 147.8, 137.64, 137.56, 137.3, 135.8, 133.3, 132.8, 131.5, 131.1, 130.4, 129.9, 129.25, 129.24, 129.0, 128.9, 128.8, 128.7, 128.6, 128.5, 128.4, 128.3, 127.64, 127.57, 66.6, 66.5, 61.7, 60.4, 55.3, 55.2, 53.8, 53.7, 38.6, 38.5, 29.5, 29.3, 21.3, 19.8, 14.2, 13.6 (diastereoisomer **A** and **B**).

HRMS: (ESI^+) $[M+Na]^+$ calcd for $C_{34}H_{32}O_5Na$: 543.2142, found: 543.2149.

Specific rotation: $[\alpha]_D^{25}$ = –71.5° (c = 1.5, CH_2Cl_2).

HPLC analysis of the product: Daicel Chiralcel OD-H column, hexane/*i*-PrOH = 96.0:4.0, 0.7 mL/min, λ = 254 nm, retention time: 28.76 min (minor), 33.55 min (major).

V. Gram-Scale Synthesis

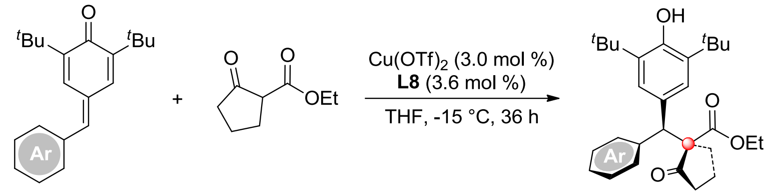

An oven-dried Schlenk flask was charged with $Cu(OTf)_2$ (32.5 mg, 0.09 mmol, 3 mol%), **L8** (79.5 mg, 0.108 mmol, 3.6 mol%), *p*-quinone methide substrates (3 mmol) and a stir bar. The flask was degased and refilled with nitrogen for 3 times, and then anhydrous THF (25 mL) was injected to the tube via a syringe. The reaction mixture was cooled to -15 °C. After stirring for 3 minutes, a solution of ethyl 2-oxocyclopentanecarboxylate (702 mg, 4.5 mmol, 1.5 equiv) in anhydrous THF (15 mL) was injected to the reaction mixture via a syringe. After stirring at the same temperature for additional 36 hours, the reaction was quenched with saturated NH_4Cl and extracted with ethyl acetate (3 × 100 mL). The combined organic layers were dried with Na_2SO_4, filtered, and concentrated under reduced pressure. The residue was purified by silica gel column chromatography (petroleum ether/ethyl acetate) to afford the pure product.

VI. Synthesis of Compounds 7 and 8

6.1 Synthesis of Compound 7

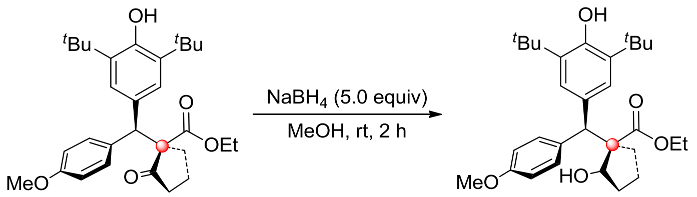

3a **S22**, 94% yield

To a solution of compound **3a** (240 mg, 0.5 mmol) in methanol (50 mL) was added $NaBH_4$ (95 mg, 2.5 mmol) in portions. The reaction mixture was then stirred at rt for 2 hours. After completion, the reaction mixture was quenched quenched with saturated NH_4Cl solution and extracted with ethyl acetate (3 × 50 mL). The combined organic solvents were concentrated under reduced pressure. The residue was purified by silica gel column chromatography (petroleum ether/ethyl acetate = 6/1) to afford a colorless oil **S22**.

(1*R*,2*S*)-ethyl 1-((*S*)-(3,5-di-*tert*-butyl-4-hydroxyphenyl)(4-methoxyphenyl)methyl)-2-hydroxycyclopentanecarboxylate (S22)

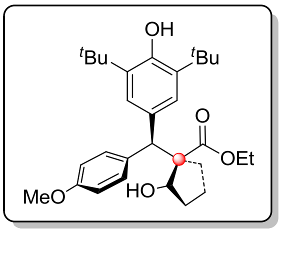

^{1}H NMR (500 MHz, $CDCl_3$) δ 7.50 (d, *J* = 8.7 Hz, 2H), 7.11 (s, 2H), 6.86 (d, *J* = 8.7 Hz, 2H), 5.05 (s, 1H), 4.51 (s, 1H), 4.44 (s, 1H), 3.90 (dq, *J* = 10.4, 7.2 Hz, 1H), 3.80 (s, 3H), 3.80 – 3.69 (m, 1H), 2.46 (ddd, *J* = 11.7, 8.6, 2.7 Hz, 1H), 2.11 – 1.95 (m, 1H), 1.89 – 1.69 (m,

3H), 1.60 – 1.51 (m, 2H), 1.41 (s, 18H), 0.93 (t, *J* = 7.1 Hz, 3H).

^{13}C NMR (125 MHz, $CDCl_3$) δ 175.2, 158.1, 152.1, 135.0, 134.6, 132.4, 130.5, 126.0, 113.7, 77.5, 64.1, 60.4, 55.2, 54.3, 34.3, 32.6, 32.0, 30.3, 20.8, 13.6.

HRMS: (ESI$^+$) $[M+Na]^+$ calcd for $C_{30}H_{42}O_5Na$: 505.2924, found: 505.2931.

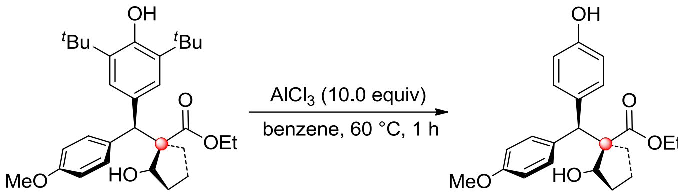

S22 **7**, 85% yield, 97% ee

An oven-dried Schlenk tube was charged with $AlCl_3$ (133.3 mg, 1.0 mmol, 10 eq.) and a stir bar. The tube was degased and refilled with nitrogen for 3 times, and then a solution of **S12** (48.2 mg, 0.1 mmol) in anhydrous benzene (5.0 mL) was injected to the tube via a syringe. The mixture was heated at 60 °C for 1 hours. The reaction was quenched with water and extracted with ethyl acetate (3 × 50 mL). The combined organic layers were dried with Na_2SO_4, filtered, and concentrated under reduced pressure. The residue was purified by silica gel column chromatography (petroleum ether/ethyl acetate = 2/1) to afford the pure product **7** as a colorless solid.

(1*R*,2*S*)-ethyl 2-hydroxy-1-((*R*)-(4-hydroxyphenyl)(4-methoxyphenyl)methyl)cyclopentanecarboxylate (7)

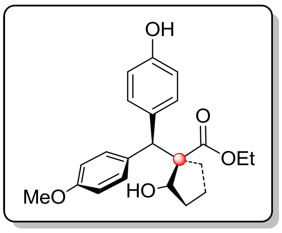

^{1}H NMR (500 MHz, $CDCl_3$) δ 7.41 (d, *J* = 8.7 Hz, 2H), 7.21 (d, *J* = 8.5 Hz, 2H), 6.83 (d, *J* = 8.7 Hz, 2H), 6.71 (d, *J* = 8.5 Hz, 2H), 5.37 (s, 1H), 4.55 (s, 1H), 4.52 (s, 1H), 3.92 (dddd, *J* = 17.8, 10.8, 7.2, 3.6 Hz, 2H), 3.79 (s, 3H), 2.44 (ddd, *J* = 11.7, 8.4, 2.9 Hz, 1H), 2.01 (dt, *J* = 13.0, 9.1 Hz, 1H), 1.89 – 1.81 (m, 1H), 1.78 – 1.65 (m, 2H), 1.59 (ddd, *J* = 13.7, 5.2, 3.1 Hz, 1H), 1.55 – 1.49 (m, 1H), 1.00 (t, *J* = 7.1 Hz, 3H).

^{13}C NMR (125 MHz, $CDCl_3$) δ 175.4, 158.1, 154.2, 134.6, 134.3, 130.9, 130.2, 114.8, 113.7, 77.4, 63.4, 60.6, 55.2, 52.9, 32.4, 32.3, 20.6, 13.7.

HRMS: (ESI$^+$) $[M+Na]^+$ calcd for $C_{22}H_{26}O_5Na$: 393.1672, found: 393.1676.

Specific rotation: $[\alpha]_D^{25}$ = -33.0° (c = 1.0, CH_2Cl_2)

HPLC analysis of the product: Daicel Chiralpak IC-3 column, hexane/*i*-PrOH = 92.0:8.0, 0.8 mL/min, λ = 210 nm, retention time: 21.73 min (minor), 22.85 min (major).

6.2 Synthesis of Compound 8

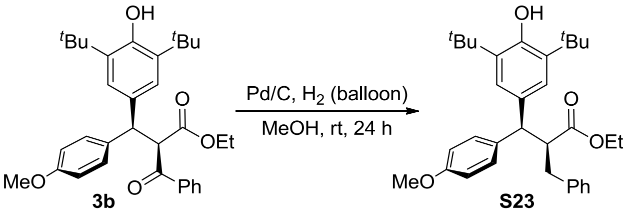

3b **S23**

An oven-dried vial was charged with compound **3b** (256 mg, 0.5 mmol), 5% Pd/C (250 mg), methanol (50 mL) and a stir bar. The reaction mixture was degased and refilled with

H_2 for 3 times, and then stirred at rt for 24 hours under H_2 atmosphere (balloon). After completion, the reaction mixture was filtered through a Celite pad and the filtride was washed with ethyl acetate for 3 times (3 × 50 mL). The combined organic solvents were concentrated under reduced pressure. The residue was purified by silica gel column chromatography (petroleum ether/ethyl acetate) to afford the pure product **S23**.

(2*S*,3*S*)-ethyl 2-benzyl-3-(3,5-di-*tert*-butyl-4-hydroxyphenyl)-3-(4-methoxyphenyl) propanoate (S23)

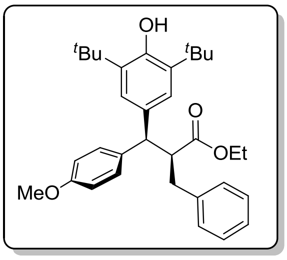

^{1}H NMR (500 MHz, $CDCl_3$) δ 7.31 – 7.22 (m, 4H), 7.21 – 7.15 (m, 3H), 7.12 – 7.06 (m, 2H), 6.80 (d, *J* = 8.7 Hz, 2H), 5.10 (s, 1H), 4.05 (d, *J* = 11.7 Hz, 1H), 3.79 – 3.70 (m, 2H), 3.76 (s, 3H), 3.44 (ddd, *J* = 11.7, 8.9, 5.7 Hz, 1H), 2.88 – 2.71 (m, 2H), 1.47 (s, 18H), 0.82 (t, *J* = 7.1 Hz, 3H).

^{13}C NMR (125 MHz, $CDCl_3$) δ 174.6, 158.1, 152.4, 139.7, 136.1, 135.6, 132.9, 128.8, 128.6, 128.3, 126.2, 124.3, 113.7, 59.9, 55.2, 54.3, 54.2, 38.1, 34.4, 30.4, 13.8.

HRMS: (ESI^+) $[M+Na]^+$ calcd for $C_{33}H_{42}O_4Na$: 525.2975, found: 525.2974.

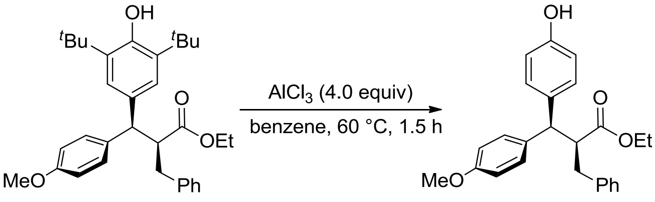

An oven-dried Schlenk tube was charged with $AlCl_3$ (26.7 mg, 0.20 mmol, 4 eq.) and a stir bar. The tube was degased and refilled with nitrogen for 3 times, and then a solution of **S23** (25.0 mg, 0.05 mmol) in anhydrous benzene (2.0 mL) was injected to the tube via a syringe. The mixture was heated at 60 °C for 1.5 hours. The reaction was quenched with water and extracted with ethyl acetate (3 × 20 mL). The combined organic layers were dried with Na_2SO_4, filtered, and concentrated under reduced pressure. The residue was purified by silica gel column chromatography (petroleum ether/ethyl acetate) to afford the product **8** as a colorless oil.

(2*S*,3*R*)-ethyl 2-benzyl-3-(4-hydroxyphenyl)-3-(4-methoxyphenyl)propanoate (8)

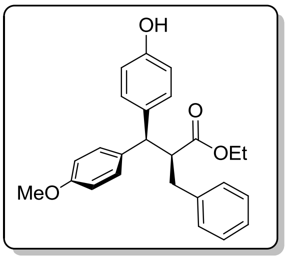

^{1}H NMR (500 MHz, $CDCl_3$) δ 7.30 – 7.22 (m, 6H), 7.21 – 7.16 (m, 1H), 7.14 – 7.07 (m, 2H), 6.82 (d, *J* = 8.5 Hz, 2H), 6.78 (d, *J* = 8.7 Hz, 2H), 5.00 (s, 1H), 4.11 (d, *J* = 11.6 Hz, 1H), 3.75 (s, 3H), 3.74 (q, *J* = 7.1 Hz, 2H), 3.44 (ddd, *J* = 11.6, 9.7, 5.3 Hz, 1H), 2.87 – 2.73 (m, 2H), 0.81 (t, *J* = 7.1 Hz, 3H).

^{13}C NMR (125 MHz, $CDCl_3$) δ 174.4, 158.2, 154.3, 139.3, 135.1, 134.9, 129.1, 128.7, 128.7, 128.3, 126.3, 115.7, 113.8, 60.00, 55.2, 53.6, 53.3, 38.1, 13.8.

HRMS: (ESI^+) $[M+Na]^+$ calcd for $C_{25}H_{26}O_4Na$: 413.1723, found: 413.1730.

Specific rotation: $[\alpha]_D^{25}$ = -8.8° (c = 0.5, CH_2Cl_2)

HPLC analysis of the product: Daicel Chiralpak AD-H column, hexane/*i*-PrOH = 80.0:20.0, 0.5 mL/min, λ = 210 nm, retention time: 34.23 min (major).

VII. X-Ray Crystallographic Analysis

7.1 Relative Configuration of 3g

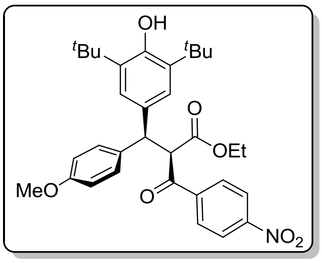

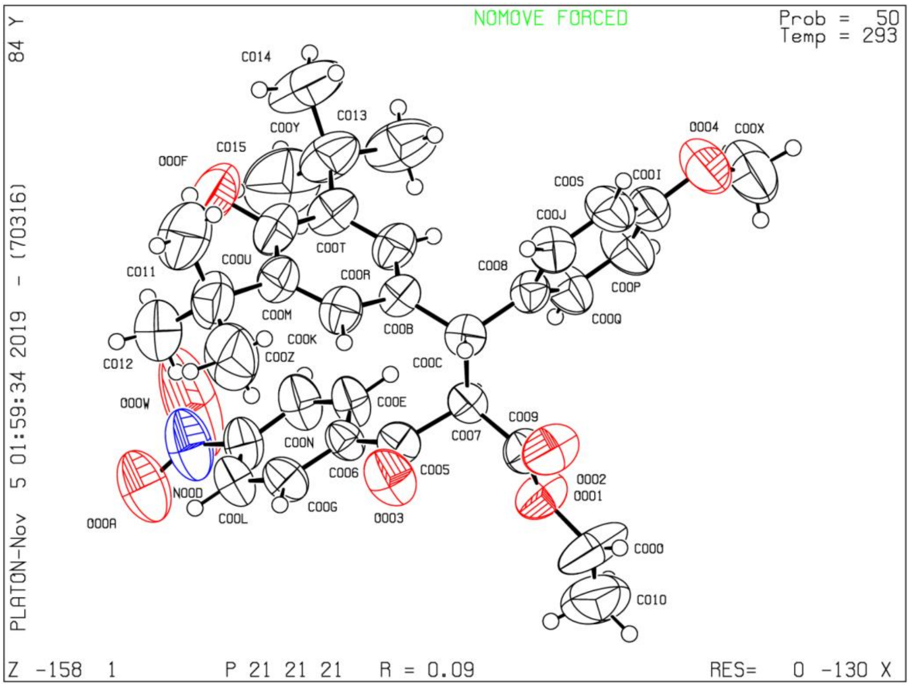

Figure S1. ORTEP Drawing of **3g** (CCDC1963665)

Empirical formula	C33 H37 N O7	
Formula weight	559.63	
Temperature	293(2) K	
Wavelength	0.71073 Å	
Crystal system	Orthorhombic	
Space group	$P2_12_12_1$	
Unit cell dimensions	a = 10.4833(19) Å	a= 90°.
	b = 14.435(2) Å	b= 90°.
	c = 21.245(4) Å	g = 90°.
Volume	3214.9(9) Å^3	
Z	4	

Density (calculated)	1.156 Mg/m^3
Absorption coefficient	0.081 mm^{-1}
F(000)	1192
Crystal size	? x ? x ? mm^3
Theta range for data collection	3.413 to 29.351°.
Index ranges	-13<=h<=14, -19<=k<=19, -27<=l<=28
Reflections collected	17567
Independent reflections	7044 [R(int) = 0.0674]
Completeness to theta = 25.242°	99.7 %
Absorption correction	None
Refinement method	Full-matrix least-squares on F^2
Data / restraints / parameters	7044 / 0 / 384
Goodness-of-fit on F^2	1.032
Final R indices [I>2sigma(I)]	R1 = 0.0864, wR2 = 0.1593
R indices (all data)	R1 = 0.2473, wR2 = 0.2271
Absolute structure parameter	-1.4(10)
Extinction coefficient	n/a
Largest diff. peak and hole	0.258 and -0.205 e.Å^{-3}

7.2 Relative Configuration of 3v

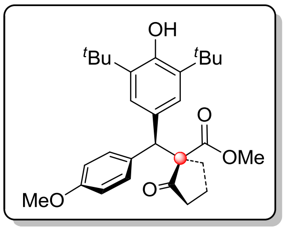

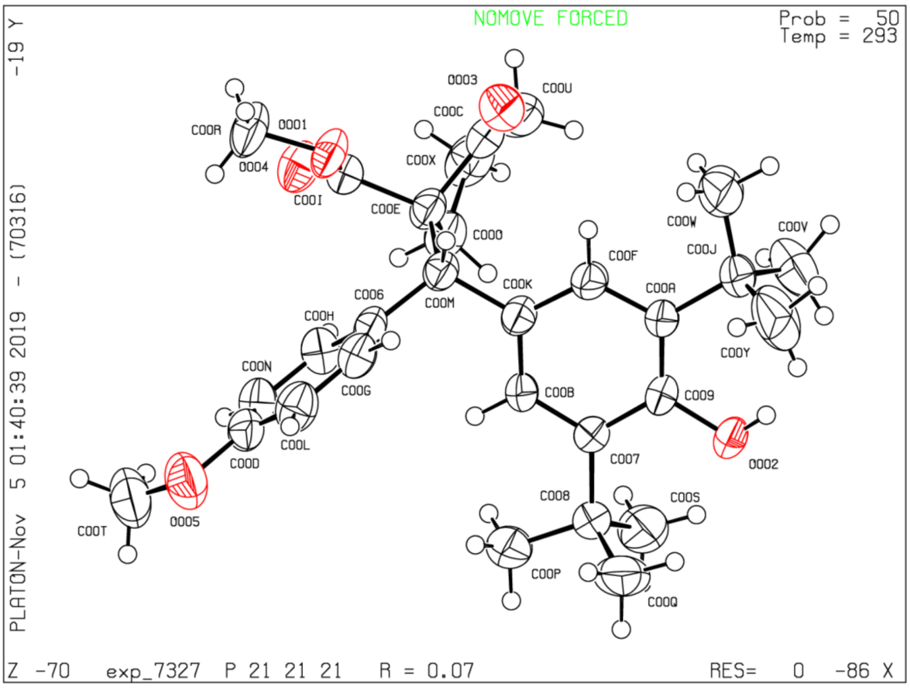

Figure S2. ORTEP Drawing of **3v** (CCDC1958259)

Identification code	exp_7327	
Empirical formula	C29 H38 O5	
Formula weight	466.59	
Temperature	293(2) K	
Wavelength	0.71073 Å	
Crystal system	Orthorhombic	
Space group	$P2_12_12_1$	
Unit cell dimensions	a = 10.0470(9) Å	a= 90°.
	b = 11.0487(7) Å	b= 90°.
	c = 24.2420(14) Å	g = 90°.
Volume	2691.0(3) Å^3	
Z	4	
Density (calculated)	1.152 Mg/m^3	
Absorption coefficient	0.077 mm^{-1}	
F(000)	1008	

Crystal size	? x ? x ? mm^3
Theta range for data collection	3.362 to 29.433°.
Index ranges	-12<=h<=13, -13<=k<=14, -32<=l<=32
Reflections collected	21343
Independent reflections	6455 [R(int) = 0.0649]
Completeness to theta = 25.242°	99.6 %
Absorption correction	Semi-empirical from equivalents
Max. and min. transmission	1.00000 and 0.30697
Refinement method	Full-matrix least-squares on F^2
Data / restraints / parameters	6455 / 2 / 316
Goodness-of-fit on F^2	1.027
Final R indices [I>2sigma(I)]	R1 = 0.0652, wR2 = 0.1506
R indices (all data)	R1 = 0.1185, wR2 = 0.1814
Absolute structure parameter	-1.4(10)
Extinction coefficient	n/a
Largest diff. peak and hole	0.230 and -0.239 e.$Å^{-3}$

7.3 Absolute Configuration of 4z

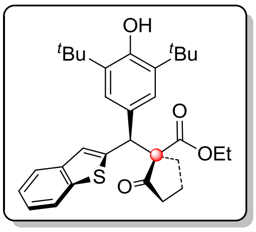

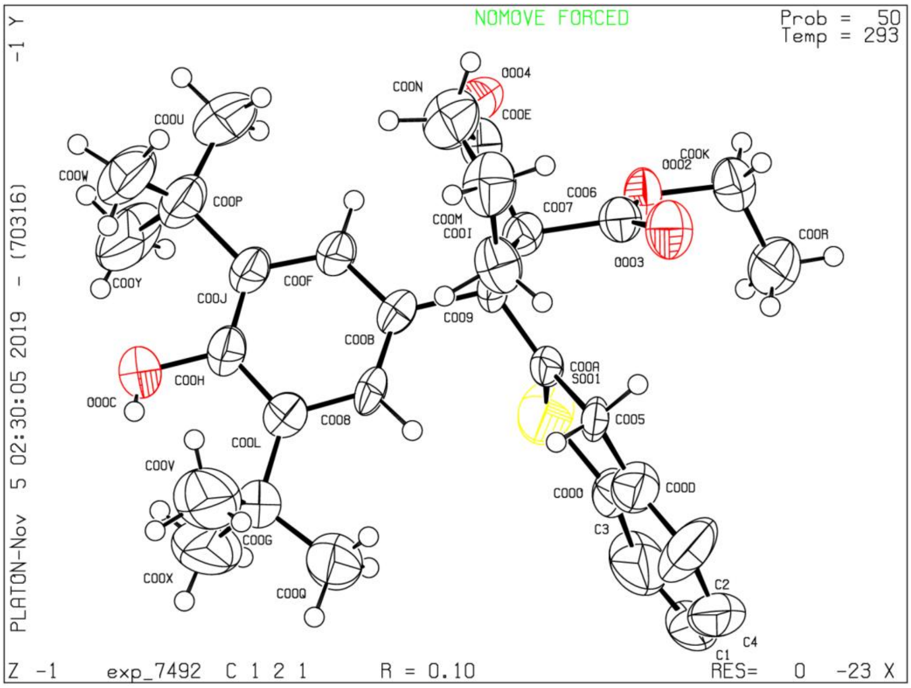

Figure S3. ORTEP Drawing of **4z** (CCDC1963687)

Identification code	exp_7492	
Empirical formula	C31 H39 O4 S	
Formula weight	507.68	
Temperature	293(2) K	
Wavelength	0.71073 Å	
Crystal system	Monoclinic	
Space group	C 1 2 1	
Unit cell dimensions	a = 23.808(4) Å	= 90°.
	b = 10.360(3) Å	= 95.046(15)°.
	c = 11.8065(17) Å	= 90°.
Volume	2900.7(9) Å^3	
Z	4	
Density (calculated)	1.163 Mg/m^3	
Absorption coefficient	0.144 mm^{-1}	
F(000)	1092	

Crystal size	? x ? x ? mm^3
Theta range for data collection	3.436 to 29.479°.
Index ranges	-32<=h<=23, -13<=k<=13, -15<=l<=15
Reflections collected	11710
Independent reflections	6219 [R(int) = 0.0758]
Completeness to theta = 25.242°	99.7 %
Absorption correction	Semi-empirical from equivalents
Max. and min. transmission	1.00000 and 0.50177
Refinement method	Full-matrix least-squares on F^2
Data / restraints / parameters	6219 / 3 / 333
Goodness-of-fit on F^2	0.992
Final R indices [I>2sigma(I)]	R1 = 0.1019, wR2 = 0.2143
R indices (all data)	R1 = 0.1964, wR2 = 0.2670
Absolute structure parameter	-0.02(18)
Extinction coefficient	n/a
Largest diff. peak and hole	0.350 and -0.328 e.$Å^{-3}$

VIII. NMR Spectra and HPLC Spectra

Please refer to next pages

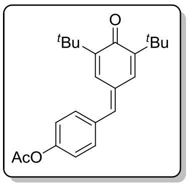
O
tBu
tBu
AcO

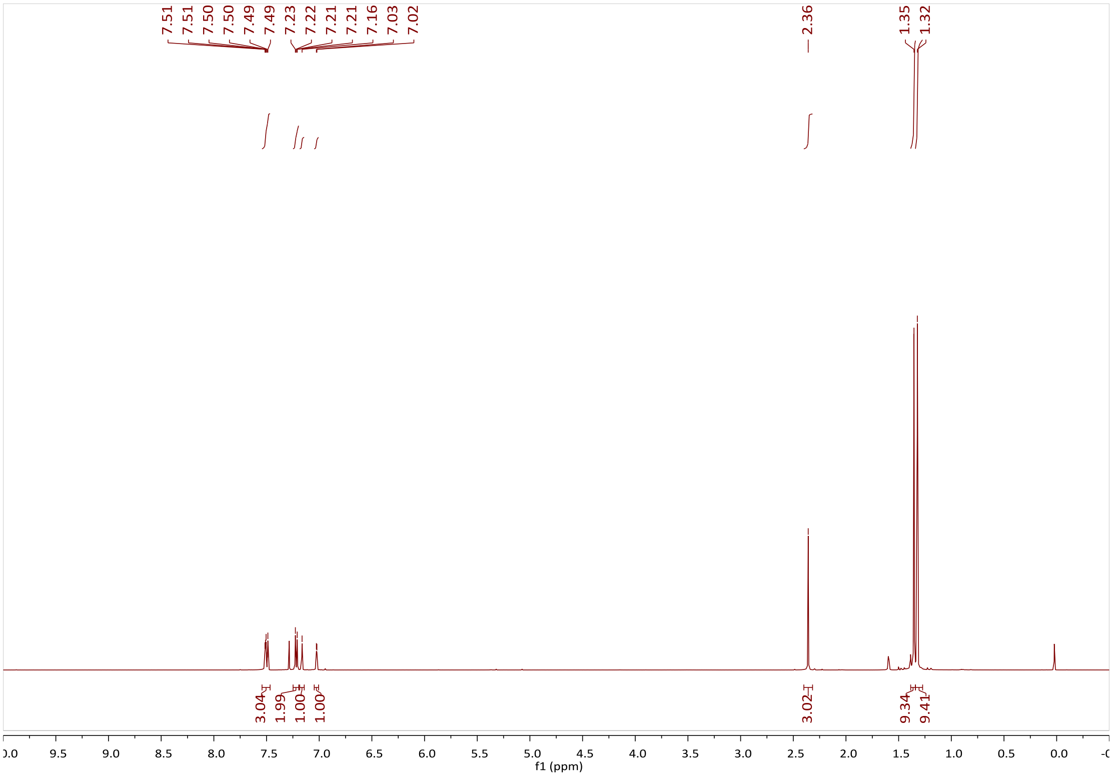
7.51
7.51
7.50
7.50
7.49
7.49
7.23
7.22
7.21
7.21
7.16
7.03
7.02
2.36
1.35
1.32
3.04
1.99
1.00
1.00
3.02
9.34
9.41
f1 (ppm)

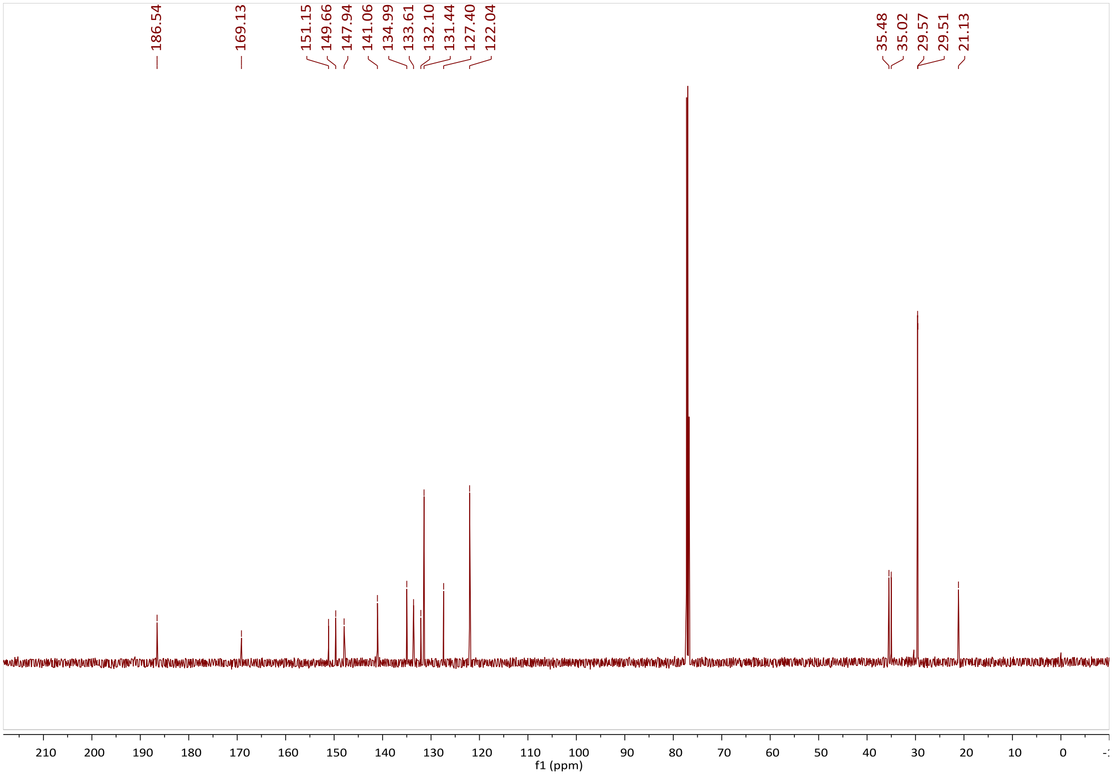
186.54
169.13
151.15
149.66
147.94
141.06
134.99
133.61
132.10
131.44
127.40
122.04
35.48
35.02
29.57
29.51
21.13
f1 (ppm)

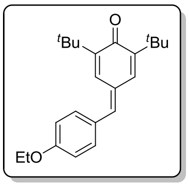
O
tBu
tBu
EtO

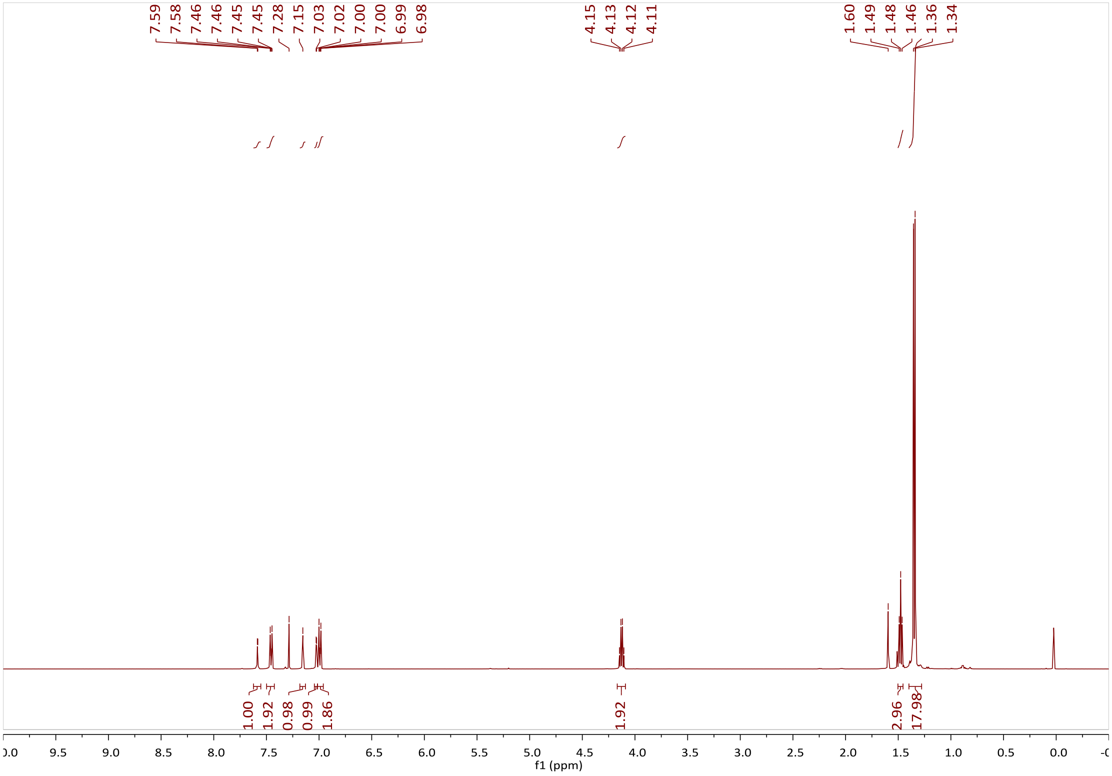
7.59
7.58
7.46
7.46
7.45
7.45
7.28
7.15
7.03
7.02
7.00
7.00
6.99
6.98
4.15
4.13
4.12
4.11
1.60
1.49
1.48
1.46
1.36
1.34
1.00
1.92
0.98
0.99
1.86
1.92
2.96
17.98
f1 (ppm)

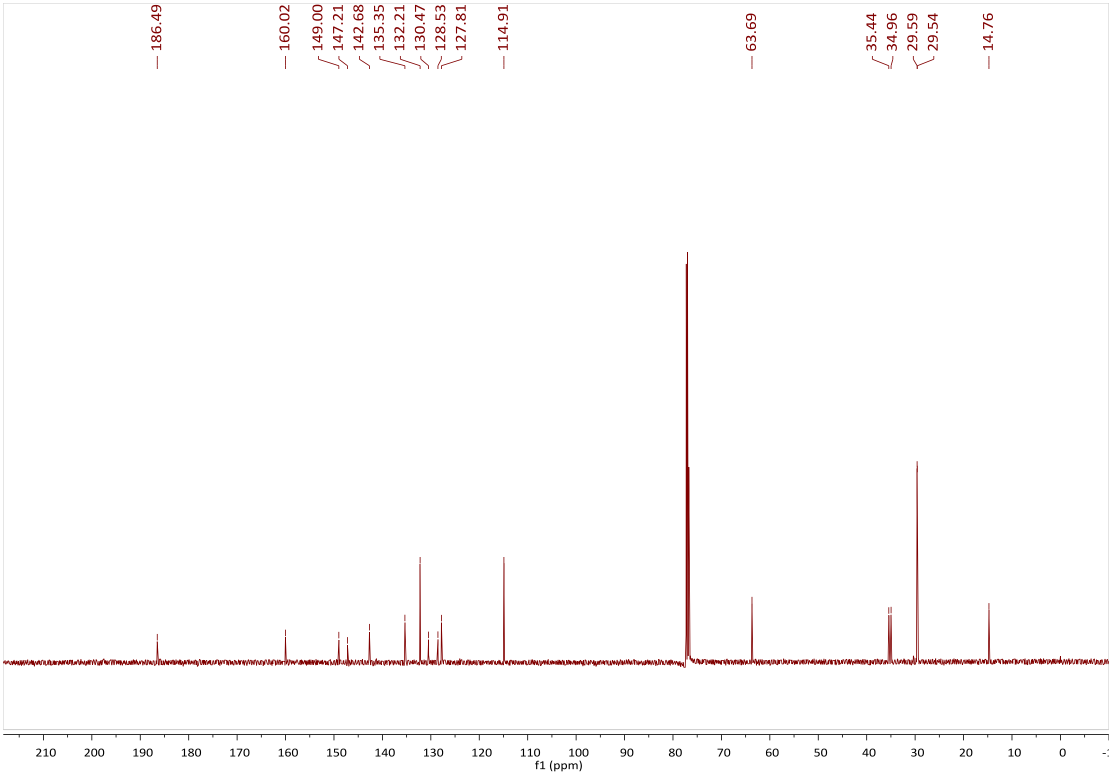
186.49
160.02
149.00
147.21
142.68
135.35
132.21
130.47
128.53
127.81
114.91
63.69
35.44
34.96
29.59
29.54
14.76
f1 (ppm)

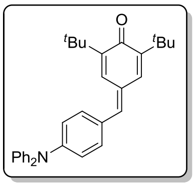
O
tBu
tBu
Ph2N

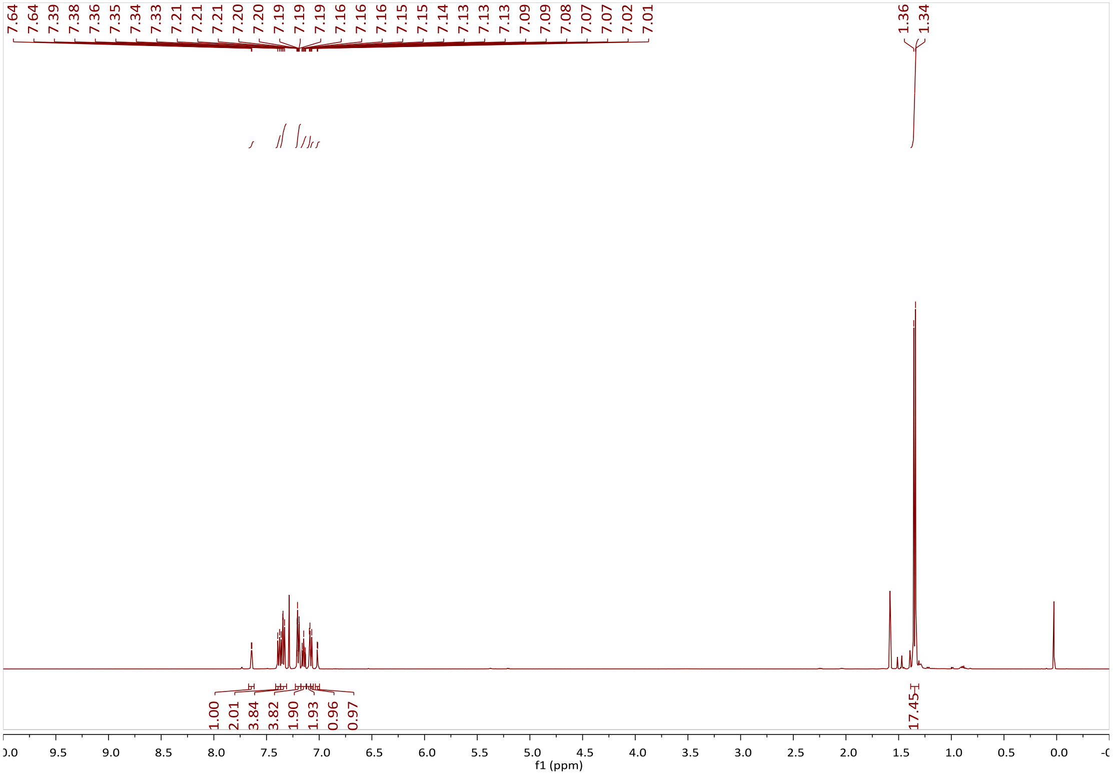
7.64 7.64 7.39 7.38 7.36 7.35 7.34 7.33 7.21 7.21 7.20 7.20 7.19 7.19 7.19 7.16 7.16 7.15 7.15 7.14 7.13 7.13 7.13 7.09 7.09 7.08 7.07 7.07 7.02 7.01
1.36 1.34
1.00 2.01 3.84 3.82 1.90 1.93 0.96 0.97
17.45
f1 (ppm)

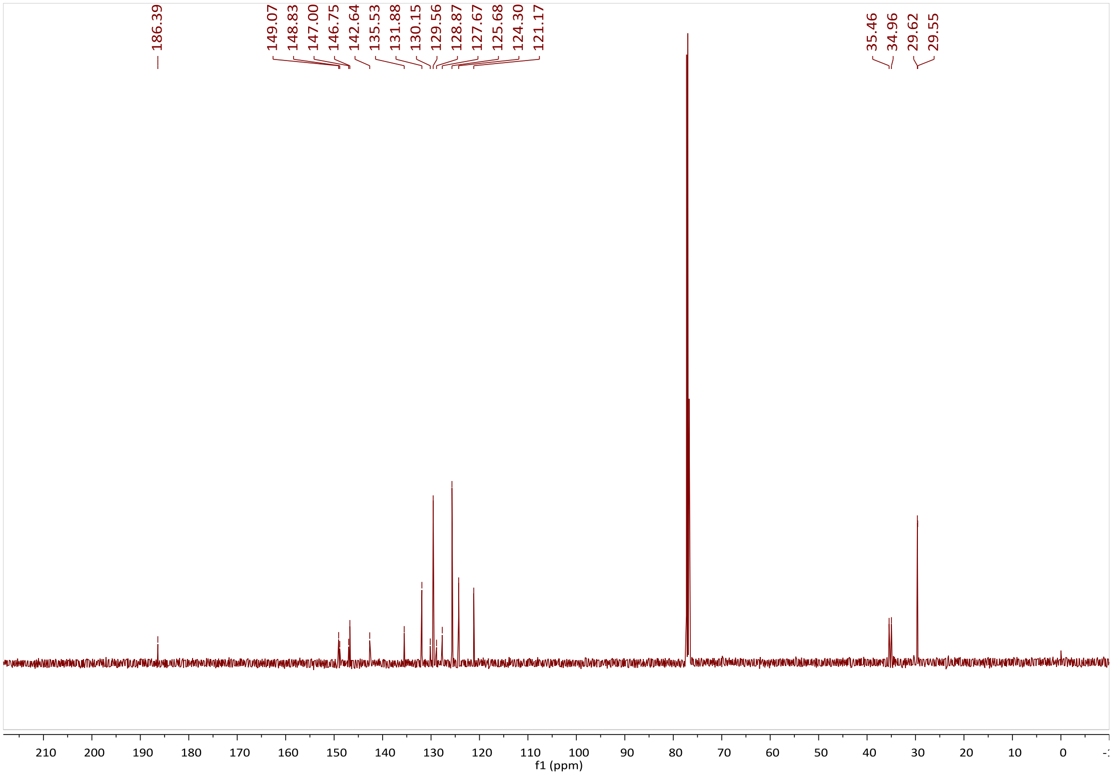
186.39
149.07 148.83 147.00 146.75 142.64 135.53 131.88 130.15 129.56 128.87 127.67 125.68 124.30 121.17
35.46 34.96 29.62 29.55
f1 (ppm)

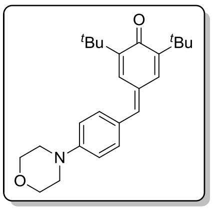

O
tBu
tBu
N
O

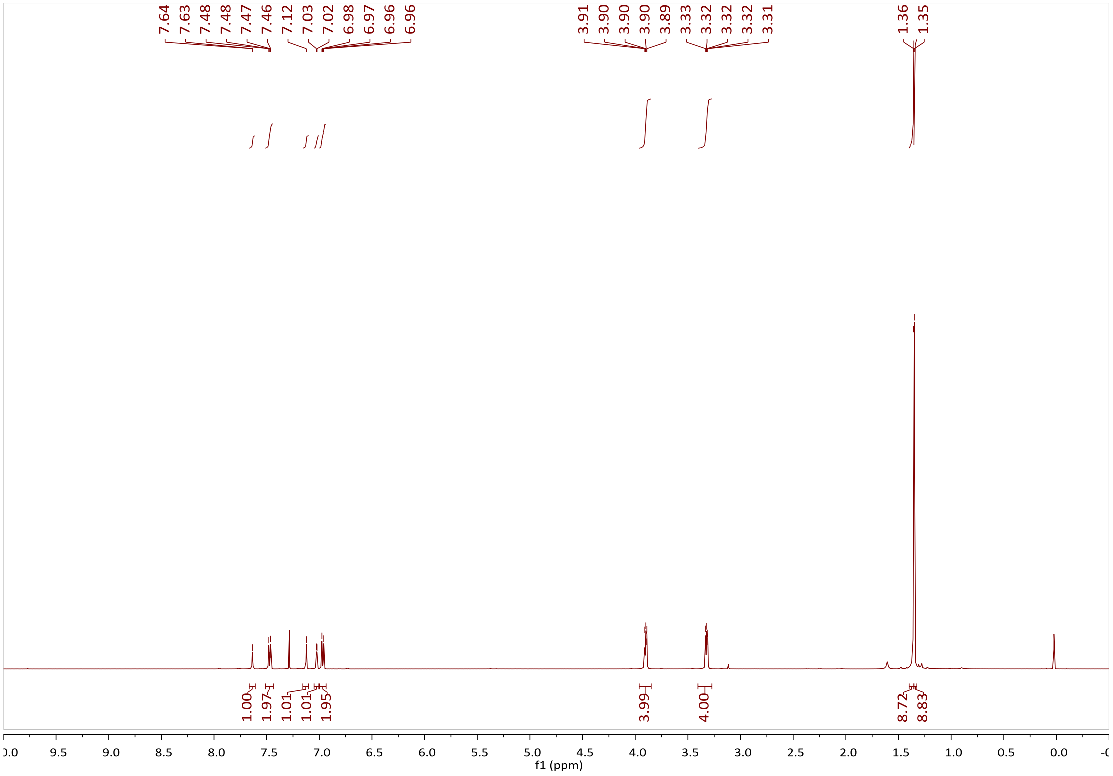

7.64
7.63
7.48
7.48
7.47
7.46
7.12
7.03
7.02
6.98
6.97
6.96
6.96
3.91
3.90
3.90
3.90
3.89
3.33
3.32
3.32
3.32
3.31
1.36
1.35
1.00
1.97
1.01
1.01
1.95
3.99
4.00
8.72
8.83
f1 (ppm)

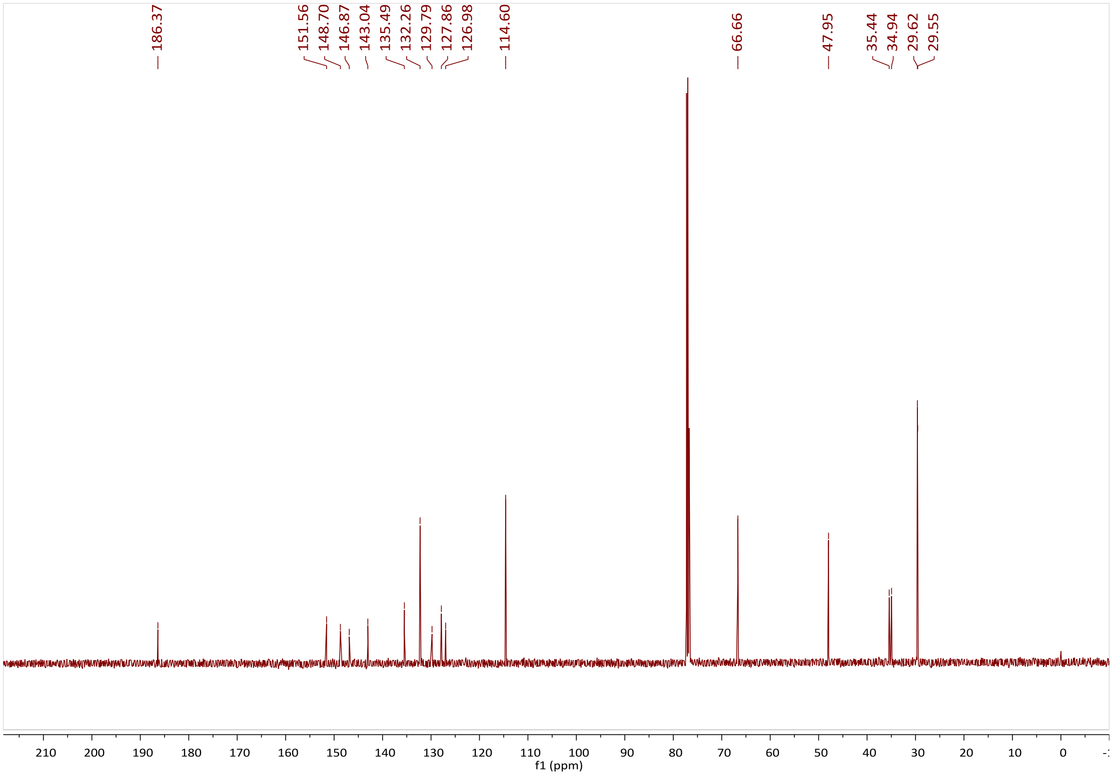

186.37
151.56
148.70
146.87
143.04
135.49
132.26
129.79
127.86
126.98
114.60
66.66
47.95
35.44
34.94
29.62
29.55
f1 (ppm)

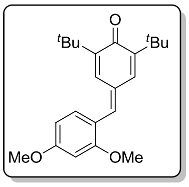

O
tBu
tBu
MeO
OMe

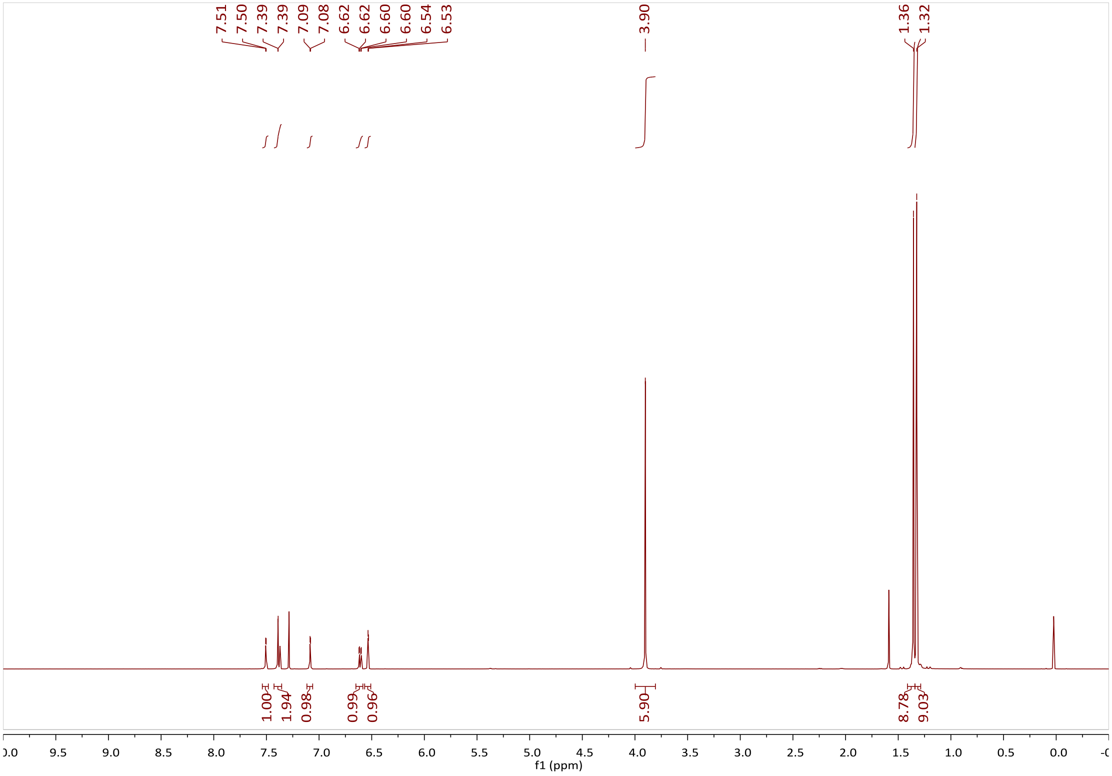

7.51
7.50
7.39
7.39
7.09
7.08
6.62
6.62
6.60
6.60
6.54
6.53
3.90
1.36
1.32
1.00
1.94
0.98
0.99
0.96
5.90
8.78
9.03
f1 (ppm)

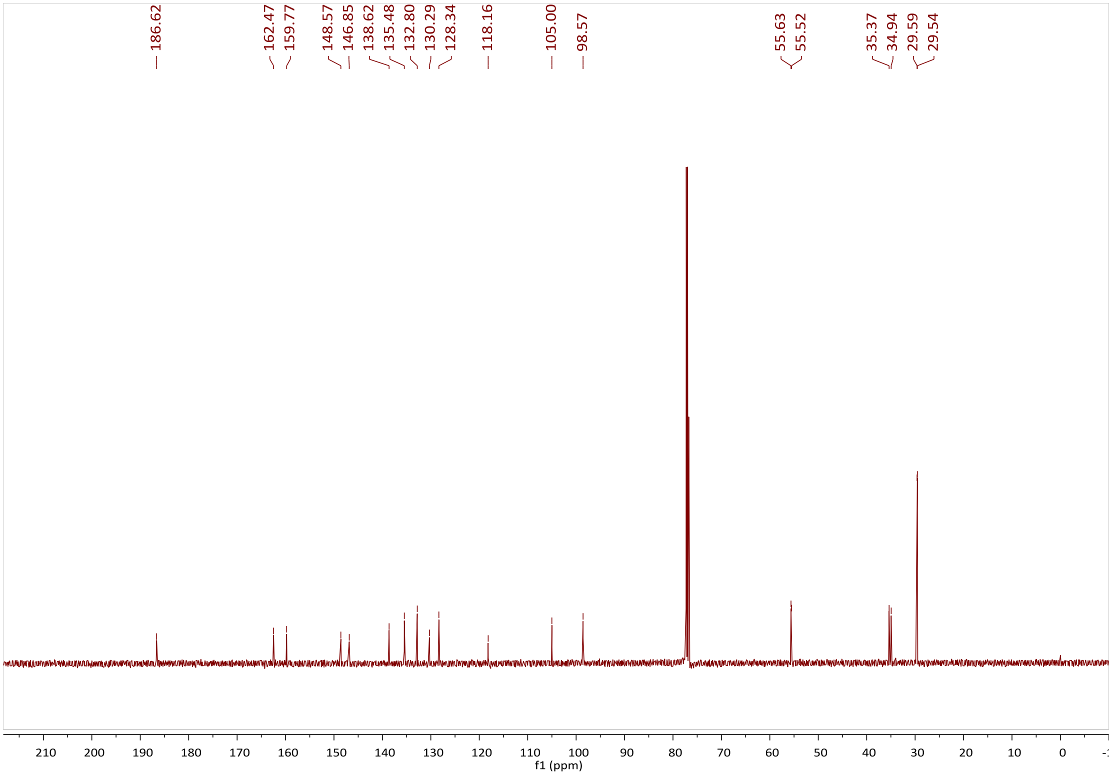

186.62
162.47
159.77
148.57
146.85
138.62
135.48
132.80
130.29
128.34
118.16
105.00
98.57
55.63
55.52
35.37
34.94
29.59
29.54
f1 (ppm)

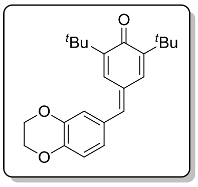
O
tBu
tBu
O
O

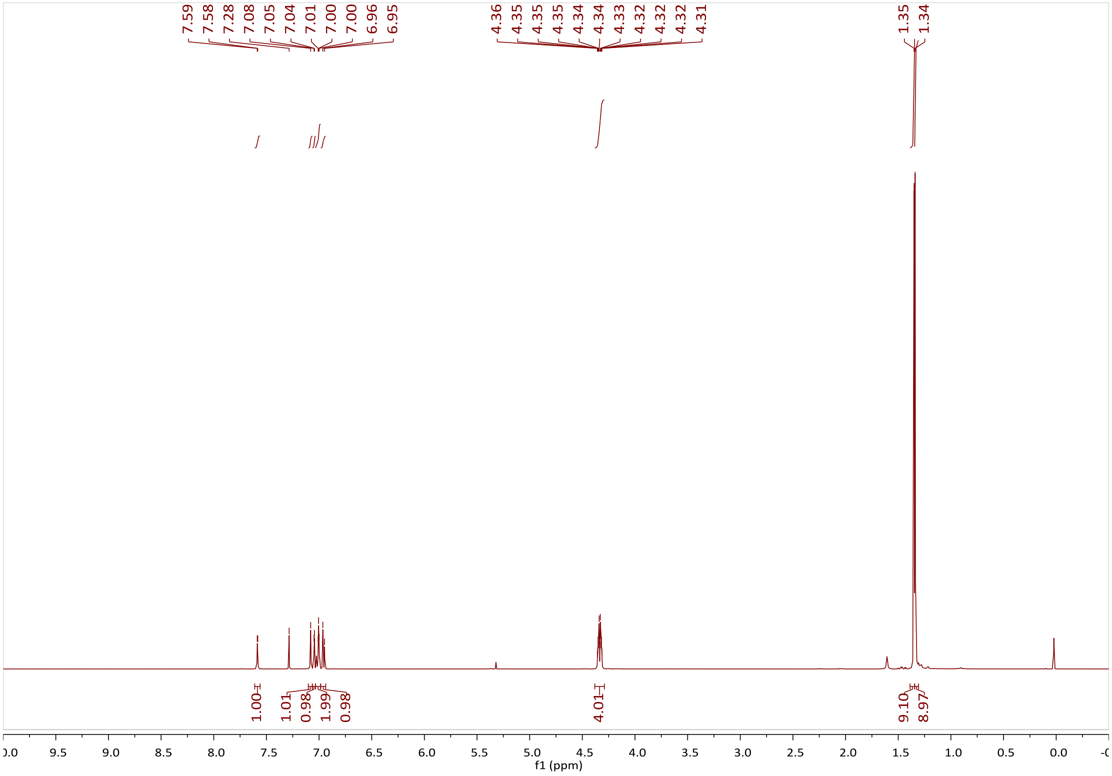
7.59
7.58
7.28
7.08
7.05
7.04
7.01
7.00
7.00
6.96
6.95
4.36
4.35
4.35
4.35
4.34
4.34
4.33
4.32
4.32
4.32
4.31
1.35
1.34
1.00
1.01
0.98
1.99
0.98
4.01
9.10
8.97
f1 (ppm)

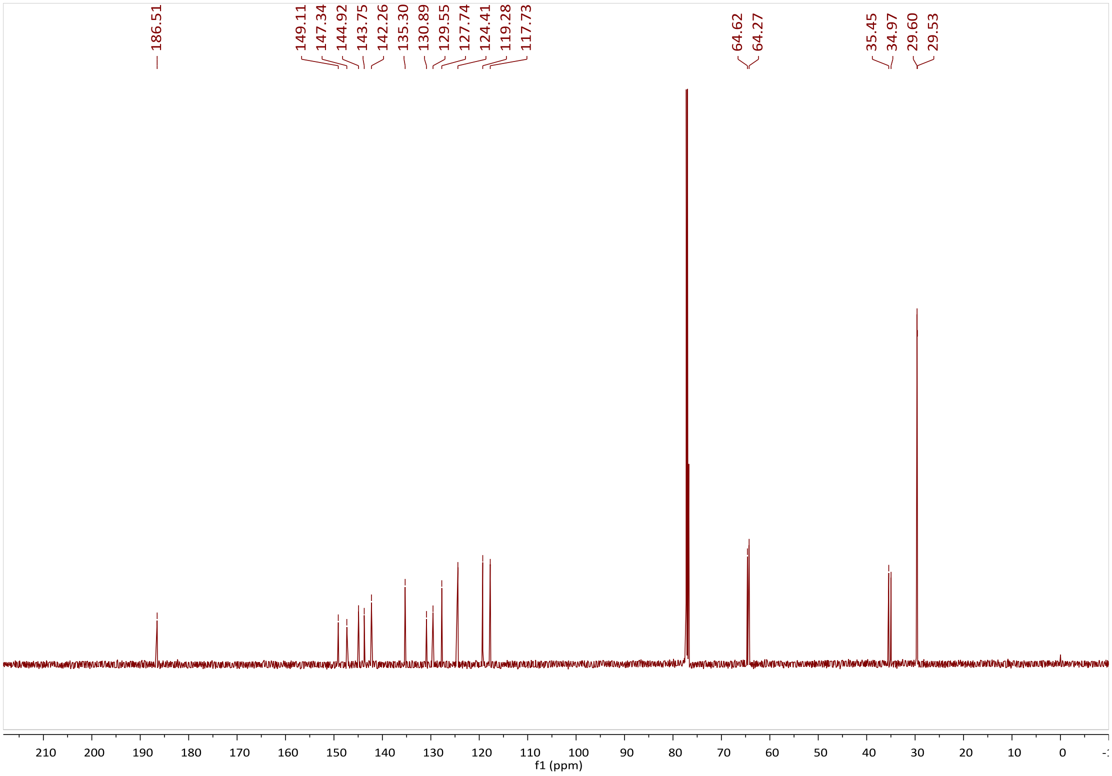
186.51
149.11
147.34
144.92
143.75
142.26
135.30
130.89
129.55
127.74
124.41
119.28
117.73
64.62
64.27
35.45
34.97
29.60
29.53
f1 (ppm)

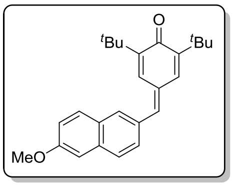
O
tBu
tBu
MeO

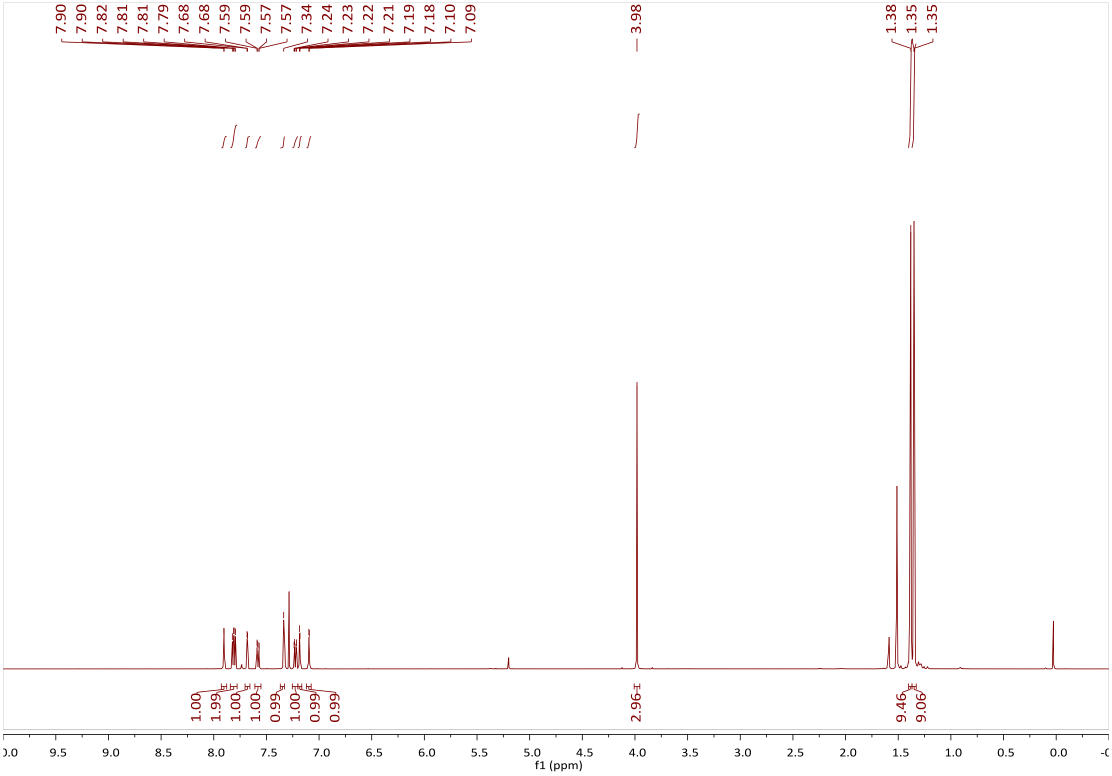
7.90
7.90
7.82
7.81
7.81
7.79
7.68
7.68
7.59
7.59
7.57
7.57
7.34
7.24
7.23
7.22
7.21
7.19
7.18
7.10
7.09
3.98
1.38
1.35
1.35
1.00
1.99
1.00
1.00
0.99
1.00
0.99
0.99
2.96
9.46
9.06
f1 (ppm)

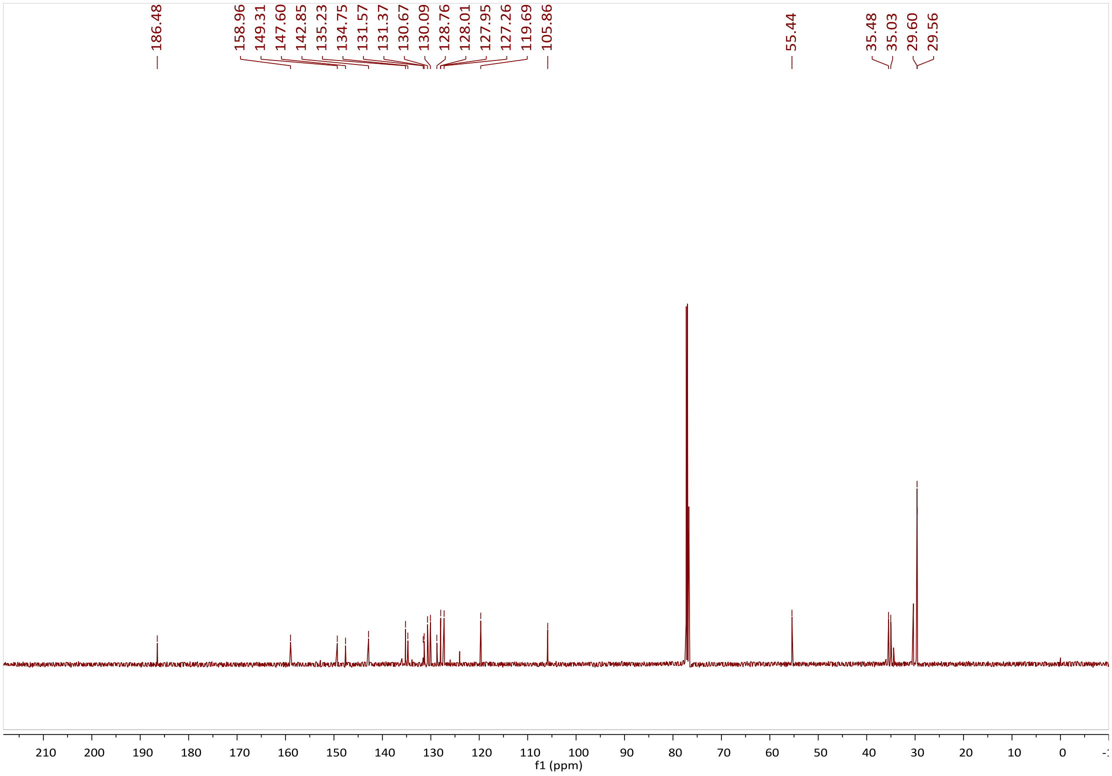
186.48
158.96
149.31
147.60
142.85
135.23
134.75
131.57
131.37
130.67
130.09
128.76
128.01
127.95
127.26
119.69
105.86
55.44
35.48
35.03
29.60
29.56
f1 (ppm)

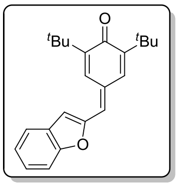
O
tBu
tBu
O

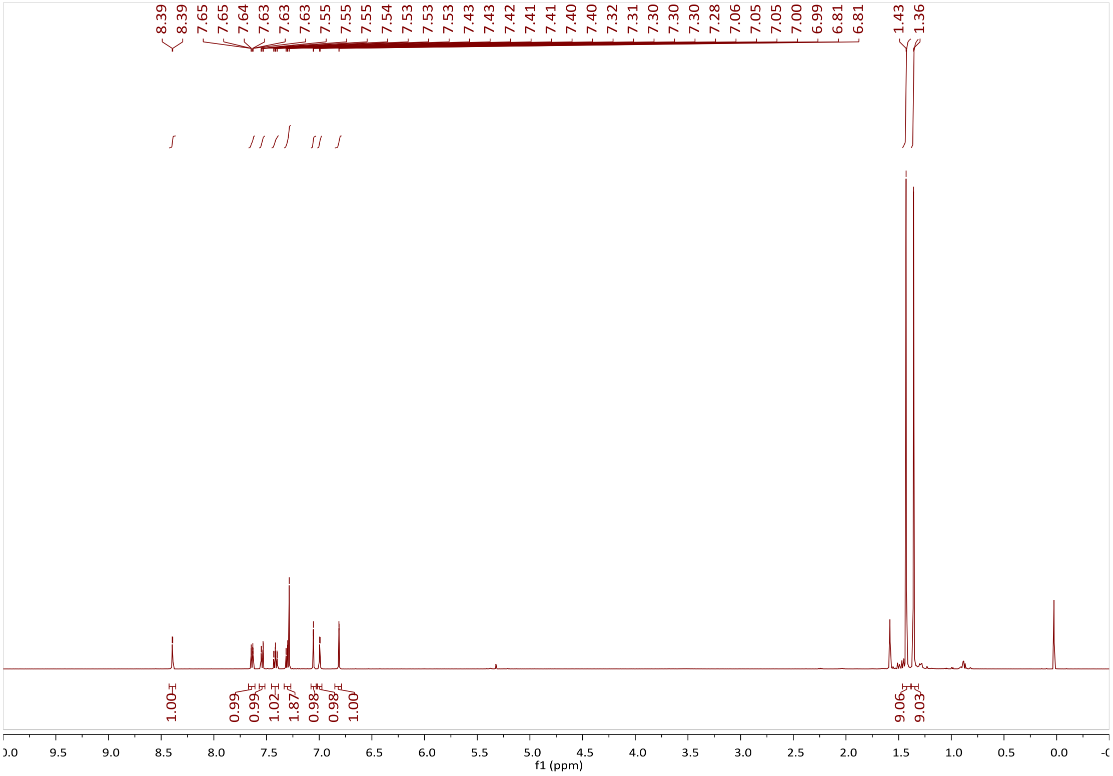

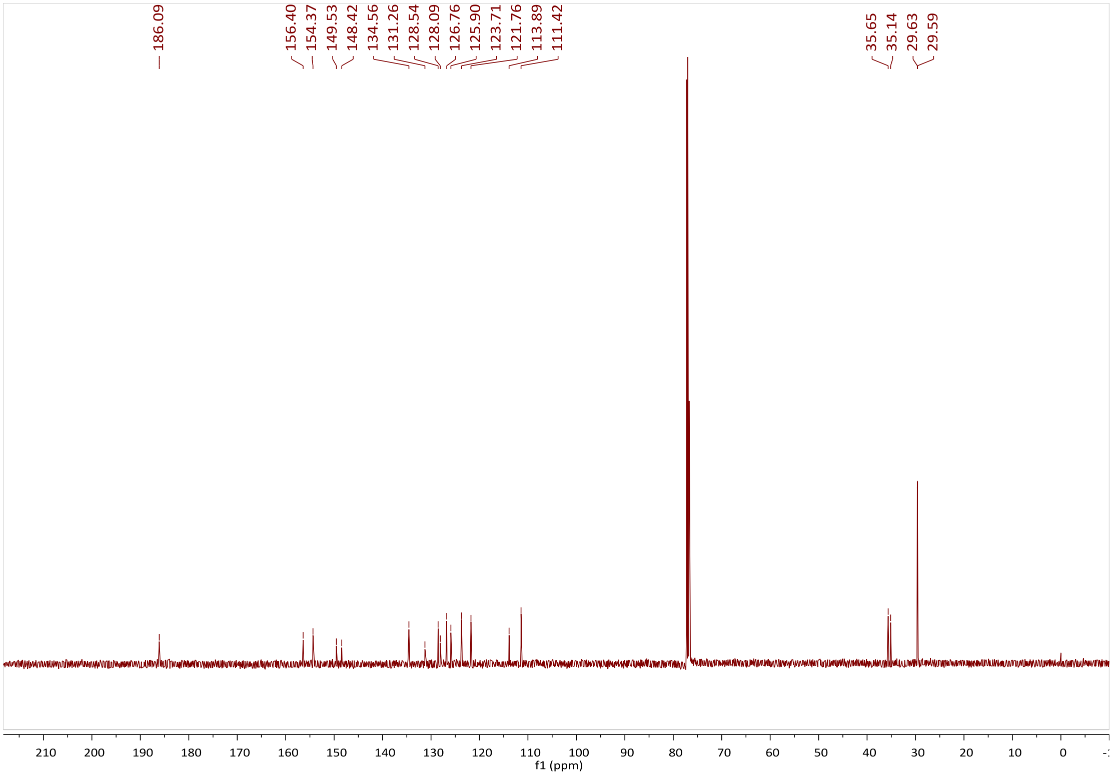

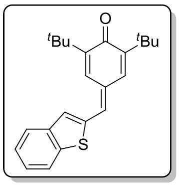
O
tBu
tBu
S

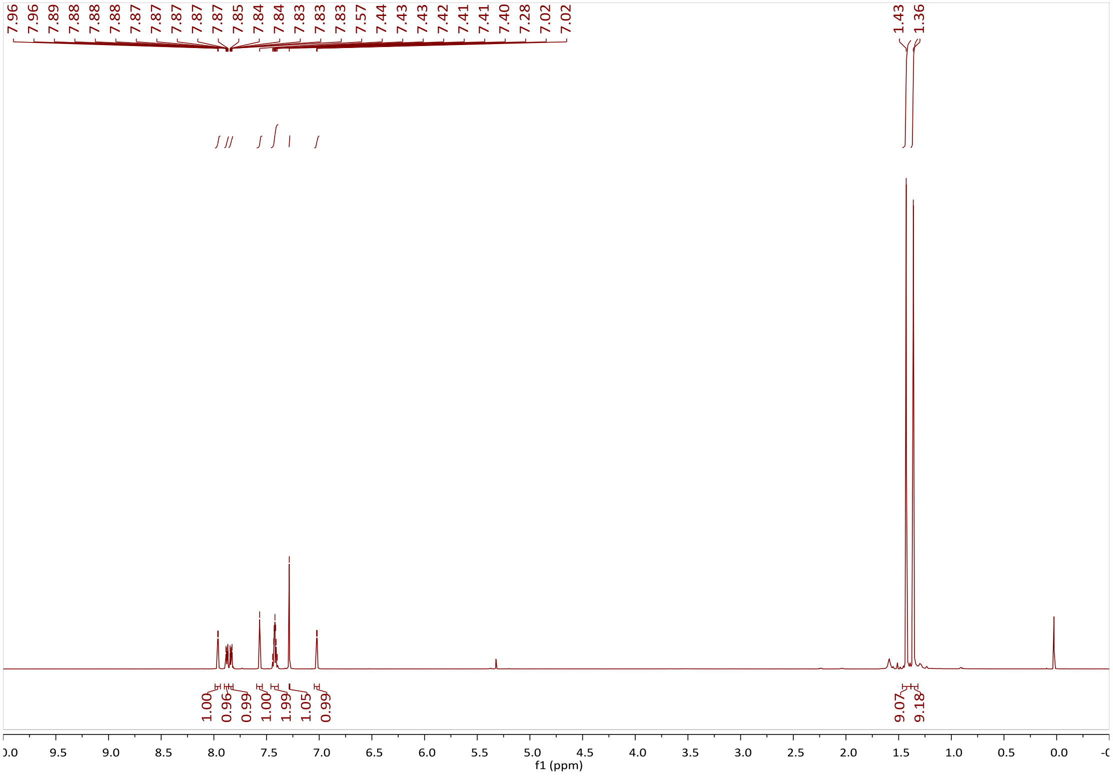
7.96 7.96 7.89 7.88 7.88 7.88 7.87 7.87 7.87 7.87 7.87 7.85 7.84 7.84 7.83 7.83 7.83 7.57 7.44 7.43 7.43 7.42 7.41 7.41 7.40 7.28 7.02 7.02
1.43 1.36
1.00 0.96 0.99 1.00 1.99 1.05 0.99
9.07 9.18
f1 (ppm)

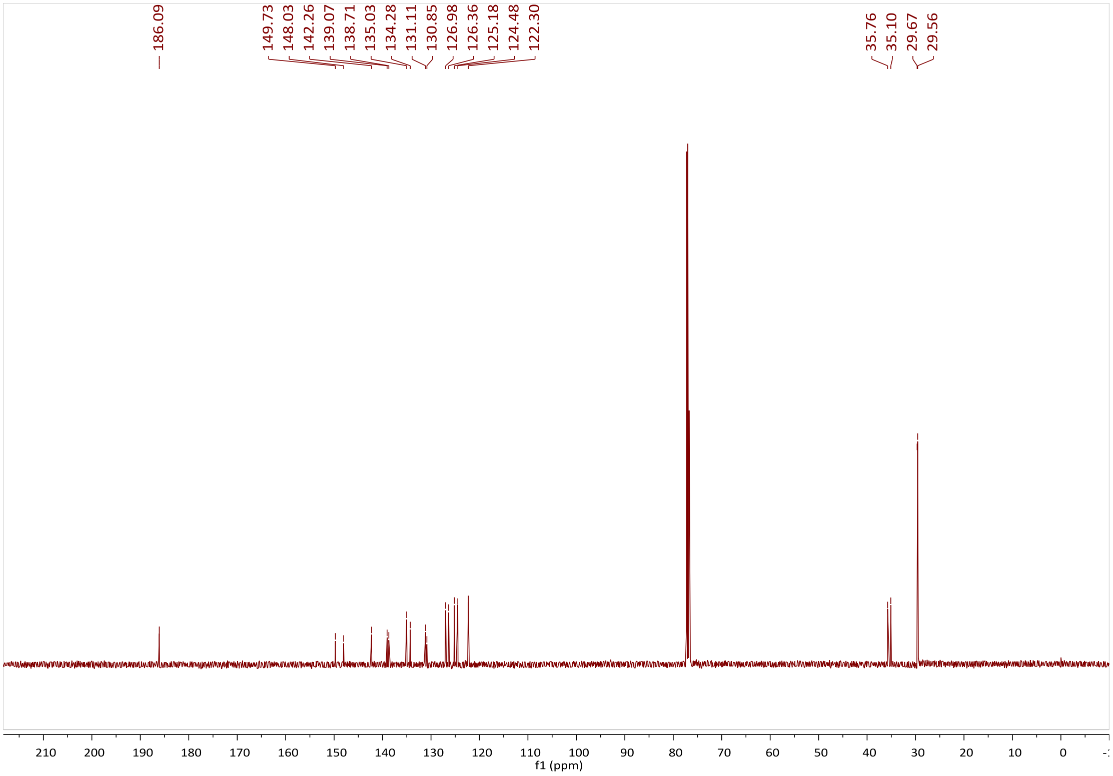
186.09
149.73 148.03 142.26 139.07 138.71 135.03 134.28 131.11 130.85 126.98 126.36 125.18 124.48 122.30
35.76 35.10 29.67 29.56
f1 (ppm)

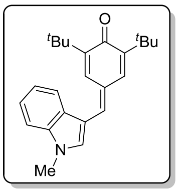
O
tBu
tBu
N
Me

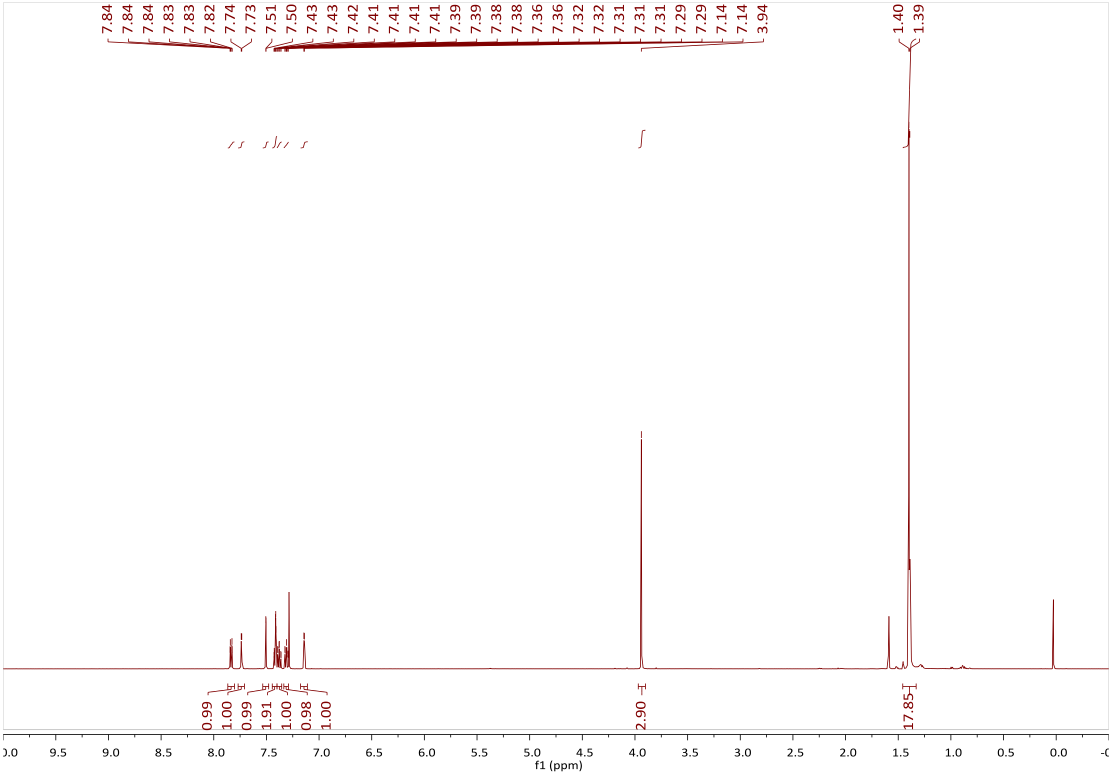
7.84
7.84
7.84
7.83
7.83
7.82
7.74
7.73
7.51
7.50
7.43
7.43
7.42
7.41
7.41
7.41
7.41
7.39
7.39
7.39
7.38
7.38
7.36
7.36
7.32
7.32
7.31
7.31
7.31
7.29
7.29
7.14
7.14
3.94
1.40
1.39
0.99
1.00
0.99
1.91
1.00
0.98
1.00
2.90
17.85
f1 (ppm)

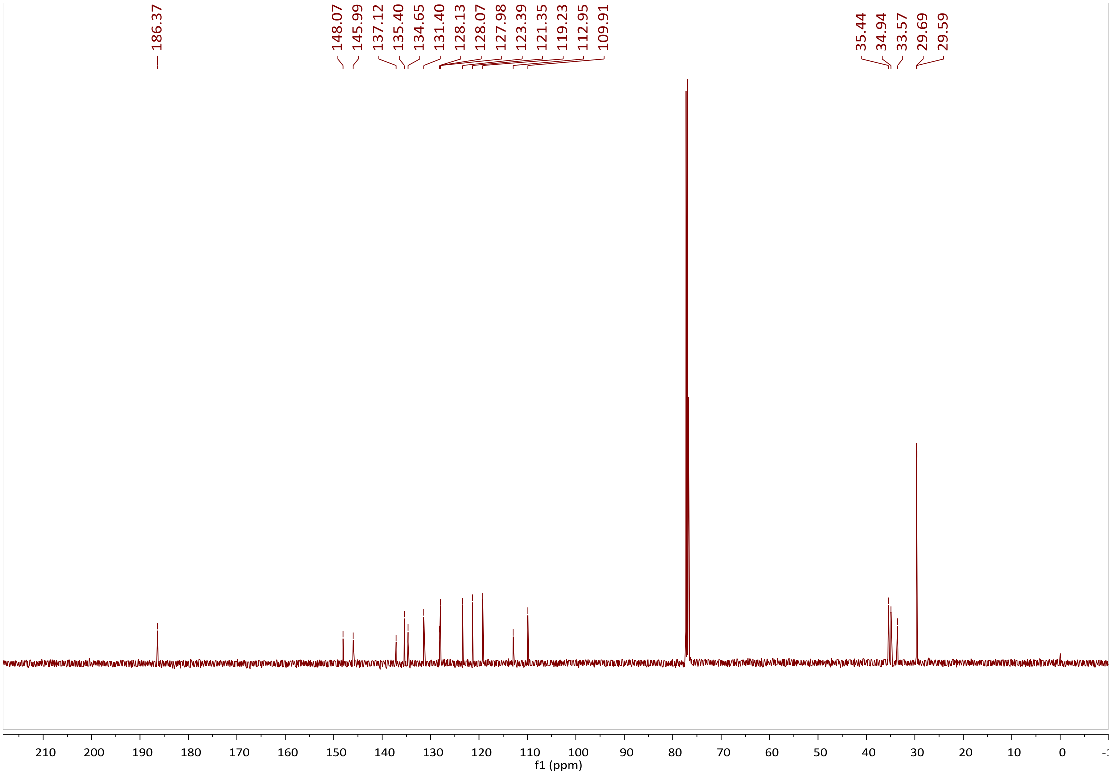
186.37
148.07
145.99
137.12
135.40
134.65
131.40
128.13
128.07
127.98
123.39
121.35
119.23
112.95
109.91
35.44
34.94
33.57
29.69
29.59
f1 (ppm)

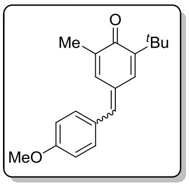
O
Me
tBu
MeO

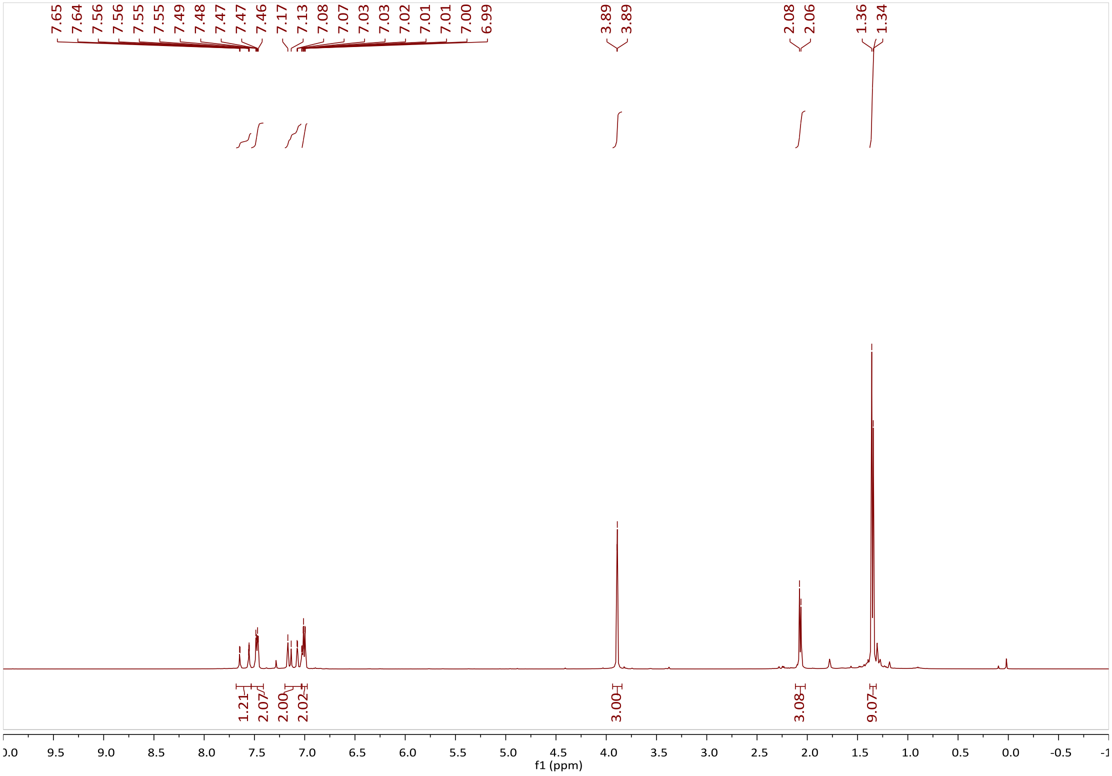
7.65
7.64
7.56
7.56
7.55
7.55
7.49
7.48
7.47
7.47
7.46
7.17
7.13
7.08
7.07
7.03
7.03
7.02
7.01
7.01
7.00
6.99
3.89
3.89
2.08
2.06
1.36
1.34
1.21
2.07
2.00
2.02
3.00
3.08
9.07
f1 (ppm)

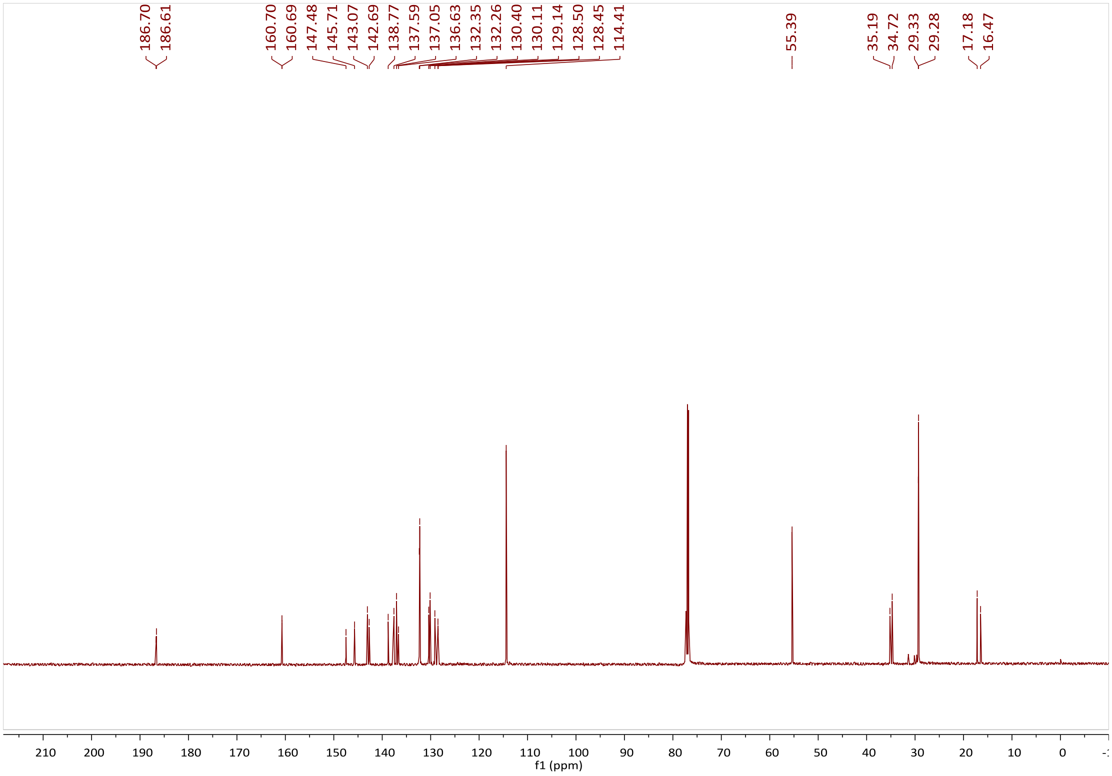
186.70
186.61
160.70
160.69
147.48
145.71
143.07
142.69
138.77
137.59
137.05
136.63
132.35
132.26
130.40
130.11
129.14
128.50
128.45
114.41
55.39
35.19
34.72
29.33
29.28
17.18
16.47
f1 (ppm)

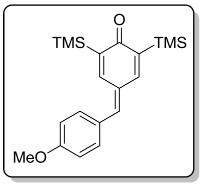
O
TMS
TMS
MeO

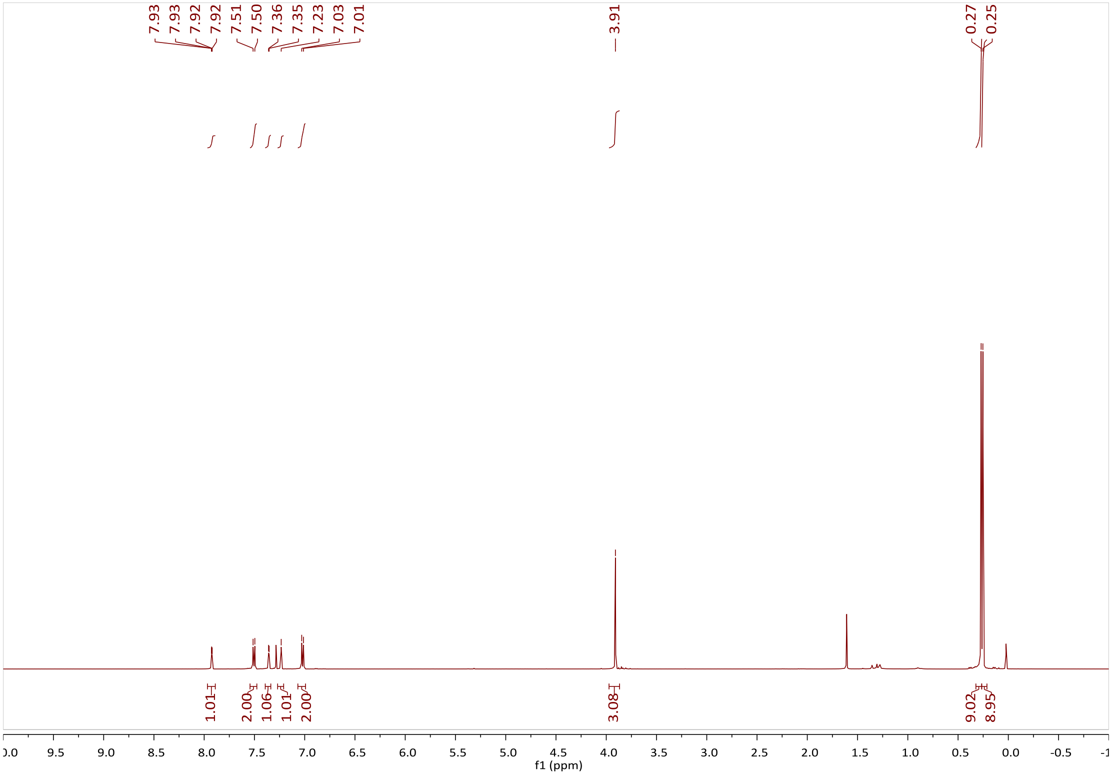
7.93
7.93
7.92
7.92
7.51
7.50
7.36
7.35
7.23
7.03
7.01
3.91
0.27
0.25
1.01
2.00
1.06
1.01
2.00
3.08
9.02
8.95
f1 (ppm)

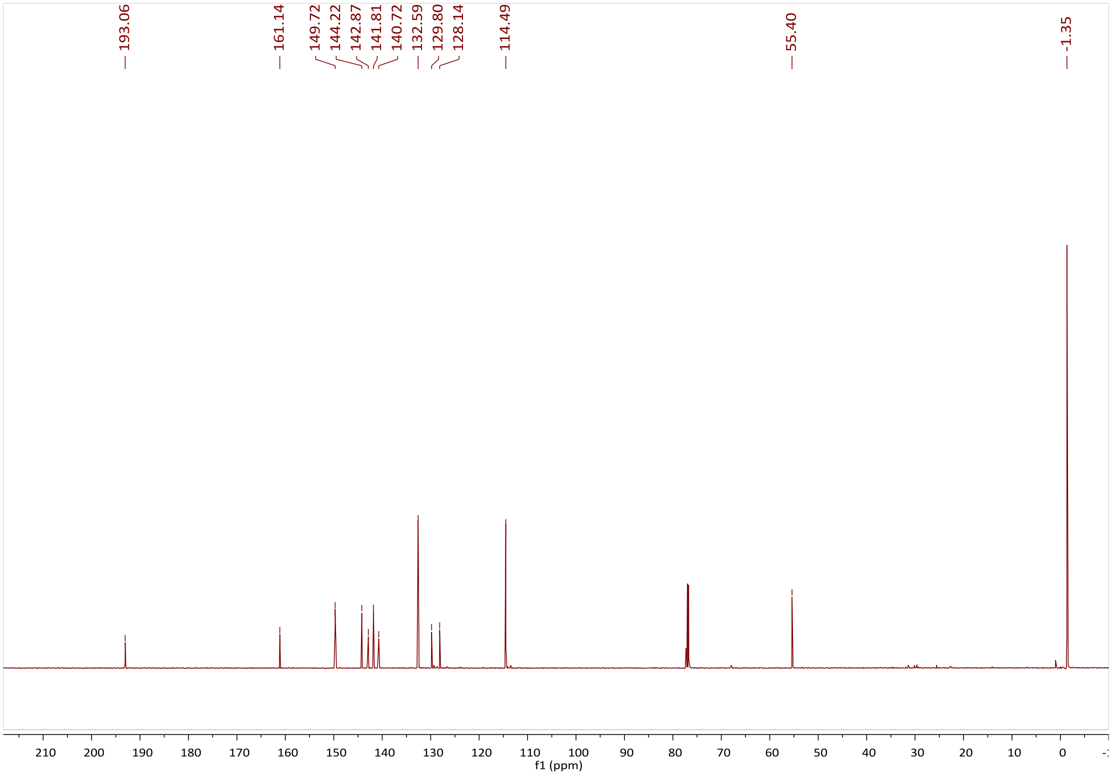
193.06
161.14
149.72
144.22
142.87
141.81
140.72
132.59
129.80
128.14
114.49
55.40
-1.35
f1 (ppm)

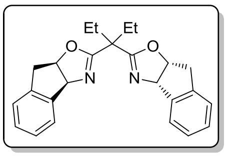
Et
Et
O
O
N
N

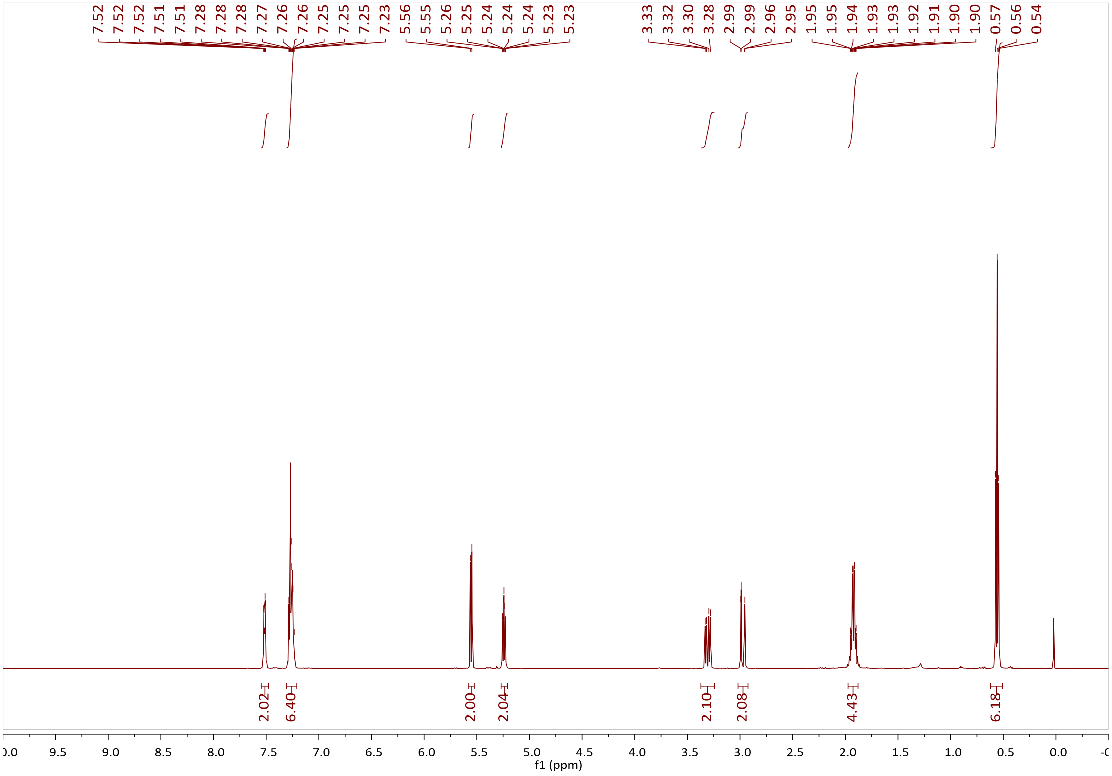
7.52
7.52
7.52
7.51
7.51
7.28
7.28
7.28
7.27
7.26
7.26
7.25
7.25
7.25
7.23
5.56
5.55
5.26
5.25
5.24
5.24
5.24
5.23
5.23
3.33
3.32
3.30
3.28
2.99
2.99
2.96
2.95
1.95
1.95
1.94
1.93
1.93
1.92
1.91
1.90
1.90
0.57
0.56
0.54
2.02
6.40
2.00
2.04
2.10
2.08
4.43
6.18
f1 (ppm)

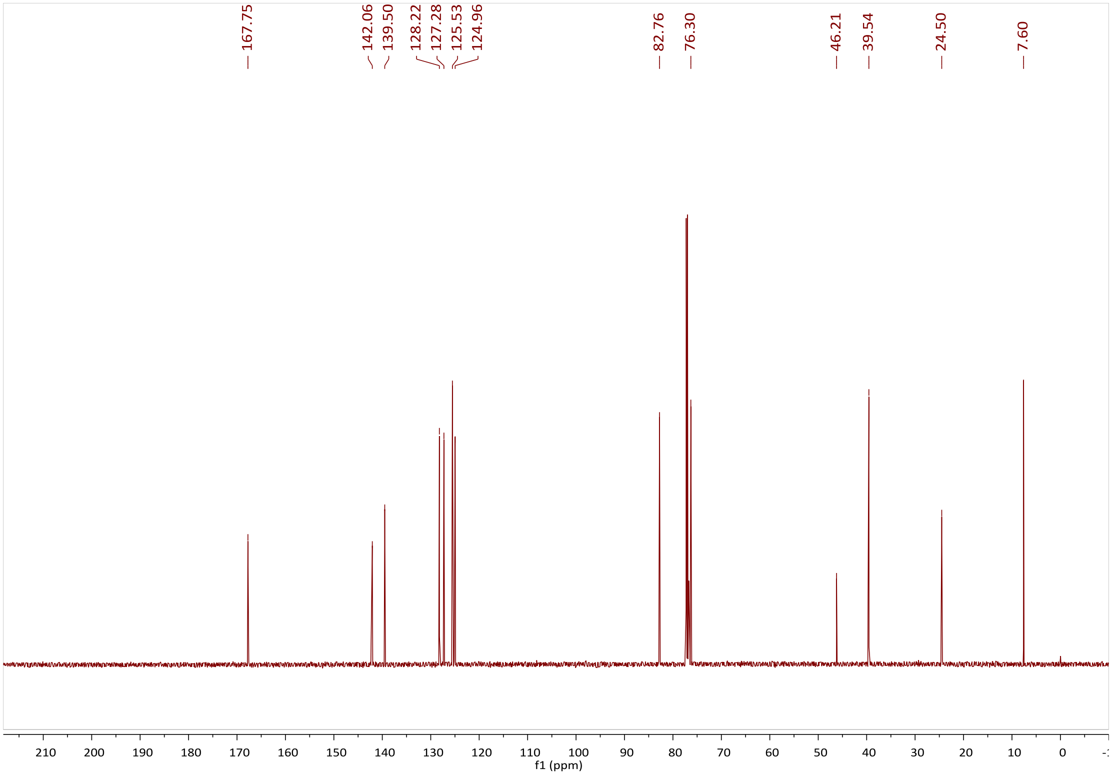
167.75
142.06
139.50
128.22
127.28
125.53
124.96
82.76
76.30
46.21
39.54
24.50
7.60
f1 (ppm)

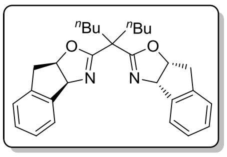

nBu nBu
O N O N

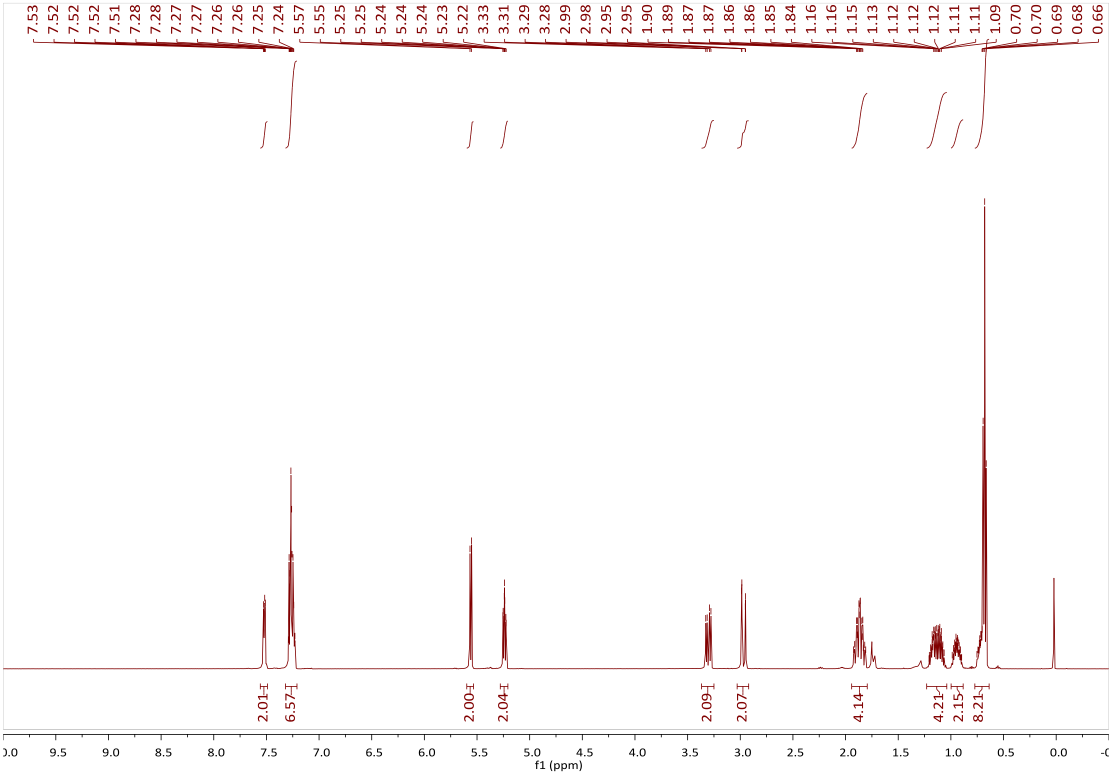

7.53 7.52 7.52 7.52 7.51 7.28 7.28 7.27 7.27 7.26 7.26 7.25 7.24 5.57 5.55 5.25 5.25 5.24 5.24 5.24 5.23 5.22 3.33 3.31 3.29 3.28 2.99 2.98 2.95 2.95 1.90 1.89 1.87 1.86 1.86 1.85 1.84 1.16 1.16 1.15 1.13 1.12 1.12 1.12 1.11 1.11 1.09 0.70 0.70 0.69 0.68 0.66
2.01 6.57 2.00 2.04 2.09 2.07 4.14 4.21 2.15 8.21
f1 (ppm)

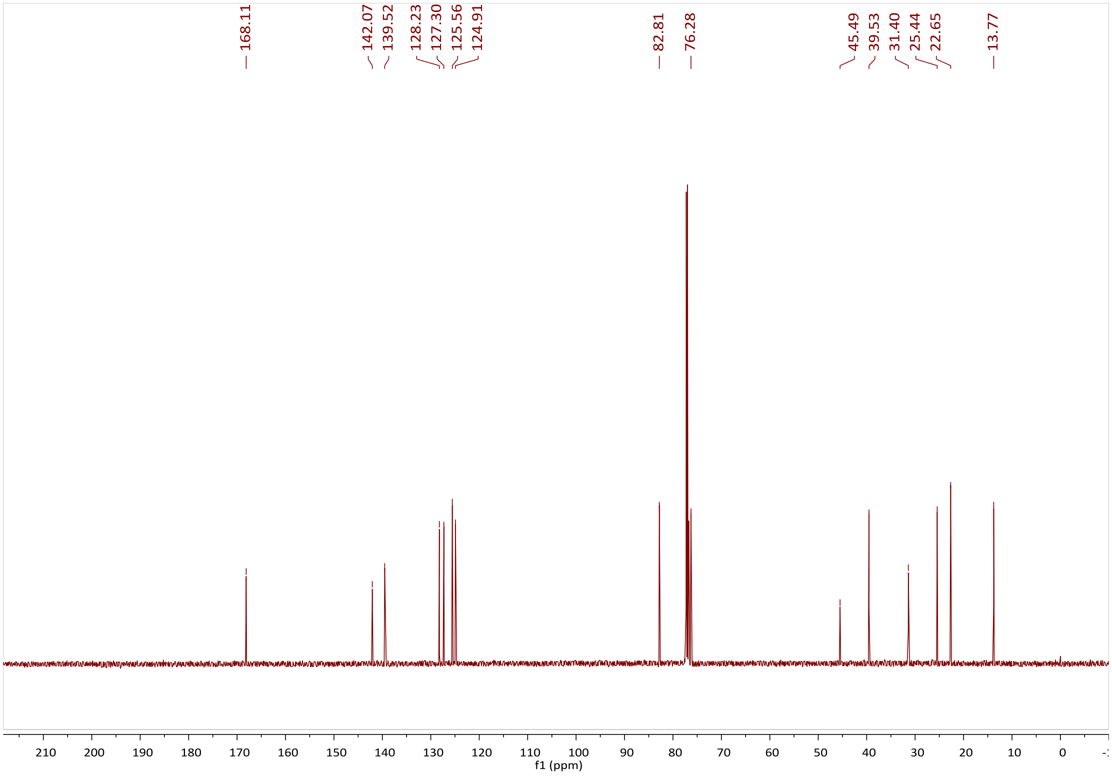

168.11 142.07 139.52 128.23 127.30 125.56 124.91 82.81 76.28 45.49 39.53 31.40 25.44 22.65 13.77
f1 (ppm)

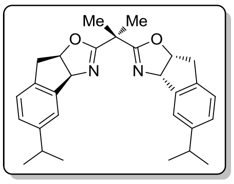
Me
Me
O
O
N
N

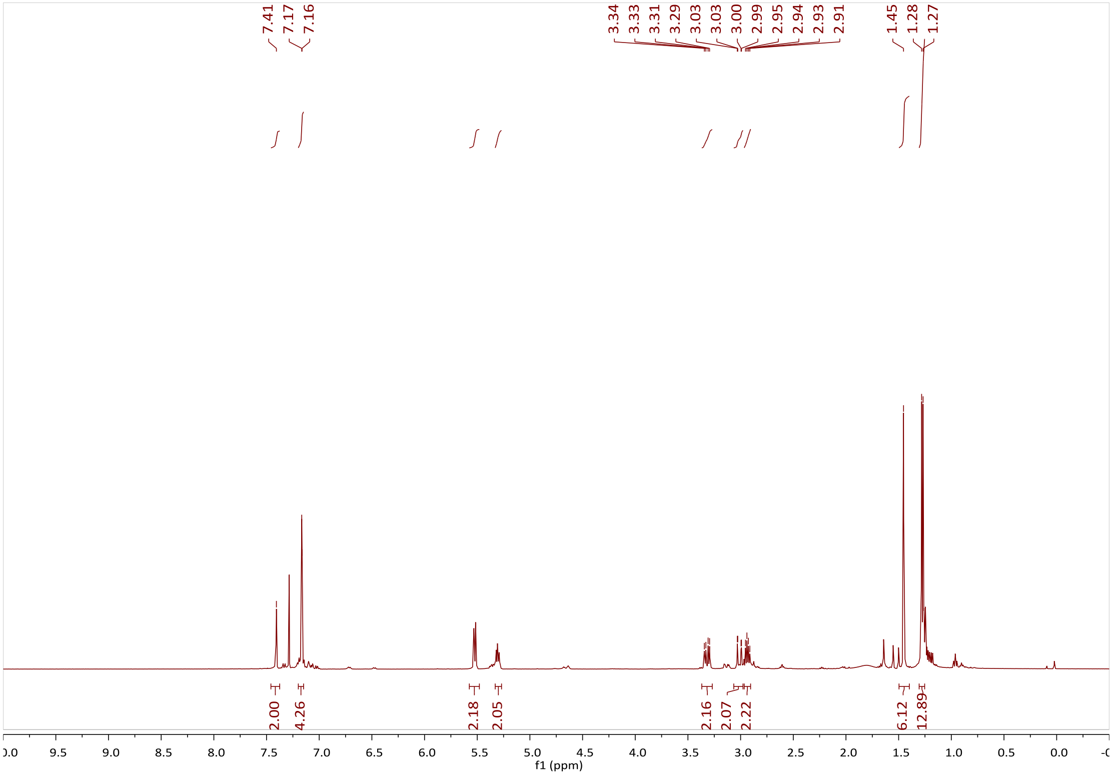
7.41
7.17
7.16
3.34
3.33
3.31
3.29
3.03
3.03
3.00
2.99
2.95
2.94
2.93
2.91
1.45
1.28
1.27
2.00
4.26
2.18
2.05
2.16
2.07
2.22
6.12
12.89
f1 (ppm)

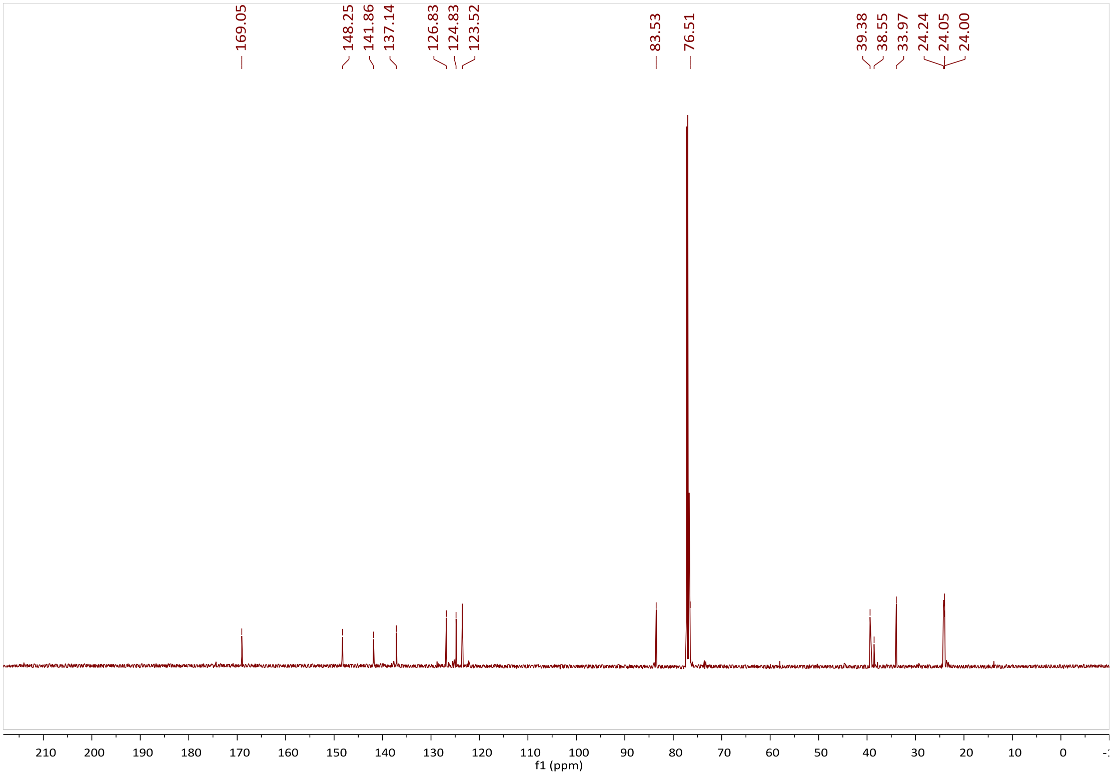
169.05
148.25
141.86
137.14
126.83
124.83
123.52
83.53
76.51
39.38
38.55
33.97
24.24
24.05
24.00
f1 (ppm)

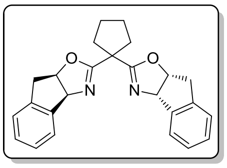
O
N
O
N

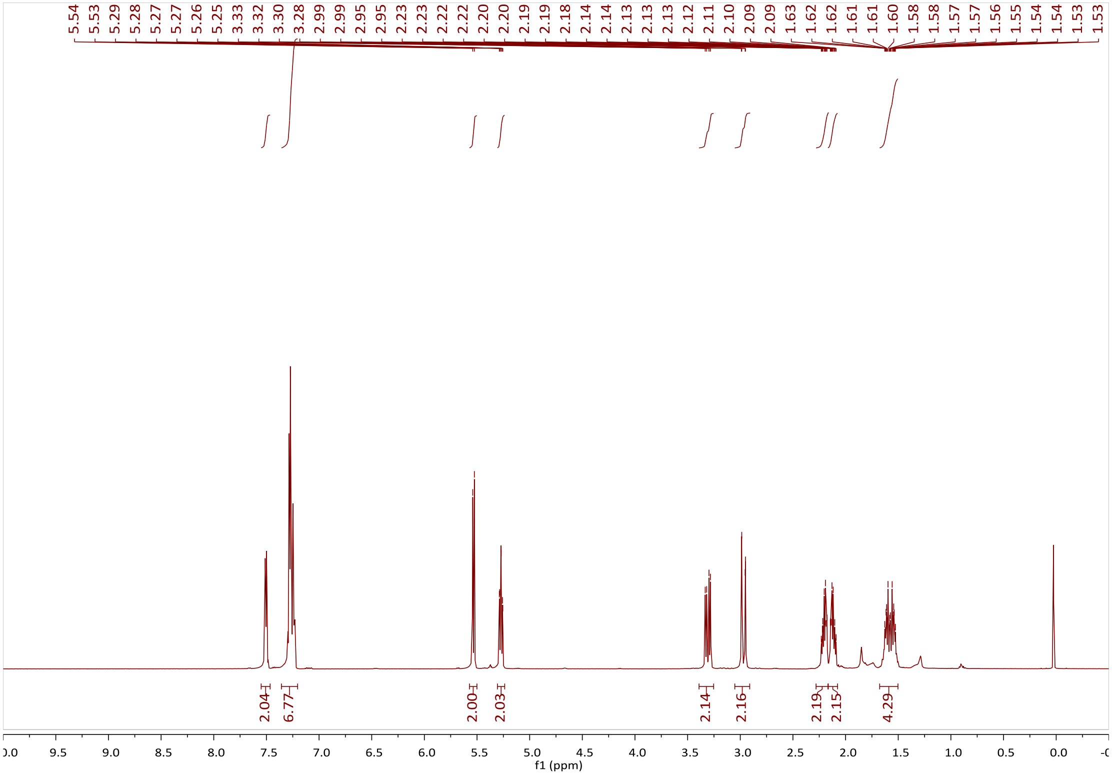
5.54
5.53
5.29
5.28
5.27
5.27
5.26
5.25
3.33
3.32
3.30
3.28
2.99
2.99
2.95
2.95
2.23
2.23
2.22
2.22
2.20
2.20
2.19
2.19
2.18
2.14
2.14
2.13
2.13
2.13
2.12
2.11
2.10
2.09
2.09
1.63
1.62
1.62
1.61
1.61
1.60
1.58
1.58
1.57
1.57
1.56
1.55
1.54
1.54
1.53
1.53
2.04
6.77
2.00
2.03
2.14
2.16
2.19
2.15
4.29
f1 (ppm)

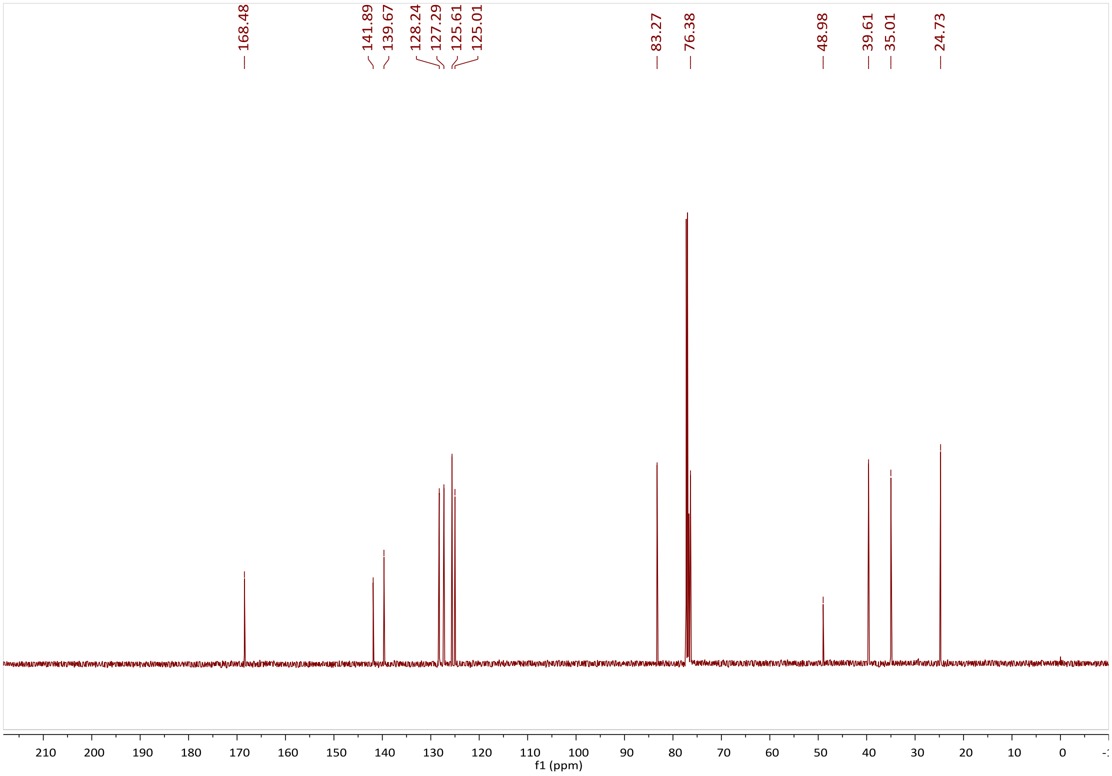
168.48
141.89
139.67
128.24
127.29
125.61
125.01
83.27
76.38
48.98
39.61
35.01
24.73
f1 (ppm)

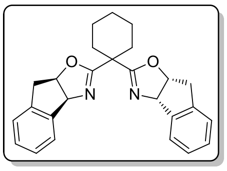
O
N
O
N

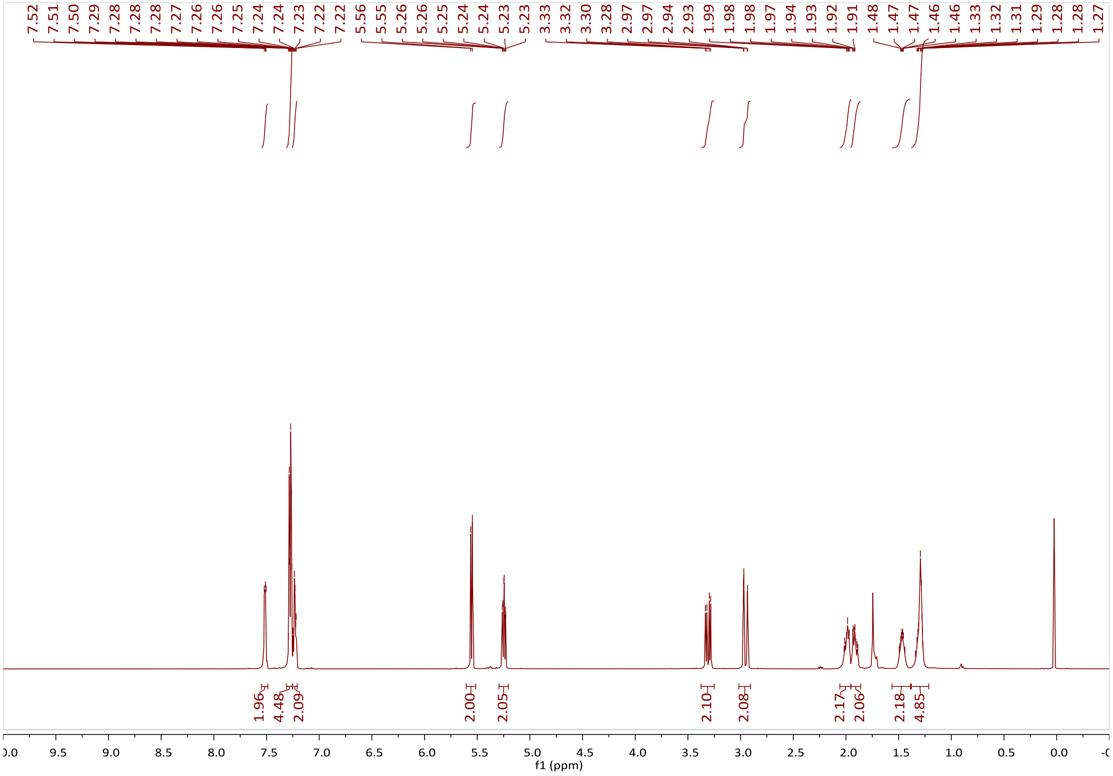
7.52
7.51
7.50
7.29
7.28
7.28
7.28
7.27
7.26
7.26
7.25
7.24
7.24
7.23
7.22
7.22
5.56
5.55
5.26
5.26
5.25
5.24
5.24
5.23
3.33
3.32
3.30
3.28
2.97
2.97
2.94
2.93
1.99
1.98
1.98
1.97
1.94
1.93
1.92
1.91
1.48
1.47
1.47
1.46
1.46
1.33
1.32
1.31
1.29
1.28
1.28
1.27
1.96
4.48
2.09
2.00
2.05
2.10
2.08
2.17
2.06
2.18
4.85
f1 (ppm)

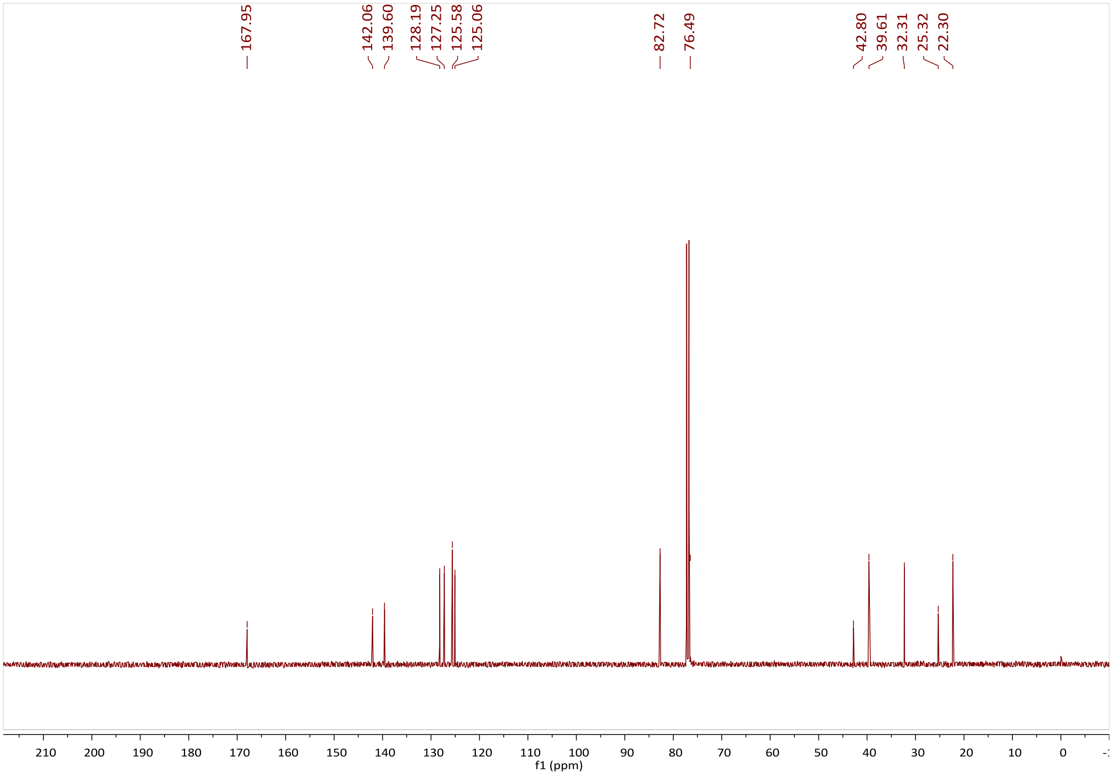
167.95
142.06
139.60
128.19
127.25
125.58
125.06
82.72
76.49
42.80
39.61
32.31
25.32
22.30
f1 (ppm)

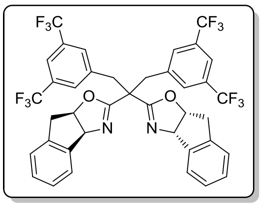
F3C
CF3
F3C
CF3
O
N
O
N

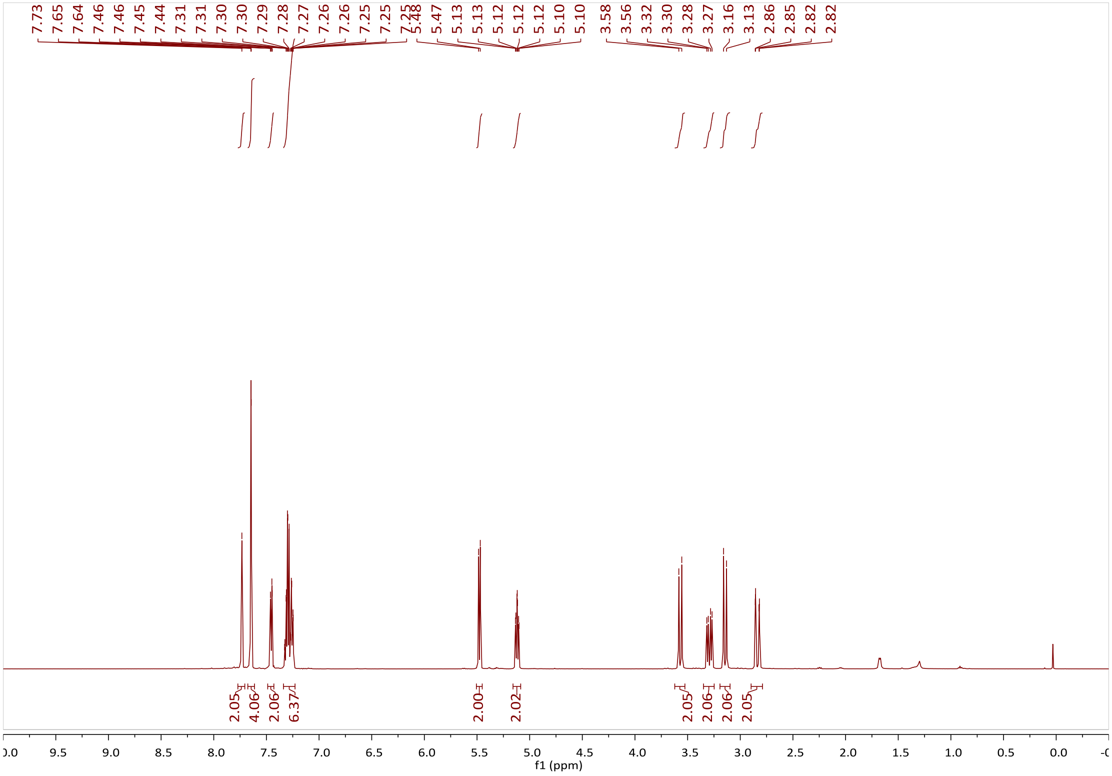
7.73
7.65
7.64
7.46
7.46
7.45
7.44
7.31
7.31
7.30
7.30
7.29
7.28
7.27
7.26
7.26
7.25
7.25
7.25
5.48
5.47
5.13
5.13
5.12
5.12
5.12
5.10
5.10
3.58
3.56
3.32
3.30
3.28
3.27
3.16
3.13
2.86
2.85
2.82
2.82
2.05
4.06
2.06
6.37
2.00
2.02
2.05
2.06
2.06
2.05
f1 (ppm)

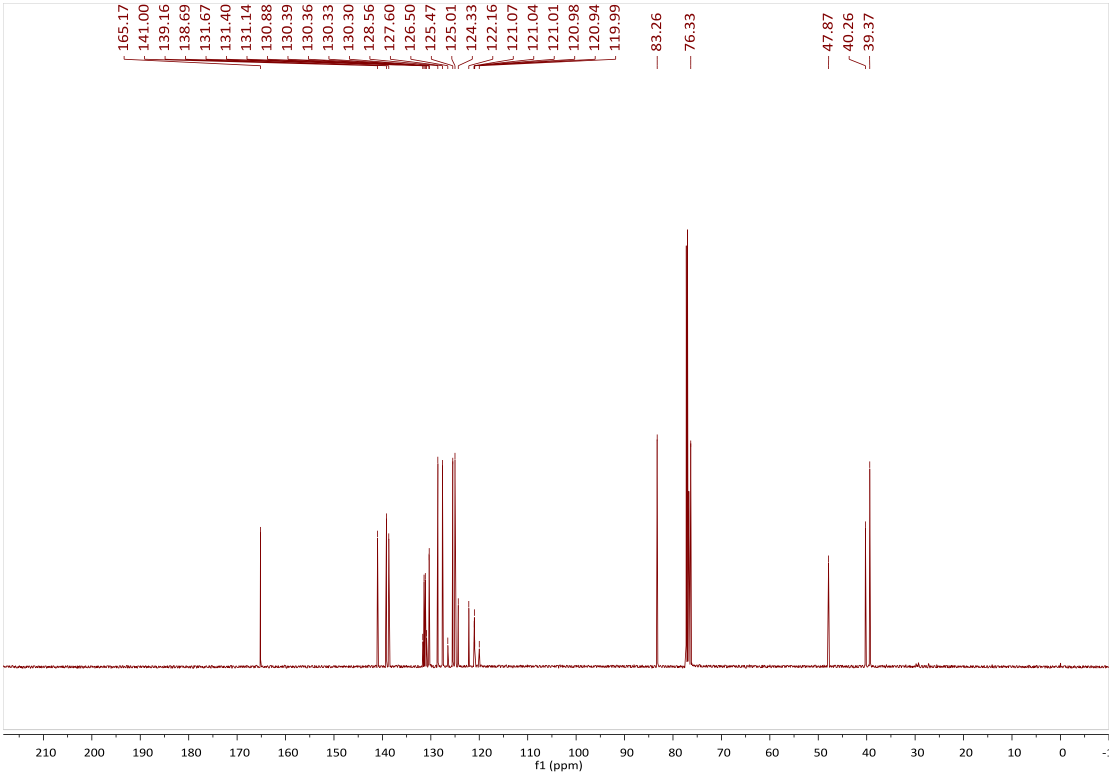
165.17
141.00
139.16
138.69
131.67
131.40
131.14
130.88
130.39
130.36
130.33
130.30
128.56
127.60
126.50
125.47
125.01
124.33
122.16
121.07
121.04
121.01
120.98
120.94
119.99
83.26
76.33
47.87
40.26
39.37
f1 (ppm)

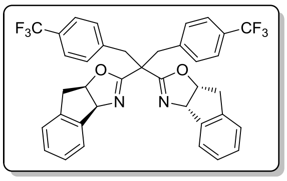
F3C
CF3
O
N
O
N

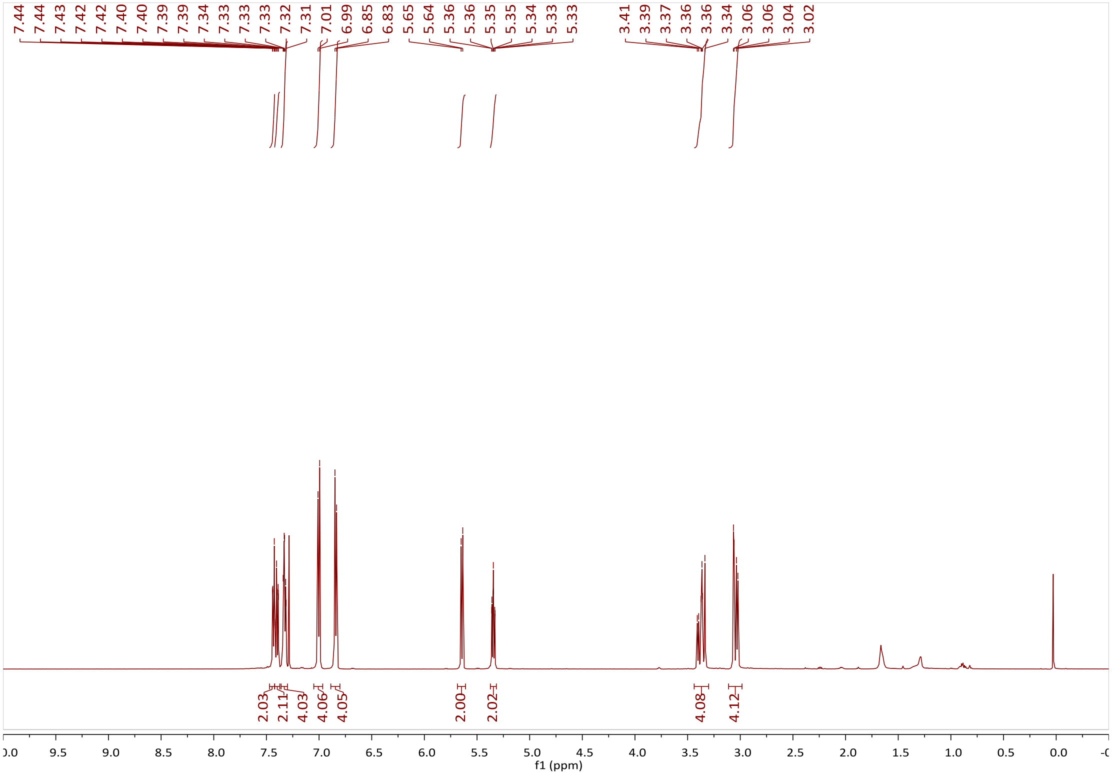
7.44
7.44
7.43
7.42
7.42
7.40
7.40
7.39
7.39
7.34
7.33
7.33
7.33
7.32
7.31
7.01
6.99
6.85
6.83
5.65
5.64
5.36
5.36
5.35
5.35
5.34
5.33
5.33
3.41
3.39
3.37
3.36
3.36
3.34
3.06
3.06
3.04
3.02
2.03
2.11
4.03
4.06
4.05
2.00
2.02
4.08
4.12
f1 (ppm)

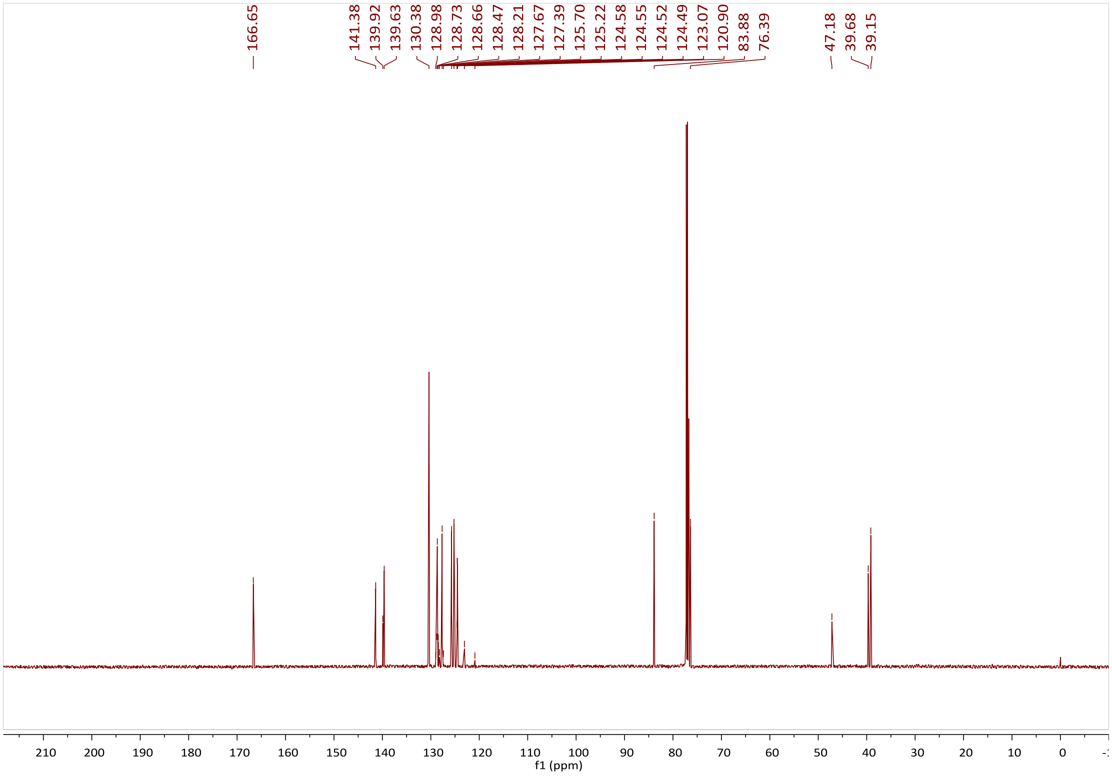
166.65
141.38
139.92
139.63
130.38
128.98
128.73
128.66
128.47
128.21
127.67
127.39
125.70
125.22
124.58
124.55
124.52
124.49
123.07
120.90
83.88
76.39
47.18
39.68
39.15
f1 (ppm)

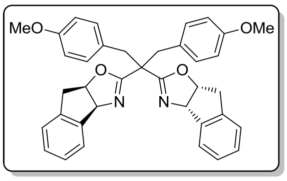
MeO
OMe
O
N
O
N

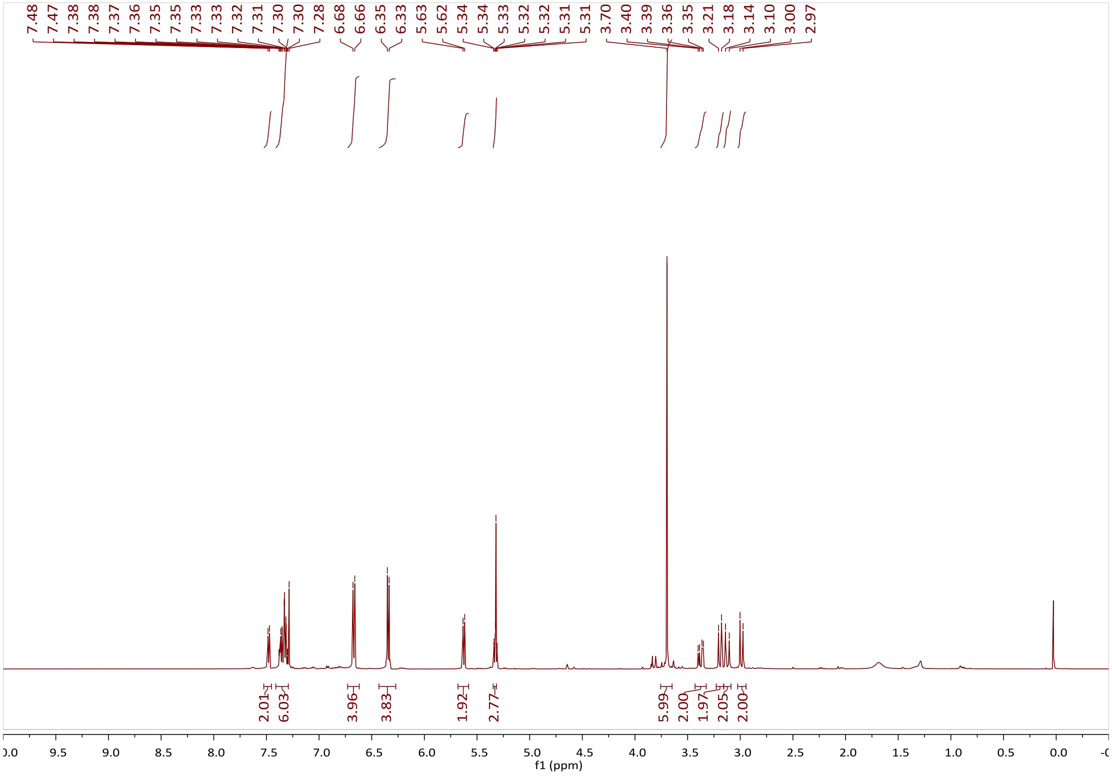
7.48
7.47
7.38
7.38
7.37
7.36
7.35
7.35
7.33
7.33
7.32
7.31
7.30
7.30
7.28
6.68
6.66
6.35
6.33
5.63
5.62
5.34
5.34
5.33
5.32
5.32
5.31
5.31
3.70
3.40
3.39
3.36
3.35
3.21
3.18
3.14
3.10
3.00
2.97
2.01
6.03
3.96
3.83
1.92
2.77
5.99
2.00
1.97
2.05
2.00
f1 (ppm)

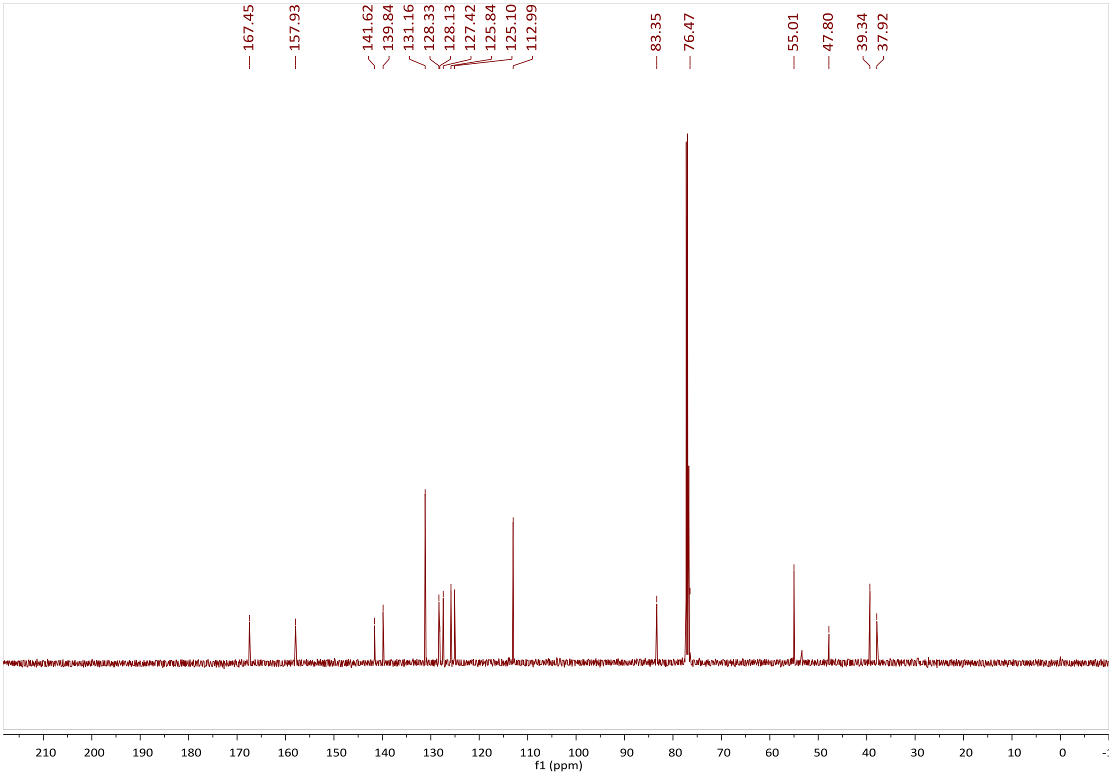
167.45
157.93
141.62
139.84
131.16
128.33
128.13
127.42
125.84
125.10
112.99
83.35
76.47
55.01
47.80
39.34
37.92
f1 (ppm)

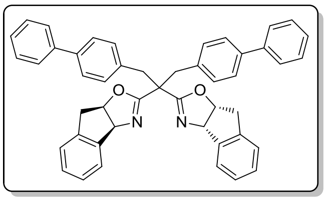

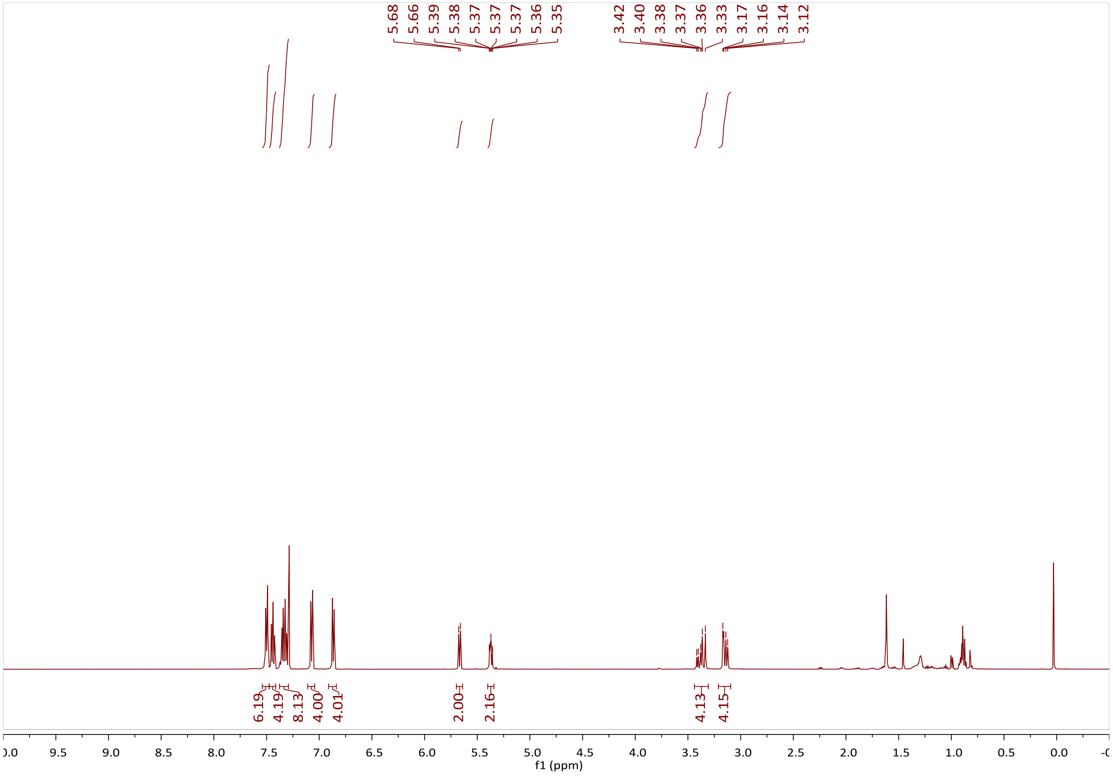
5.68
5.66
5.39
5.38
5.37
5.37
5.37
5.36
5.35
3.42
3.40
3.38
3.37
3.36
3.33
3.17
3.16
3.14
3.12
6.19
4.19
8.13
4.00
4.01
2.00
2.16
4.13
4.15
f1 (ppm)

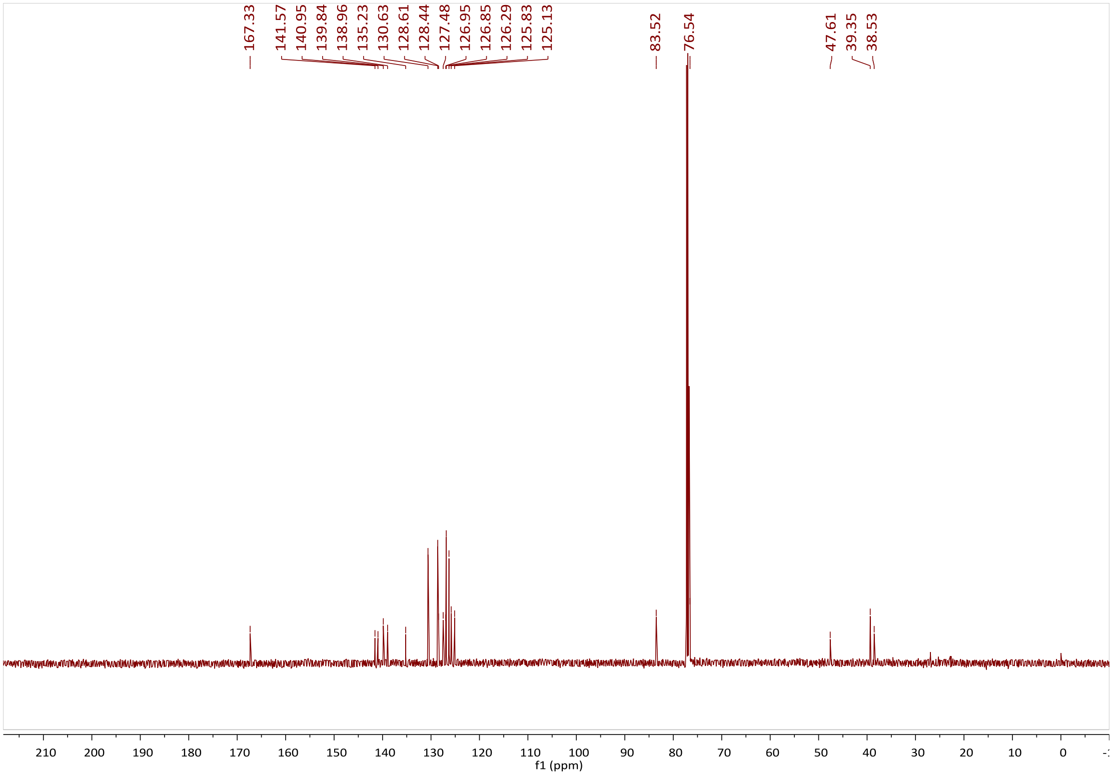
167.33
141.57
140.95
139.84
138.96
135.23
130.63
128.61
128.44
127.48
126.95
126.85
126.29
125.83
125.13
83.52
76.54
47.61
39.35
38.53
f1 (ppm)

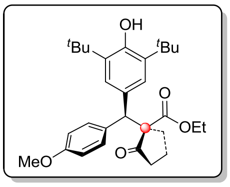

OH
tBu
tBu
O
OEt
O
MeO

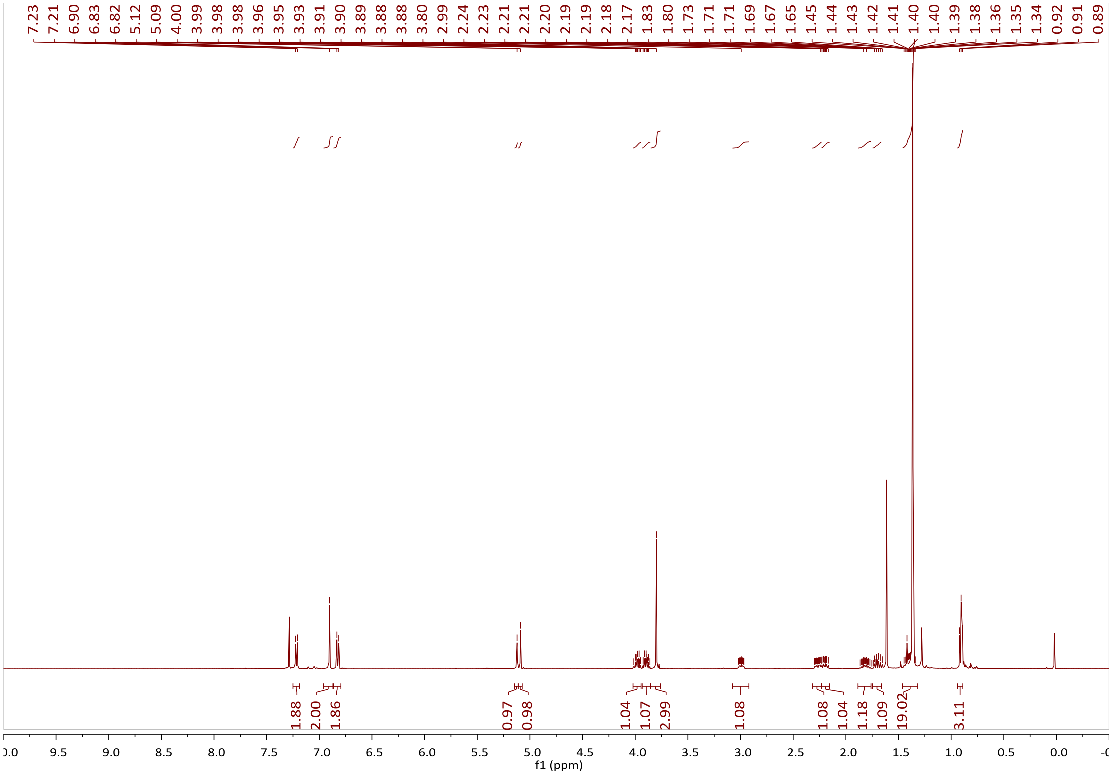

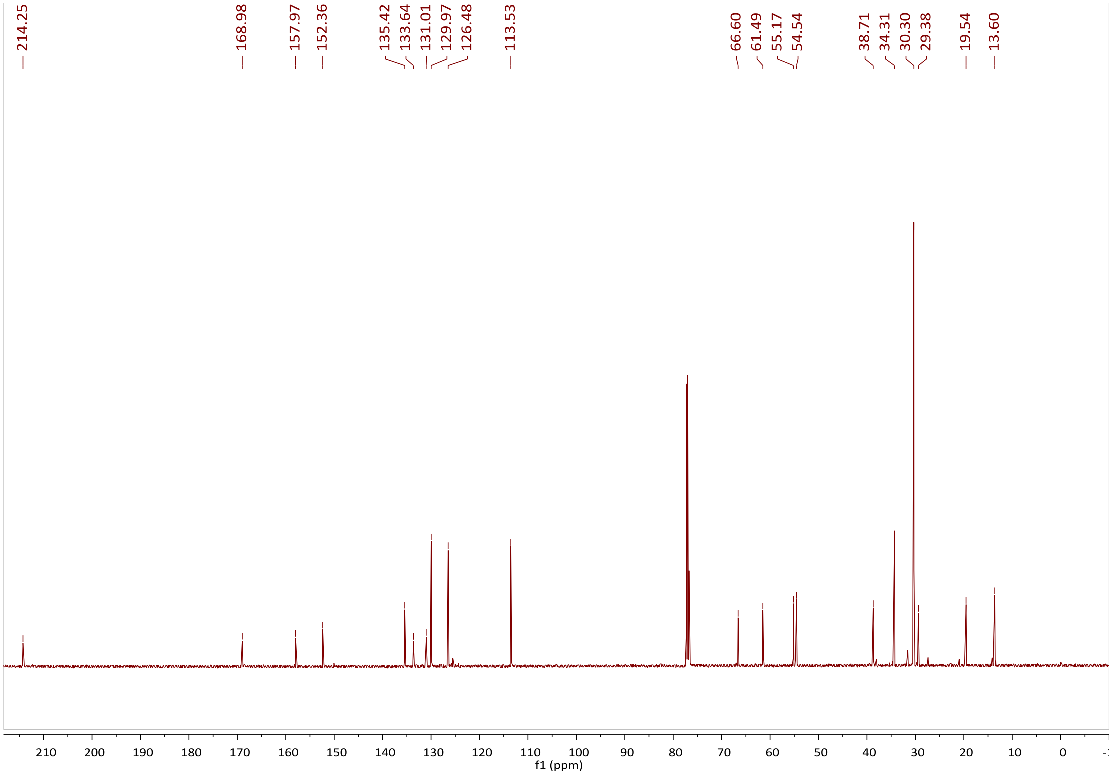

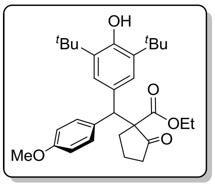

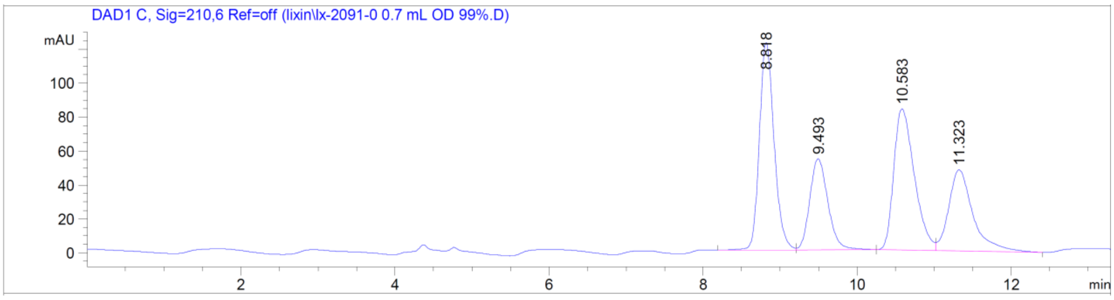

Peak #	RetTime [min]	Type	Width [min]	Area [mAU*s]	Height [mAU]	Area %
1	8.818	BV	0.2025	1620.01392	122.35436	31.8096
2	9.493	VB	0.2531	878.31750	53.61618	17.2461
3	10.583	BV	0.2845	1544.20459	83.21940	30.3210
4	11.323	VB	0.3262	1050.31299	47.83245	20.6233

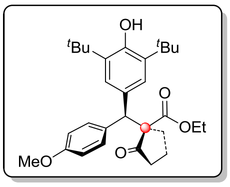

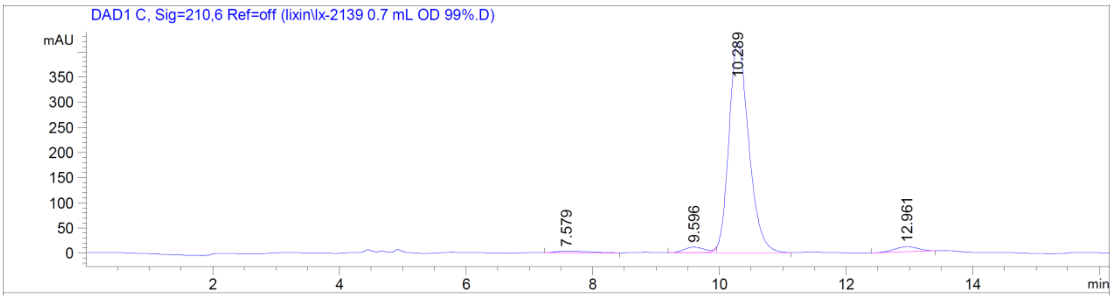

Peak #	RetTime [min]	Type	Width [min]	Area [mAU*s]	Height [mAU]	Area %
1	7.579	BB	0.4491	109.58862	3.18734	1.1302
2	9.596	BV E	0.3437	244.46278	11.06776	2.5212
3	10.289	VB R	0.3337	9080.55176	417.53647	93.6514
4	12.961	BB	0.4150	261.52100	10.00333	2.6972

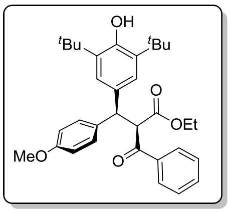
OH
tBu
tBu
O
OEt
MeO
O

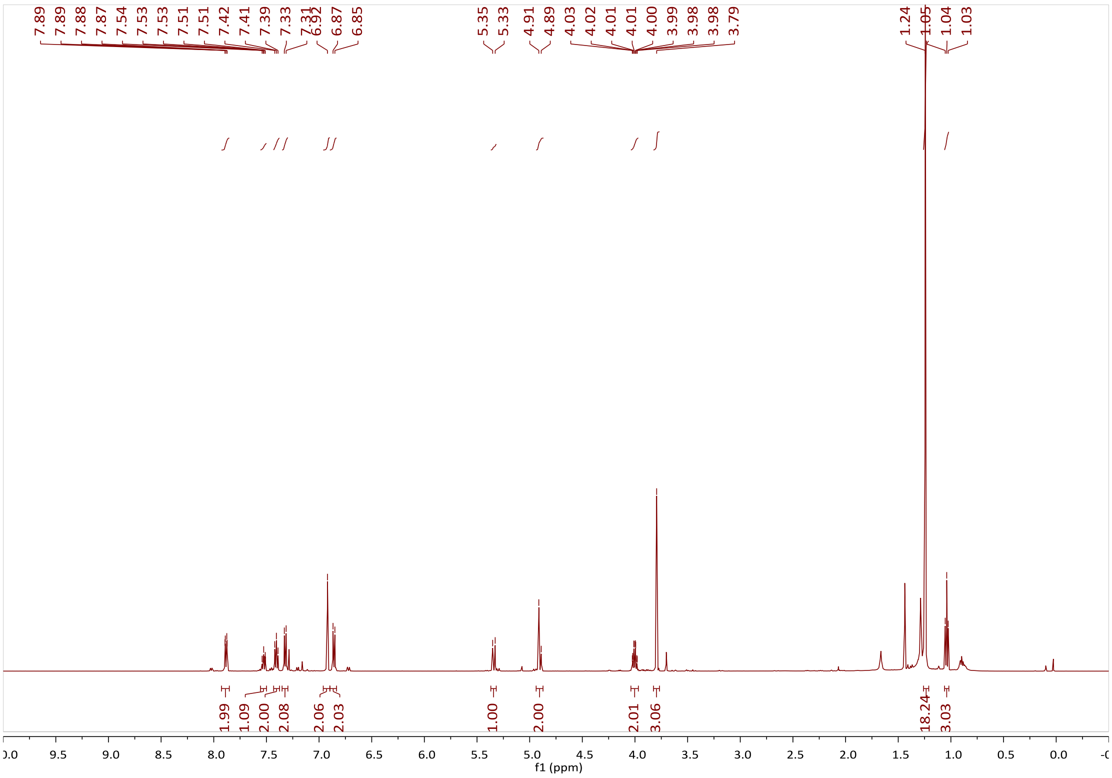

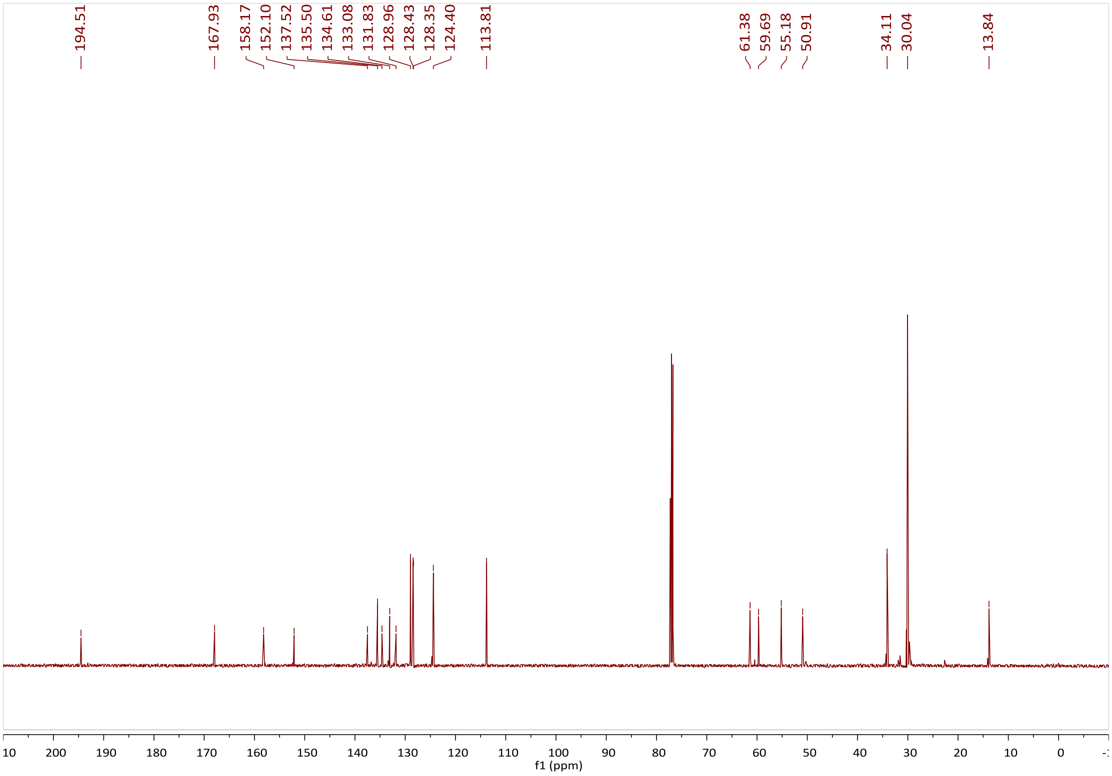

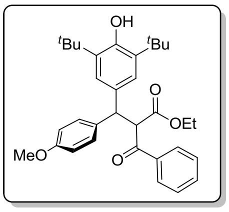

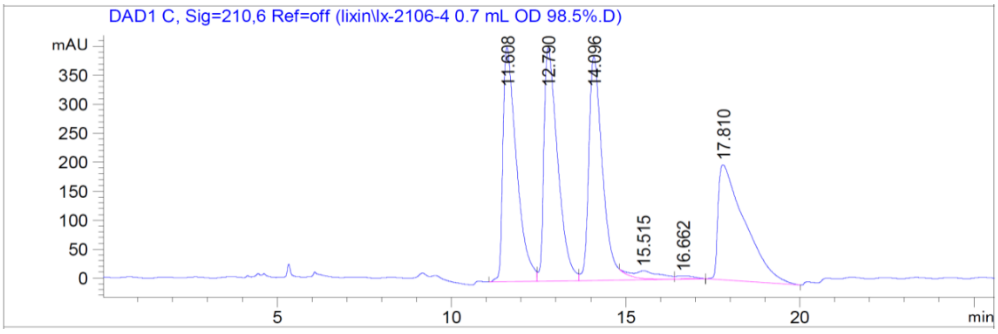

Peak #	RetTime [min]	Type	Width [min]	Area [mAU*s]	Height [mAU]	Area %
1	11.608	BV	0.4073	1.09948e4	401.74408	24.8412
2	12.790	VV	0.4259	1.10621e4	400.88791	24.9932
3	14.096	VV R	0.4219	1.07713e4	385.62802	24.3363
4	15.515	VV E	0.6898	688.06812	13.16368	1.5546
5	16.662	VB E	0.5820	181.23712	5.10722	0.4095
6	17.810	BB	0.7394	1.05628e4	198.95833	23.8652

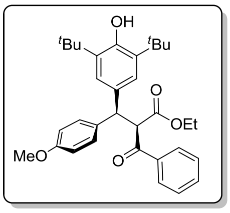

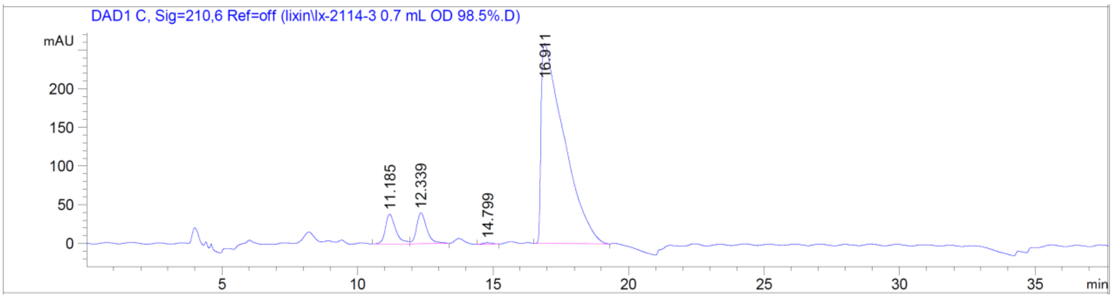

Peak #	RetTime [min]	Type	Width [min]	Area [mAU*s]	Height [mAU]	Area %
1	11.185	BV	0.3976	1013.98352	38.20911	5.8679
2	12.339	VB	0.3923	1026.96887	39.63898	5.9430
3	14.799	BB	0.3235	46.98057	1.96111	0.2719
4	16.911	BB	0.7867	1.51923e4	258.84998	87.9172

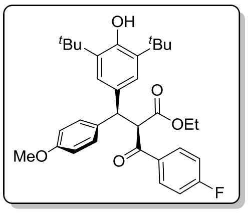
OH
tBu
tBu
O
OEt
MeO
O
F

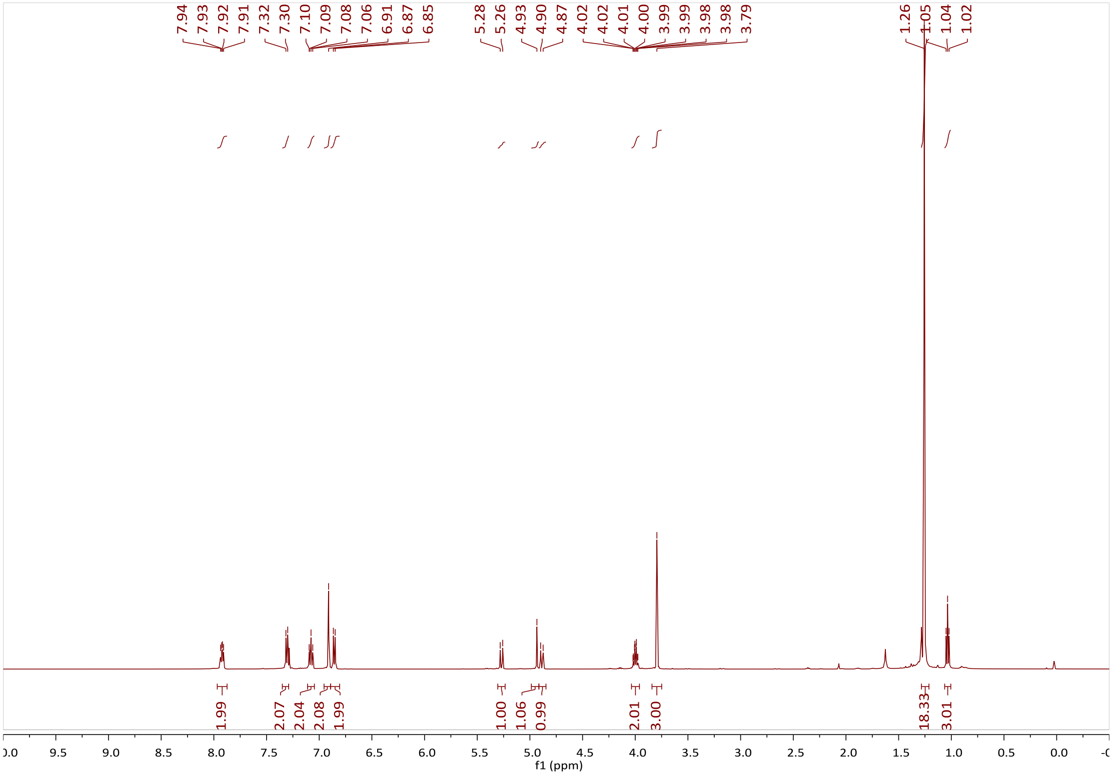
7.94
7.93
7.92
7.91
7.32
7.30
7.10
7.09
7.08
7.06
6.91
6.87
6.85
5.28
5.26
4.93
4.90
4.87
4.02
4.02
4.01
4.00
3.99
3.99
3.98
3.98
3.79
1.26
1.05
1.04
1.02
1.99
2.07
2.04
2.08
1.99
1.00
1.06
0.99
2.01
3.00
18.33
3.01
f1 (ppm)

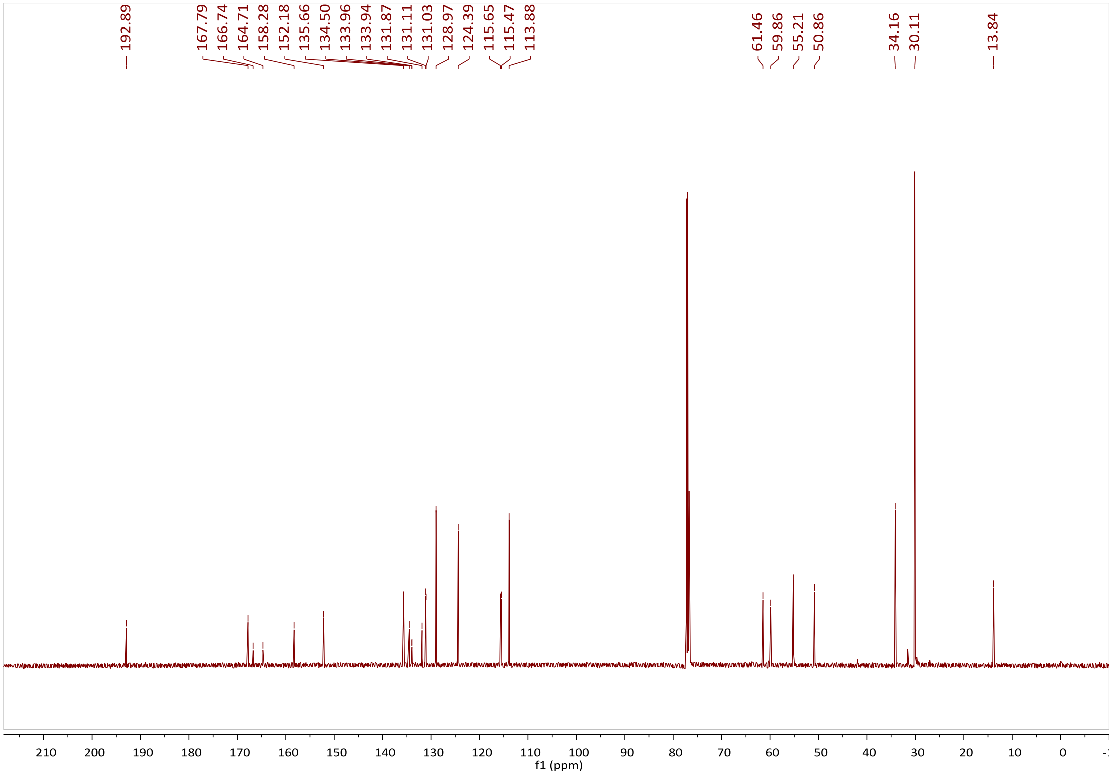
192.89
167.79
166.74
164.71
158.28
152.18
135.66
134.50
133.96
133.94
131.87
131.11
131.03
128.97
124.39
115.65
115.47
113.88
61.46
59.86
55.21
50.86
34.16
30.11
13.84
f1 (ppm)

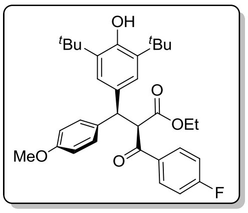
OH
tBu
tBu
O
OEt
MeO
O
F

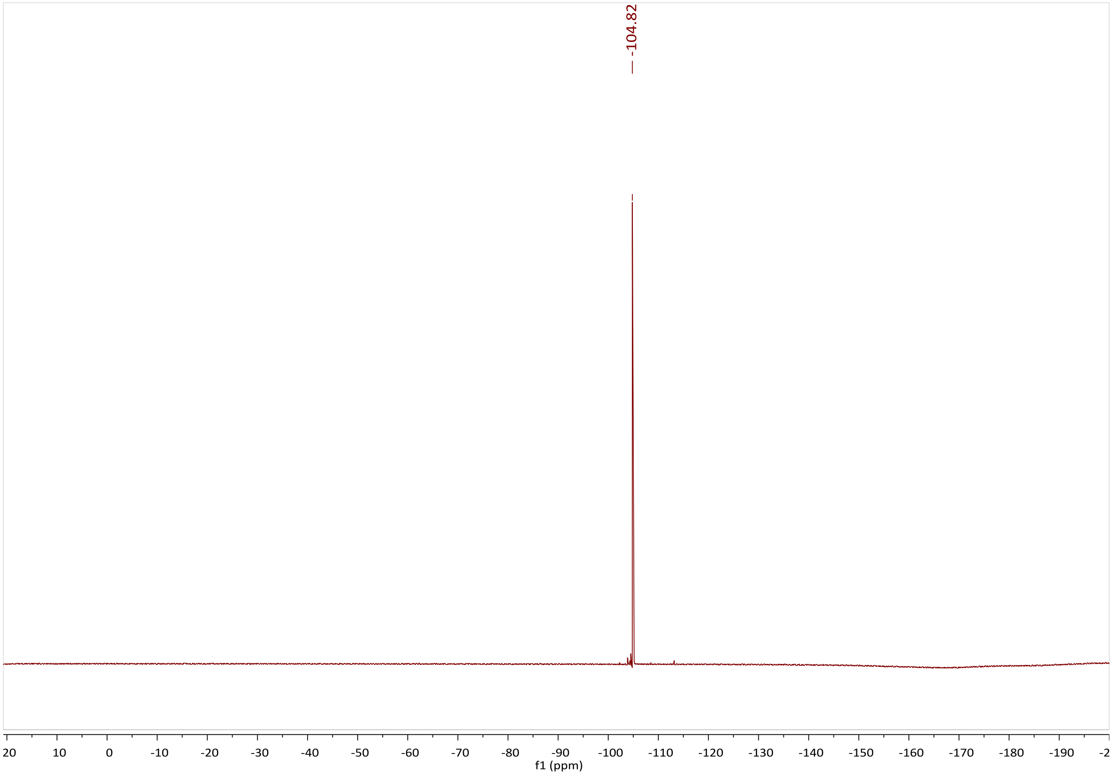
-104.82
20
10
0
-10
-20
-30
-40
-50
-60
-70
-80
-90
-100
-110
-120
-130
-140
-150
-160
-170
-180
-190
f1 (ppm)

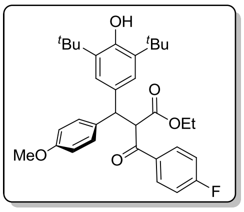

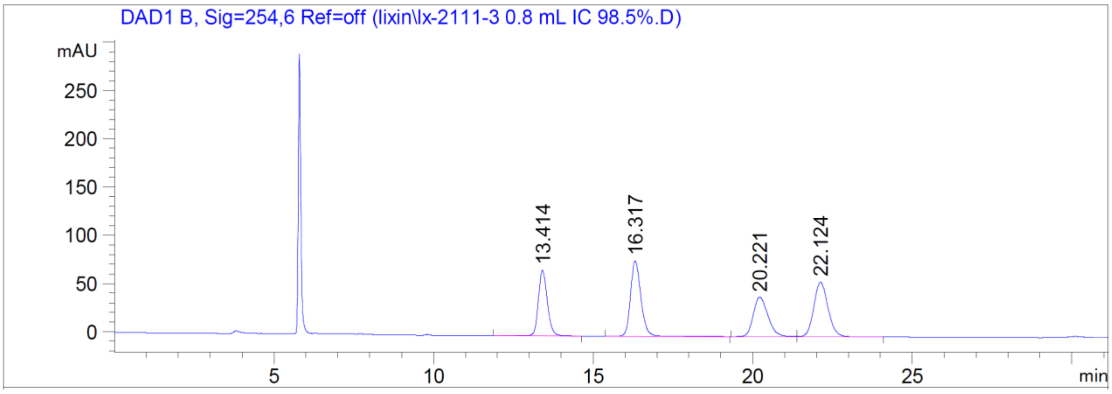

Peak #	RetTime [min]	Type	Width [min]	Area [mAU*s]	Height [mAU]	Area %
1	13.414	VB R	0.3087	1329.76343	67.76629	21.5803
2	16.317	BV R	0.3436	1771.35400	78.19429	28.7467
3	20.221	BV	0.4935	1307.29395	40.83018	21.2156
4	22.124	VB	0.4814	1753.53357	56.60907	28.4575

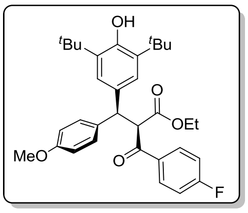

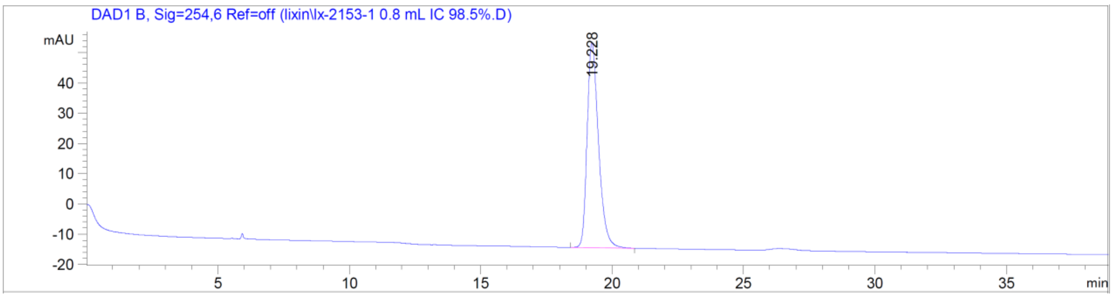

Peak #	RetTime [min]	Type	Width [min]	Area [mAU*s]	Height [mAU]	Area %
1	19.228	BB	0.4605	2040.50671	67.91088	100.0000

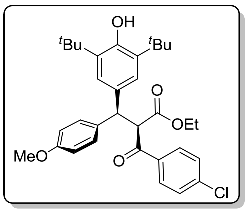
OH
tBu
tBu
O
OEt
MeO
O
Cl

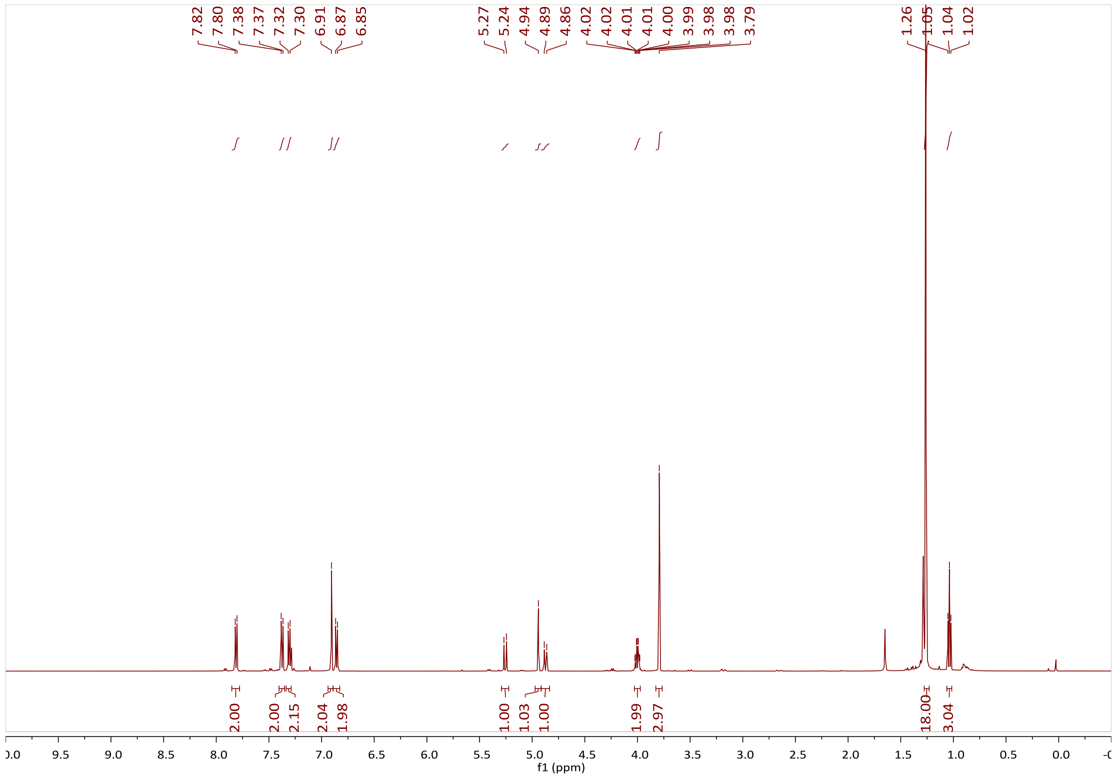

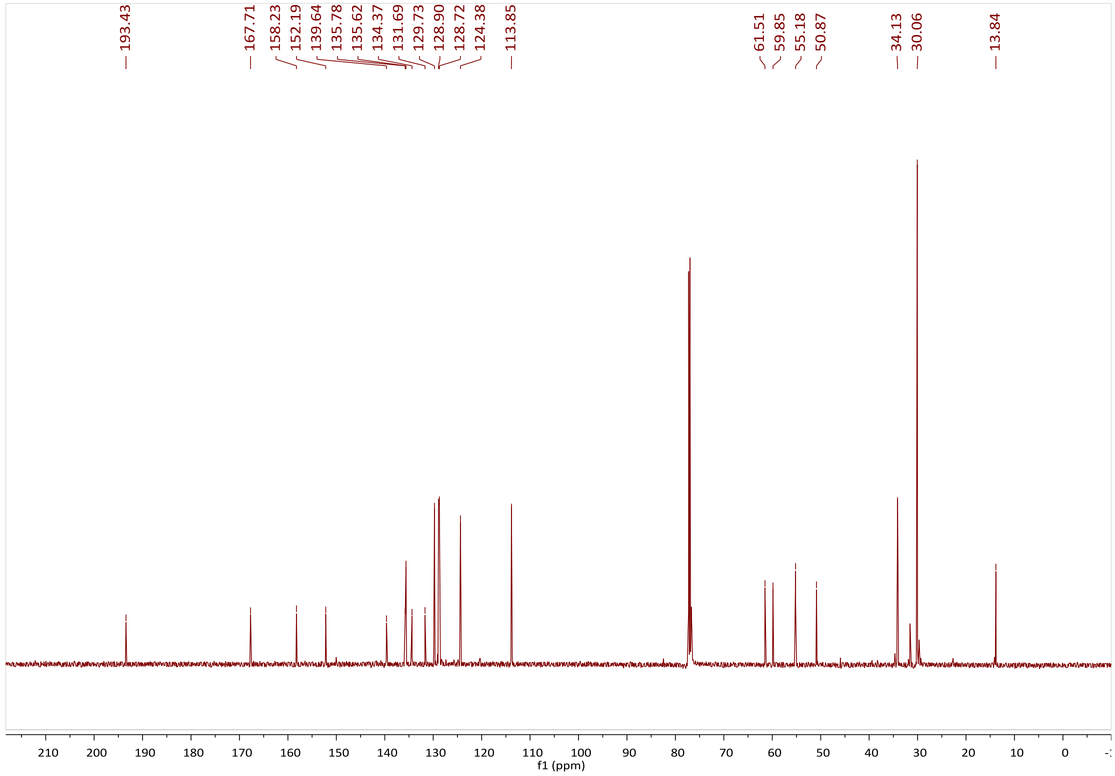

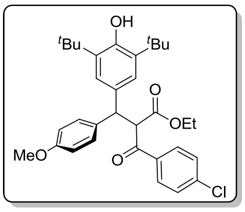

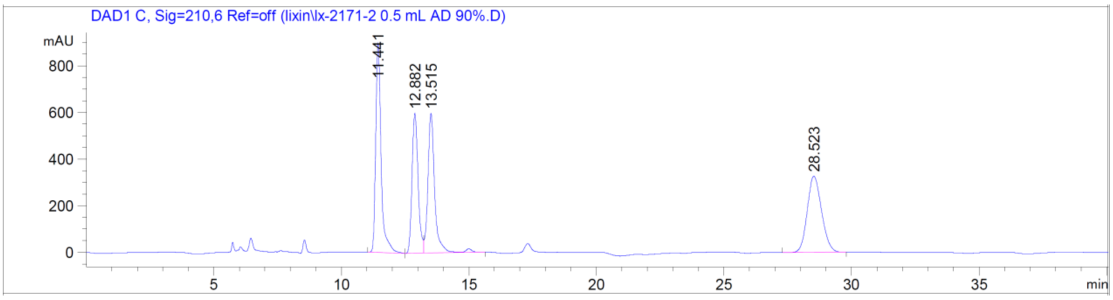

Peak #	RetTime [min]	Type	Width [min]	Area [mAU*s]	Height [mAU]	Area %
1	11.441	BB	0.2211	1.31686e4	897.81628	28.0297
2	12.882	BV	0.2519	9863.95508	599.63757	20.9956
3	13.515	VV R	0.2737	1.12122e4	599.60980	23.8654
4	28.523	BB	0.6045	1.27362e4	326.91876	27.1092

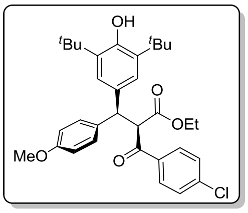

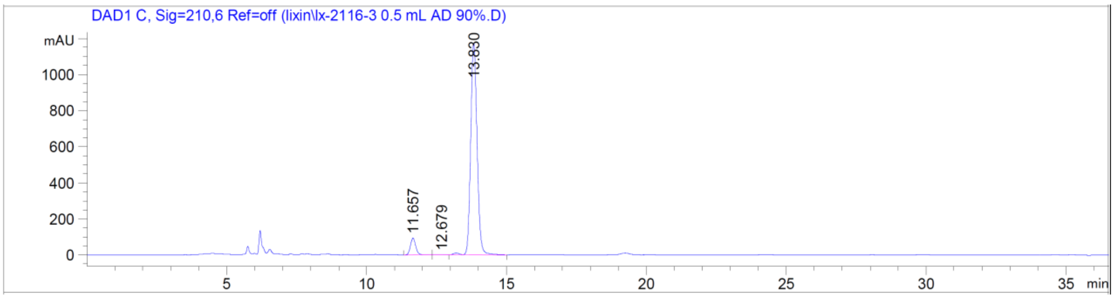

Peak #	RetTime [min]	Type	Width [min]	Area [mAU*s]	Height [mAU]	Area %
1	11.657	BB	0.2038	1222.91516	92.77933	6.1172
2	12.679	BV E	0.2358	23.33870	1.37679	0.1167
3	13.830	VB R	0.2468	1.87453e4	1174.19495	93.7661

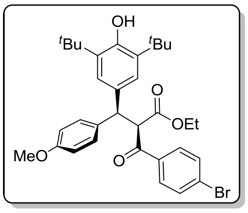
OH
tBu
tBu
O
OEt
MeO
O
Br

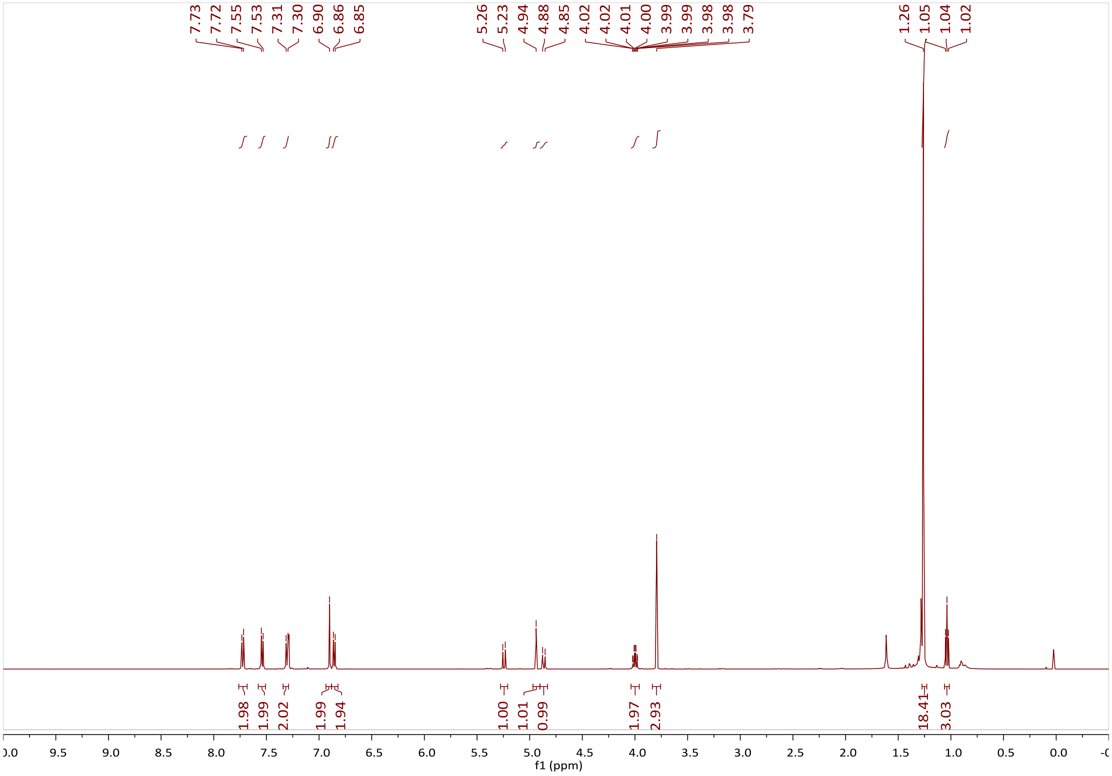
7.73
7.72
7.55
7.53
7.31
7.30
6.90
6.86
6.85
5.26
5.23
4.94
4.88
4.85
4.02
4.02
4.01
4.00
3.99
3.99
3.98
3.98
3.79
1.26
1.05
1.04
1.02
1.98
1.99
2.02
1.99
1.94
1.00
1.01
0.99
1.97
2.93
18.41
3.03
f1 (ppm)

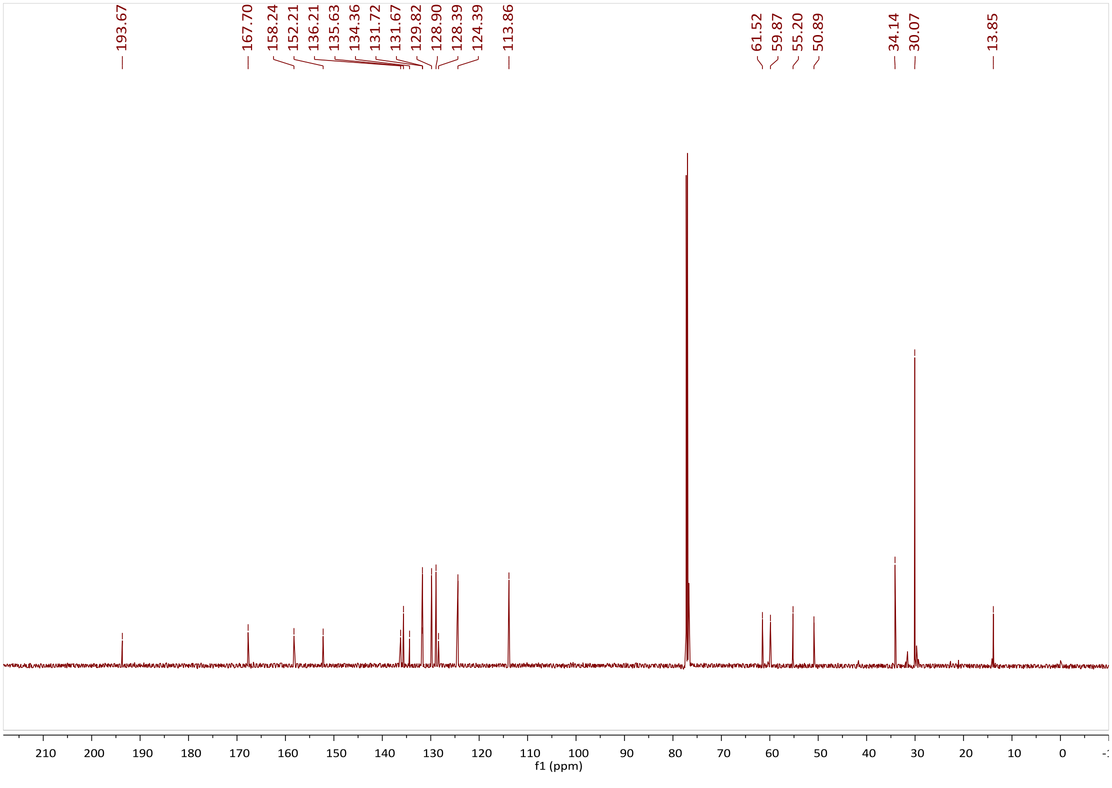
193.67
167.70
158.24
152.21
136.21
135.63
134.36
131.72
131.67
129.82
128.90
128.39
124.39
113.86
61.52
59.87
55.20
50.89
34.14
30.07
13.85
f1 (ppm)

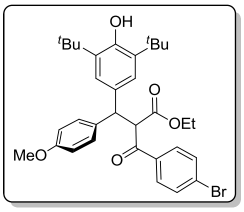

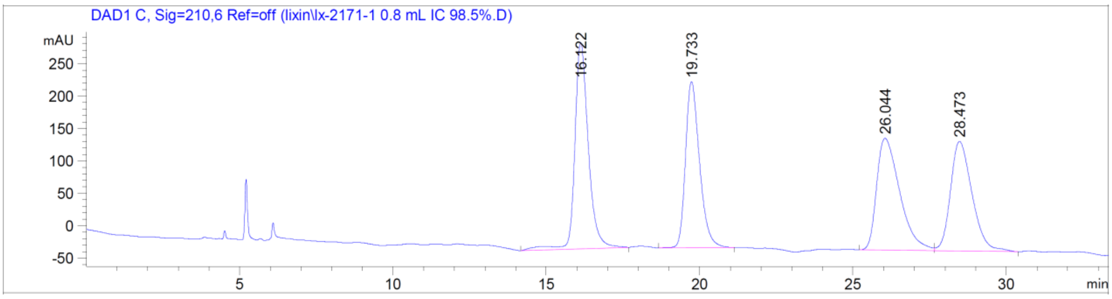

Peak #	RetTime [min]	Type	Width [min]	Area [mAU*s]	Height [mAU]	Area %
1	16.122	BB	0.4645	9760.11328	317.63513	27.6911
2	19.733	BB	0.4853	8157.81201	256.30795	23.1451
3	26.044	BV	0.8182	9045.38574	172.38573	25.6633
4	28.473	VB	0.7456	8283.14160	169.06358	23.5006

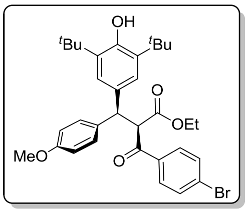

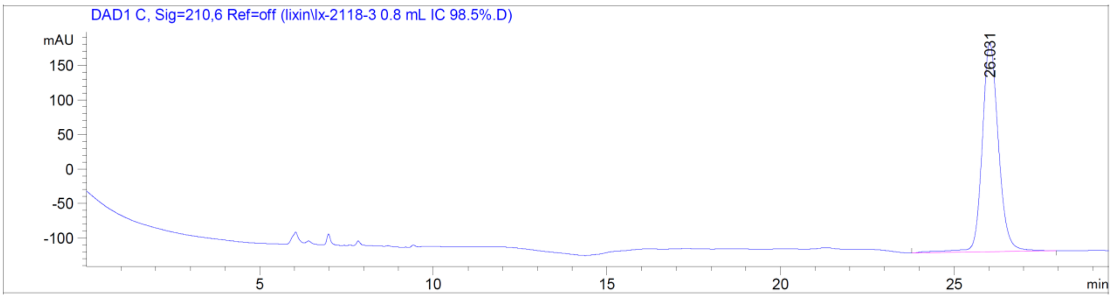

Peak #	RetTime [min]	Type	Width [min]	Area [mAU*s]	Height [mAU]	Area %
1	26.031	BB	0.4992	9851.46289	303.08517	100.0000

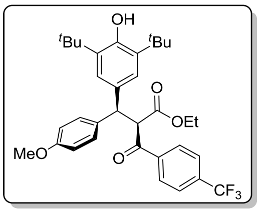
OH
tBu
tBu
O
OEt
MeO
O
CF3

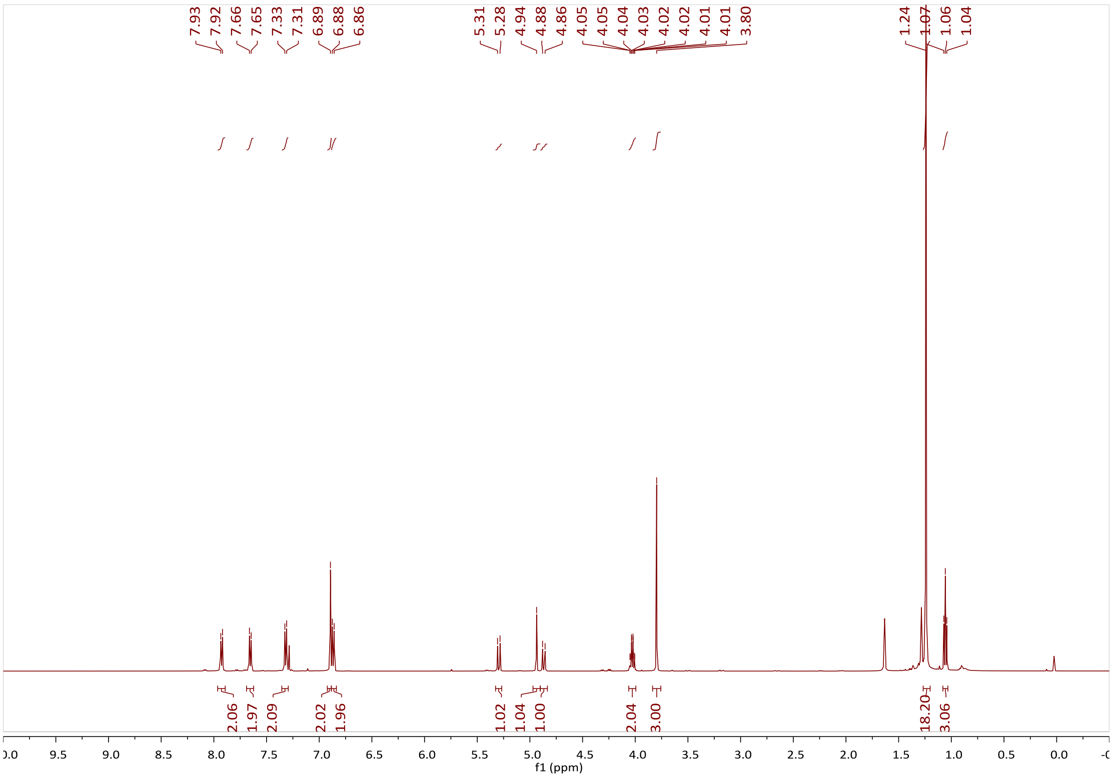

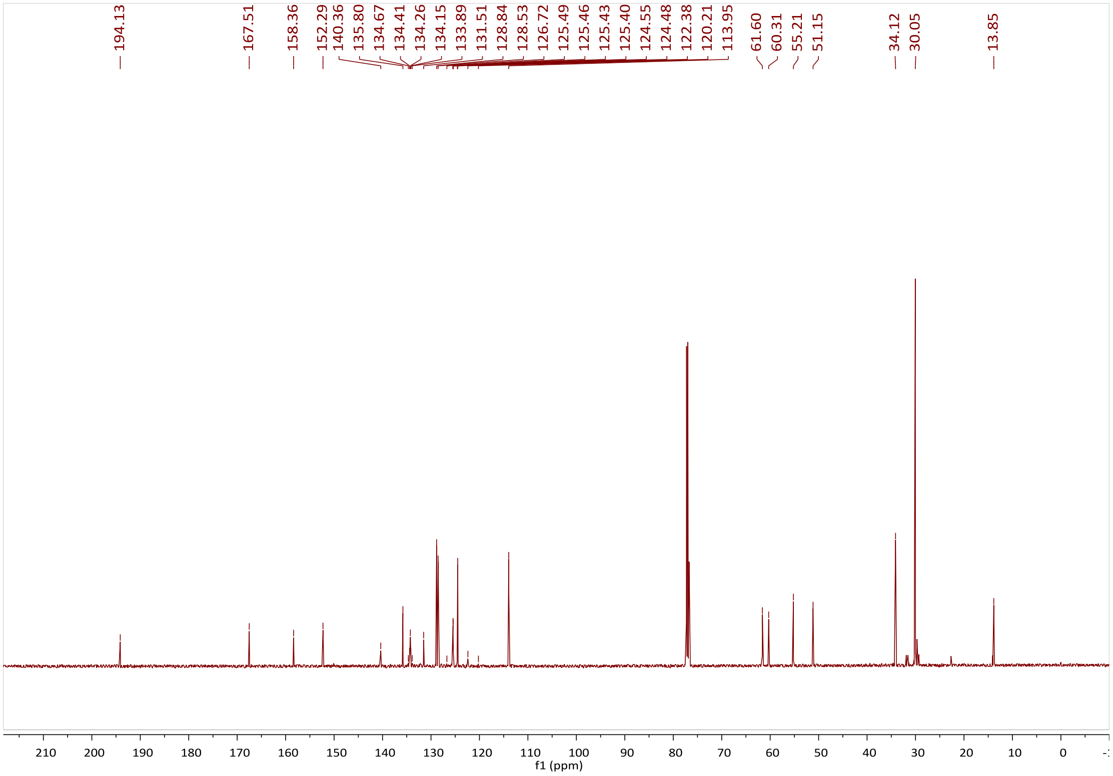

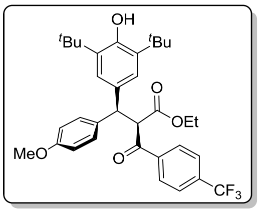

OH
ᵗBu
ᵗBu
O
OEt
MeO
O
CF3

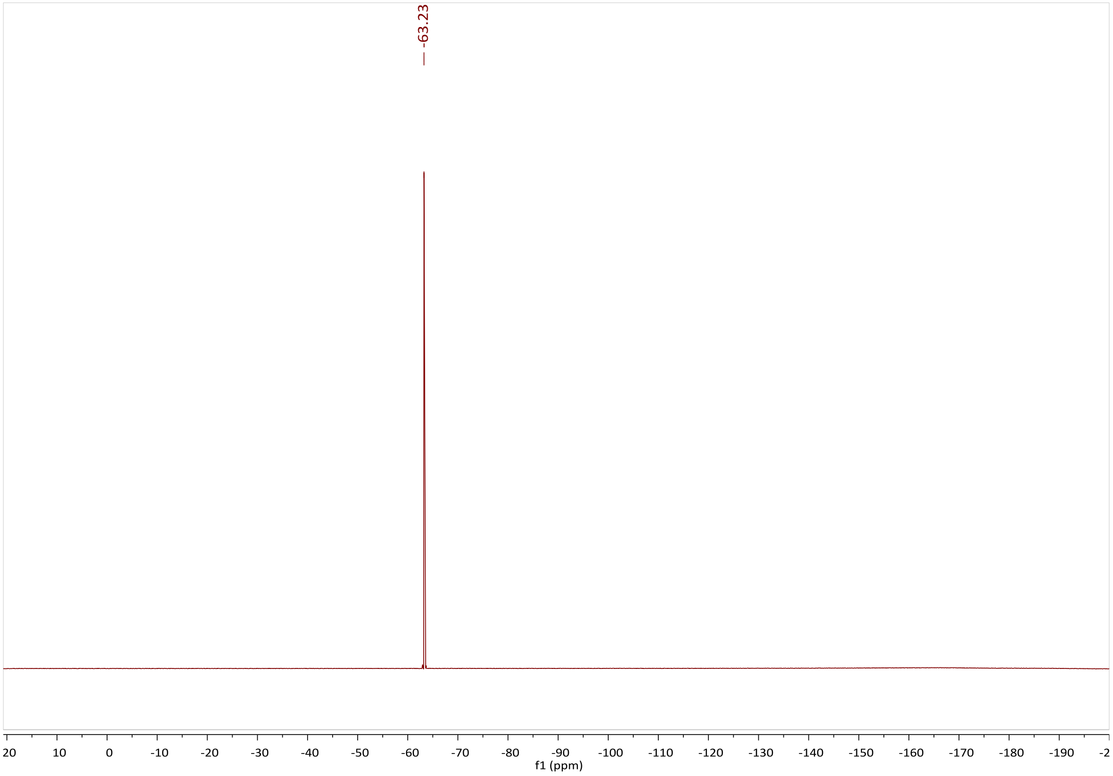

-63.23
20
10
0
-10
-20
-30
-40
-50
-60
-70
-80
-90
-100
-110
-120
-130
-140
-150
-160
-170
-180
-190
-2
f1 (ppm)

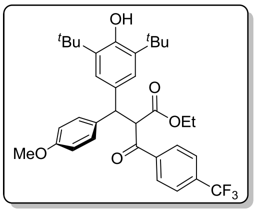

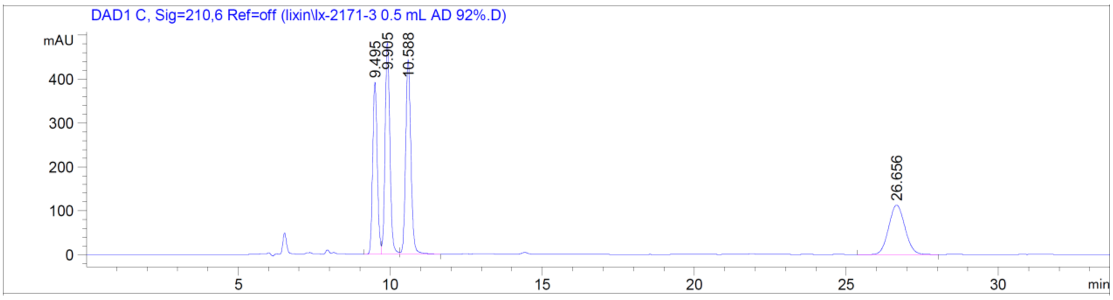

Peak #	RetTime [min]	Type	Width [min]	Area [mAU*s]	Height [mAU]	Area %
1	9.495	BV	0.1640	4093.63843	391.55130	21.6057
2	9.905	VV	0.1674	5266.91943	482.15863	27.7981
3	10.588	VB	0.1864	5344.69092	444.06894	28.2085
4	26.656	BB	0.5812	4241.81152	113.20433	22.3877

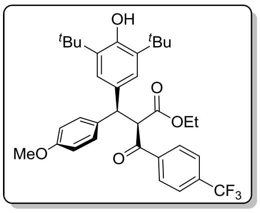

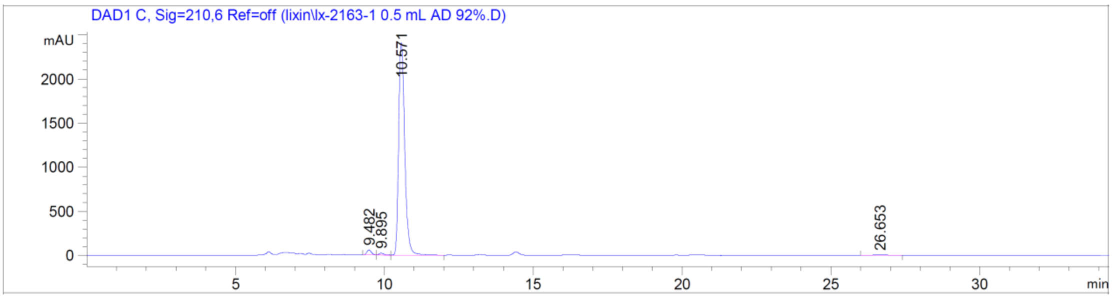

Peak #	RetTime [min]	Type	Width [min]	Area [mAU*s]	Height [mAU]	Area %
1	9.482	BV	0.1719	636.51666	57.15864	1.8025
2	9.895	VB	0.1766	249.69833	21.64906	0.7071
3	10.571	BB	0.2200	3.42337e4	2405.36792	96.9412
4	26.653	BB	0.4831	193.96458	5.67012	0.5493

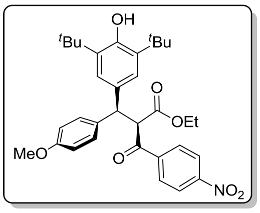
OH
tBu
tBu
O
OEt
MeO
O
NO2

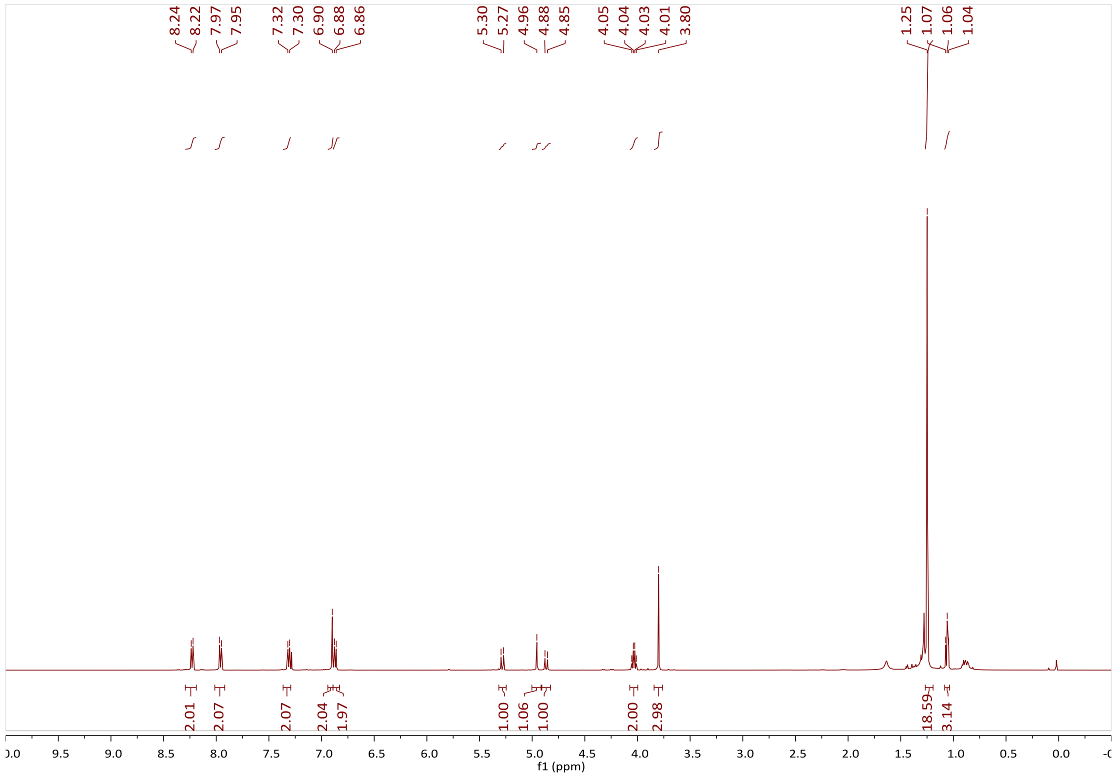

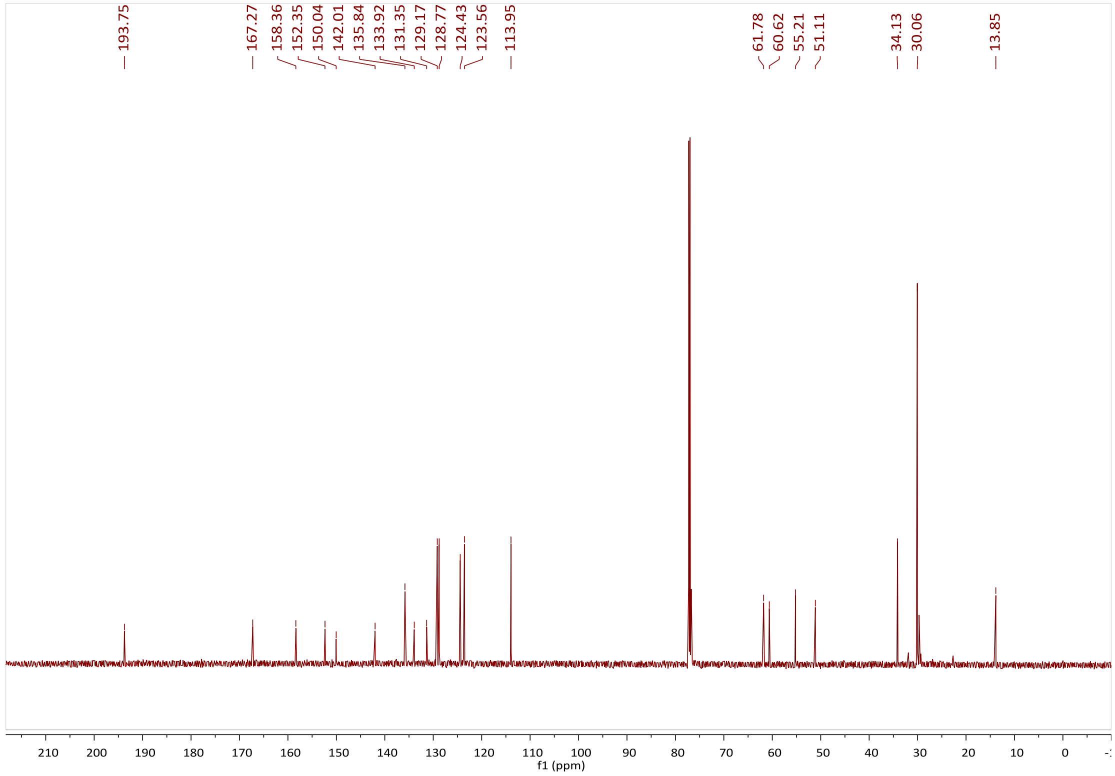

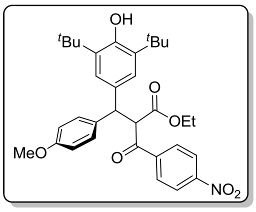

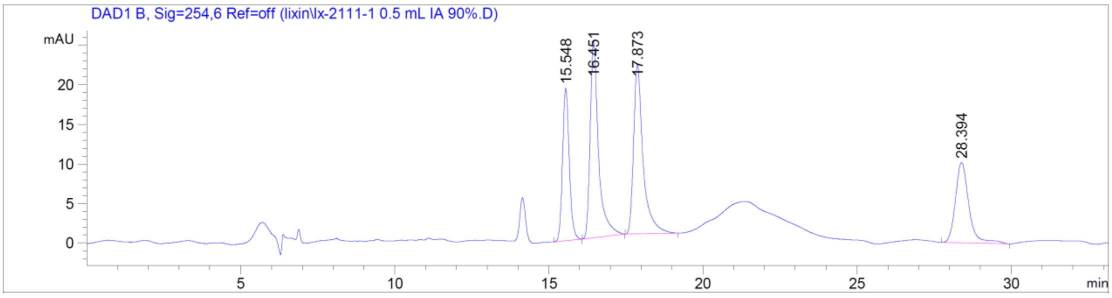

Peak #	RetTime [min]	Type	Width [min]	Area [mAU*s]	Height [mAU]	Area %
1	15.548	BB	0.2301	290.73160	19.25895	19.0532
2	16.451	BB	0.2778	468.02454	24.83919	30.6721
3	17.873	BB	0.3164	460.84305	21.46884	30.2015
4	28.394	BB	0.4460	306.29721	10.15161	20.0733

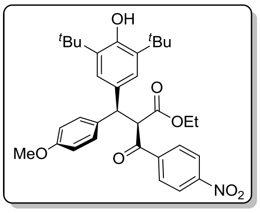

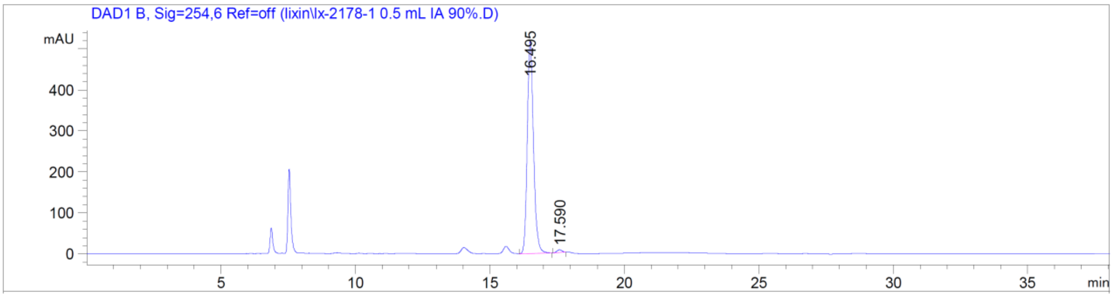

Peak #	RetTime [min]	Type	Width [min]	Area [mAU*s]	Height [mAU]	Area %
1	16.495	BB	0.2452	8312.83594	517.93628	99.0109
2	17.590	BB	0.2054	83.04434	6.39733	0.9891

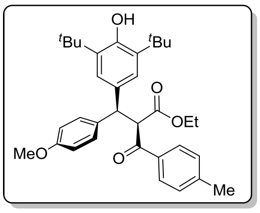
OH
tBu
tBu
O
OEt
MeO
O
Me

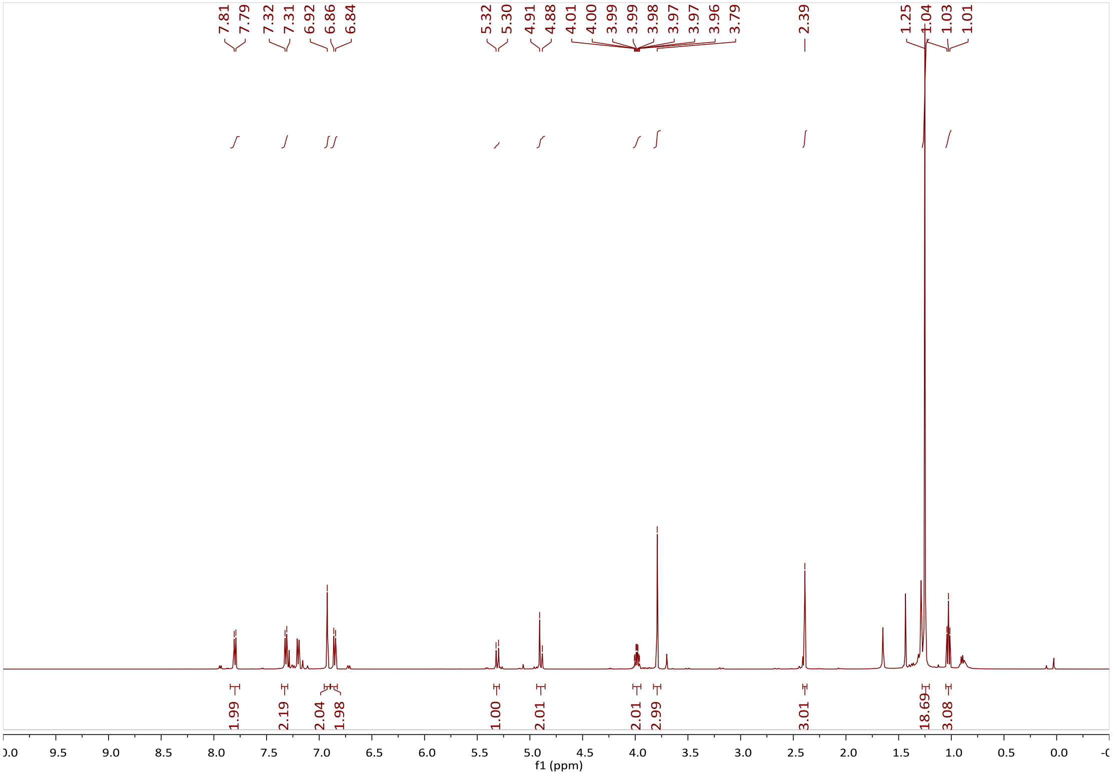
7.81
7.79
7.32
7.31
6.92
6.86
6.84
5.32
5.30
4.91
4.88
4.01
4.00
3.99
3.99
3.98
3.97
3.97
3.96
3.79
2.39
1.25
1.04
1.03
1.01
1.99
2.19
2.04
1.98
1.00
2.01
2.01
2.99
3.01
18.69
3.08
f1 (ppm)

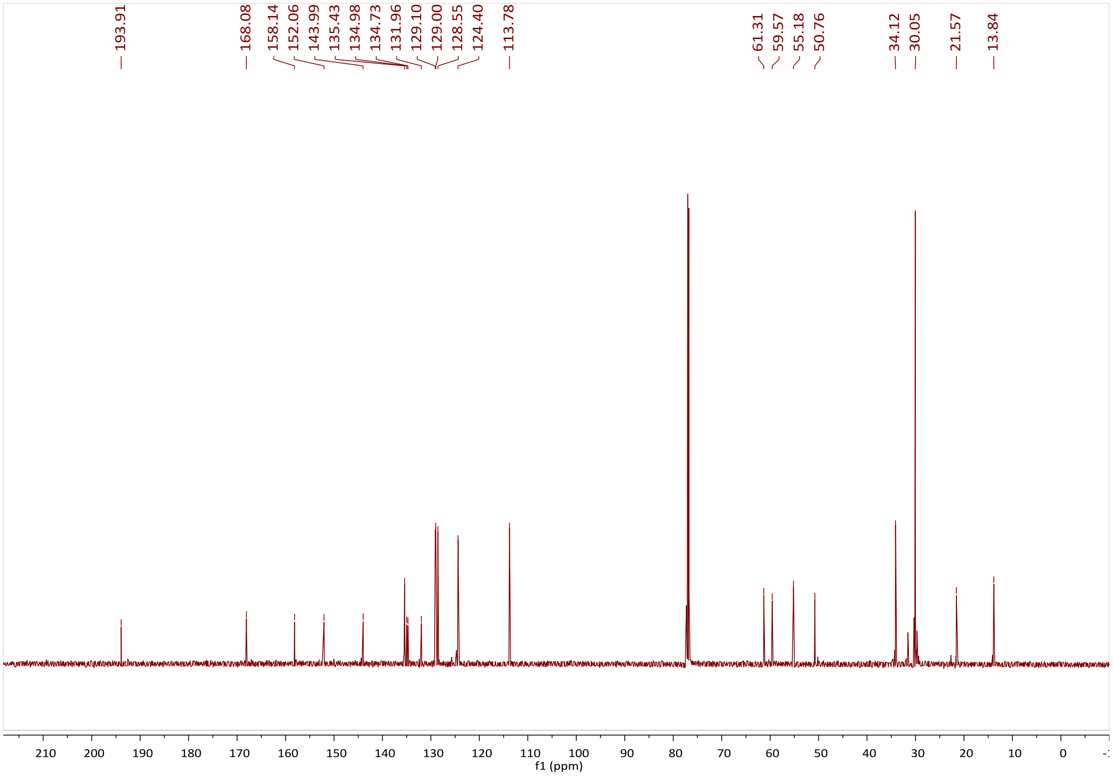
193.91
168.08
158.14
152.06
143.99
135.43
134.98
134.73
131.96
129.10
129.00
128.55
124.40
113.78
61.31
59.57
55.18
50.76
34.12
30.05
21.57
13.84
f1 (ppm)

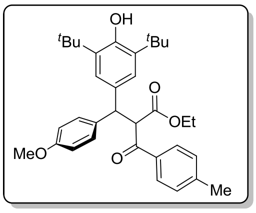

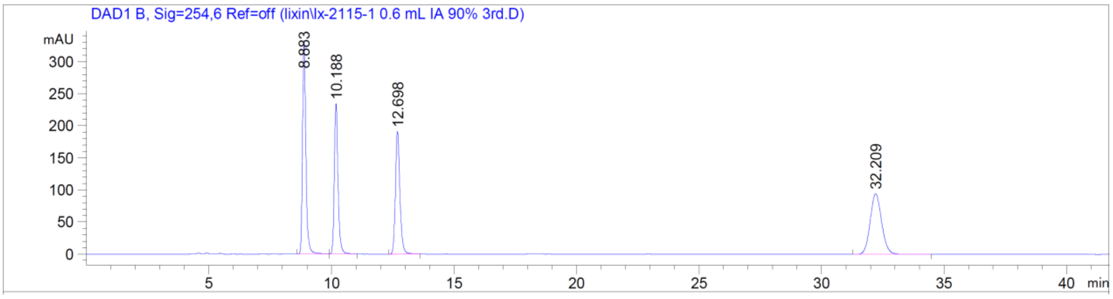

Peak #	RetTime [min]	Type	Width [min]	Area [mAU*s]	Height [mAU]	Area %
1	8.883	BB	0.1417	3112.66821	331.08389	28.0387
2	10.188	BB	0.1578	2439.96582	233.71851	21.9791
3	12.698	BB	0.1958	2453.72412	191.05081	22.1030
4	32.209	BB	0.5037	3094.95703	94.60931	27.8792

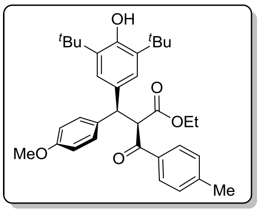

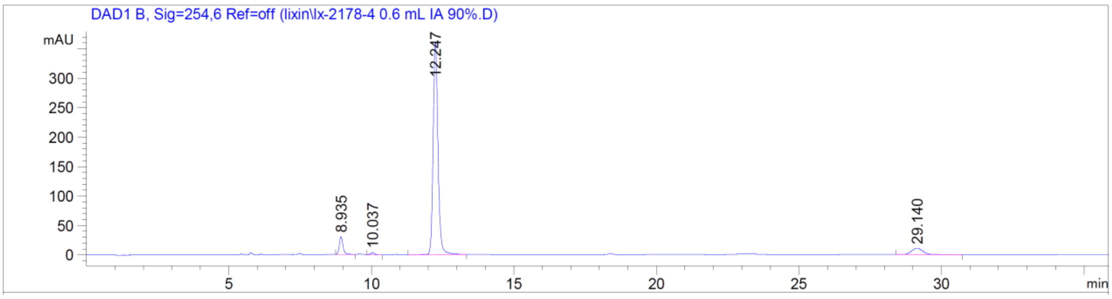

Peak #	RetTime [min]	Type	Width [min]	Area [mAU*s]	Height [mAU]	Area %
1	8.935	BB	0.1346	262.45718	29.83787	5.3612
2	10.037	BB	0.1429	31.40182	3.36352	0.6414
3	12.247	BB	0.1824	4286.68359	361.43555	87.5642
4	29.140	BB	0.4359	314.93463	11.00304	6.4332

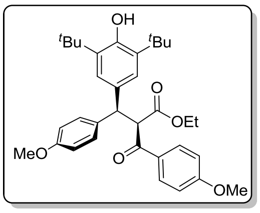
OH
tBu
tBu
O
OEt
MeO
O
OMe

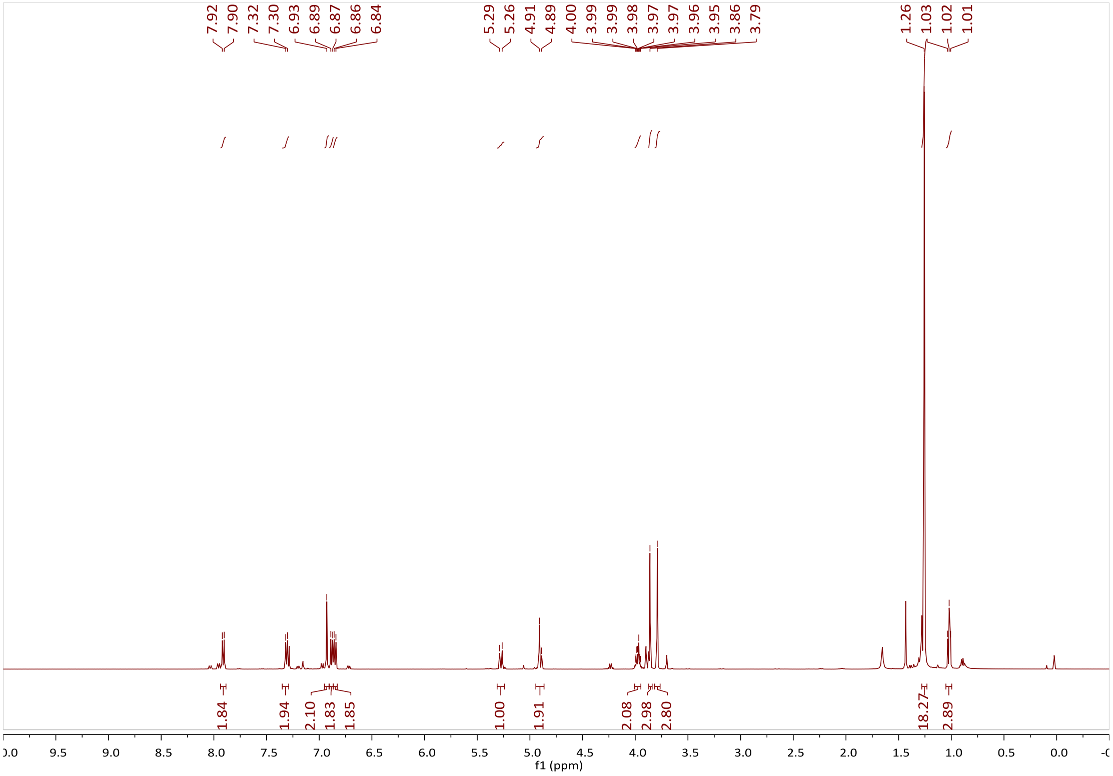
7.92
7.90
7.32
7.30
6.93
6.89
6.87
6.86
6.84
5.29
5.26
4.91
4.89
4.00
3.99
3.99
3.98
3.97
3.97
3.96
3.95
3.86
3.79
1.26
1.03
1.02
1.01
1.84
1.94
2.10
1.83
1.85
1.00
1.91
2.08
2.98
2.80
18.27
2.89
f1 (ppm)

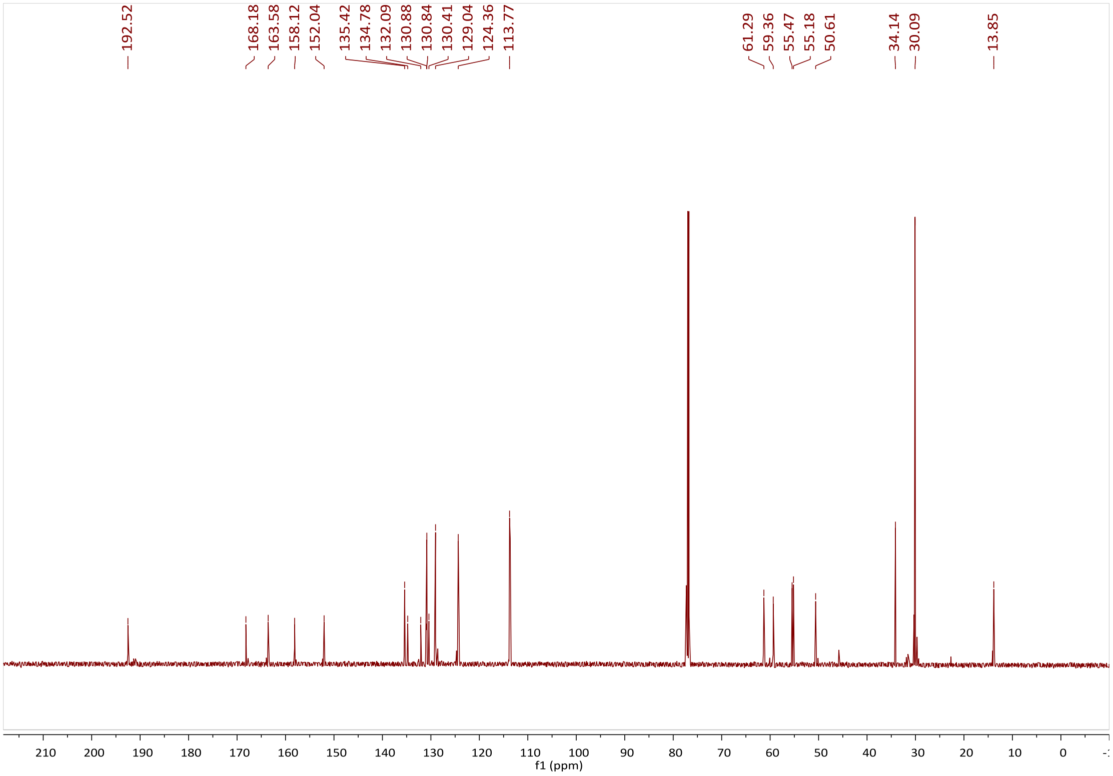
192.52
168.18
163.58
158.12
152.04
135.42
134.78
132.09
130.88
130.84
130.41
129.04
124.36
113.77
61.29
59.36
55.47
55.18
50.61
34.14
30.09
13.85
f1 (ppm)

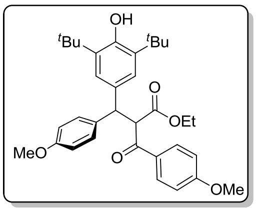

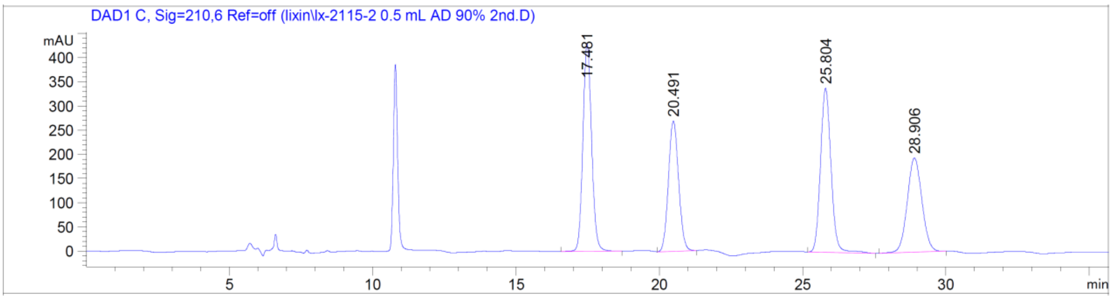

Peak #	RetTime [min]	Type	Width [min]	Area [mAU*s]	Height [mAU]	Area %
1	17.481	BB	0.3276	9069.72168	430.88080	28.9274
2	20.491	BB	0.3857	6641.52588	269.35156	21.1828
3	25.804	BB	0.3973	8700.80664	339.10632	27.7508
4	28.906	BB	0.5579	6941.31982	194.70026	22.1390

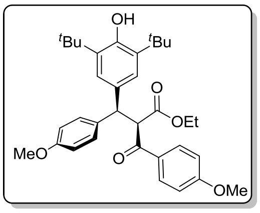

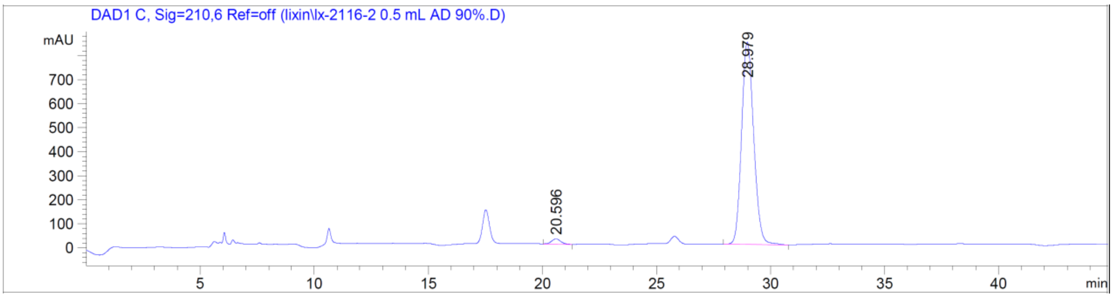

Peak #	RetTime [min]	Type	Width [min]	Area [mAU*s]	Height [mAU]	Area %
1	20.596	BB	0.4187	593.37311	21.99978	1.8766
2	28.979	BB	0.5768	3.10263e4	840.27081	98.1234

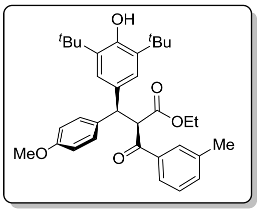

OH
ᵗBu
ᵗBu
O
OEt
MeO
O
Me

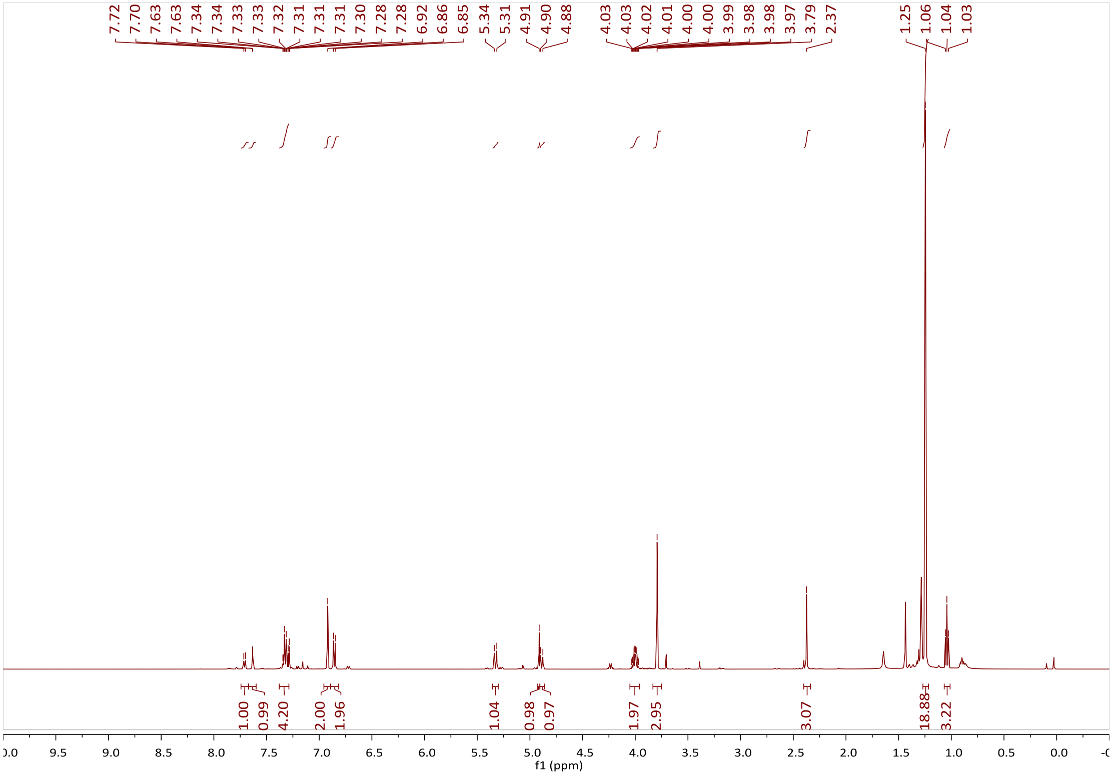

7.72 7.70 7.63 7.63 7.34 7.34 7.33 7.33 7.32 7.31 7.31 7.31 7.30 7.28 7.28 6.92 6.86 6.85 5.34 5.31 4.91 4.90 4.88 4.03 4.03 4.02 4.01 4.00 4.00 3.99 3.98 3.98 3.97 3.79 2.37 1.25 1.06 1.04 1.03
1.00 0.99 4.20 2.00 1.96 1.04 0.98 0.97 1.97 2.95 3.07 18.88 3.22
f1 (ppm)

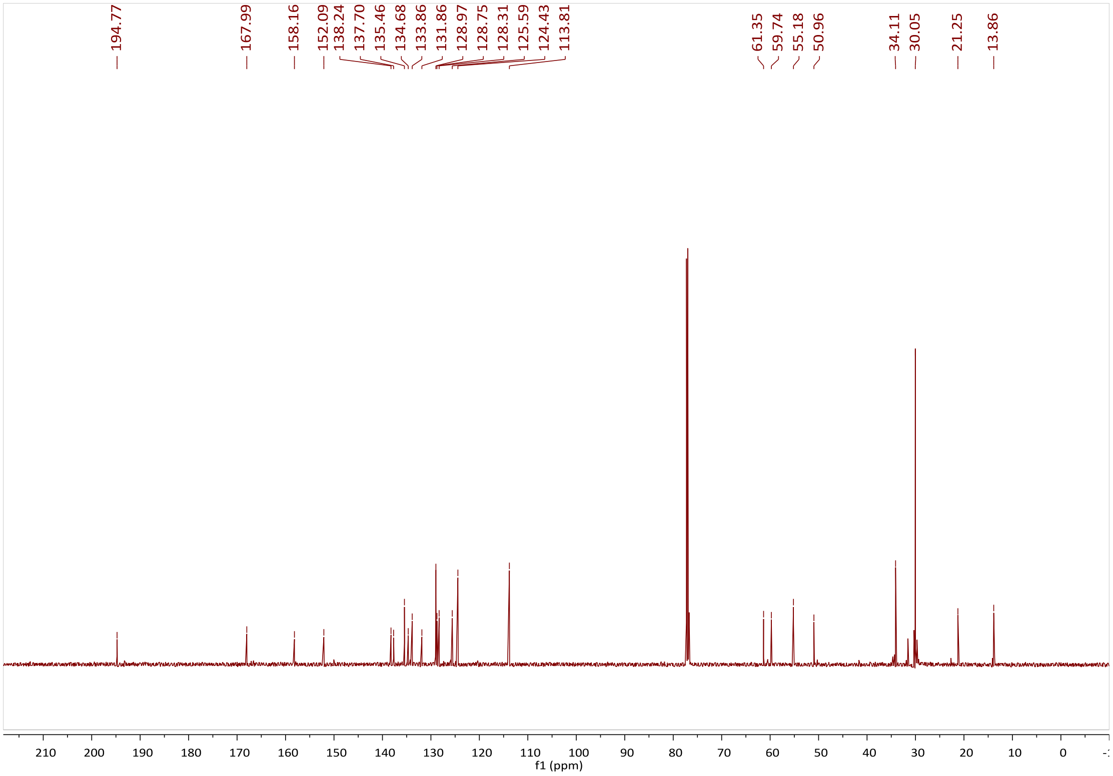

194.77 167.99 158.16 152.09 138.24 137.70 135.46 134.68 133.86 131.86 128.97 128.75 128.31 125.59 124.43 113.81 61.35 59.74 55.18 50.96 34.11 30.05 21.25 13.86
f1 (ppm)

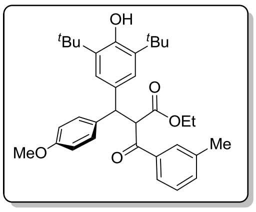

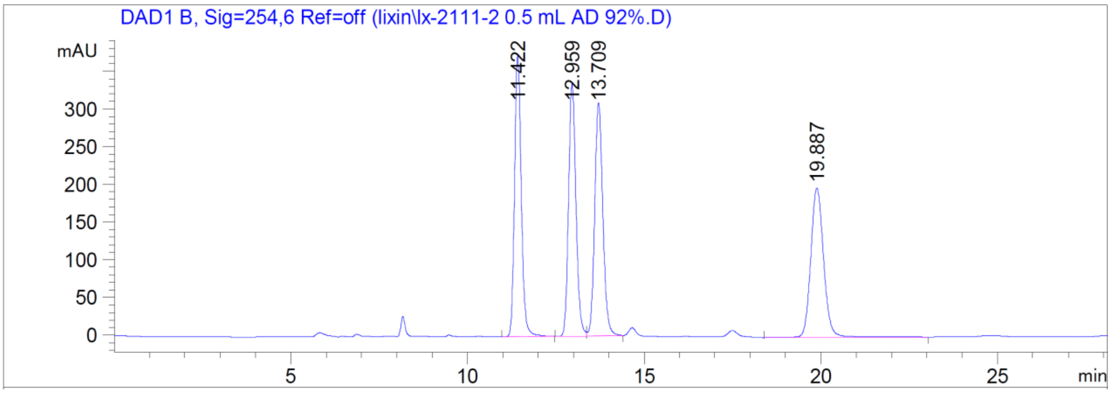

Peak #	RetTime [min]	Type	Width [min]	Area [mAU*s]	Height [mAU]	Area %
1	11.422	BB	0.2032	4961.38135	372.95795	25.0187
2	12.959	BV	0.2264	4872.22510	333.61206	24.5692
3	13.709	VB	0.2515	4939.85742	307.32446	24.9102
4	19.887	BB	0.3937	5057.19141	198.15967	25.5019

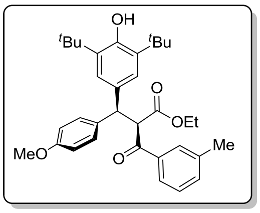

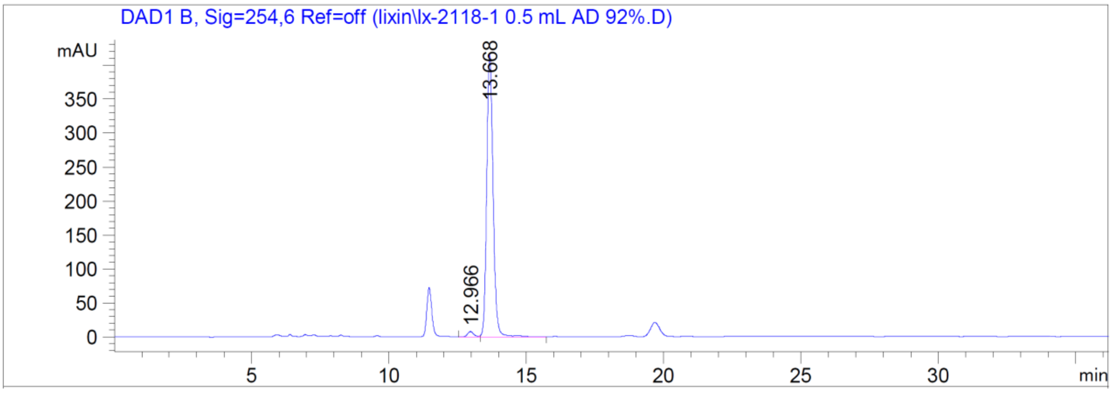

Peak #	RetTime [min]	Type	Width [min]	Area [mAU*s]	Height [mAU]	Area %
1	12.966	BV E	0.2424	116.59943	7.62598	1.7135
2	13.668	VV R	0.2431	6688.10889	415.32471	98.2865

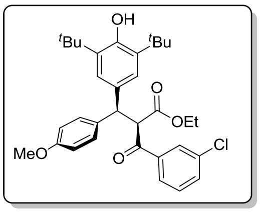

OH
tBu
tBu
O
OEt
MeO
O
Cl

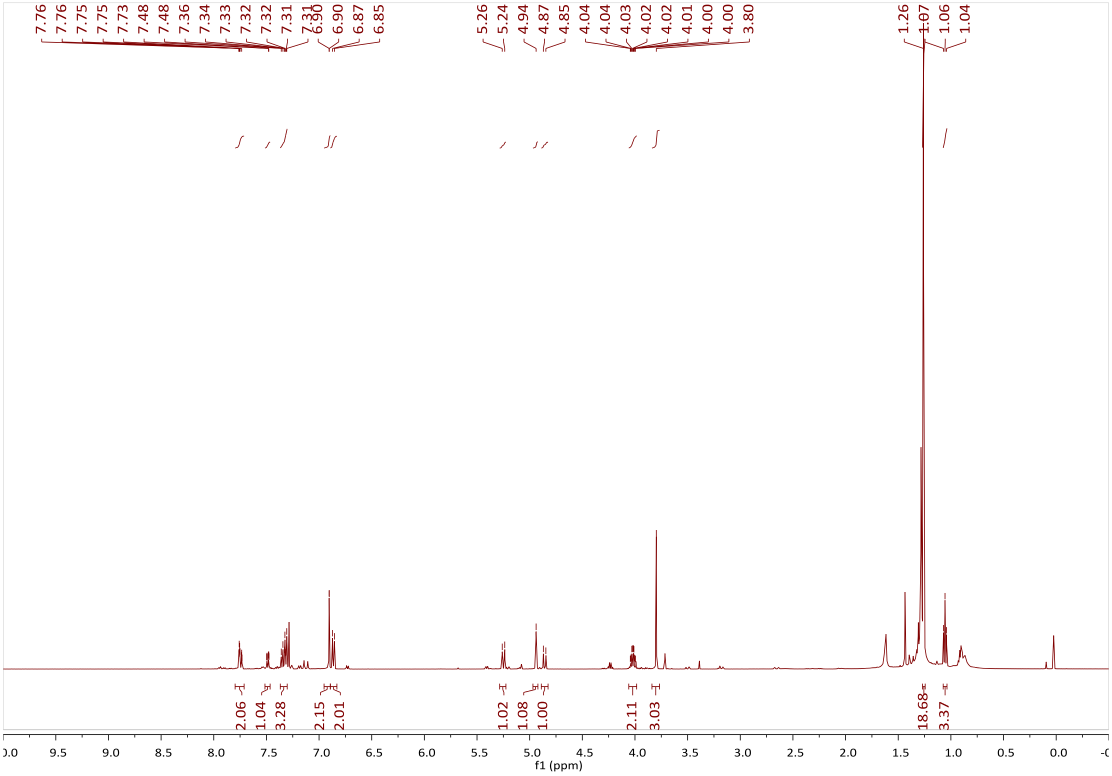

7.76
7.76
7.75
7.75
7.73
7.48
7.48
7.36
7.34
7.33
7.32
7.32
7.31
7.31
6.90
6.90
6.87
6.85
5.26
5.24
4.94
4.87
4.85
4.04
4.04
4.03
4.02
4.02
4.01
4.00
4.00
3.80
1.26
1.07
1.06
1.04
2.06
1.04
3.28
2.15
2.01
1.02
1.08
1.00
2.11
3.03
18.68
3.37
f1 (ppm)

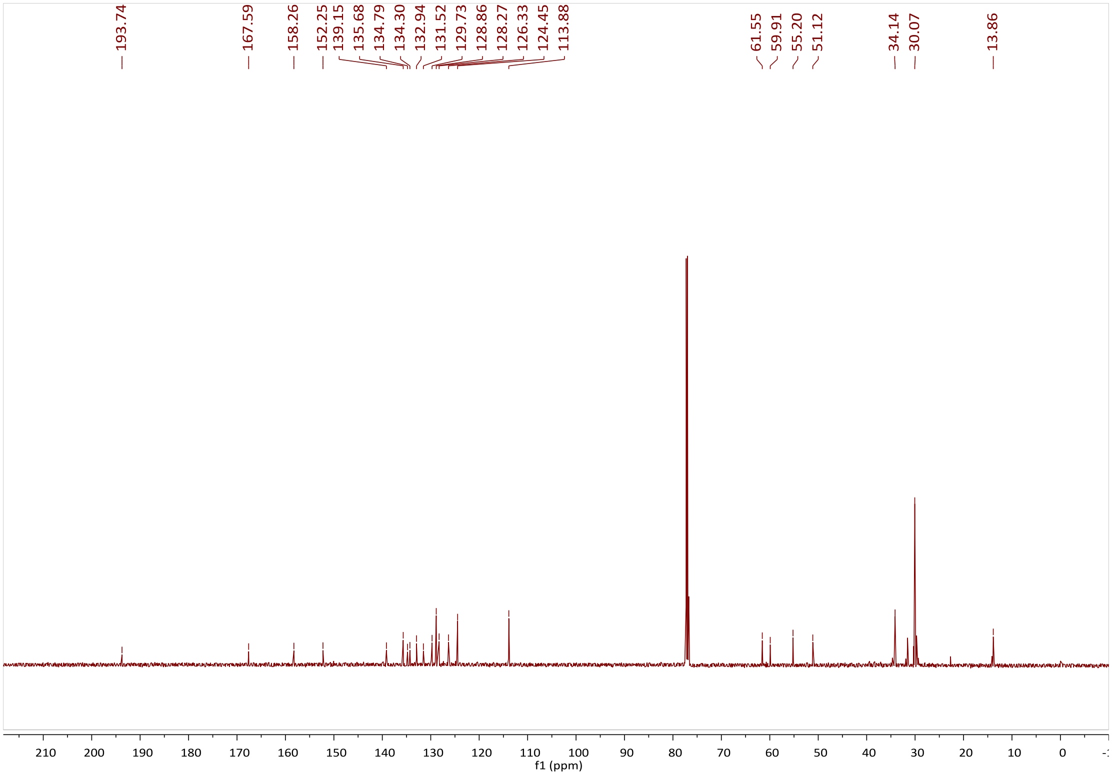

193.74
167.59
158.26
152.25
139.15
135.68
134.79
134.30
132.94
131.52
129.73
128.86
128.27
126.33
124.45
113.88
61.55
59.91
55.20
51.12
34.14
30.07
13.86
f1 (ppm)

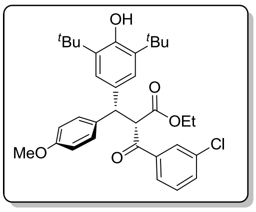

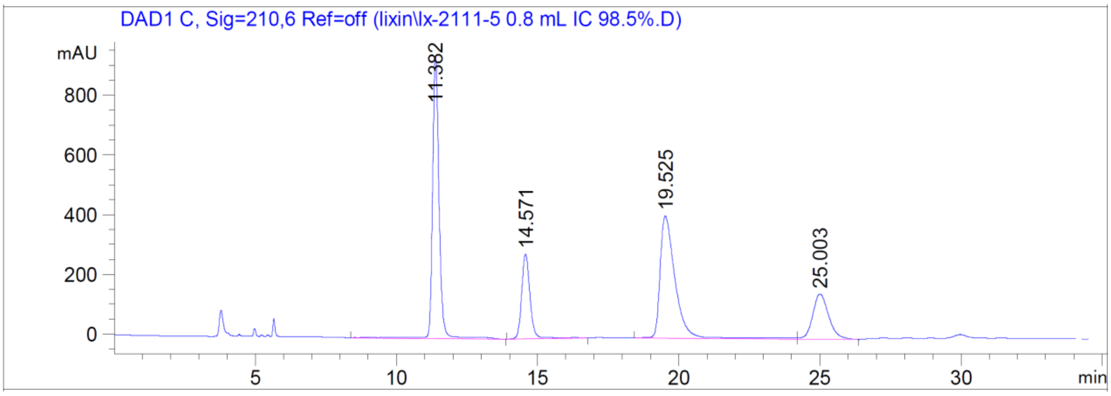

Peak #	RetTime [min]	Type	Width [min]	Area [mAU*s]	Height [mAU]	Area %
1	11.382	VV R	0.2411	1.55251e4	944.63605	36.3763
2	14.571	BV R	0.3089	5914.54346	284.38367	13.8582
3	19.525	BV R	0.5397	1.52807e4	409.29648	35.8038
4	25.003	VB	0.6031	5958.71680	151.42682	13.9617

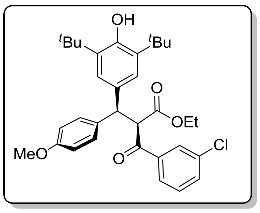

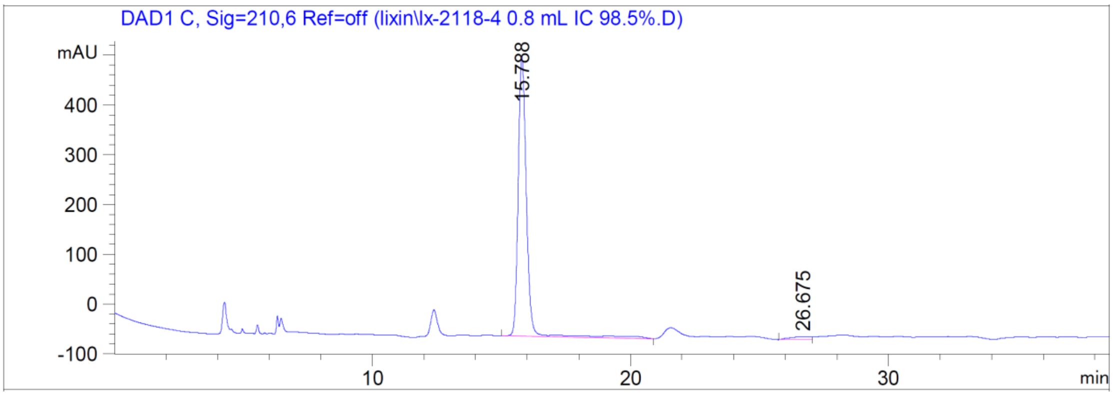

Peak #	RetTime [min]	Type	Width [min]	Area [mAU*s]	Height [mAU]	Area %
1	15.788	BV R	0.3297	1.28646e4	563.77795	97.7830
2	26.675	BV	0.8108	291.67194	5.16509	2.2170

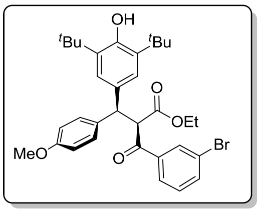
OH
tBu
tBu
O
OEt
MeO
O
Br

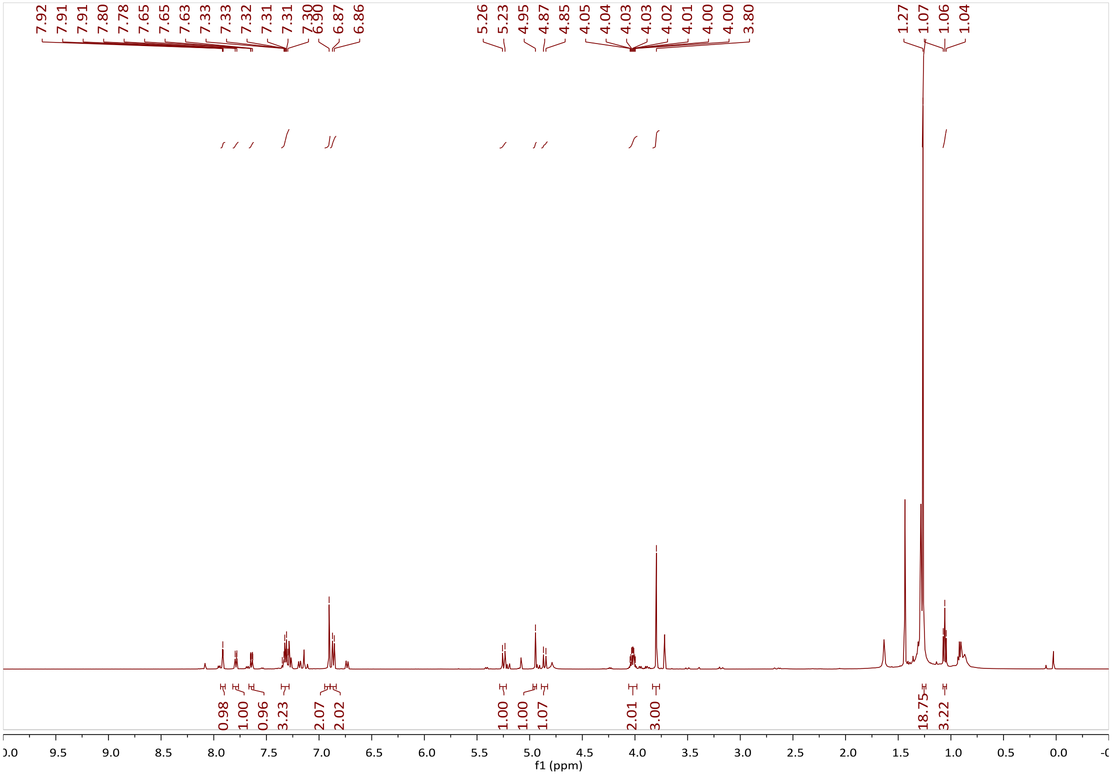

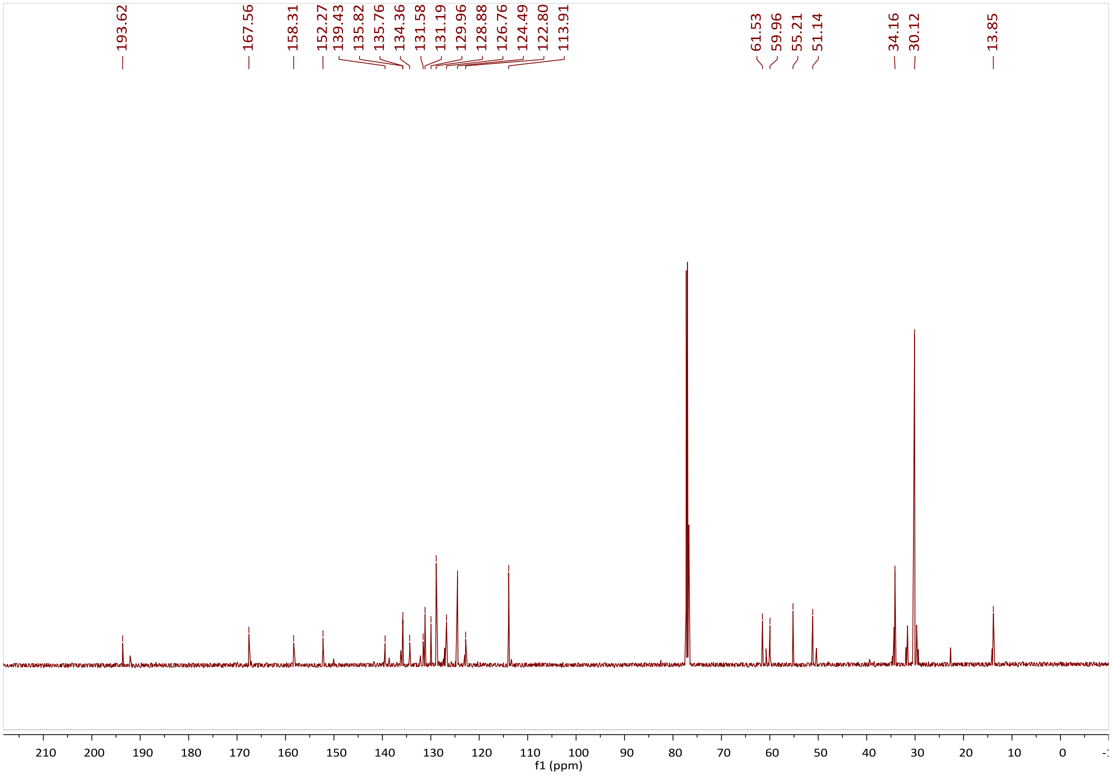

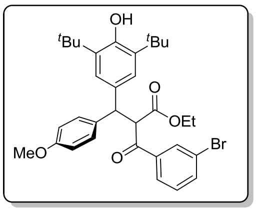

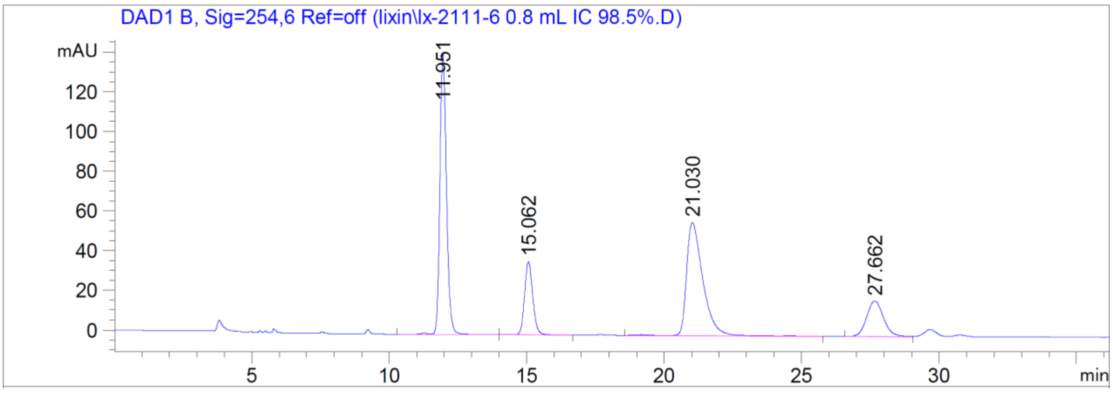

Peak #	RetTime [min]	Type	Width [min]	Area [mAU*s]	Height [mAU]	Area %
1	11.951	VB R	0.2590	2374.21875	140.90221	37.8990
2	15.062	VB R	0.3222	768.55597	36.85001	12.2682
3	21.030	VV R	0.6219	2357.91064	56.98749	37.6386
4	27.662	BV	0.6567	763.91736	17.94394	12.1942

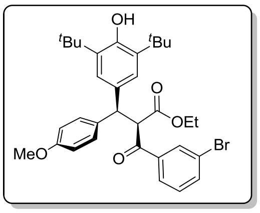

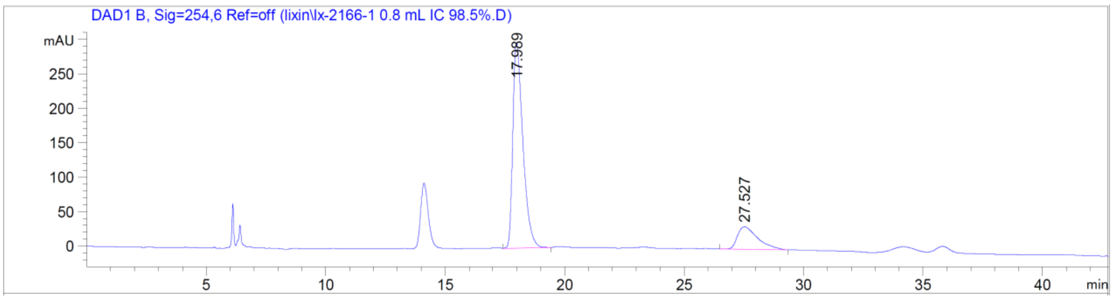

Peak #	RetTime [min]	Type	Width [min]	Area [mAU*s]	Height [mAU]	Area %
1	17.989	BB	0.4470	8757.70020	297.87286	81.8043
2	27.527	BB	0.8647	1947.96619	32.80621	18.1957

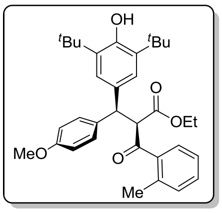
OH
tBu
tBu
O
OEt
MeO
O
Me

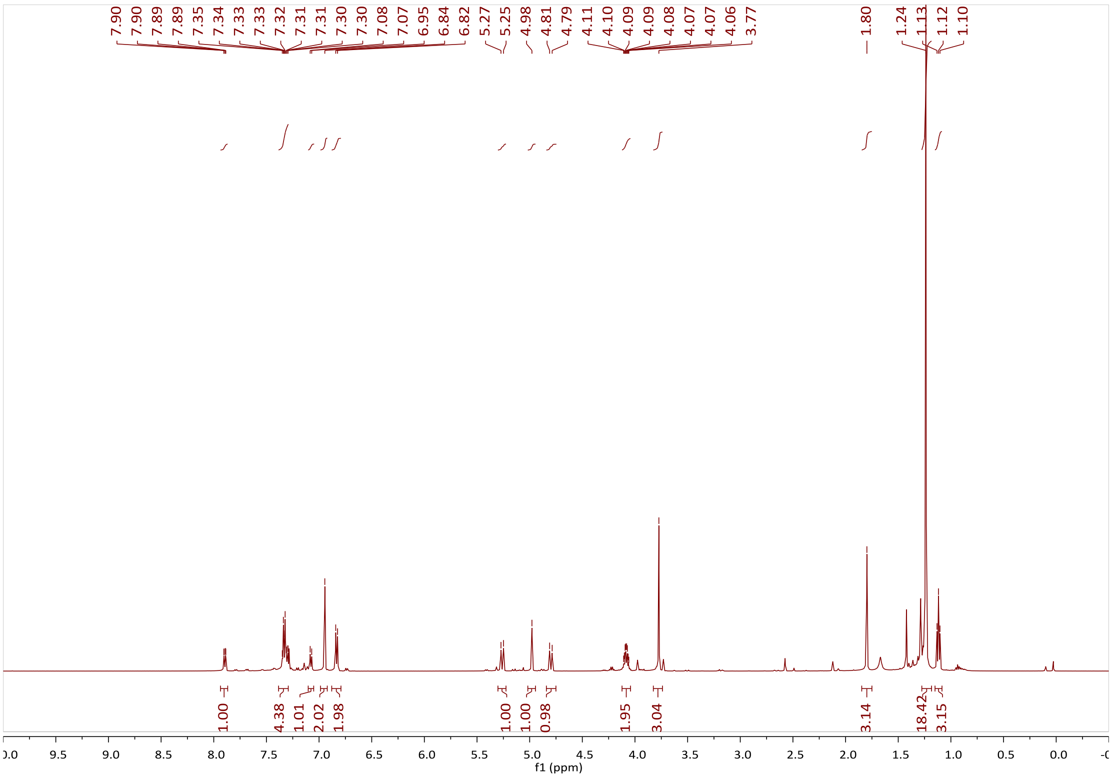
7.90 7.90 7.89 7.89 7.35 7.34 7.33 7.33 7.32 7.31 7.31 7.30 7.30 7.08 7.07 6.95 6.84 6.82 5.27 5.25 4.98 4.81 4.79 4.11 4.10 4.09 4.09 4.08 4.07 4.07 4.06 3.77 1.80 1.24 1.13 1.12 1.10
1.00 4.38 1.01 2.02 1.98 1.00 1.00 0.98 1.95 3.04 3.14 18.42 3.15
f1 (ppm)

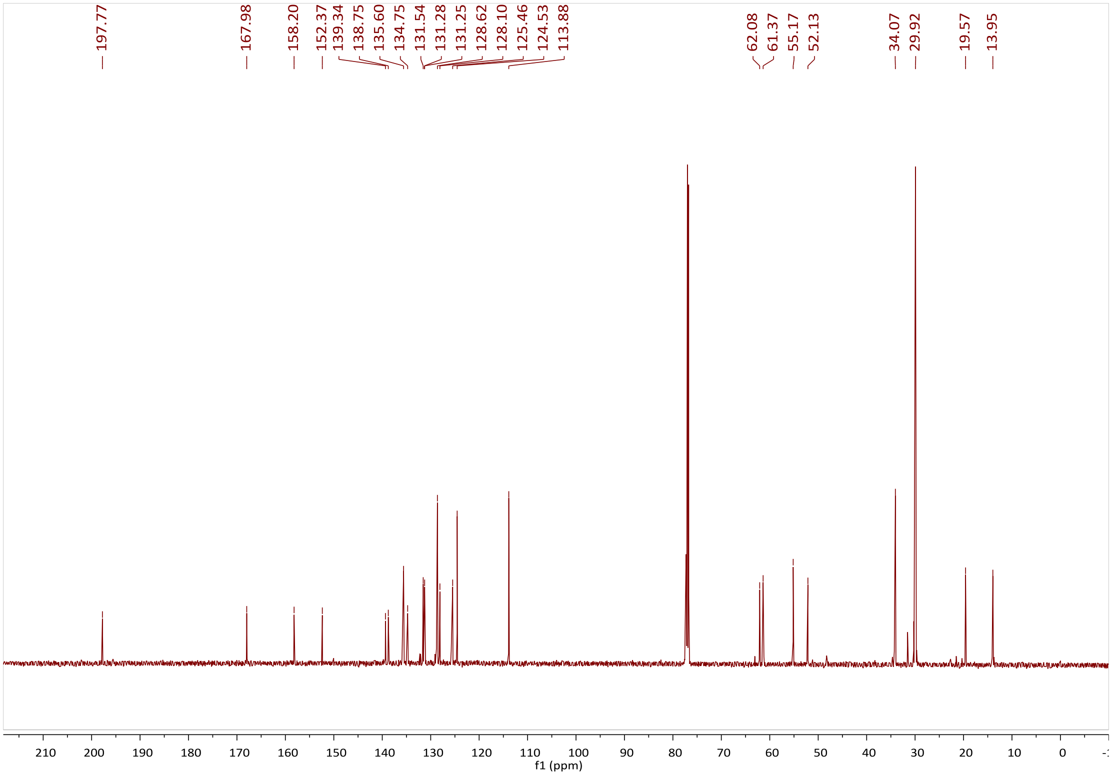
197.77 167.98 158.20 152.37 139.34 138.75 135.60 134.75 131.54 131.28 131.25 128.62 128.10 125.46 124.53 113.88 62.08 61.37 55.17 52.13 34.07 29.92 19.57 13.95
f1 (ppm)

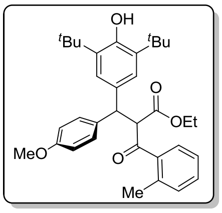

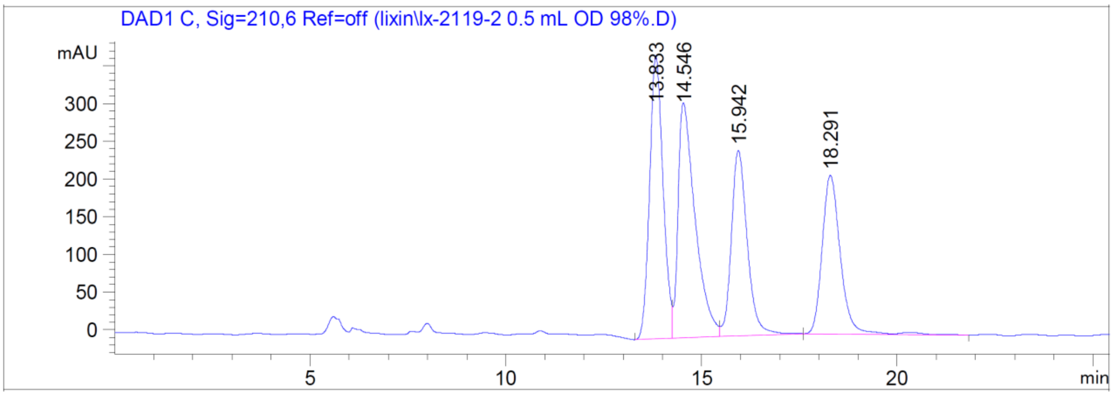

Peak #	RetTime [min]	Type	Width [min]	Area [mAU*s]	Height [mAU]	Area %
1	13.833	BV	0.3755	8991.82129	375.30170	27.7951
2	14.546	VV	0.4524	9495.72168	310.84753	29.3527
3	15.942	VB	0.4349	6971.92627	245.83466	21.5513
4	18.291	BV R	0.4964	6890.91699	210.10516	21.3009

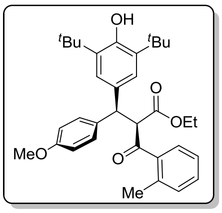

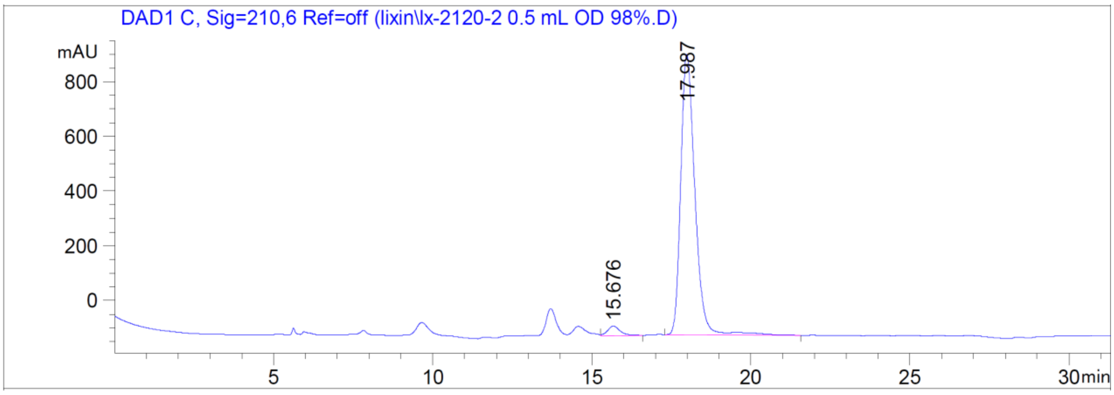

Peak #	RetTime [min]	Type	Width [min]	Area [mAU*s]	Height [mAU]	Area %
1	15.676	VB	0.4228	985.88251	35.19396	3.0103
2	17.987	BV R	0.4743	3.17644e4	1022.72363	96.9897

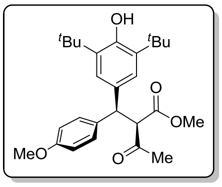
OH
tBu
tBu
O
OMe
MeO
O
Me

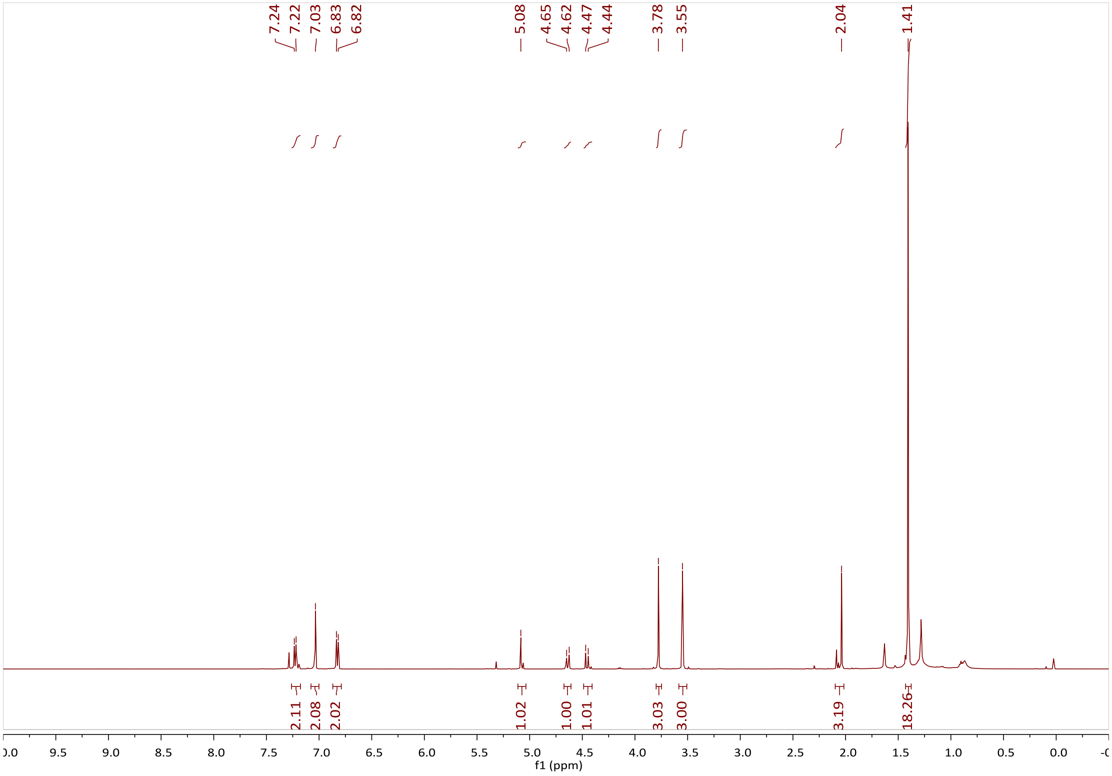
7.24
7.22
7.03
6.83
6.82
5.08
4.65
4.62
4.47
4.44
3.78
3.55
2.04
1.41
2.11
2.08
2.02
1.02
1.00
1.01
3.03
3.00
3.19
18.26
f1 (ppm)

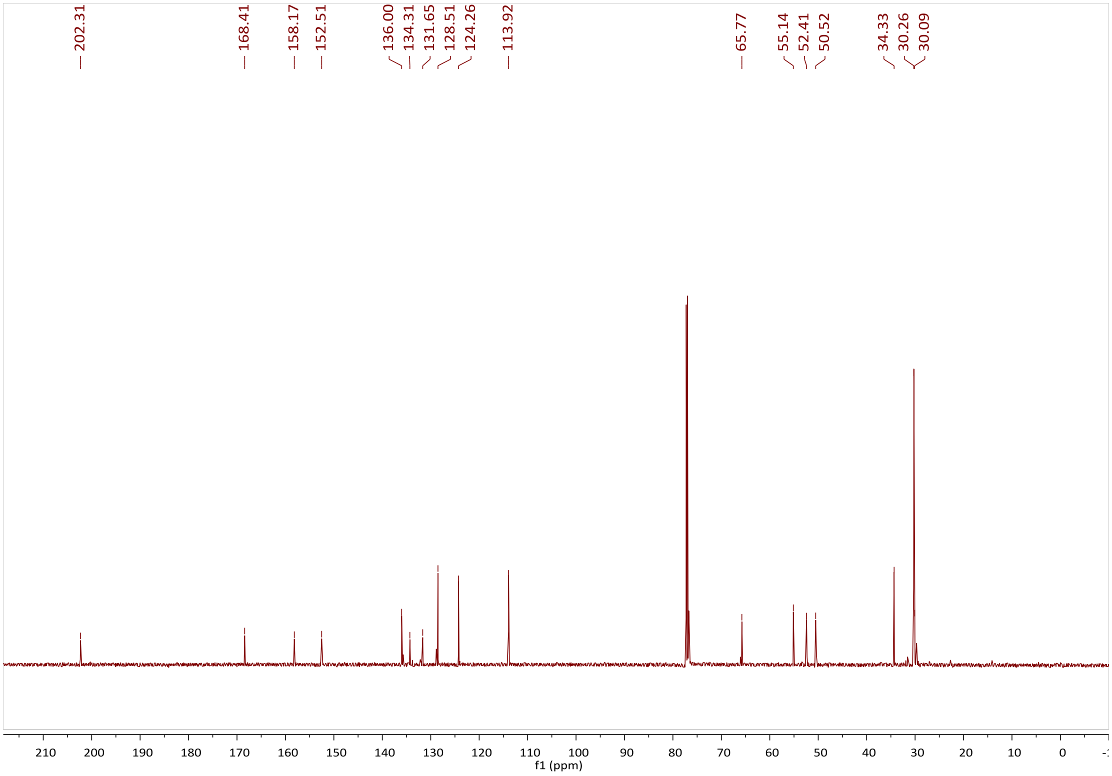
202.31
168.41
158.17
152.51
136.00
134.31
131.65
128.51
124.26
113.92
65.77
55.14
52.41
50.52
34.33
30.26
30.09
f1 (ppm)

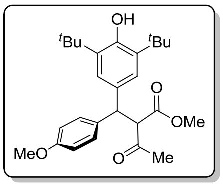

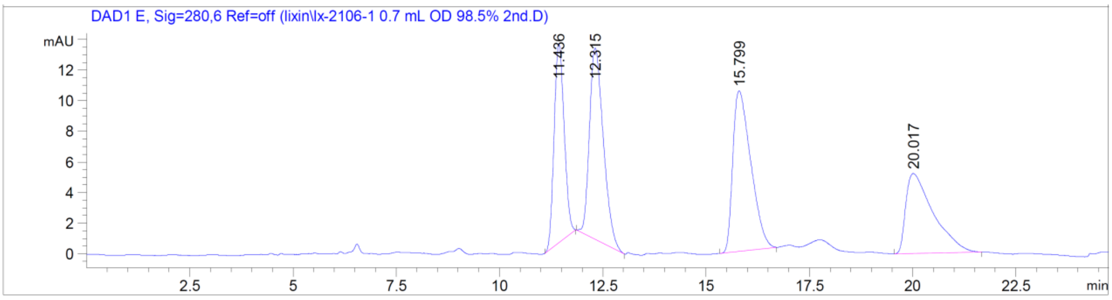

Peak #	RetTime [min]	Type	Width [min]	Area [mAU*s]	Height [mAU]	Area %
1	11.436	BB	0.2630	219.33919	12.98876	20.5095
2	12.315	BB	0.3612	292.10883	12.47531	27.3139
3	15.799	BB	0.4487	317.07516	10.48841	29.6484
4	20.017	BB	0.6359	240.92737	5.22547	22.5281

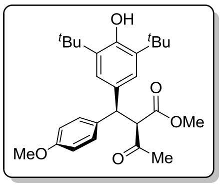

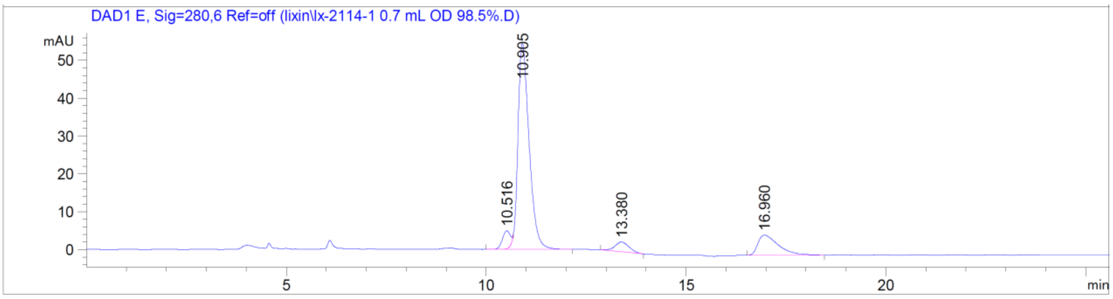

Peak #	RetTime [min]	Type	Width [min]	Area [mAU*s]	Height [mAU]	Area %
1	10.516	BV E	0.2096	63.71282	4.71795	4.6259
2	10.905	VB R	0.2949	1059.58069	54.47292	76.9314
3	13.380	BB	0.3730	64.78043	2.59731	4.7034
4	16.960	BB	0.5166	189.23131	5.32526	13.7392

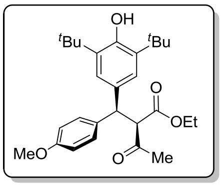
OH
tBu
tBu
O
OEt
MeO
O
Me

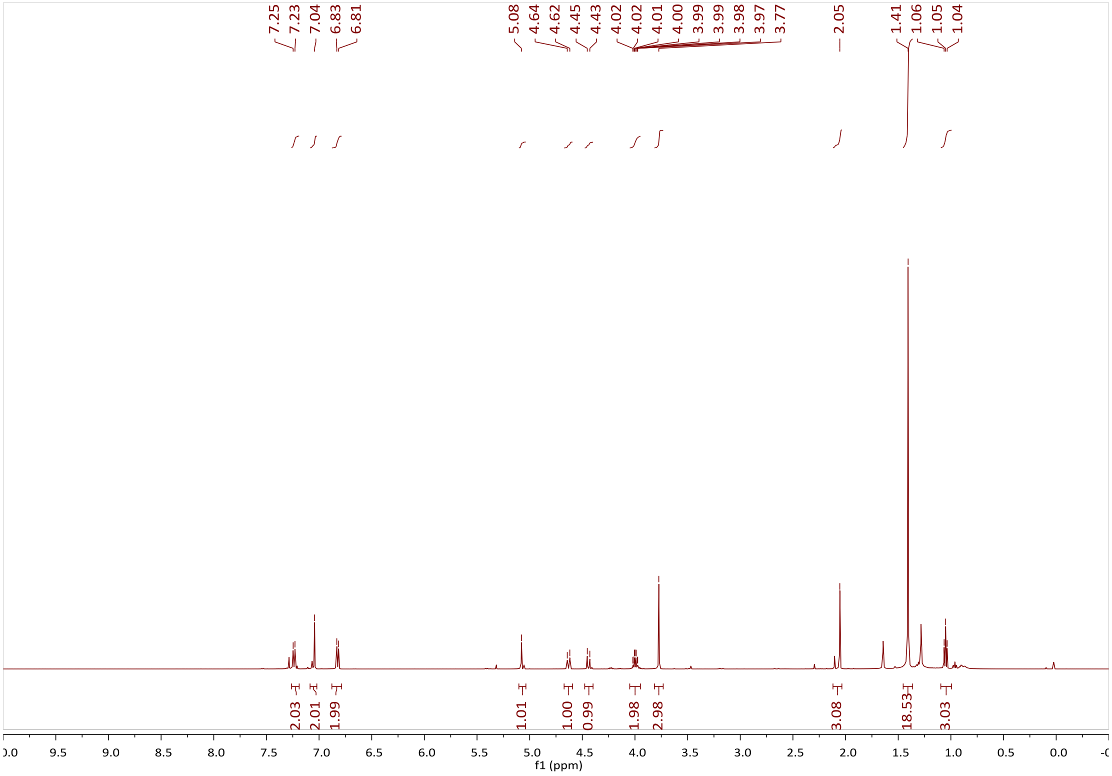
7.25
7.23
7.04
6.83
6.81
5.08
4.64
4.62
4.45
4.43
4.02
4.02
4.01
4.00
3.99
3.99
3.98
3.97
3.77
2.05
1.41
1.06
1.05
1.04
2.03
2.01
1.99
1.01
1.00
0.99
1.98
2.98
3.08
18.53
3.03
f1 (ppm)

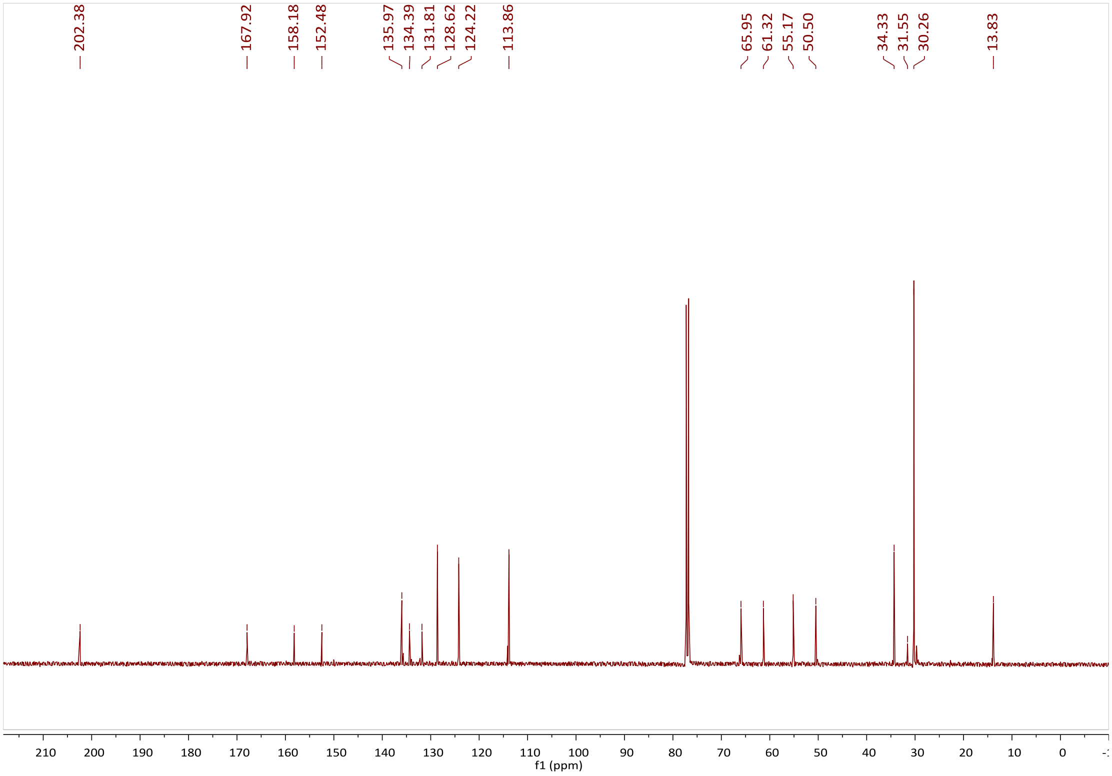
202.38
167.92
158.18
152.48
135.97
134.39
131.81
128.62
124.22
113.86
65.95
61.32
55.17
50.50
34.33
31.55
30.26
13.83
f1 (ppm)

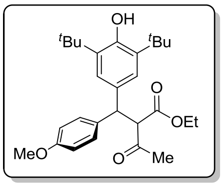

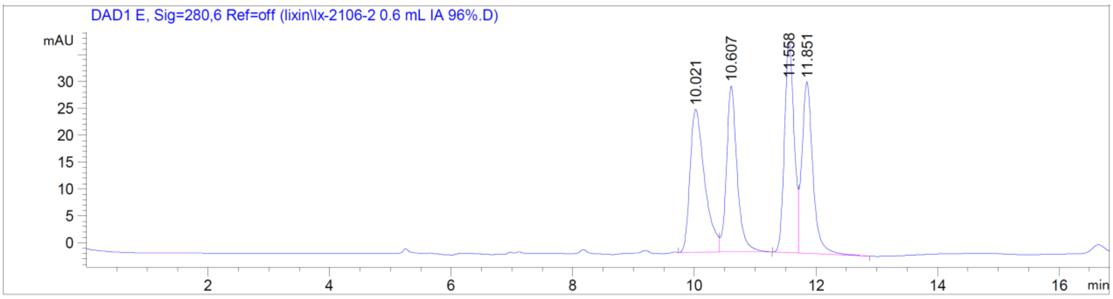

Peak #	RetTime [min]	Type	Width [min]	Area [mAU*s]	Height [mAU]	Area %
1	10.021	BV	0.2526	447.91220	26.58543	26.9907
2	10.607	VB	0.1822	376.92108	30.92938	22.7128
3	11.558	BV	0.1688	431.11761	39.04399	25.9787
4	11.851	VB	0.1852	403.55502	31.98929	24.3178

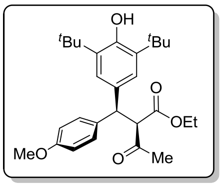

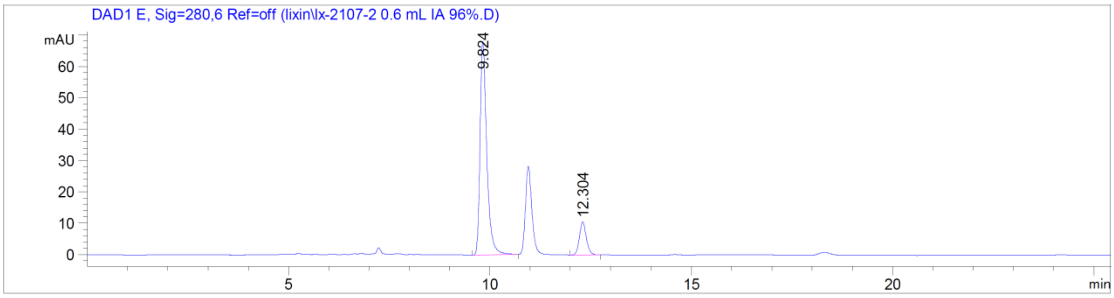

Peak #	RetTime [min]	Type	Width [min]	Area [mAU*s]	Height [mAU]	Area %
1	9.824	BB	0.1738	789.80359	67.87210	86.3255
2	12.304	BB	0.1831	125.11005	10.49387	13.6745

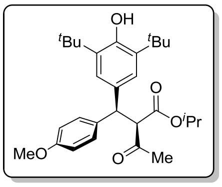
OH
tBu
tBu
O
OiPr
MeO
O
Me

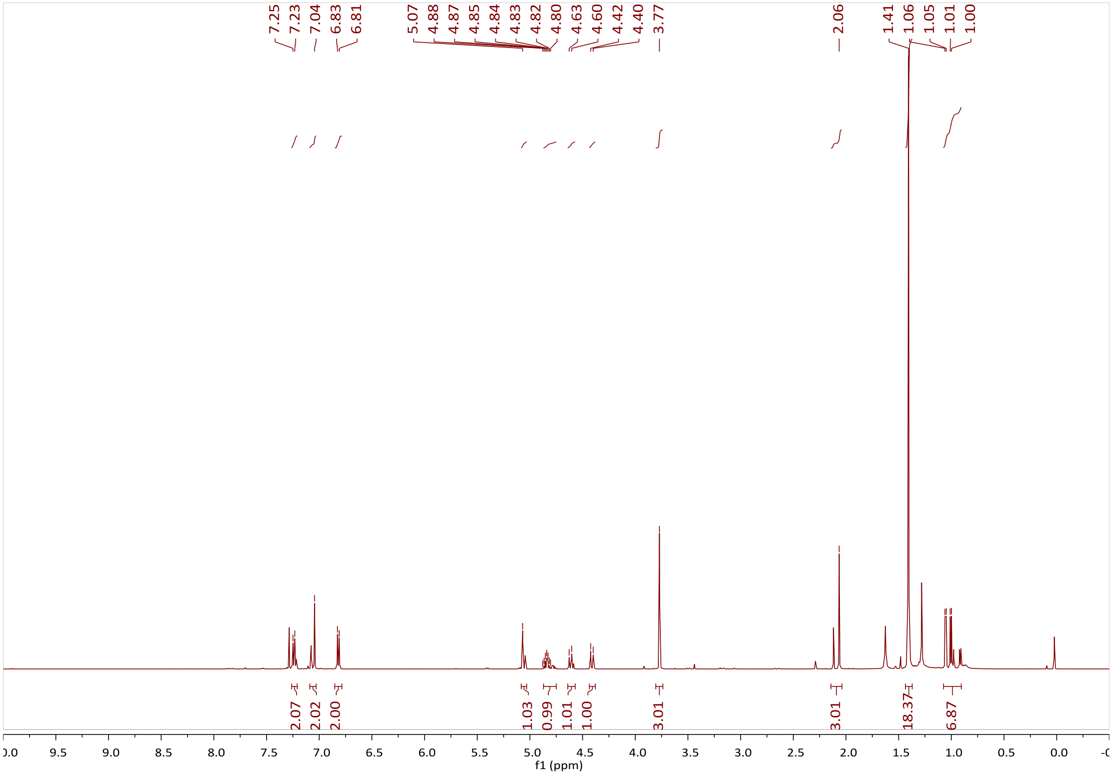

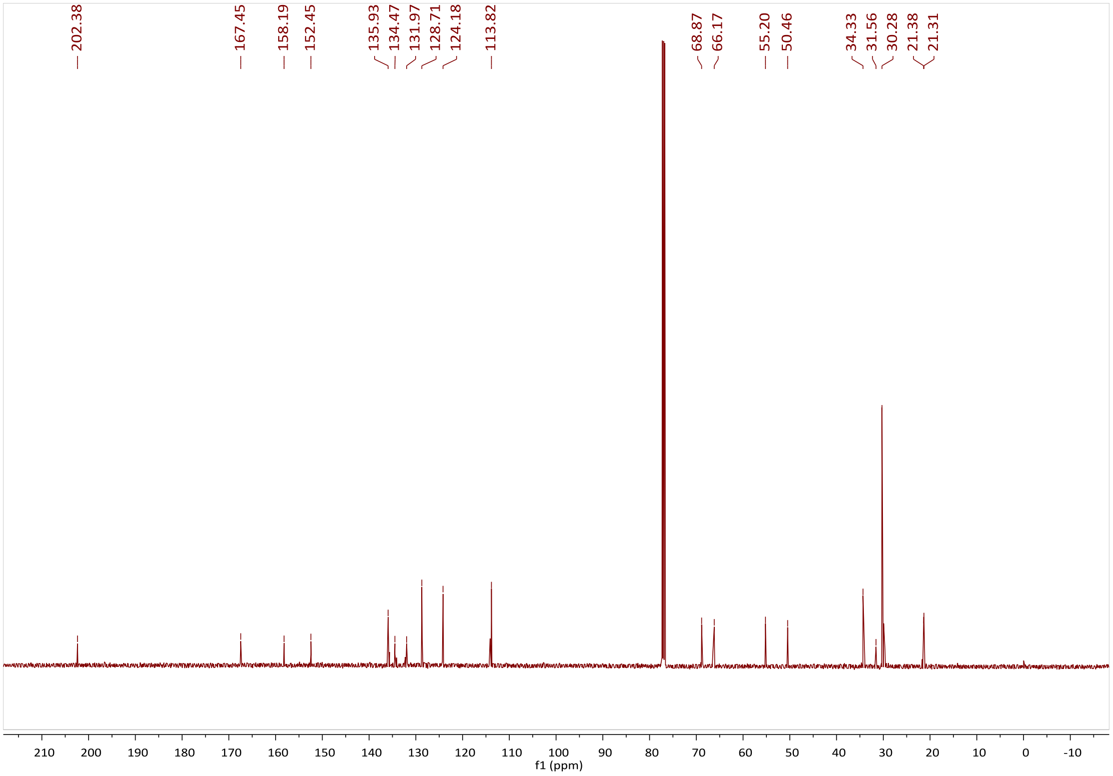

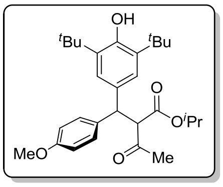

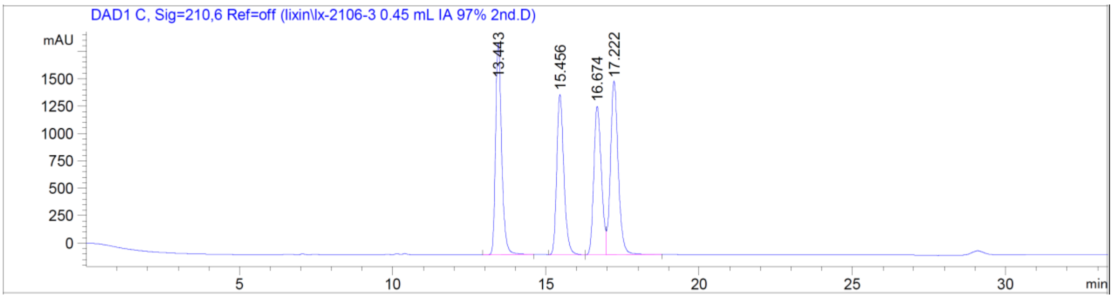

Peak #	RetTime [min]	Type	Width [min]	Area [mAU*s]	Height [mAU]	Area %
1	13.443	BB	0.2108	2.67177e4	1937.90088	26.2849
2	15.456	BB	0.2458	2.35157e4	1460.98657	23.1348
3	16.674	BV	0.2669	2.33474e4	1355.93909	22.9693
4	17.222	VB	0.2684	2.80656e4	1586.52917	27.6110

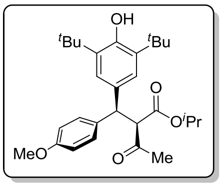

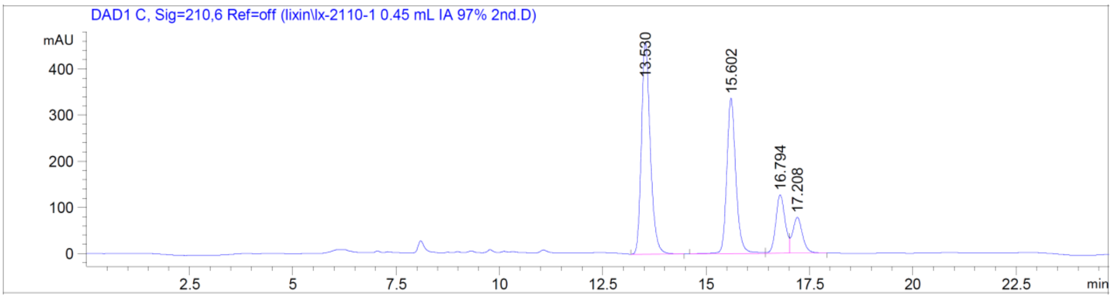

Peak #	RetTime [min]	Type	Width [min]	Area [mAU*s]	Height [mAU]	Area %
1	13.530	BB	0.2280	6844.13477	458.81247	44.8408
2	15.602	BV	0.2325	5152.74561	336.78214	33.7593
3	16.794	VV	0.2414	1989.53760	126.53970	13.0349
4	17.208	VB	0.2482	1276.76123	77.49190	8.3650

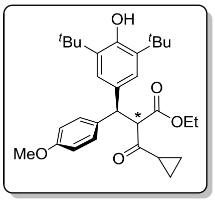

dr = 1.8:1

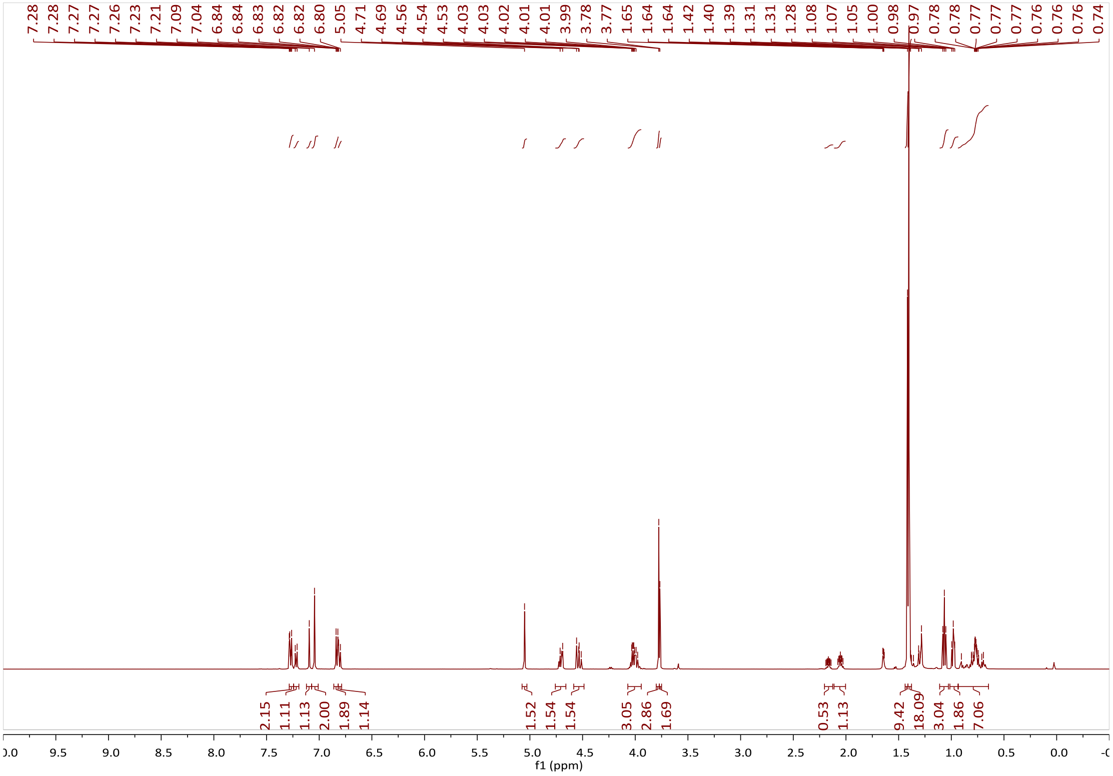

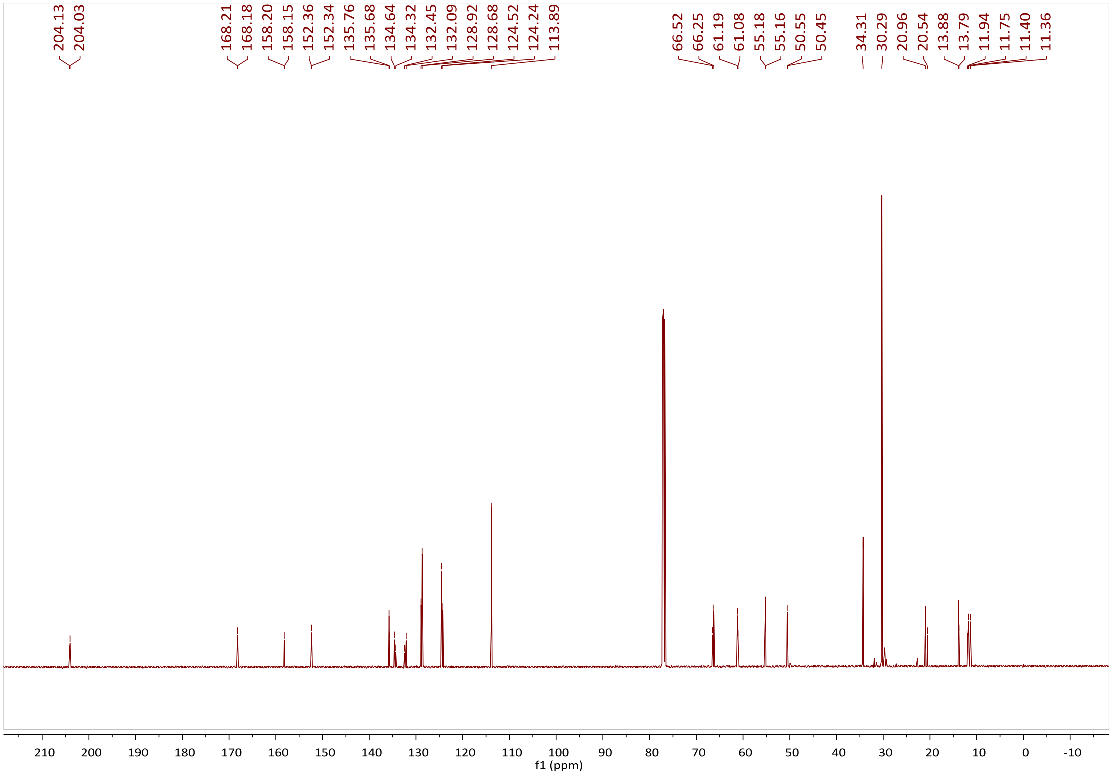

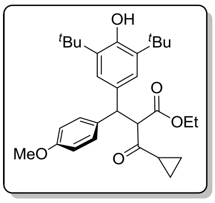

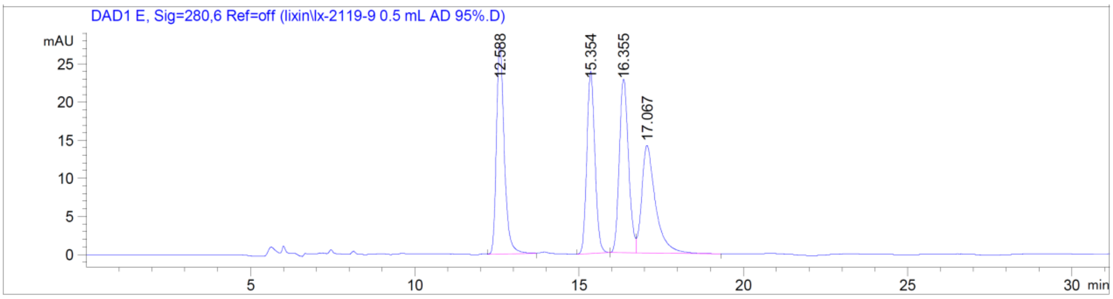

Peak #	RetTime [min]	Type	Width [min]	Area [mAU*s]	Height [mAU]	Area %
1	12. 588	BB	0. 2486	455. 50131	27. 58817	26. 5030
2	15. 354	BB	0. 2607	406. 24695	23. 85800	23. 6371
3	16. 355	BV	0. 3037	444. 05624	22. 75021	25. 8370
4	17. 067	VB	0. 4295	412. 87698	14. 10403	24. 0229

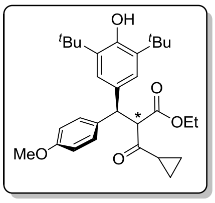

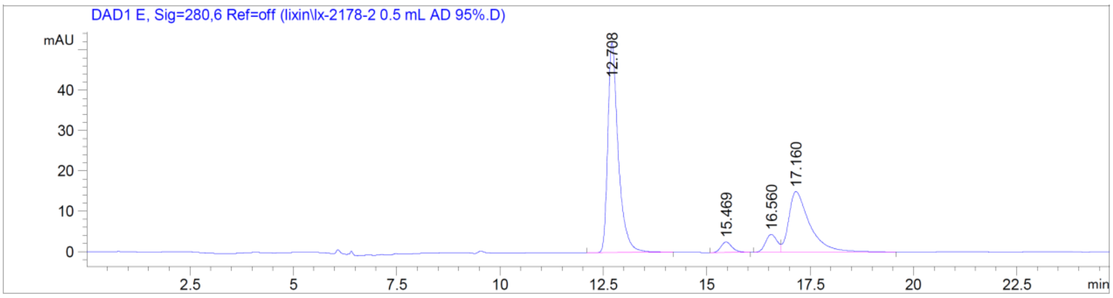

Peak #	RetTime [min]	Type	Width [min]	Area [mAU*s]	Height [mAU]	Area %
1	12. 708	BB	0. 2639	919. 29767	52. 08566	57. 7148
2	15. 469	BB	0. 2882	49. 06168	2. 62363	3. 0802
3	16. 560	BV	0. 2975	86. 59732	4. 44101	5. 4367
4	17. 160	VB	0. 5190	537. 87042	15. 04872	33. 7683

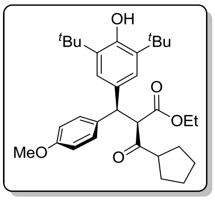
OH
tBu
tBu
O
OEt
MeO
O

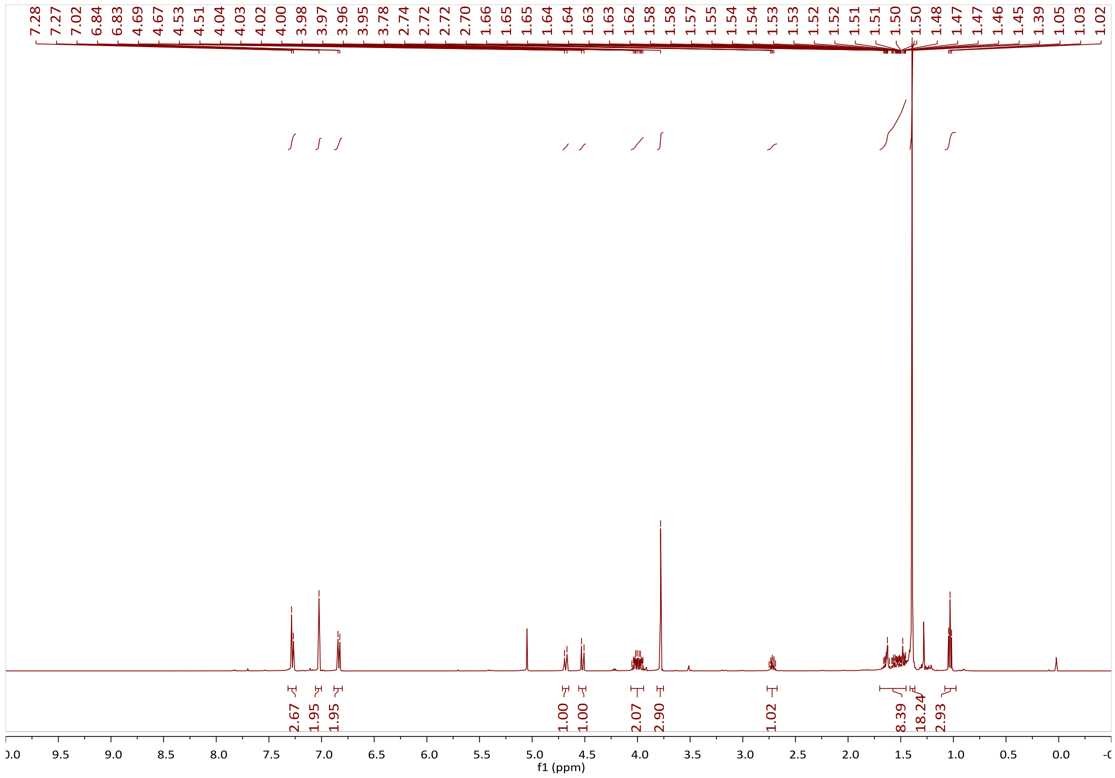

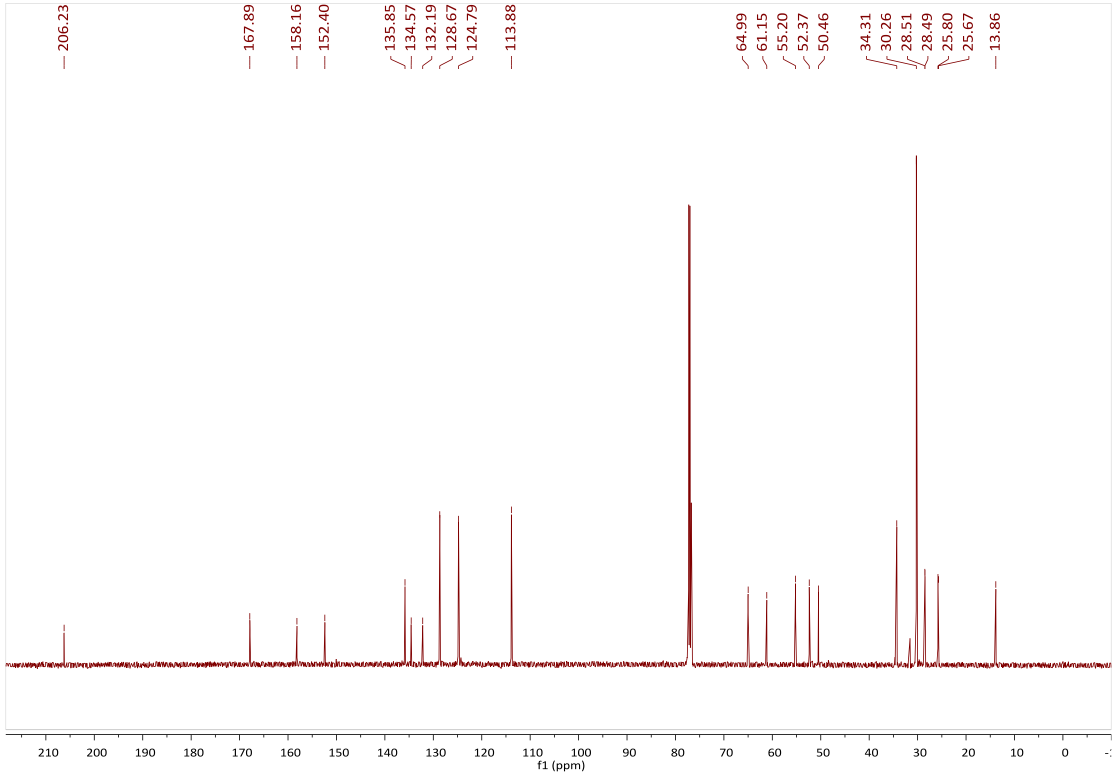

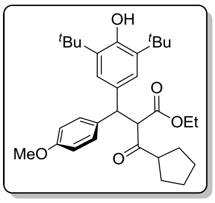

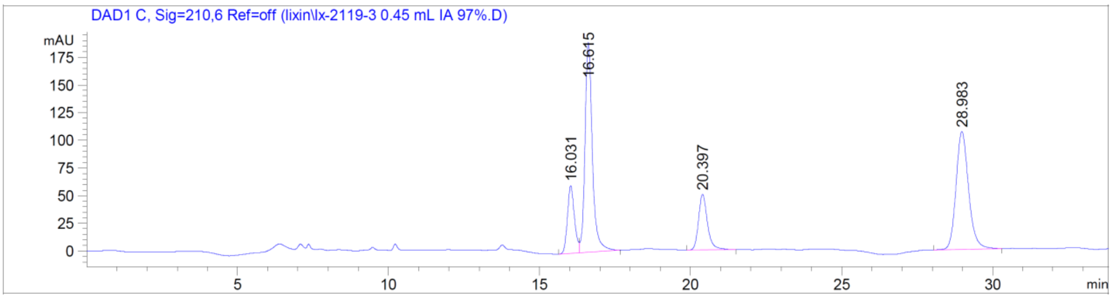

Peak #	RetTime [min]	Type	Width [min]	Area [mAU*s]	Height [mAU]	Area %
1	16.031	BV	0.2465	987.44672	61.10079	11.8594
2	16.615	VB	0.2559	3210.76123	189.24934	38.5619
3	20.397	BB	0.3113	1042.77161	50.40765	12.5239
4	28.983	BB	0.4377	3085.27881	106.58026	37.0548

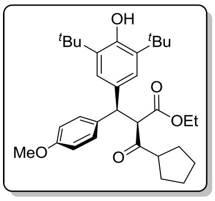

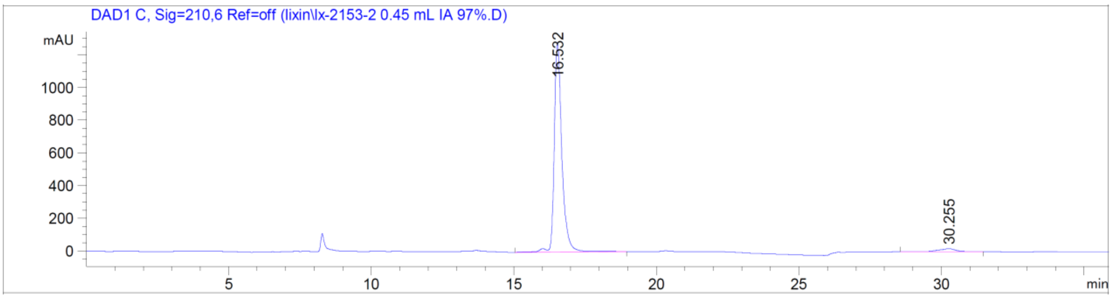

Peak #	RetTime [min]	Type	Width [min]	Area [mAU*s]	Height [mAU]	Area %
1	16.532	VB R	0.2728	2.37779e4	1282.13757	96.7913
2	30.255	BB	0.6084	788.26190	18.01918	3.2087

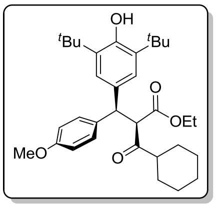
OH
tBu
tBu
O
OEt
MeO
O

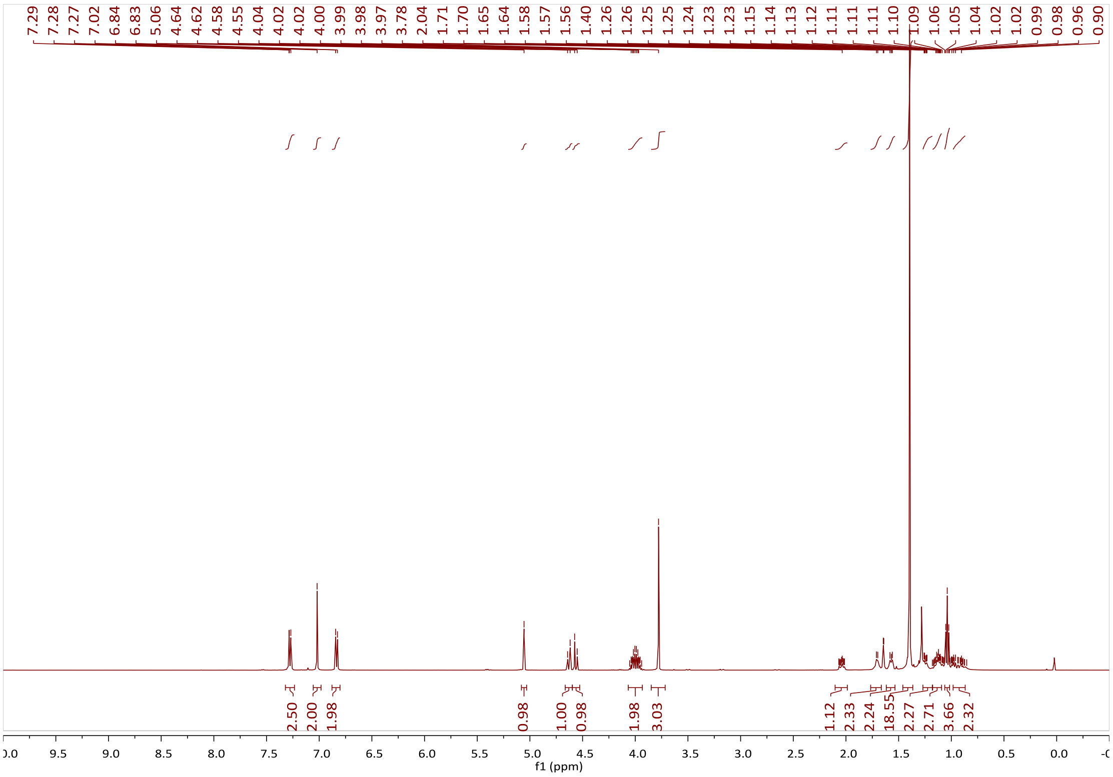
7.29 7.28 7.27 7.02 6.84 6.83 5.06 4.64 4.62 4.58 4.55 4.04 4.02 4.00 3.99 3.98 3.97 3.78 2.04 1.71 1.70 1.65 1.64 1.58 1.57 1.56 1.40 1.26 1.25 1.25 1.24 1.23 1.23 1.15 1.14 1.13 1.12 1.11 1.11 1.11 1.10 1.09 1.06 1.05 1.04 1.02 1.02 0.99 0.98 0.96 0.90
2.50 2.00 1.98 0.98 1.00 0.98 1.98 3.03 1.12 2.33 2.24 18.55 2.27 2.71 3.66 2.32
f1 (ppm)

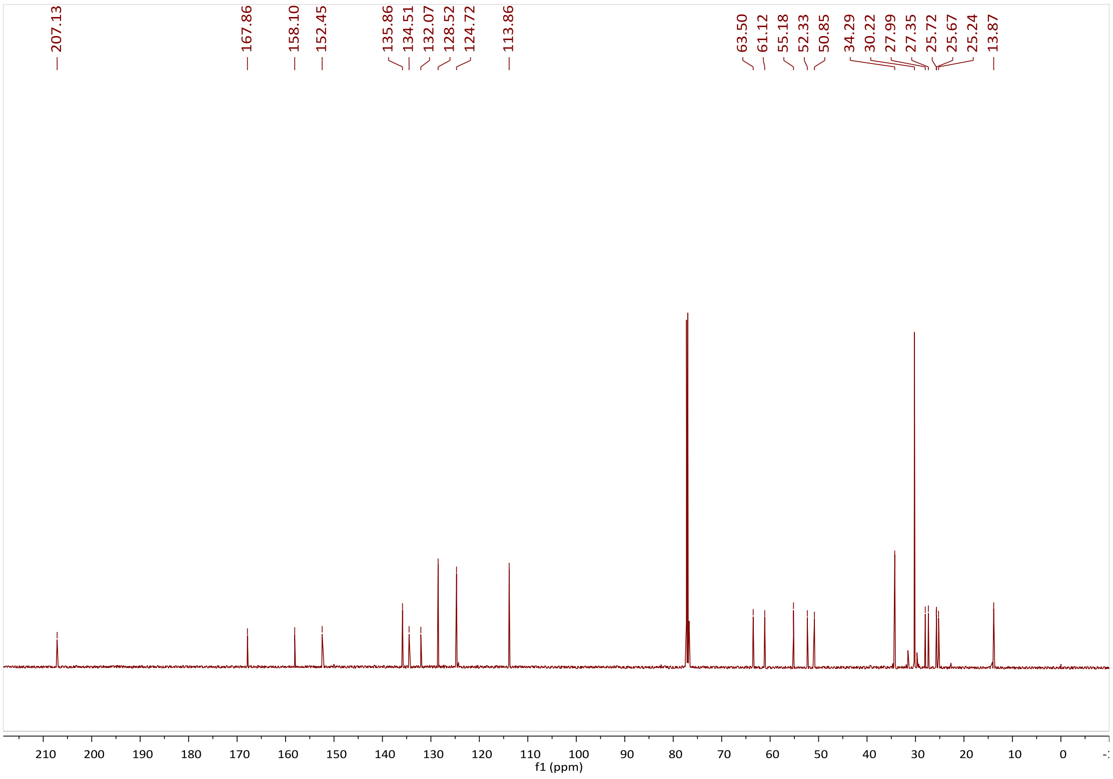
207.13 167.86 158.10 152.45 135.86 134.51 132.07 128.52 124.72 113.86 63.50 61.12 55.18 52.33 50.85 34.29 30.22 27.99 27.35 25.72 25.67 25.24 13.87
f1 (ppm)

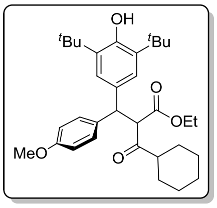

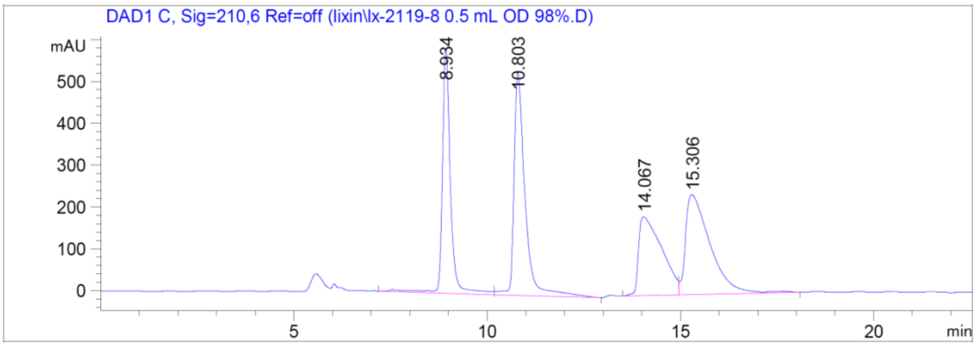

Peak #	RetTime [min]	Type	Width [min]	Area [mAU*s]	Height [mAU]	Area %
1	8.934	VV R	0.2163	8610.02148	574.67969	22.8665
2	10.803	VB	0.2999	1.06124e4	524.86365	28.1845
3	14.067	BV	0.7054	7870.21094	187.62021	20.9017
4	15.306	VV R	0.6655	1.05608e4	237.95599	28.0473

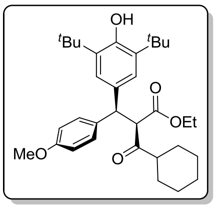

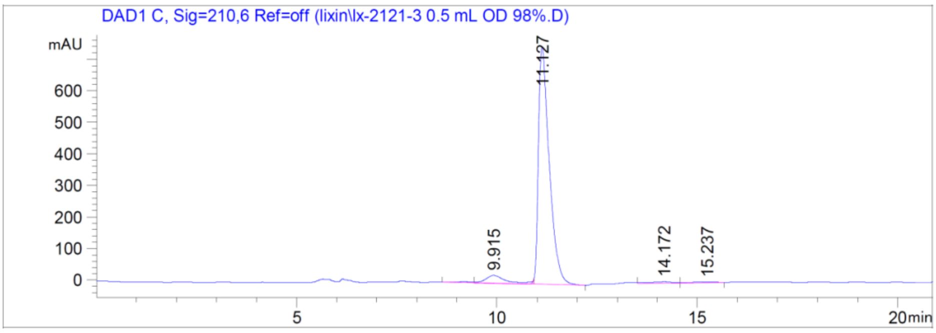

Peak #	RetTime [min]	Type	Width [min]	Area [mAU*s]	Height [mAU]	Area %
1	9.915	VV E	0.5352	930.60742	25.17933	6.0059
2	11.127	VB R	0.2844	1.42488e4	749.33350	91.9573
3	14.172	VV	0.5725	226.44160	5.19516	1.4614
4	15.237	VB	0.7503	89.15880	1.58974	0.5754

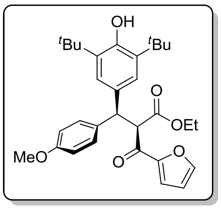

OH
tBu
tBu
O
OEt
MeO
O
O

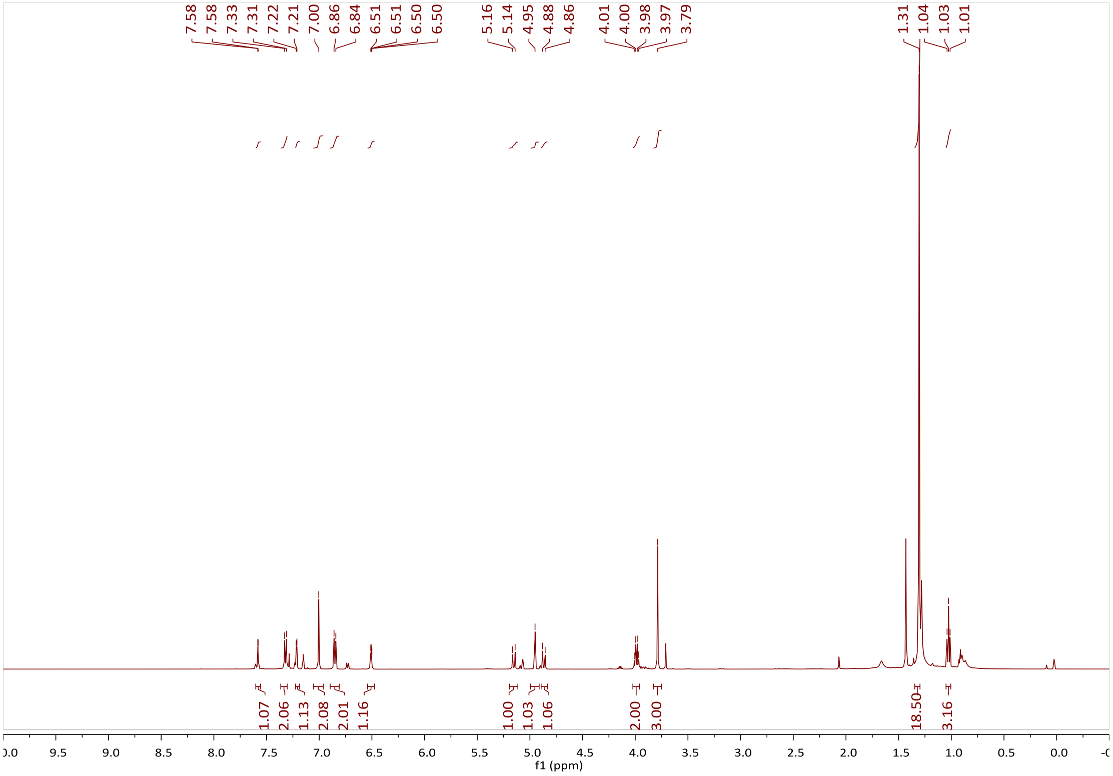

7.58
7.58
7.33
7.31
7.22
7.21
7.00
6.86
6.84
6.51
6.51
6.50
6.50
5.16
5.14
4.95
4.88
4.86
4.01
4.00
3.98
3.97
3.79
1.31
1.04
1.03
1.01
1.07
2.06
1.13
2.08
2.01
1.16
1.00
1.03
1.06
2.00
3.00
18.50
3.16
f1 (ppm)

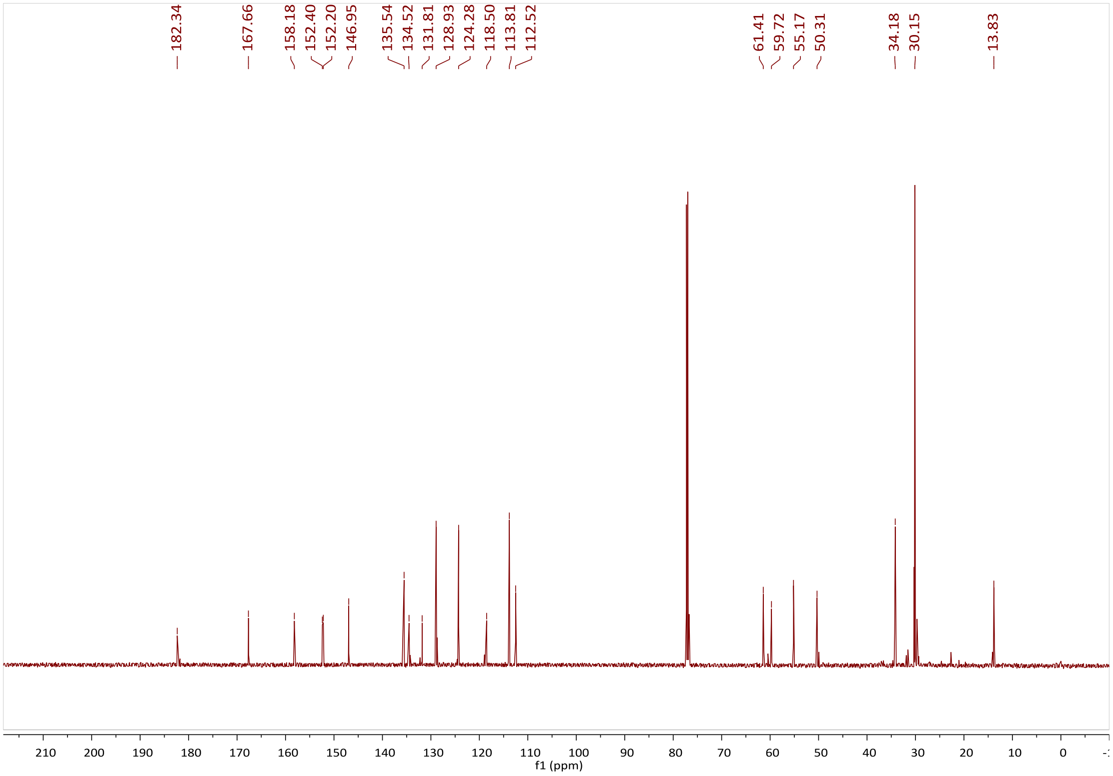

182.34
167.66
158.18
152.40
152.20
146.95
135.54
134.52
131.81
128.93
124.28
118.50
113.81
112.52
61.41
59.72
55.17
50.31
34.18
30.15
13.83
f1 (ppm)

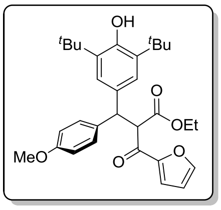

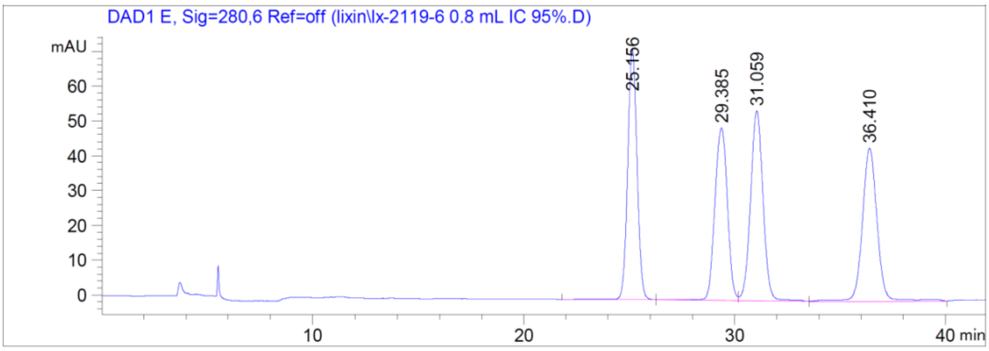

Peak #	RetTime [min]	Type	Width [min]	Area [mAU*s]	Height [mAU]	Area %
1	25.156	VB R	0.4774	2216.85913	71.80289	25.5820
2	29.385	VV R	0.6629	2047.67358	49.49503	23.6296
3	31.059	VB	0.6338	2251.16431	54.50805	25.9779
4	36.410	BV R	0.7516	2149.99976	43.91474	24.8105

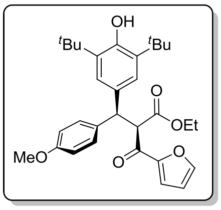

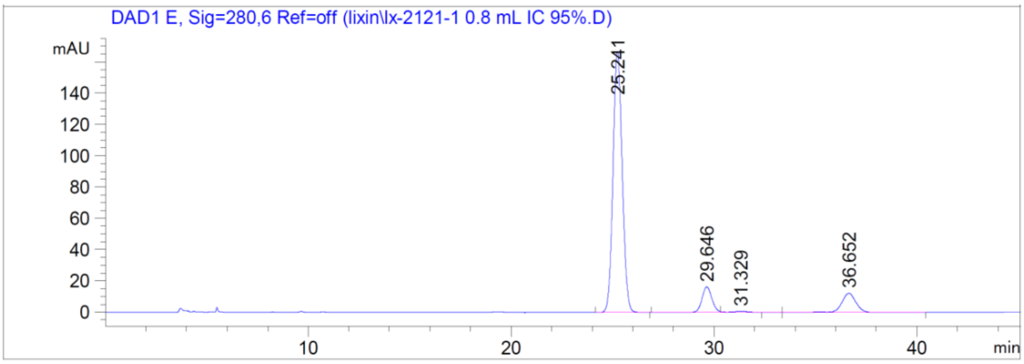

Peak #	RetTime [min]	Type	Width [min]	Area [mAU*s]	Height [mAU]	Area %
1	25.241	BB	0.4876	5259.70215	166.91177	81.0169
2	29.646	VV R	0.5312	569.17609	16.34356	8.7672
3	31.329	VV E	0.6921	42.69526	8.95732e-1	0.6576
4	36.652	VV R	0.7285	620.52881	12.29725	9.5582

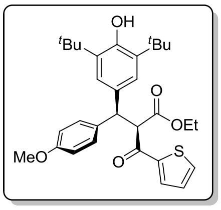
OH
tBu
tBu
O
OEt
MeO
O
S

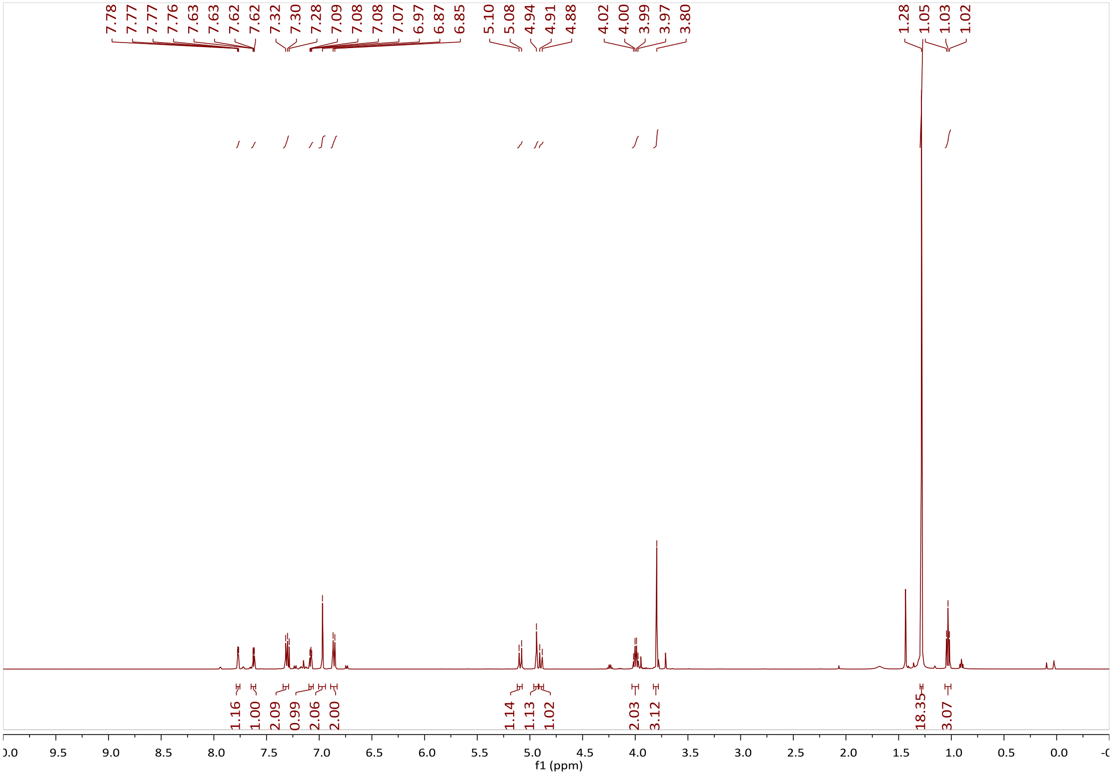
f1 (ppm)

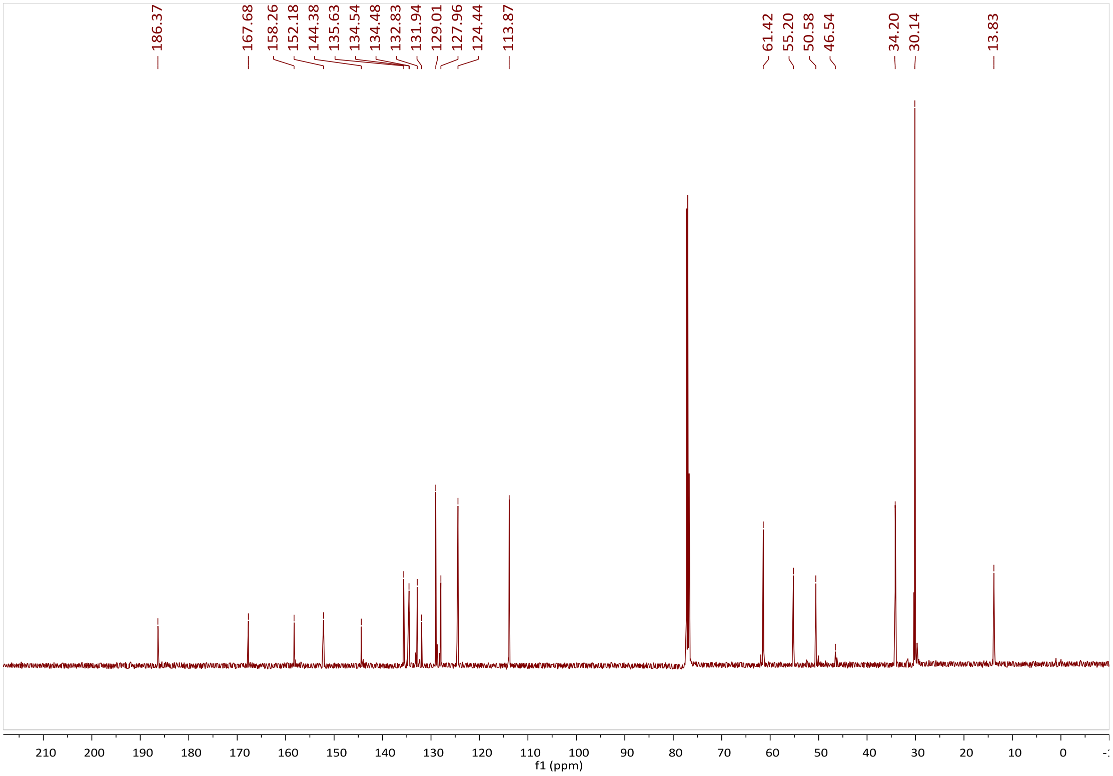
186.37
167.68
158.26
152.18
144.38
135.63
134.54
134.48
132.83
131.94
129.01
127.96
124.44
113.87
61.42
55.20
50.58
46.54
34.20
30.14
13.83
f1 (ppm)

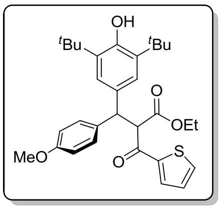

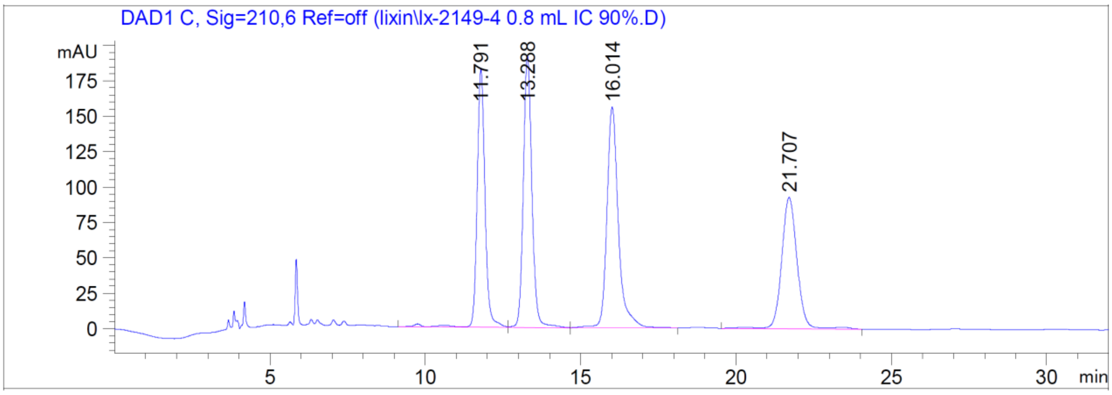

Peak #	RetTime [min]	Type	Width [min]	Area [mAU*s]	Height [mAU]	Area %
1	11.791	VV R	0.2727	3244.83936	181.34914	22.8505
2	13.288	VB	0.2960	3688.12354	192.08798	25.9722
3	16.014	BV R	0.3908	4039.87671	155.67432	28.4493
4	21.707	VV R	0.5341	3227.43701	92.72589	22.7280

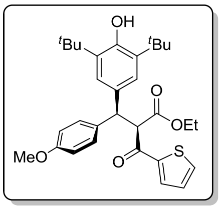

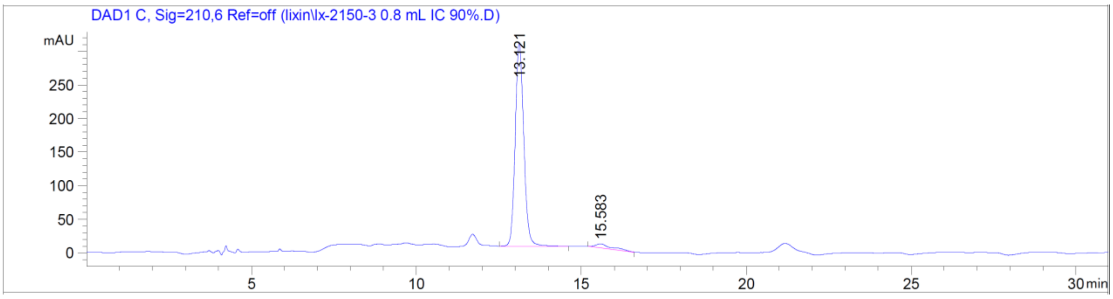

Peak #	RetTime [min]	Type	Width [min]	Area [mAU*s]	Height [mAU]	Area %
1	13.121	BB	0.2783	5458.13770	302.85736	96.4085
2	15.583	BB	0.4814	203.33443	5.88032	3.5915

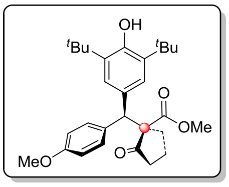
OH
tBu
tBu
O
OMe
O
MeO

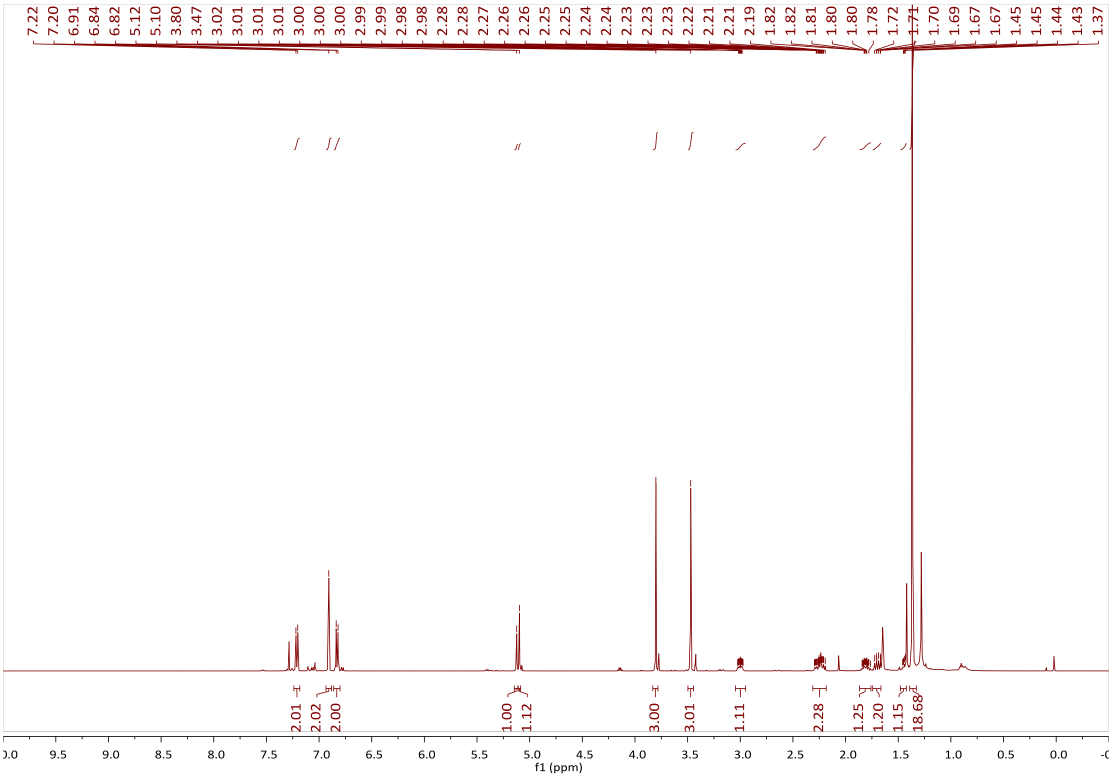
7.22 7.20 6.91 6.84 6.82 5.12 5.10 3.80 3.47 3.02 3.01 3.01 3.01 3.00 3.00 3.00 2.99 2.99 2.98 2.98 2.28 2.28 2.27 2.26 2.26 2.25 2.25 2.24 2.24 2.23 2.23 2.23 2.23 2.22 2.21 2.21 2.19 1.82 1.82 1.81 1.80 1.80 1.78 1.72 1.71 1.70 1.69 1.67 1.67 1.45 1.45 1.44 1.43 1.37
2.01 2.02 2.00 1.00 1.12 3.00 3.01 1.11 2.28 1.25 1.20 1.15 18.68
f1 (ppm)

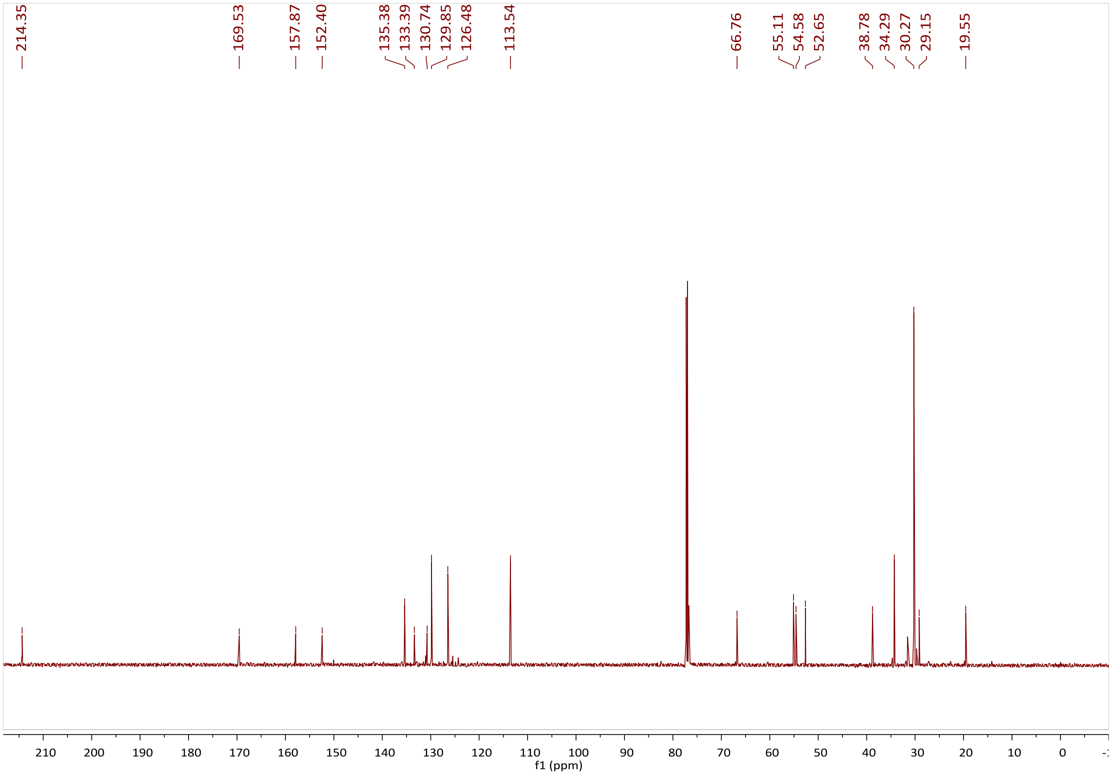
214.35 169.53 157.87 152.40 135.38 133.39 130.74 129.85 126.48 113.54 66.76 55.11 54.58 52.65 38.78 34.29 30.27 29.15 19.55
f1 (ppm)

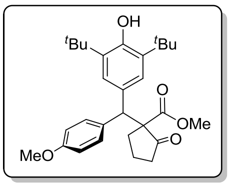

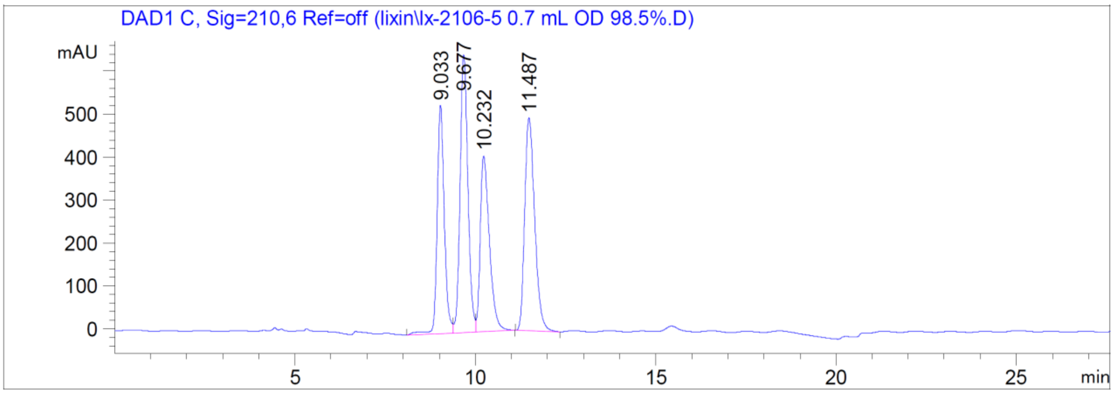

Peak #	RetTime [min]	Type	Width [min]	Area [mAU*s]	Height [mAU]	Area %
1	9.033	BV	0.2098	7346.66553	529.58002	21.7026
2	9.677	VV	0.2408	9741.04004	642.86279	28.7758
3	10.232	VB	0.2703	7230.87354	408.89154	21.3606
4	11.487	BB	0.3048	9532.89551	494.76224	28.1609

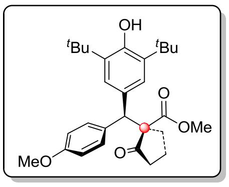

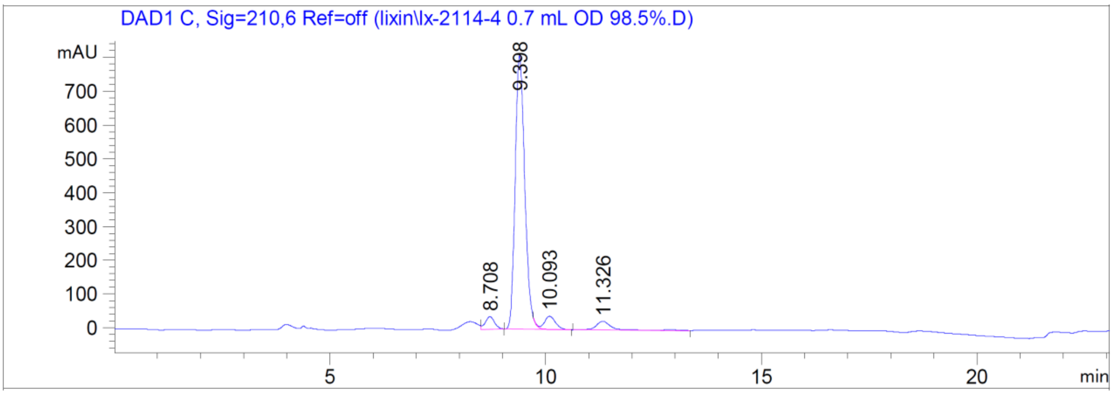

Peak #	RetTime [min]	Type	Width [min]	Area [mAU*s]	Height [mAU]	Area %
1	8.708	VB	0.2304	554.89728	37.12251	3.8228
2	9.398	BV R	0.2460	1.25797e4	806.24609	86.6641
3	10.093	VB E	0.2798	698.29895	39.19778	4.8107
4	11.326	BV R	0.3603	682.57336	25.95677	4.7024

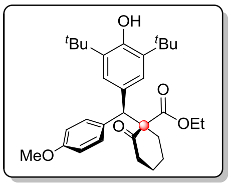
OH
tBu
tBu
O
OEt
O
MeO

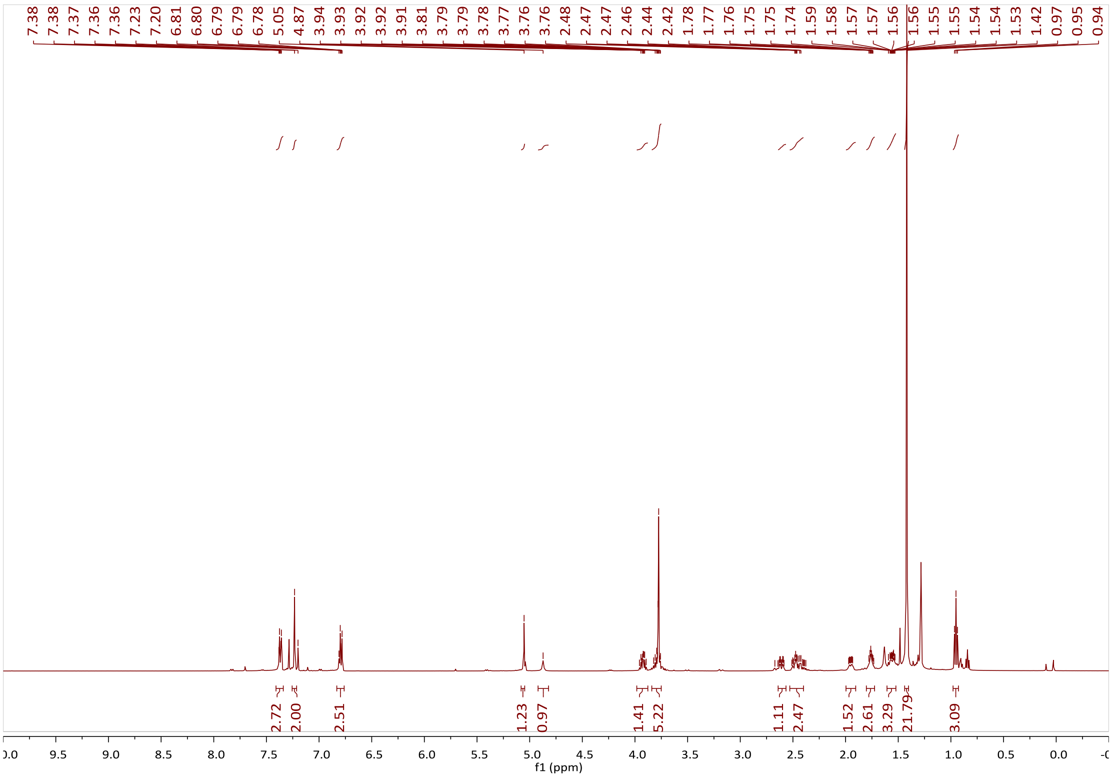
7.38 7.38 7.37 7.36 7.36 7.23 7.20 6.81 6.80 6.79 6.79 6.78 5.05 4.87 3.94 3.93 3.92 3.92 3.91 3.81 3.79 3.79 3.78 3.77 3.76 3.76 2.48 2.47 2.47 2.46 2.44 2.42 1.78 1.77 1.76 1.75 1.75 1.74 1.59 1.58 1.57 1.57 1.56 1.56 1.55 1.55 1.54 1.54 1.53 1.42 0.97 0.95 0.94
2.72 2.00 2.51 1.23 0.97 1.41 5.22 1.11 2.47 1.52 2.61 3.29 21.79 3.09
9.5 9.0 8.5 8.0 7.5 7.0 6.5 6.0 5.5 5.0 4.5 4.0 3.5 3.0 2.5 2.0 1.5 1.0 0.5 0.0
f1 (ppm)

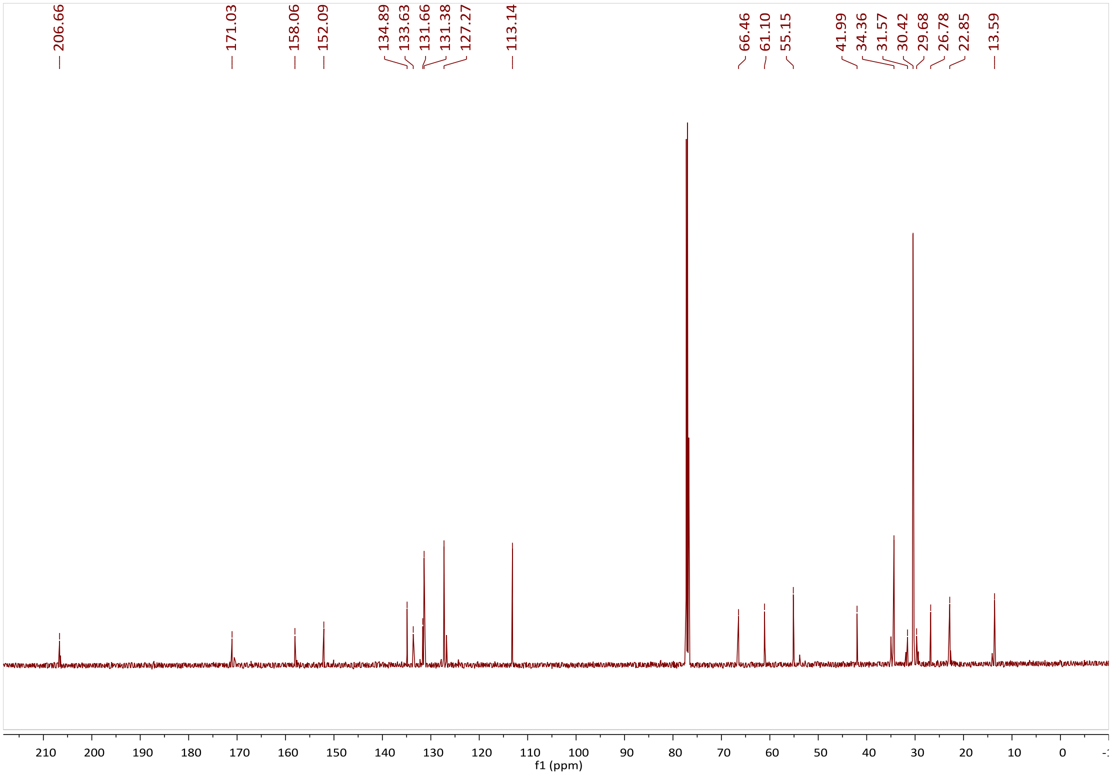
206.66 171.03 158.06 152.09 134.89 133.63 131.66 131.38 127.27 113.14 66.46 61.10 55.15 41.99 34.36 31.57 30.42 29.68 26.78 22.85 13.59
210 200 190 180 170 160 150 140 130 120 110 100 90 80 70 60 50 40 30 20 10 0
f1 (ppm)

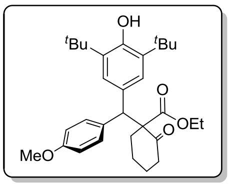

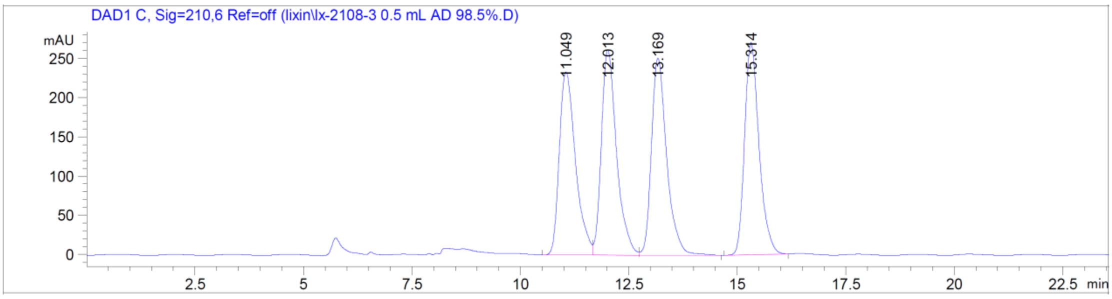

Peak #	RetTime [min]	Type	Width [min]	Area [mAU*s]	Height [mAU]	Area %
1	11.049	BV	0.4003	6059.26465	233.83890	24.3556
2	12.013	VV	0.3727	6401.92188	262.30228	25.7329
3	13.169	VB	0.3757	6157.46387	251.48016	24.7503
4	15.314	BB	0.3537	6259.67236	270.82025	25.1612

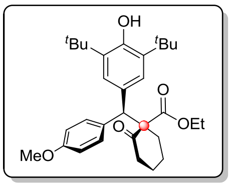

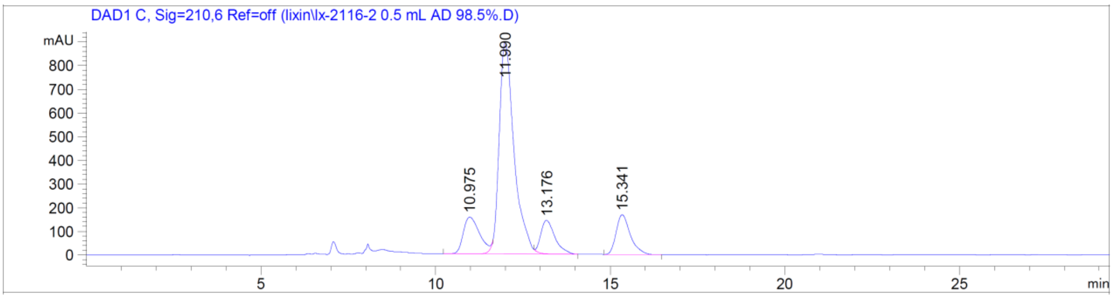

Peak #	RetTime [min]	Type	Width [min]	Area [mAU*s]	Height [mAU]	Area %
1	10.975	BV E	0.5033	4999.48633	154.58099	12.3626
2	11.990	VV R	0.4555	2.67411e4	892.58423	66.1247
3	13.176	VB E	0.4261	3943.36963	141.93524	9.7510
4	15.341	BB	0.4327	4756.49756	167.81361	11.7617

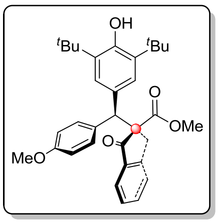
OH
tBu
tBu
O
OMe
O
MeO

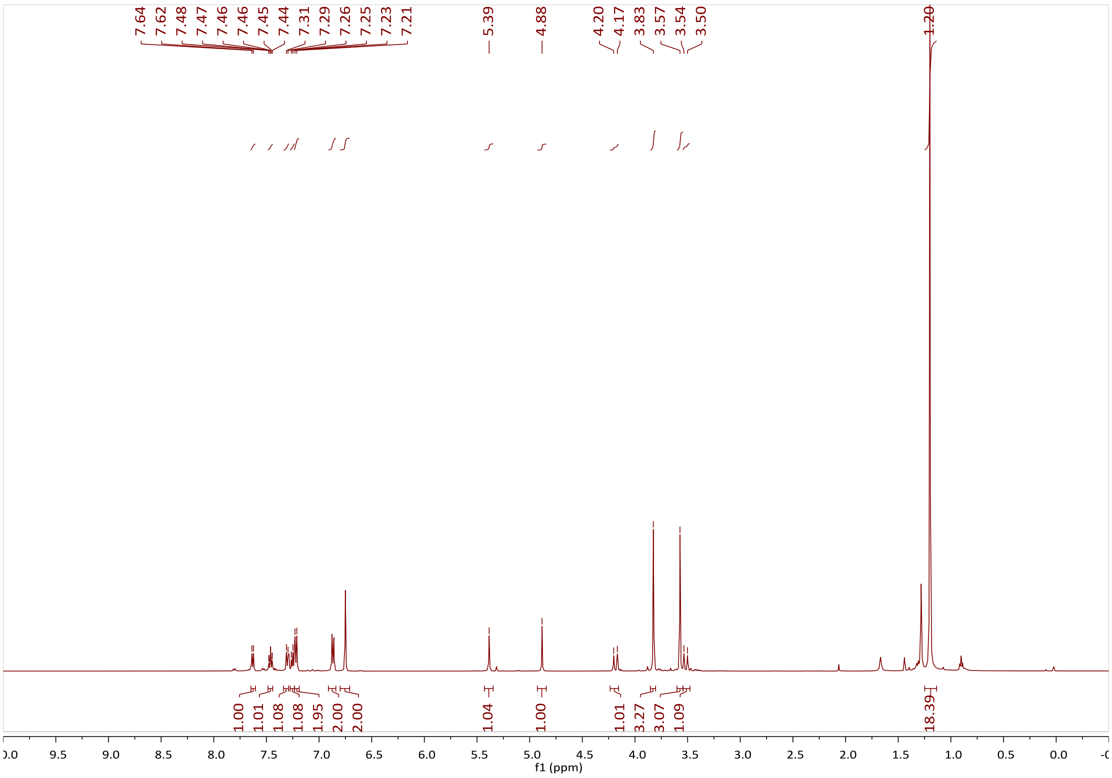
7.64
7.62
7.48
7.47
7.46
7.45
7.44
7.31
7.29
7.26
7.25
7.23
7.21
5.39
4.88
4.20
4.17
3.83
3.57
3.54
3.50
1.20
1.00
1.01
1.08
1.08
1.95
2.00
2.00
1.04
1.00
1.01
3.27
3.07
1.09
18.39
f1 (ppm)

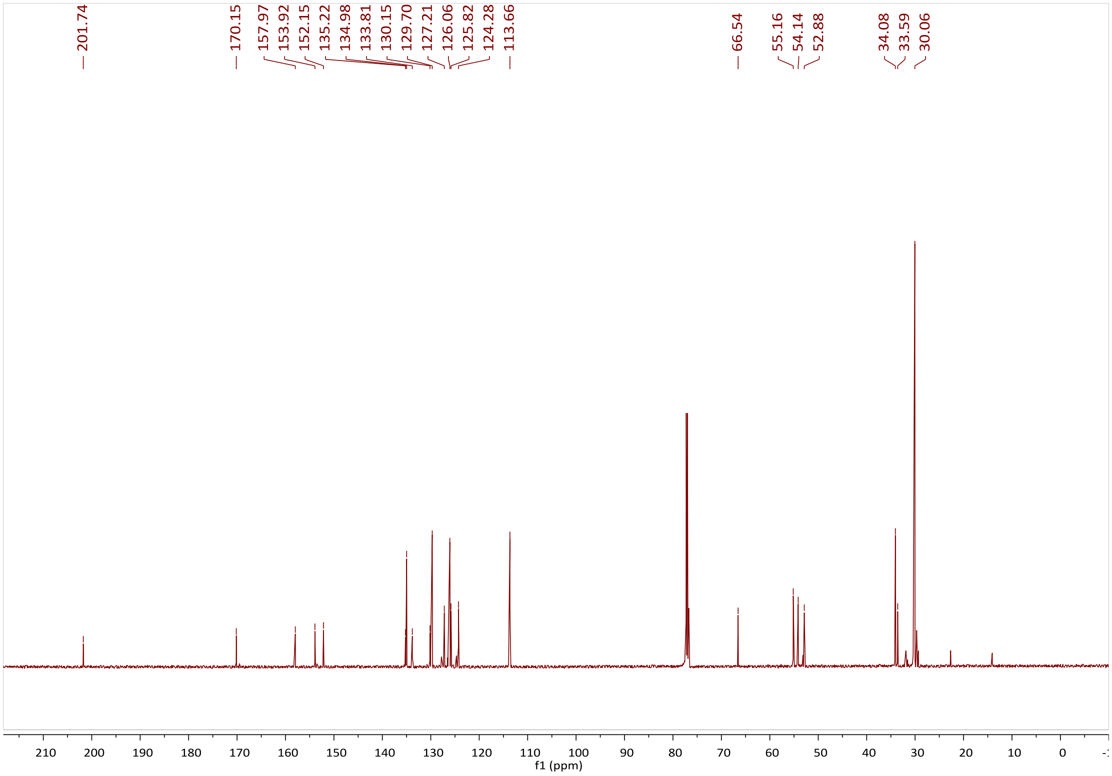
201.74
170.15
157.97
153.92
152.15
135.22
134.98
133.81
130.15
129.70
127.21
126.06
125.82
124.28
113.66
66.54
55.16
54.14
52.88
34.08
33.59
30.06
f1 (ppm)

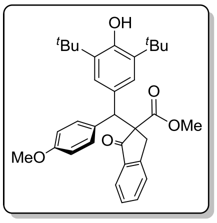

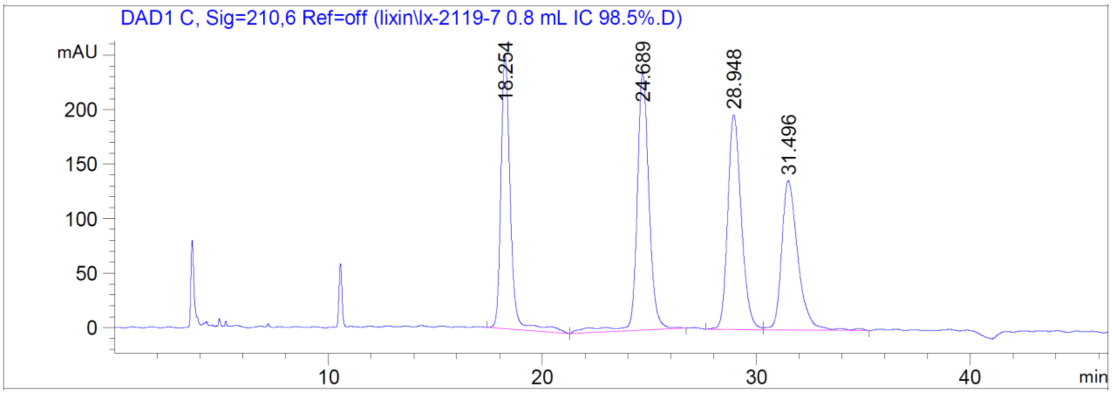

Peak #	RetTime [min]	Type	Width [min]	Area [mAU*s]	Height [mAU]	Area %
1	18.254	BV R	0.4534	7784.59326	251.17899	23.5461
2	24.689	VV R	0.5823	9371.14063	236.10056	28.3450
3	28.948	BV	0.6818	8664.12500	196.72520	26.2064
4	31.496	VV R	0.7931	7241.19922	136.72217	21.9025

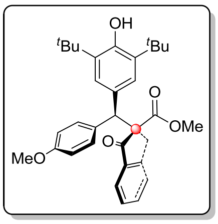

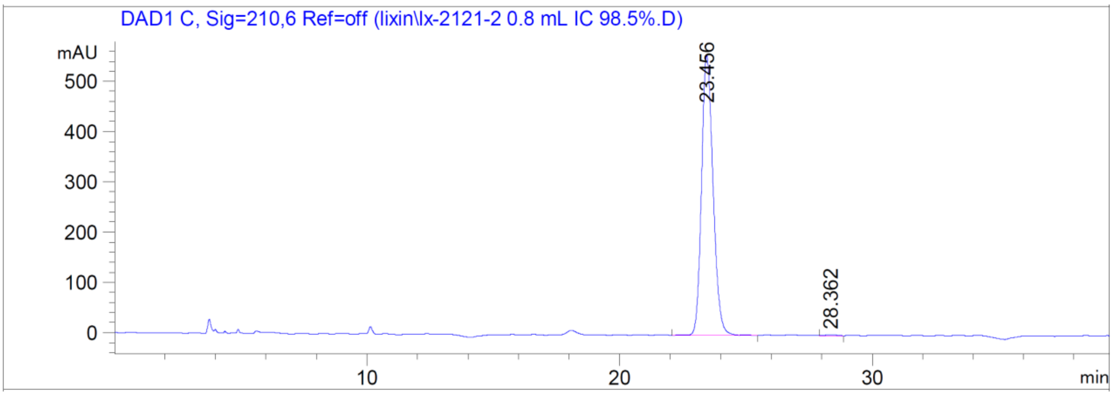

Peak #	RetTime [min]	Type	Width [min]	Area [mAU*s]	Height [mAU]	Area %
1	23.456	VV R	0.4898	1.76296e4	556.12042	99.5860
2	28.362	VV	0.5225	73.29155	2.30813	0.4140

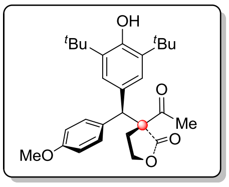
OH
tBu
tBu
O
Me
O
O
MeO

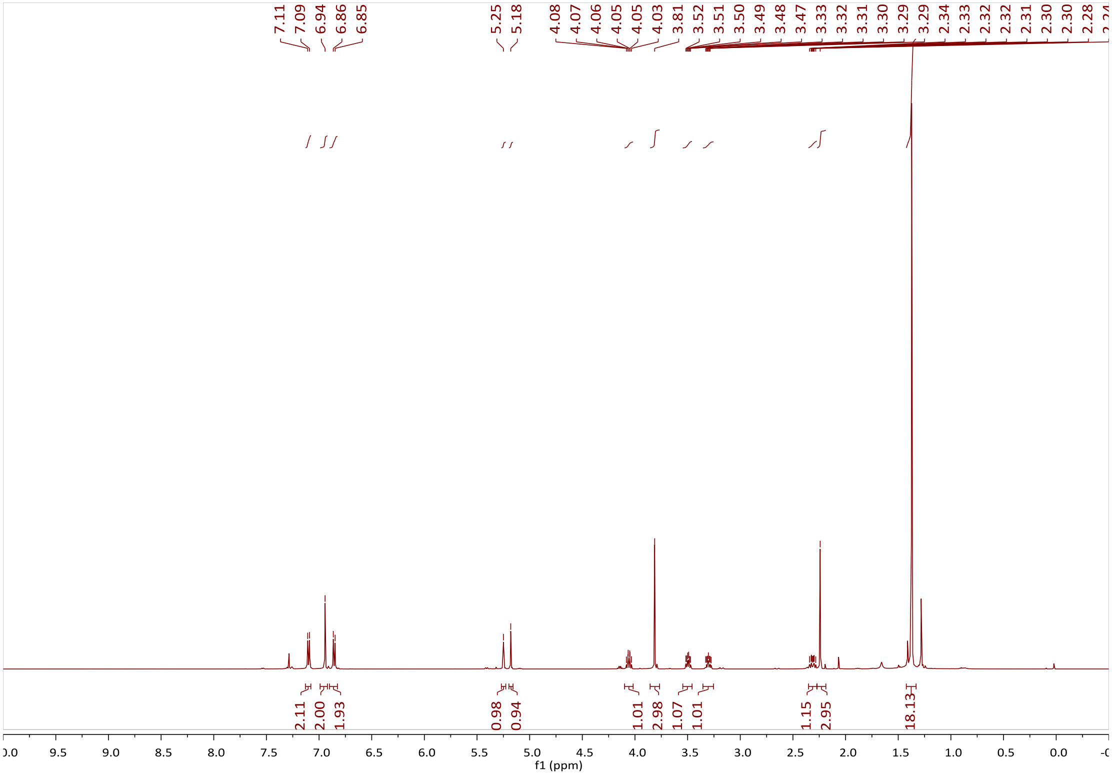

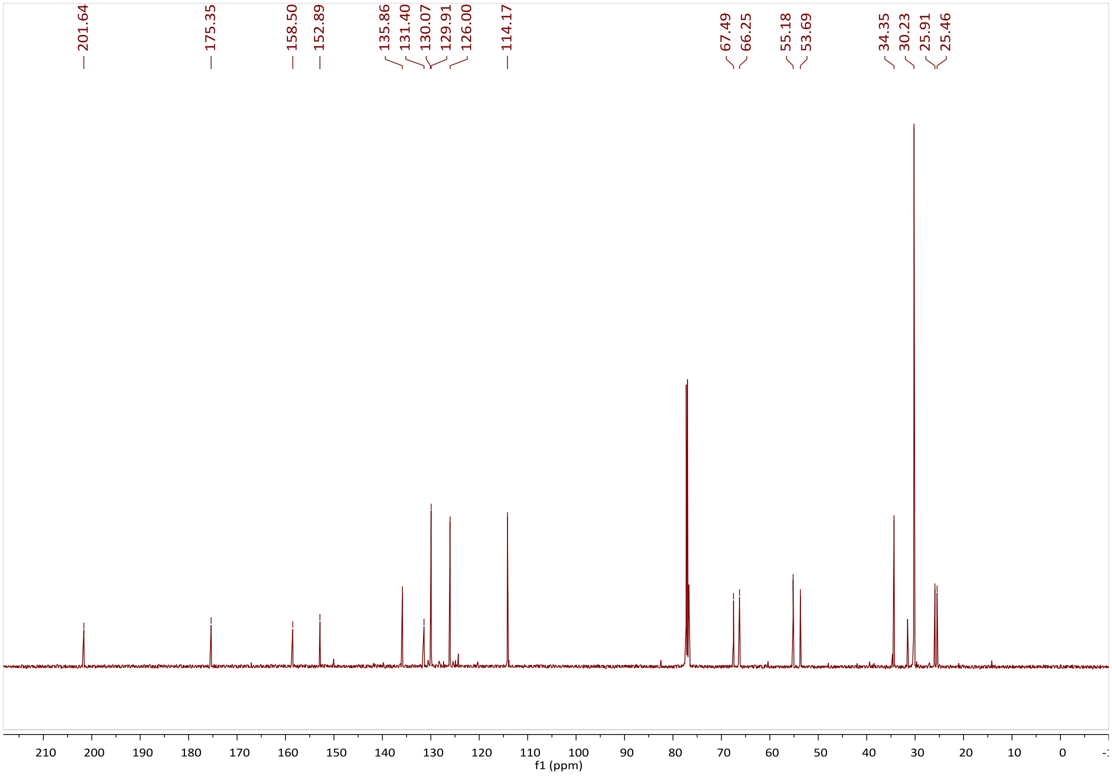

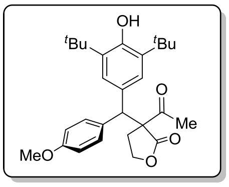

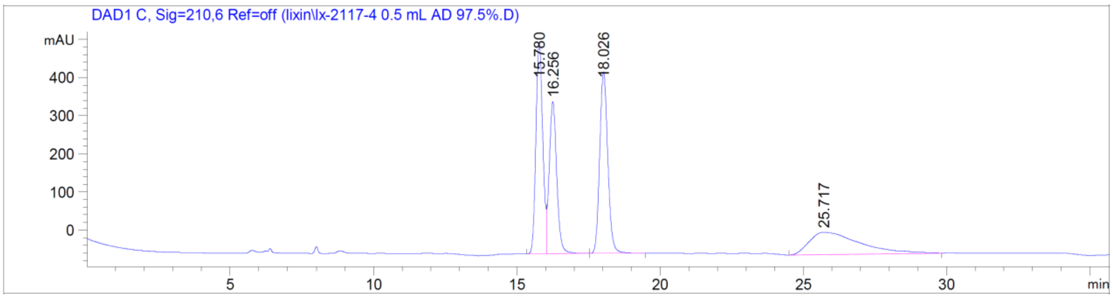

Peak #	RetTime [min]	Type	Width [min]	Area [mAU*s]	Height [mAU]	Area %
1	15.780	BV	0.2566	9148.44336	554.07855	27.8570
2	16.256	VB	0.2764	7250.90137	398.22513	22.0790
3	18.026	BB	0.3031	9349.63867	476.18875	28.4696
4	25.717	BB	1.5779	7091.76660	58.10468	21.5944

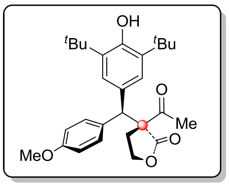

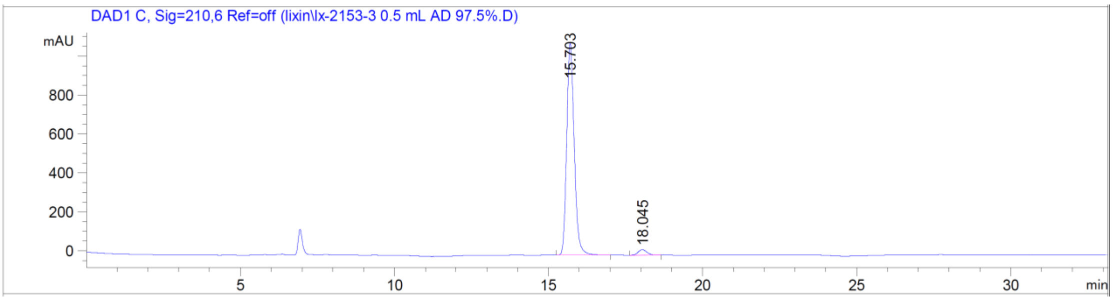

Peak #	RetTime [min]	Type	Width [min]	Area [mAU*s]	Height [mAU]	Area %
1	15.703	BB	0.2607	1.83171e4	1086.64856	97.0229
2	18.045	BB	0.3073	562.05084	28.35166	2.9771

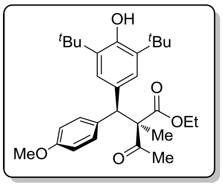
OH
tBu
tBu
O
OEt
Me
MeO
O
Me

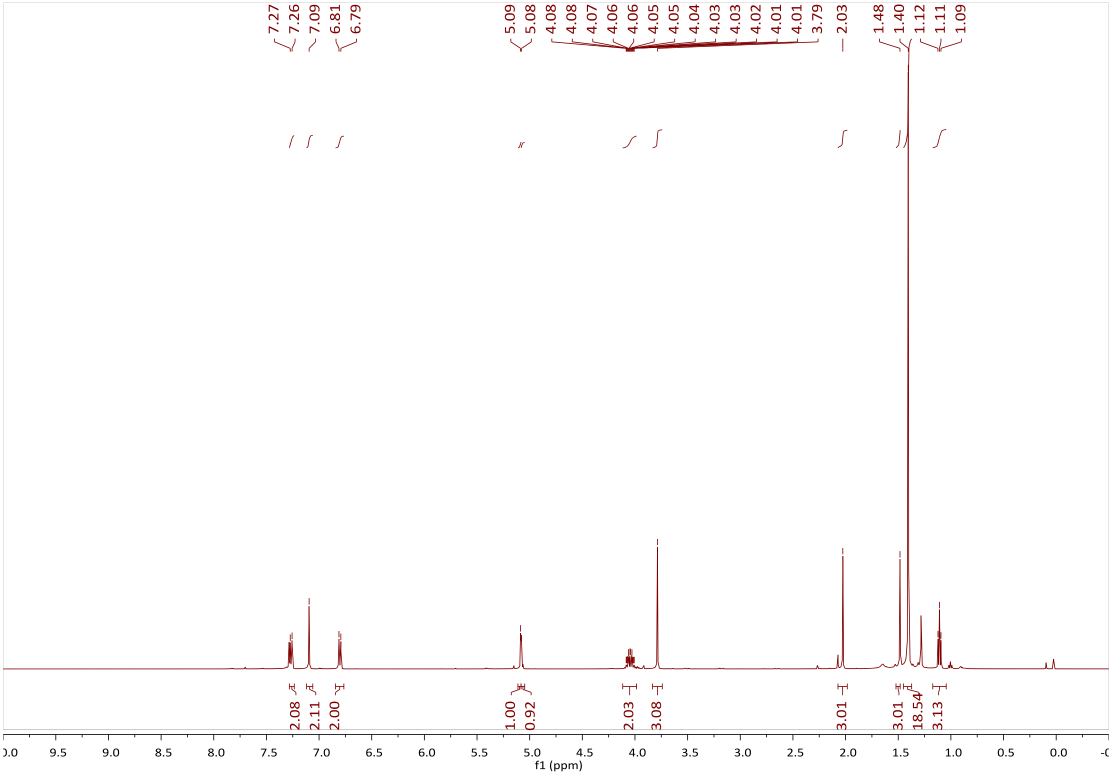
7.27
7.26
7.09
6.81
6.79
5.09
5.08
4.08
4.08
4.07
4.06
4.06
4.05
4.05
4.04
4.03
4.03
4.02
4.01
4.01
3.79
2.03
1.48
1.40
1.12
1.11
1.09
2.08
2.11
2.00
1.00
0.92
2.03
3.08
3.01
3.01
18.54
3.13
9.5
9.0
8.5
8.0
7.5
7.0
6.5
6.0
5.5
5.0
4.5
4.0
3.5
3.0
2.5
2.0
1.5
1.0
0.5
0.0
f1 (ppm)

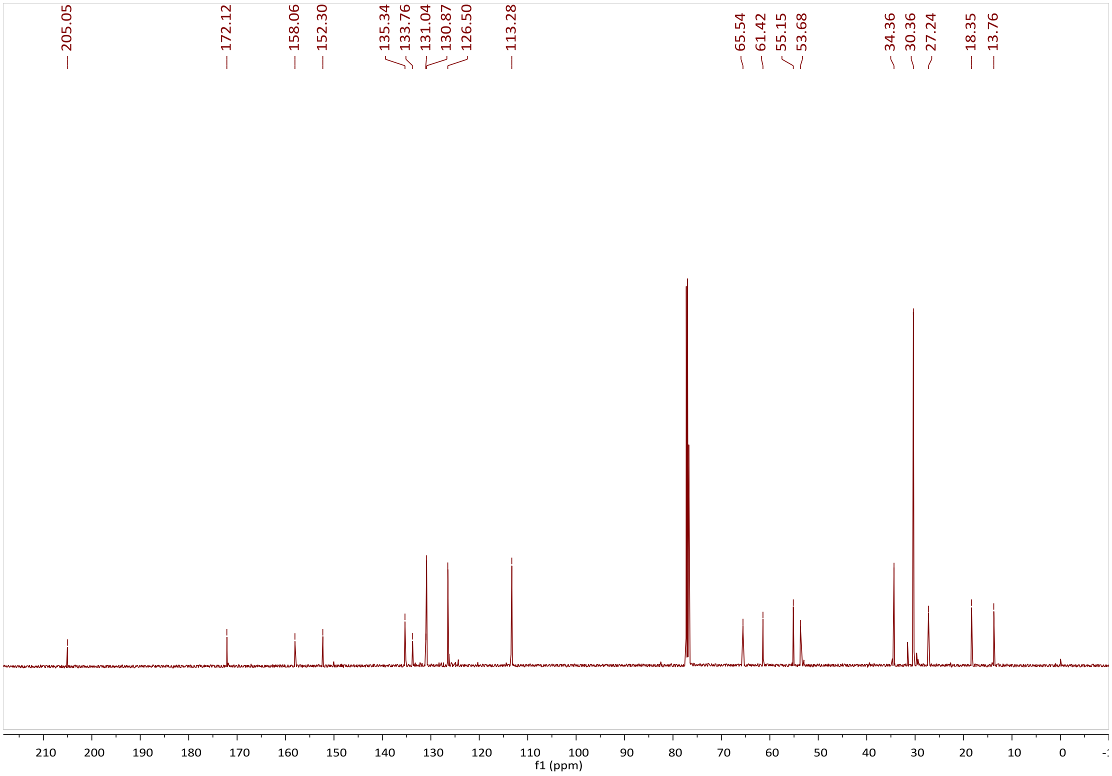
205.05
172.12
158.06
152.30
135.34
133.76
131.04
130.87
126.50
113.28
65.54
61.42
55.15
53.68
34.36
30.36
27.24
18.35
13.76
210
200
190
180
170
160
150
140
130
120
110
100
90
80
70
60
50
40
30
20
10
0
f1 (ppm)

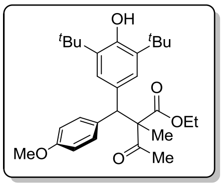

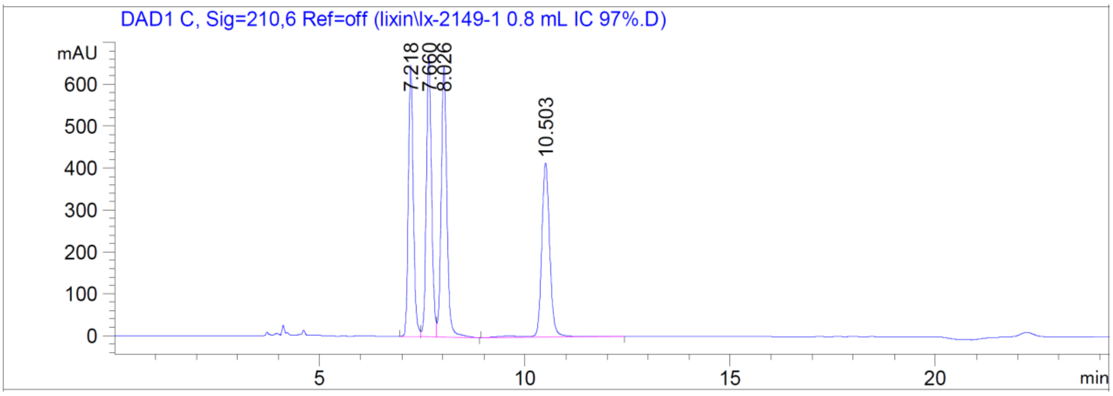

Peak #	RetTime [min]	Type	Width [min]	Area [mAU*s]	Height [mAU]	Area %
1	7.218	BV	0.1305	5422.11523	642.33398	23.0219
2	7.660	VV	0.1453	5985.21338	663.26019	25.4128
3	8.026	VB	0.1472	6280.74805	636.06702	26.6676
4	10.503	VV R	0.2078	5863.87549	414.65347	24.8976

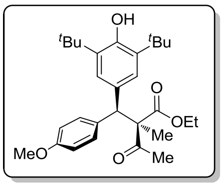

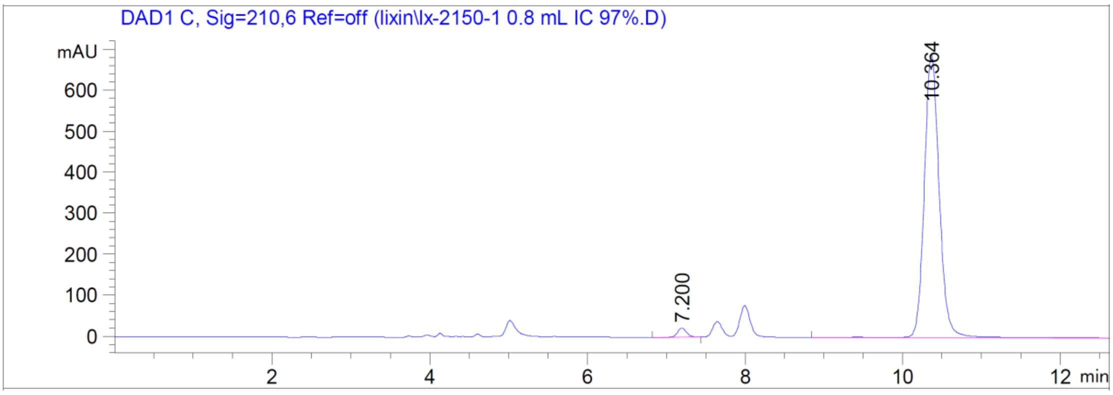

Peak #	RetTime [min]	Type	Width [min]	Area [mAU*s]	Height [mAU]	Area %
1	7.200	BB	0.1289	183.22093	22.06487	1.9377
2	10.364	VV R	0.2051	9272.56445	688.70886	98.0623

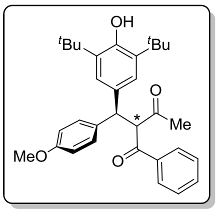

dr = 1.2:1

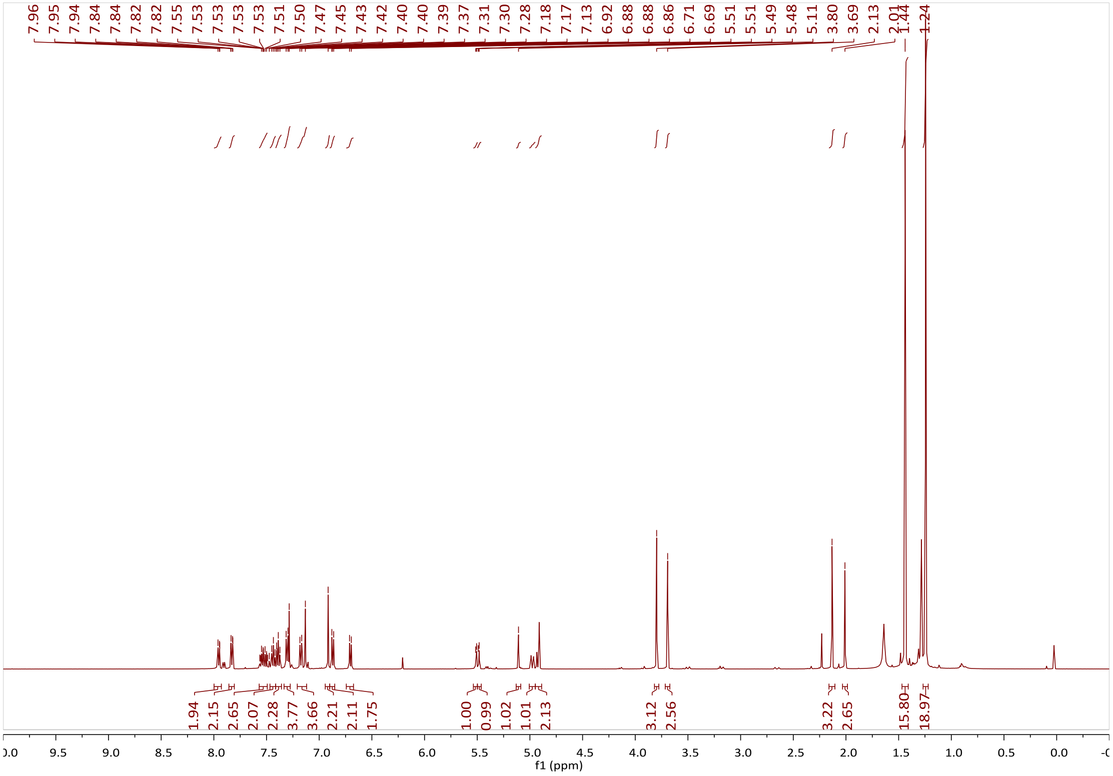

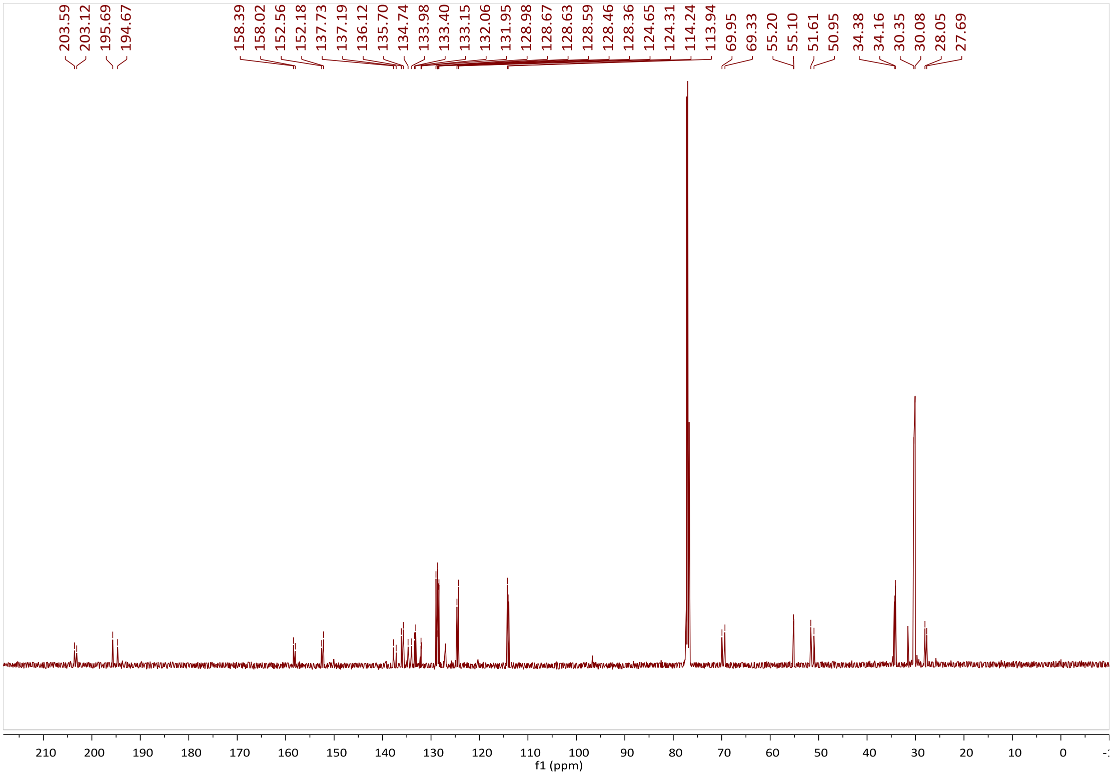

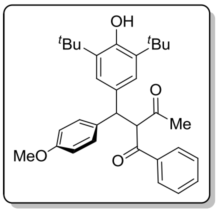

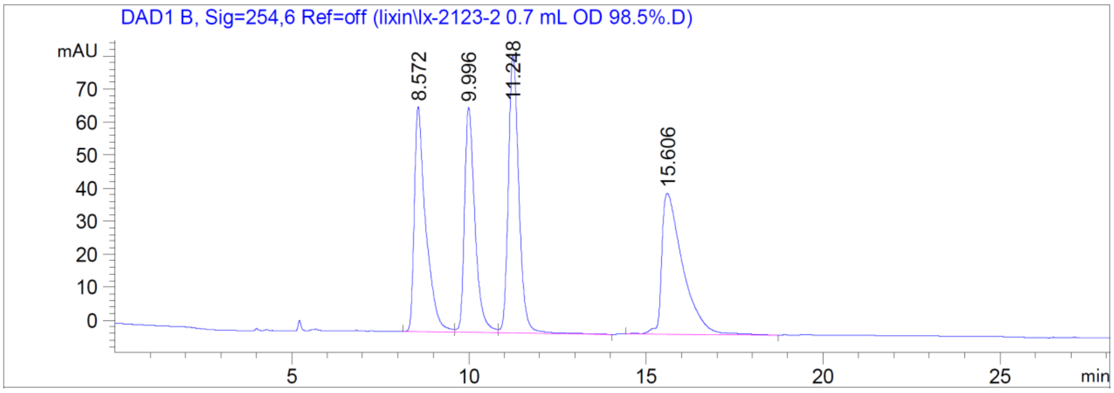

Peak #	RetTime [min]	Type	Width [min]	Area [mAU*s]	Height [mAU]	Area %
1	8.572	BV	0.3270	1531.86768	67.96443	23.7829
2	9.996	VV	0.3107	1392.34302	68.03885	21.6167
3	11.248	VB	0.3208	1740.41443	84.31390	27.0207
4	15.606	VB R	0.6035	1776.41760	42.74687	27.5797

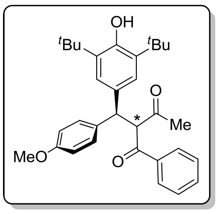

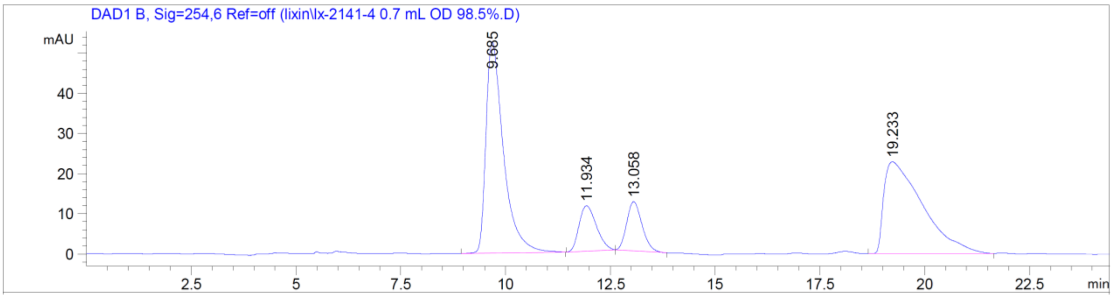

Peak #	RetTime [min]	Type	Width [min]	Area [mAU*s]	Height [mAU]	Area %
1	9.685	BB	0.4371	1531.35327	52.67975	42.0714
2	11.934	BB	0.4445	323.41205	11.28021	8.8852
3	13.058	BB	0.3906	310.82974	12.22462	8.5395
4	19.233	BB	0.8751	1474.29651	22.89889	40.5039

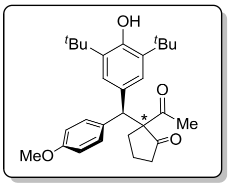

dr = 1.6:1

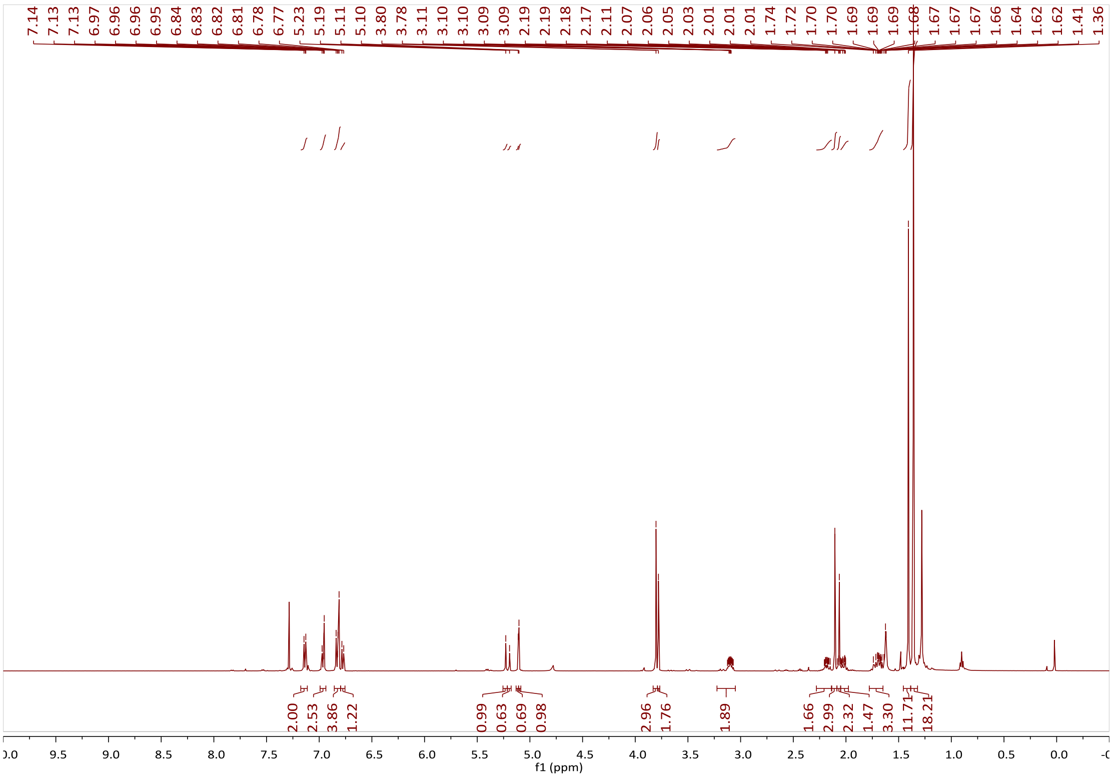

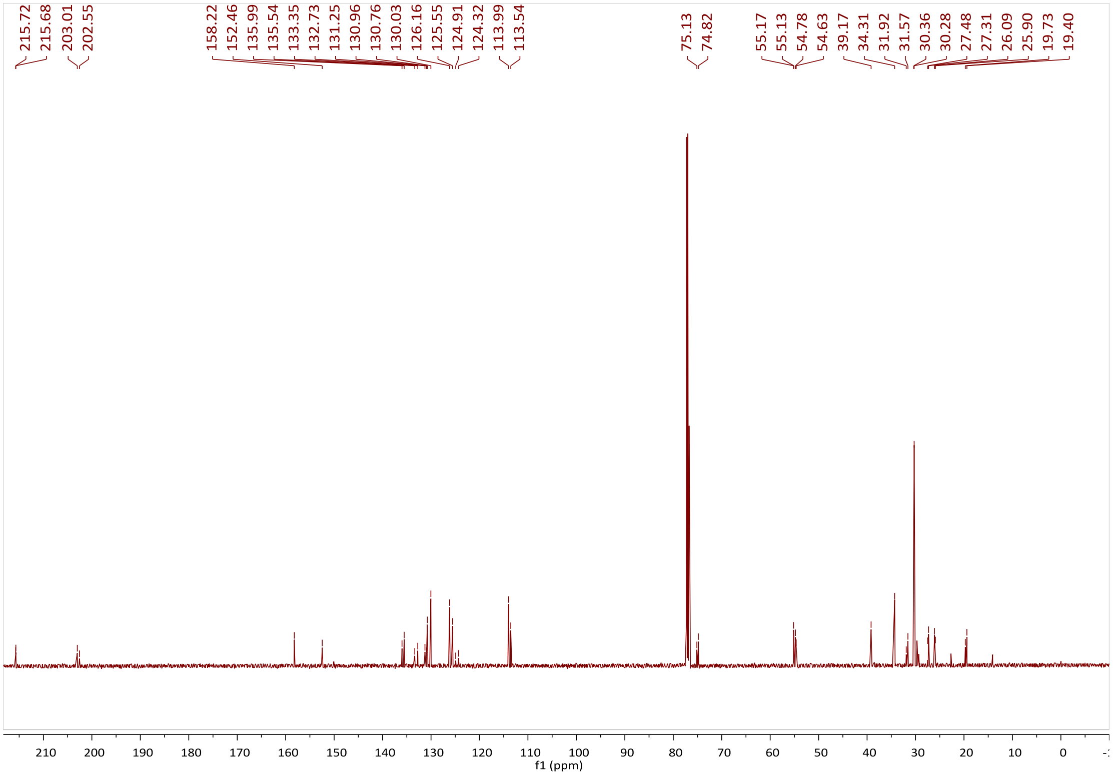

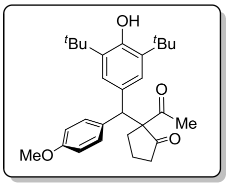

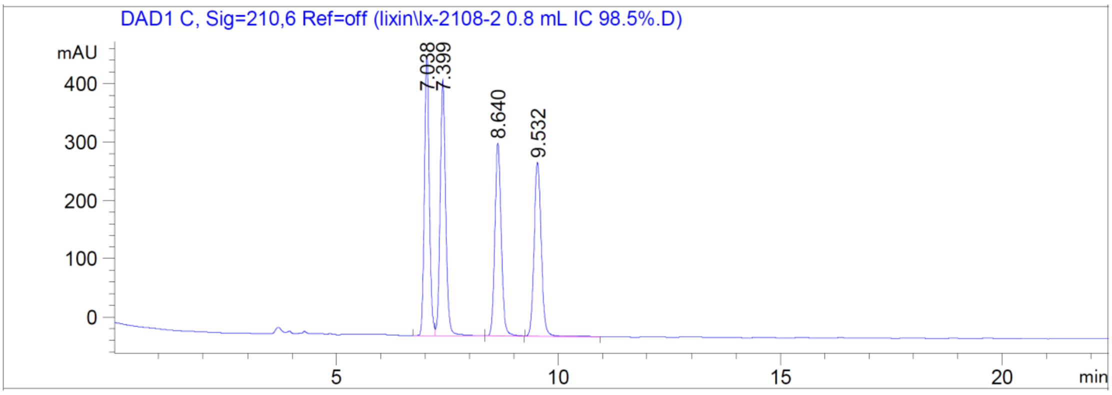

Peak #	RetTime [min]	Type	Width [min]	Area [mAU*s]	Height [mAU]	Area %
1	7.038	BV	0.1238	3716.73828	472.14755	26.2695
2	7.399	VB	0.1425	3814.45435	434.18027	26.9601
3	8.640	BB	0.1655	3297.90186	327.45908	23.3092
4	9.532	BV R	0.1710	3319.41211	295.61563	23.4612

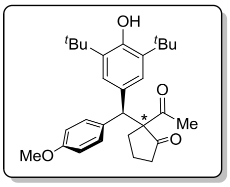

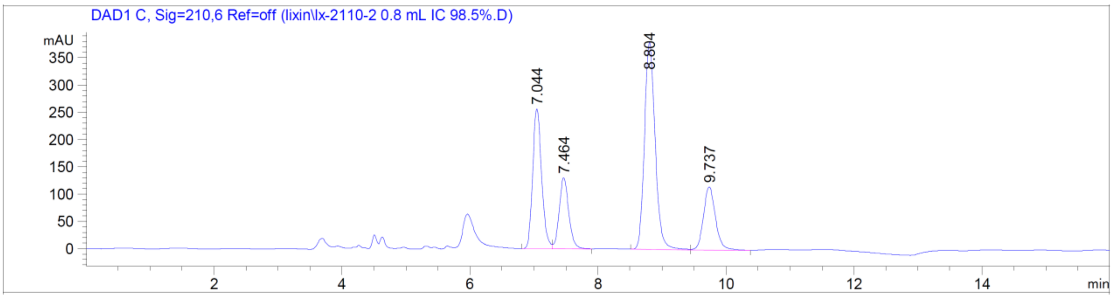

Peak #	RetTime [min]	Type	Width [min]	Area [mAU*s]	Height [mAU]	Area %
1	7.044	BV	0.1497	2496.10767	256.17279	25.5715
2	7.464	VB	0.1593	1352.56592	130.13994	13.8564
3	8.804	BV	0.1795	4407.53906	379.38504	45.1532
4	9.737	VB	0.1998	1505.09204	115.68549	15.4190

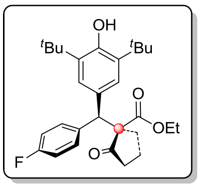

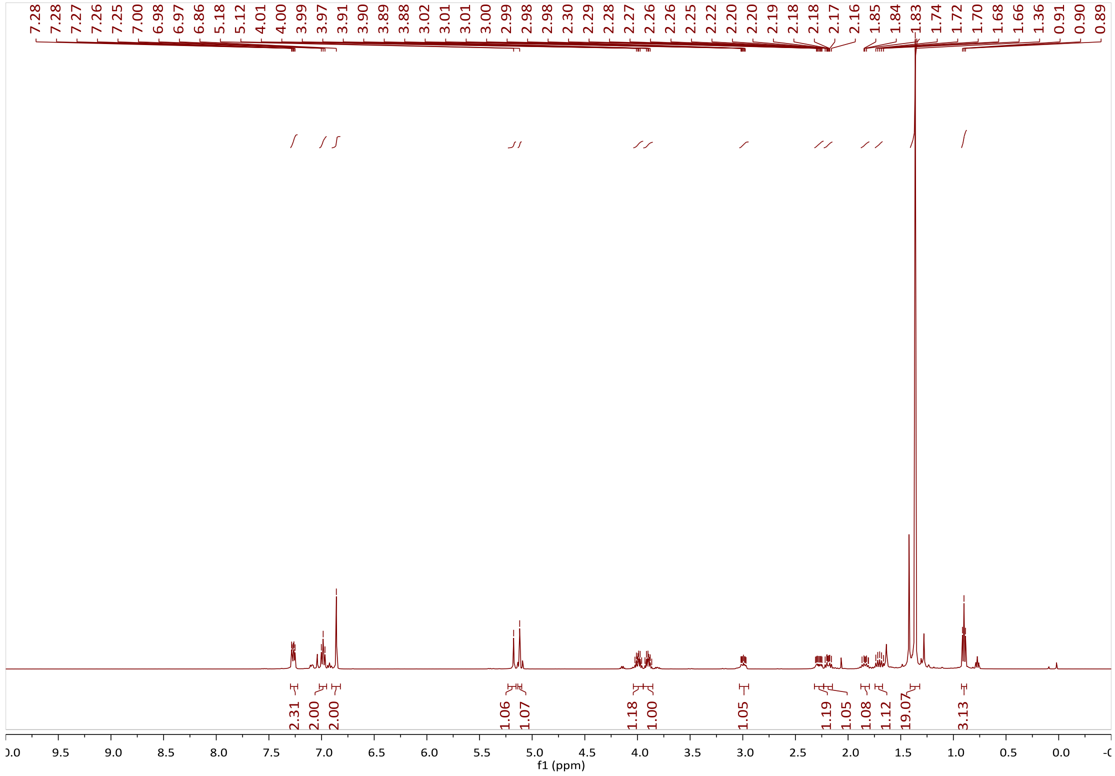

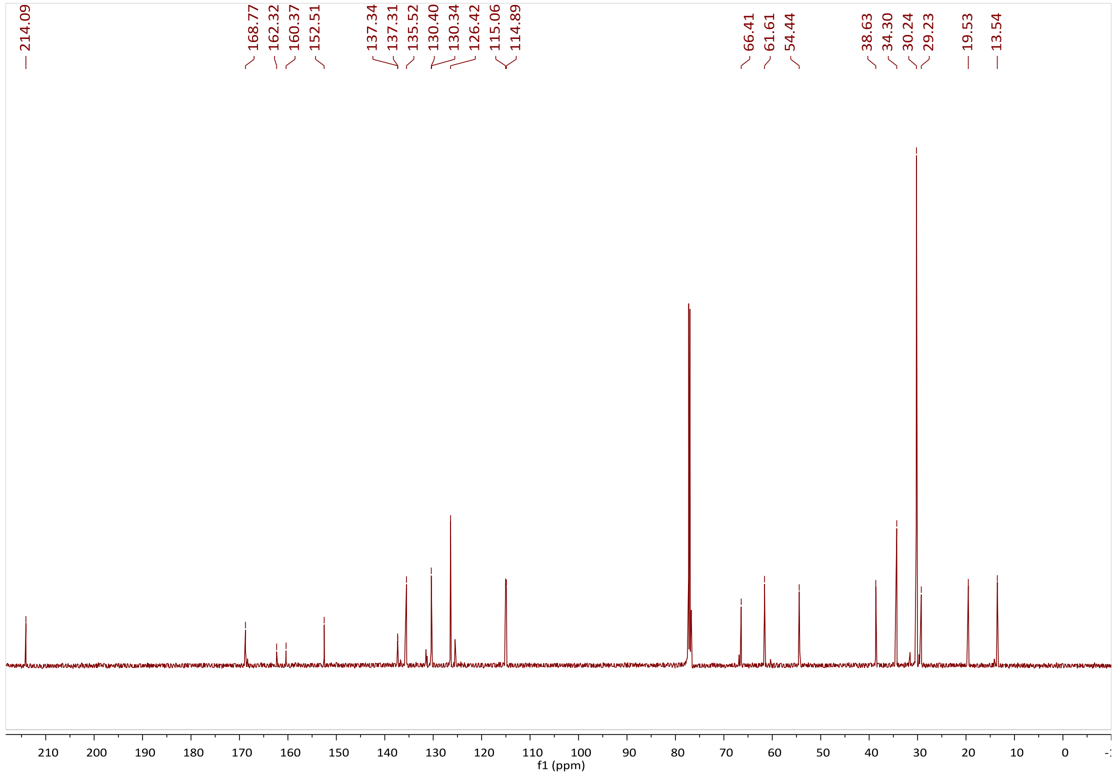

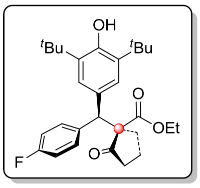
OH
tBu
tBu
O
OEt
F
O

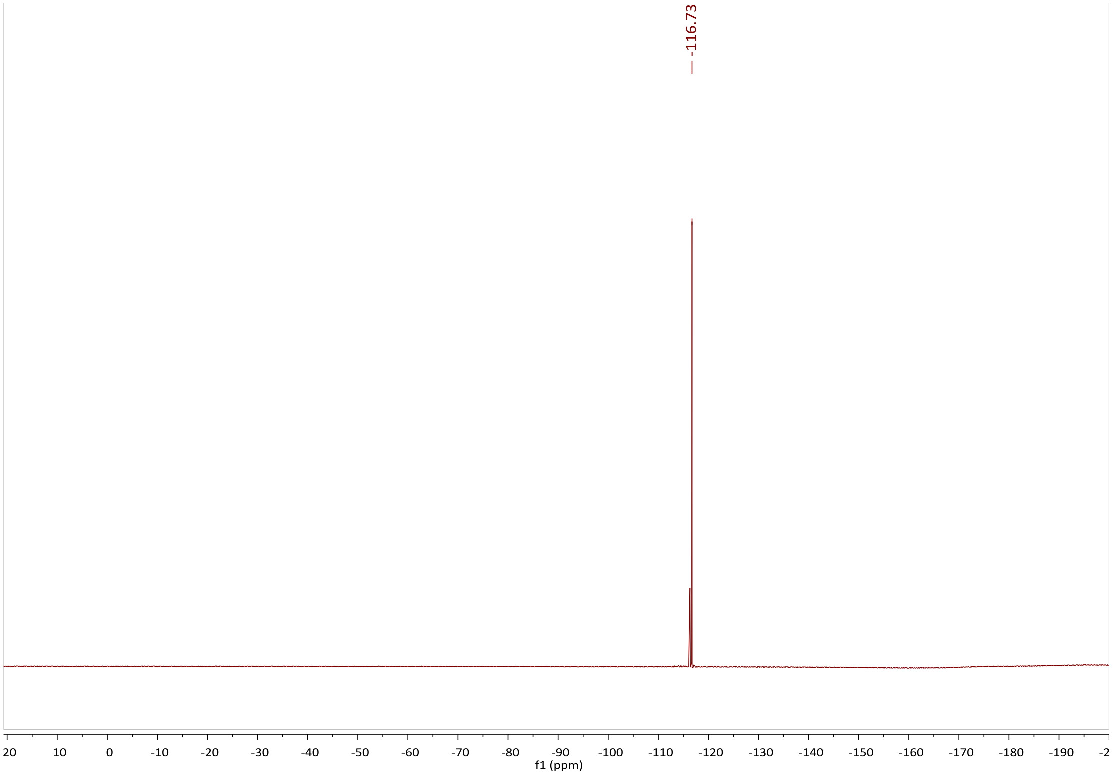
-116.73
20
10
0
-10
-20
-30
-40
-50
-60
-70
-80
-90
-100
-110
-120
-130
-140
-150
-160
-170
-180
-190
-2
f1 (ppm)

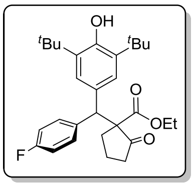

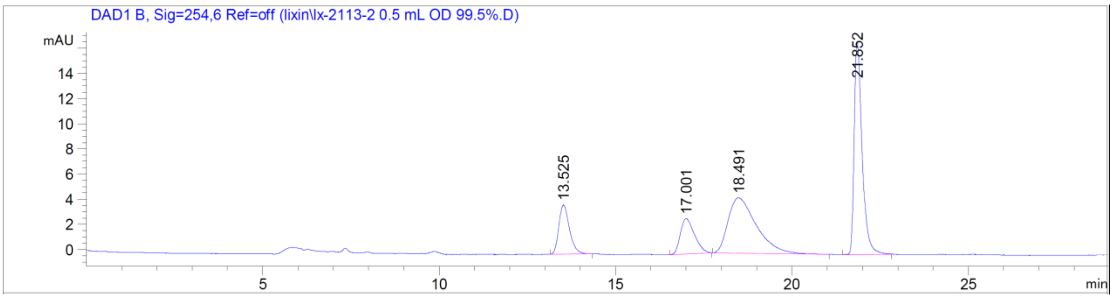

Peak #	RetTime [min]	Type	Width [min]	Area [mAU*s]	Height [mAU]	Area %
1	13.525	BB	0.3157	82.45230	3.91588	12.2556
2	17.001	BB	0.4021	78.40158	2.82229	11.6535
3	18.491	BB	0.7199	246.21324	4.40666	36.5967
4	21.852	BB	0.2419	265.70749	16.85353	39.4943

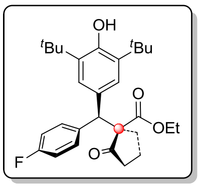

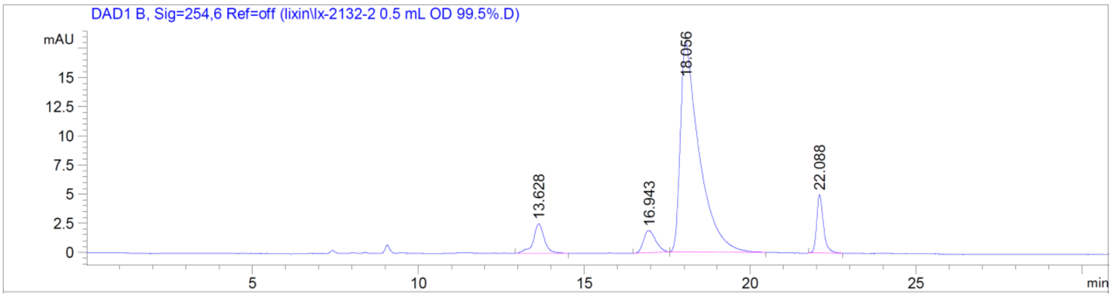

Peak #	RetTime [min]	Type	Width [min]	Area [mAU*s]	Height [mAU]	Area %
1	13.628	BB	0.3347	58.76923	2.53257	6.6019
2	16.943	BB	0.3620	49.13311	1.92379	5.5195
3	18.056	BB	0.5457	711.26233	18.05146	79.9009
4	22.088	BB	0.2148	71.01564	5.02774	7.9777

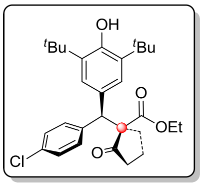
OH
tBu
tBu
O
OEt
Cl
O

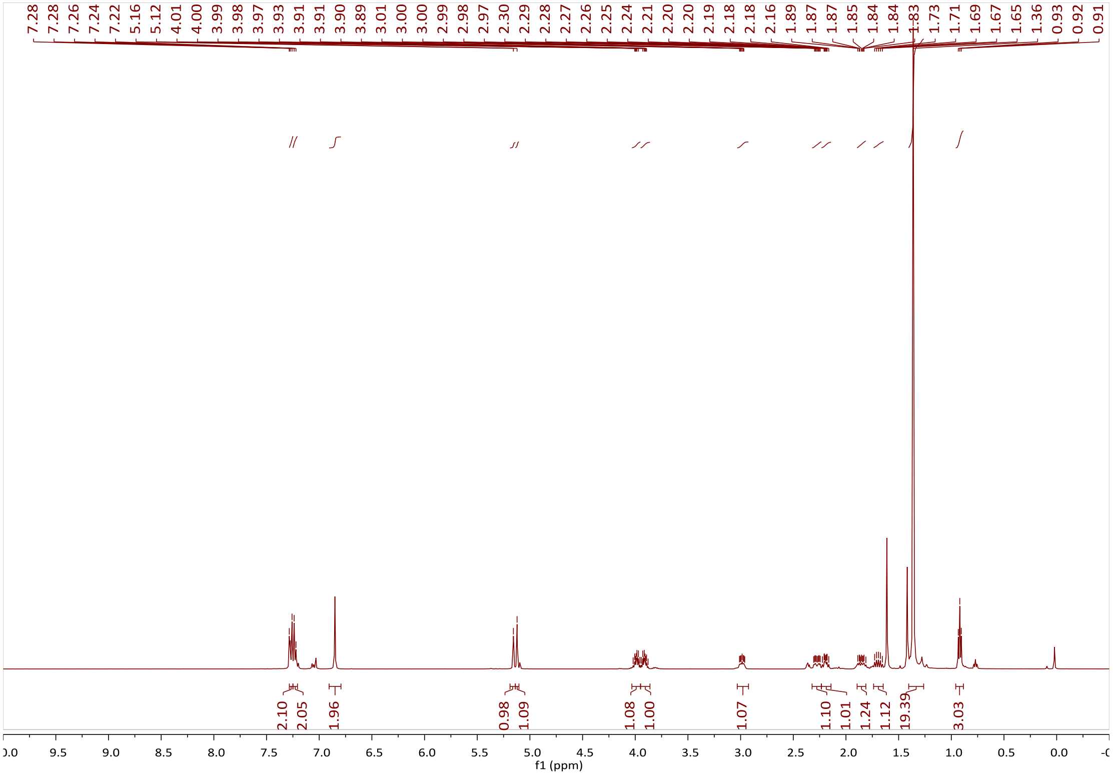
7.28 7.28 7.26 7.24 7.22 5.16 5.12 4.01 4.00 3.99 3.98 3.97 3.93 3.91 3.91 3.90 3.89 3.01 3.00 3.00 2.99 2.98 2.97 2.30 2.29 2.28 2.27 2.26 2.25 2.24 2.21 2.20 2.20 2.19 2.18 2.18 2.16 1.89 1.87 1.87 1.85 1.84 1.84 1.83 1.73 1.71 1.69 1.67 1.65 1.36 0.93 0.92 0.91
2.10 2.05 1.96 0.98 1.09 1.08 1.00 1.07 1.10 1.01 1.24 1.12 19.39 3.03
f1 (ppm)

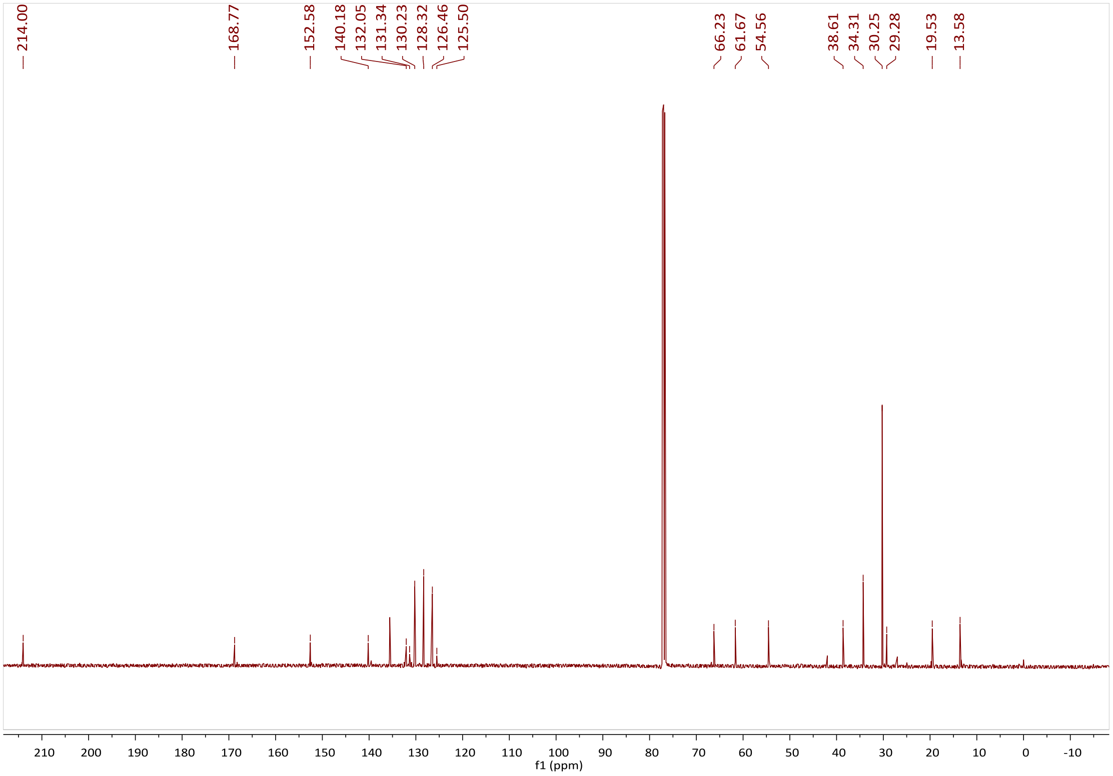
214.00 168.77 152.58 140.18 132.05 131.34 130.23 128.32 126.46 125.50 66.23 61.67 54.56 38.61 34.31 30.25 29.28 19.53 13.58
f1 (ppm)

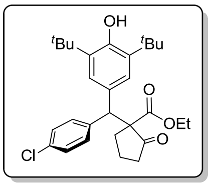

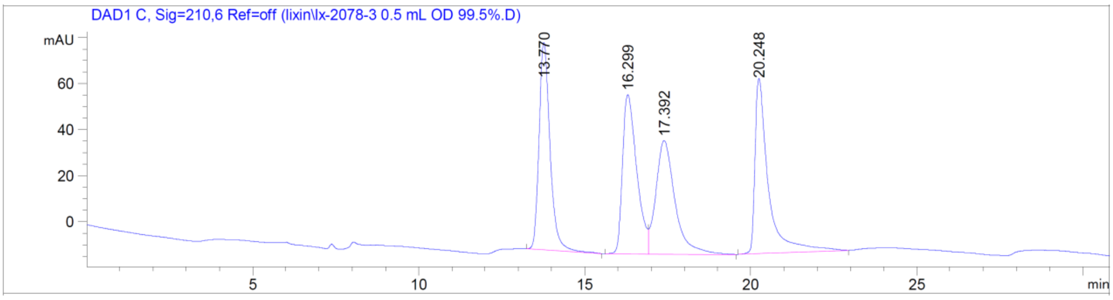

Peak #	RetTime [min]	Type	Width [min]	Area [mAU*s]	Height [mAU]	Area %
1	13.770	BB	0.3504	2068.84351	89.94075	25.1248
2	16.299	BV	0.4551	2054.40747	69.05700	24.9495
3	17.392	VB	0.6011	1977.74463	49.18896	24.0185
4	20.248	BB	0.4057	2133.27588	75.95226	25.9073

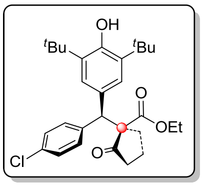

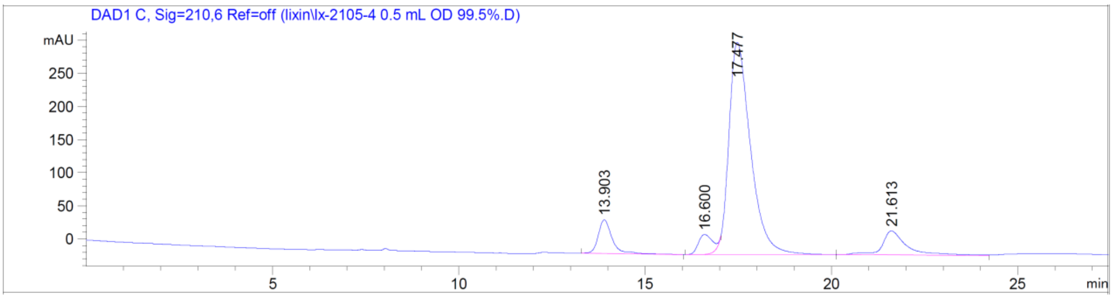

Peak #	RetTime [min]	Type	Width [min]	Area [mAU*s]	Height [mAU]	Area %
1	13.903	BB	0.3878	1310.78467	50.67213	7.8056
2	16.600	BV E	0.4036	765.38818	29.80852	4.5578
3	17.477	VB R	0.6180	1.30746e4	320.48593	77.8576
4	21.613	BB	0.6438	1642.17957	36.29714	9.7790

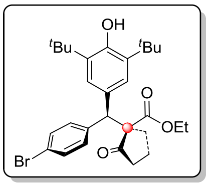
OH
tBu
tBu
O
OEt
Br
O

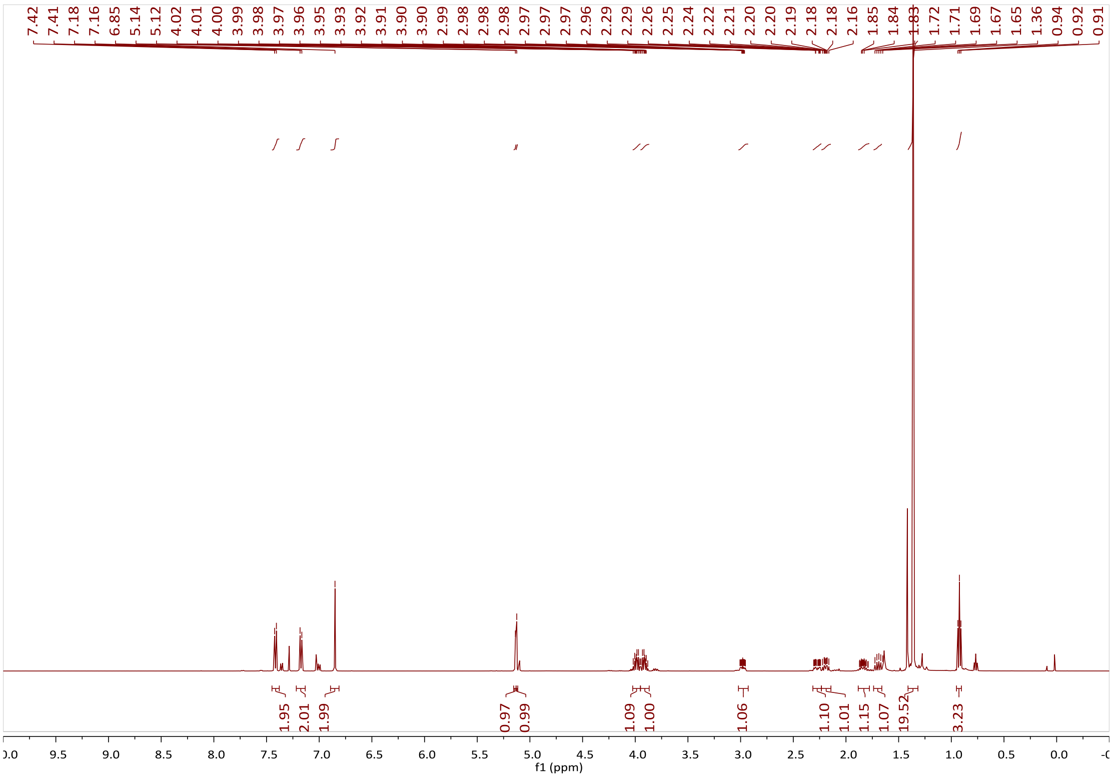
7.42 7.41 7.18 7.16 6.85 5.14 5.12 4.02 4.01 4.00 3.99 3.98 3.97 3.96 3.95 3.93 3.92 3.91 3.90 3.90 2.99 2.98 2.98 2.98 2.97 2.97 2.97 2.96 2.29 2.26 2.25 2.24 2.22 2.21 2.20 2.20 2.19 2.18 2.18 2.16 1.85 1.84 1.83 1.72 1.71 1.69 1.67 1.65 1.36 0.94 0.92 0.91
1.95 2.01 1.99 0.97 0.99 1.09 1.00 1.06 1.10 1.01 1.15 1.07 19.52 3.23
f1 (ppm)

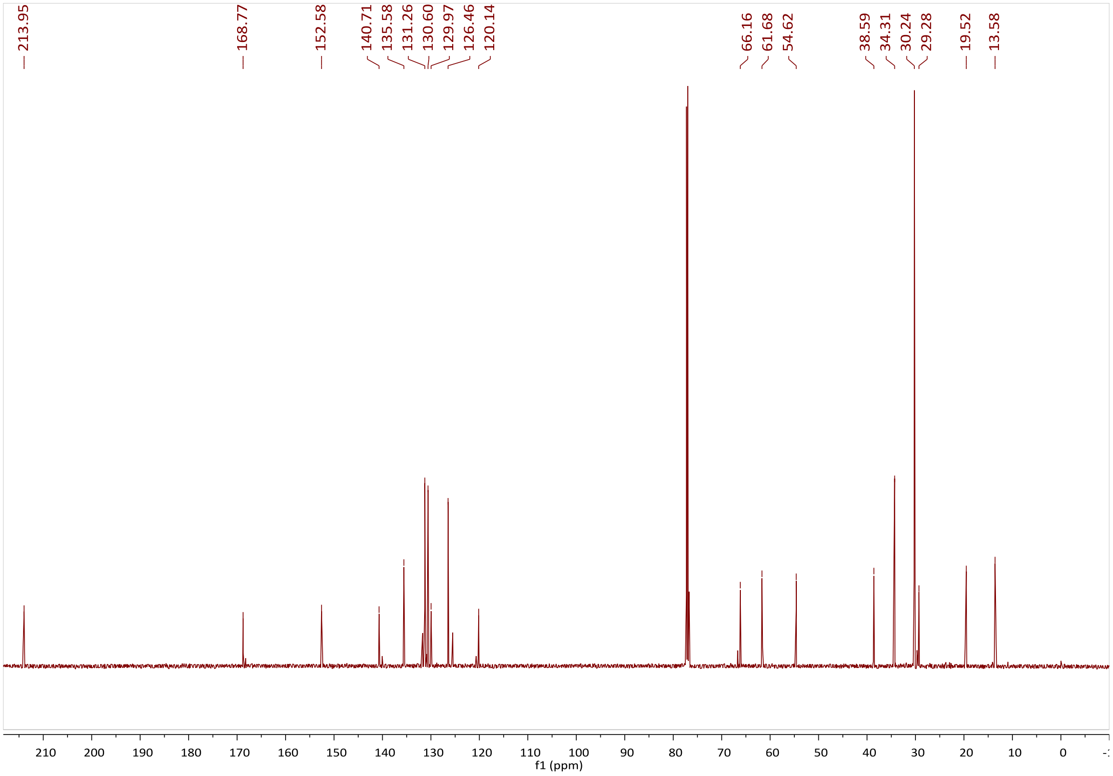
213.95 168.77 152.58 140.71 135.58 131.26 130.60 129.97 126.46 120.14 66.16 61.68 54.62 38.59 34.31 30.24 29.28 19.52 13.58
f1 (ppm)

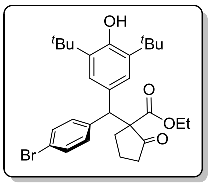

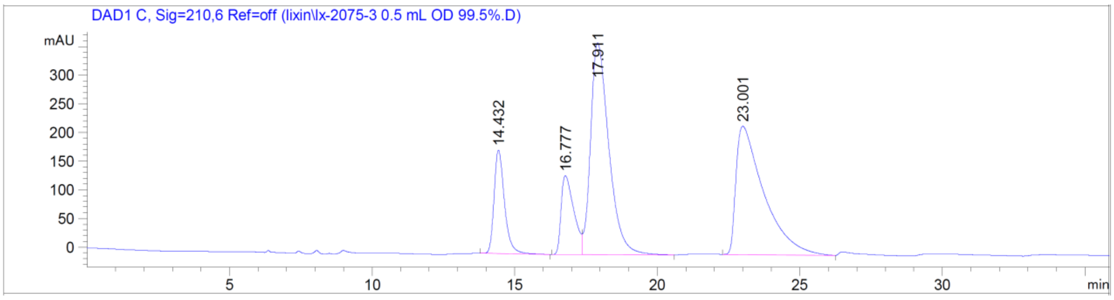

Peak #	RetTime [min]	Type	Width [min]	Area [mAU*s]	Height [mAU]	Area %
1	14.432	BB	0.3877	4611.97510	180.73631	11.3733
2	16.777	BV	0.4697	4282.09668	138.11496	10.5598
3	17.911	VB	0.6797	1.64766e4	369.88248	40.6319
4	23.001	BB	0.9649	1.51803e4	223.91840	37.4351

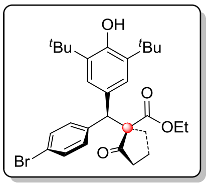

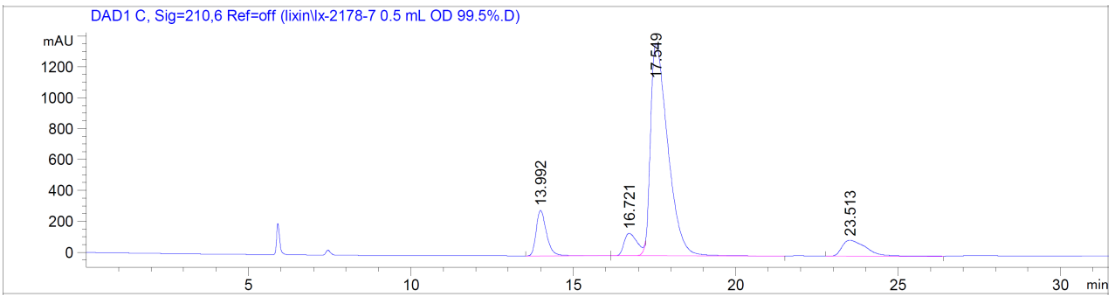

Peak #	RetTime [min]	Type	Width [min]	Area [mAU*s]	Height [mAU]	Area %
1	13.992	BB	0.3377	6566.94971	294.95740	9.7823
2	16.721	BV E	0.4320	4037.18921	145.37434	6.0139
3	17.549	VB R	0.5617	5.11689e4	1376.42908	76.2222
4	23.513	BB	0.7424	5358.21191	103.77435	7.9817

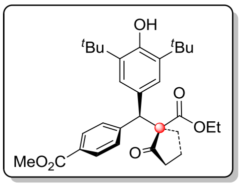
OH
tBu
tBu
O
OEt
MeO2C
O

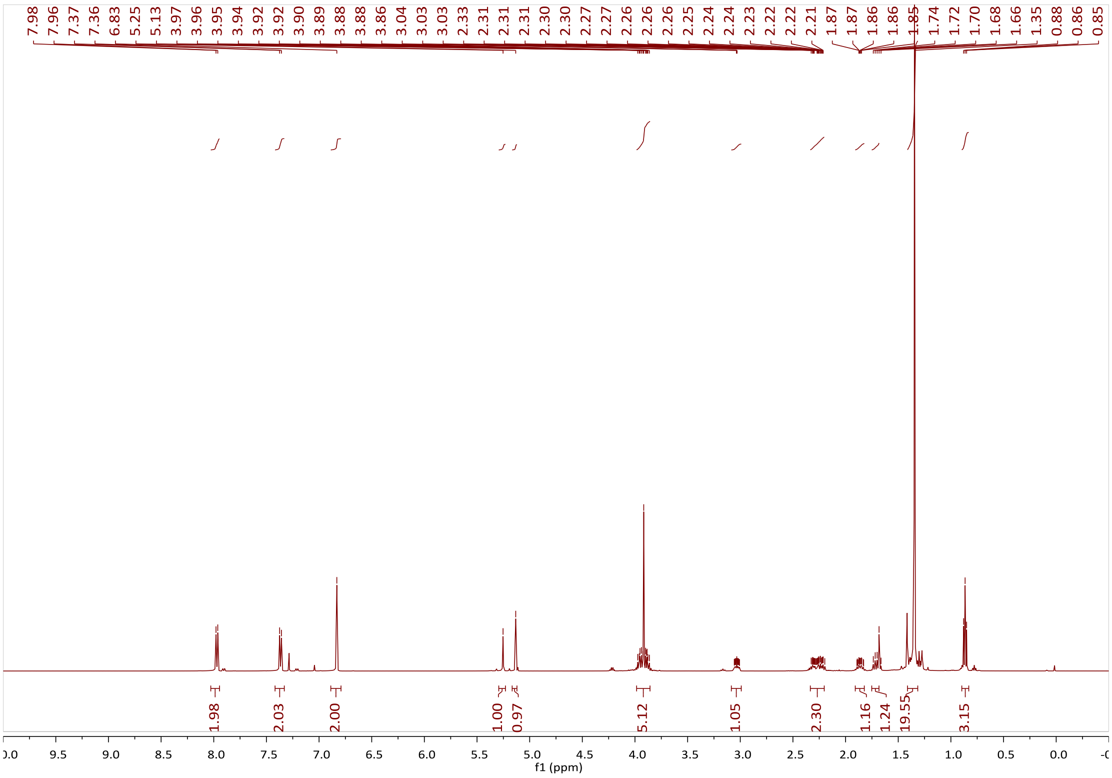
7.98
7.96
7.37
7.36
6.83
5.25
5.13
3.97
3.96
3.95
3.94
3.92
3.92
3.90
3.89
3.88
3.88
3.86
3.04
3.03
3.03
2.33
2.31
2.31
2.31
2.30
2.30
2.27
2.27
2.26
2.26
2.26
2.25
2.24
2.24
2.23
2.22
2.22
2.21
1.87
1.87
1.86
1.86
1.85
1.74
1.72
1.70
1.68
1.66
1.35
0.88
0.86
0.85
1.98
2.03
2.00
1.00
0.97
5.12
1.05
2.30
1.16
1.24
19.55
3.15
f1 (ppm)

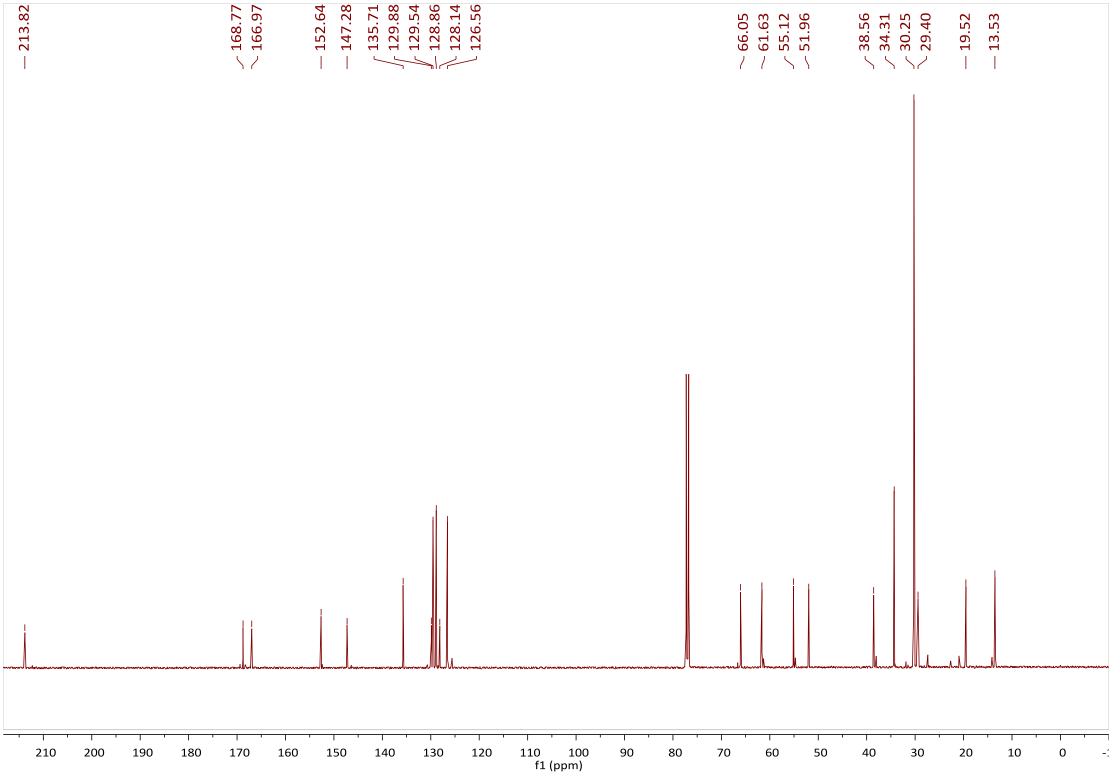
213.82
168.77
166.97
152.64
147.28
135.71
129.88
129.54
128.86
128.14
126.56
66.05
61.63
55.12
51.96
38.56
34.31
30.25
29.40
19.52
13.53
f1 (ppm)

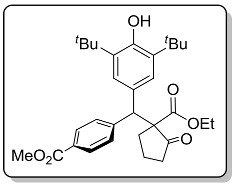

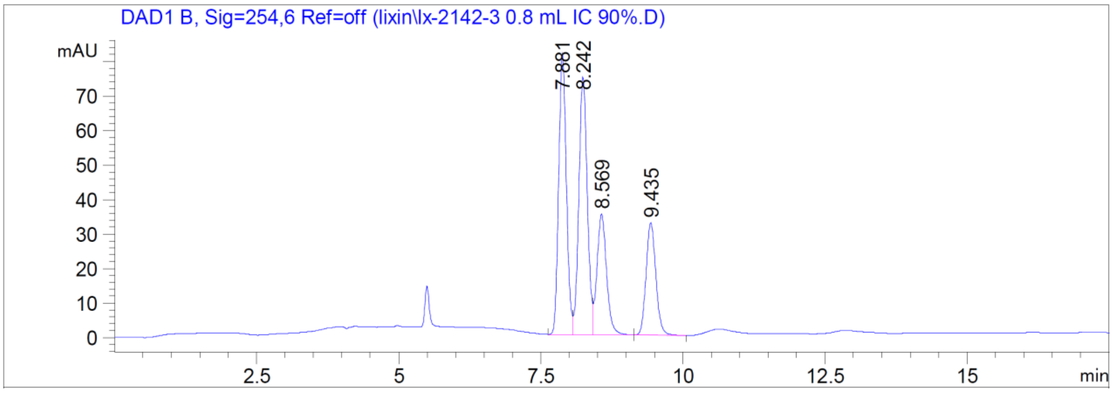

Peak #	RetTime [min]	Type	Width [min]	Area [mAU*s]	Height [mAU]	Area %
1	7.881	BV	0.1508	766.04126	80.67302	32.6879
2	8.242	VV	0.1625	774.88220	73.82061	33.0651
3	8.569	VB	0.1776	410.50931	34.81165	17.5169
4	9.435	BB	0.1892	392.06885	32.40253	16.7300

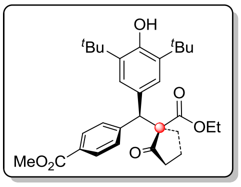

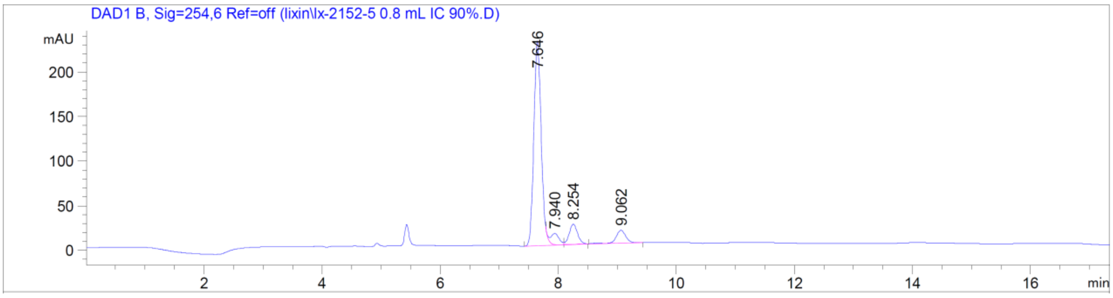

Peak #	RetTime [min]	Type	Width [min]	Area [mAU*s]	Height [mAU]	Area %
1	7.646	BV R	0.1359	1998.84216	228.93124	79.6863
2	7.940	VV E	0.1428	119.81644	12.61357	4.7766
3	8.254	VB E	0.1528	222.33044	22.59768	8.8635
4	9.062	BB	0.1761	167.39993	14.56363	6.6736

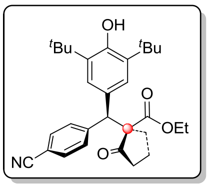
OH
tBu
tBu
O
OEt
NC
O

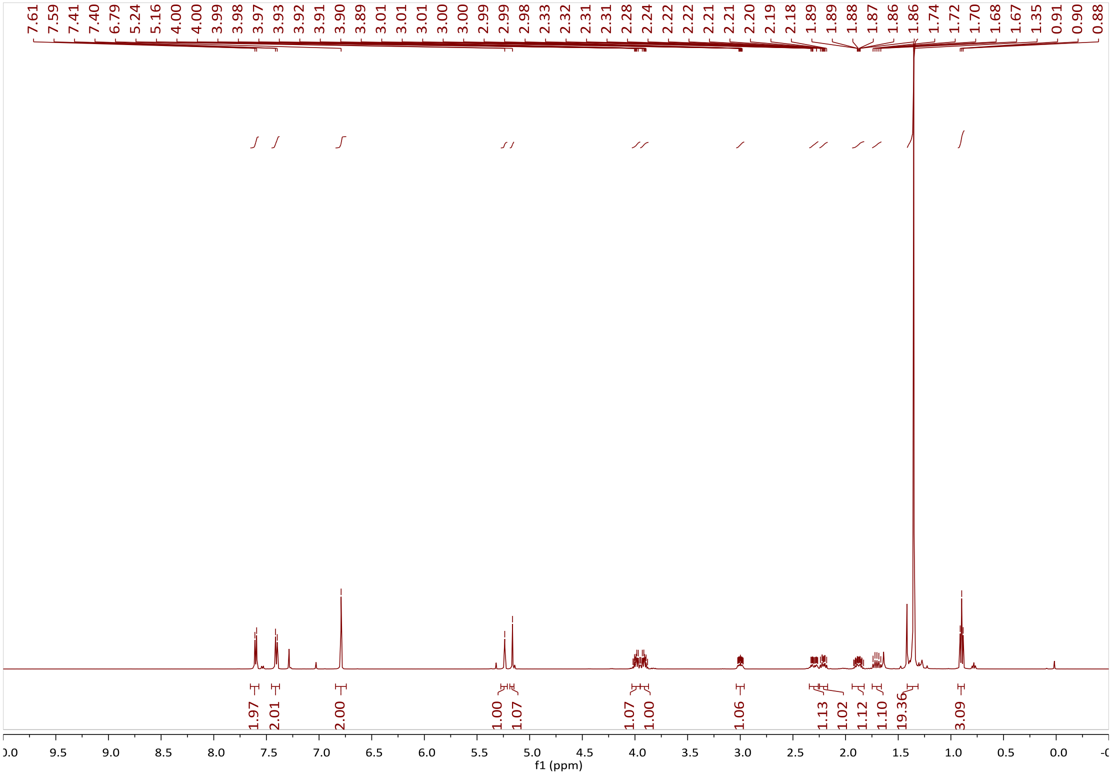
7.61 7.59 7.41 7.40 6.79 5.24 5.16 4.00 3.99 3.98 3.97 3.93 3.92 3.91 3.90 3.89 3.01 3.01 3.01 3.00 3.00 2.99 2.99 2.98 2.33 2.32 2.31 2.31 2.28 2.24 2.22 2.22 2.21 2.21 2.20 2.19 2.18 1.89 1.89 1.88 1.87 1.86 1.86 1.74 1.72 1.70 1.68 1.67 1.35 0.91 0.90 0.88
1.97 2.01 2.00 1.00 1.07 1.07 1.00 1.06 1.13 1.02 1.12 1.10 19.36 3.09
f1 (ppm)

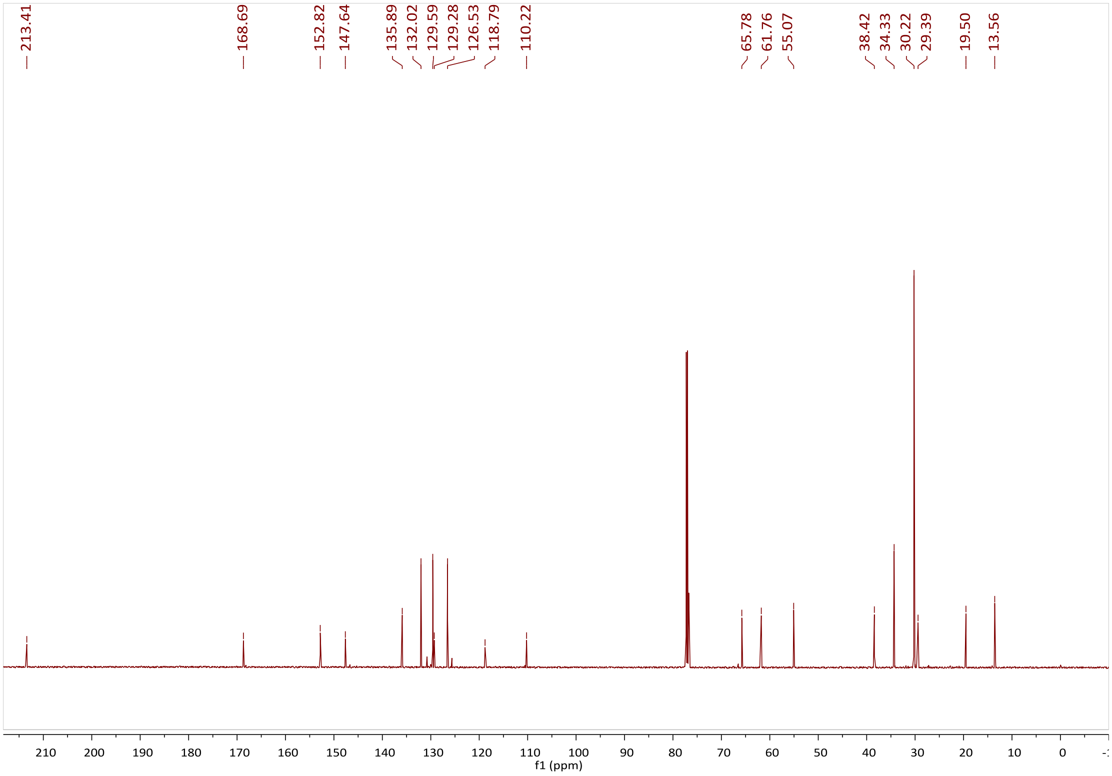
213.41 168.69 152.82 147.64 135.89 132.02 129.59 129.28 126.53 118.79 110.22 65.78 61.76 55.07 38.42 34.33 30.22 29.39 19.50 13.56
f1 (ppm)

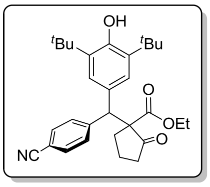

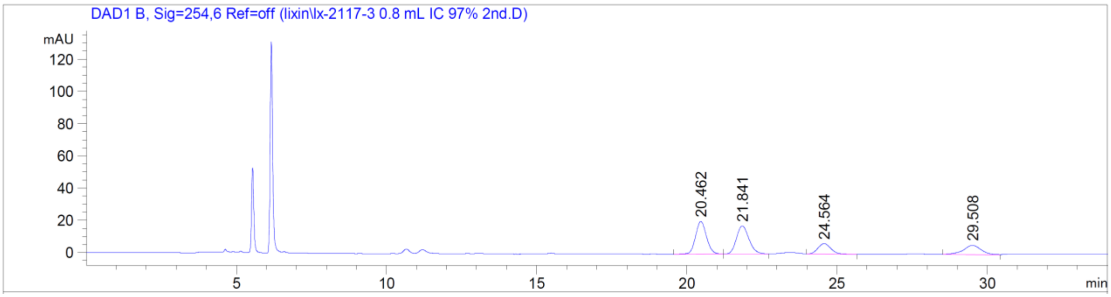

Peak #	RetTime [min]	Type	Width [min]	Area [mAU*s]	Height [mAU]	Area %
1	20.462	BB	0.3950	516.47723	20.28951	35.8235
2	21.841	BB	0.4490	506.97284	17.44513	35.1643
3	24.564	BB	0.4614	195.68358	6.53348	13.5729
4	29.508	BB	0.5839	222.59216	5.64611	15.4393

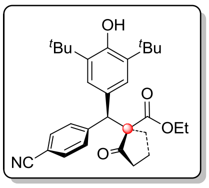

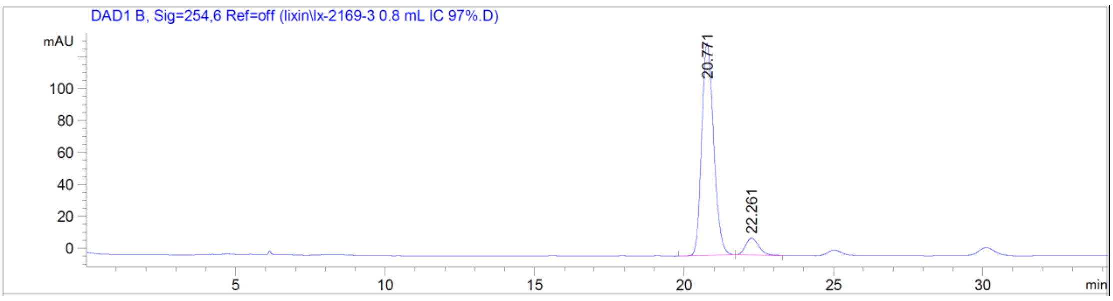

Peak #	RetTime [min]	Type	Width [min]	Area [mAU*s]	Height [mAU]	Area %
1	20.771	BB	0.4336	3707.22388	132.83463	92.0810
2	22.261	BB	0.4693	318.82248	10.52896	7.9190

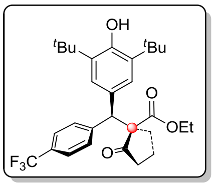

OH
tBu
tBu
O
OEt
F3C
O

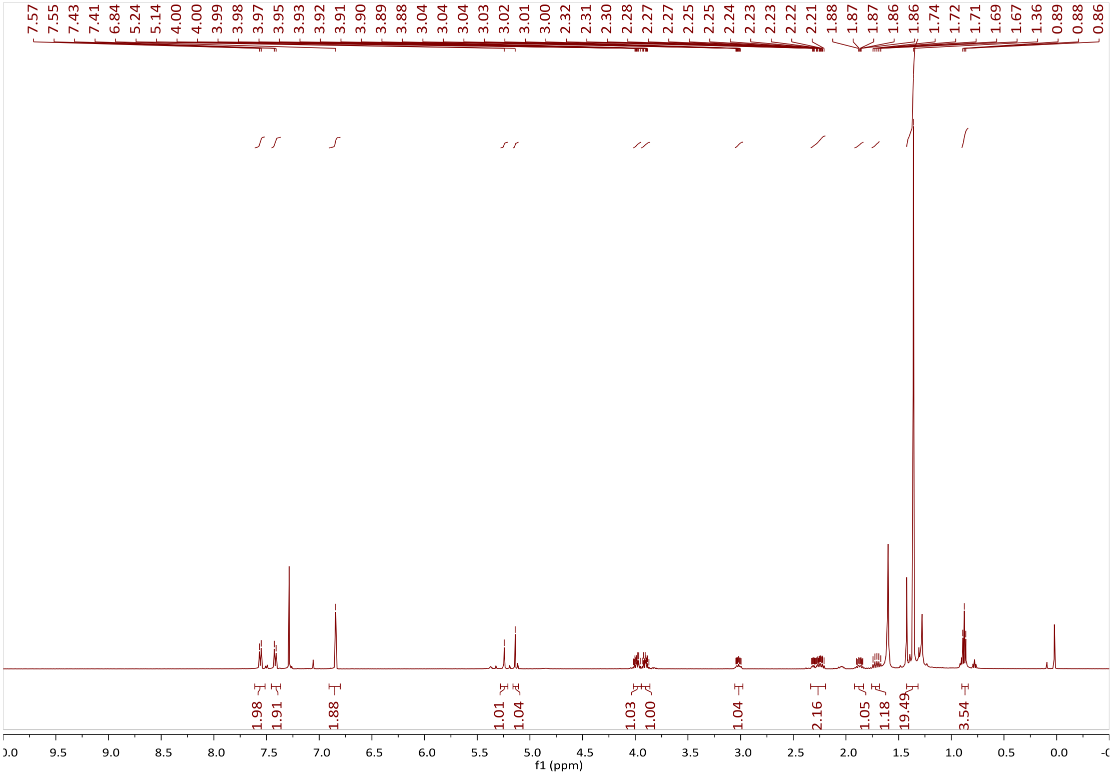

7.57
7.55
7.43
7.41
6.84
5.24
5.14
4.00
3.99
3.98
3.97
3.95
3.93
3.92
3.91
3.90
3.89
3.88
3.04
3.04
3.04
3.03
3.02
3.01
3.00
2.32
2.31
2.30
2.28
2.27
2.27
2.25
2.25
2.24
2.23
2.23
2.22
2.21
1.88
1.87
1.87
1.86
1.86
1.74
1.72
1.71
1.69
1.67
1.36
0.89
0.88
0.86
1.98
1.91
1.88
1.01
1.04
1.03
1.00
1.04
2.16
1.05
1.18
19.49
3.54
f1 (ppm)

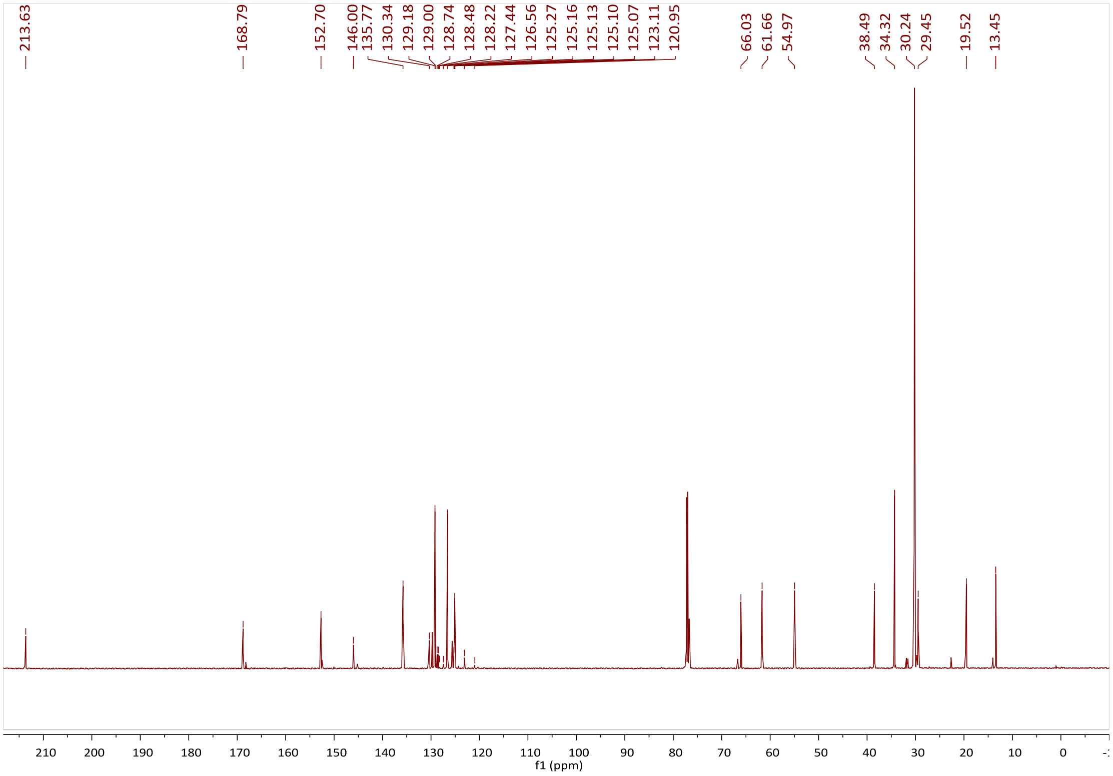

213.63
168.79
152.70
146.00
135.77
130.34
129.18
129.00
128.74
128.48
128.22
127.44
126.56
125.27
125.16
125.13
125.10
125.07
123.11
120.95
66.03
61.66
54.97
38.49
34.32
30.24
29.45
19.52
13.45
f1 (ppm)

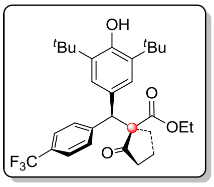
OH
tBu
tBu
O
OEt
O
F3C

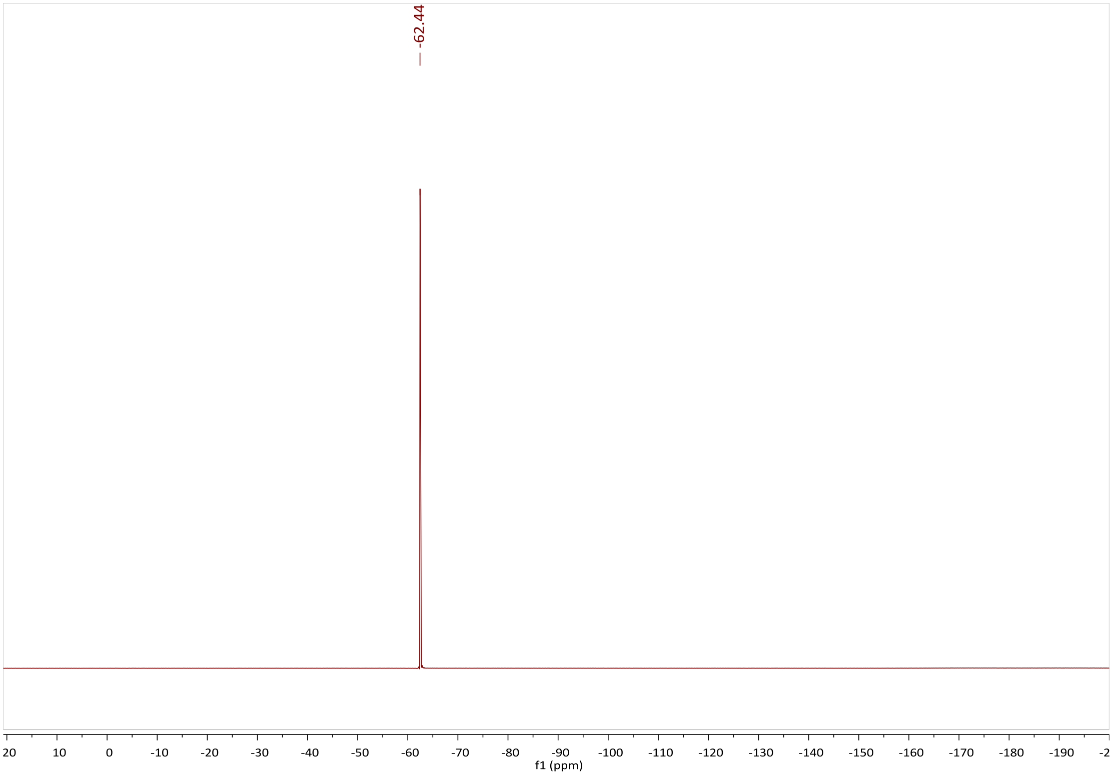
-62.44
20
10
0
-10
-20
-30
-40
-50
-60
-70
-80
-90
-100
-110
-120
-130
-140
-150
-160
-170
-180
-190
-2
f1 (ppm)

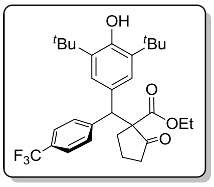

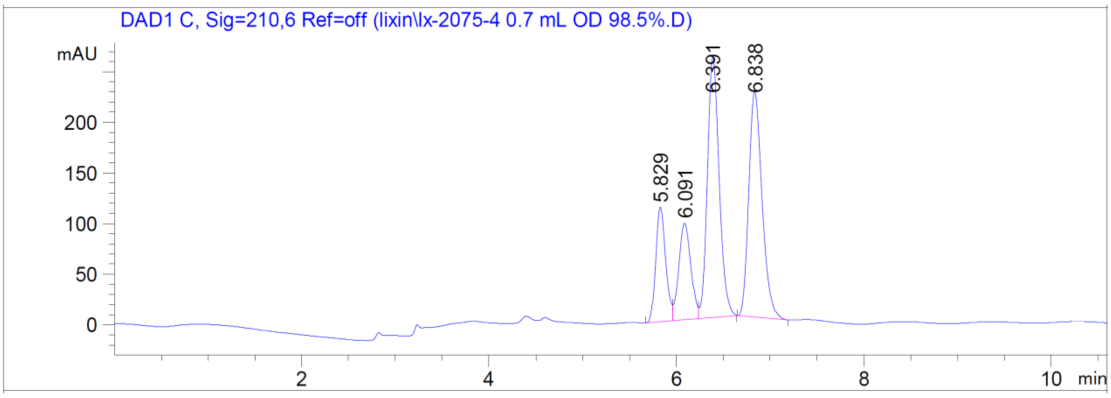

Peak #	RetTime [min]	Type	Width [min]	Area [mAU*s]	Height [mAU]	Area %
1	5.829	BV	0.1187	73.68540	9.91421	12.2623
2	6.091	VV	0.1445	74.13377	8.27458	12.3370
3	6.391	VB	0.1441	228.19540	25.57241	37.9751
4	6.838	BB	0.1580	224.89366	22.22635	37.4256

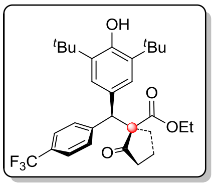

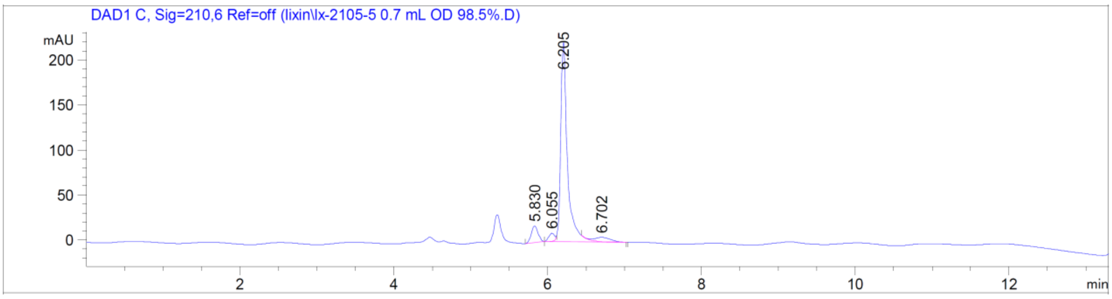

Peak #	RetTime [min]	Type	Width [min]	Area [mAU*s]	Height [mAU]	Area %
1	5.830	BB	0.0962	111.98220	18.09220	7.1169
2	6.055	BV E	0.0781	44.27112	8.89284	2.8136
3	6.205	VV R	0.0871	1348.55347	221.40514	85.7055
4	6.702	VB E	0.2218	68.66701	4.77505	4.3640

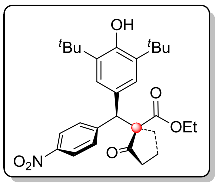
OH
tBu
tBu
O
OEt
O
O2N

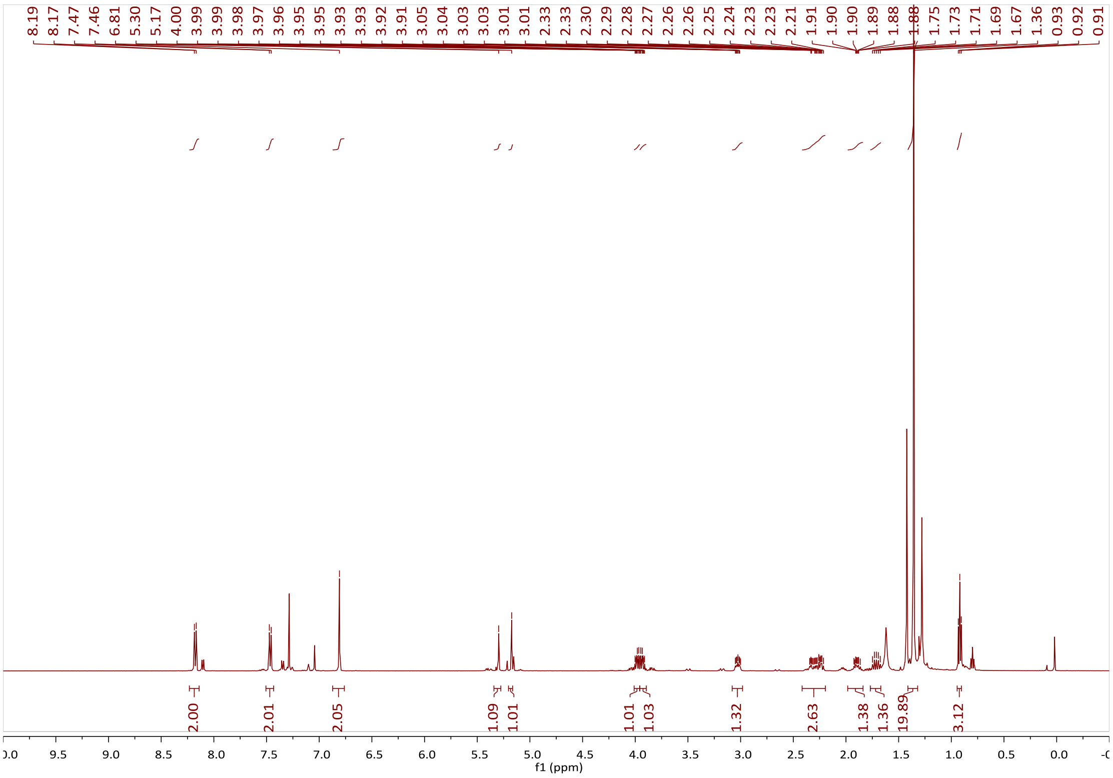
8.19
8.17
7.47
7.46
6.81
5.30
5.17
4.00
3.99
3.99
3.98
3.97
3.96
3.95
3.95
3.93
3.93
3.92
3.91
3.05
3.04
3.03
3.03
3.01
3.01
2.33
2.33
2.30
2.29
2.28
2.27
2.26
2.26
2.25
2.24
2.23
2.23
2.21
1.91
1.90
1.90
1.89
1.88
1.88
1.75
1.73
1.71
1.69
1.67
1.36
0.93
0.92
0.91
2.00
2.01
2.05
1.09
1.01
1.01
1.03
1.32
2.63
1.38
1.36
19.89
3.12
f1 (ppm)

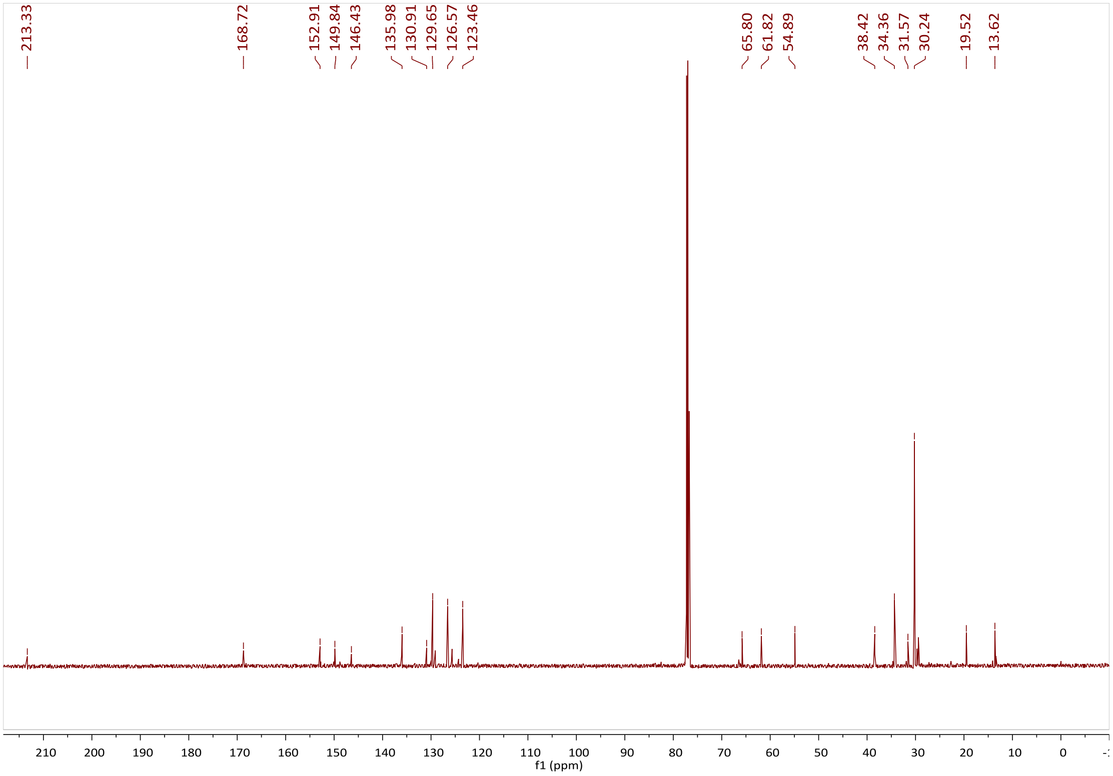
213.33
168.72
152.91
149.84
146.43
135.98
130.91
129.65
126.57
123.46
65.80
61.82
54.89
38.42
34.36
31.57
30.24
19.52
13.62
f1 (ppm)

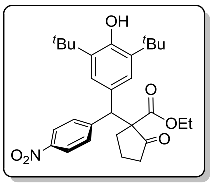

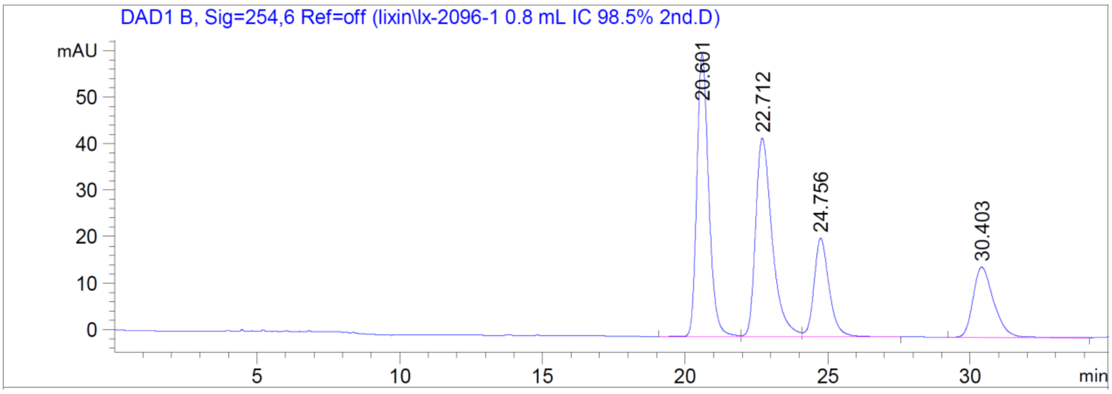

Peak #	RetTime [min]	Type	Width [min]	Area [mAU*s]	Height [mAU]	Area %
1	20.601	VV R	0.4426	1771.11353	60.85490	34.9785
2	22.712	VV	0.6176	1737.47388	42.80026	34.3141
3	24.756	VB	0.5627	791.25043	21.23731	15.6268
4	30.403	BV R	0.7710	763.59644	15.11681	15.0806

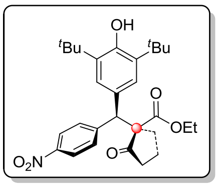

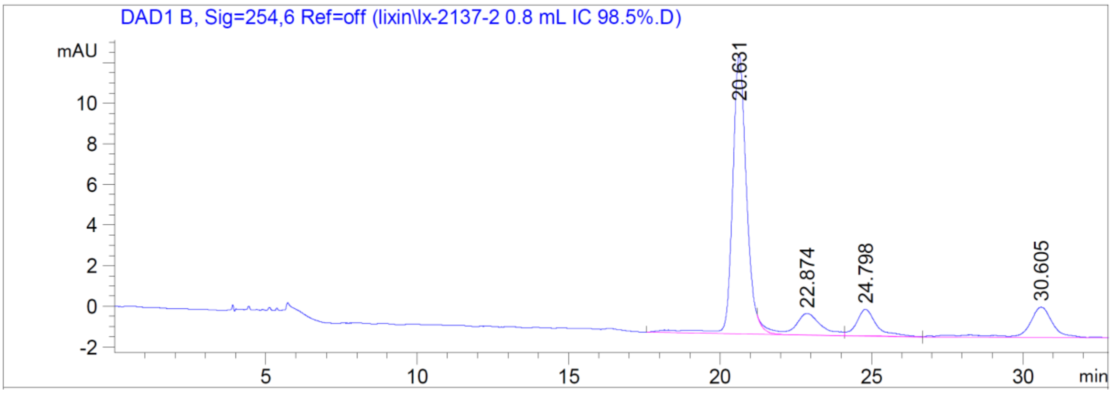

Peak #	RetTime [min]	Type	Width [min]	Area [mAU*s]	Height [mAU]	Area %
1	20.631	VV R	0.4870	447.75009	13.78463	67.6644
2	22.874	VV E	0.8921	65.53629	1.05592	9.9039
3	24.798	VB E	0.6636	60.23436	1.31197	9.1027
4	30.605	VV R	0.7704	88.20069	1.50067	13.3290

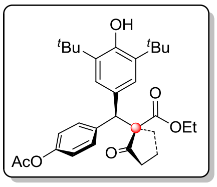

OH
^{t}Bu
^{t}Bu
O
OEt
AcO
O

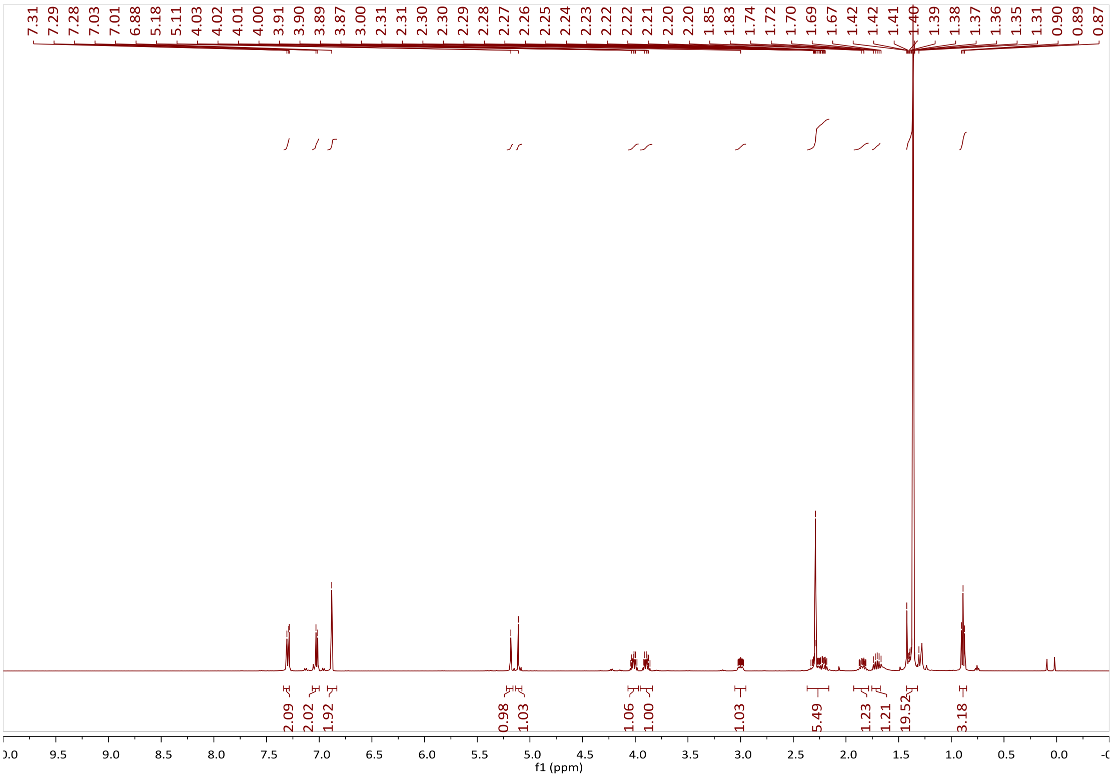

7.31 7.29 7.28 7.03 7.01 6.88 5.18 5.11 4.03 4.02 4.01 4.00 3.91 3.90 3.89 3.87 3.00 2.31 2.31 2.30 2.30 2.29 2.28 2.27 2.26 2.25 2.24 2.23 2.22 2.22 2.21 2.20 2.20 1.85 1.83 1.74 1.72 1.70 1.69 1.67 1.42 1.42 1.41 1.40 1.39 1.38 1.37 1.36 1.35 1.31 0.90 0.89 0.87
2.09 2.02 1.92 0.98 1.03 1.06 1.00 1.03 5.49 1.23 1.21 19.52 3.18
f1 (ppm)

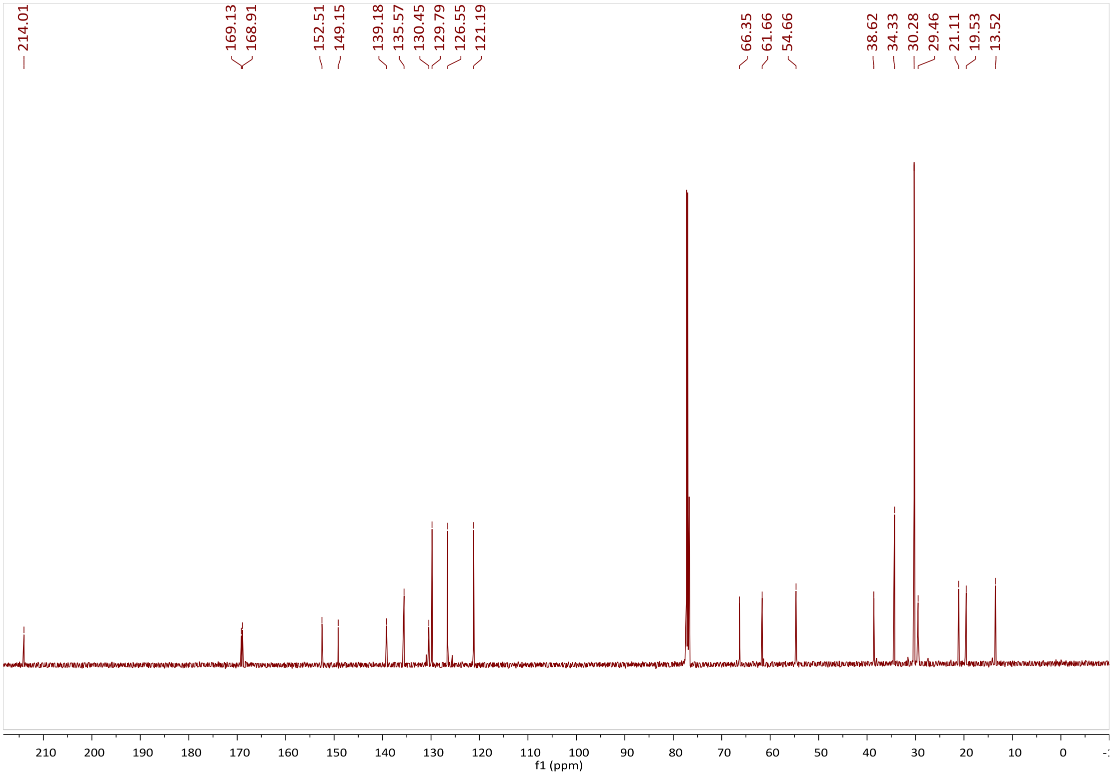

214.01 169.13 168.91 152.51 149.15 139.18 135.57 130.45 129.79 126.55 121.19 66.35 61.66 54.66 38.62 34.33 30.28 29.46 21.11 19.53 13.52
f1 (ppm)

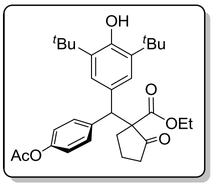

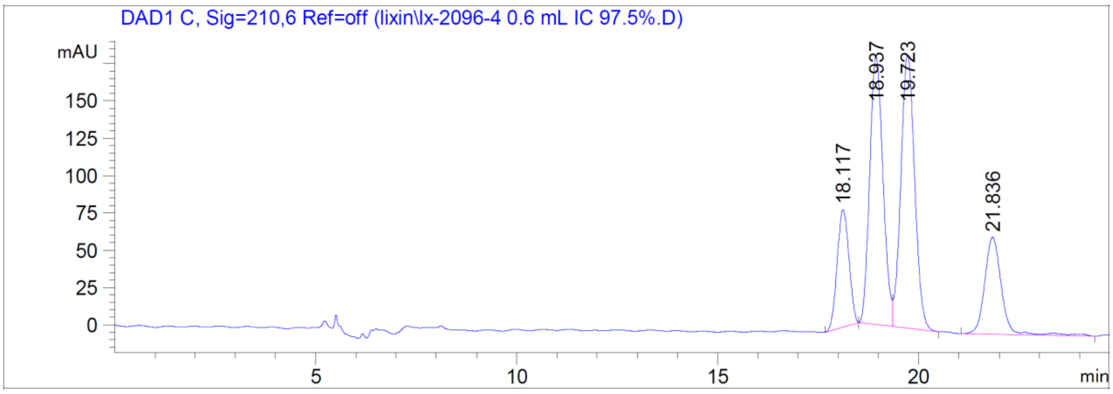

Peak #	RetTime [min]	Type	Width [min]	Area [mAU*s]	Height [mAU]	Area %
1	18.117	BB	0.3262	1603.70190	78.55562	13.3672
2	18.937	BV	0.3643	4123.07422	179.29413	34.3667
3	19.723	VB	0.3741	4362.26758	182.99480	36.3605
4	21.836	BV R	0.4470	1908.24207	64.97588	15.9056

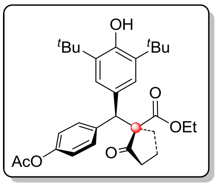

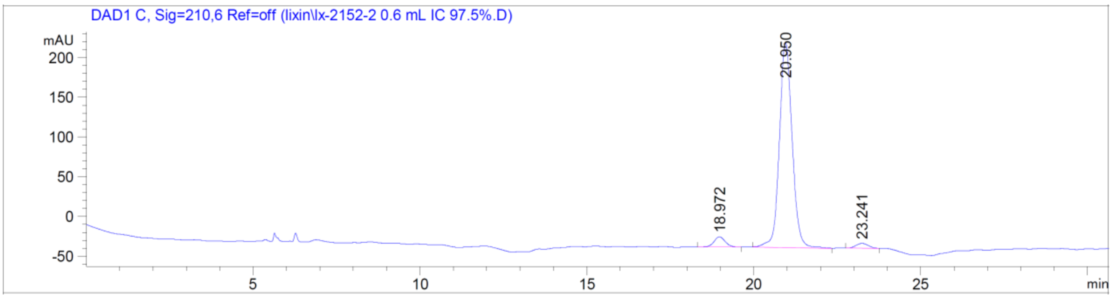

Peak #	RetTime [min]	Type	Width [min]	Area [mAU*s]	Height [mAU]	Area %
1	18.972	BB	0.3650	313.11130	13.09260	4.3047
2	20.950	BB	0.4065	6806.86914	257.38055	93.5825
3	23.241	BB	0.3826	153.67906	6.12845	2.1128

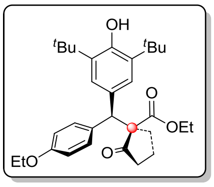
OH
tBu
tBu
O
OEt
EtO
O

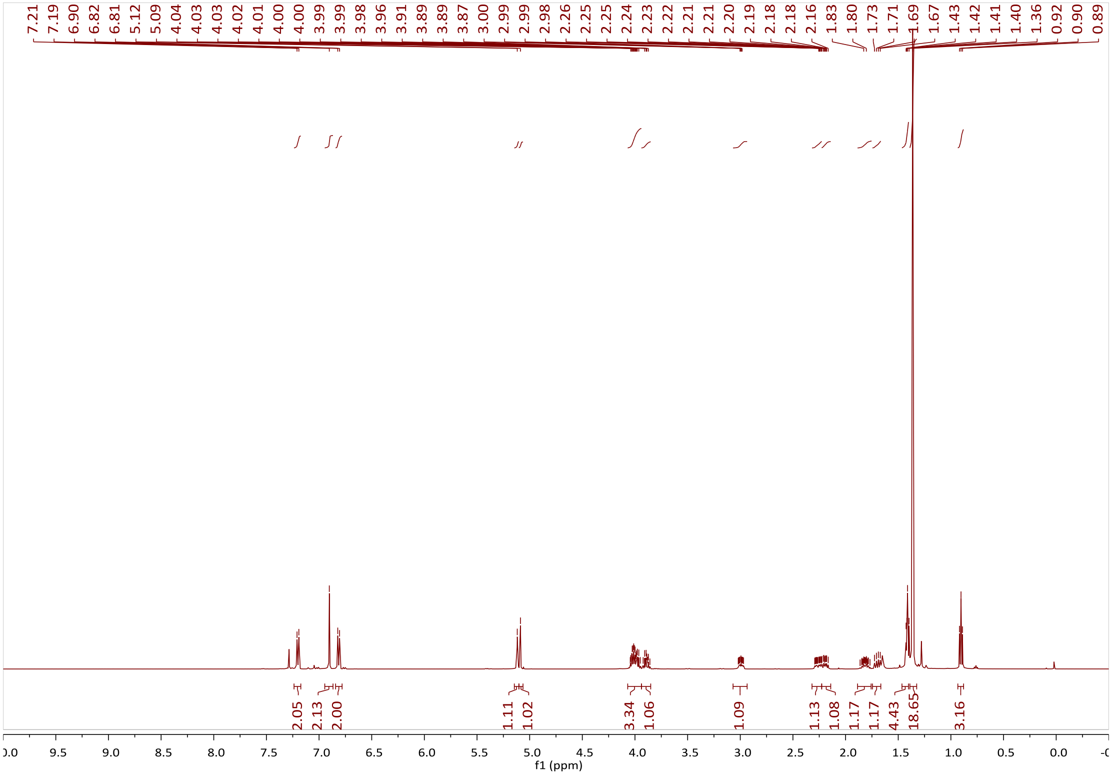
7.21 7.19 6.90 6.82 6.81 5.12 5.09 4.04 4.03 4.03 4.02 4.01 4.00 4.00 3.99 3.99 3.98 3.96 3.91 3.89 3.89 3.87 3.00 2.99 2.99 2.98 2.26 2.25 2.25 2.24 2.23 2.22 2.21 2.21 2.20 2.19 2.18 2.18 2.16 1.83 1.80 1.73 1.71 1.69 1.67 1.43 1.42 1.41 1.40 1.36 0.92 0.90 0.89
2.05 2.13 2.00 1.11 1.02 3.34 1.06 1.09 1.13 1.08 1.17 1.17 4.43 18.65 3.16
f1 (ppm)

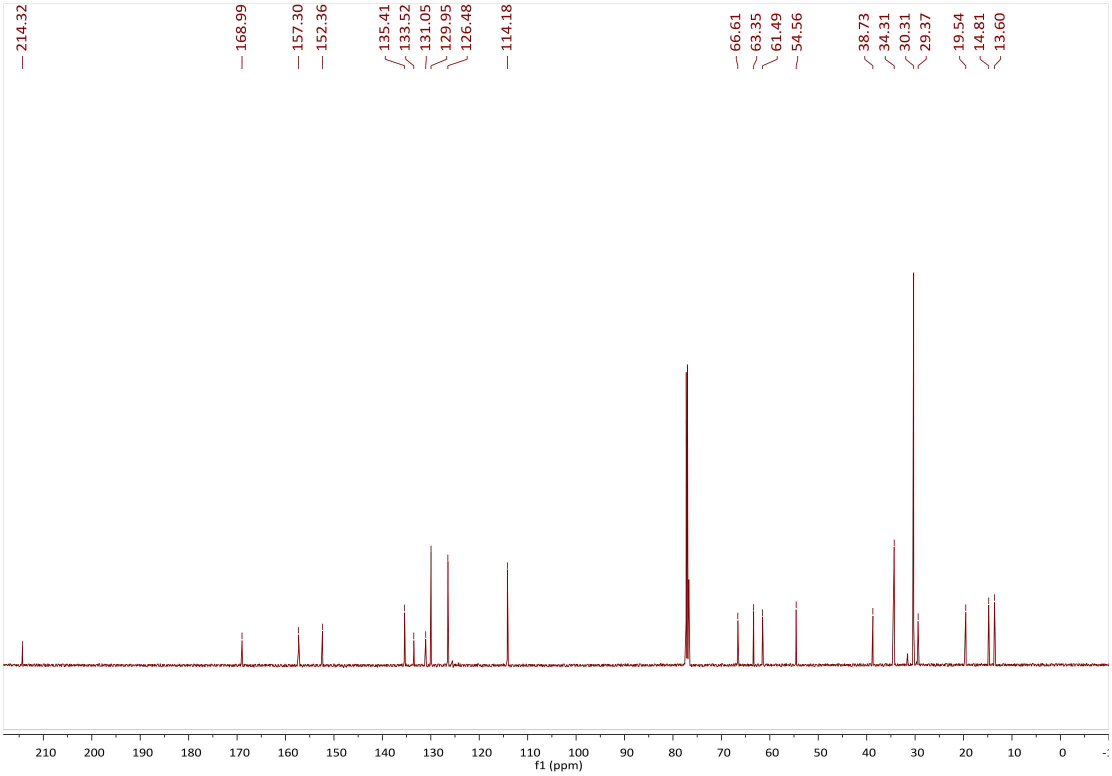
214.32 168.99 157.30 152.36 135.41 133.52 131.05 129.95 126.48 114.18 66.61 63.35 61.49 54.56 38.73 34.31 30.31 29.37 19.54 14.81 13.60
f1 (ppm)

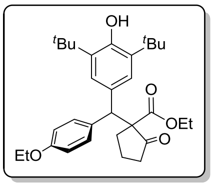

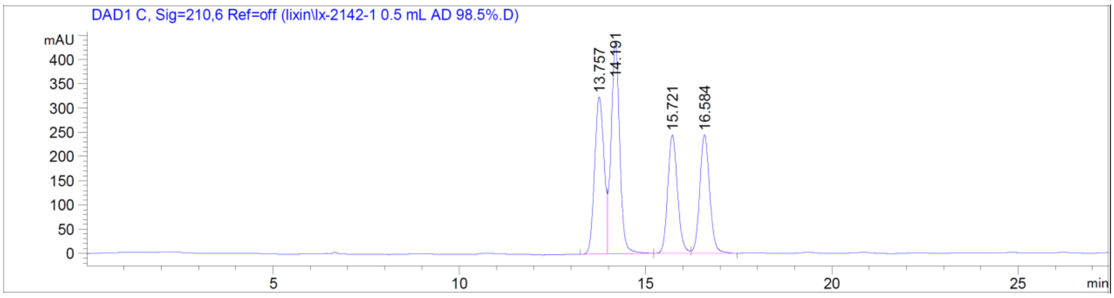

Peak #	RetTime [min]	Type	Width [min]	Area [mAU*s]	Height [mAU]	Area %
1	13.757	BV	0.2727	5750.36035	324.58197	26.4570
2	14.191	VB	0.2442	7047.83252	436.82874	32.4266
3	15.721	BV	0.2794	4419.53955	243.89597	20.3340
4	16.584	VB	0.2858	4516.96924	244.24625	20.7823

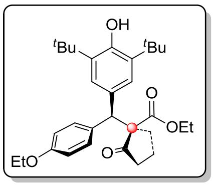

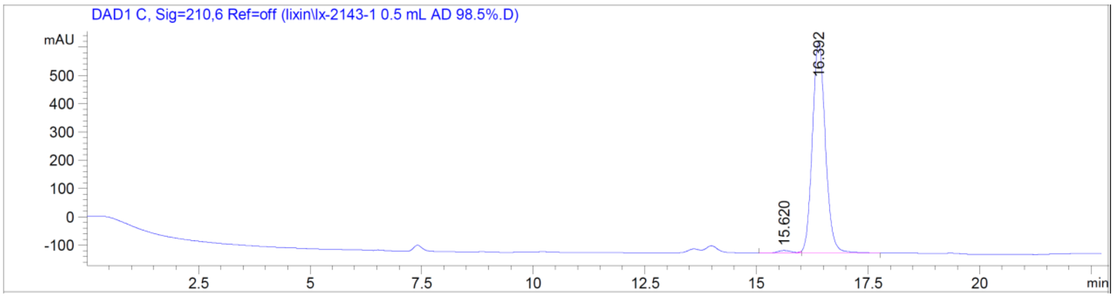

Peak #	RetTime [min]	Type	Width [min]	Area [mAU*s]	Height [mAU]	Area %
1	15.620	BV E	0.3187	199.53831	9.51499	1.3028
2	16.392	VB R	0.3144	1.51167e4	746.05170	98.6972

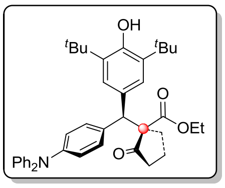
OH
tBu
tBu
O
OEt
O
Ph2N

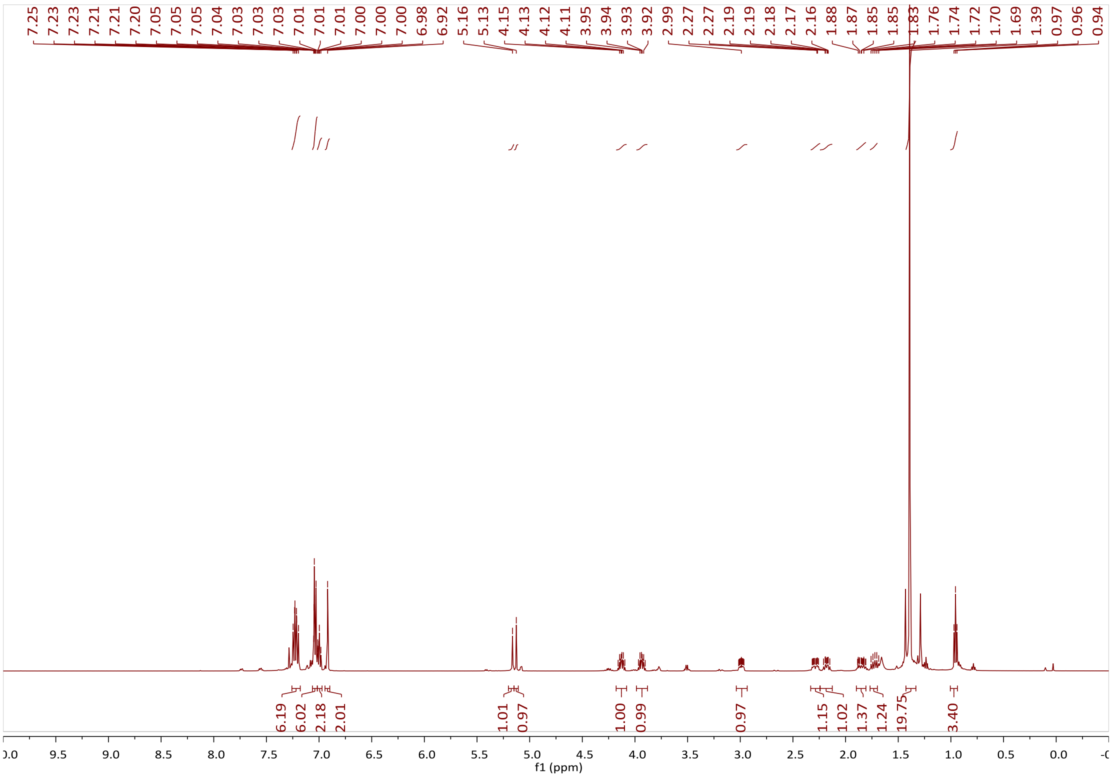
7.25 7.23 7.23 7.21 7.21 7.20 7.05 7.05 7.04 7.03 7.03 7.01 7.01 7.00 7.00 6.98 6.92 5.16 5.13 4.15 4.13 4.12 4.11 3.95 3.94 3.93 3.92 2.99 2.27 2.27 2.19 2.19 2.18 2.17 2.16 1.88 1.87 1.85 1.85 1.83 1.76 1.74 1.72 1.70 1.69 1.39 0.97 0.96 0.94
6.19 6.02 2.18 2.01 1.01 0.97 1.00 0.99 0.97 1.15 1.02 1.37 1.24 19.75 3.40
f1 (ppm)

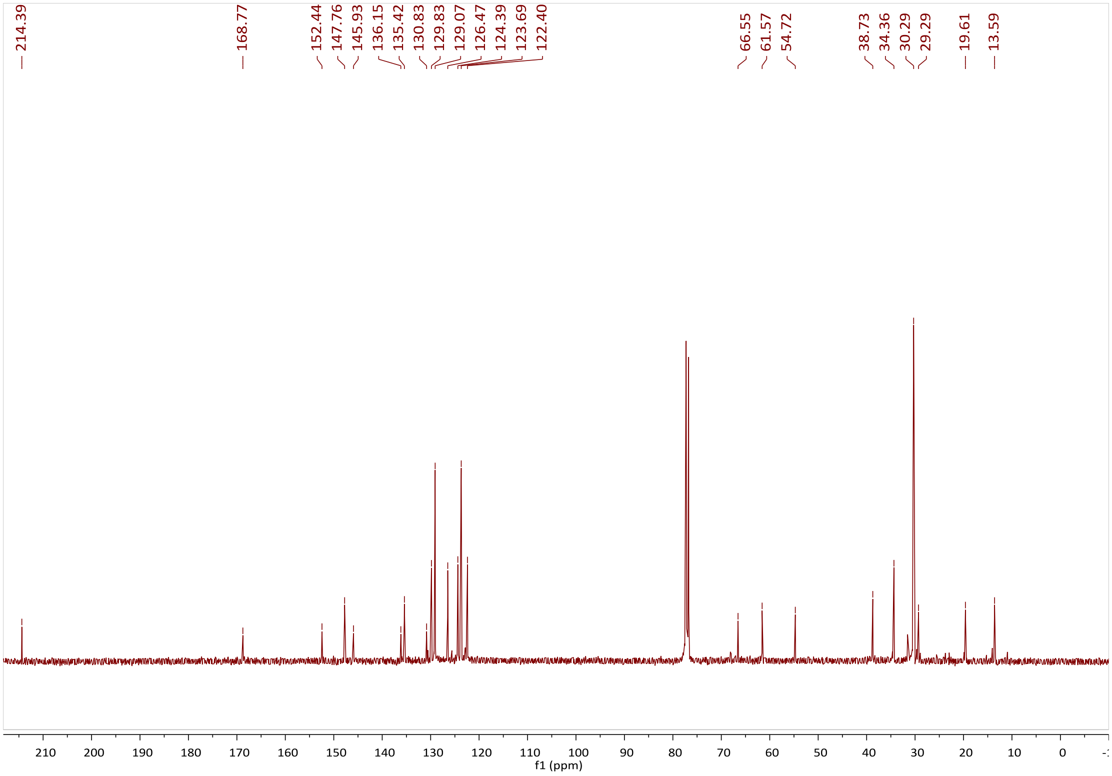
214.39 168.77 152.44 147.76 145.93 136.15 135.42 130.83 129.83 129.07 126.47 124.39 123.69 122.40 66.55 61.57 54.72 38.73 34.36 30.29 29.29 19.61 13.59
f1 (ppm)

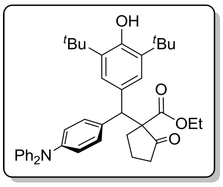

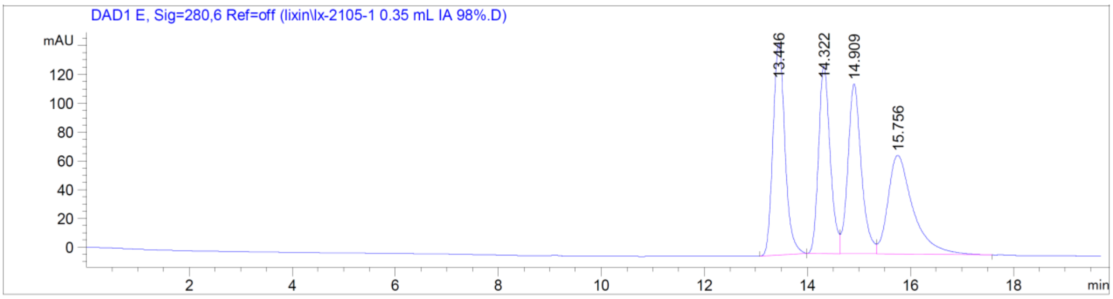

Peak #	RetTime [min]	Type	Width [min]	Area [mAU*s]	Height [mAU]	Area %
1	13.446	BB	0.2498	2372.83838	147.38861	26.6774
2	14.322	BV	0.2414	2046.57544	130.17674	23.0093
3	14.909	VV	0.2744	2150.11865	118.07677	24.1734
4	15.756	VB	0.4990	2325.02661	68.66835	26.1399

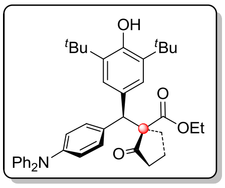

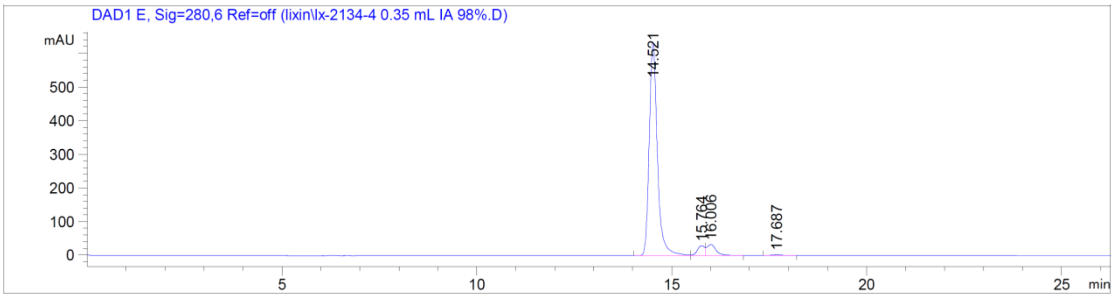

Peak #	RetTime [min]	Type	Width [min]	Area [mAU*s]	Height [mAU]	Area %
1	14.521	BV	0.2189	9176.25586	633.76440	90.5038
2	15.764	VV	0.1965	383.78079	29.36015	3.7852
3	16.006	VB	0.2373	533.85358	32.90141	5.2653
4	17.687	BB	0.2783	45.19367	2.50748	0.4457

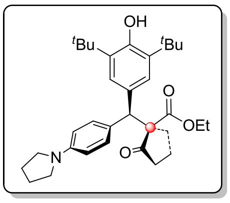

OH
^{t}Bu
^{t}Bu
O
OEt
O
N

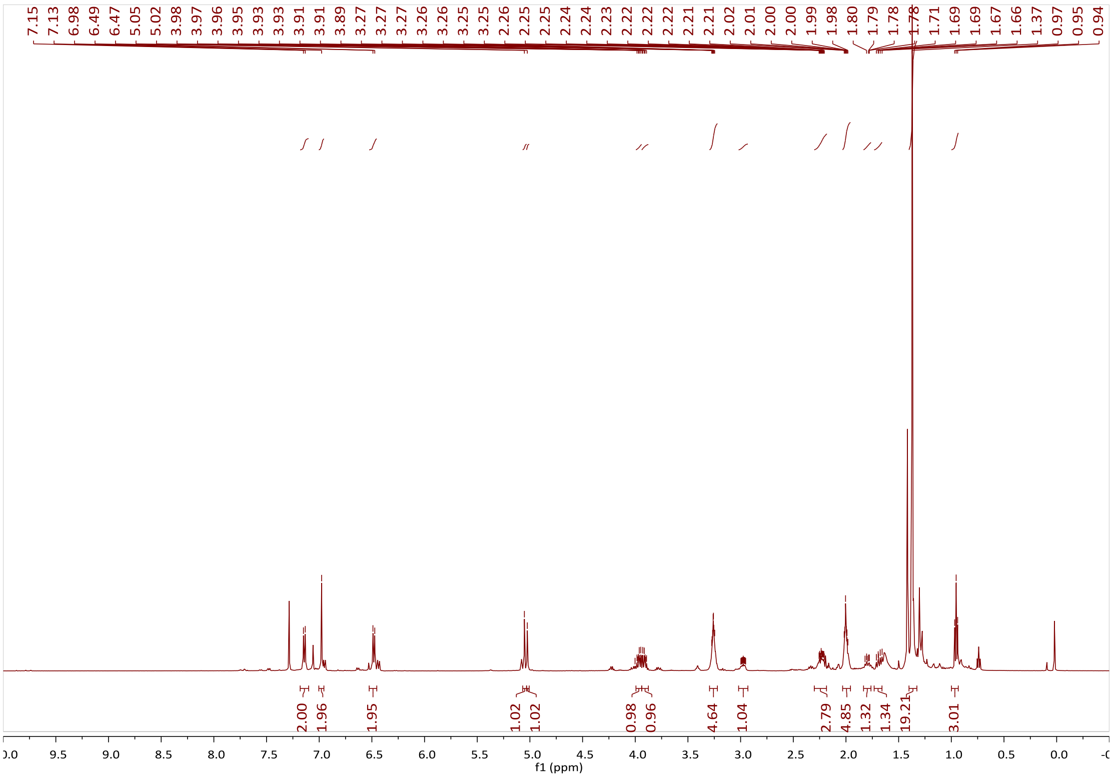

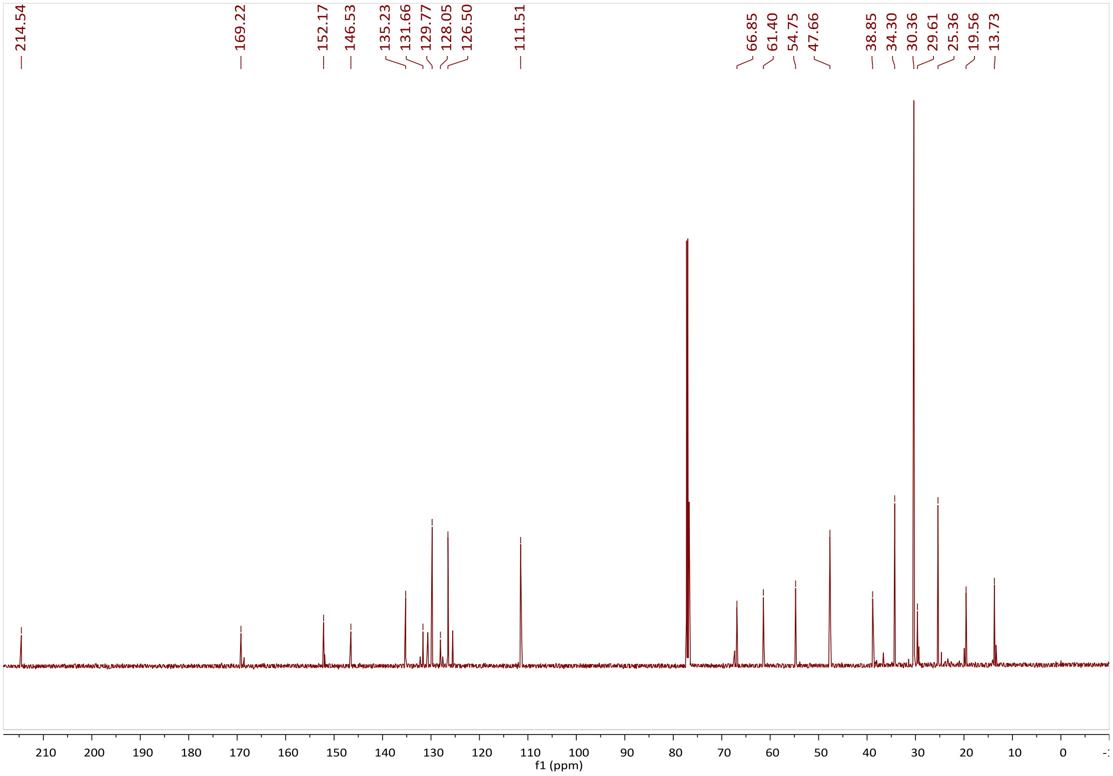

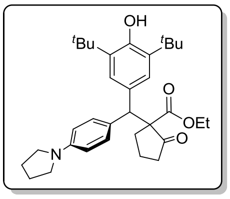

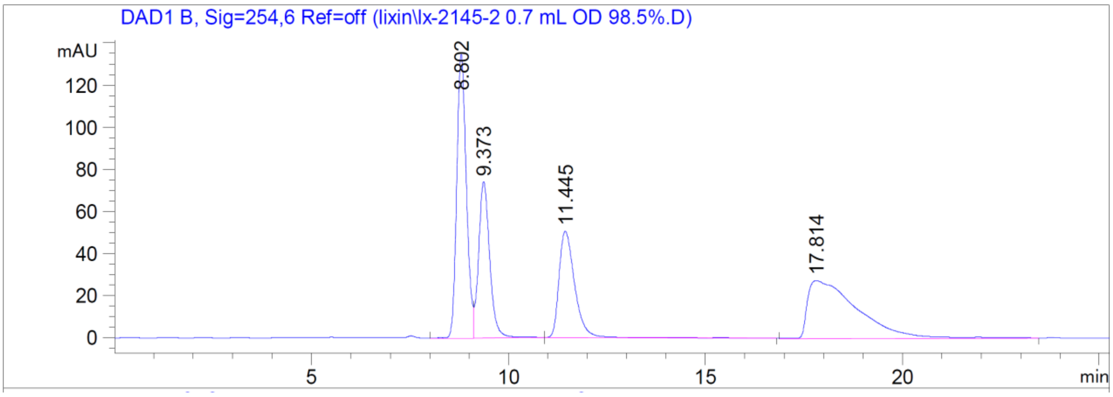

Peak #	RetTime [min]	Type	Width [min]	Area [mAU*s]	Height [mAU]	Area %
1	8.802	BV	0.2625	2286.06860	134.33971	30.3488
2	9.373	VV R	0.2997	1447.70581	73.99107	19.2191
3	11.445	BV R	0.4239	1405.67859	50.38414	18.6612
4	17.814	VV R	1.1299	2393.18555	27.47340	31.7709

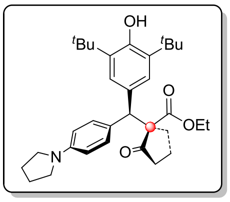

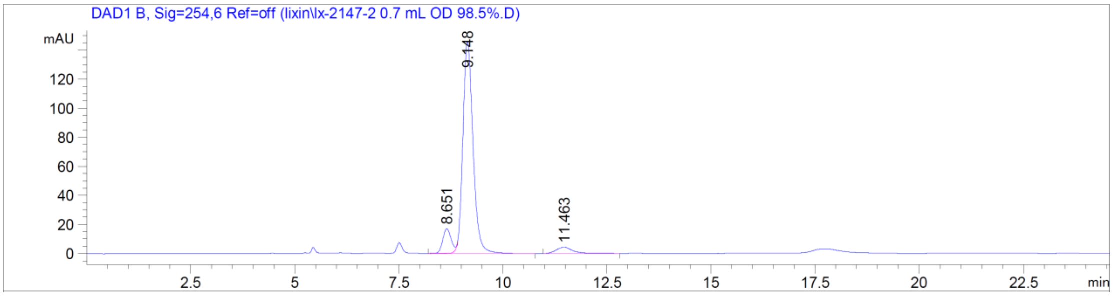

Peak #	RetTime [min]	Type	Width [min]	Area [mAU*s]	Height [mAU]	Area %
1	8.651	BV E	0.2164	237.36667	17.05150	8.3178
2	9.148	VB R	0.2609	2495.06372	146.36200	87.4320
3	11.463	BB	0.3705	121.28978	4.47070	4.2502

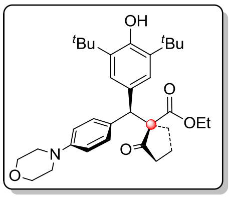
OH
tBu
tBu
O
OEt
O
N
O

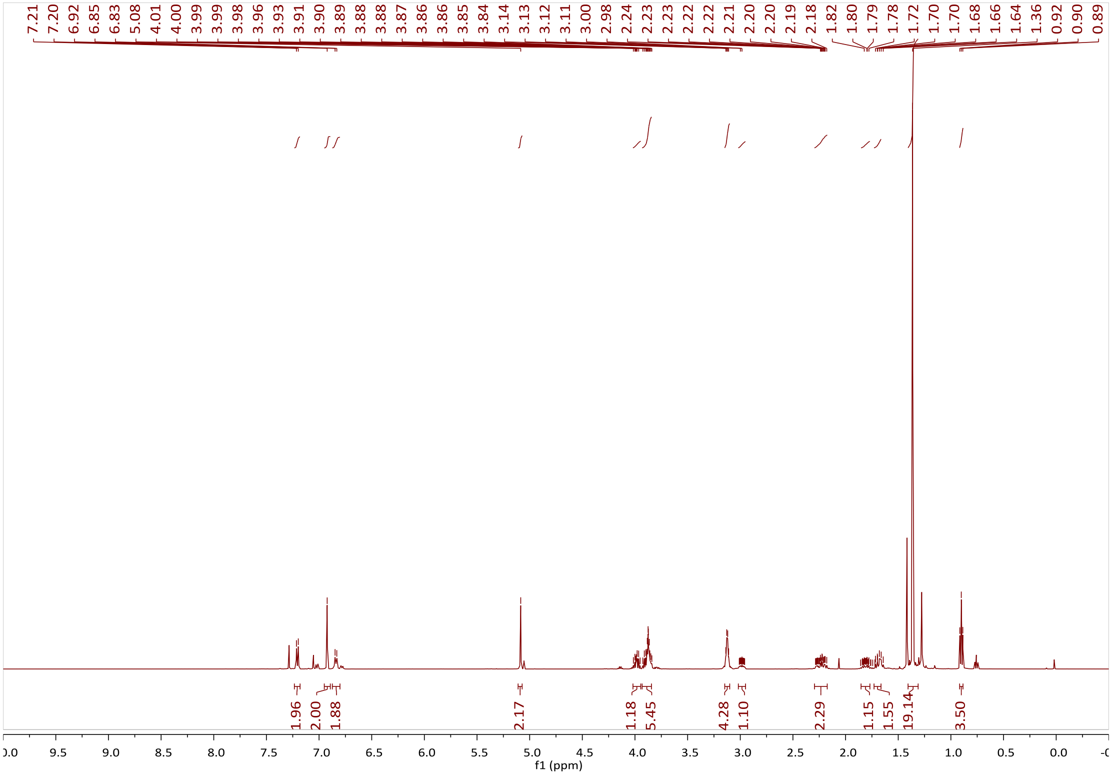
7.21
7.20
6.92
6.85
6.83
5.08
4.01
4.00
3.99
3.99
3.98
3.96
3.93
3.91
3.90
3.89
3.88
3.88
3.87
3.86
3.86
3.85
3.84
3.14
3.13
3.12
3.11
3.00
2.98
2.24
2.23
2.23
2.22
2.22
2.21
2.20
2.20
2.19
2.18
1.82
1.80
1.79
1.78
1.72
1.70
1.70
1.68
1.66
1.64
1.36
0.92
0.90
0.89
1.96
2.00
1.88
2.17
1.18
5.45
4.28
1.10
2.29
1.15
1.55
19.14
3.50
f1 (ppm)

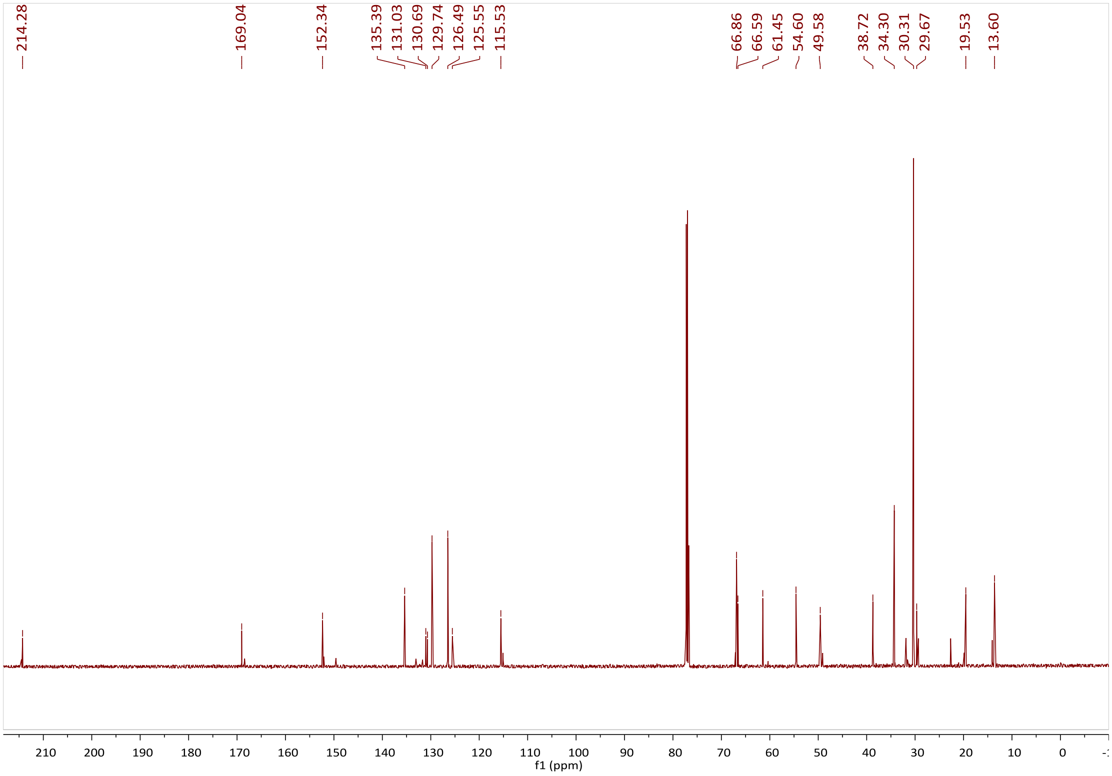
214.28
169.04
152.34
135.39
131.03
130.69
129.74
126.49
125.55
115.53
66.86
66.59
61.45
54.60
49.58
38.72
34.30
30.31
29.67
19.53
13.60
f1 (ppm)

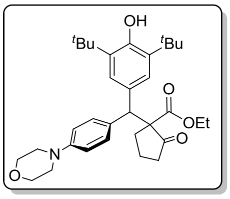

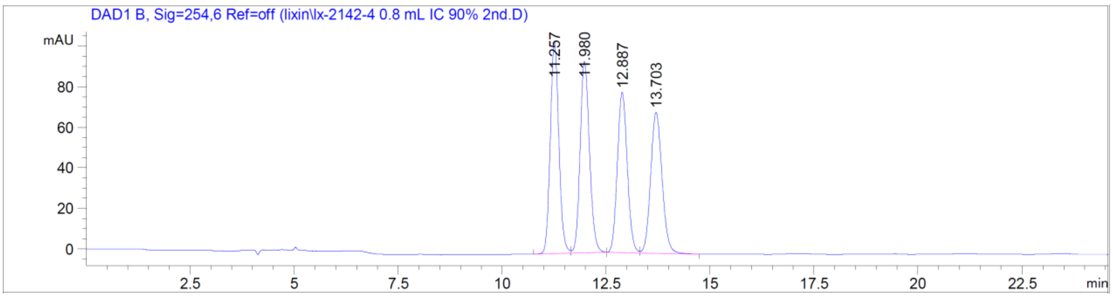

Peak #	RetTime [min]	Type	Width [min]	Area [mAU*s]	Height [mAU]	Area %
1	11.257	BV	0.2215	1493.88623	104.07958	26.6167
2	11.980	VB	0.2429	1477.72266	94.27510	26.3287
3	12.887	BV	0.2576	1309.74756	78.93672	23.3359
4	13.703	VB	0.2979	1331.23608	69.37405	23.7187

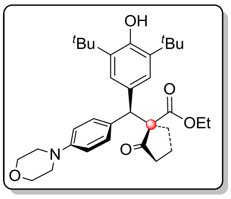

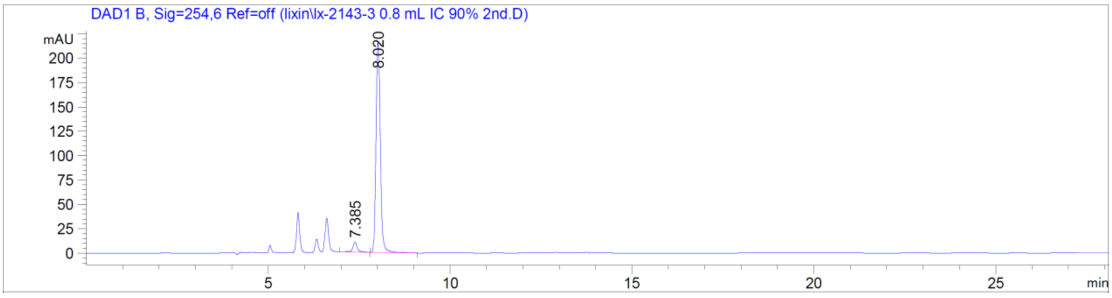

Peak #	RetTime [min]	Type	Width [min]	Area [mAU*s]	Height [mAU]	Area %
1	7.385	BB	0.1302	88.48915	10.10199	4.7265
2	8.020	BB	0.1261	1783.70056	216.50288	95.2735

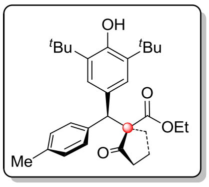
OH
tBu
tBu
O
OEt
O
Me

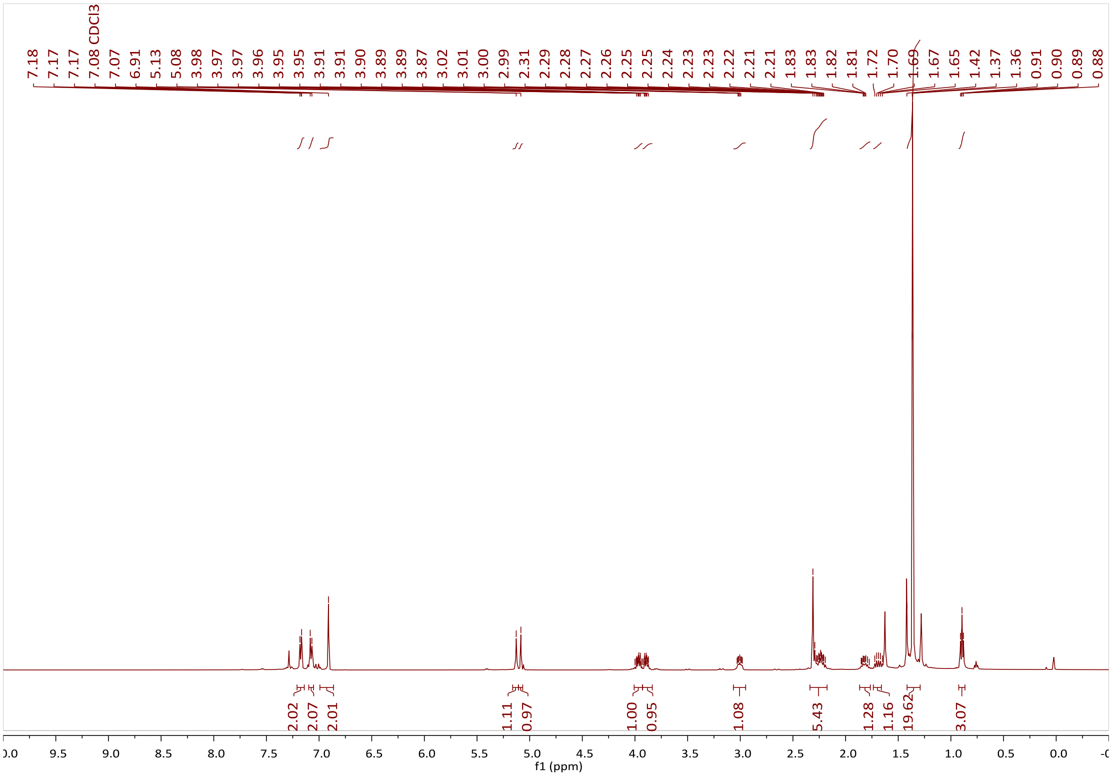
7.18
7.17
7.17
7.08 CDCl3
7.07
6.91
5.13
5.08
3.98
3.97
3.97
3.96
3.95
3.95
3.91
3.91
3.90
3.89
3.89
3.87
3.02
3.01
3.00
2.99
2.31
2.29
2.28
2.27
2.26
2.25
2.25
2.24
2.23
2.23
2.22
2.21
2.21
1.83
1.83
1.82
1.81
1.72
1.70
1.69
1.67
1.65
1.42
1.37
1.36
0.91
0.90
0.89
0.88
2.02
2.07
2.01
1.11
0.97
1.00
0.95
1.08
5.43
1.28
1.16
19.62
3.07
f1 (ppm)

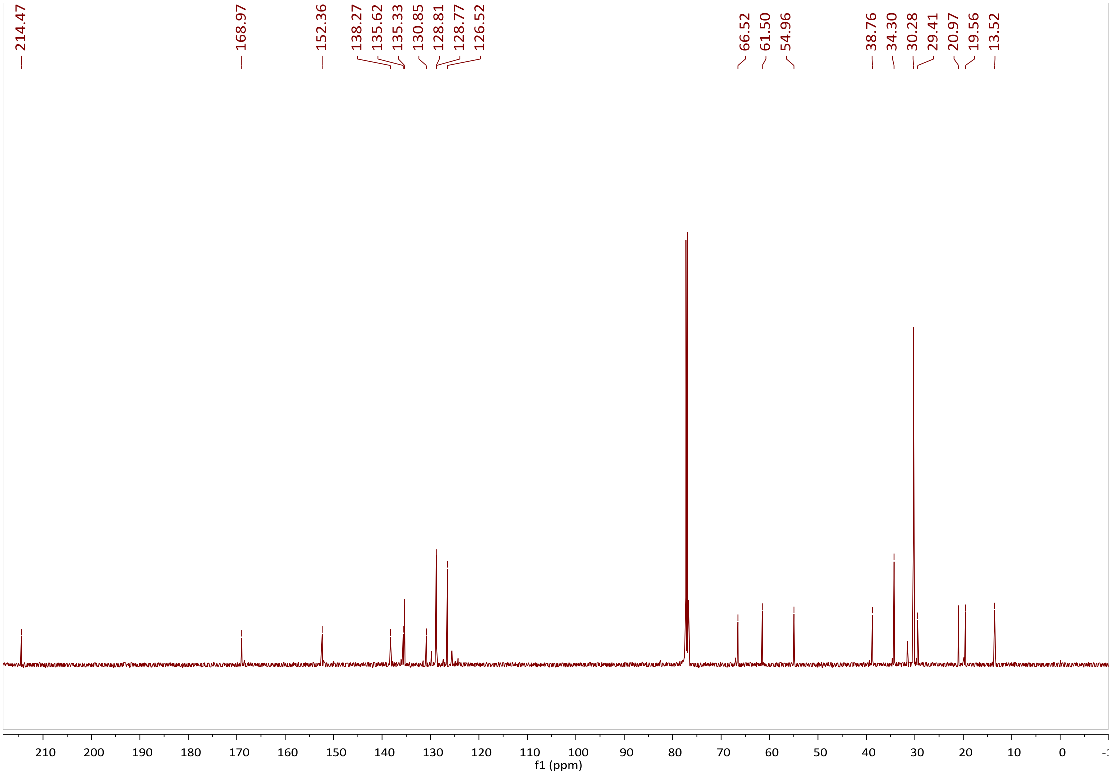
214.47
168.97
152.36
138.27
135.62
135.33
130.85
128.81
128.77
126.52
66.52
61.50
54.96
38.76
34.30
30.28
29.41
20.97
19.56
13.52
f1 (ppm)

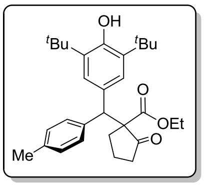

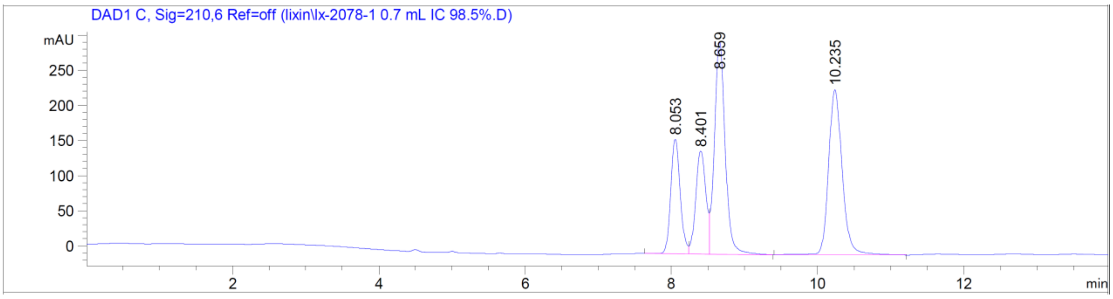

Peak #	RetTime [min]	Type	Width [min]	Area [mAU*s]	Height [mAU]	Area %
1	8.053	BV	0.1393	1469.94873	162.84169	16.2736
2	8.401	VV	0.1483	1408.34753	146.34930	15.5916
3	8.659	VB	0.1562	3108.70801	301.64221	34.4160
4	10.235	BB	0.1994	3045.72998	234.64156	33.7188

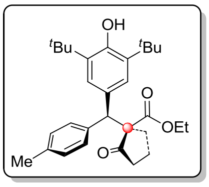

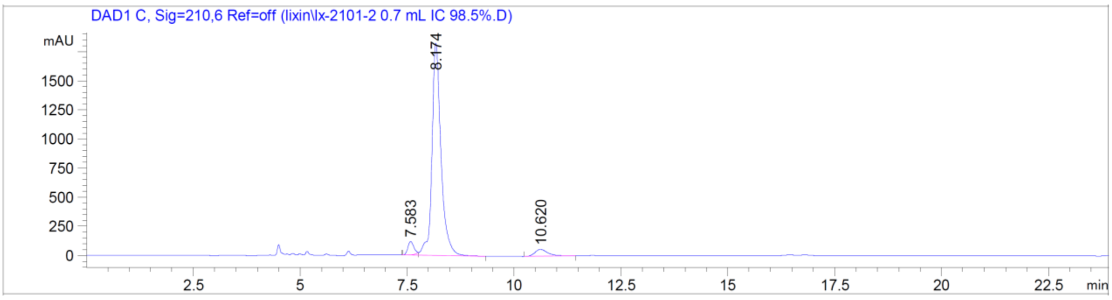

Peak #	RetTime [min]	Type	Width [min]	Area [mAU*s]	Height [mAU]	Area %
1	7.583	BV E	0.1473	1089.11060	114.17785	3.7069
2	8.174	VB R	0.2209	2.70492e4	1824.96887	92.0642
3	10.620	BB	0.3199	1242.48865	58.93825	4.2289

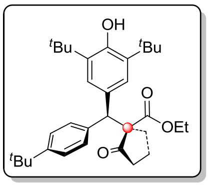
OH
tBu
tBu
O
OEt
O
tBu

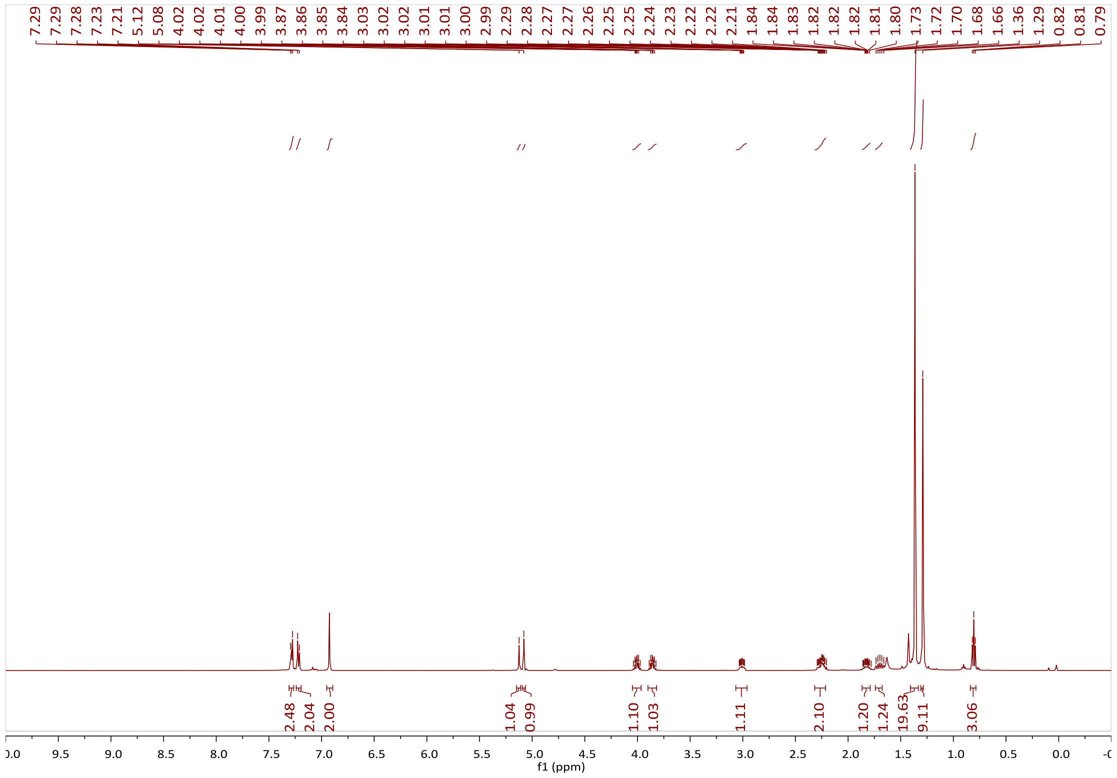

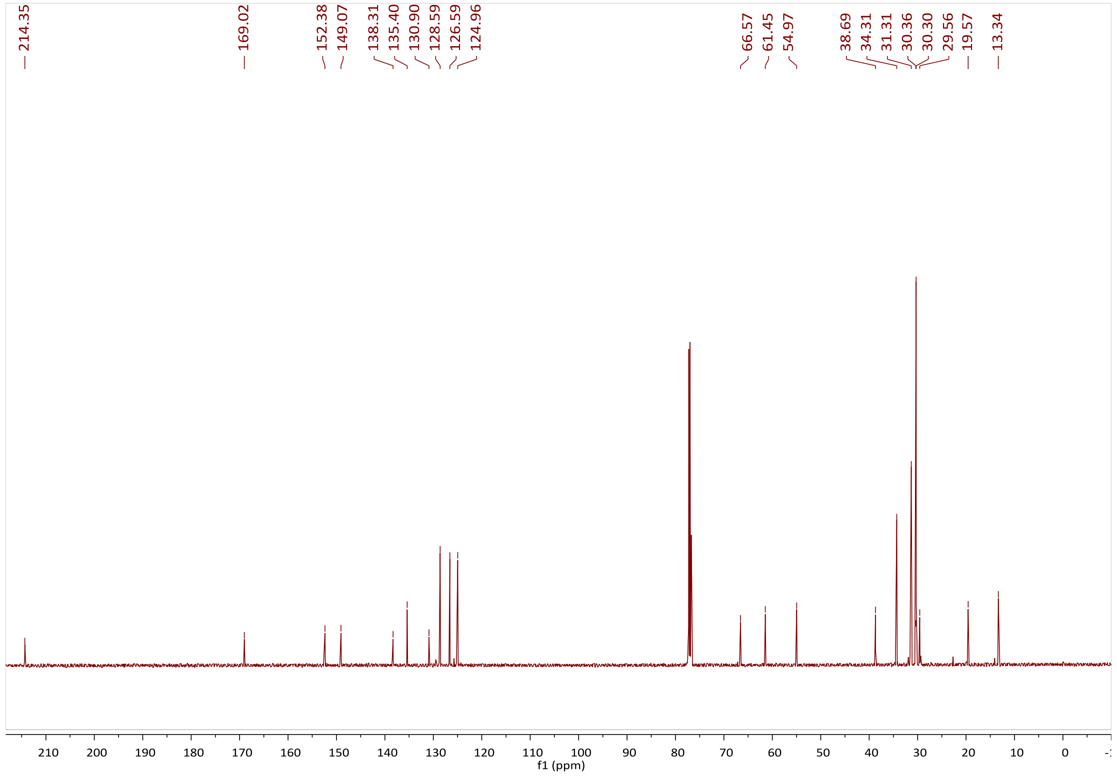

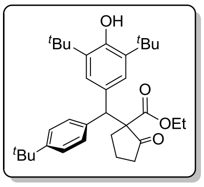

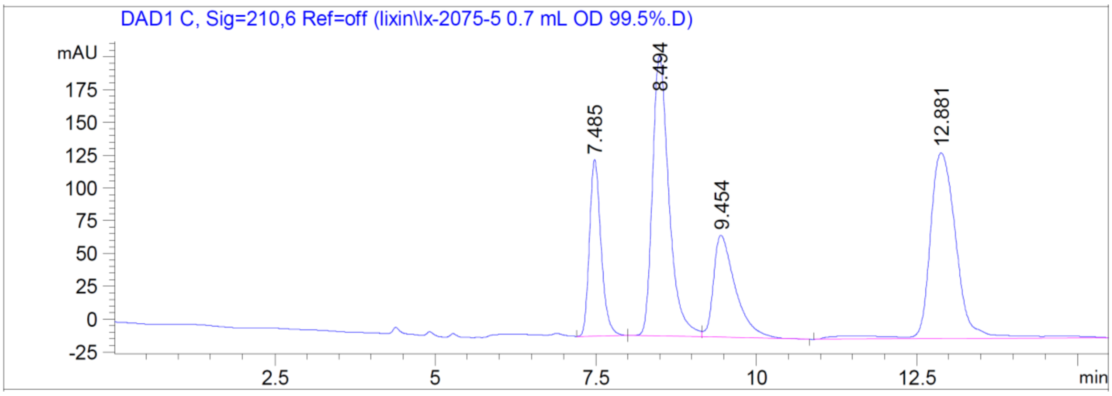

Peak #	RetTime [min]	Type	Width [min]	Area [mAU*s]	Height [mAU]	Area %
1	7.485	BB	0.1945	1685.98926	134.21985	14.6415
2	8.494	BV	0.2741	3839.83081	213.21825	33.3459
3	9.454	VB	0.3506	1855.95483	77.71581	16.1175
4	12.881	VV R	0.4553	4133.36426	141.59445	35.8950

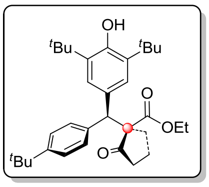

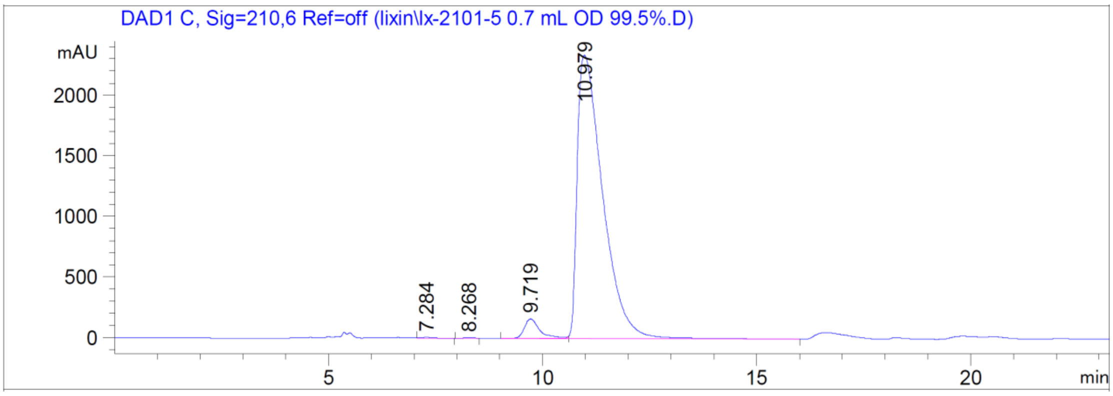

Peak #	RetTime [min]	Type	Width [min]	Area [mAU*s]	Height [mAU]	Area %
1	7.284	VB	0.3122	154.31569	7.25509	0.1529
2	8.268	BV	0.3044	80.89815	4.20634	0.0801
3	9.719	BV E	0.3839	4106.14502	161.89053	4.0682
4	10.979	VB R	0.6259	9.65923e4	2338.31738	95.6988

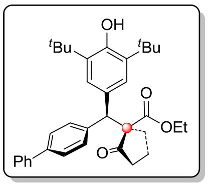
OH
tBu
tBu
O
OEt
Ph
O

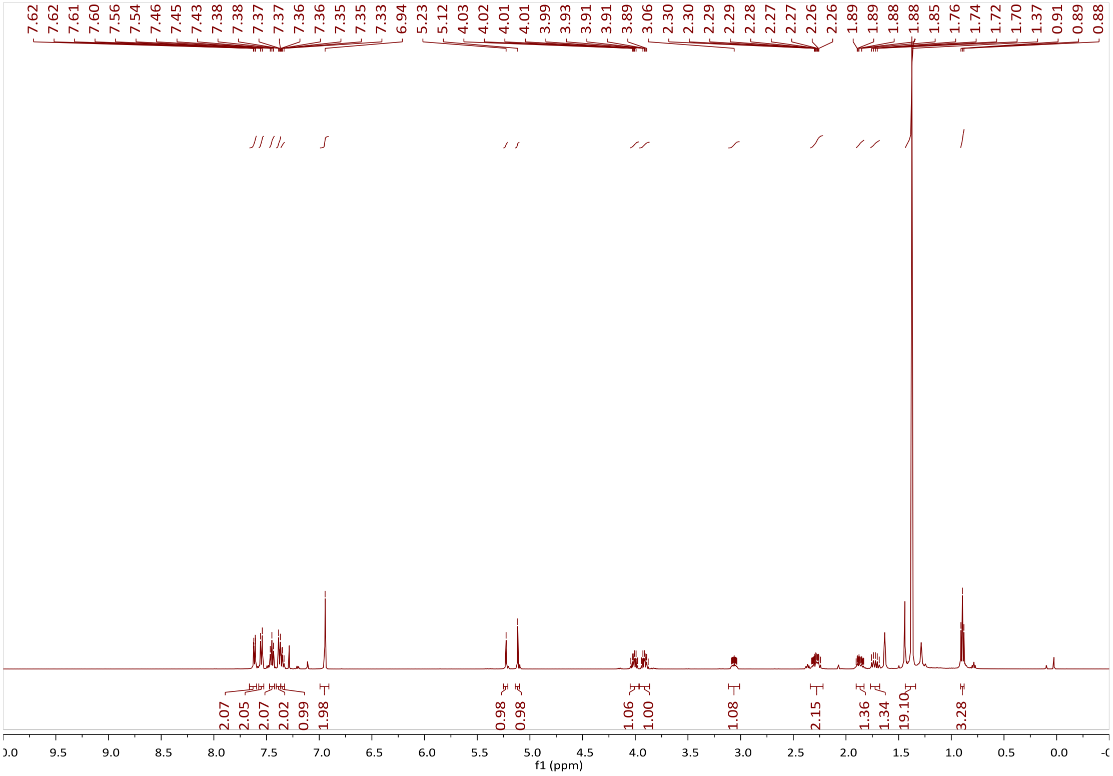
7.62 7.62 7.61 7.60 7.56 7.54 7.46 7.45 7.43 7.38 7.38 7.37 7.37 7.36 7.36 7.35 7.33 6.94 5.23 5.12 4.03 4.02 4.01 4.01 3.99 3.93 3.91 3.91 3.89 3.06 2.30 2.30 2.29 2.29 2.28 2.27 2.27 2.26 2.26 1.89 1.89 1.88 1.88 1.85 1.76 1.74 1.72 1.70 1.37 0.91 0.89 0.88
2.07 2.05 2.07 2.02 0.99 1.98 0.98 0.98 1.06 1.00 1.08 2.15 1.36 1.34 19.10 3.28
f1 (ppm)

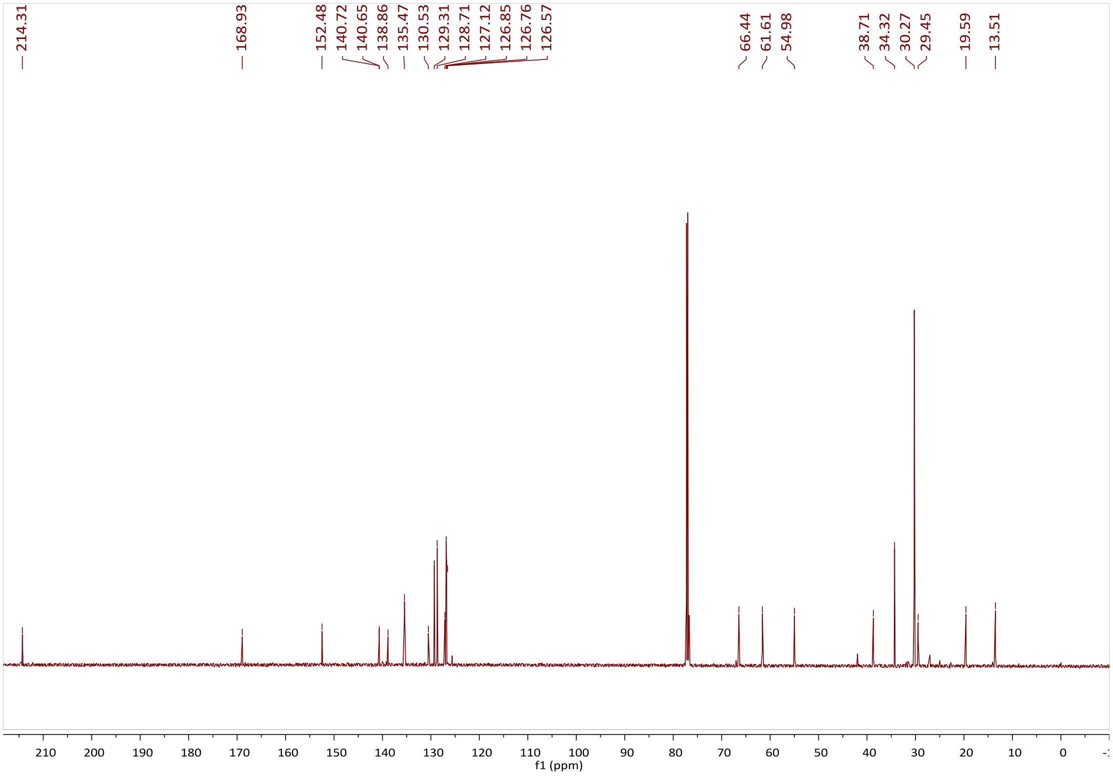
214.31 168.93 152.48 140.72 140.65 138.86 135.47 130.53 129.31 128.71 127.12 126.85 126.76 126.57 66.44 61.61 54.98 38.71 34.32 30.27 29.45 19.59 13.51
f1 (ppm)

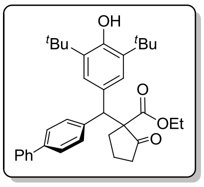

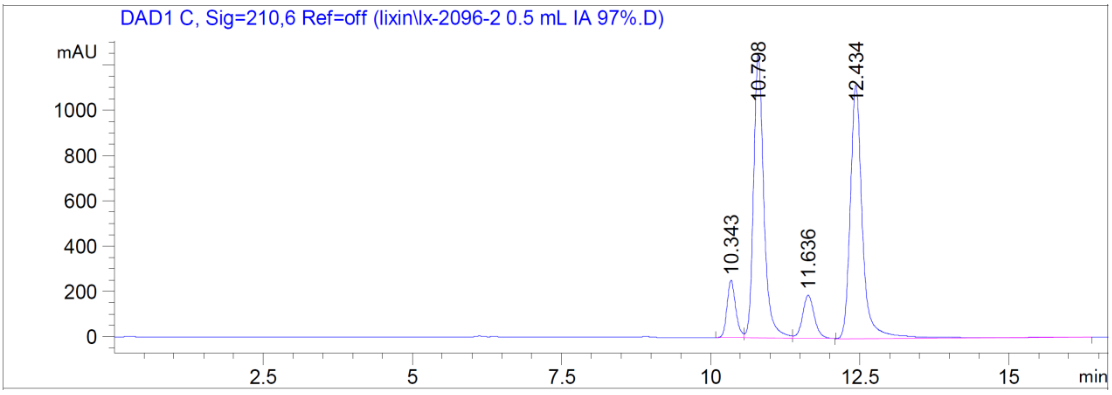

Peak #	RetTime [min]	Type	Width [min]	Area [mAU*s]	Height [mAU]	Area %
1	10.343	BV	0.1582	2545.32349	251.29065	7.0564
2	10.798	VV	0.1886	1.49702e4	1242.49036	41.5019
3	11.636	VB	0.2154	2597.66016	190.15742	7.2015
4	12.434	BV R	0.2153	1.59579e4	1113.09985	44.2402

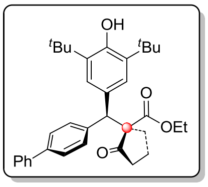

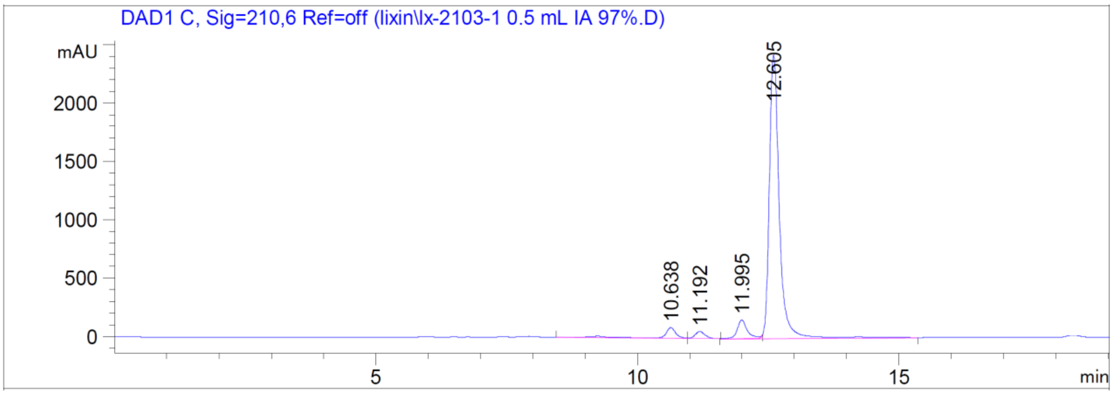

Peak #	RetTime [min]	Type	Width [min]	Area [mAU*s]	Height [mAU]	Area %
1	10.638	VV R	0.1962	1749.04431	91.86221	4.7290
2	11.192	VB	0.2039	811.98242	60.77063	2.1954
3	11.995	BV E	0.1958	2137.88135	160.11205	5.7803
4	12.605	VV R	0.2024	3.22869e4	2424.33447	87.2954

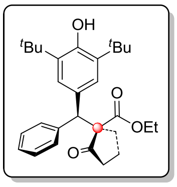
OH
tBu
tBu
O
OEt
O

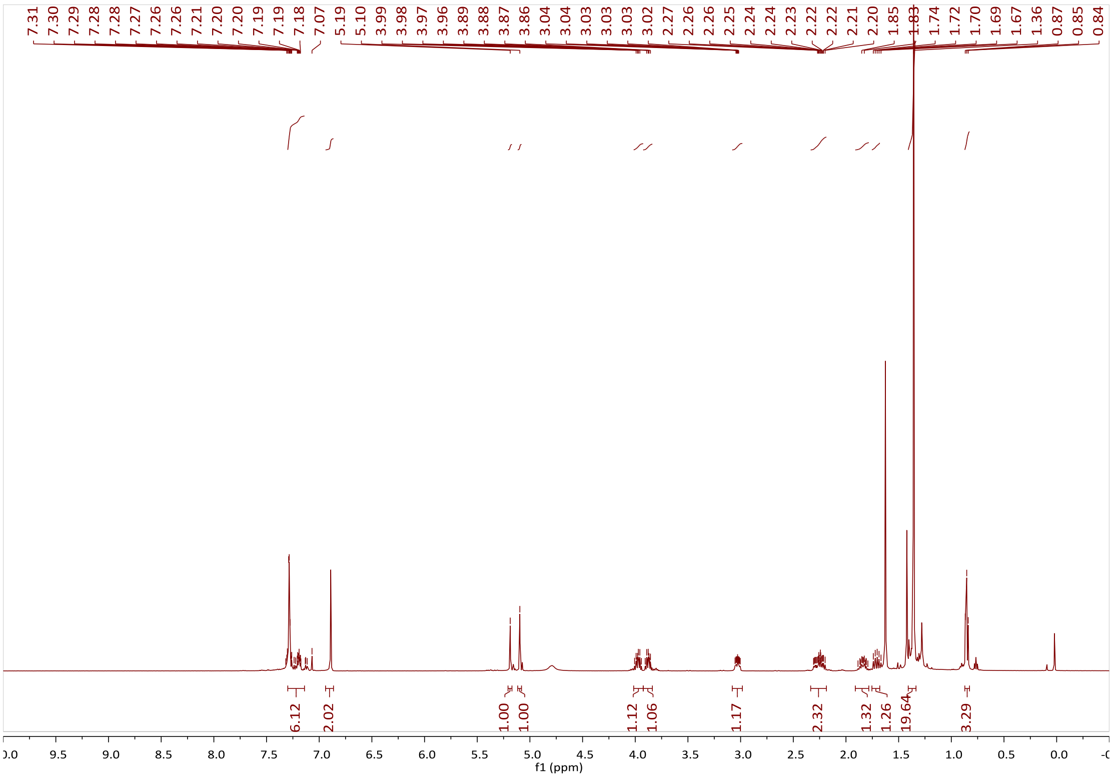
7.31 7.30 7.29 7.28 7.28 7.27 7.26 7.26 7.21 7.20 7.20 7.19 7.19 7.18 7.07 5.19 5.10 3.99 3.98 3.97 3.96 3.89 3.88 3.87 3.86 3.04 3.04 3.03 3.03 3.02 2.27 2.26 2.26 2.25 2.24 2.24 2.23 2.22 2.22 2.21 2.20 1.85 1.83 1.74 1.72 1.70 1.69 1.67 1.36 0.87 0.85 0.84
6.12
2.02
1.00
1.00
1.12
1.06
1.17
2.32
1.32
1.26
19.64
3.29
f1 (ppm)

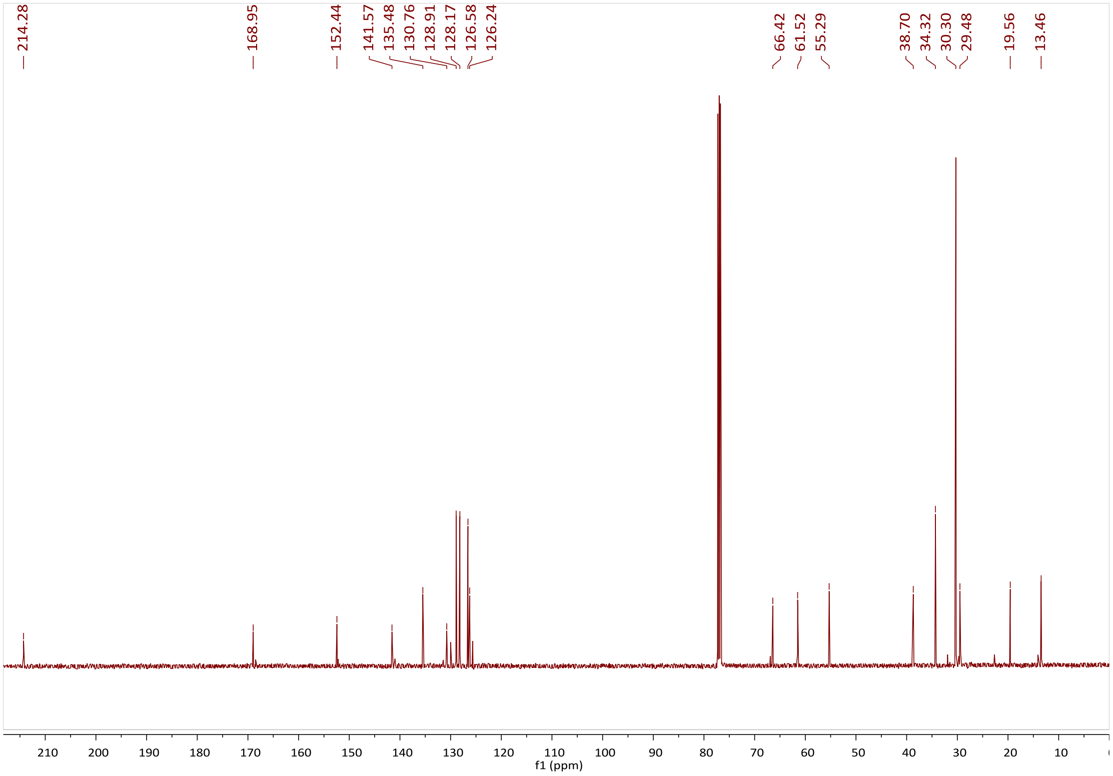
214.28
168.95
152.44
141.57 135.48 130.76 128.91 128.17 126.58 126.24
66.42 61.52 55.29
38.70 34.32 30.30 29.48
19.56
13.46
f1 (ppm)

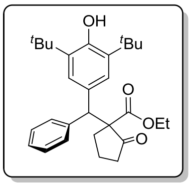

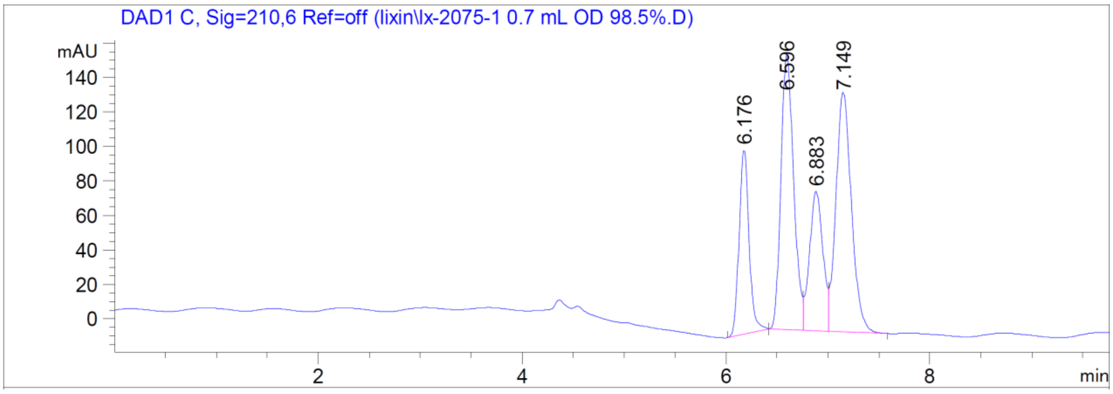

Peak #	RetTime [min]	Type	Width [min]	Area [mAU*s]	Height [mAU]	Area %
1	6.176	BB	0.1052	725.00977	106.77317	16.7564
2	6.596	BV	0.1333	1388.50879	159.85616	32.0911
3	6.883	VV	0.1386	739.15875	80.86410	17.0834
4	7.149	VB	0.1617	1474.09473	138.98164	34.0692

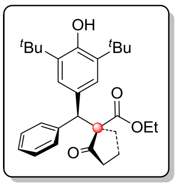

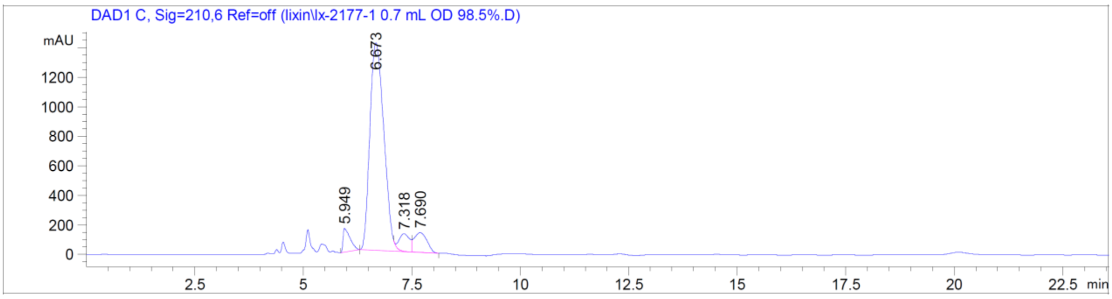

Peak #	RetTime [min]	Type	Width [min]	Area [mAU*s]	Height [mAU]	Area %
1	5.949	BB	0.1470	1819.43652	162.08327	4.7688
2	6.673	BV R	0.3605	3.16453e4	1406.62390	82.9430
3	7.318	VV E	0.2454	1929.78821	118.86016	5.0580
4	7.690	VB E	0.3269	2758.56665	133.58318	7.2303

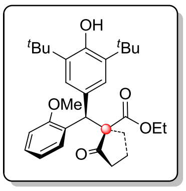
OH
tBu
tBu
OMe
O
OEt
O

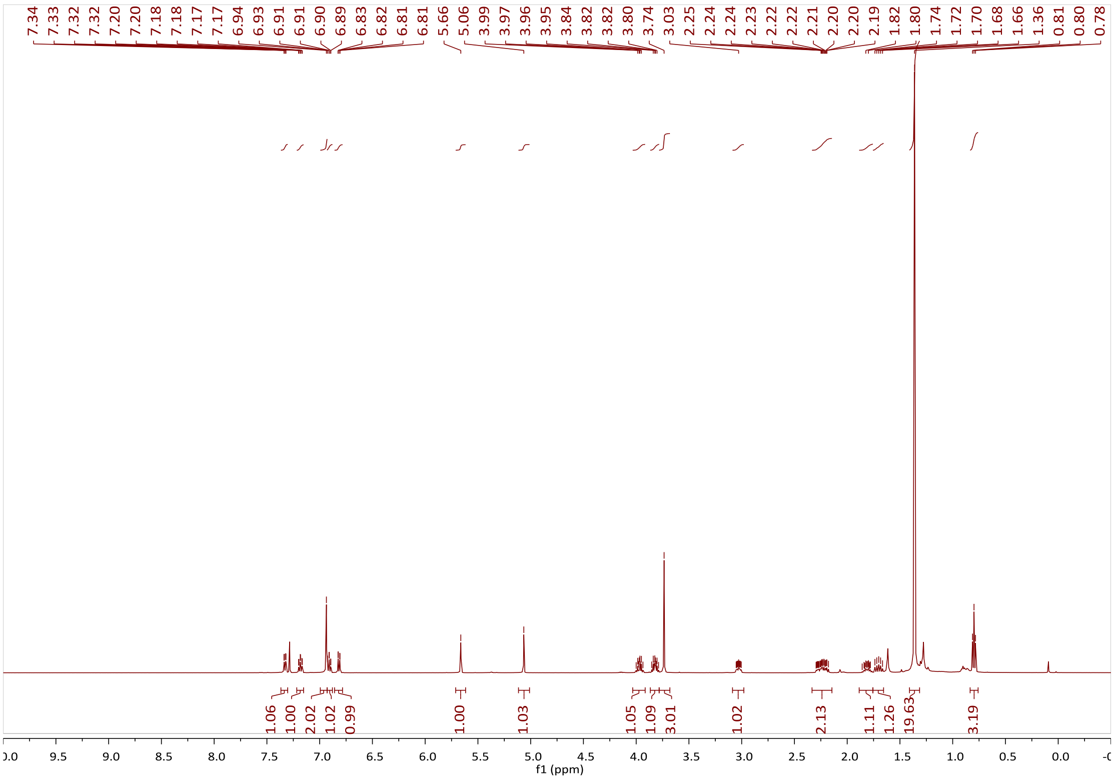
7.34 7.33 7.32 7.32 7.20 7.20 7.18 7.18 7.17 7.17 6.94 6.93 6.91 6.91 6.90 6.89 6.83 6.82 6.81 6.81 5.66 5.06 3.99 3.97 3.96 3.95 3.84 3.82 3.82 3.80 3.74 3.03 2.25 2.24 2.24 2.23 2.22 2.22 2.21 2.20 2.20 2.19 1.82 1.80 1.74 1.72 1.70 1.68 1.66 1.36 0.81 0.80 0.78
1.06 1.00 2.02 1.02 0.99 1.00 1.03 1.05 1.09 3.01 1.02 2.13 1.11 1.26 19.63 3.19
f1 (ppm)

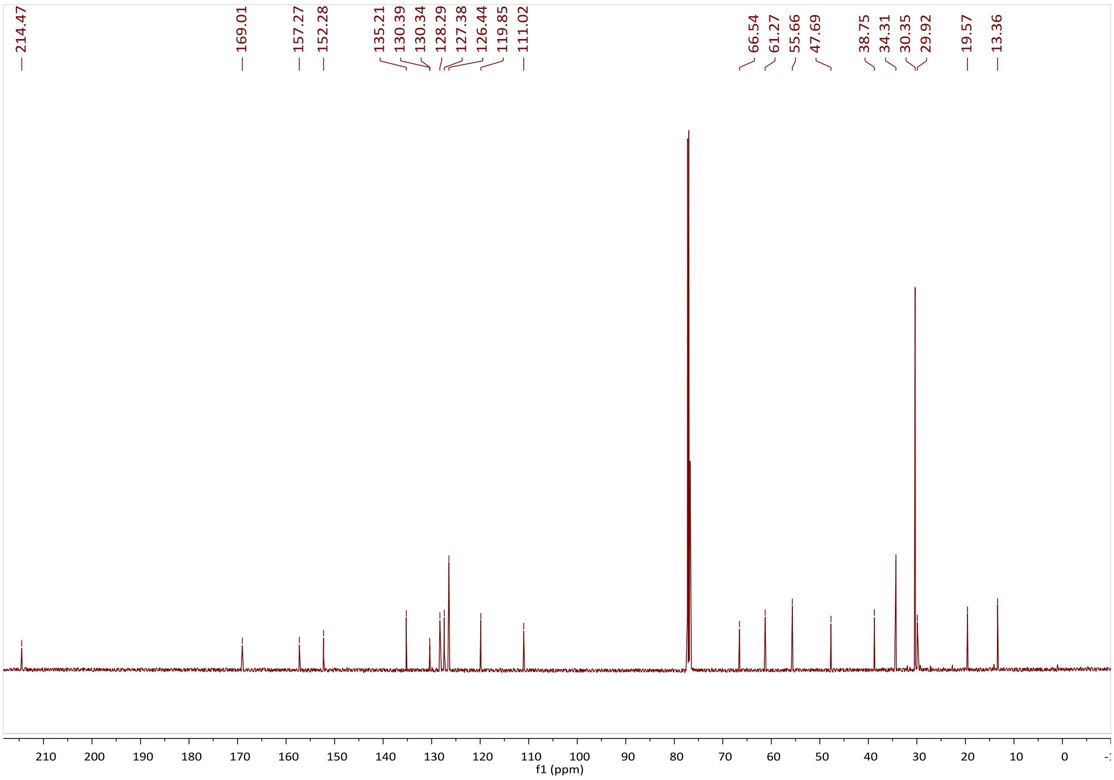
214.47
169.01
157.27
152.28
135.21 130.39 130.34 128.29 127.38 126.44 119.85
111.02
66.54 61.27 55.66 47.69
38.75 34.31 30.35 29.92
19.57
13.36
f1 (ppm)

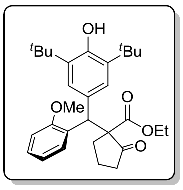

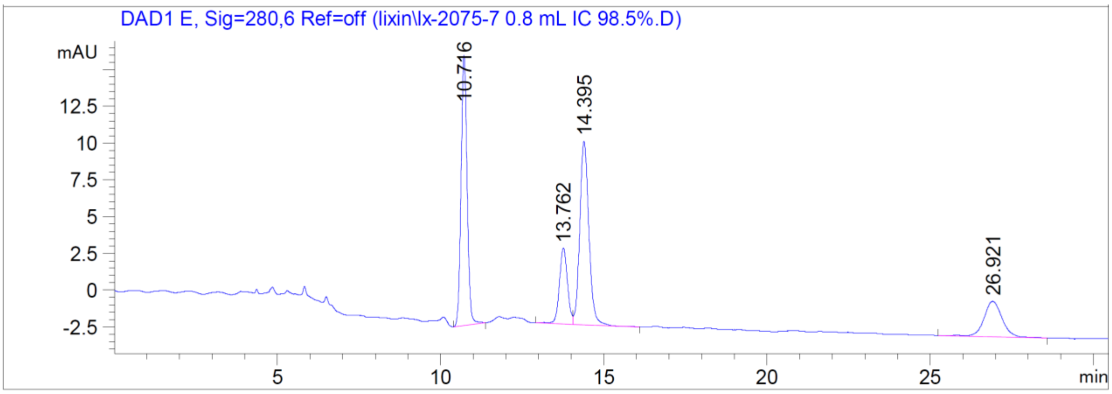

Peak #	RetTime [min]	Type	Width [min]	Area [mAU*s]	Height [mAU]	Area %
1	10.716	BB	0.1988	236.21201	18.27972	35.4655
2	13.762	BV	0.2671	89.55058	5.14507	13.4454
3	14.395	VB	0.3006	244.51947	12.47864	36.7128
4	26.921	VB R	0.5977	95.75100	2.42066	14.3763

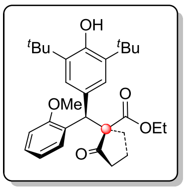

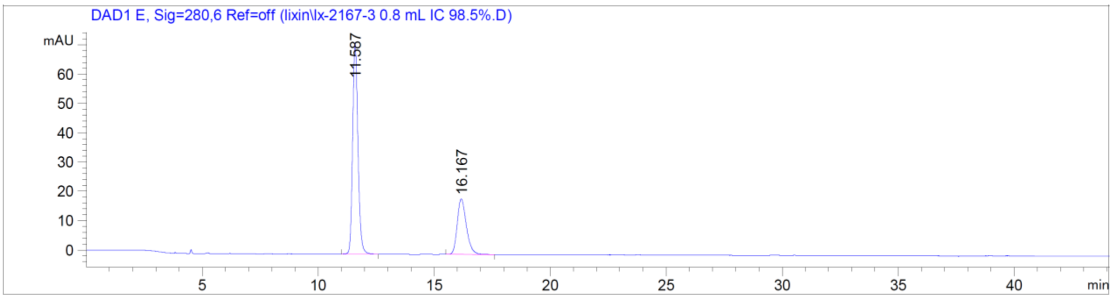

Peak #	RetTime [min]	Type	Width [min]	Area [mAU*s]	Height [mAU]	Area %
1	11.587	BB	0.2445	1153.12500	72.13361	68.8897
2	16.167	BB	0.4199	520.74725	18.99386	31.1103

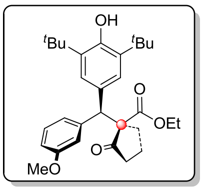
OH
tBu
tBu
O
OEt
O
MeO

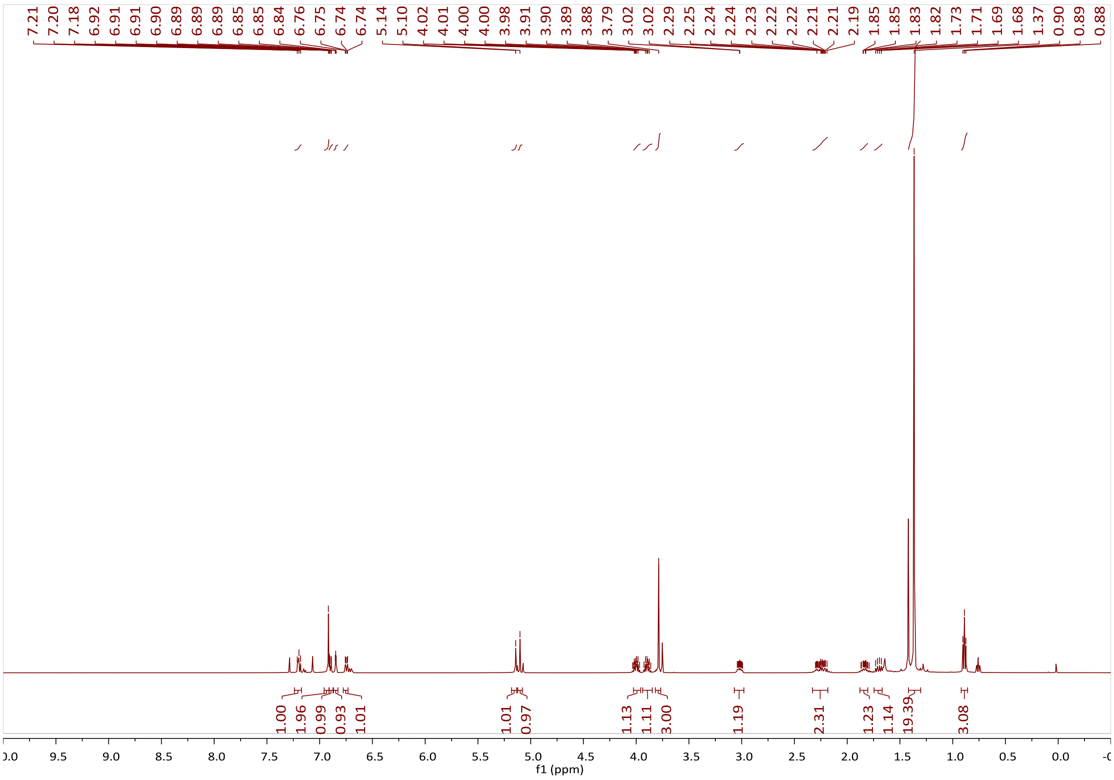
7.21
7.20
7.18
6.92
6.91
6.91
6.90
6.89
6.89
6.89
6.85
6.85
6.84
6.76
6.75
6.74
6.74
5.14
5.10
4.02
4.01
4.00
4.00
3.98
3.91
3.90
3.89
3.88
3.79
3.02
3.02
2.29
2.25
2.24
2.24
2.23
2.22
2.22
2.21
2.21
2.19
1.85
1.85
1.83
1.82
1.73
1.71
1.69
1.68
1.37
0.90
0.89
0.88
1.00
1.96
0.99
0.93
1.01
1.01
0.97
1.13
1.11
3.00
1.19
2.31
1.23
1.14
19.39
3.08
9.5
9.0
8.5
8.0
7.5
7.0
6.5
6.0
5.5
5.0
4.5
4.0
3.5
3.0
2.5
2.0
1.5
1.0
0.5
0.0
f1 (ppm)

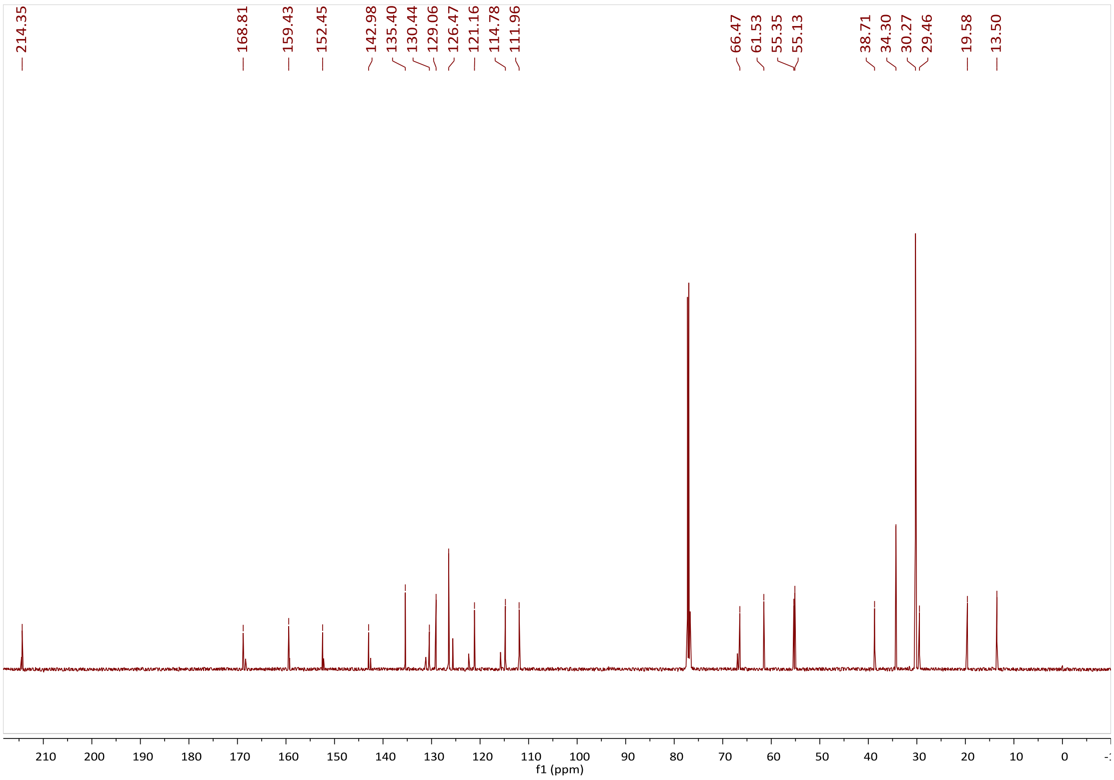
214.35
168.81
159.43
152.45
142.98
135.40
130.44
129.06
126.47
121.16
114.78
111.96
66.47
61.53
55.35
55.13
38.71
34.30
30.27
29.46
19.58
13.50
210
200
190
180
170
160
150
140
130
120
110
100
90
80
70
60
50
40
30
20
10
0
f1 (ppm)

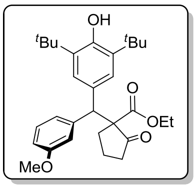

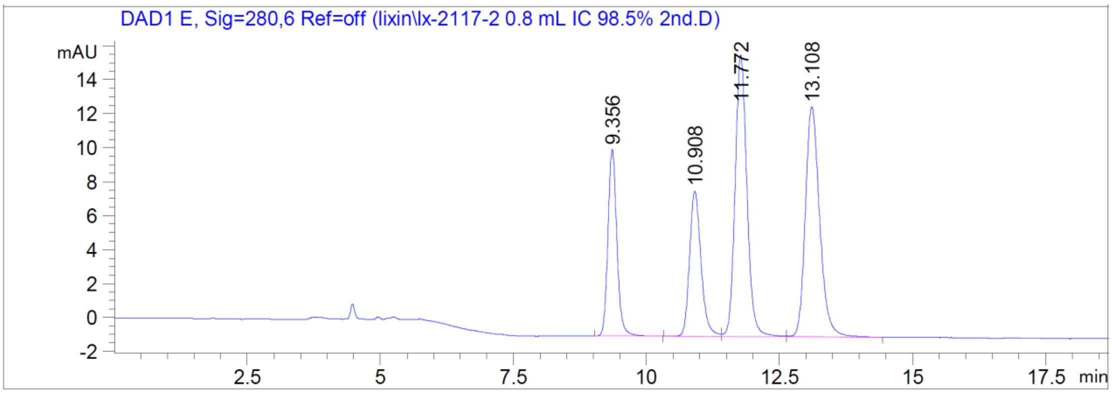

Peak #	RetTime [min]	Type	Width [min]	Area [mAU*s]	Height [mAU]	Area %
1	9.356	BB	0.1847	128.20503	10.94332	16.7026
2	10.908	BV	0.2319	128.39276	8.51333	16.7271
3	11.772	VV	0.2371	256.56372	16.52964	33.4253
4	13.108	VB	0.2913	254.41156	13.53224	33.1449

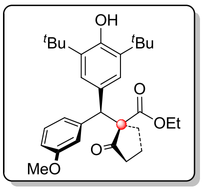

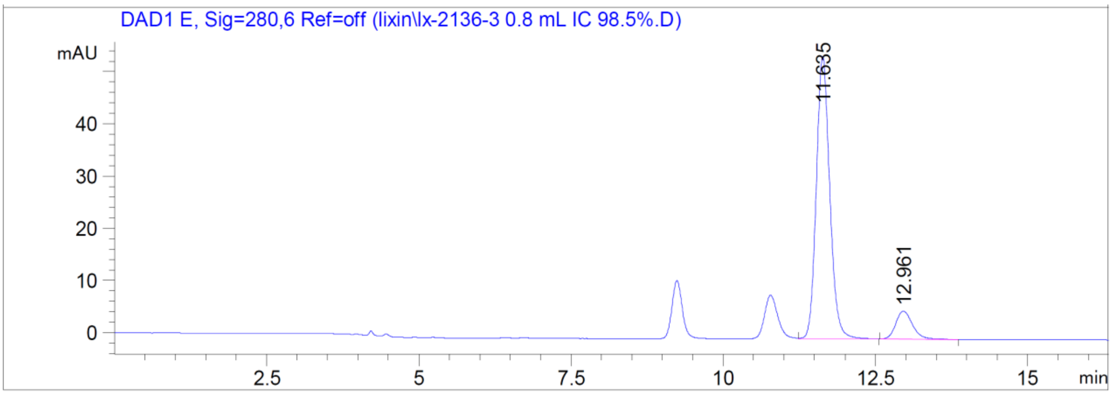

Peak #	RetTime [min]	Type	Width [min]	Area [mAU*s]	Height [mAU]	Area %
1	11.635	VB	0.2361	833.05762	53.95769	89.0880
2	12.961	BB	0.2949	102.03762	5.34180	10.9120

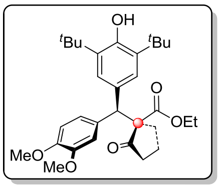
OH
tBu
tBu
O
OEt
O
MeO
MeO

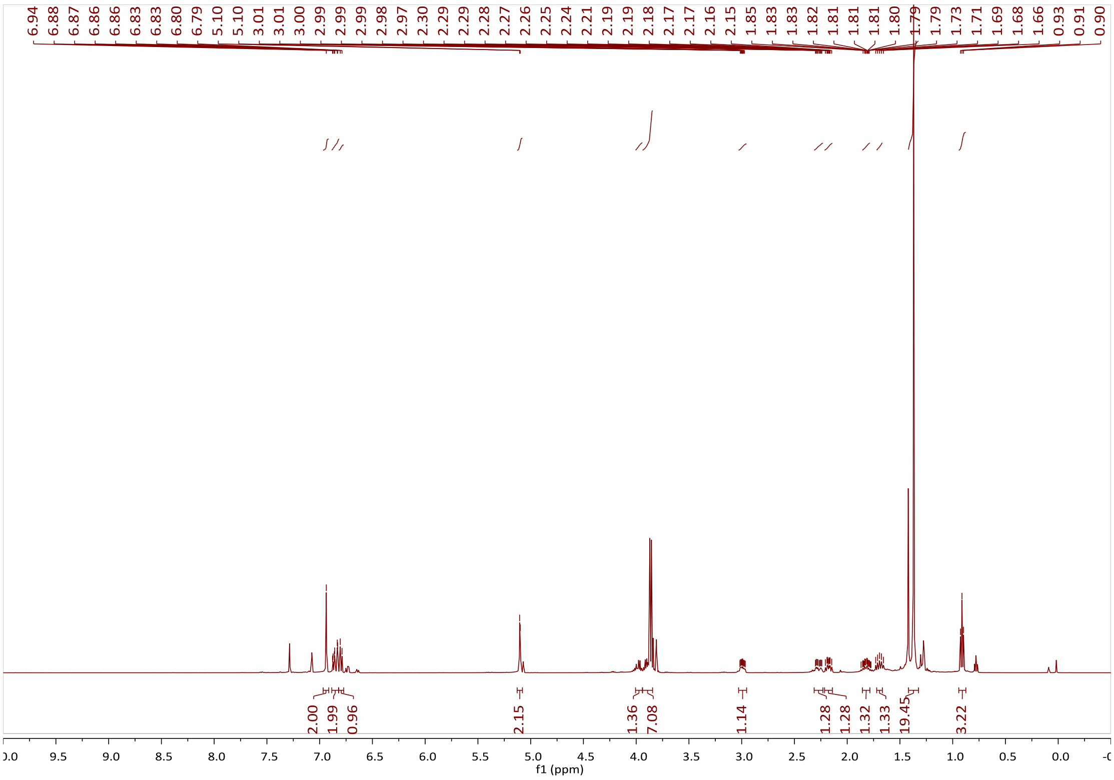
6.94
6.88
6.87
6.86
6.86
6.83
6.83
6.80
6.79
5.10
5.10
3.01
3.00
2.99
2.99
2.99
2.98
2.97
2.30
2.29
2.29
2.28
2.27
2.26
2.25
2.24
2.21
2.19
2.19
2.18
2.17
2.17
2.16
2.15
1.85
1.83
1.83
1.82
1.81
1.81
1.81
1.80
1.79
1.79
1.73
1.71
1.69
1.68
1.66
0.93
0.91
0.90
2.00
1.99
0.96
2.15
1.36
7.08
1.14
1.28
1.28
1.32
1.33
19.45
3.22
f1 (ppm)

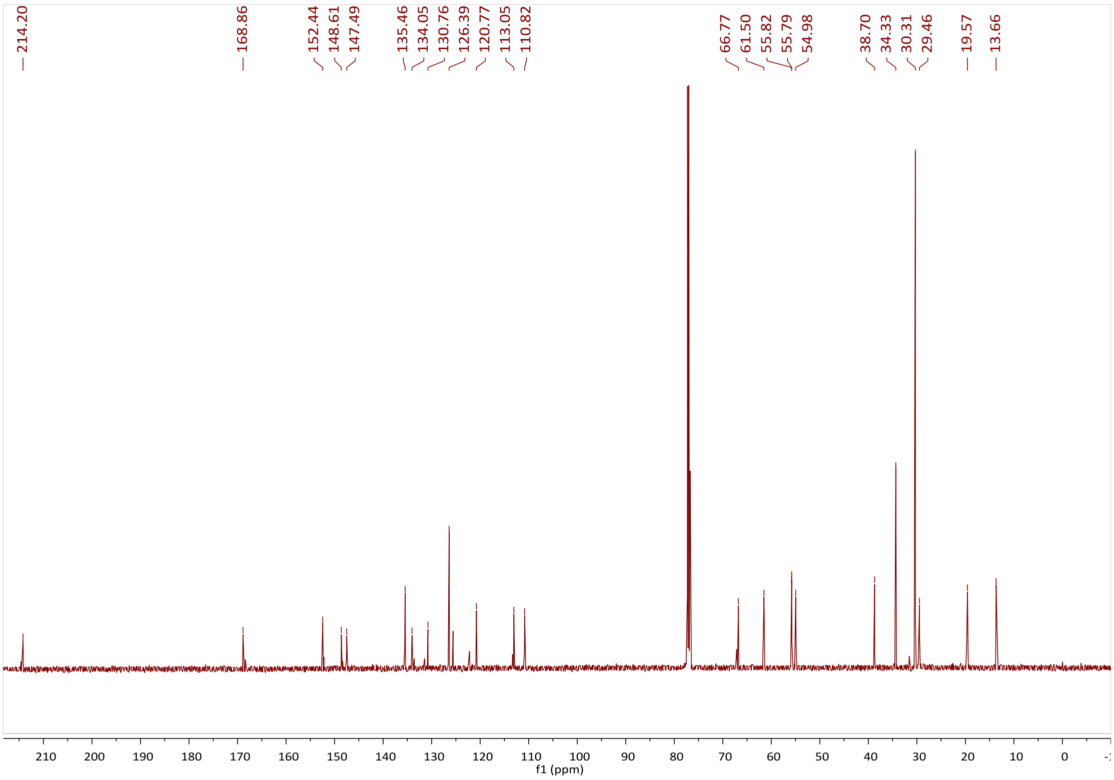
214.20
168.86
152.44
148.61
147.49
135.46
134.05
130.76
126.39
120.77
113.05
110.82
66.77
61.50
55.82
55.79
54.98
38.70
34.33
30.31
29.46
19.57
13.66
f1 (ppm)

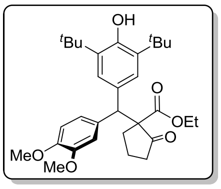

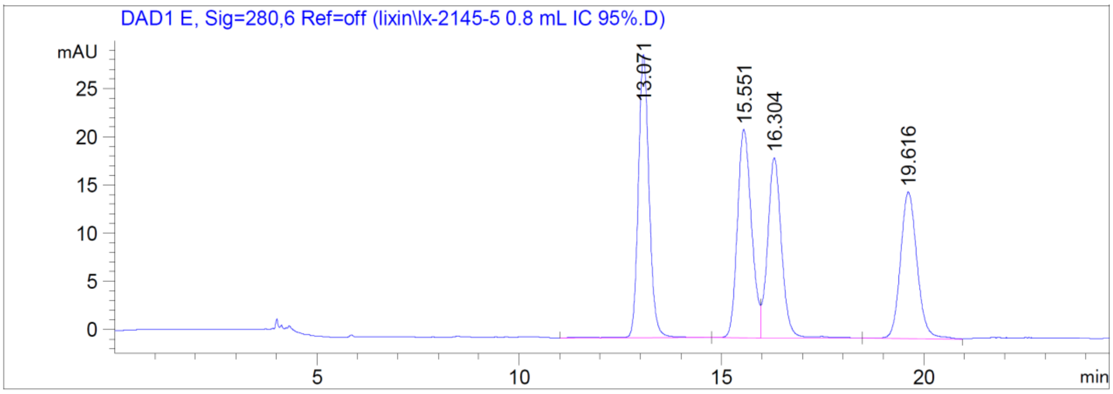

Peak #	RetTime [min]	Type	Width [min]	Area [mAU*s]	Height [mAU]	Area %
1	13.071	VB R	0.2810	547.38855	29.29502	28.0284
2	15.551	BV	0.3786	522.79639	21.58084	26.7692
3	16.304	VV R	0.3655	449.12469	18.66358	22.9969
4	19.616	BB	0.4368	433.66684	15.20045	22.2054

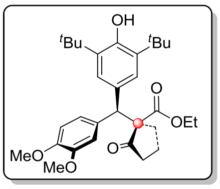

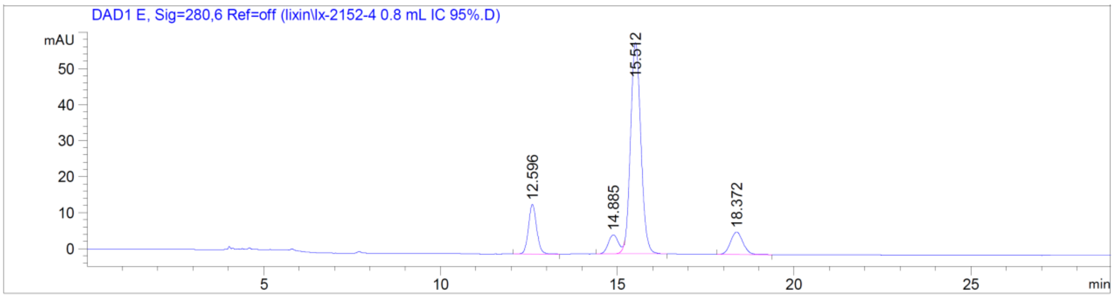

Peak #	RetTime [min]	Type	Width [min]	Area [mAU*s]	Height [mAU]	Area %
1	12.596	BB	0.2507	223.46402	13.81766	13.2947
2	14.885	BV E	0.3046	104.52560	5.33441	6.2186
3	15.512	VB R	0.3163	1199.94775	58.73967	71.3896
4	18.372	BB	0.3792	152.90733	6.25530	9.0971

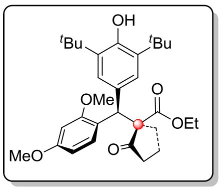

OH
tBu
tBu
OMe
O
OEt
MeO
O

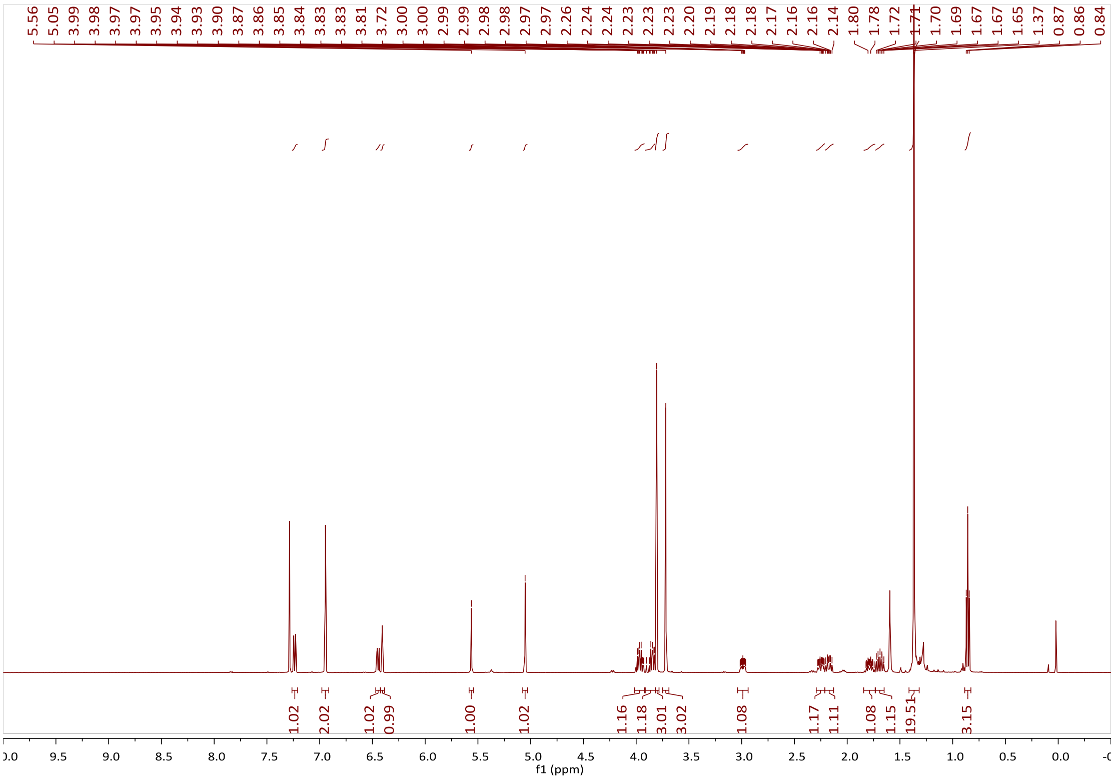

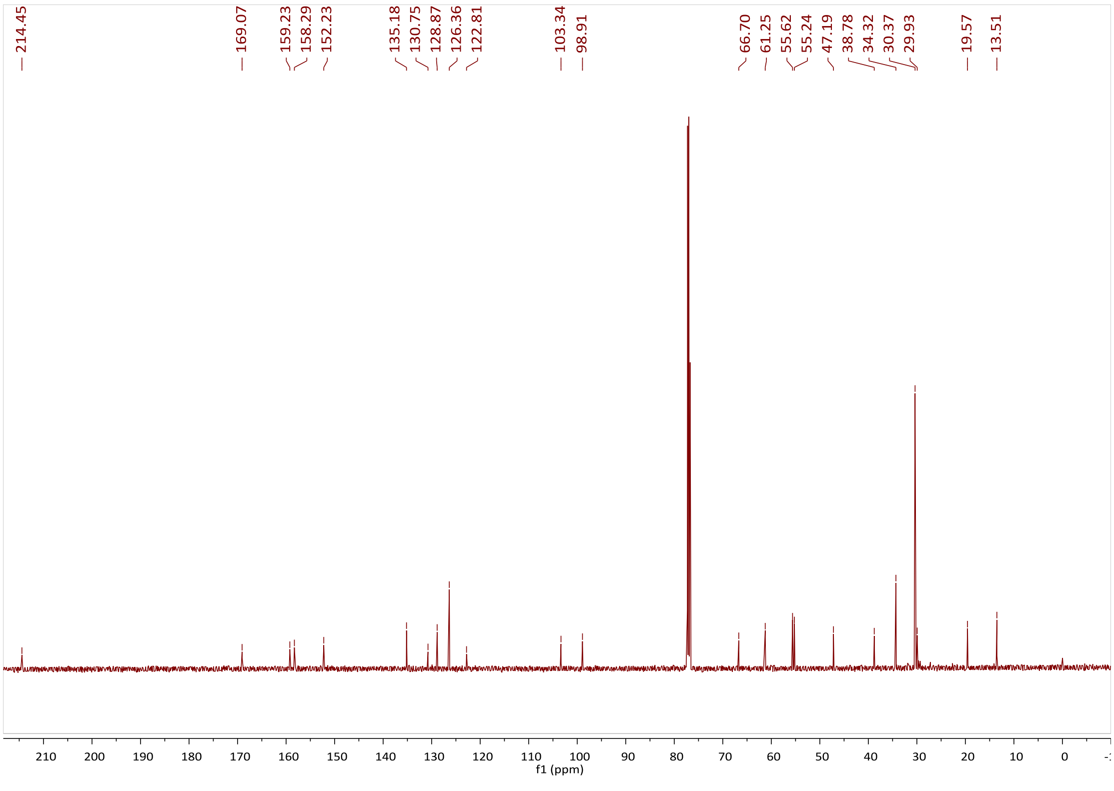

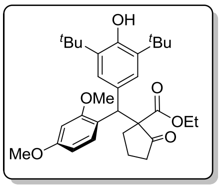

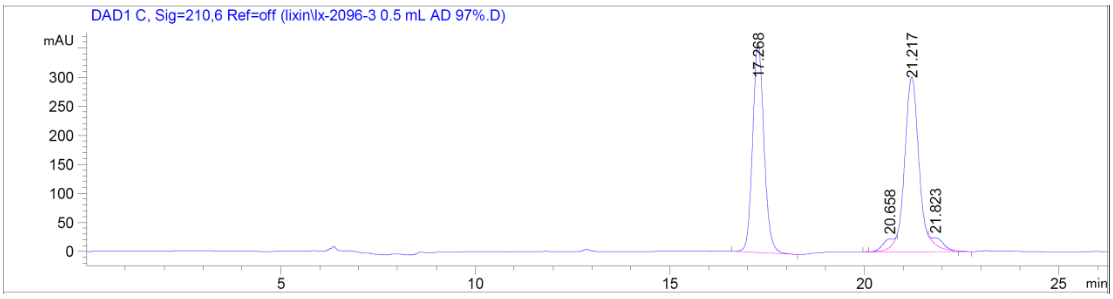

Peak #	RetTime [min]	Type	Width [min]	Area [mAU*s]	Height [mAU]	Area %
1	17.268	BB	0.3125	7324.68848	361.27548	46.1718
2	20.658	BV E	0.3022	290.96011	14.24978	1.8341
3	21.217	VV R	0.4022	7995.40674	300.68402	50.3997
4	21.823	VB E	0.3429	252.94128	10.81427	1.5944

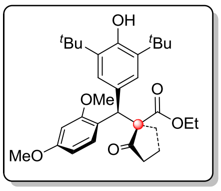

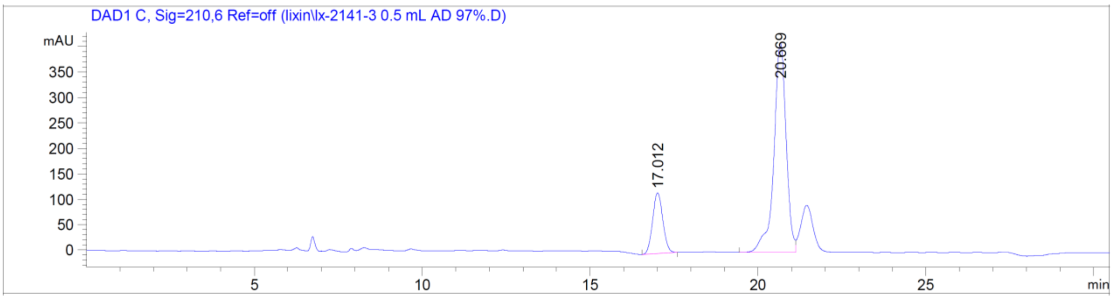

Peak #	RetTime [min]	Type	Width [min]	Area [mAU*s]	Height [mAU]	Area %
1	17.012	BB	0.3288	2523.56616	119.29267	19.4076
2	20.669	BV	0.3872	1.04794e4	411.35666	80.5924

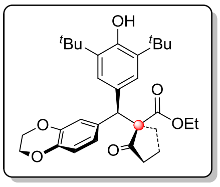
OH
^{t}Bu
^{t}Bu
O
OEt
O
O
O

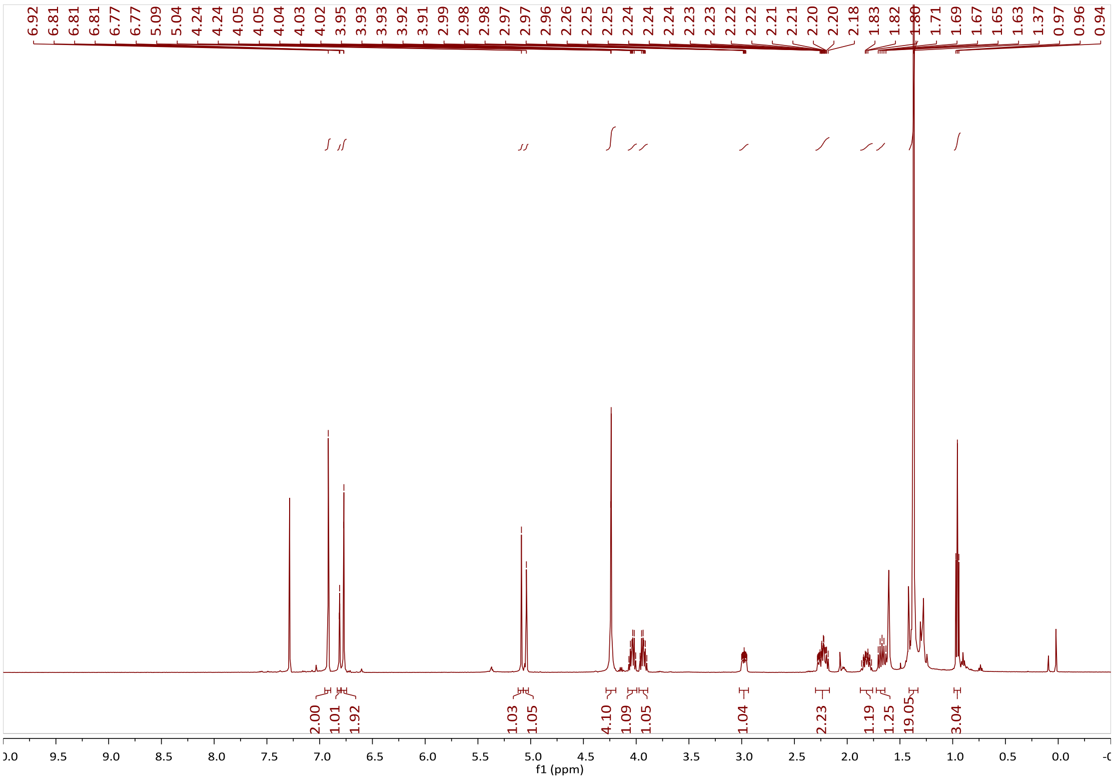
6.92 6.81 6.81 6.81 6.77 6.77 5.09 5.04 4.24 4.24 4.05 4.05 4.04 4.03 4.02 3.95 3.93 3.93 3.92 3.91 2.99 2.98 2.98 2.97 2.97 2.96 2.26 2.25 2.25 2.24 2.24 2.24 2.23 2.23 2.22 2.22 2.21 2.21 2.20 2.20 2.18 1.83 1.82 1.80 1.71 1.69 1.67 1.65 1.63 1.37 0.97 0.96 0.94
2.00 1.01 1.92 1.03 1.05 4.10 1.09 1.05 1.04 2.23 1.19 1.25 19.05 3.04
f1 (ppm)

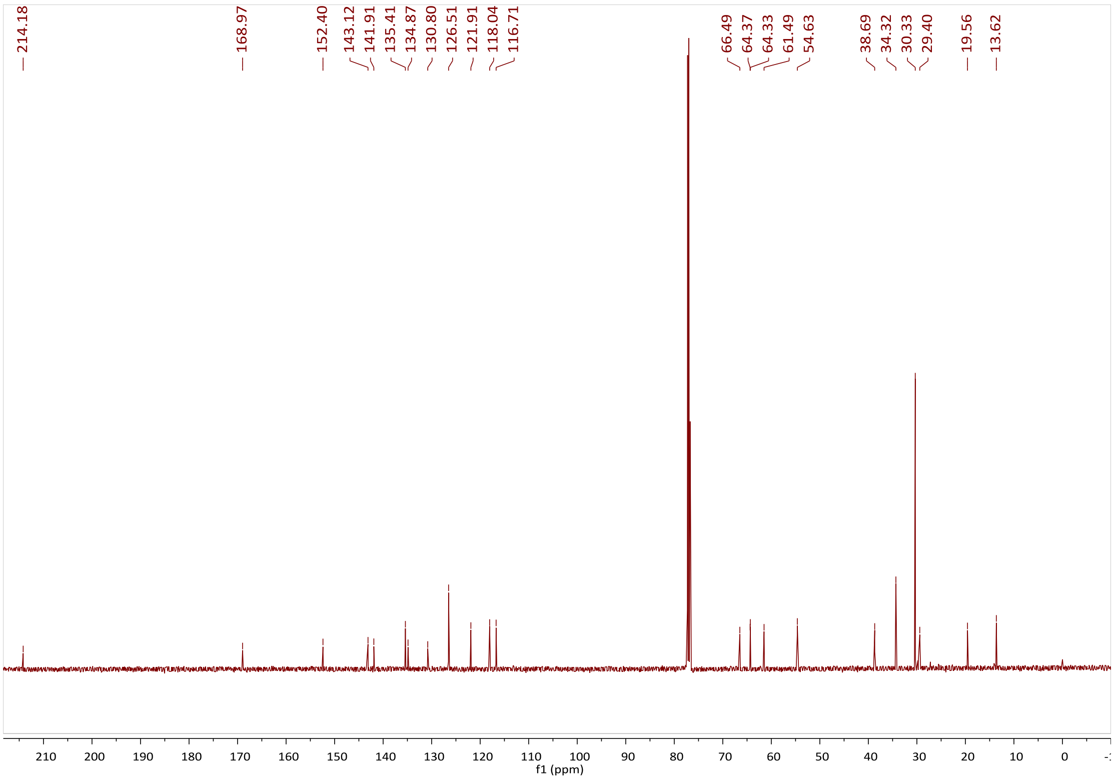
214.18 168.97 152.40 143.12 141.91 135.41 134.87 130.80 126.51 121.91 118.04 116.71 66.49 64.37 64.33 61.49 54.63 38.69 34.32 30.33 29.40 19.56 13.62
f1 (ppm)

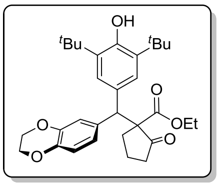

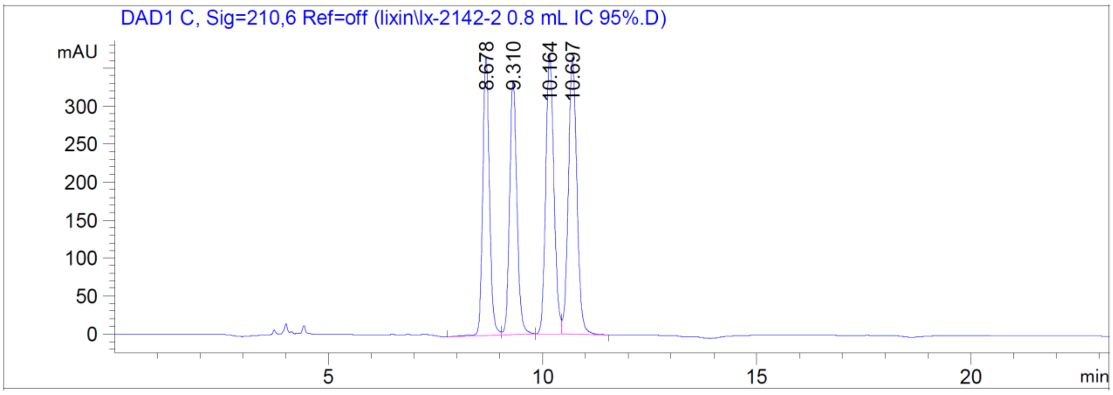

Peak #	RetTime [min]	Type	Width [min]	Area [mAU*s]	Height [mAU]	Area %
1	8.678	BV	0.1685	4002.18481	363.40536	22.3619
2	9.310	VB	0.1880	3957.82764	329.86627	22.1140
3	10.164	BV	0.2040	4882.44043	365.05630	27.2802
4	10.697	VB	0.2110	5054.92432	361.91153	28.2439

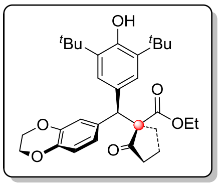

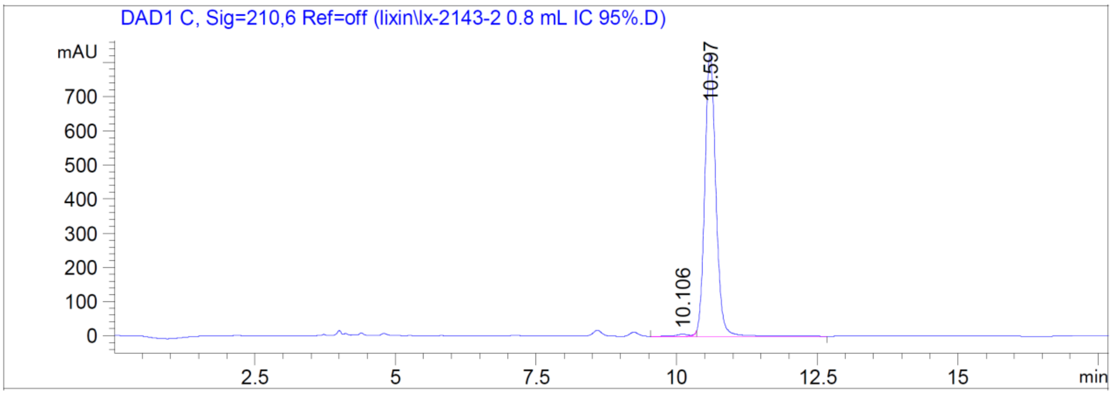

Peak #	RetTime [min]	Type	Width [min]	Area [mAU*s]	Height [mAU]	Area %
1	10.106	BV E	0.2402	101.62663	6.43828	0.8869
2	10.597	VB R	0.2173	1.13569e4	821.18353	99.1131

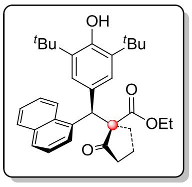

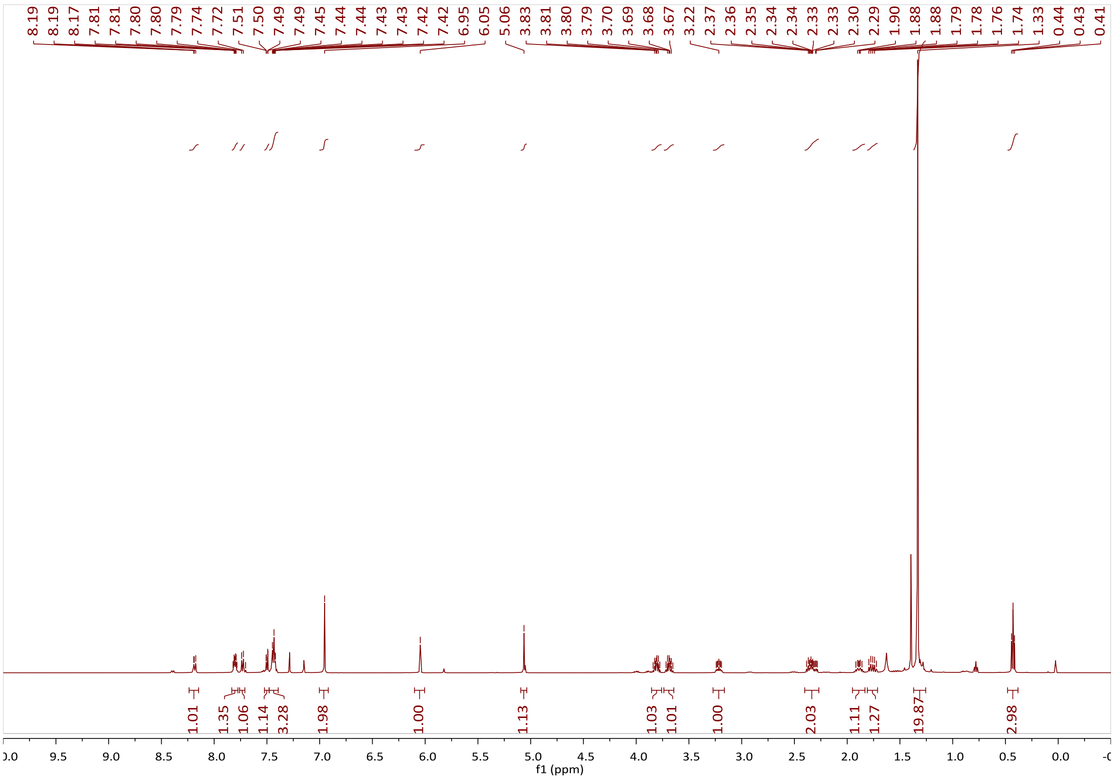
8.19
8.19
8.17
7.81
7.81
7.80
7.80
7.79
7.74
7.72
7.51
7.50
7.49
7.49
7.45
7.44
7.44
7.43
7.43
7.42
7.42
6.95
6.05
5.06
3.83
3.81
3.80
3.79
3.70
3.69
3.68
3.67
3.22
2.37
2.36
2.35
2.34
2.34
2.33
2.33
2.30
2.29
1.90
1.88
1.88
1.79
1.78
1.76
1.74
1.33
0.44
0.43
0.41
1.01
1.35
1.06
1.14
3.28
1.98
1.00
1.13
1.03
1.01
1.00
2.03
1.11
1.27
19.87
2.98
f1 (ppm)

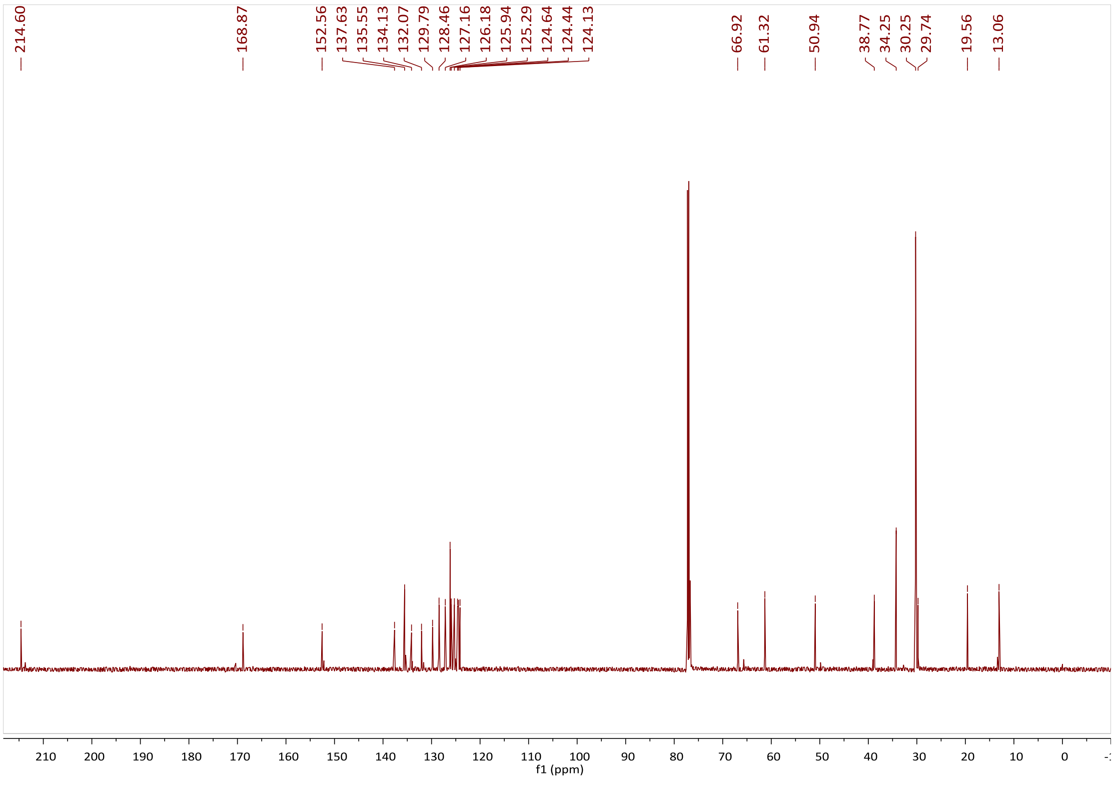
214.60
168.87
152.56
137.63
135.55
134.13
132.07
129.79
128.46
127.16
126.18
125.94
125.29
124.64
124.44
124.13
66.92
61.32
50.94
38.77
34.25
30.25
29.74
19.56
13.06
f1 (ppm)

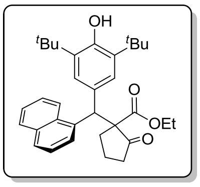

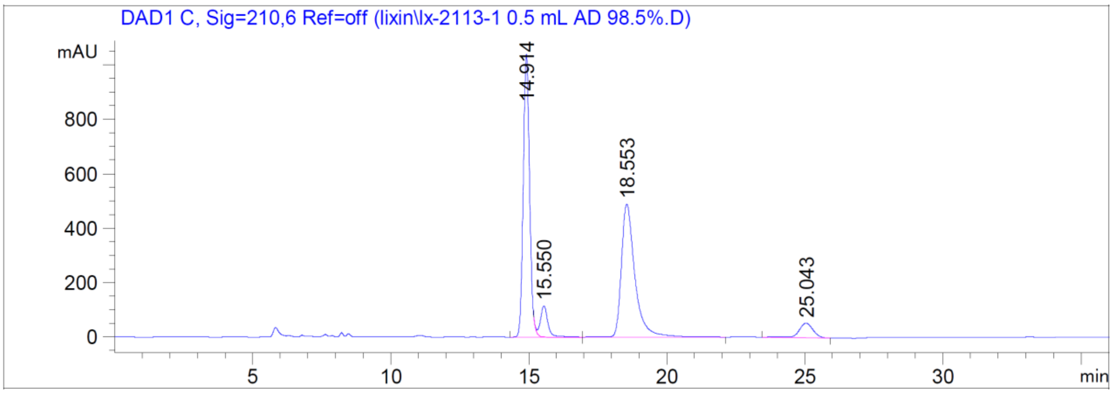

Peak #	RetTime [min]	Type	Width [min]	Area [mAU*s]	Height [mAU]	Area %
1	14.914	BV R	0.2505	1.65463e4	1035.07825	44.3666
2	15.550	VB E	0.2921	2152.65112	114.08670	5.7720
3	18.553	VV R	0.5083	1.66818e4	489.20352	44.7298
4	25.043	VB R	0.5305	1913.78882	54.03866	5.1316

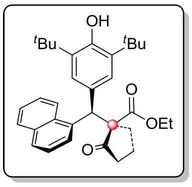

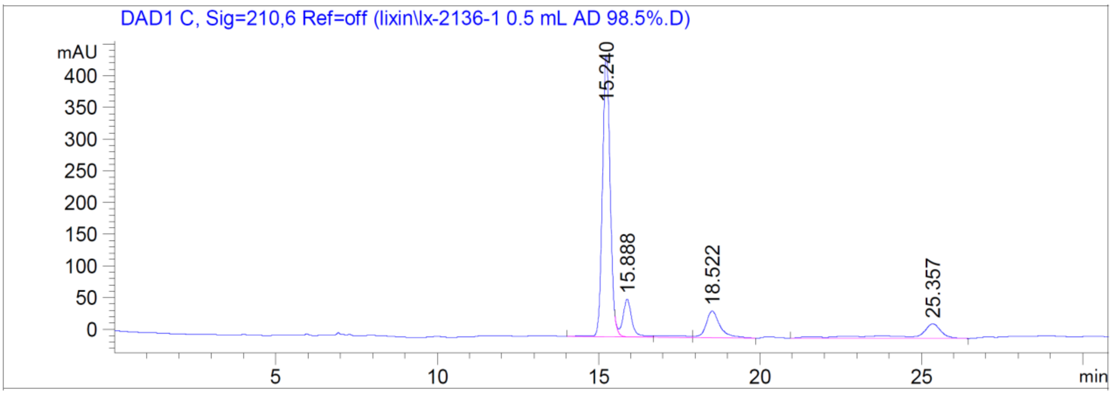

Peak #	RetTime [min]	Type	Width [min]	Area [mAU*s]	Height [mAU]	Area %
1	15.240	BV R	0.2584	7557.73193	443.05884	66.9057
2	15.888	VV E	0.2865	1129.58740	59.22829	9.9998
3	18.522	VB	0.4380	1262.67615	42.09131	11.1780
4	25.357	VB R	0.5325	1346.09680	22.98726	11.9165

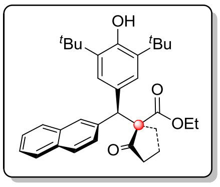
OH
tBu
tBu
O
OEt
O

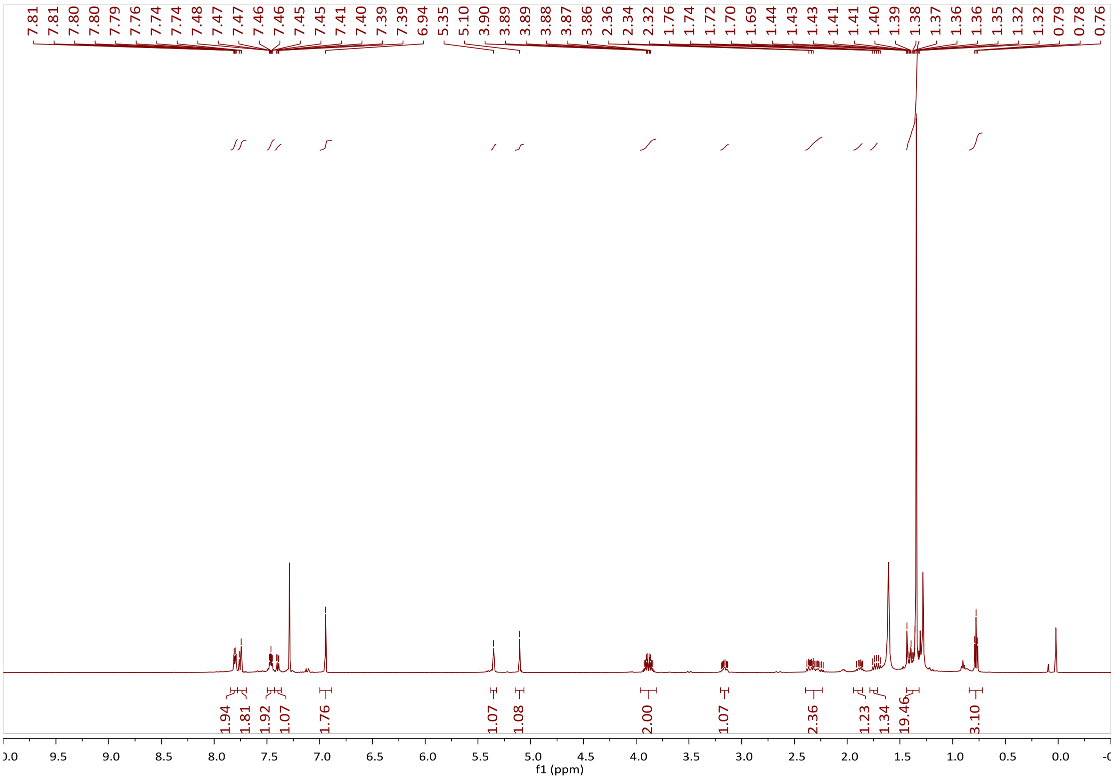

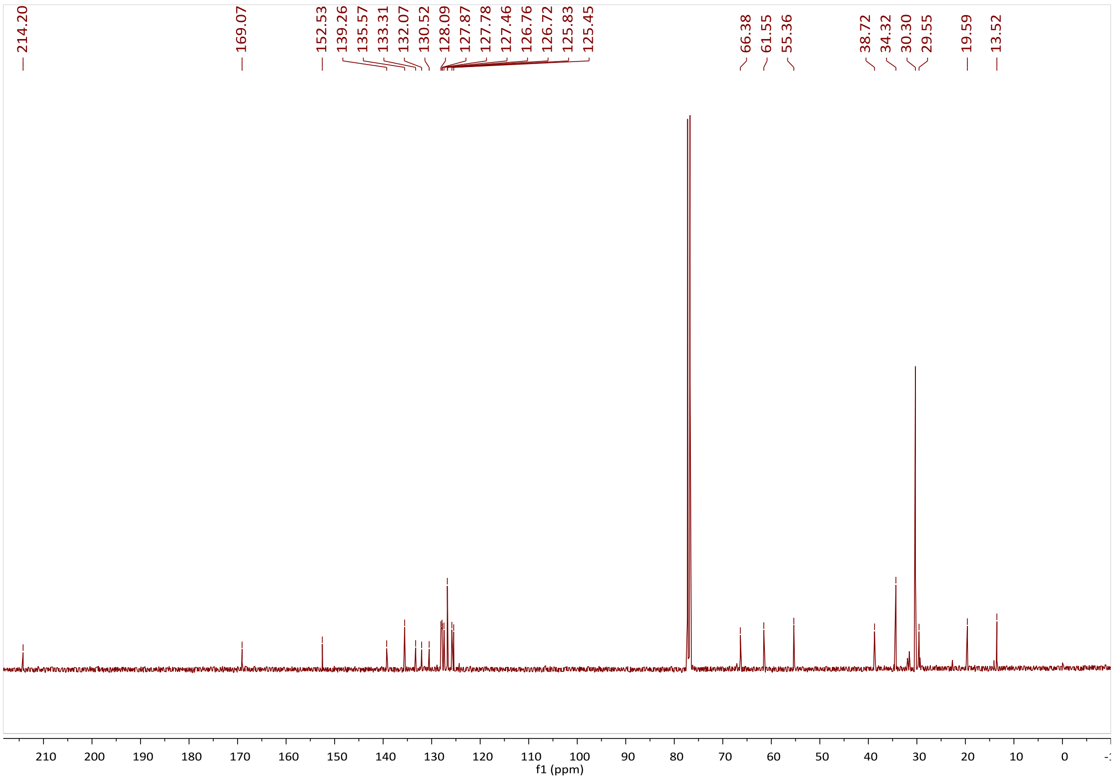

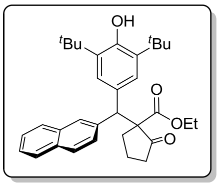

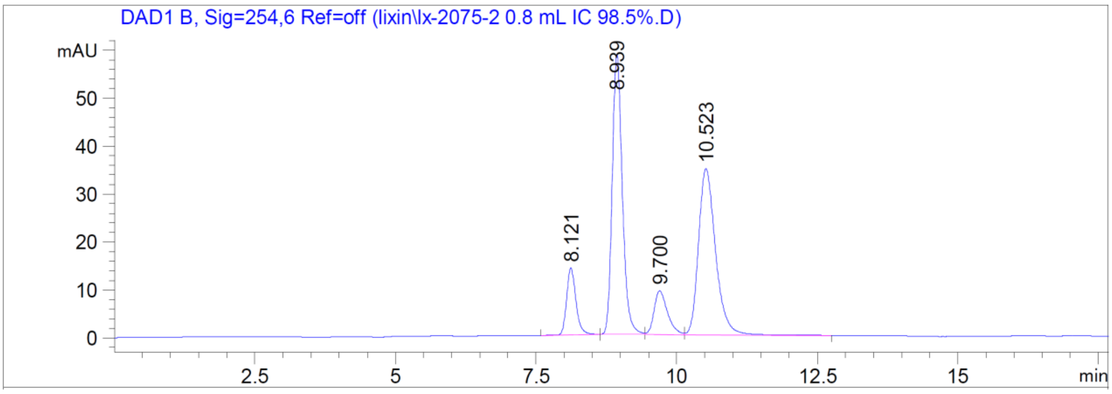

Peak #	RetTime [min]	Type	Width [min]	Area [mAU*s]	Height [mAU]	Area %
1	8.121	BB	0.1761	160.39052	13.95206	9.0589
2	8.939	BV	0.1917	729.77838	58.42525	41.2179
3	9.700	VV	0.2531	151.05519	9.12661	8.5316
4	10.523	VB	0.3216	729.31531	34.64781	41.1917

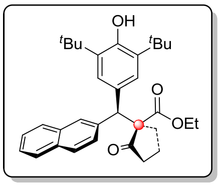

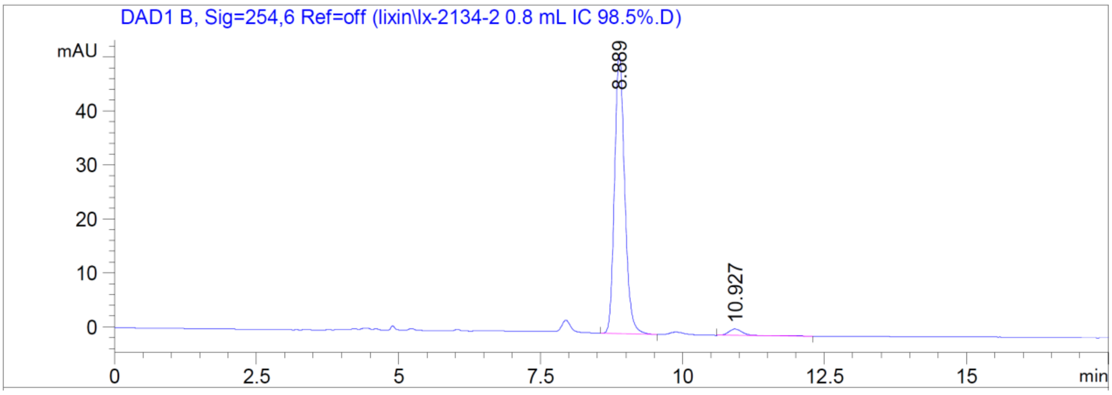

Peak #	RetTime [min]	Type	Width [min]	Area [mAU*s]	Height [mAU]	Area %
1	8.889	BB	0.1912	645.24048	51.83414	96.8214
2	10.927	BB	0.2630	21.18292	1.19403	3.1786

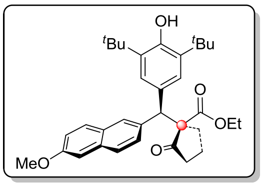

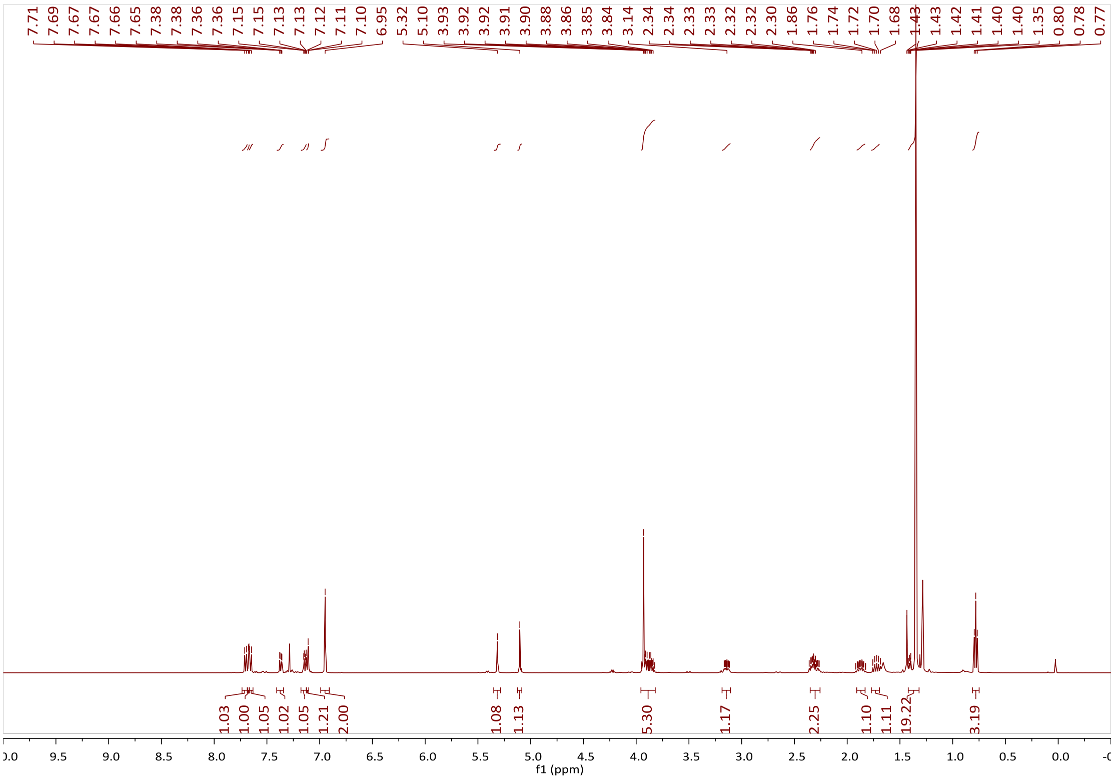

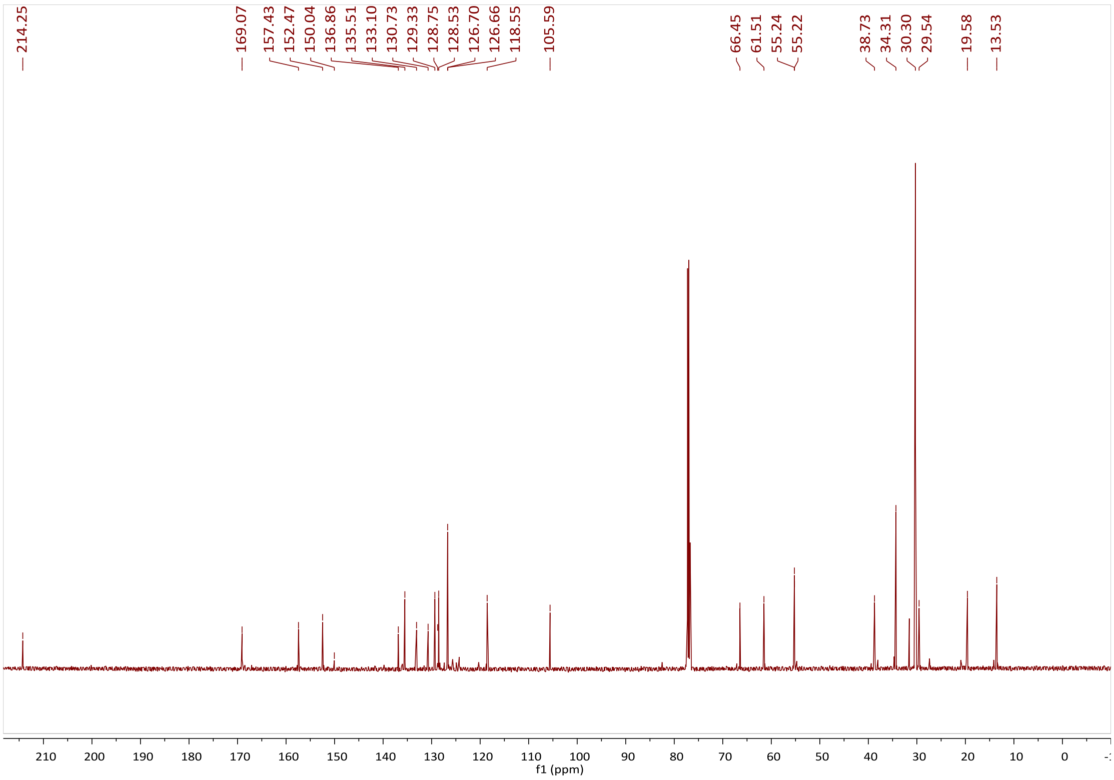

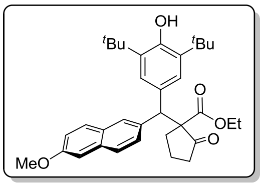

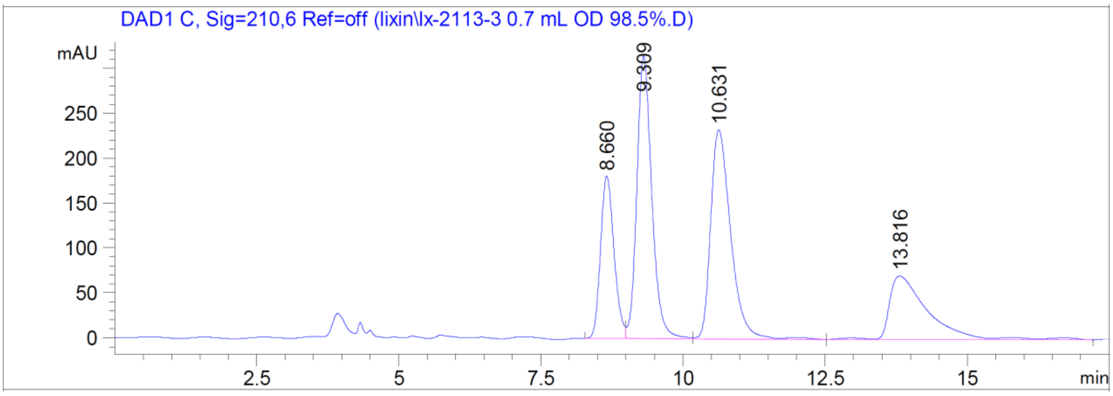

Peak #	RetTime [min]	Type	Width [min]	Area [mAU*s]	Height [mAU]	Area %
1	8.660	BV	0.2574	2988.45410	180.30756	16.7856
2	9.309	VV	0.2773	5732.22217	313.51987	32.1968
3	10.631	VV R	0.3798	5712.43652	232.61742	32.0856
4	13.816	VV R	0.6577	3370.60596	70.75017	18.9320

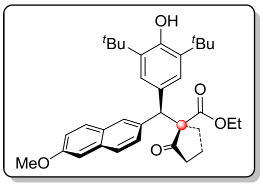

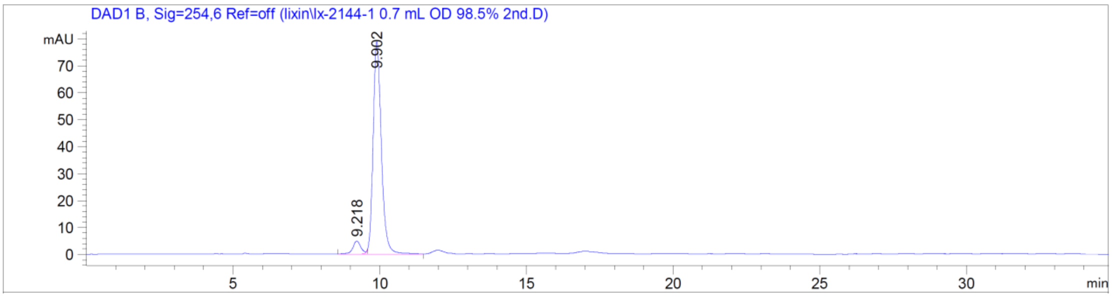

Peak #	RetTime [min]	Type	Width [min]	Area [mAU*s]	Height [mAU]	Area %
1	9.218	BV E	0.2823	89.31616	4.77399	5.4920
2	9.902	VB R	0.2975	1536.98804	78.81986	94.5080

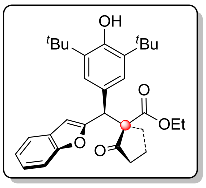
OH
tBu
tBu
O
OEt
O
O

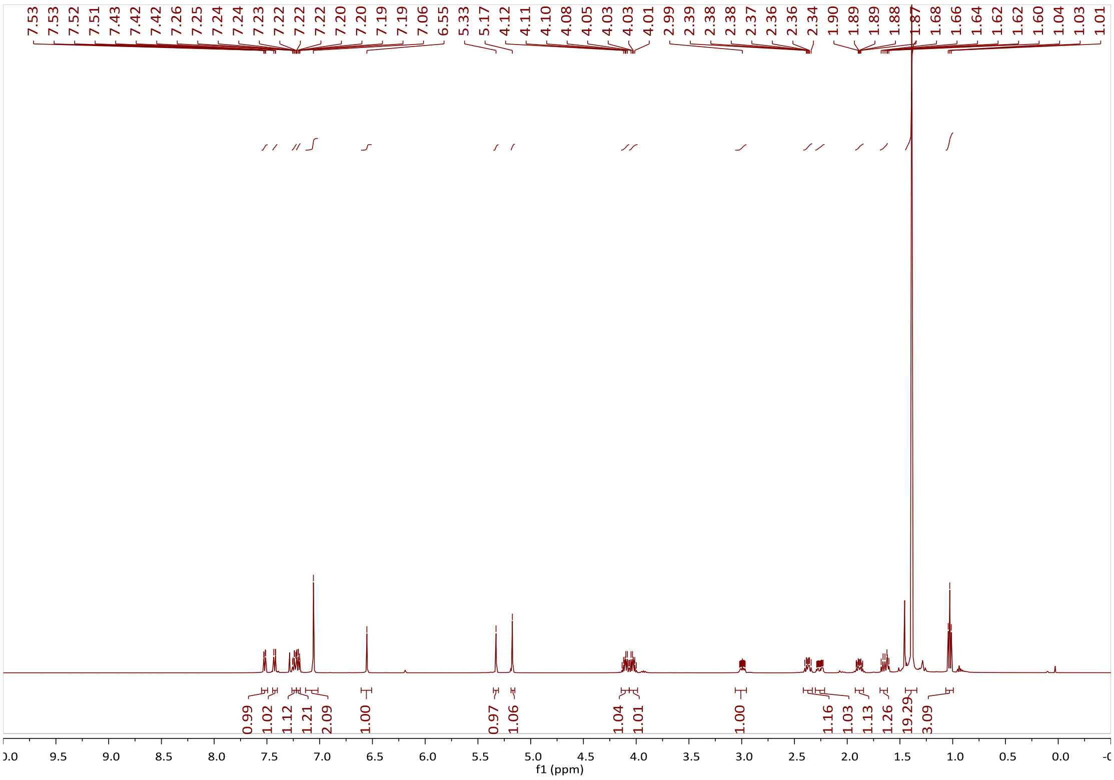
7.53 7.53 7.52 7.51 7.43 7.42 7.42 7.26 7.25 7.24 7.24 7.23 7.22 7.22 7.22 7.20 7.19 7.19 7.06 6.55 5.33 5.17 4.12 4.11 4.10 4.08 4.05 4.03 4.03 4.01 2.99 2.39 2.38 2.38 2.37 2.36 2.36 2.34 1.90 1.89 1.89 1.88 1.87 1.68 1.66 1.64 1.62 1.62 1.60 1.04 1.03 1.01
0.99 1.02 1.12 1.21 2.09 1.00 0.97 1.06 1.04 1.01 1.00 1.16 1.03 1.13 1.26 19.29 3.09
9.5 9.0 8.5 8.0 7.5 7.0 6.5 6.0 5.5 5.0 4.5 4.0 3.5 3.0 2.5 2.0 1.5 1.0 0.5 0.0
f1 (ppm)

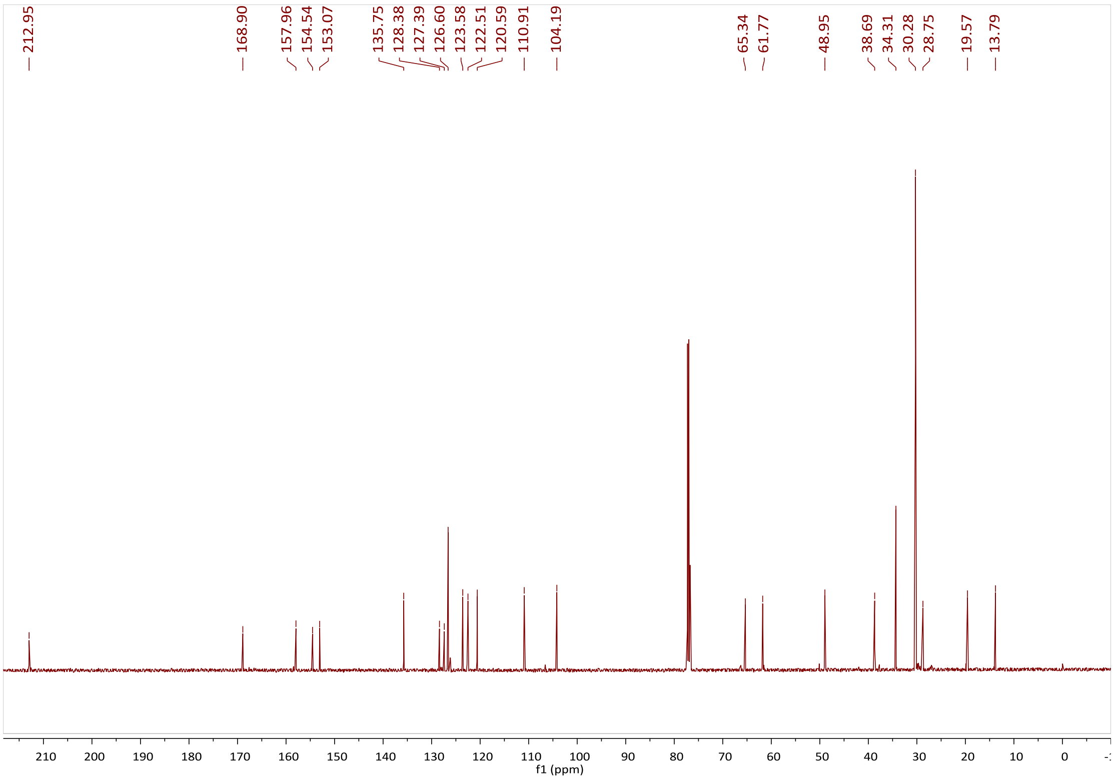
212.95
168.90
157.96 154.54 153.07
135.75 128.38 127.39 126.60 123.58 122.51 120.59 110.91 104.19
65.34 61.77
48.95
38.69 34.31 30.28 28.75
19.57 13.79
210 200 190 180 170 160 150 140 130 120 110 100 90 80 70 60 50 40 30 20 10 0
f1 (ppm)

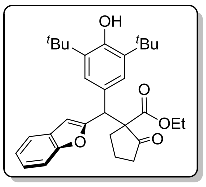

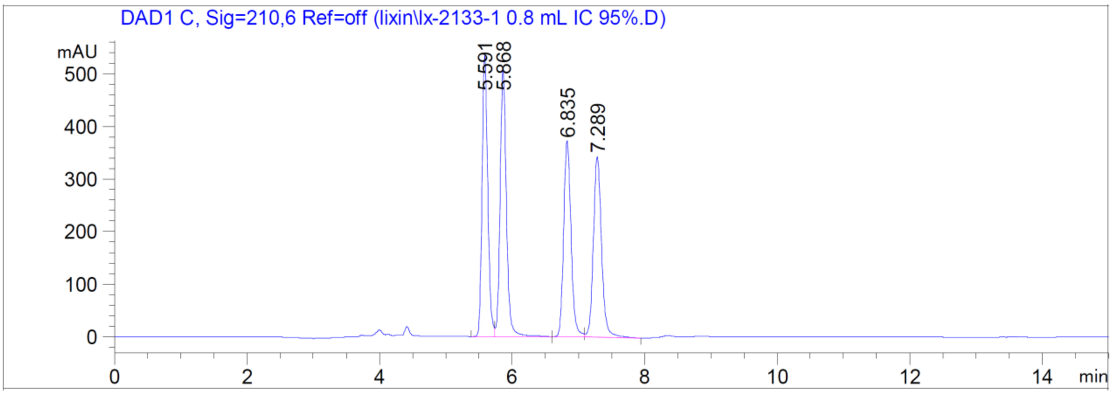

Peak #	RetTime [min]	Type	Width [min]	Area [mAU*s]	Height [mAU]	Area %
1	5.591	BV	0.0946	3180.60132	525.37842	25.6457
2	5.868	VB	0.1115	3357.73999	492.80194	27.0740
3	6.835	BV	0.1234	2882.17505	367.99380	23.2394
4	7.289	VB	0.1424	2981.57715	339.80167	24.0409

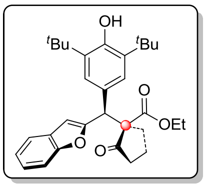

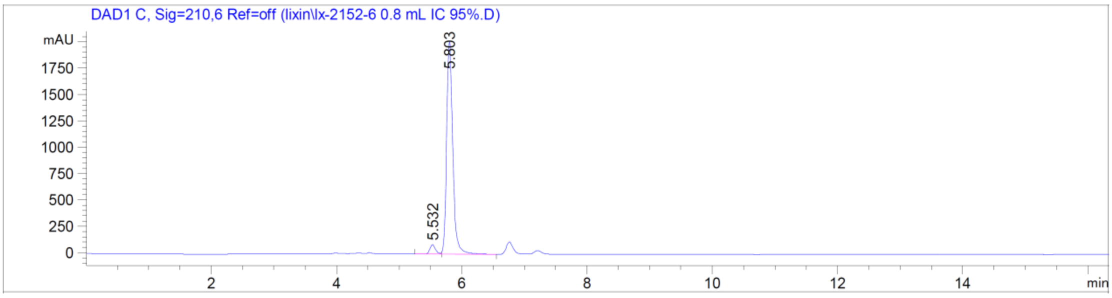

Peak #	RetTime [min]	Type	Width [min]	Area [mAU*s]	Height [mAU]	Area %
1	5.532	BV E	0.0943	557.17432	89.93005	3.8855
2	5.803	VB R	0.1037	1.37827e4	2016.53650	96.1145

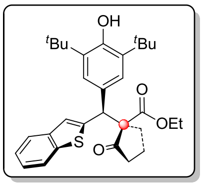
OH
tBu
tBu
O
OEt
S
O

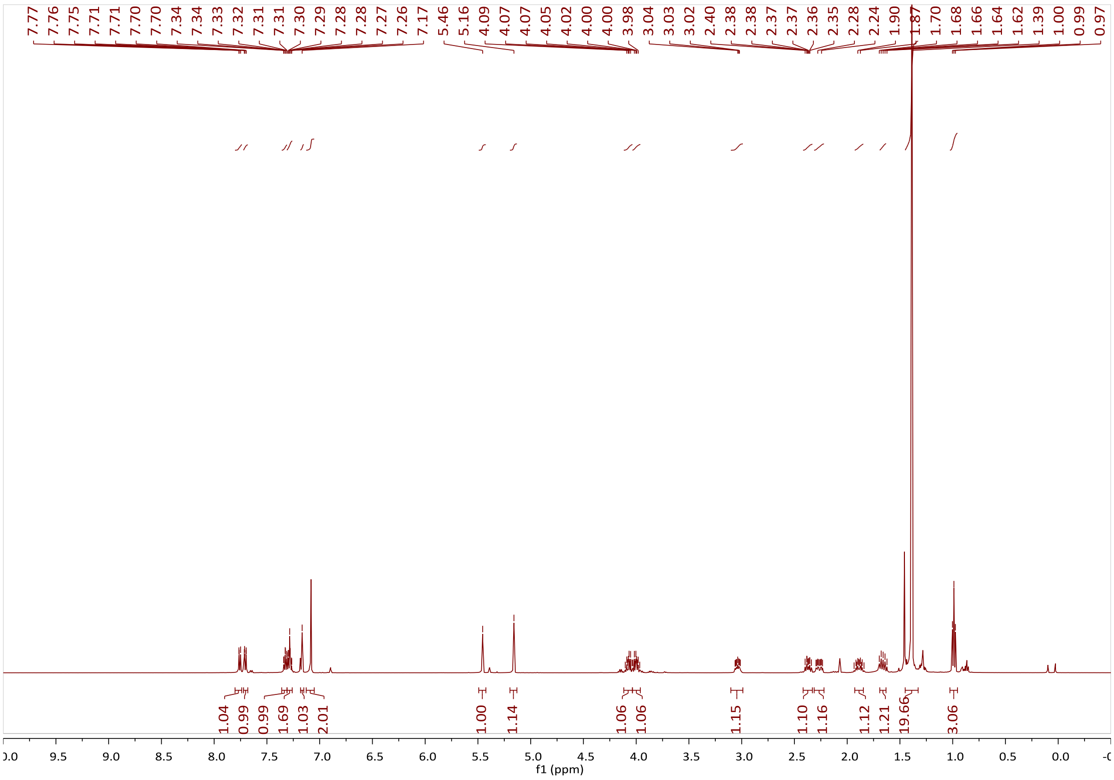
7.77
7.76
7.75
7.71
7.71
7.70
7.70
7.34
7.34
7.33
7.32
7.31
7.31
7.30
7.29
7.28
7.28
7.27
7.26
7.17
5.46
5.16
4.09
4.07
4.07
4.05
4.02
4.00
4.00
3.98
3.04
3.02
3.02
2.40
2.38
2.38
2.37
2.37
2.36
2.35
2.28
2.24
1.90
1.87
1.70
1.68
1.66
1.64
1.62
1.39
1.00
0.99
0.97
1.04
0.99
0.99
1.69
1.03
2.01
1.00
1.14
1.06
1.06
1.15
1.10
1.16
1.12
1.21
19.66
3.06
f1 (ppm)

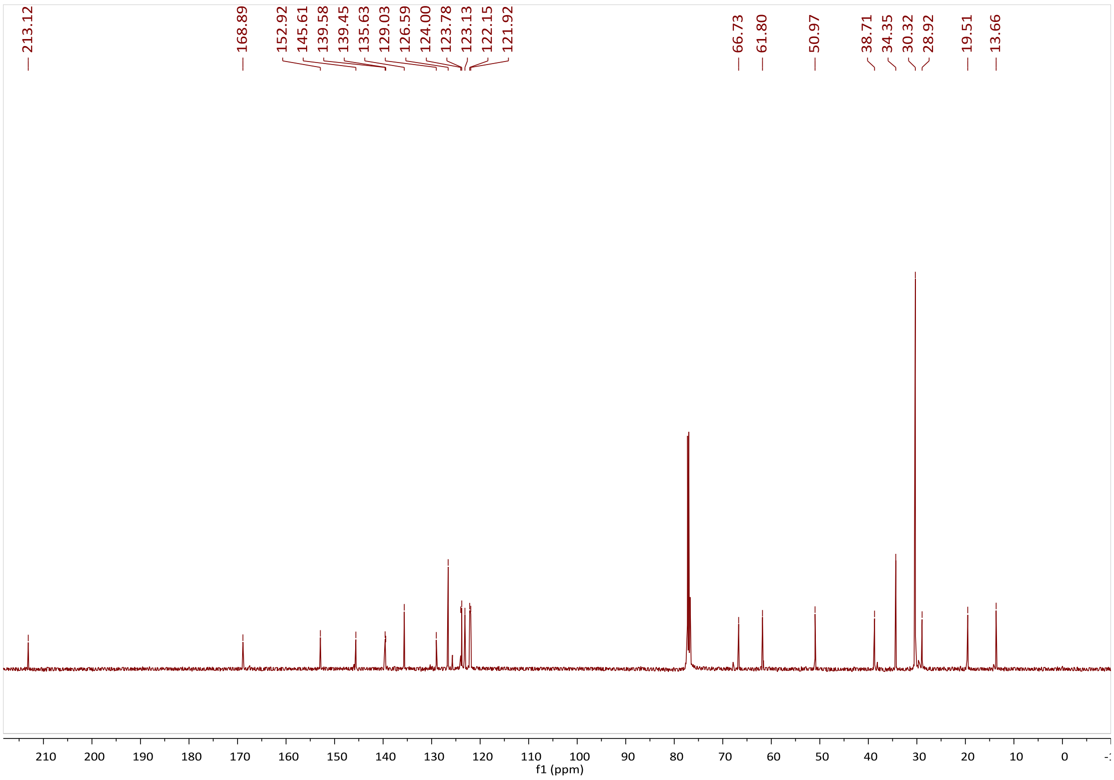
213.12
168.89
152.92
145.61
139.58
139.45
135.63
129.03
126.59
124.00
123.78
123.13
122.15
121.92
66.73
61.80
50.97
38.71
34.35
30.32
28.92
19.51
13.66
f1 (ppm)

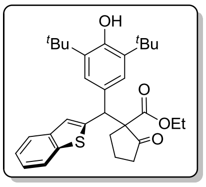

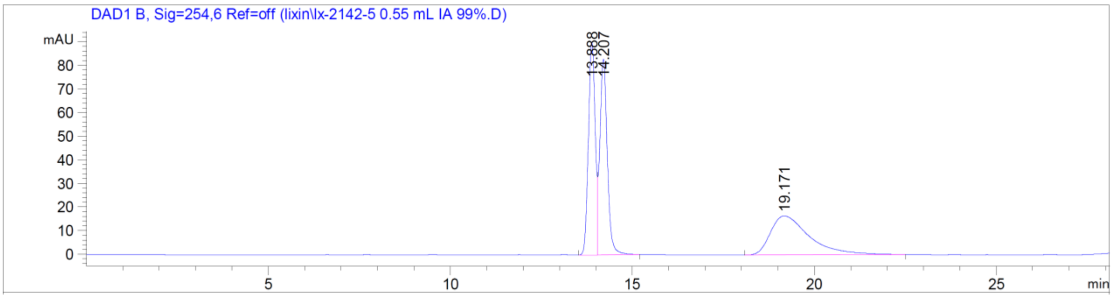

Peak #	RetTime [min]	Type	Width [min]	Area [mAU*s]	Height [mAU]	Area %
1	13.888	BV	0.2016	1173.12183	90.27656	32.8581
2	14.207	VB	0.2131	1173.07434	82.90812	32.8567
3	19.171	BB	1.0287	1224.07397	16.55349	34.2852

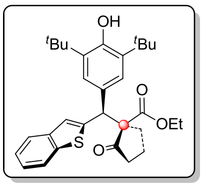

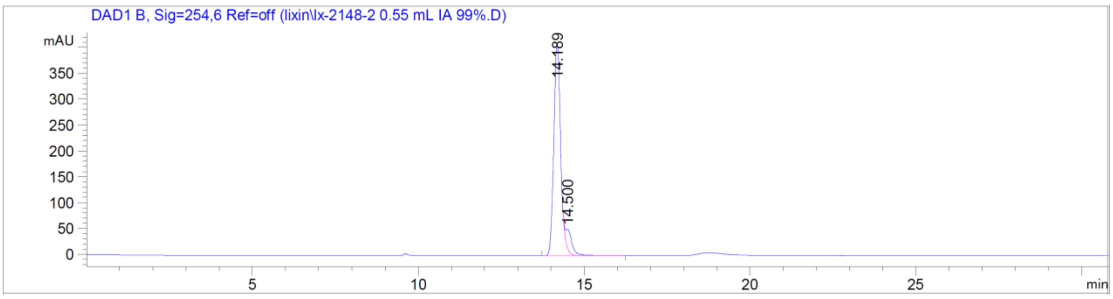

Peak #	RetTime [min]	Type	Width [min]	Area [mAU*s]	Height [mAU]	Area %
1	14.189	BV R	0.2224	5941.44482	411.58832	91.0960
2	14.500	VB E	0.2231	580.73248	36.99688	8.9040

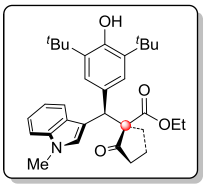
OH
tBu
tBu
O
OEt
N
Me
O

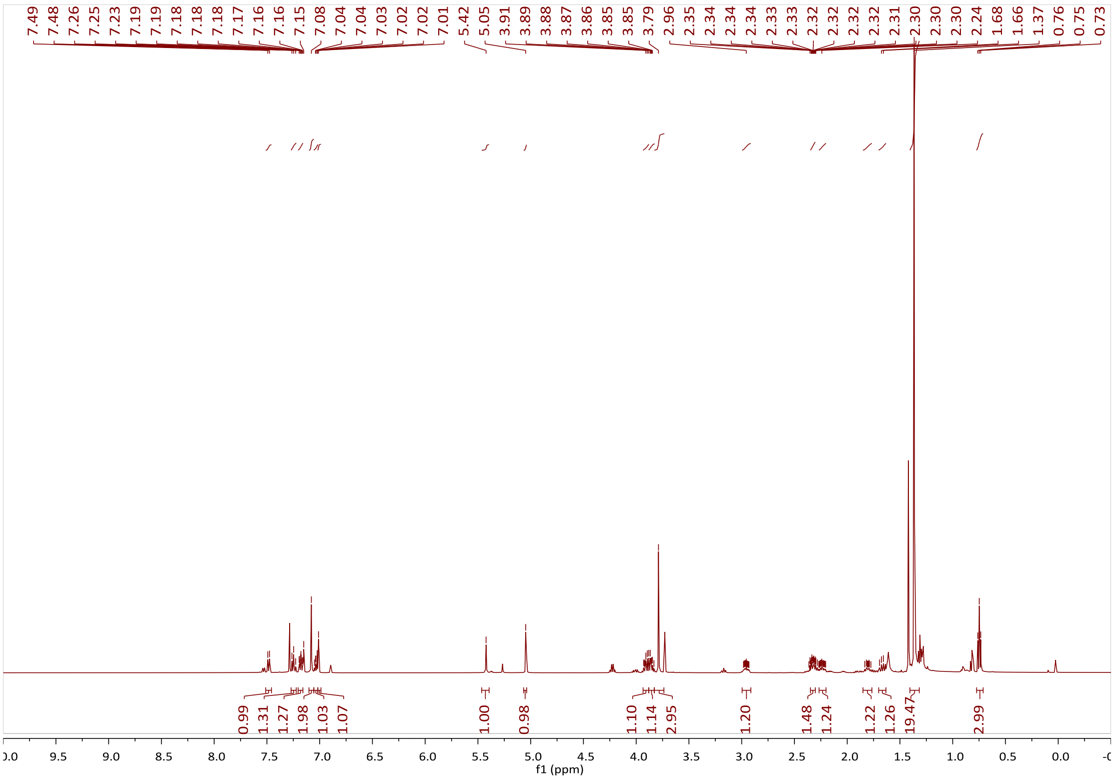
7.49
7.48
7.26
7.25
7.23
7.19
7.19
7.18
7.18
7.17
7.16
7.16
7.15
7.08
7.04
7.03
7.02
7.02
7.01
5.42
5.05
3.91
3.89
3.88
3.87
3.86
3.85
3.79
2.96
2.35
2.34
2.34
2.34
2.33
2.33
2.32
2.32
2.32
2.32
2.31
2.30
2.30
2.30
2.24
1.68
1.66
1.37
0.76
0.75
0.73
0.99
1.31
1.27
1.98
1.03
1.07
1.00
0.98
1.10
1.14
2.95
1.20
1.48
1.24
1.22
1.26
19.47
2.99
f1 (ppm)

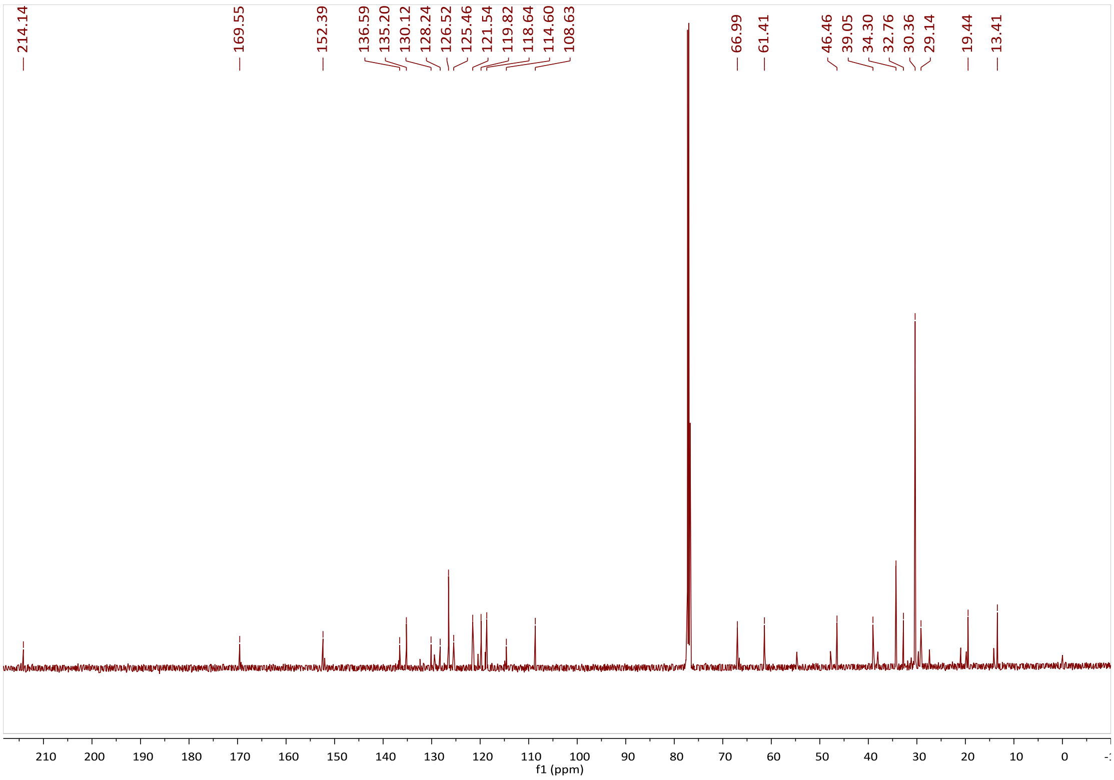
214.14
169.55
152.39
136.59
135.20
130.12
128.24
126.52
125.46
121.54
119.82
118.64
114.60
108.63
66.99
61.41
46.46
39.05
34.30
32.76
30.36
29.14
19.44
13.41
f1 (ppm)

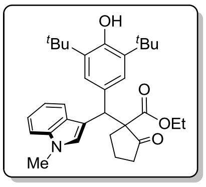

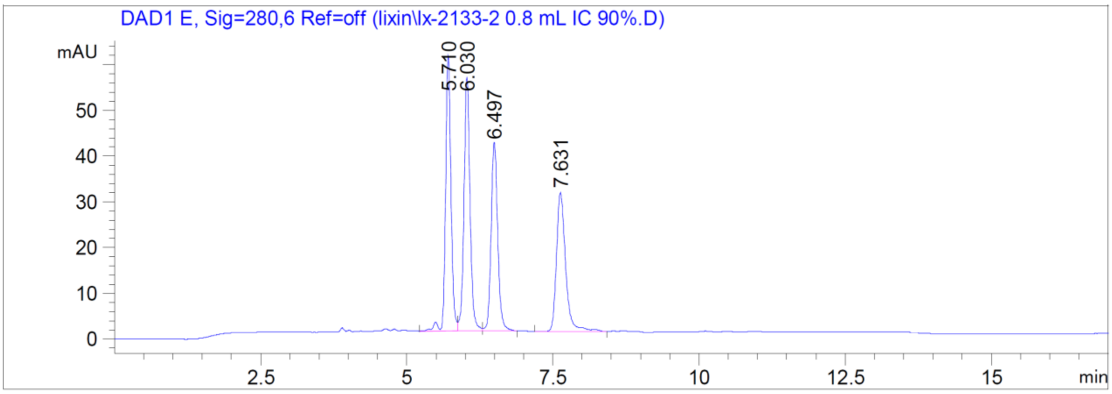

Peak #	RetTime [min]	Type	Width [min]	Area [mAU*s]	Height [mAU]	Area %
1	5.710	BV	0.1103	392.70038	58.50858	27.0667
2	6.030	VV	0.1181	401.38361	54.36011	27.6652
3	6.497	VB	0.1247	323.41547	40.68876	22.2912
4	7.631	BB	0.1692	333.36362	30.09654	22.9769

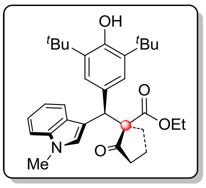

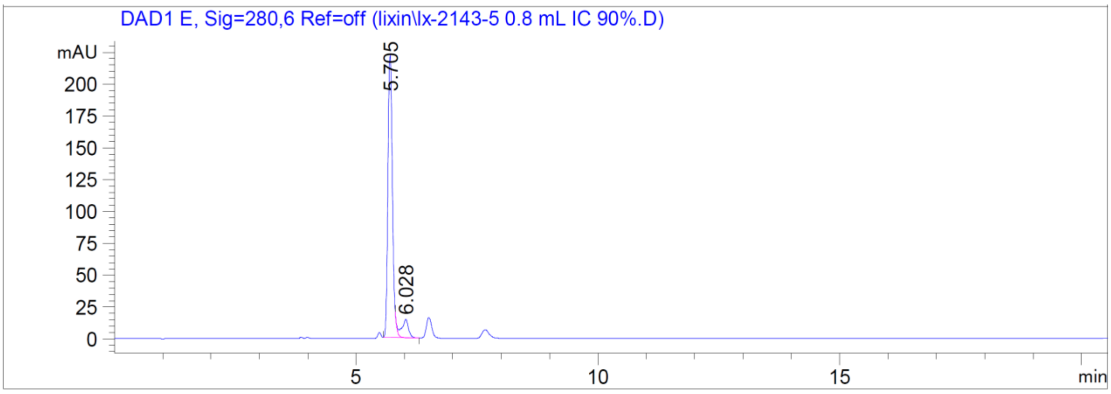

Peak #	RetTime [min]	Type	Width [min]	Area [mAU*s]	Height [mAU]	Area %
1	5.705	BV R	0.1086	1423.55835	216.93916	91.0470
2	6.028	VB E	0.1482	139.98412	14.05317	8.9530

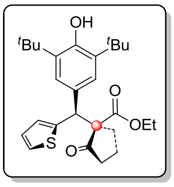
OH
tBu
tBu
O
OEt
S
O

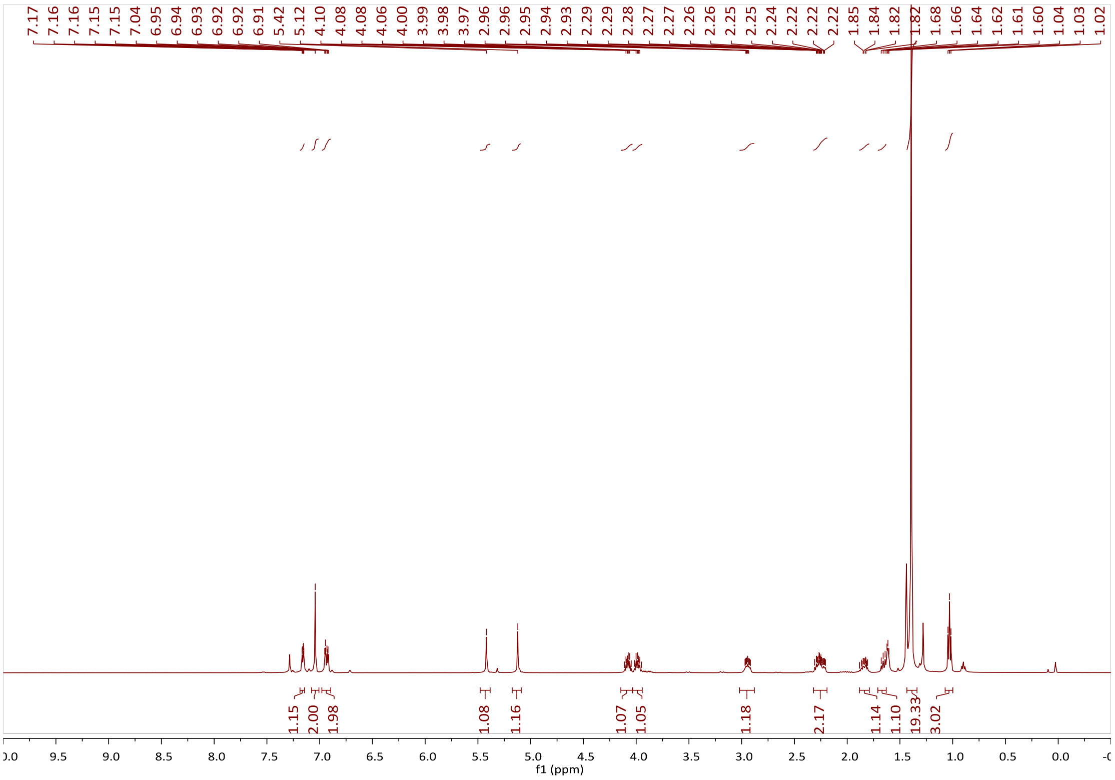

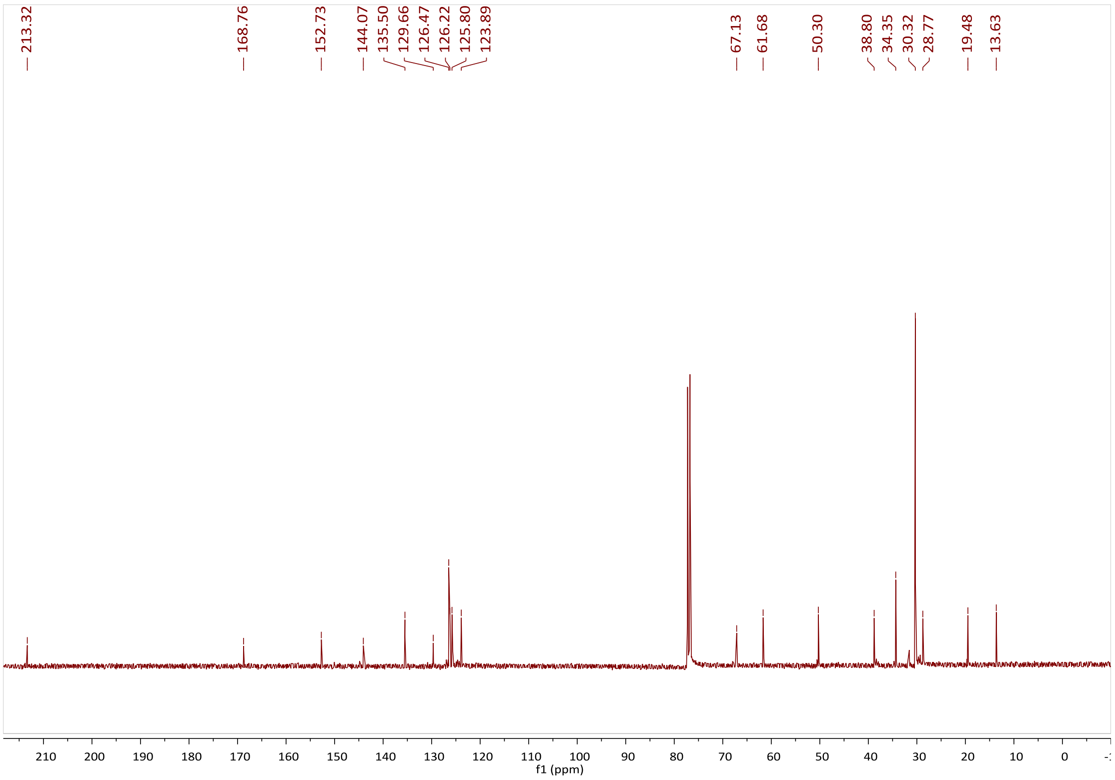

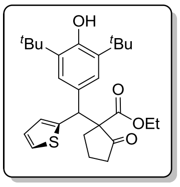

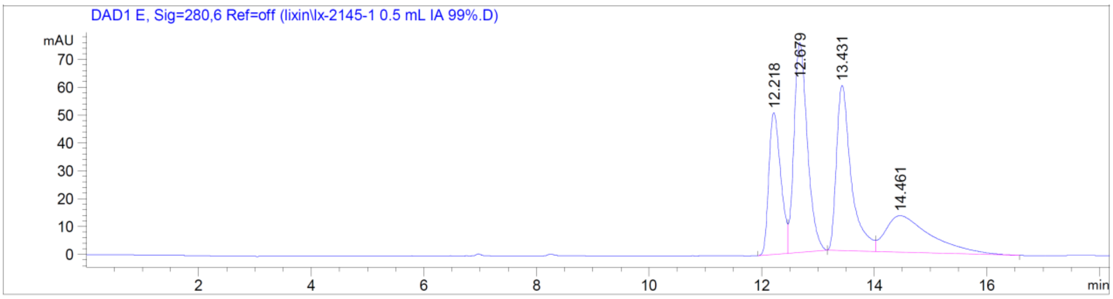

Peak #	RetTime [min]	Type	Width [min]	Area [mAU*s]	Height [mAU]	Area %
1	12.218	BV	0.2275	747.78461	50.85527	19.6821
2	12.679	VB	0.2547	1240.13037	75.08587	32.6409
3	13.431	BV	0.2721	1077.95630	59.28917	28.3724
4	14.461	VB	0.7913	733.44080	13.05751	19.3046

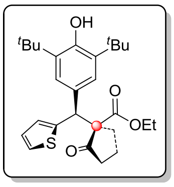

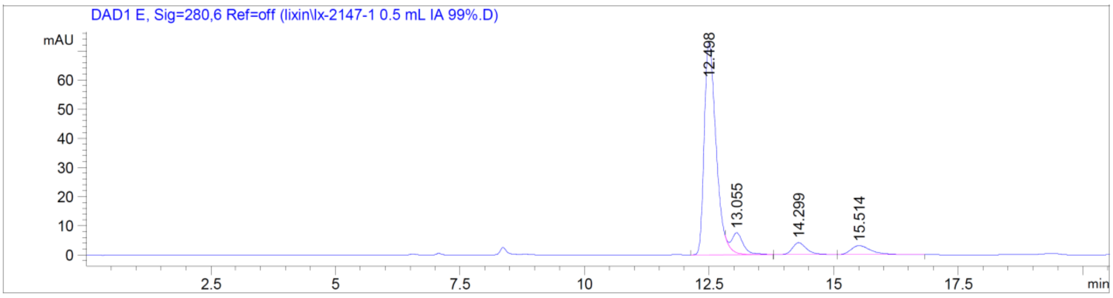

Peak #	RetTime [min]	Type	Width [min]	Area [mAU*s]	Height [mAU]	Area %
1	12.498	BV R	0.2553	1207.21985	72.86346	81.6090
2	13.055	VB E	0.2330	107.17213	6.83175	7.2449
3	14.299	BB	0.2966	80.37186	4.06591	5.4332
4	15.514	BB	0.4058	84.50947	3.06412	5.7129

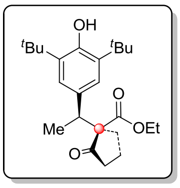
OH
tBu
tBu
Me
O
OEt
O

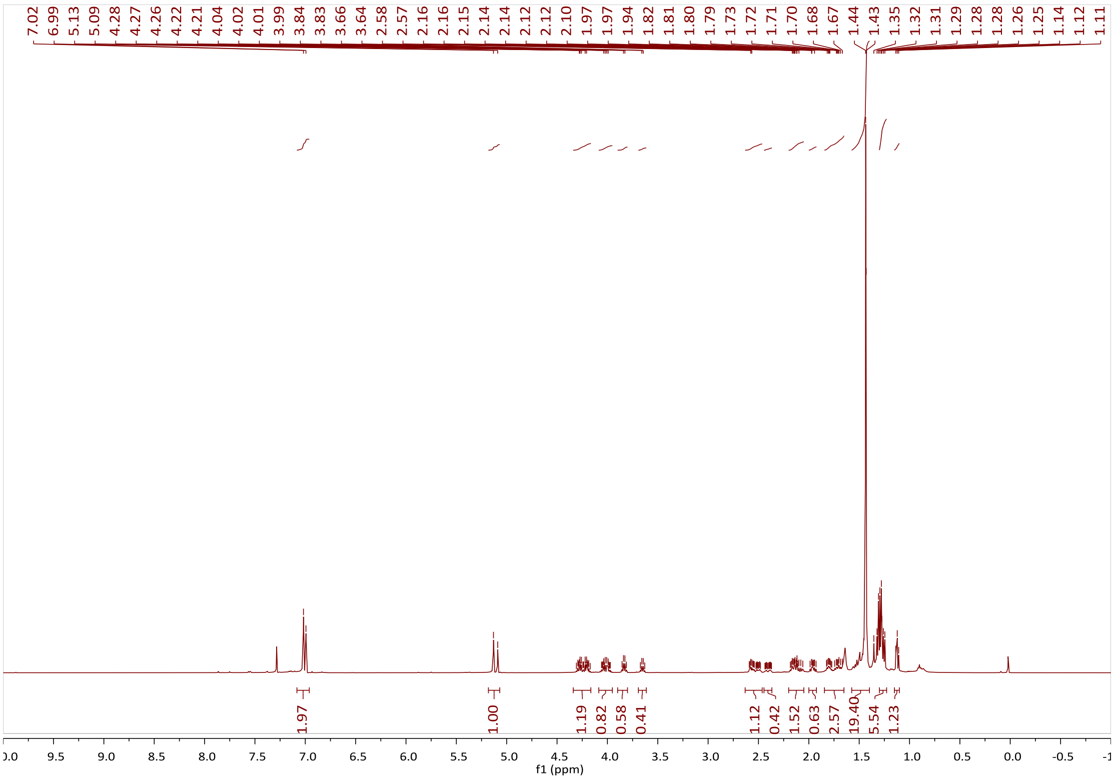
7.02 6.99 5.13 5.09 4.28 4.27 4.26 4.22 4.21 4.04 4.02 4.01 3.99 3.84 3.83 3.66 3.64 2.58 2.57 2.16 2.16 2.15 2.14 2.14 2.12 2.12 2.10 1.97 1.97 1.94 1.82 1.81 1.80 1.79 1.73 1.72 1.71 1.70 1.68 1.67 1.44 1.43 1.35 1.32 1.31 1.29 1.28 1.28 1.26 1.25 1.14 1.12 1.11
1.97 1.00 1.19 0.82 0.58 0.41 1.12 0.42 1.52 0.63 2.57 19.40 5.54 1.23
f1 (ppm)

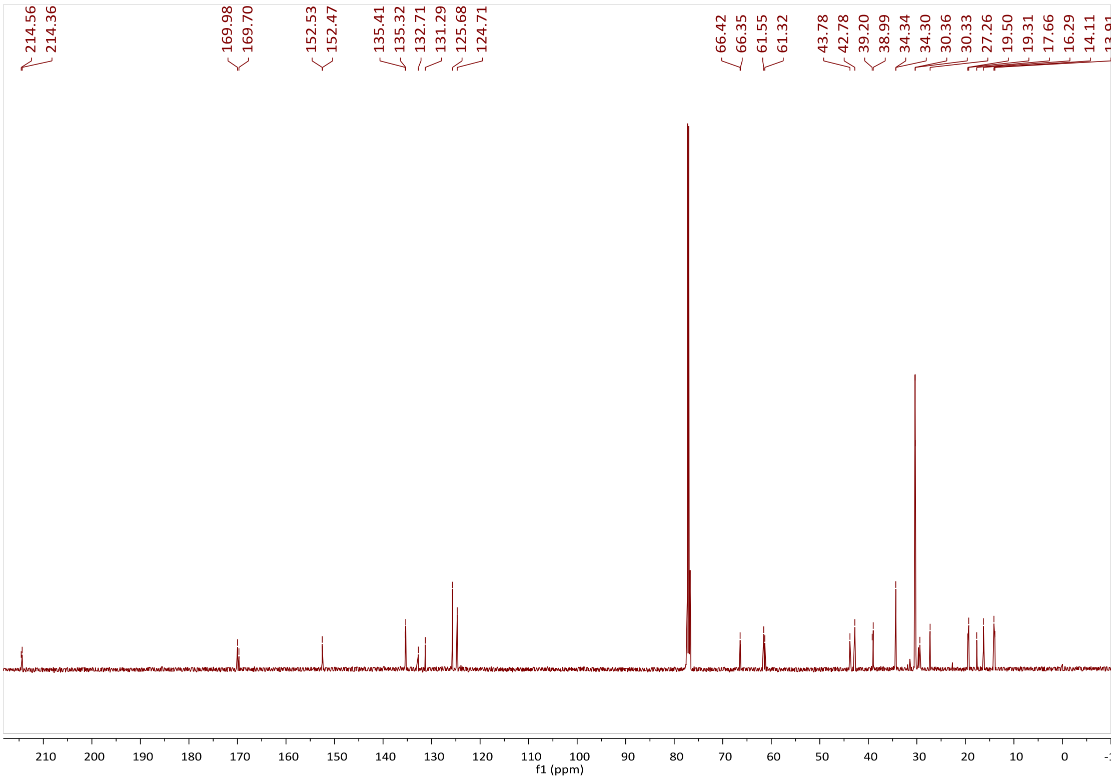
214.56 214.36 169.98 169.70 152.53 152.47 135.41 135.32 132.71 131.29 125.68 124.71 66.42 66.35 61.55 61.32 43.78 42.78 39.20 38.99 34.34 34.30 30.36 30.33 27.26 19.50 19.31 17.66 16.29 14.11
f1 (ppm)

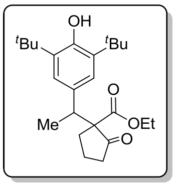

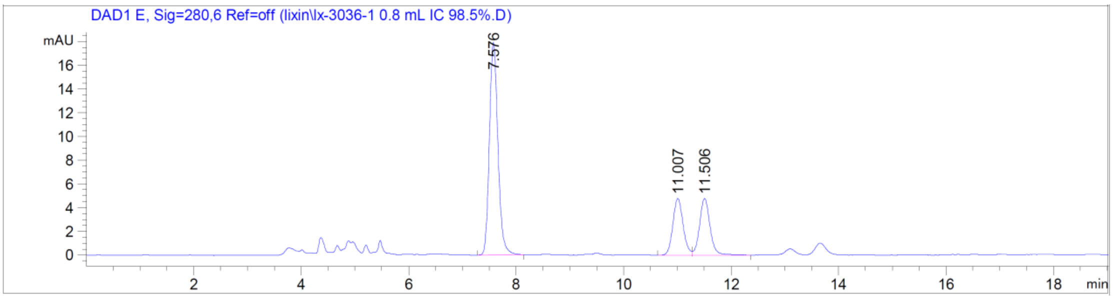

Peak #	RetTime [min]	Type	Width [min]	Area [mAU*s]	Height [mAU]	Area %
1	7.576	BB	0.1708	197.76097	17.90735	60.7900
2	11.007	BV	0.2019	62.95538	4.77298	19.3519
3	11.506	VB	0.2080	64.60209	4.77146	19.8581

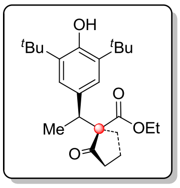

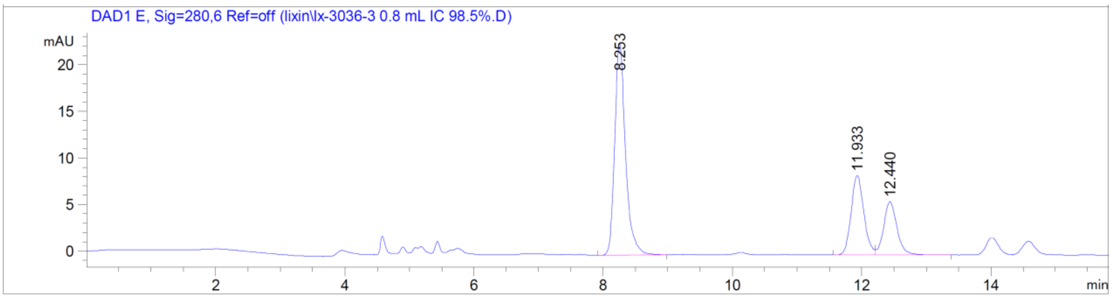

Peak #	RetTime [min]	Type	Width [min]	Area [mAU*s]	Height [mAU]	Area %
1	8.253	BB	0.1762	265.48730	22.73929	57.1869
2	11.933	BV	0.2119	116.79064	8.52051	25.1571
3	12.440	VB	0.2171	81.96734	5.72184	17.6560

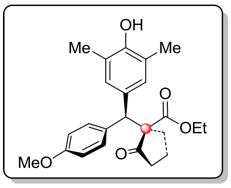
OH
Me
Me
O
OEt
MeO
O

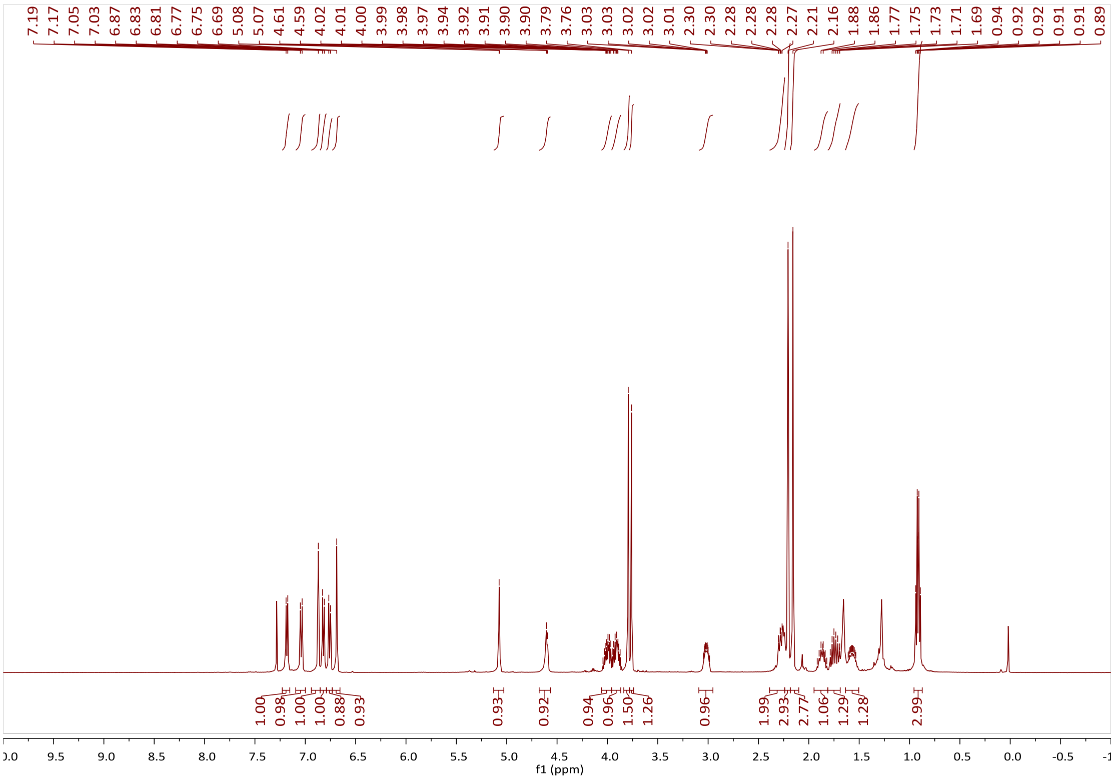
f1 (ppm)

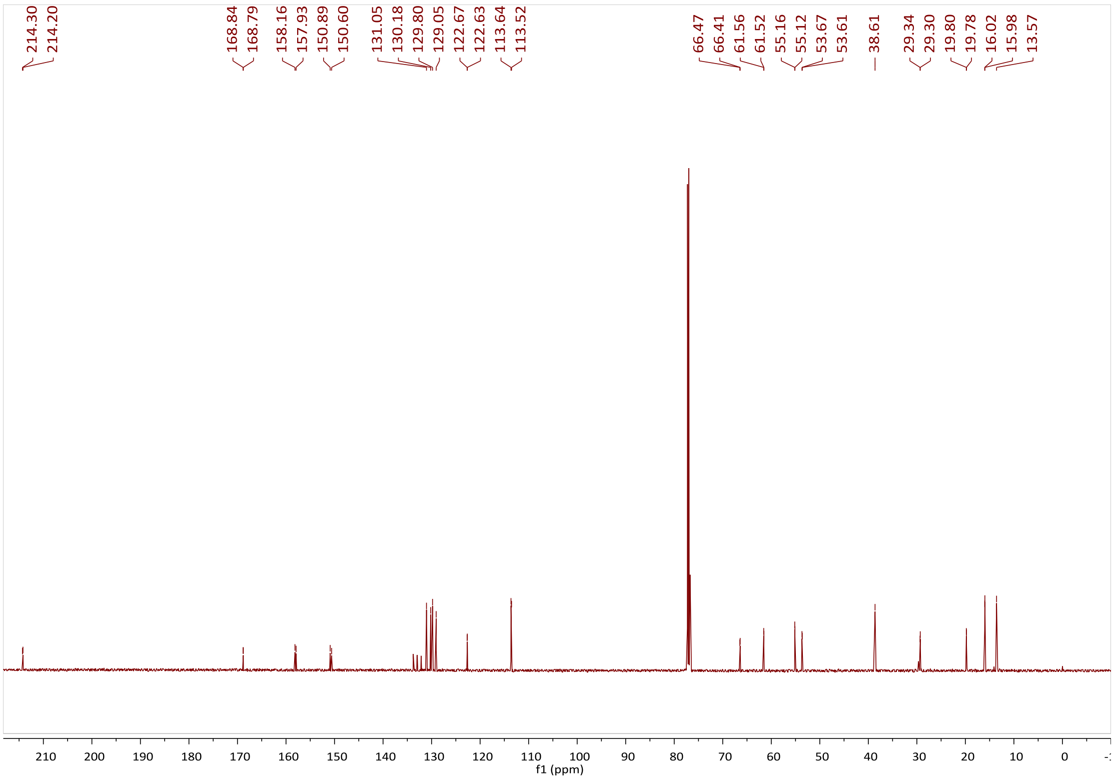
f1 (ppm)

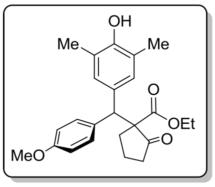

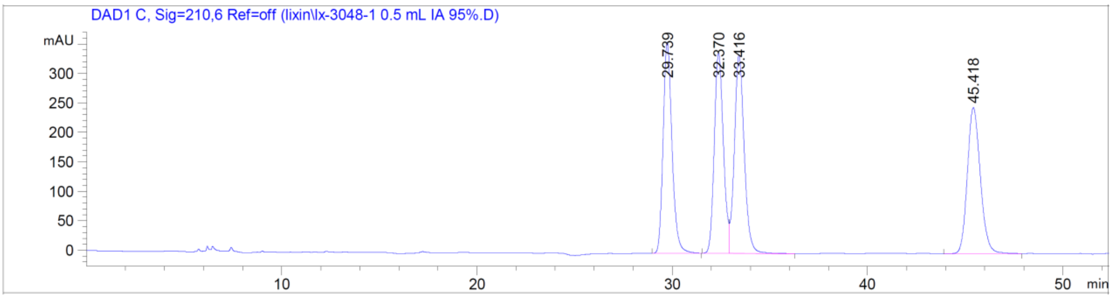

Peak #	RetTime [min]	Type	Width [min]	Area [mAU*s]	Height [mAU]	Area %
1	29.739	BB	0.4814	1.13295e4	357.84882	24.7865
2	32.370	BV	0.5017	1.10246e4	340.56436	24.1195
3	33.416	VB	0.5304	1.18184e4	337.59784	25.8560
4	45.418	BB	0.7161	1.15359e4	248.28638	25.2380

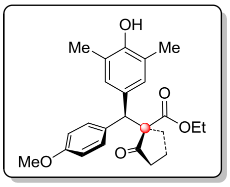

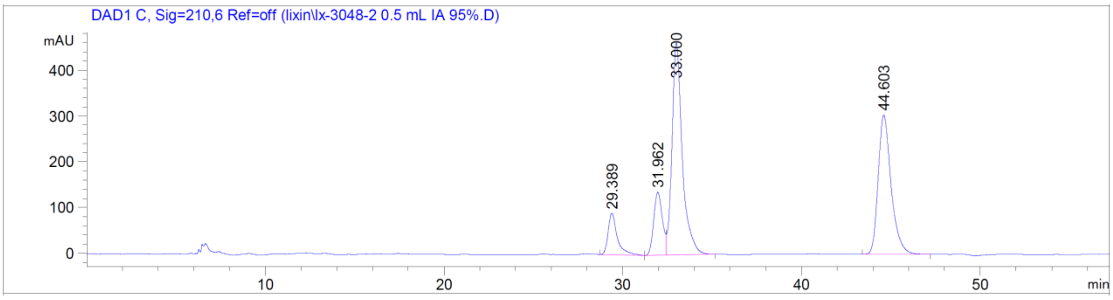

Peak #	RetTime [min]	Type	Width [min]	Area [mAU*s]	Height [mAU]	Area %
1	29.389	BB	0.5295	3244.38379	90.66420	7.7638
2	31.962	BV	0.5200	4661.09570	137.30804	11.1540
3	33.000	VB	0.5985	1.85899e4	462.97427	44.4857
4	44.603	BB	0.7610	1.52931e4	303.95435	36.5965

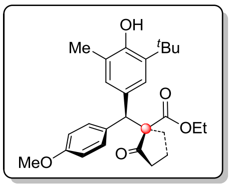
OH
Me
tBu
O
OEt
O
MeO

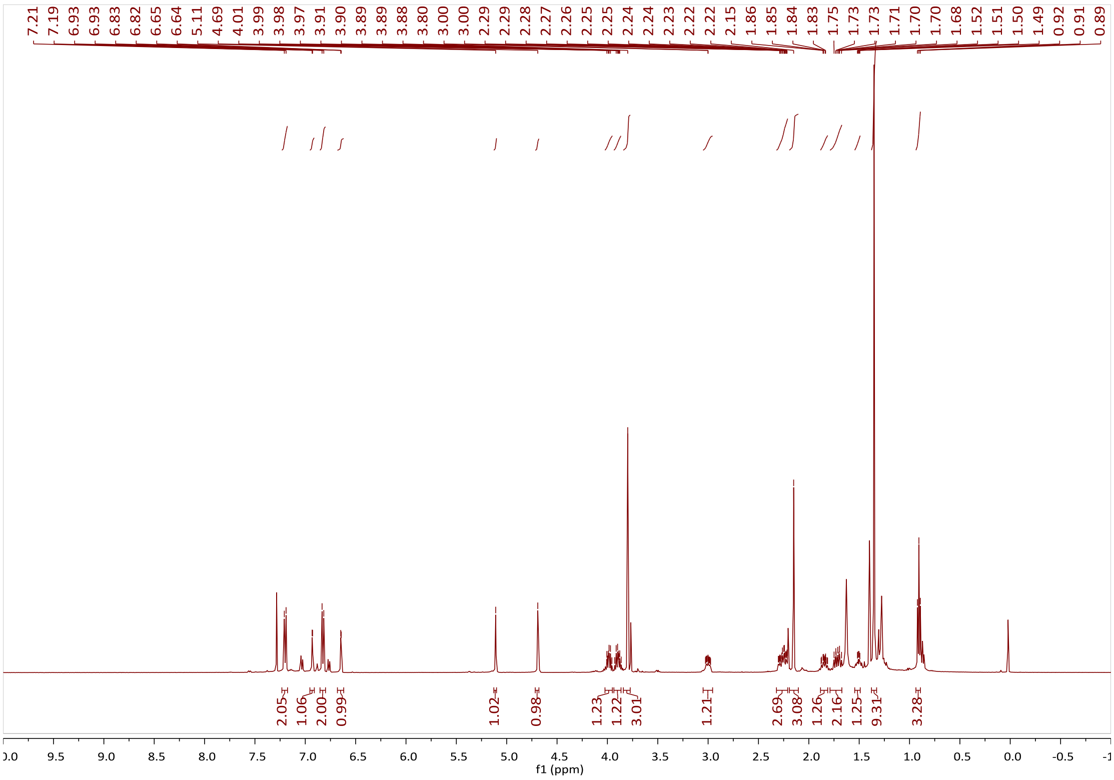
7.21
7.19
6.93
6.93
6.83
6.82
6.65
6.64
5.11
4.69
4.01
3.99
3.98
3.97
3.91
3.90
3.89
3.89
3.88
3.80
3.00
3.00
2.29
2.29
2.28
2.27
2.26
2.25
2.25
2.24
2.24
2.23
2.22
2.22
2.15
1.86
1.85
1.84
1.83
1.75
1.73
1.73
1.71
1.70
1.70
1.68
1.52
1.51
1.50
1.49
0.92
0.91
0.89
2.05
1.06
2.00
0.99
1.02
0.98
1.23
1.22
3.01
1.21
2.69
3.08
1.26
2.16
1.25
9.31
3.28
f1 (ppm)

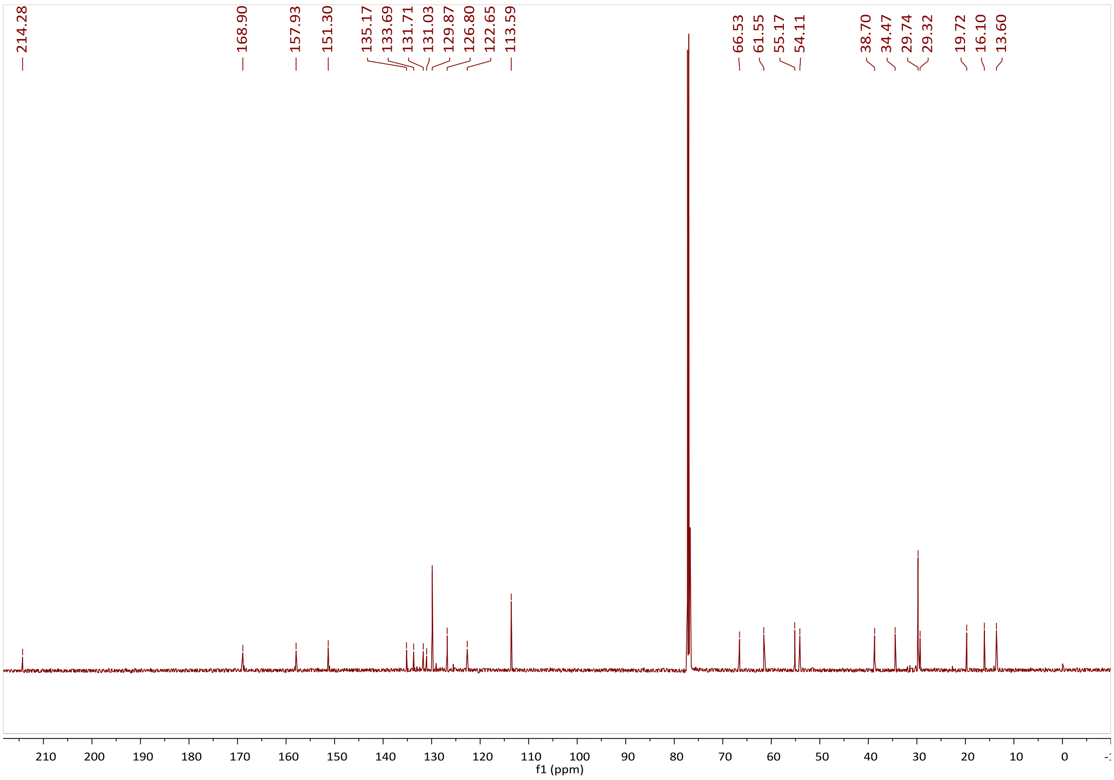
214.28
168.90
157.93
151.30
135.17
133.69
131.71
131.03
129.87
126.80
122.65
113.59
66.53
61.55
55.17
54.11
38.70
34.47
29.74
29.32
19.72
16.10
13.60
f1 (ppm)

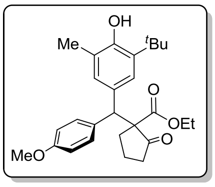

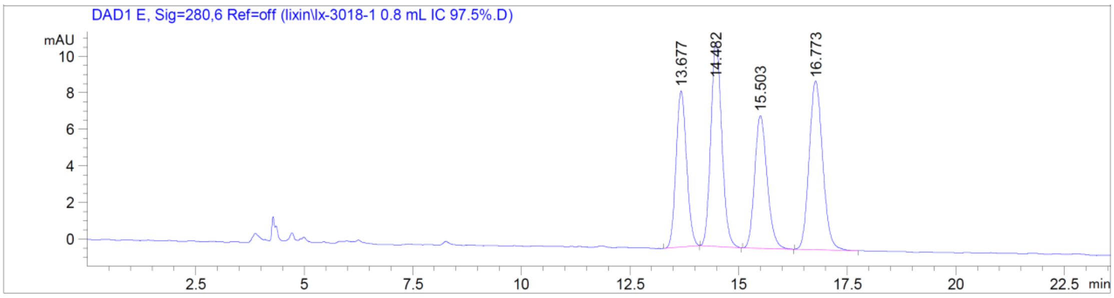

Peak #	RetTime [min]	Type	Width [min]	Area [mAU*s]	Height [mAU]	Area %
1	13.677	BB	0.2604	143.78377	8.54199	20.9614
2	14.482	BB	0.2733	196.58699	11.17237	28.6592
3	15.503	BB	0.3055	145.49522	7.26863	21.2109
4	16.773	BB	0.3388	200.08087	9.23551	29.1686

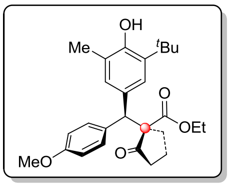

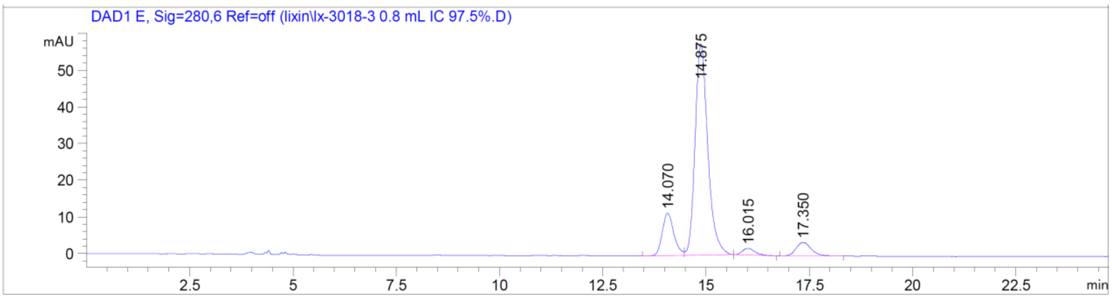

Peak #	RetTime [min]	Type	Width [min]	Area [mAU*s]	Height [mAU]	Area %
1	14.070	BV	0.3024	226.61327	11.57590	14.1997
2	14.875	VB	0.3300	1234.47620	57.61588	77.3528
3	16.015	BB	0.3242	39.75277	1.85338	2.4909
4	17.350	BB	0.3809	95.06054	3.73602	5.9565

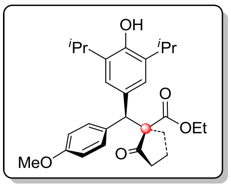
OH
iPr
iPr
O
OEt
MeO
O

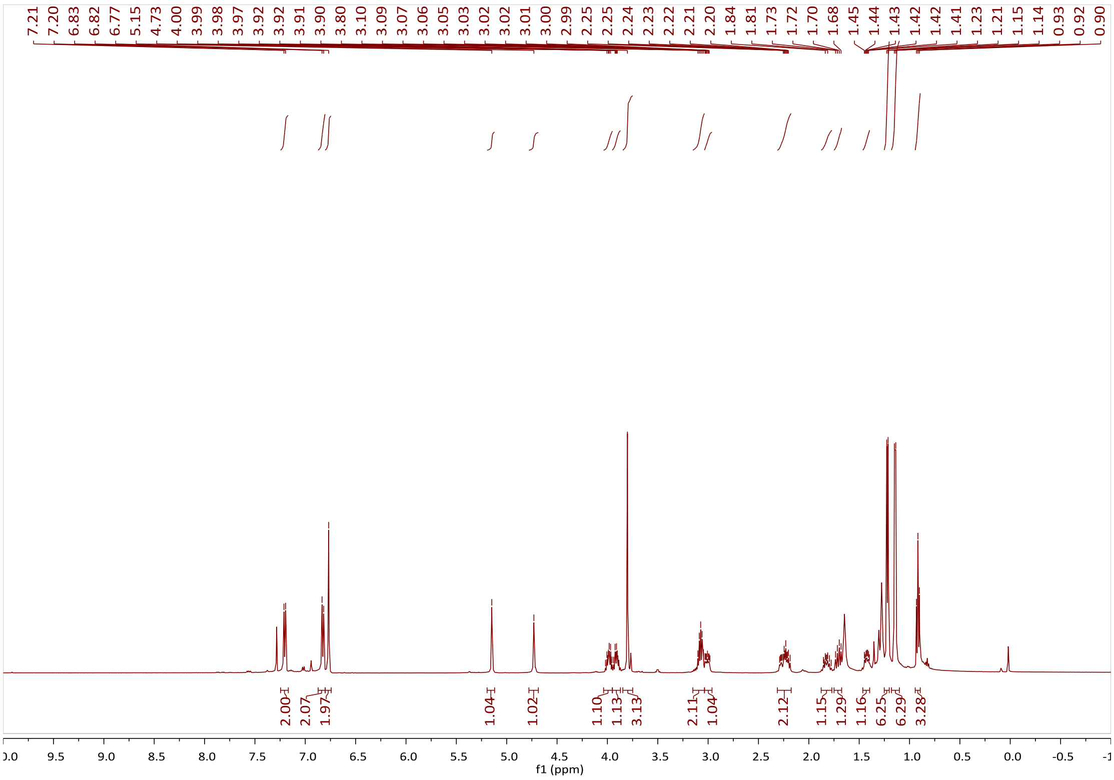
7.21 7.20 6.83 6.82 6.77 5.15 4.73 4.00 3.99 3.98 3.97 3.92 3.92 3.91 3.90 3.80 3.10 3.09 3.07 3.06 3.05 3.03 3.02 3.02 3.01 3.00 2.99 2.25 2.25 2.24 2.23 2.22 2.21 2.20 1.84 1.81 1.73 1.72 1.70 1.68 1.45 1.44 1.43 1.42 1.42 1.41 1.23 1.21 1.15 1.14 0.93 0.92 0.90
2.00 2.07 1.97 1.04 1.02 1.10 1.13 3.13 2.11 1.04 2.12 1.15 1.29 1.16 6.25 6.29 3.28
f1 (ppm)

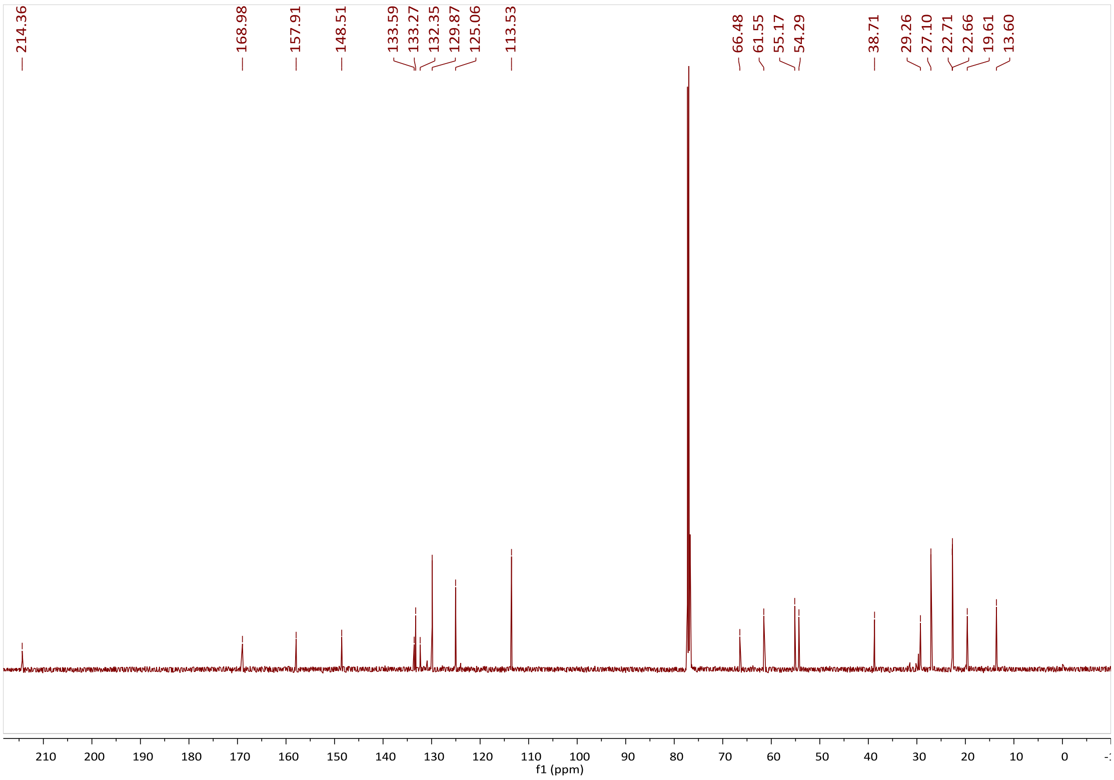
214.36 168.98 157.91 148.51 133.59 133.27 132.35 129.87 125.06 113.53 66.48 61.55 55.17 54.29 38.71 29.26 27.10 22.71 22.66 19.61 13.60
f1 (ppm)

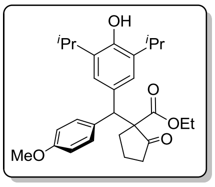

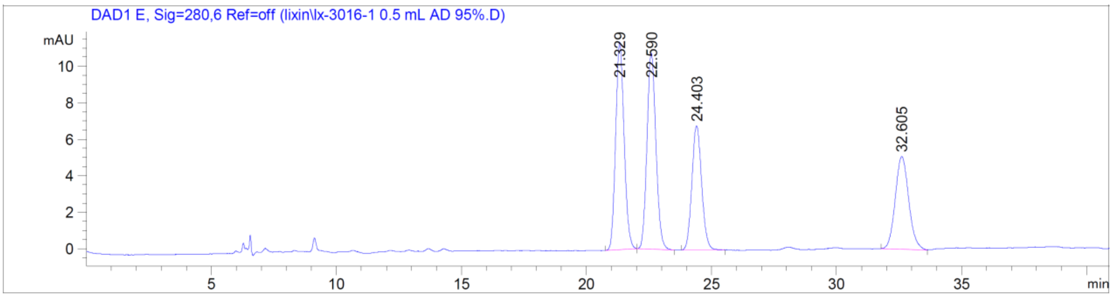

Peak #	RetTime [min]	Type	Width [min]	Area [mAU*s]	Height [mAU]	Area %
1	21.329	BB	0.3597	266.70422	11.36937	29.3375
2	22.590	BB	0.3861	267.35431	10.75305	29.4090
3	24.403	BB	0.4161	185.83469	6.81547	20.4418
4	32.605	BB	0.5546	189.19640	5.07868	20.8116

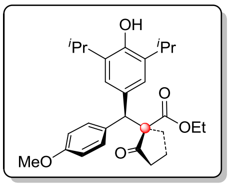

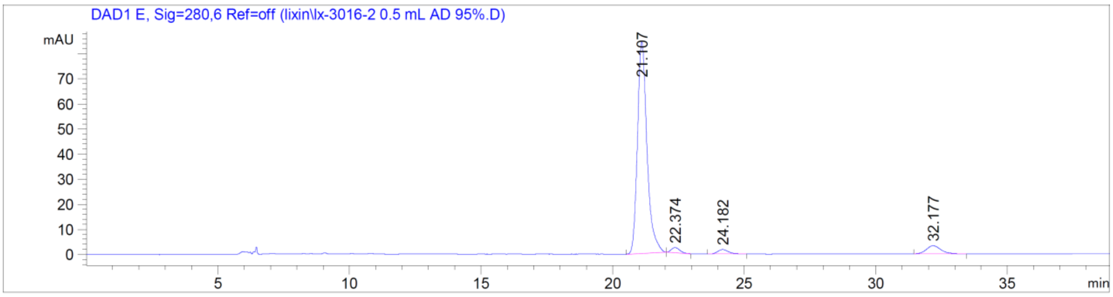

Peak #	RetTime [min]	Type	Width [min]	Area [mAU*s]	Height [mAU]	Area %
1	21.107	BB	0.3848	2131.23779	84.33810	90.5878
2	22.374	BB	0.3396	45.72258	2.08730	1.9434
3	24.182	BB	0.3960	52.60191	1.82620	2.2358
4	32.177	BB	0.5402	123.11397	3.21926	5.2329

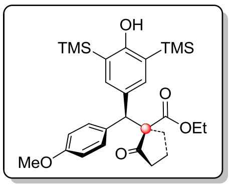
OH
TMS
TMS
O
OEt
MeO
O

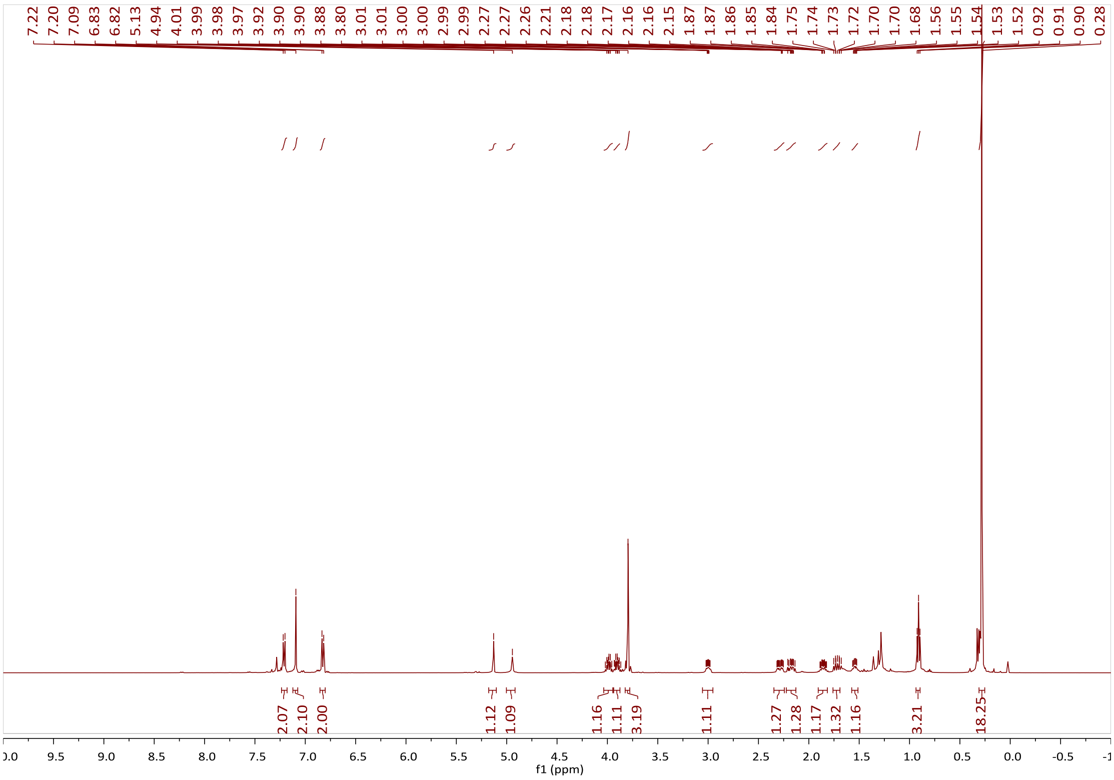
7.22 7.20 7.09 6.83 6.82 5.13 4.94 4.01 3.99 3.98 3.97 3.92 3.90 3.90 3.88 3.80 3.01 3.01 3.00 3.00 2.99 2.99 2.27 2.27 2.26 2.21 2.18 2.18 2.17 2.16 2.16 2.15 1.87 1.87 1.86 1.85 1.84 1.75 1.74 1.73 1.72 1.70 1.70 1.68 1.56 1.55 1.54 1.53 1.52 0.92 0.91 0.90 0.28
2.07 2.10 2.00 1.12 1.09 1.16 1.11 3.19 1.11 1.27 1.28 1.17 1.32 1.16 3.21 18.25
f1 (ppm)

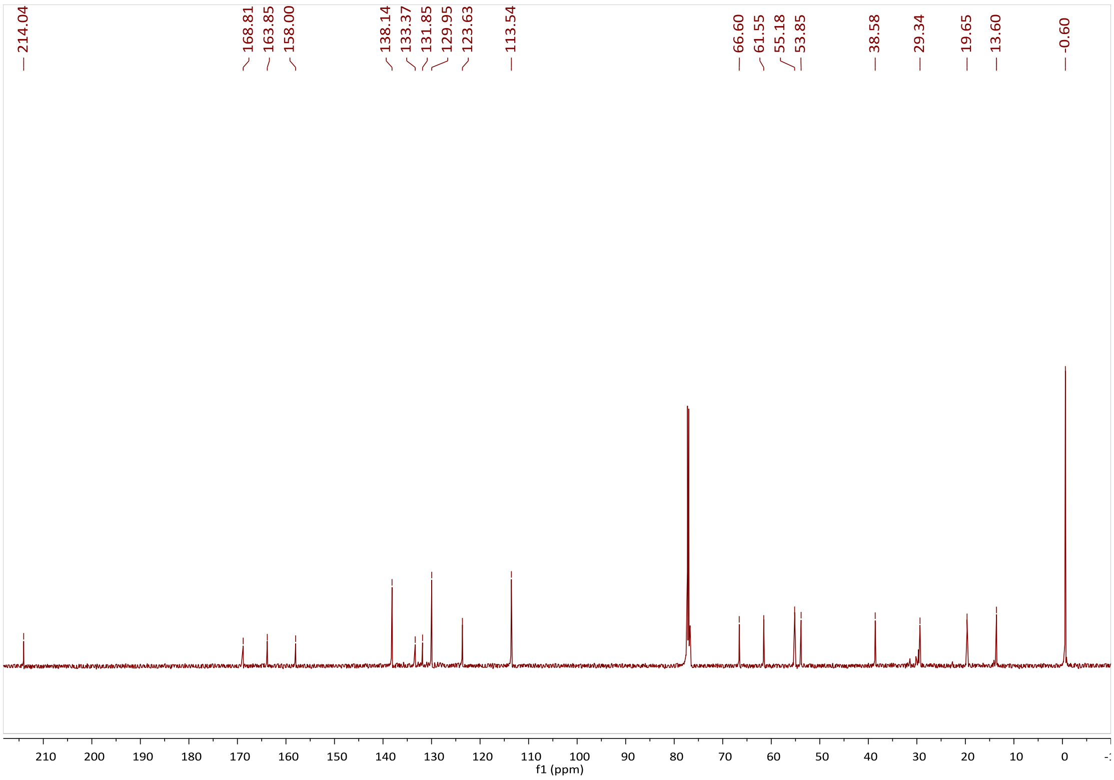
214.04 168.81 163.85 158.00 138.14 133.37 131.85 129.95 123.63 113.54 66.60 61.55 55.18 53.85 38.58 29.34 19.65 13.60 -0.60
f1 (ppm)

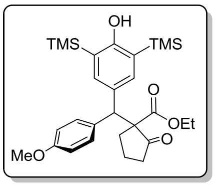

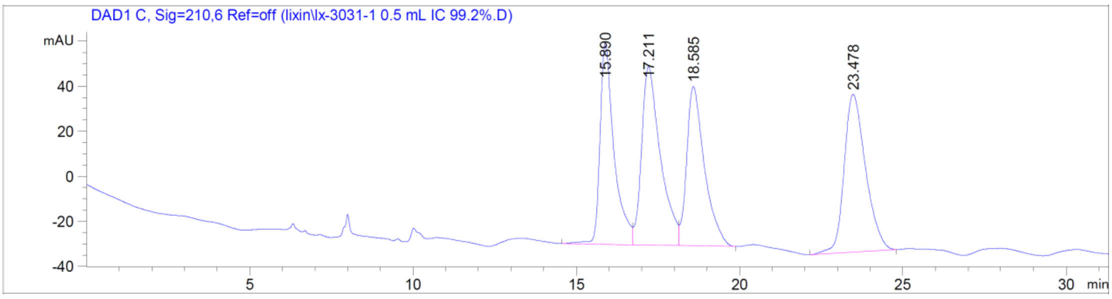

Peak #	RetTime [min]	Type	Width [min]	Area [mAU*s]	Height [mAU]	Area %
1	15.890	BV	0.4355	2681.47925	89.51240	22.7982
2	17.211	VV	0.5831	3185.41650	79.21661	27.0827
3	18.585	VB	0.5609	2683.53516	70.68896	22.8156
4	23.478	BB	0.6946	3211.39624	70.08749	27.3035

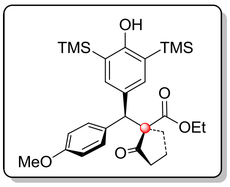

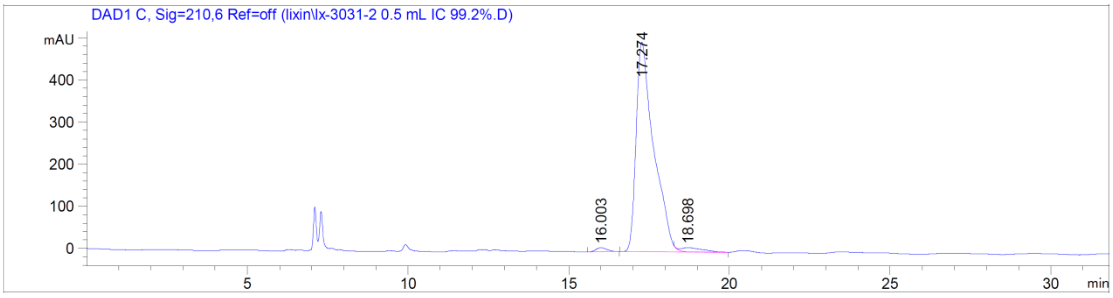

Peak #	RetTime [min]	Type	Width [min]	Area [mAU*s]	Height [mAU]	Area %
1	16.003	BB	0.3792	239.49644	9.79727	1.2189
2	17.274	BV R	0.5380	1.89455e4	497.77588	96.4254
3	18.698	VB E	0.6199	462.83081	9.92772	2.3556

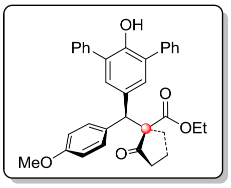
OH
Ph
Ph
O
OEt
O
MeO

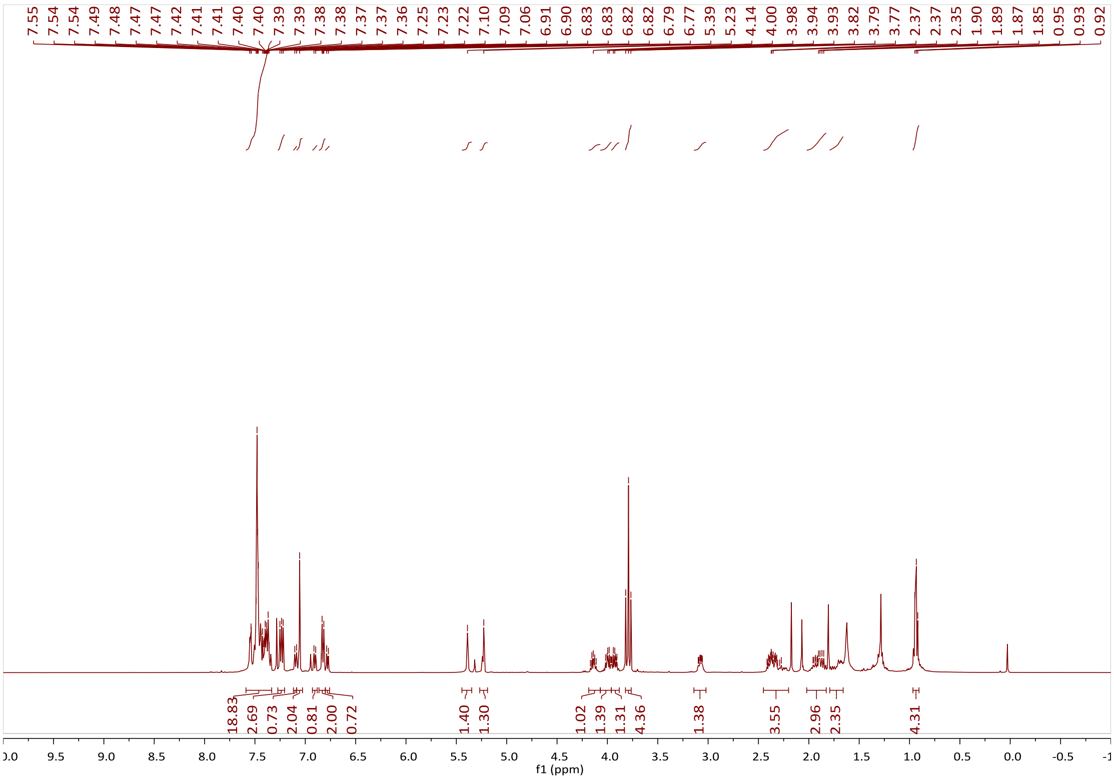
7.55 7.54 7.54 7.49 7.48 7.47 7.47 7.42 7.41 7.41 7.40 7.40 7.39 7.39 7.38 7.38 7.37 7.37 7.36 7.25 7.23 7.22 7.10 7.09 7.06 6.91 6.90 6.83 6.83 6.82 6.82 6.79 6.77 5.39 5.23 4.14 4.00 3.98 3.94 3.93 3.82 3.79 3.77 2.37 2.37 2.35 1.90 1.89 1.87 1.85 0.95 0.93 0.92
18.83 2.69 0.73 2.04 0.81 2.00 0.72 1.40 1.30 1.02 1.39 1.31 4.36 1.38 3.55 2.96 2.35 4.31
f1 (ppm)

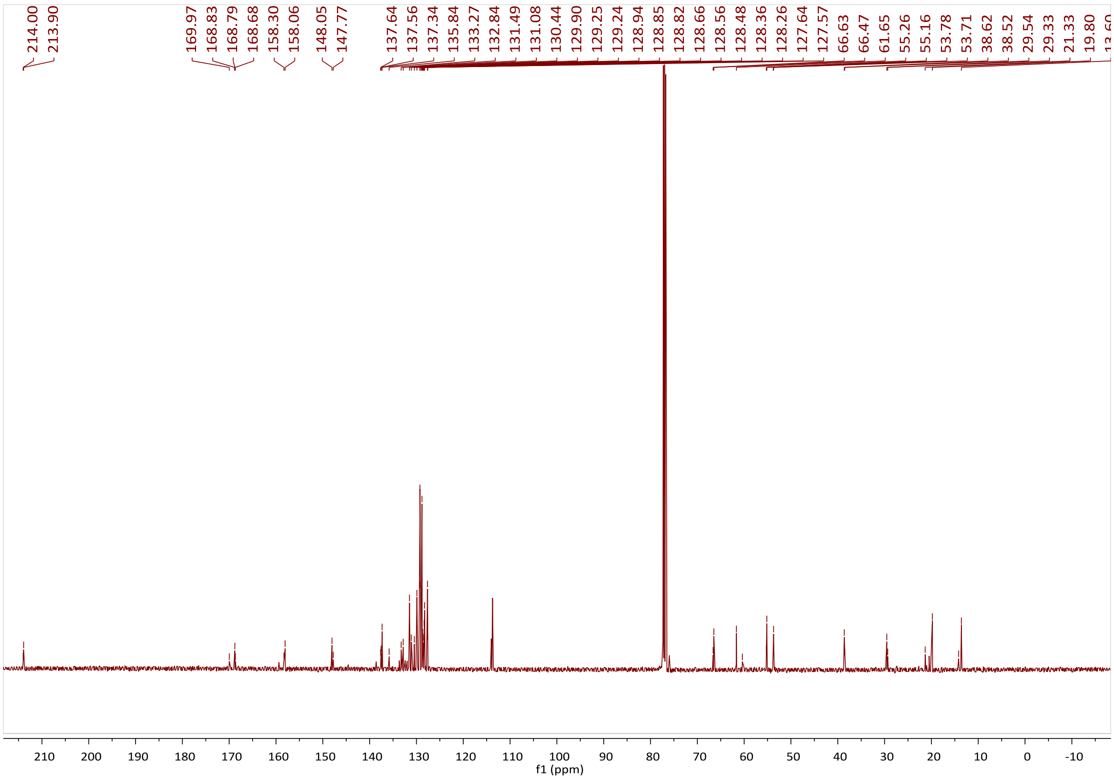
214.00 213.90 169.97 168.83 168.79 168.68 158.30 158.06 148.05 147.77 137.64 137.56 137.34 135.84 133.27 132.84 131.49 131.08 130.44 129.90 129.25 129.24 128.94 128.85 128.82 128.66 128.56 128.48 128.36 128.26 127.64 127.57 66.63 66.47 61.65 55.26 55.16 53.78 53.71 38.62 38.52 29.54 29.33 21.33 19.80
f1 (ppm)

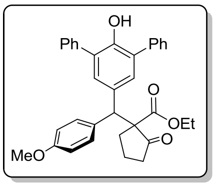

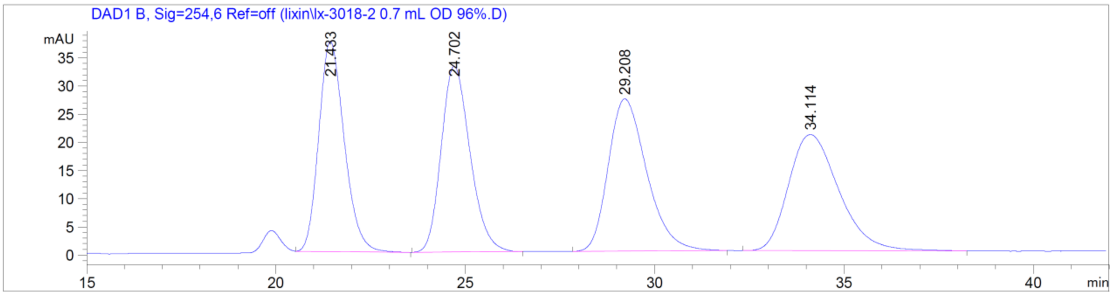

Peak #	RetTime [min]	Type	Width [min]	Area [mAU*s]	Height [mAU]	Area %
1	21.433	BB	0.7215	1744.09412	37.30320	23.8384
2	24.702	BB	0.8057	1751.29395	32.97206	23.9369
3	29.208	BB	1.0680	1910.73108	27.05408	26.1161
4	34.114	BB	1.2411	1910.18787	20.65232	26.1086

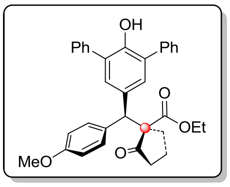

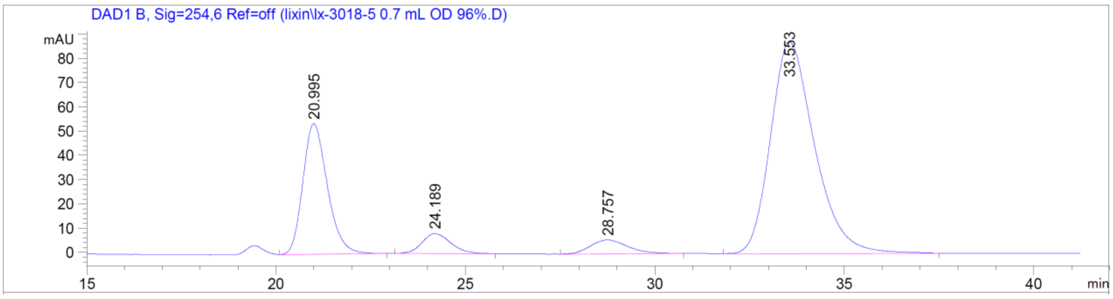

Peak #	RetTime [min]	Type	Width [min]	Area [mAU*s]	Height [mAU]	Area %
1	20.995	BB	0.6908	2420.13159	53.80505	23.5019
2	24.189	BB	0.7666	434.43671	8.22039	4.2188
3	28.757	BB	0.8613	395.33597	5.75625	3.8391
4	33.553	BB	1.2093	7047.69971	87.16129	68.4402

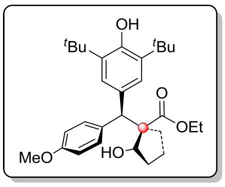
OH
tBu
tBu
O
OEt
MeO
HO

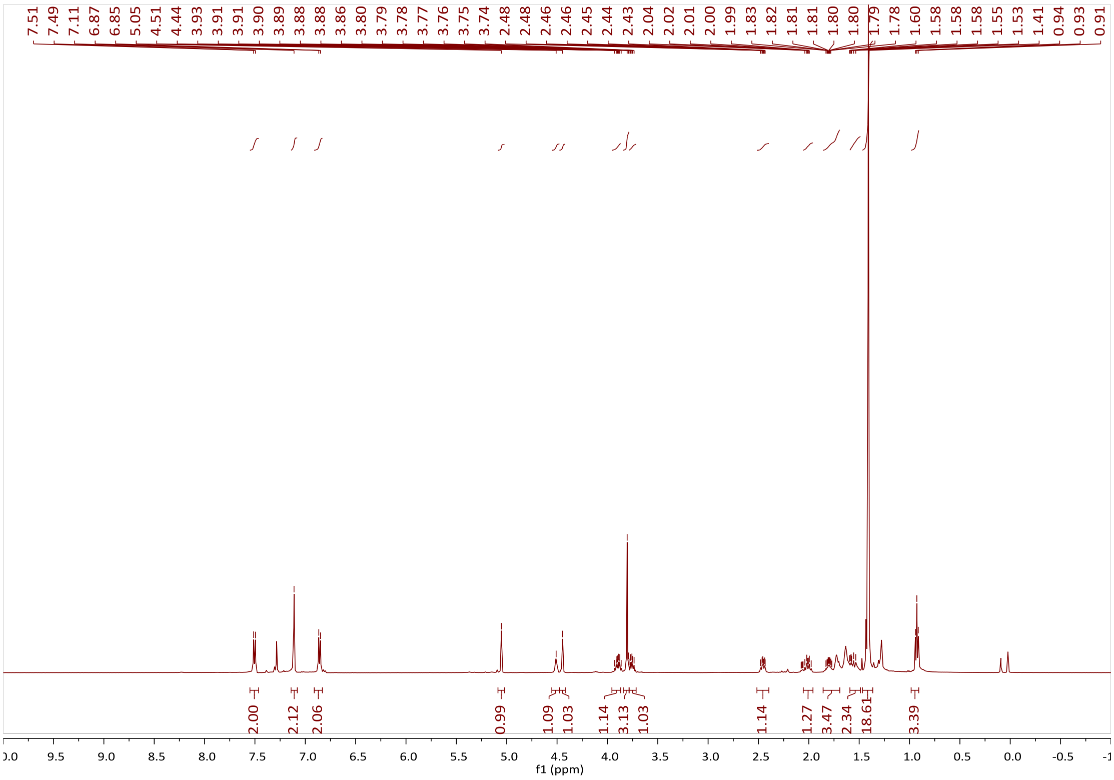
f1 (ppm)

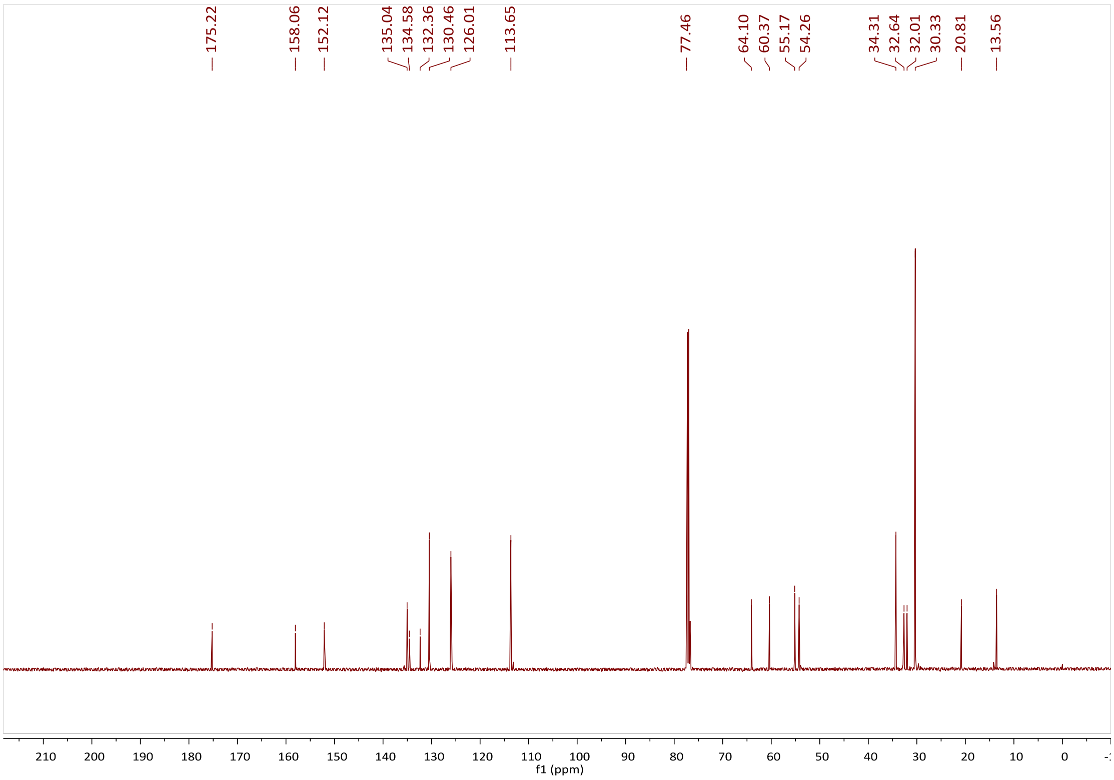
175.22
158.06
152.12
135.04
134.58
132.36
130.46
126.01
113.65
77.46
64.10
60.37
55.17
54.26
34.31
32.64
32.01
30.33
20.81
13.56
f1 (ppm)

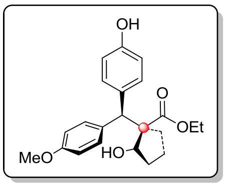
OH
O
OEt
MeO
HO

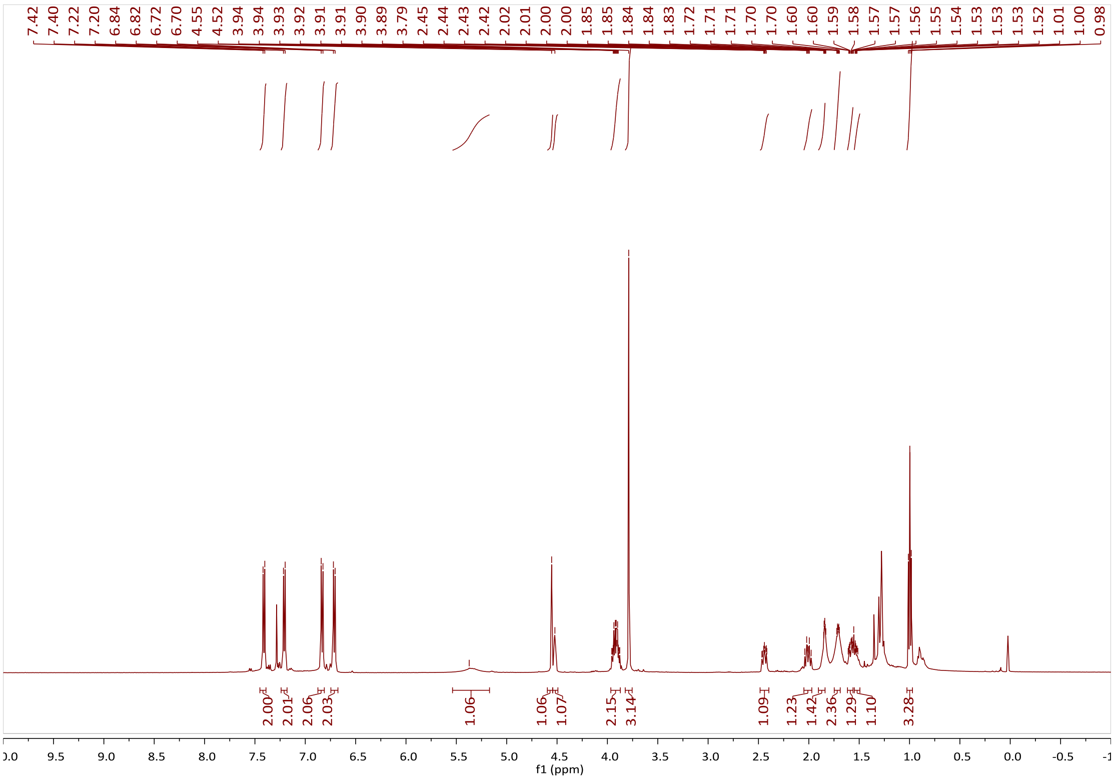
7.42 7.40 7.22 7.20 6.84 6.82 6.72 6.70 4.55 4.52 3.94 3.94 3.93 3.92 3.91 3.91 3.90 3.89 3.79 2.45 2.44 2.43 2.42 2.02 2.01 2.00 2.00 1.85 1.85 1.84 1.84 1.83 1.72 1.71 1.71 1.70 1.70 1.60 1.60 1.59 1.58 1.57 1.57 1.56 1.55 1.54 1.53 1.53 1.53 1.52 1.01 1.00 0.98
2.00 2.01 2.06 2.03 1.06 1.06 1.07 2.15 3.14 1.09 1.23 1.42 2.36 1.29 1.10 3.28
f1 (ppm)

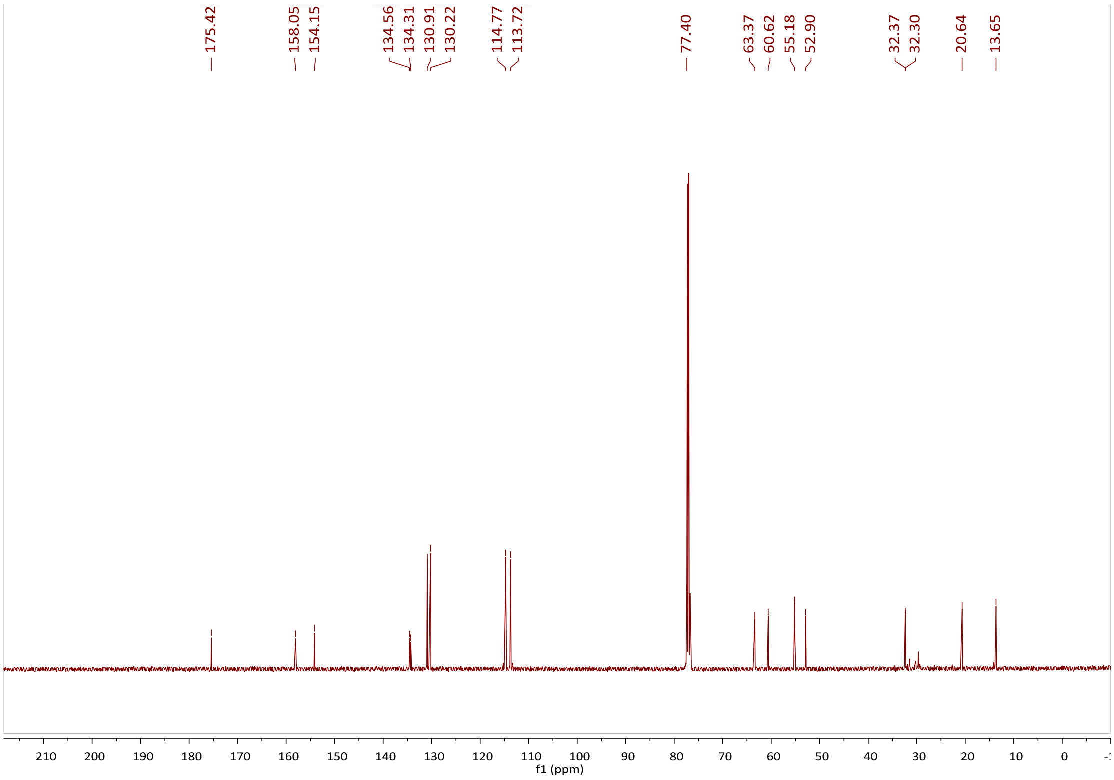
175.42 158.05 154.15 134.56 134.31 130.91 130.22 114.77 113.72 77.40 63.37 60.62 55.18 52.90 32.37 32.30 20.64 13.65
f1 (ppm)

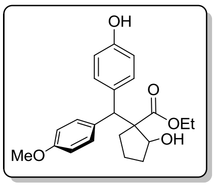

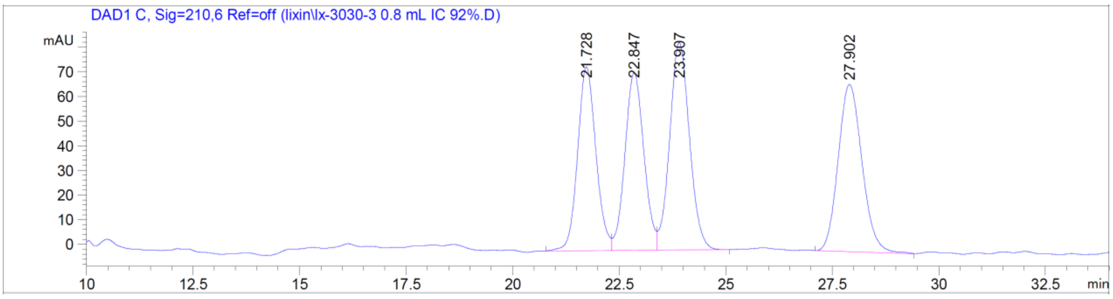

Peak #	RetTime [min]	Type	Width [min]	Area [mAU*s]	Height [mAU]	Area %
1	21.728	BV	0.4567	2229.91870	74.59883	22.7006
2	22.847	VV	0.4795	2232.86426	72.06480	22.7306
3	23.907	VB	0.4992	2724.71289	84.27470	27.7376
4	27.902	BB	0.6047	2635.66919	67.91964	26.8312

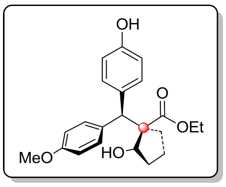

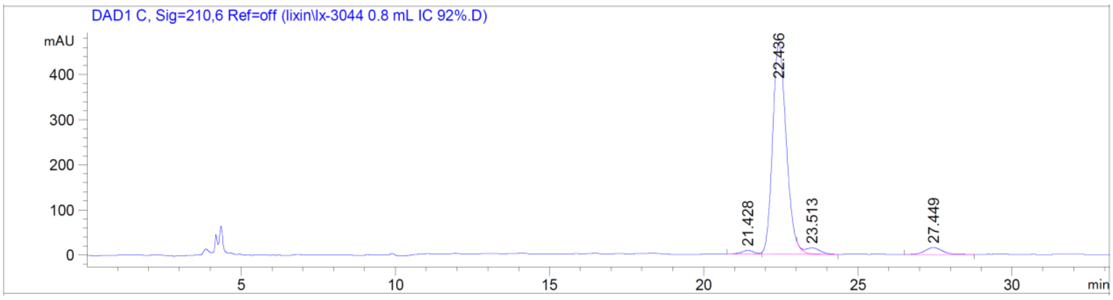

Peak #	RetTime [min]	Type	Width [min]	Area [mAU*s]	Height [mAU]	Area %
1	21.428	BV E	0.4091	232.92355	8.40991	1.4785
2	22.436	VV R	0.4794	1.44816e4	467.43732	91.9246
3	23.513	VB E	0.4751	418.72577	13.16331	2.6579
4	27.449	BB	0.6079	620.52545	15.27567	3.9389

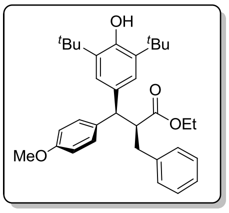
OH
tBu
tBu
O
OEt
MeO

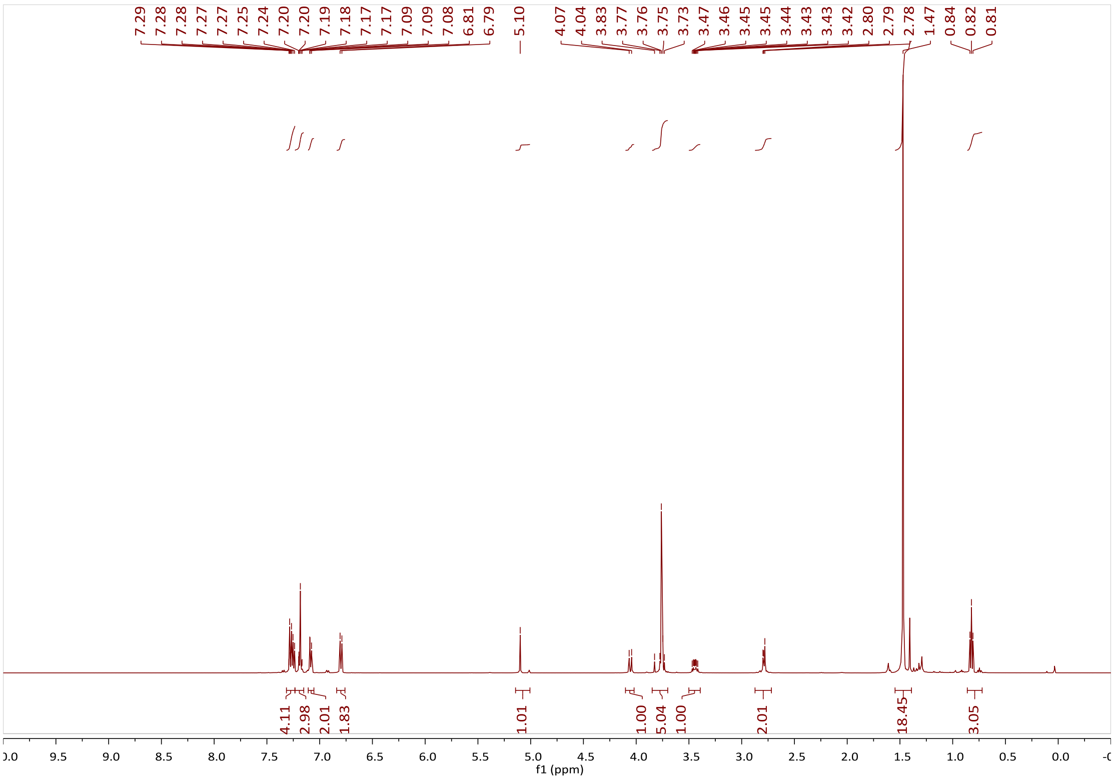
7.29 7.28 7.28 7.27 7.27 7.25 7.24 7.20 7.20 7.19 7.18 7.17 7.17 7.09 7.09 7.08 6.81 6.79
5.10
4.07 4.04 3.83 3.77 3.76 3.75 3.73 3.47 3.46 3.45 3.45 3.44 3.43 3.43 3.42 2.80 2.79 2.78 1.47 0.84 0.82 0.81
4.11 2.98 2.01 1.83 1.01 1.00 5.04 1.00 2.01 18.45 3.05
f1 (ppm)

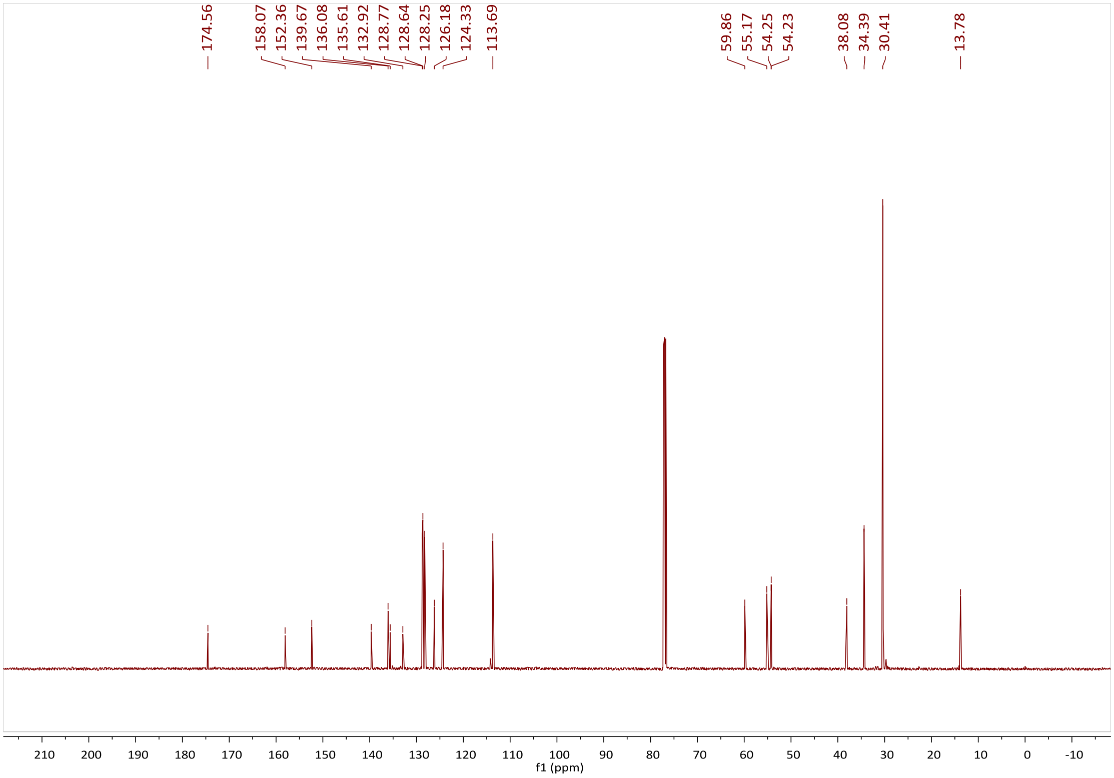
174.56
158.07 152.36 139.67 136.08 135.61 132.92 128.77 128.64 128.25 126.18 124.33 113.69
59.86 55.17 54.25 54.23
38.08 34.39 30.41
13.78
f1 (ppm)

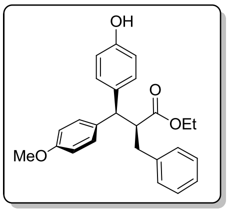
OH
O
OEt
MeO

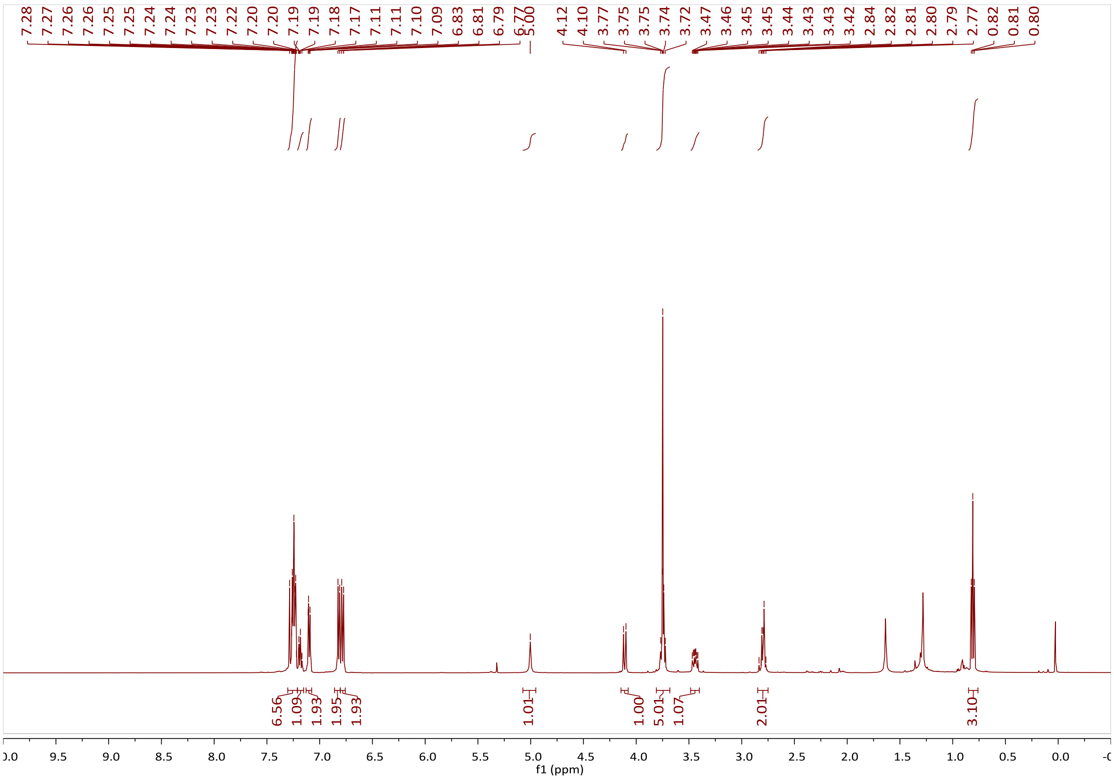

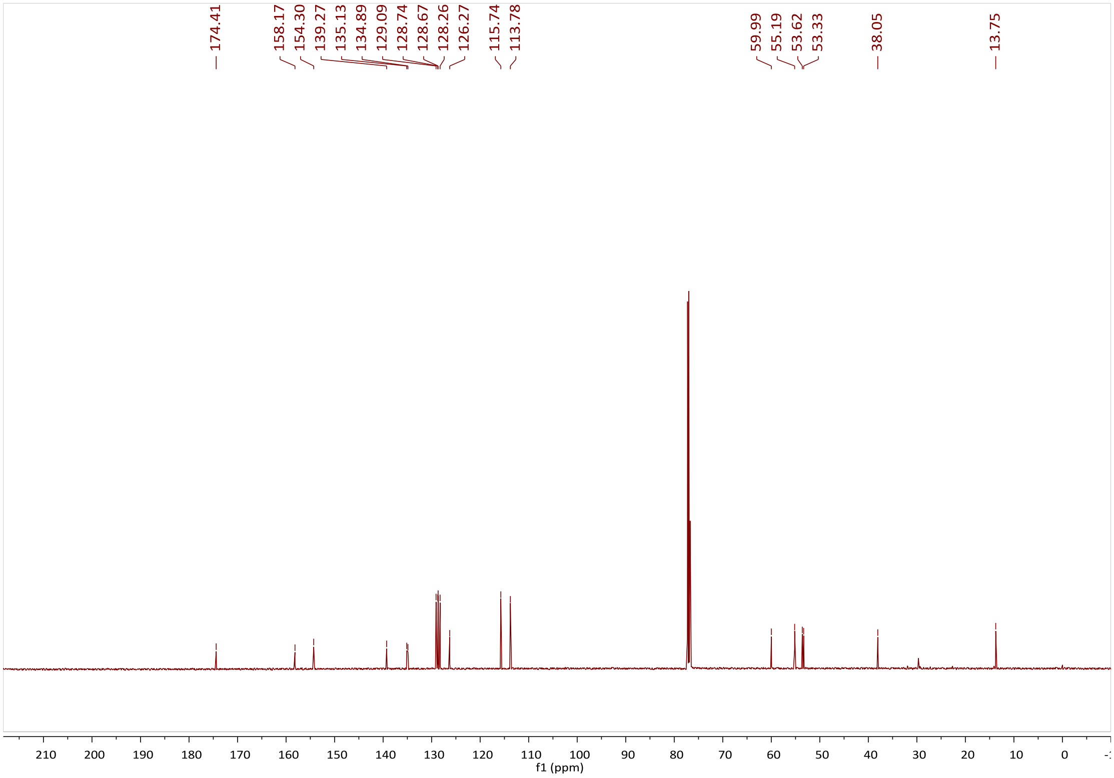

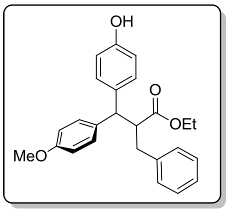

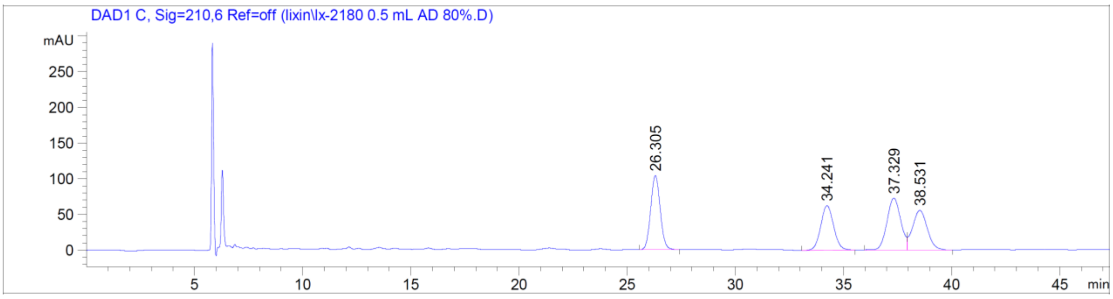

Peak #	RetTime [min]	Type	Width [min]	Area [mAU*s]	Height [mAU]	Area %
1	26.305	BB	0.4749	3180.76172	103.98998	27.3097
2	34.241	BB	0.6380	2606.00732	62.56393	22.3749
3	37.329	BV	0.6819	3215.57788	72.72440	27.6086
4	38.531	VB	0.7099	2644.67651	55.70596	22.7069

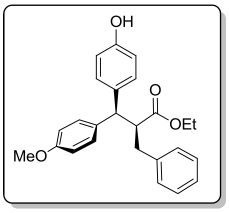

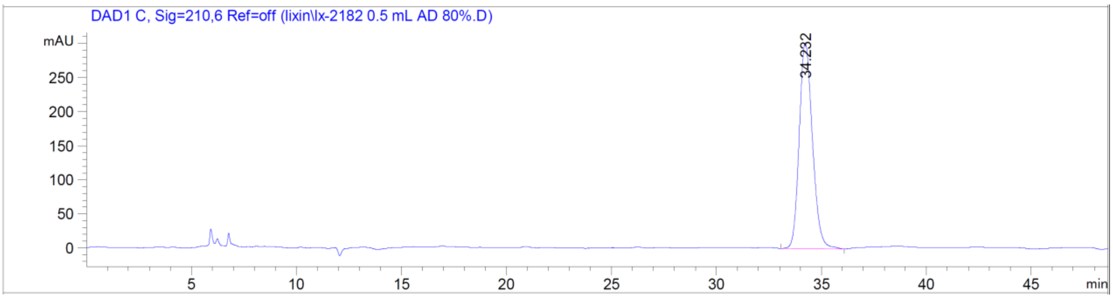

Peak #	RetTime [min]	Type	Width [min]	Area [mAU*s]	Height [mAU]	Area %
1	34.232	BB	0.6766	1.31845e4	301.27390	100.0000
